# Supplementary material for: A diminutive new basilosaurid whale reveals the trajectory of the cetacean life histories during the Eocene
Source: Commun Biol. 2023 Aug 10;6:707. doi: 10.1038/s42003-023-04986-w (PMC10415296; doi:10.1038/s42003-023-04986-w)
Supplement: Supplementary file 2 — Supplementary Information [file 42003_2023_4986_MOESM2_ESM.pdf]

Supplementary Information

A diminutive new basilosaurid whale reveals the trajectory of the cetacean life histories during the Eocene

Mohammed S. Antar[1,2,*], Abdullah S. Gohar[1,3], Heba El-Desouky[1,4], Erik R. Seiffert[5], Sanaa El-Sayed[1,6], Alexander G. Claxton[7] and Hesham M. Sallam[1,3]

[1] Mansoura University Vertebrate Paleontology Center (MUVP), Mansoura University, Mansoura, 35516, Egypt.
[2] Nature Conservation Sector, Egyptian Environmental Affairs Agency, Cairo, 11728, Egypt.
[3] Institute of Global Health and Human Ecology (I-GHHE), School of Sciences and Engineering, American University in Cairo, New Cairo, 11835, Egypt.
[4] Department of Geology, Faculty of Science, Mansoura University, Mansoura, 35516, Egypt
[5] Department of Integrative Anatomical Sciences, Keck School of Medicine of USC, University of Southern California, Los Angeles, CA, 90033, USA
[6] Department of Earth and Environmental Sciences, University of Michigan, Ann Arbor, MI, 48109, USA.
[7] Department of Anatomy and Cell Biology, Oklahoma State University Center for Health Sciences, Tulsa, OK, 74107, USA
[*] Corresponding author: m.s.antar@mans.edu.eg

Contents

Supplementary Note 1: Comparison and Extended Diagnosis
Supplementary Note 2: Locality and Stratigraphy
Supplementary Methods
Supplementary Results
A. Extended Description
B. Phylogenetic Analyses
Supplementary Figures
Supplementary Tables
Supplementary References

Supplementary Note 1: Comparison and Extended Diagnosis

Tutcetus differs from *Pontogeneus peruvianus* (MNHN.F.PRU10) and *Supayacetus muizoni* (MUSM 1465) in having equal sizes of the medial and lateral posterior prominences in the auditory bulla. *Tutcetus* further differs from *Dorudon atrox*[1], *Pontogeneus peruvianus* (MNHN.F.PRU10), *Zygorhiza*, *Chrysocetus*, and *Saghacetus* [similar to *Ocucajea picklingi* (MUSM 1442)] in having the posterior edge of the postorbital process oriented at approximately 90° to the sagittal crest and in having an anterodorsal orientation of the supraoccipital shield. *Ocucajea picklingi* (MUSM 1442) has a relatively wider supraorbital region than does *Tutcetus*, and further differs from *Tutcetus* in having diastemata between its lower molars. *Tutcetus* differs from *Ancalecetus* and *Dorudon atrox* (but not *Zygorhiza kochii*[2]) in having convex articular facets on the atlas for the axis. *Tutcetus* lacks the pinching of the nuchal crest just dorsal to the occipital crest that is seen in *Saghacetus osiris*[3]. In contrast to *Chrysocetus* (SCSM 87.195) and *Saghacetus osiris*[3], which have a single-rooted P_1, the left dentary of MUVP 501 preserves a double-rooted P_1, similar to *Dorudon atrox*[1] and *Zygorhiza kochii*[2]. *Tutcetus* differs from *Antaecetus*[4] in being smaller, having more gracile teeth, and in having a different configuration of the accessory cusps on premolars. Furthermore, the upper molars of *Tutcetus* differ from those of *Antaecetus*[4] in lacking distolingual expansion. The skull of *Tutcetus* further differs from *Antaecetus*[4] in having a relatively narrow constriction of the rostrum. In contrast to *Saghacetus osiris*[5], which has narrow transverse processes on C1, the atlas vertebra of MUVP 501 shows wide transverse processes, similar to *Dorudon atrox*[1] and *Zygorhiza kochii*[2].

Supplementary Note 2: Locality and Stratigraphy

The holotype of *Tutcetus rayanensis* (gen. et sp. nov.) was found in an indurated limestone block near the Upper Lake in the Wadi El-Rayan area (29°15' N, 30°25' E), a locality 40 km NE of the Wadi El-Hitan World Heritage Site in Fayum, Egypt. The block derives from the lowest levels of the Sath El-Hadid Formation, several meters above the underlying Midawara Formation (Supplementary Figure 1). The nummulitic limestone of the Sath El-Hadid Formation contains variable sizes of *Nummulites* and covers several mesas (flat-topped small hills) in the Wadi El-Rayan area. The Sath El-Hadid Formation conformably overlies the Midawara Formation with a gradual contact between the uppermost glauconitic sandy brown limestone of the Midawara Formation and the basal snow-white marly limestone of the Sath El-Hadid Formation[6]. Bryozoan limestone from the top of the Sath El-Hadid Formation is in sharp contact with the reddish limestone of the upper El-Gharaq Formation, which is laden with *Nummulites lyelli*[7]. This contact with the El-Gharaq Formation is distinguished by the initial appearance of dark and light brown shales above the snow-white nummulitic limestone[6]. The Sath El-Hadid Formation has been assigned to the earliest part of the late middle Eocene (earliest Bartonian) (ca. 41 Ma)[8]. Besides *Tutcetus*, the Sath El Hadid Formation furnished other vertebrate fossils, represented by sirenians, and selachian fish fossils[9].

Stratigraphically, the Gehannam Formation of the uppermost Bartonian (middle Eocene) and lowermost Priabonian (late Eocene)[8,10], is one of the oldest geological formations that produced basilosaurids in the Fayum Depression and produced *Dorudon atrox*[1], *Masracetus markgrafi*[5], and *Basilosaurus isis*[8]. The overlying Birket Qarun Formation of the early Priabonian age is one of the most geological formations in Africa that has been extensively prospected for cetaceans[9-10]. It produced four archaeocetes which are known from partial to complete skeletons; from smallest to largest, *Ancalecetus simonsi*[11], *Dorudon atrox*[1], *Masracetus markgrafi*[5,12], and *Basilosaurus*

isis[12,14]. One or two additional cetaceans are represented by vertebrae that seem to differ from all others, but these are not yet known from diagnostic specimens. Five to six species is the greatest archaeocete diversity known from any single formation in Egypt. The overlying Qasr El Sagha Formation of the late Priabonian age has two named archaeocetes. These are, from smaller to larger, *Saghacetus osiris* (the first cetacean named from Egypt)[3,15] and *Stromerius nidensis*[5]. Both were probably totally aquatic like *Dorudon*, albeit neither is well enough known postcranially to allow for interpretation of their distinctions from *Dorudon* in terms of swimming locomotion.

Supplementary Methods

Matrices of the phylogenetic analysis:
The data matrix from Gohar et al.[16] was modified in Supplementary Data (1) by adding the new basilosaurid described here, *Tutcetus rayanensis*, the Egyptian remingtonocetid *Rayanistes afer*, and the Egyptian protocetid *Aegicetus gehennae*. *Cynthiacetus* was replaced with *Pontogeneus* in both Supplementary Data (1) and Supplementary Data (2) as we followed Gingerich[17] in synonymizing the two names. Additionally, we used *Pachycetus* as the generic name for taxa that were formerly included in *Platyosphys* and *Basilotritus* in Supplementary Data (1) in accordance with Gingerich et al.[4]. Considering the suggested taxonomy and synonyms provided by Gingerich et al.[4], we altered *Platyosphys einori* to *Pachycetus paulsonii*, added the species *P. wardii*, and amended the scores for both species and further changed *Platyosphys aithai* to *Antaecetus aithai* and revised its scoring in the matrix. As for the Characters used for phylogenetic analyses, we added 5 additional characters to the matrix of Gohar et al.[16] with some coding changes. As a result, 47 taxa with 195 characters (37 ordered) were incorporated into Supplementary Data (1). Characters 1–190 are from Gohar et al.[16], and readers are referred to that study for character descriptions and citations. The newly added taxa and characters (i.e., 191-195) are described below.

191. **Acetabular notch:**
 0. open
 1. narrow (nearly closed)
 2. closed
 (Character 166 Kassegne et al.[18] *– based on Bebej et al.*[19]*)*

192. **Femoral head orientation:**
 0. perpendicular to the shaft
 1. about 45° from the shaft
 2. in the continuation of the shaft
 (Character 167 Kassegne et al.[18]*)*

193. **Pachyosteosclerotic vertebrae:**
 0. absent
 1. present
 (This study – based on Gingerich et al.[4]*)*

194. **Pachyosteosclerotic ribs:**
 0. absent

1. present
(This study – based on Gingerich et al.[4]*)*

195. **Rib articulations with the thoracic vertebrae:**
0. synovial
1. Cartilaginous and ligamentous connective tissue
(This study – based on Gingerich et al.[4]*)*

The data matrix from Martínez-Cáceres et al.[20] was simply expanded by implanting *Tutcetus rayanensis* (MUVP 501). As a result, 32 taxa with 101 characters (95 ordered) were incorporated into Supplementary Data (2). This matrix seeks to establish the relationships of MUVP 501 among the Basilosauridae and focuses on the relationships of the more derived archaeocetes in general and on the Archaeoceti-Neoceti transition.

We prefer Bayesian methods over parsimony to estimate phylogenetic topology because the former can combine various types of data (such as temporal and stratigraphic information, both parsimony informative and uninformative characters, rates of morphological evolution, and an underlying model of evolution) into a single analysis. Furthermore, under various circumstances, Bayesian methods can outperform parsimony[21-22]. The tip-dating trees obtained reveal topological variations between the two matrices utilized in this investigation, which are probably caused by assumptions about the evolution of morphological characters, the uncertainty brought on by missing data, and a lack of taxon overlap.

Supplementary Results

A. Extended Description

The cranium was found upside down in the field.
Premaxilla*:* A 10 cm part of the premaxilla is detached from the rest of the cranium. Between the alveoli of the incisors, the premaxilla has deep laterally opened embrasure pits that receive the crowns of the lower incisors.
Nasal: The roof of the narial cavity is represented by the nasals, which are nearly complete in MUVP 501. While the nasals articulate with the maxilla and frontal laterally, they meet medially along the midline. It is unclear where the anterior nasals end in relation to the tooth row because the ventral surface of the rostrum is severely damaged.
Vomer: The anterior 30 mm of the vomer can be seen from the ventral surface with a height of around 23 mm. When the vomeral wings meet in the middle, a sharp ridge arises, which serves as a septum, separating the two narial passages. This division makes the vomer triangular in cross-section.
Presphenoid: The presphenoid portion of MUVP 501's cranium is a robust midline element that is 87 mm in length. It had an anterior breadth of about 18 mm and widens posteriorly to a width of about 40 mm, where it had cartilaginous contact with the sphenoid. A thin layer of frontal bone covers most of the lateral sides of the presphenoid.
Basisphenoid: Anteriorly, at its connection with the presphenoid, the basisphenoid has a pentagonal cross-section, measuring approximately 40 mm wide. It widens posteriorly as it approaches the

cerebral cavity, where it was supposed to touch the missing basioccipital and make the floor of the braincase.

Alisphenoid: In the lateral view of the skull of MUVP 501, the alisphenoid can be seen as a part of the anterior medial wall of the temporal fossa. The sphenoparietal suture, which rises anteriorly and dorsally, connects the alisphenoid to the frontal anteriorly, the parietal, and the squamosal posteriorly.

Frontal: The frontal articulates posteriorly with the parietal and anteriorly with the nasal and the maxilla. The frontal (supraorbital) shield is formed by the frontal expanding laterally. The frontal shield has a frontal foramen on its posteroventral border. The medial borders of the frontals divide them along the midline, while the posterior edge of the maxillae bordered them anteriorly.

Parietal: The dorsolateral portion of the cranium is formed by the parietal. The parietooccipital suture runs posteroventrally until it reaches the squamosal. Left and right parietals articulate along the midline to form a strong and prominent sagittal crest that runs from the apex of the nuchal crest posteriorly and continues to the frontoparietal suture anteriorly.

Occipital: The exoccipital portion of the occiput is missing on the right side.

Squamosal: The posterolateral wall of the cranium is formed by the squamosal. It contacts anterodorsally with the parietal, anteriorly with the alisphenoid, posteriorly with the exoccipital, and medially with the anterior processes of the periotic. The squamosal forms the lateral wall of the braincase and the glenoid and squamosal fossae. Much of the medial surface is in contact with the anterior process of the periotic.

Tympanic bulla: A piece of the involucrum of the left tympanic bulla is still embedded in the basicranium, while a nearly complete isolated right tympanic bulla is preserved, and therefore the description is based on the right bulla. *Tutcetus* has a slightly convex medial edge on its tympanic bulla, in contrast to protocetids, which have a concave medial margin[23-25]. The lateral posterior eminence is longer and more pronounced than the medial posterior eminence. The medial eminence is the posterior part of the involucrum of the bulla. The tympanic cavity is dorsally open, narrowest posteriorly, and widens toward the anterior border of the bulla. The involucrum is convex on the medial, posterior, and dorsal sides. The involucrum is divided into two halves, one more bulbous on the posterior side and the other relatively low on the anterior side. The convexity of the posterior half is higher than the anterior part.

Mandible: The mandibular condyle of the left mandible is not preserved. The dorsal surface of the dentary continues to extend upward toward the apex of the coronoid process. The edge of the coronoid process turns ventrally again and curves just slightly anteriorly, leaving the coronoid process projecting both dorsally and posteriorly. The posterior edge of the mandible continues ventrally, forming the mandibular notch, between the coronoid process and the mandibular condyle, which is just ventral to the lower border of the coronoid process. The articular surface of the right condyle is generally subtriangular in dorsal view and its surface is broadly convex. The entire articular surface is angled at about 45° to the horizontal ramus, sloping down and back. The condyloid crest arises from the mandibular condyle and extends anteriorly along the lateral face of the dentary to around P_4.

Dentition

dC_1: While the left deciduous lower canine (dC_1) is exposed, the right one is unexposed but can be distinguished using the CT scan. Hence, the left lower deciduous canine is used as the basis for the description. There are no well-developed ridges or cingula on the crown.

Upper posterior premolars (P^{3-4}): Both the upper posterior premolars (P^{3-4}) have a distolingual expansion on the distal root and crown, a remnant of the protocone that is present in non-basilosaurid archaeocetes.
Upper molars (M^{1-2}): The upper molars are much smaller than the preserved upper premolars.
Lower incisors (I_{2-3}): The left and right third incisors both have an angle of about 150° between the longitudinal axis of the root and the crown, while the left second incisor has an angle of 140°.
Lower second premolar (P_2): The crown is straight and triangular. There is a 16 mm diastema separating P_2 from the posterior lower premolars.
Lower posterior premolars (P_{3-4}): The mesial side of the left third lower premolar is severely damaged. The enamel is smooth.

Hyoid apparatus
Stylohyal: MUVP 501 has a 105-mm long and 7.5-mm-wide stylohyal.
Thyrohyal: The thyrohyal is estimated to be 62 mm long, shorter than the stylohyal. The proximal end is much wider (15 mm) than the distal, nearly cylindrical end. The thyrohyal narrows (8 mm) posterior to this wide end, forming the almost cylindrical and almost straight thyrohyal body.

Vertebra
Atlas (C1): The atlas, the first cervical vertebra (C1), does not have a vertebral body and is similar to those of other basilosaurids[1]. While the ventral arch presents two highly concave articular foveae cranially that articulate with the occipital condyles of the skull, it has a broad convex articular surface caudally that receives the odontoid process of the axis. The ventral arch extends laterally and caudally, forming the transverse process. The articular surfaces are connected ventrally and separated dorsally by a wide supracondylar notch.

B. Phylogenetic Analyses

Bayesian tip-dating analysis of Supplementary Data (1): Included in the main manuscript (Phylogenetic relationships).
Our phylogenetic analysis of Supplementary Data (1) demonstrates a notable affinity between pachycetines and Neoceti. However, the phylogenetic position of pachycetines remains somewhat uncertain due to the limited availability of well-preserved specimens. Pachycetines exhibit some basal traits among basilosaurids, such as relatively thicker posterior mandible walls and primitive auditory region anatomy. Nevertheless, their affinity with Neoceti may arise from specialized adaptations for utilizing low-frequency sounds, similar to those found in Miocene and extant baleen whales[26]. Our results highlight the shared anatomical features between pachycetines and both early and modern Neoceti[27]. For instance, some pachycetines (e.g., GMTSNUK 2638) (Supplementary Table 1) display an anteriorly inclined supraoccipital shield, an autapomorphy of Neoceti. Furthermore, the maxillae diverge from the typical basilosaurid morphology[1], bearing resemblance to the earliest mysticete, *Mystacodon selenensis*[28-29]. The tooth row also terminates more anteriorly than in basilosaurids such as *Pontogeneus peruvianus*[23] and *Dorudon atrox*[1], with the alveolus for the rearmost tooth situated directly ventral to the lacrimal canal—similar to *Mystacodon selenensis*[28]. These shared features offer persuasive evidence for an evolutionary relationship between pachycetines and Neoceti. However, additional, more comprehensive specimens are required to ascertain the relationship between these groups.

Bayesian tip-dating analysis of Supplementary Data (2): The second, and taxonomically more restricted, BTD analysis recovered *Georgiacetus* as the most crownward protocetid and identified a strongly supported (PP = 1.0) Pelagiceti clade. Within the Pelagiceti clade, the analysis recovered Basilosauridae as paraphyletic, with *Tutcetus* recovered as the most basal basilosaurid. The next-most crownward branch within the basilosaurid grade is a moderately-supported (PP = 0.65) clade that includes the North American *Chrysocetus healyorum* and the North African *Ancalecetus simonsi*. Another very weakly-supported (PP = 0.25) clade farther up the cetacean stem lineage includes *Zygorhiza kochii* and *Saghacetus osiris*, followed by a weakly-supported (PP = 0.18) clade composed of some of the late Eocene basilosaurids (*Pontogeneus*, *Dorudon*, and *Basilosaurus*) and all neocetes. Included is a moderately-supported (PP = 0.78) clade containing *Pontogeneus peruvianus*, *Dorudon atrox*, *Basilosaurus cetoides*, and *Basilosaurus isis*. *Pontogeneus* is the sister group of a moderately-supported (PP = 0.55) clade composed of *Dorudon* and *Basilosaurus*, with *Dorudon* being the sister taxon of the monophyletic, almost maximally supported (PP = 0.99), *Basilosaurus* clade (*Basilosaurus cetoides* + *Basilosaurus isis*). In this second tip-dating analysis, the clade that comprised *Pontogeneus peruvianus*, *Dorudon atrox*, *Basilosaurus cetoides*, and *Basilosaurus isis* is the sister taxon of the more derived neocetes and leads to a maximally supported (PP = 1.0) Neoceti clade.

Ancestral State Reconstructions (ASRs): Bayesian ancestral state reconstructions (ASRs) were calculated for all characters in MBASR[30] (Supplementary Data (3)). One reconstructed trait is the posterior margin of the nasal in relation to the posterior margin of the maxilla (PMM) (Character 6; Supplementary Figure 16 and Supplementary Table 9). According to the ASRs, the retention of the nasal extension posterior to the PMM, which is evident in most basilosaurids such as *Saghacetus*, *Pontogeneus*, *Dorudon*, and *Basilosaurus*, represents the ancestral state of probably all non-basilosaurid archaeocetes. As a result, possessing a posterior nasal edge at or anterior to the PMM in the basilosaurids *Ocucajea*, *Zygorhiza* and Neoceti is a derived state that probably evolved convergently in *Tutcetus*-clade and in Neoceti (Node #73).

Another reconstructed trait is the nasal process of the frontal (Character 16; Supplementary Figure 16 and Supplementary Table 10). According to ASRs, the absence of the nasal process of the frontal, which is evident in some basilosaurids such as *Saghacetus* and *Ocucajea*, represents a derived trait in some Neoceti (Node #79). ASRs for the accessory denticles on P_3 (Character 106; Supplementary Figure 19 and Supplementary Table 13) reveal that more denticles on the distal edge of the tooth is the ancestral trait of all basilosaurids and Neoceti and is probably retained by their common ancestor (Node #72). Therefore, the equal number of denticles on both edges of the tooth evolved by most basilosaurids such as *Zygorhiza*, *Saghacetus*, *Pontogeneus*, *Dorudon*, and *Basilosaurus* is a derived trait that arose again in the early Oligocene mysticete *Coronodon*. ASRs for the prominent denticulate cingula on $P^{2\text{-}4}$ (Character 119; Supplementary Figure 20 and Supplementary Table 16) reveals that the absence of the prominent denticulate cingula on $P^{2\text{-}4}$ is likely the ancestral state of all non-basilosaurid archaeocetes. Therefore, the evolution of this trait represents a derived state that arose in the common ancestor of the *Tutcetus*-clade (Node #86) and in the basilosaurids *Zygorhiza* and *Pachycetus paulsonii*. This trait arose again in the Oligocene odontocete *Mirocetus*.

Supplementary Figures

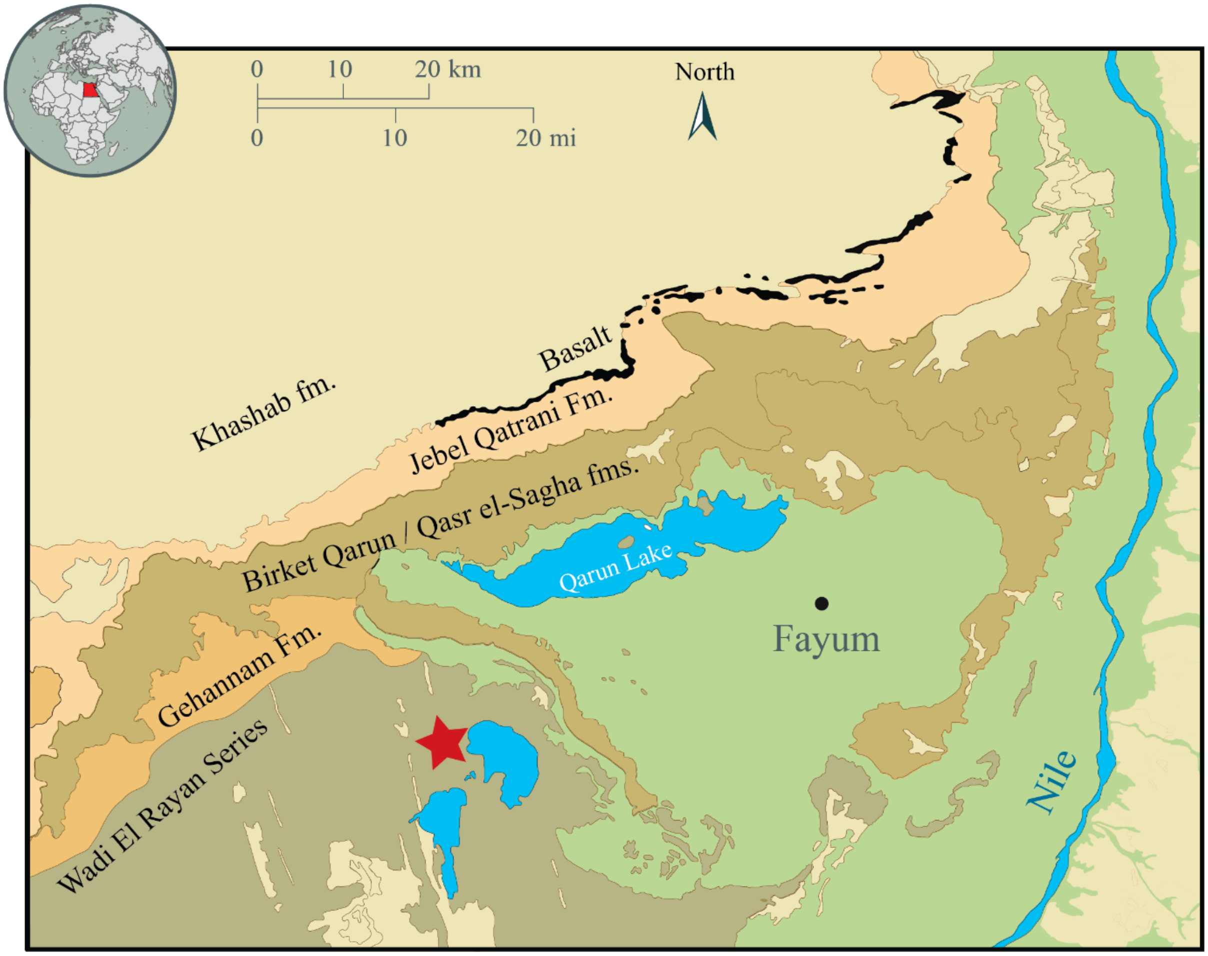

Supplementary Figure 1. The geologic map of the Fayum Depression shows the type locality of the holotype of *Tutcetus rayanensis*, n. gen. et sp. (red star).

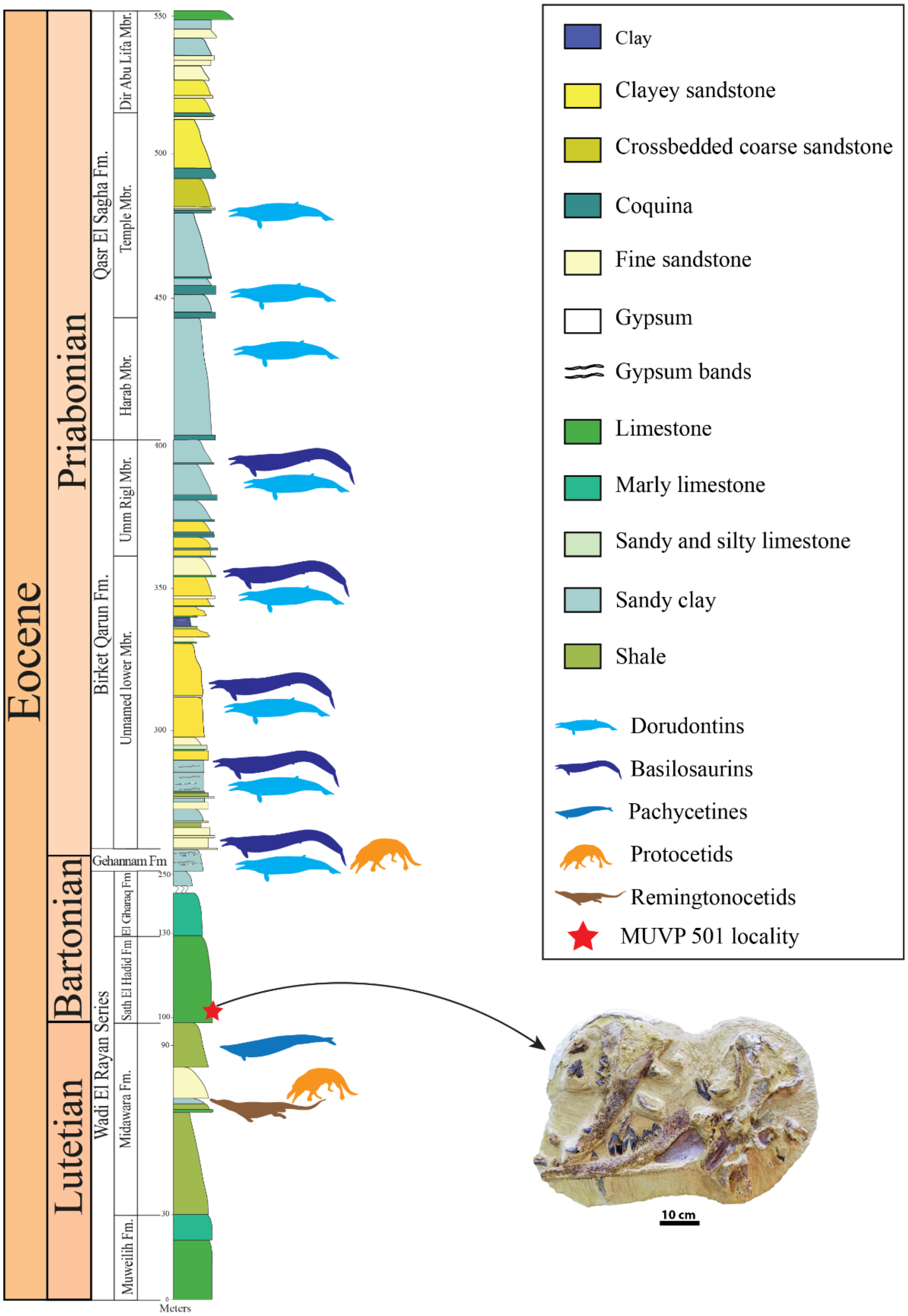

Supplementary Figure 2. The stratigraphic column of the Eocene geological formations of the Fayum Depression, modified after Zalmout and Gingerich[9], shows the cetacean records and indicates the position of *Tutcetus rayanensis*, n. gen. et sp. as one of the oldest definitive basilosaurids from the Fayum Depression.

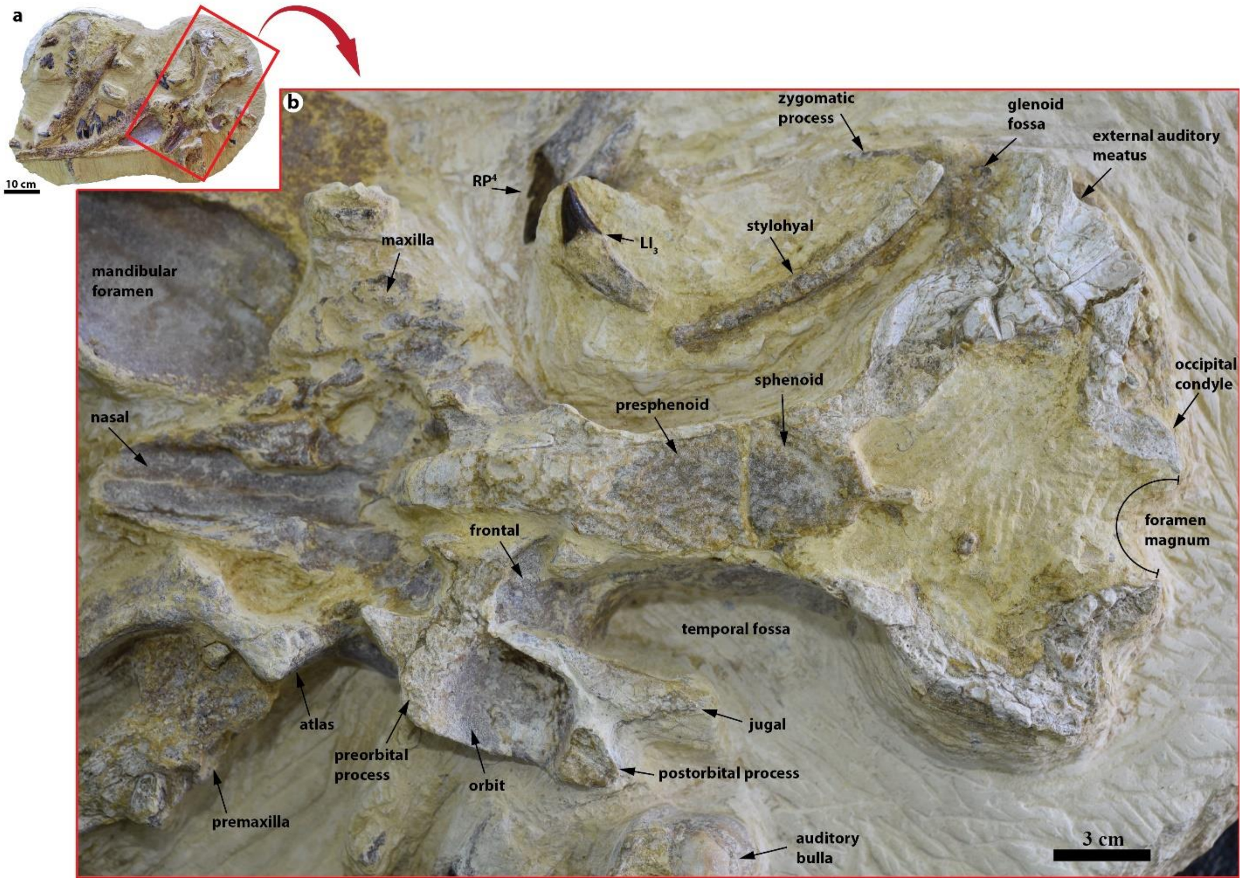

Supplementary Figure 3. a, Block containing the holotype specimen of *T. rayanensis* (MUVP 501); **b**, Close-up of the ventral view of the skull of *T. rayanensis*.

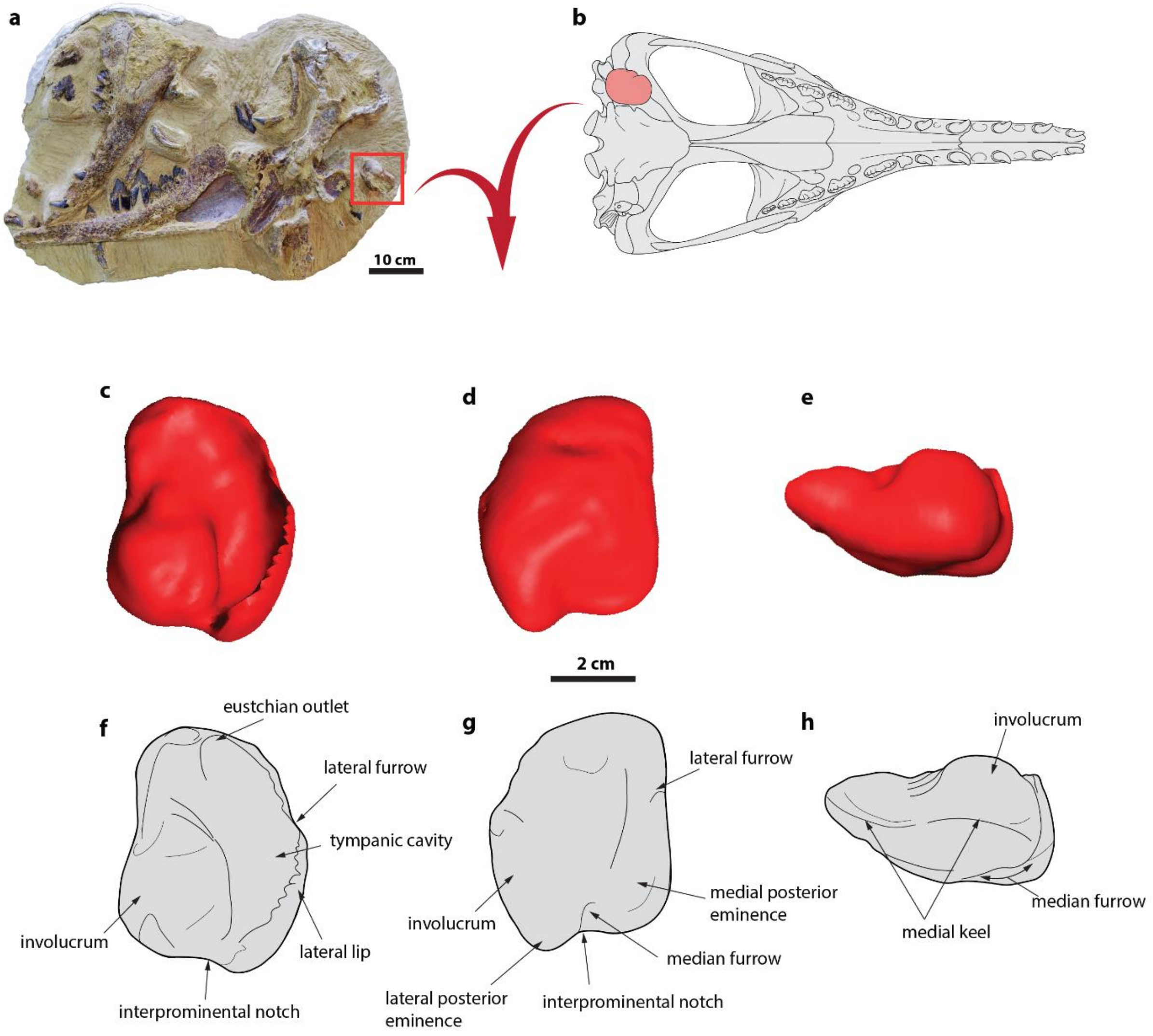

Supplementary Figure 4. a, Block containing the holotype specimen of *T. rayanensis* (MUVP 501) with the location of its right auditory bulla indicated by a red rectangle; **b**, Ventral view of a typical basilosaurid skull shows the location of the auditory bulla; digital model of the auditory bulla based on CT scans (**c-e**) and line drawing (**f-h**) of the right auditory bulla of *T. rayanensis* in dorsal (**c, f**), ventral (**d, g**) and posteromedial (**e, h**) views.

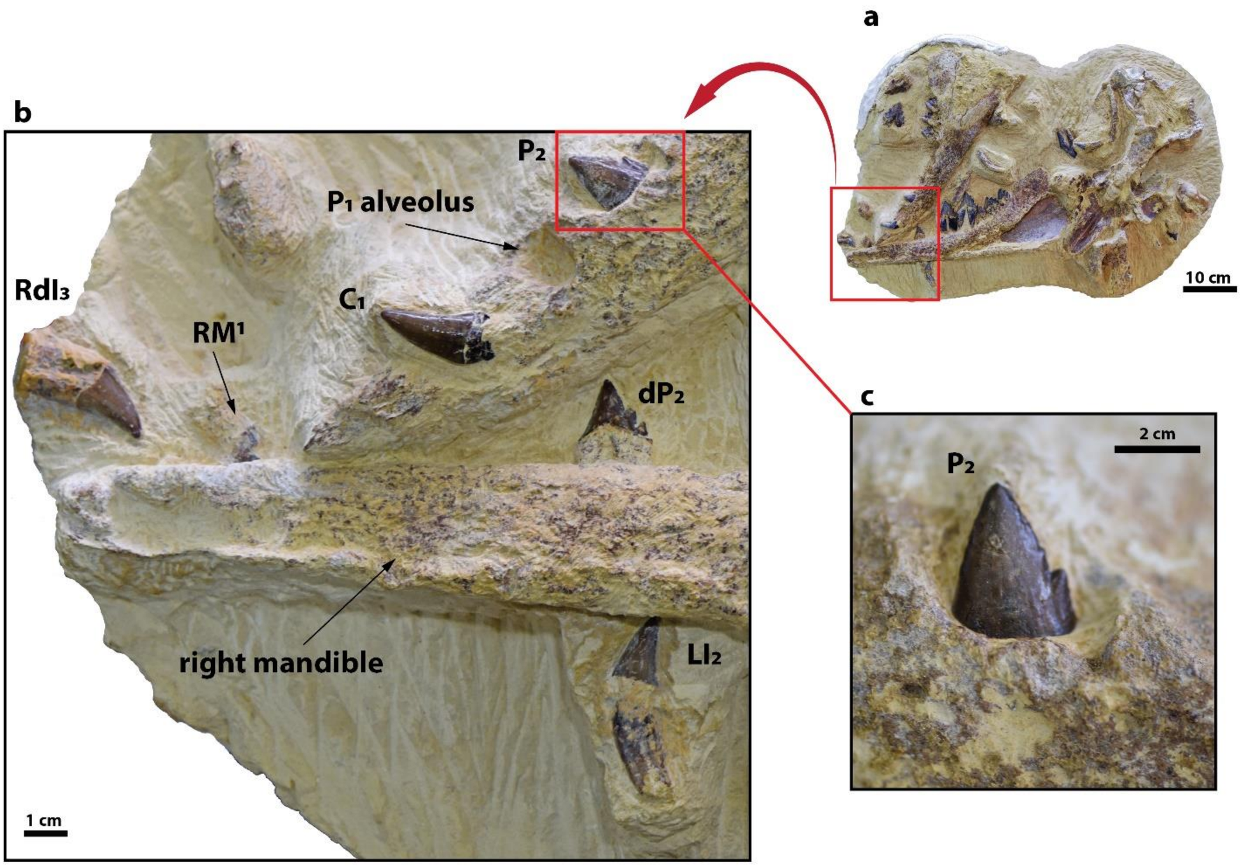

Supplementary Figure 5. a, Block containing the holotype specimen of *T. rayanensis* (MUVP 501); **b**, Close-up view of the anterior mandibular region; **c**, Close-up view of the left P_2 in labial view.

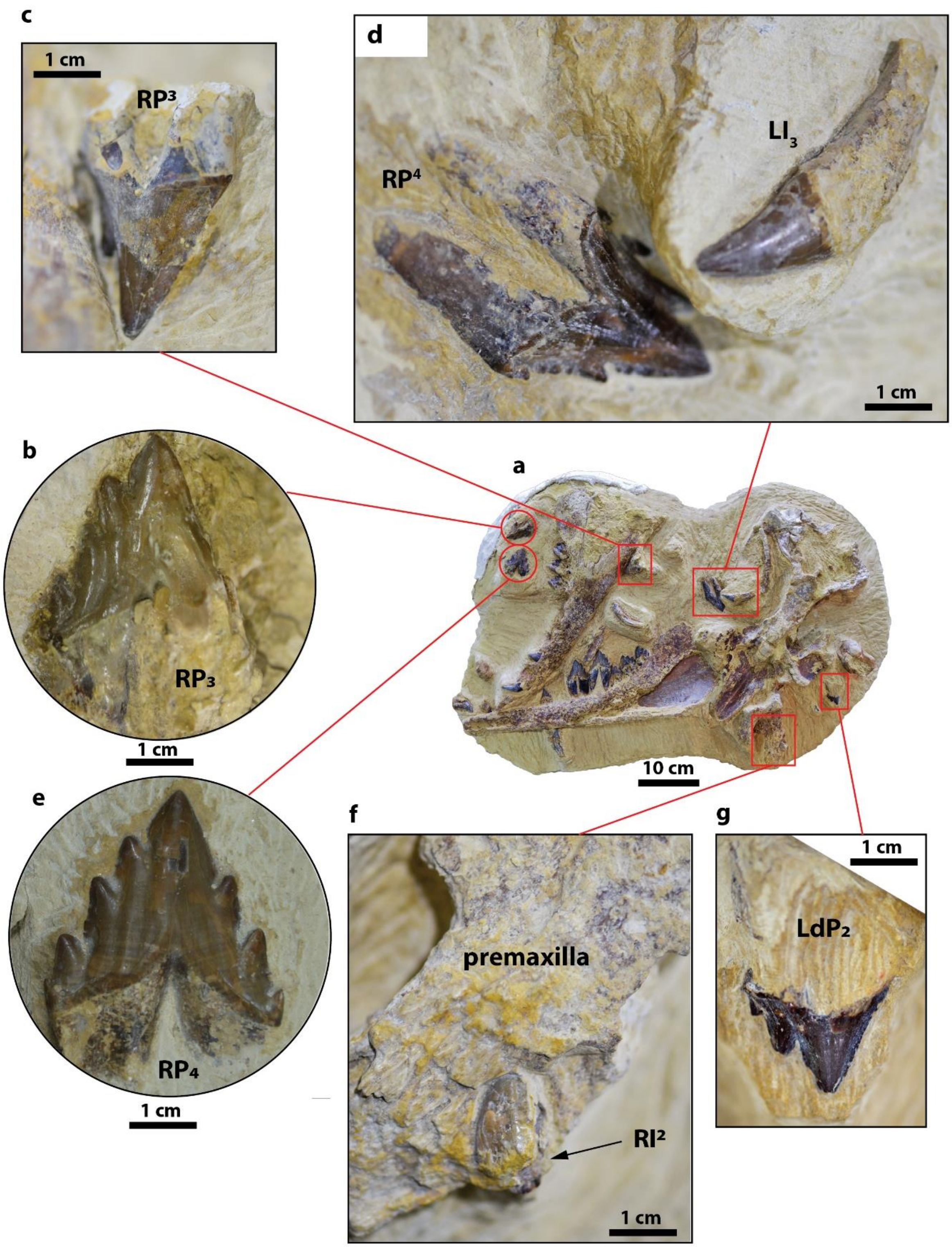

Supplementary Figure 6. a, Block containing the holotype specimen of *T. rayanensis* (MUVP 501); **b-g**, Close-up views of the scattered teeth associated with the holotype; **b**, Right P_3 in lingual view; **c**, Right P^3 in lingual view; **d**, Right P^4 and the left I_3; **e**, Right P_4 in labial view; **f**, Damaged right I^2; **g**, Left deciduous P_2.

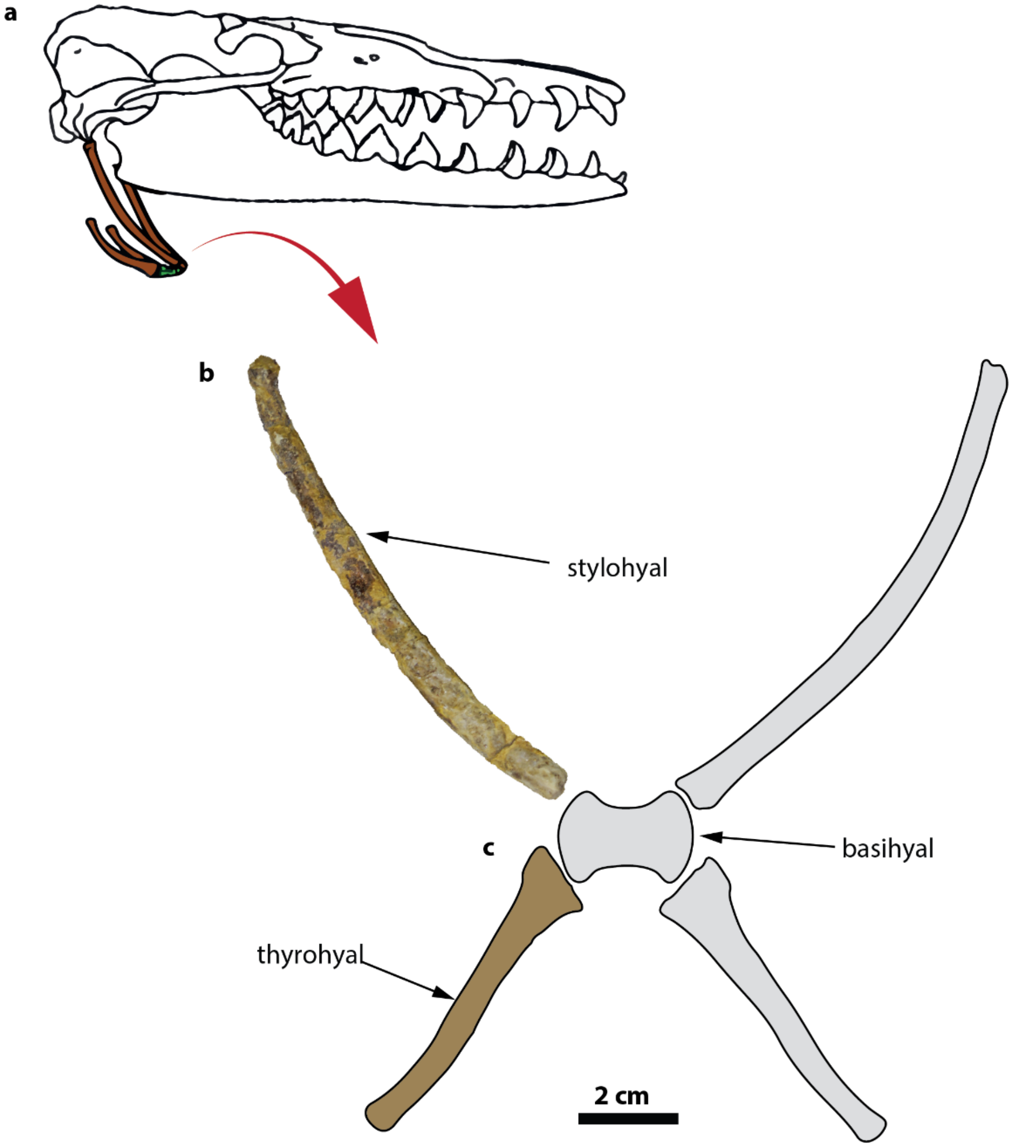

Supplementary Figure 7. a, Lateral view of a typical basilosaurid skull showing the location of the hyoid apparatus; **b-c**, hyoid apparatus of *Tutcetus rayanensis* (MUVP 501, holotype).

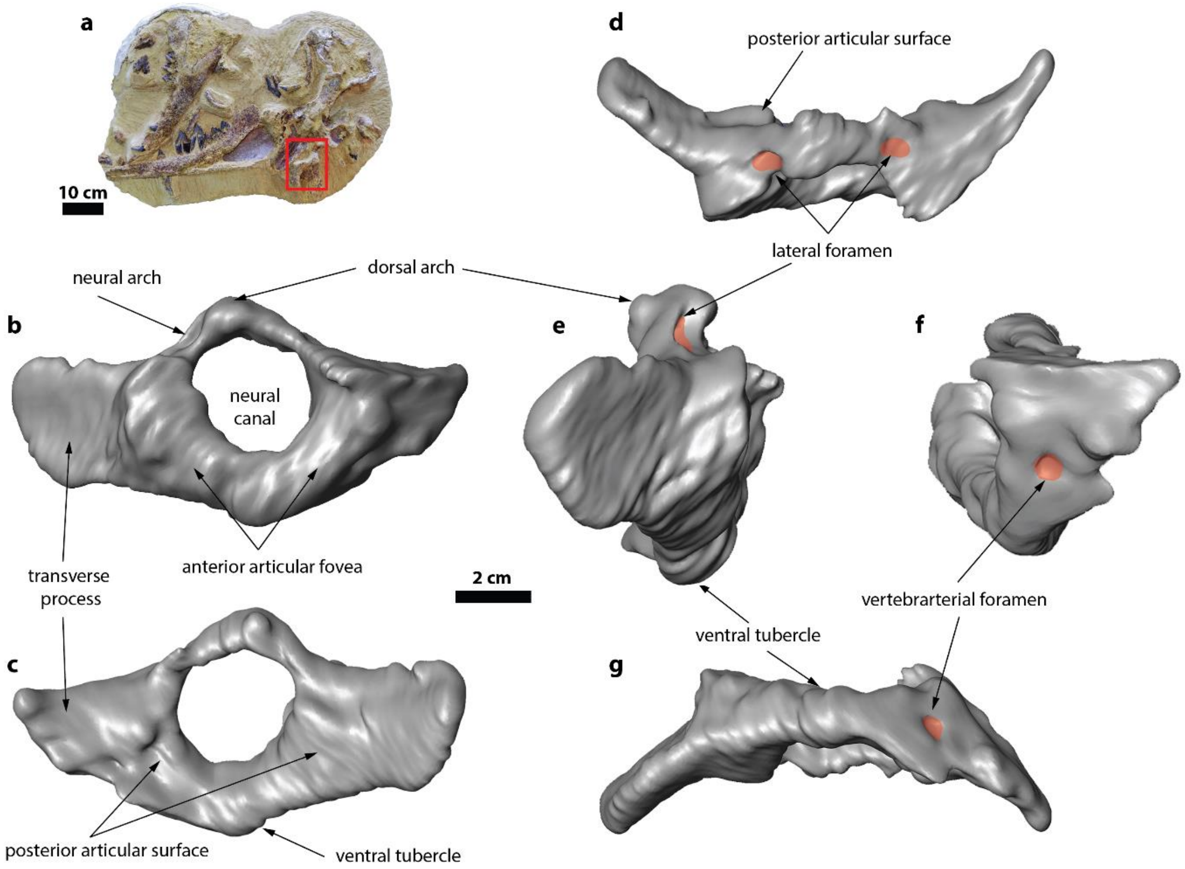

Supplementary Figure 8. a, Block containing the holotype specimen of *T. rayanensis* (MUVP 501) showing the position of the atlas (red rectangle); **b-g**, CT-based digital reconstruction of the atlas of *Tutcetus rayanensis*. Atlas in (**b**) anterior, (**c**) posterior, (**d**) dorsal (**e**) right lateral (**f**) left lateral, and (**g**) ventral views.

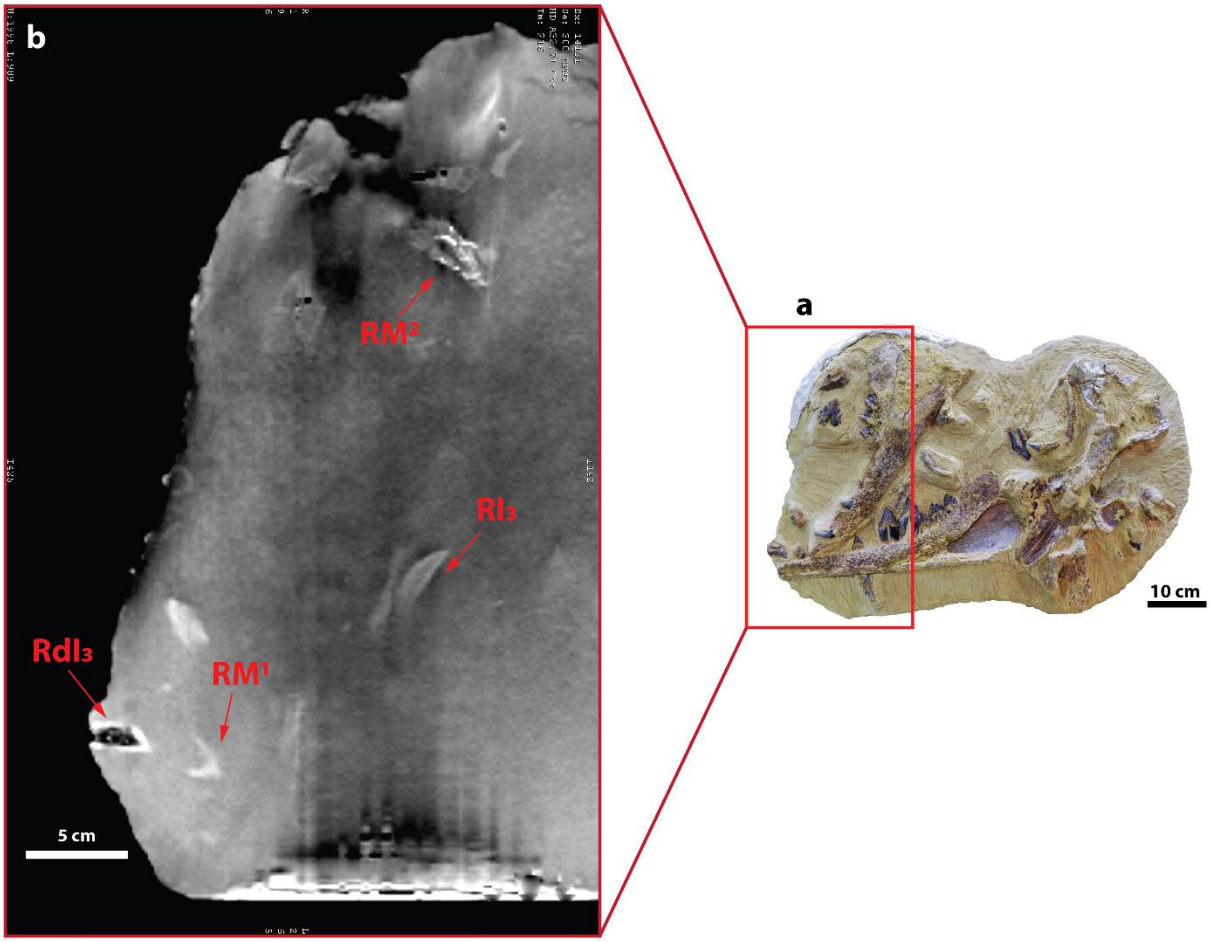

Supplementary Figure 9. a, Block containing the holotype specimen of *T. rayanensis* (MUVP 501); **b**, zoomed, leveled, and labeled portion of a CT slice from the CT scan of the block containing the holotype of *T. rayanensis* showing different isolated teeth.

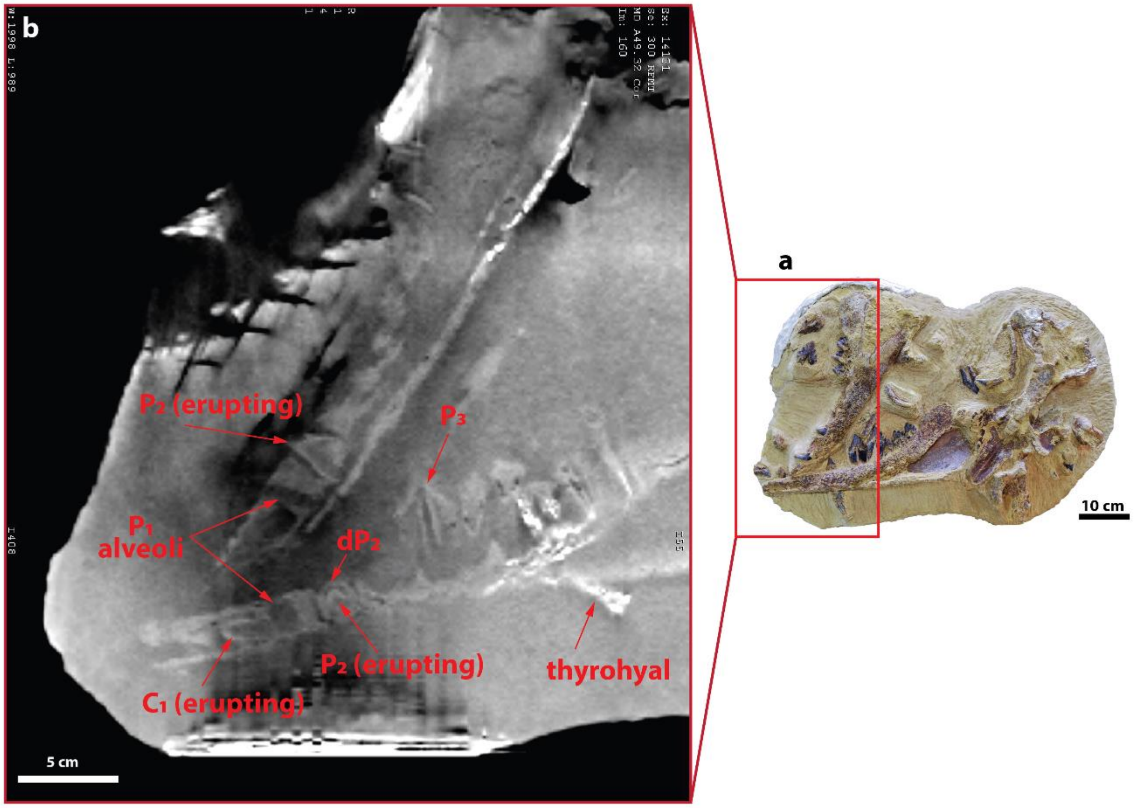

Supplementary Figure 10. a, Block containing the holotype specimen of *T. rayanensis* (MUVP 501); **b**, Zoomed, leveled, and labeled portion of a CT slice from the block containing the holotype of *T. rayanensis* showing empty P_1 alveoli, the eruption of P_2 and C_1, and the thyrohyal bone.

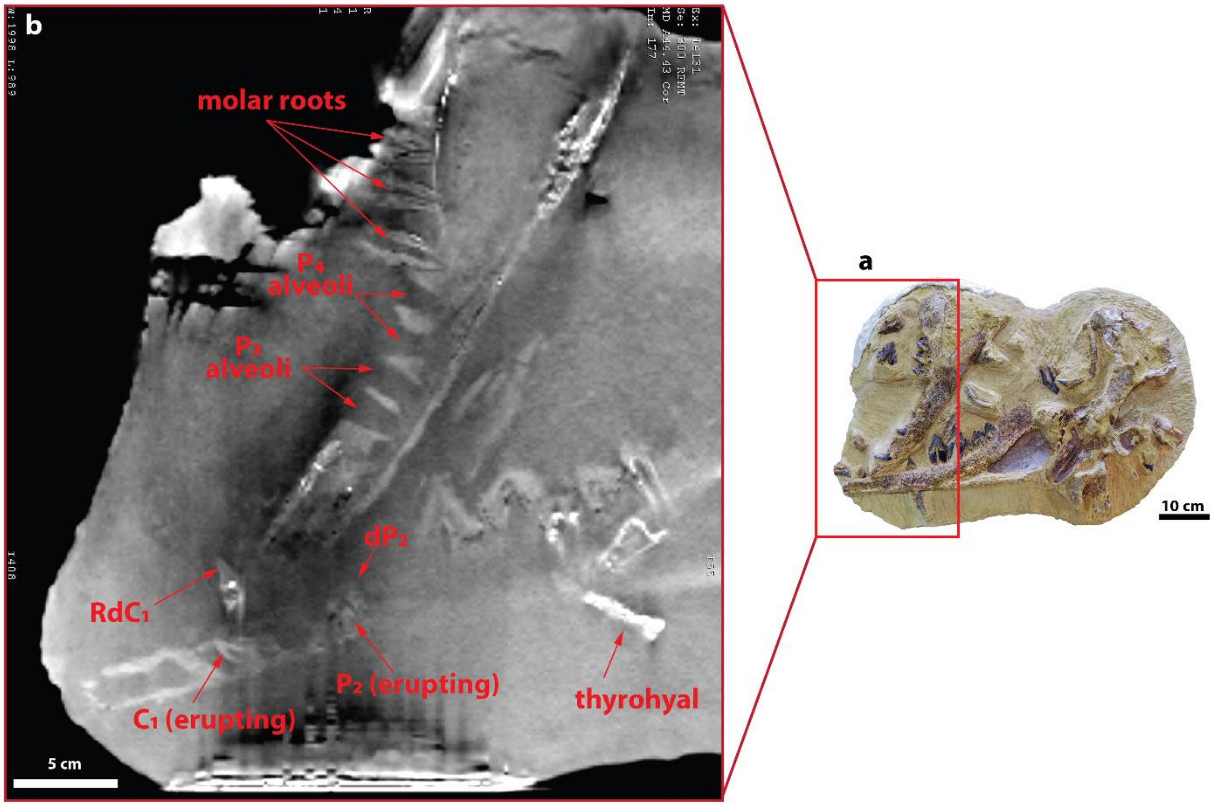

Supplementary Figure 11. a, Block containing the holotype specimen of *T. rayanensis* (MUVP 501); **b**, Zoomed, leveled, and labeled portion of a CT slice from the block containing the holotype of *T. rayanensis* showing the eruption of P_2 and C_1, lower long molar roots, deep alveoli of P_{3-4}, and the thyrohyal bone.

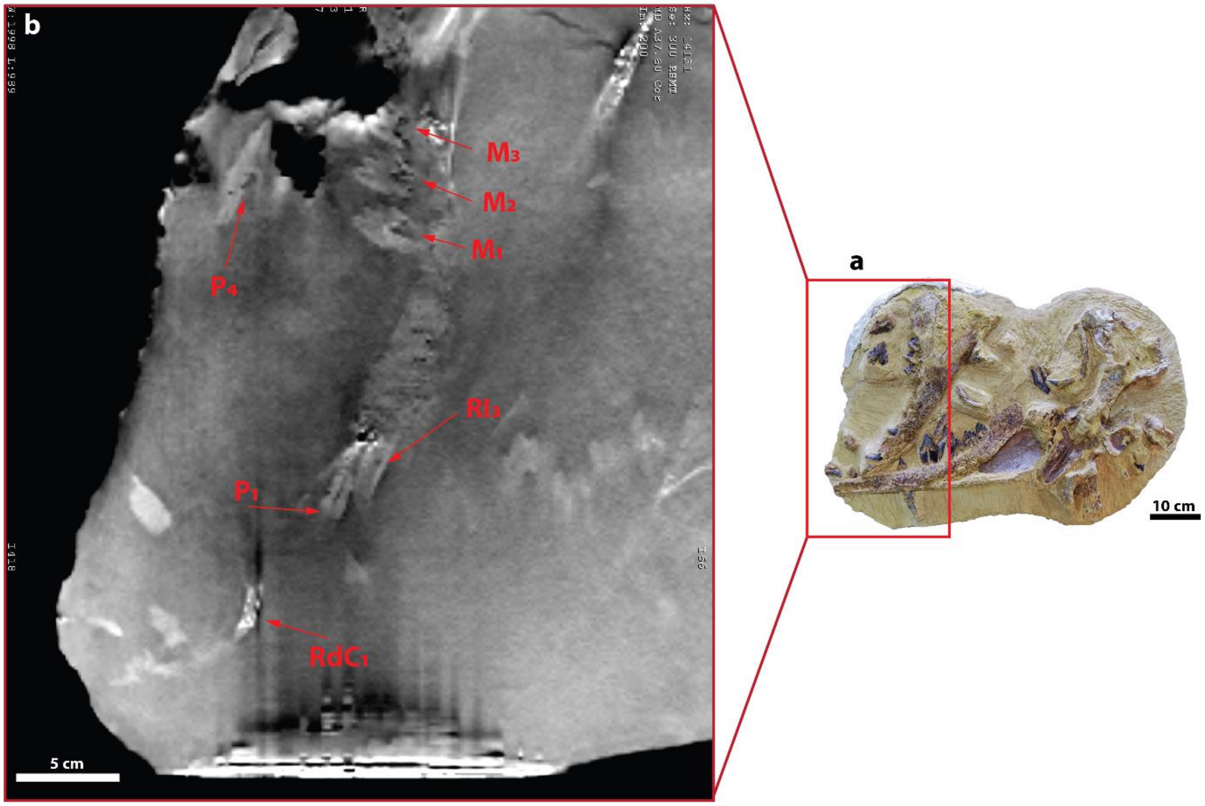

Supplementary Figure 12. a, Block containing the holotype specimen of *T. rayanensis* (MUVP 501); **b**, Zoomed, leveled, and labeled portion of a CT slice from the block containing the holotype of *T. rayanensis* showing lower molars, double-rooted P_1, and other isolated teeth.

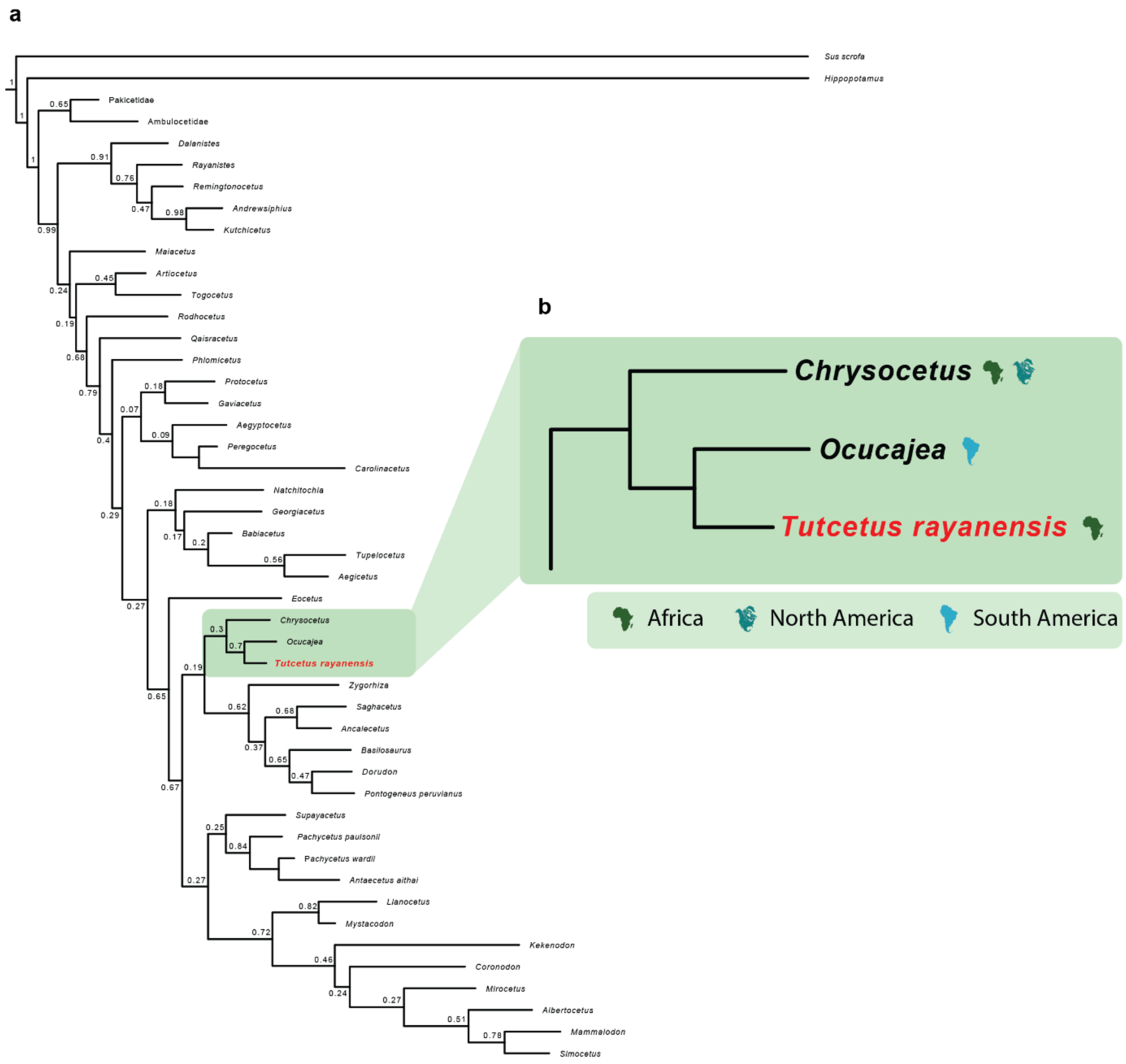

Supplementary Figure 13. **a**, Allcompat consensus tree from the Bayesian tip-dating analysis of the 195-character matrix in MrBayes 3.2.5 with the implementation of the FBD prior. Numerical values to the left of nodes represent posterior probabilities (PPs). Icons above taxon names reflect the geographic location of each fossil. **b**, An expanded subset (*Tutcetus*-clade) of the 'allcompat' consensus tree from the Bayesian tip-dating analysis shows the phylogenetic relationships in the *Tutcetus*-clade.

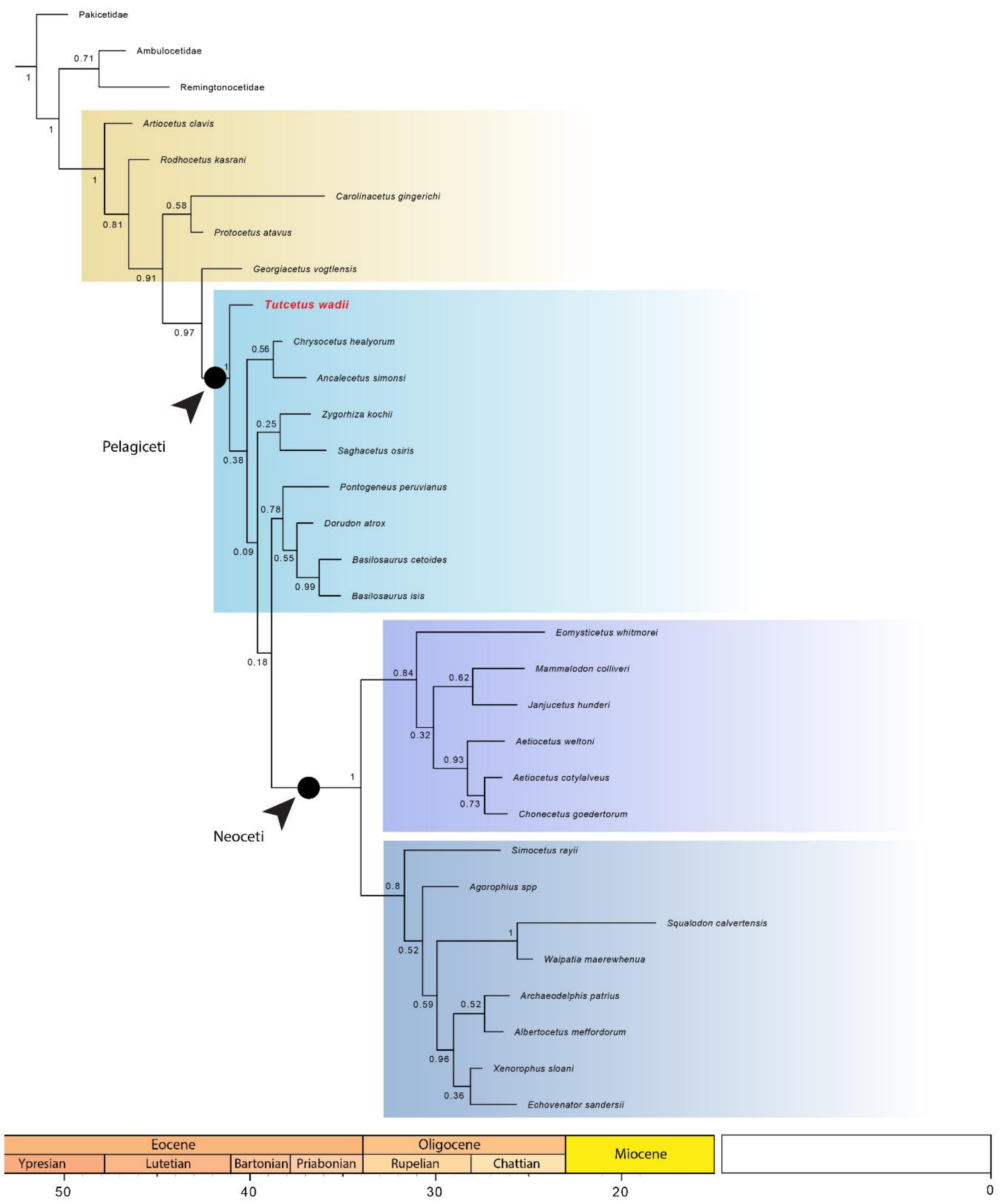

Supplementary Figure 14. Phylogenetic relationships and biogeography of *Tutcetus rayanensis* (MUVP 501) and other extinct cetaceans. 'Allcompat' consensus (majority-rule plus compatible groups) tree from the Bayesian tip-dating analysis of the 101-character matrix in MrBayes 3.2.5. Numerical values to the left of nodes represent posterior probabilities (PPs).

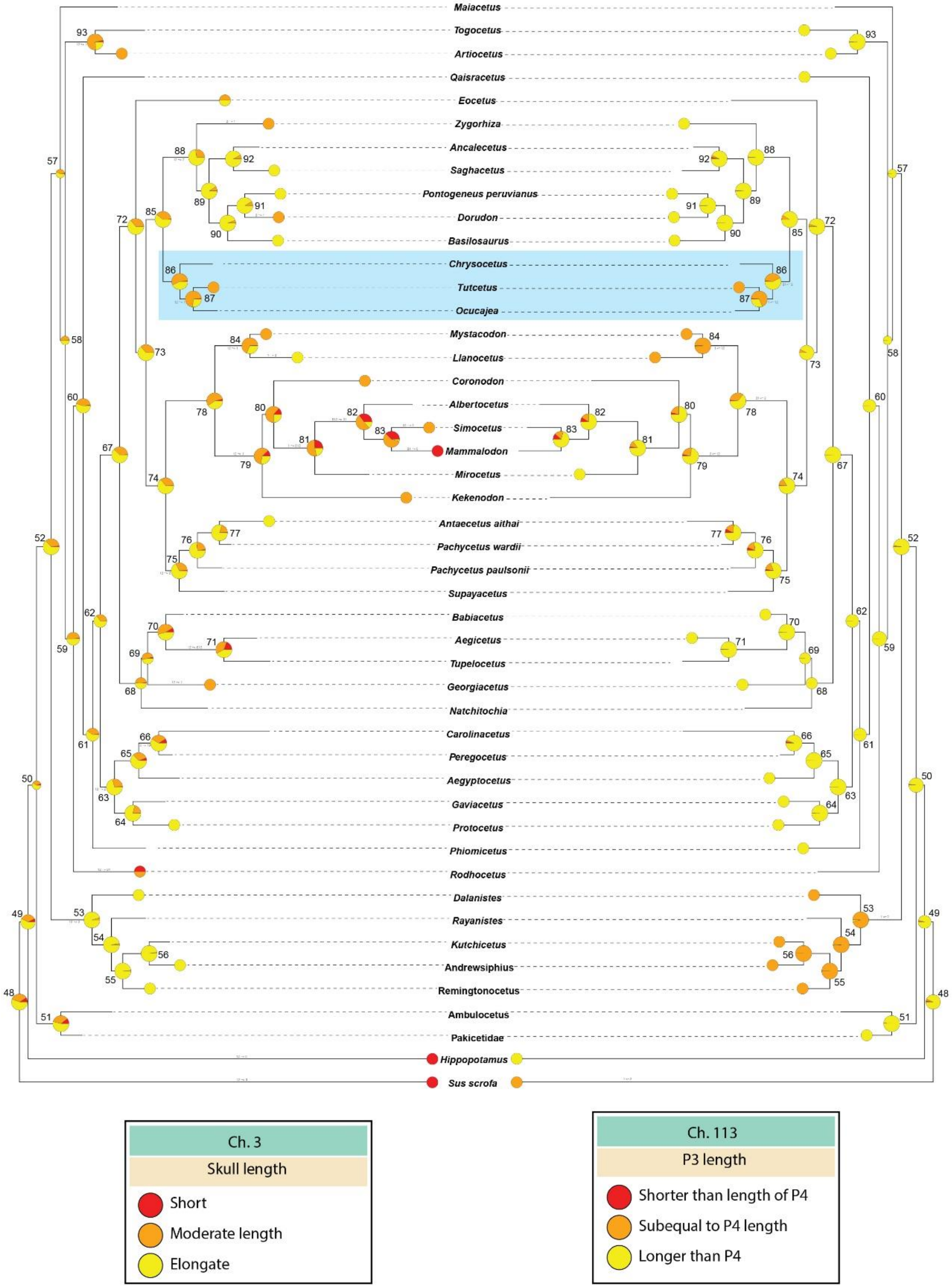

Supplementary Figure 15. Bayesian ancestral state reconstruction of skull length (Ch. 3; left) and P3 length (Ch. 113; right) showing the posterior probability of the states for the internal nodes resulting from analysis in MBASR using the 'allcompat' consensus tree from the Bayesian tip-dating analysis.

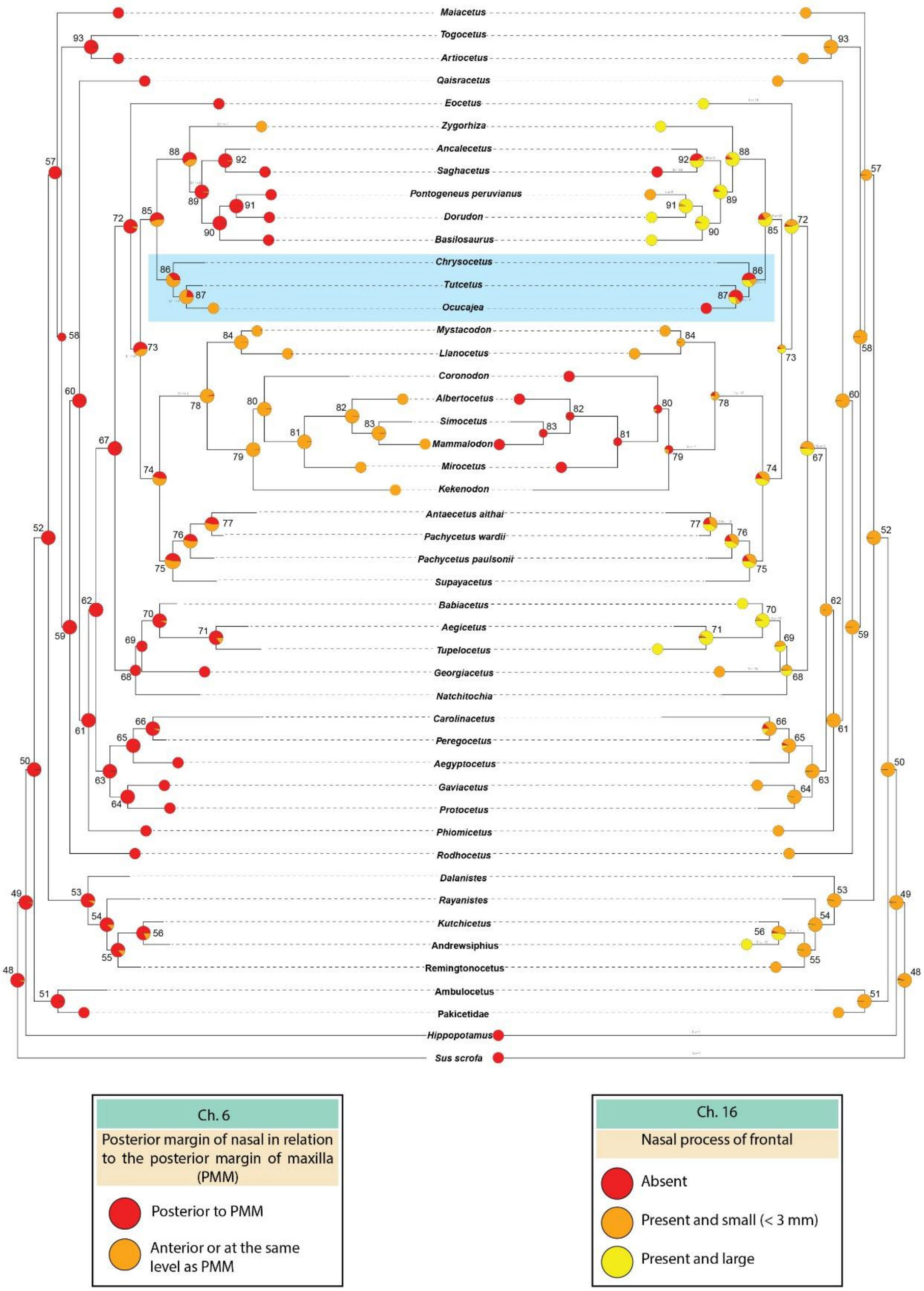

Supplementary Figure 16. Bayesian ancestral state reconstruction of the posterior margin of the nasal in relation to the posterior margin of maxilla (Ch. 6; left) and nasal process of frontal (Ch. 16; right) showing the posterior probability of the states for the internal nodes resulting from analysis in MBASR using the 'allcompat' consensus tree from the Bayesian tip-dating analysis.

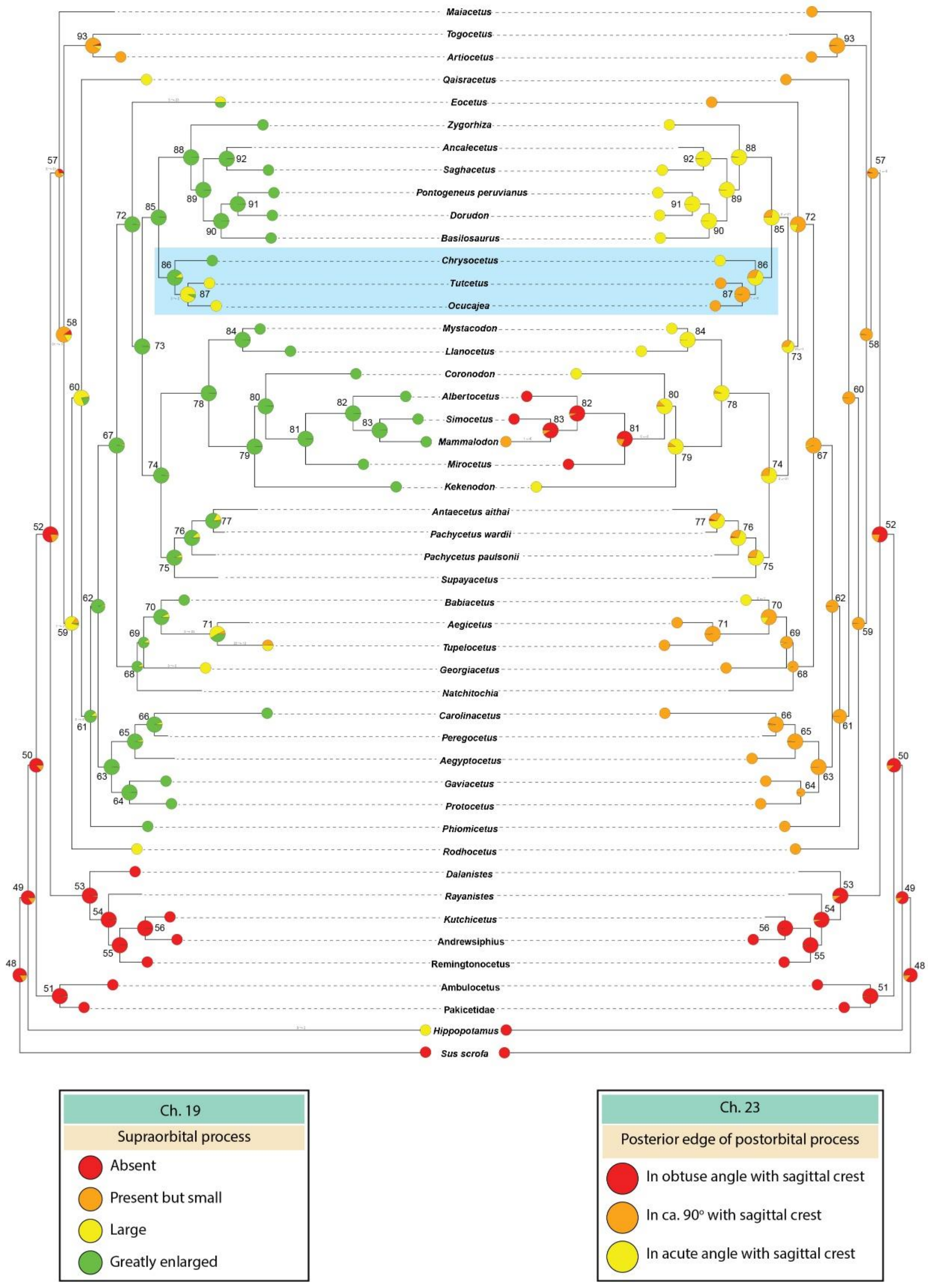

Supplementary Figure 17. Bayesian ancestral state reconstruction of the supraorbital process (Ch. 19; left) and the posterior edge of the postorbital process (Ch. 23; right) show the posterior probability of the states for the internal nodes resulting from analysis in MBASR using the 'allcompat' consensus tree from the Bayesian tip-dating analysis.

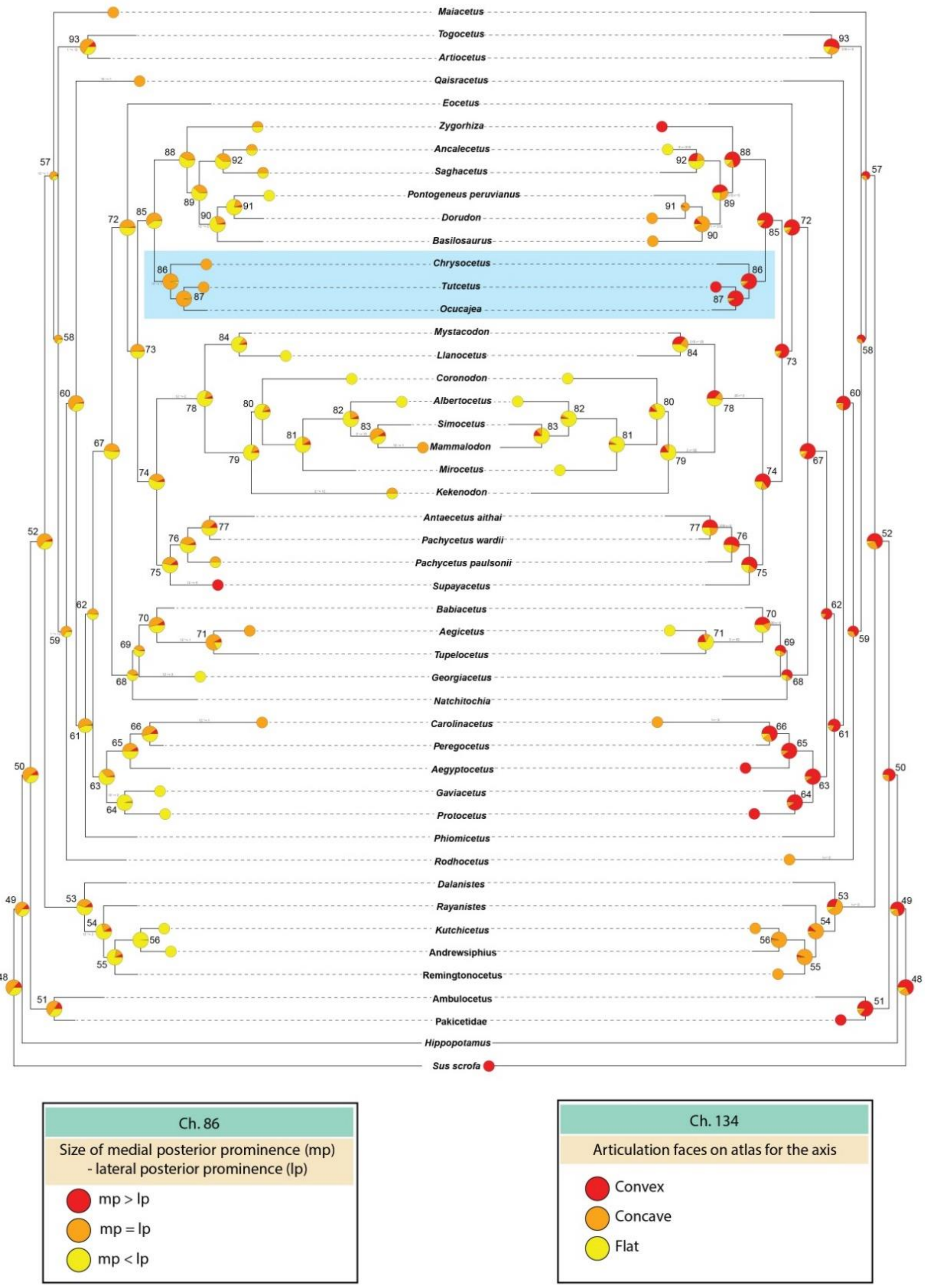

Supplementary Figure 18. Bayesian ancestral state reconstruction of the size of the medial posterior prominence – lateral posterior prominence (Ch. 86; left) and the articulation facets on the atlas for the axis (Ch. 134; right) show the posterior probability of the states for the internal nodes resulting from analysis in MBASR using the 'allcompat' consensus tree from the Bayesian tip-dating analysis.

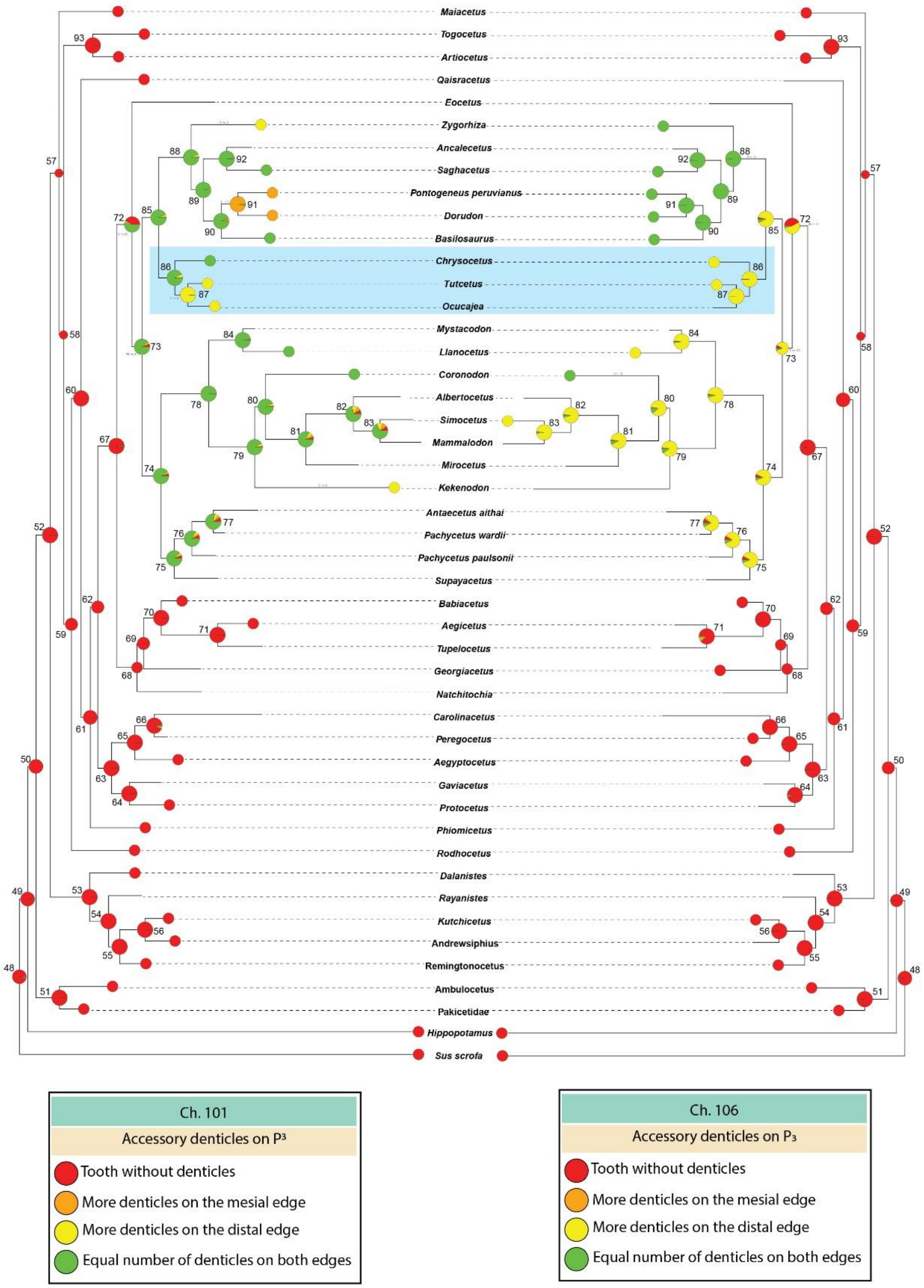

Supplementary Figure 19. Bayesian ancestral state reconstruction of the accessory denticles on P^3 (Ch. 101; left) and P_3 (Ch. 106; right) shows the posterior probability of the states for the internal nodes resulting from analysis in MBASR using the 'allcompat' consensus tree from the Bayesian tip-dating analysis.

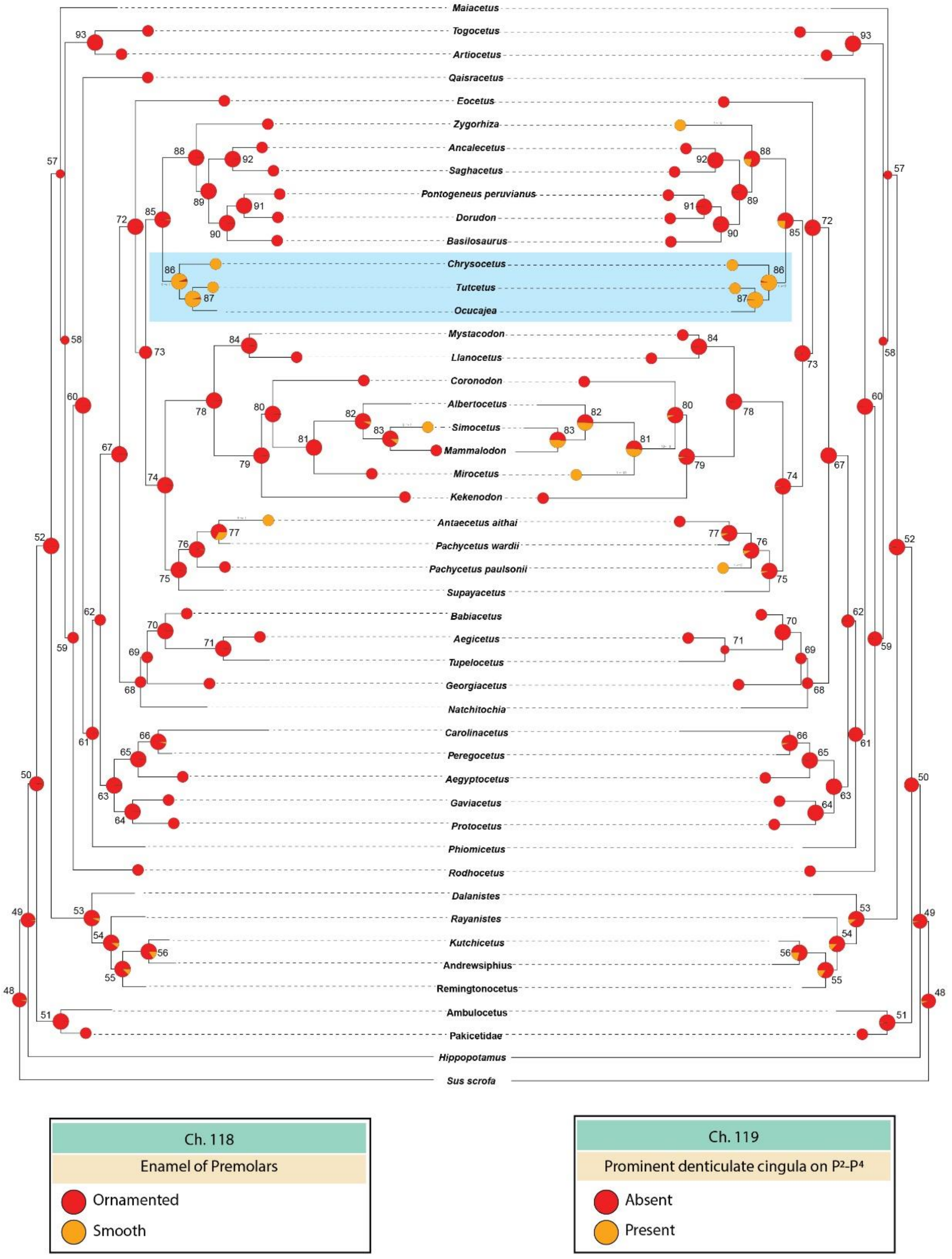

Supplementary Figure 20. Bayesian ancestral state reconstruction of the enamel of the premolars (Ch. 118; left) and the prominent denticulate cingula on P^{2-4} (Ch. 119; right) shows the posterior probability of the states for the internal nodes resulting from analysis in MBASR using the 'allcompat' consensus tree from the Bayesian tip-dating analysis.

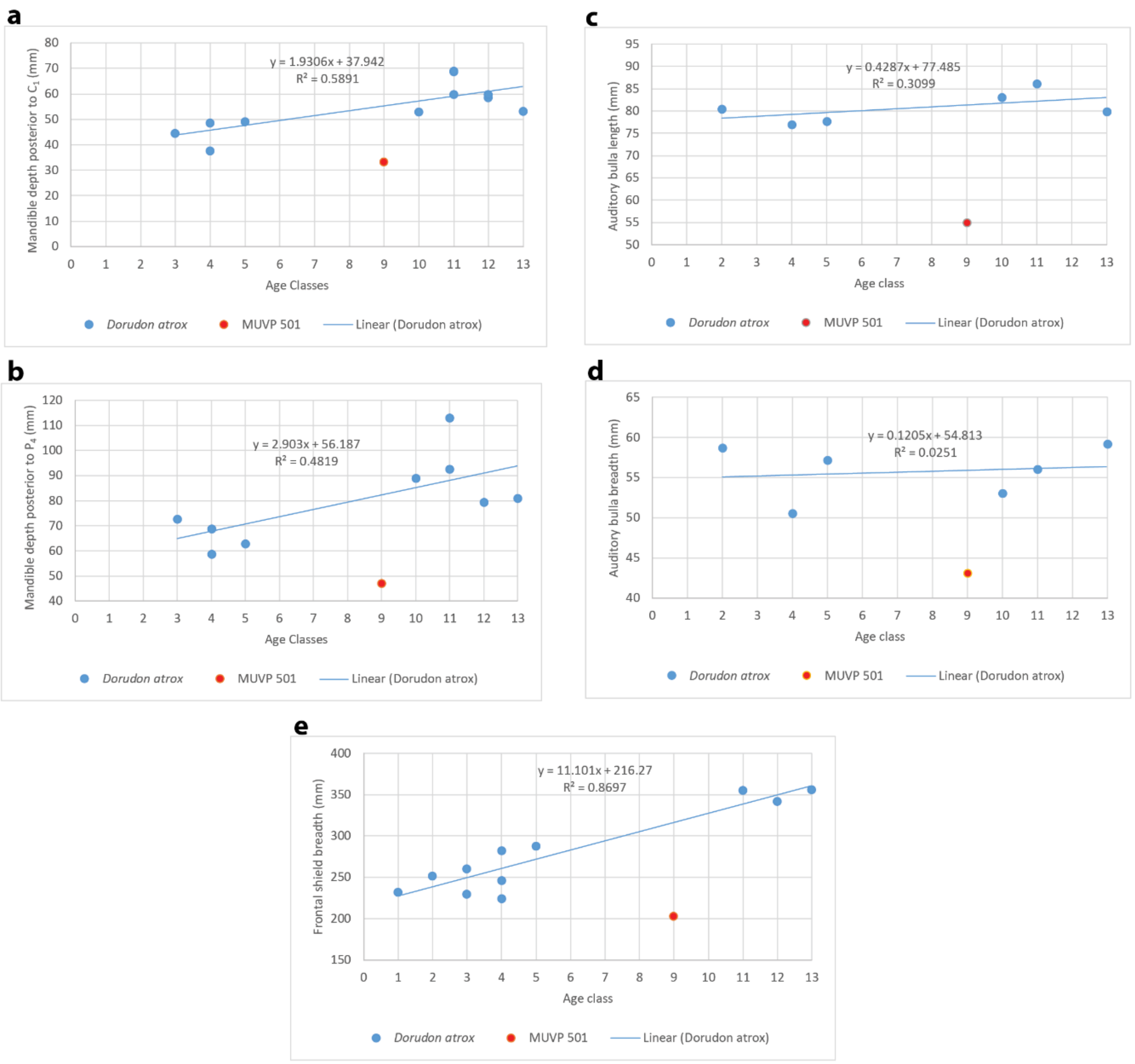

Supplementary Figure 21. Anatomical characteristics of *Tutcetus rayanensis* (MUVP 501) compared to the ontogenetic growth curves for *Dorudon atrox* (Uhen, 2004). MUVP 501 corresponds to ontogenetic stage 9 of Uhen's[1] 13 tooth eruption and dental wear stages. Growth in mandibular depth at C_1 (**a**) and P_4 (**b**). Change in the length (**c**) and breadth (**d**) of the auditory bulla; **e**, Changes in the breadth of the frontal shield.

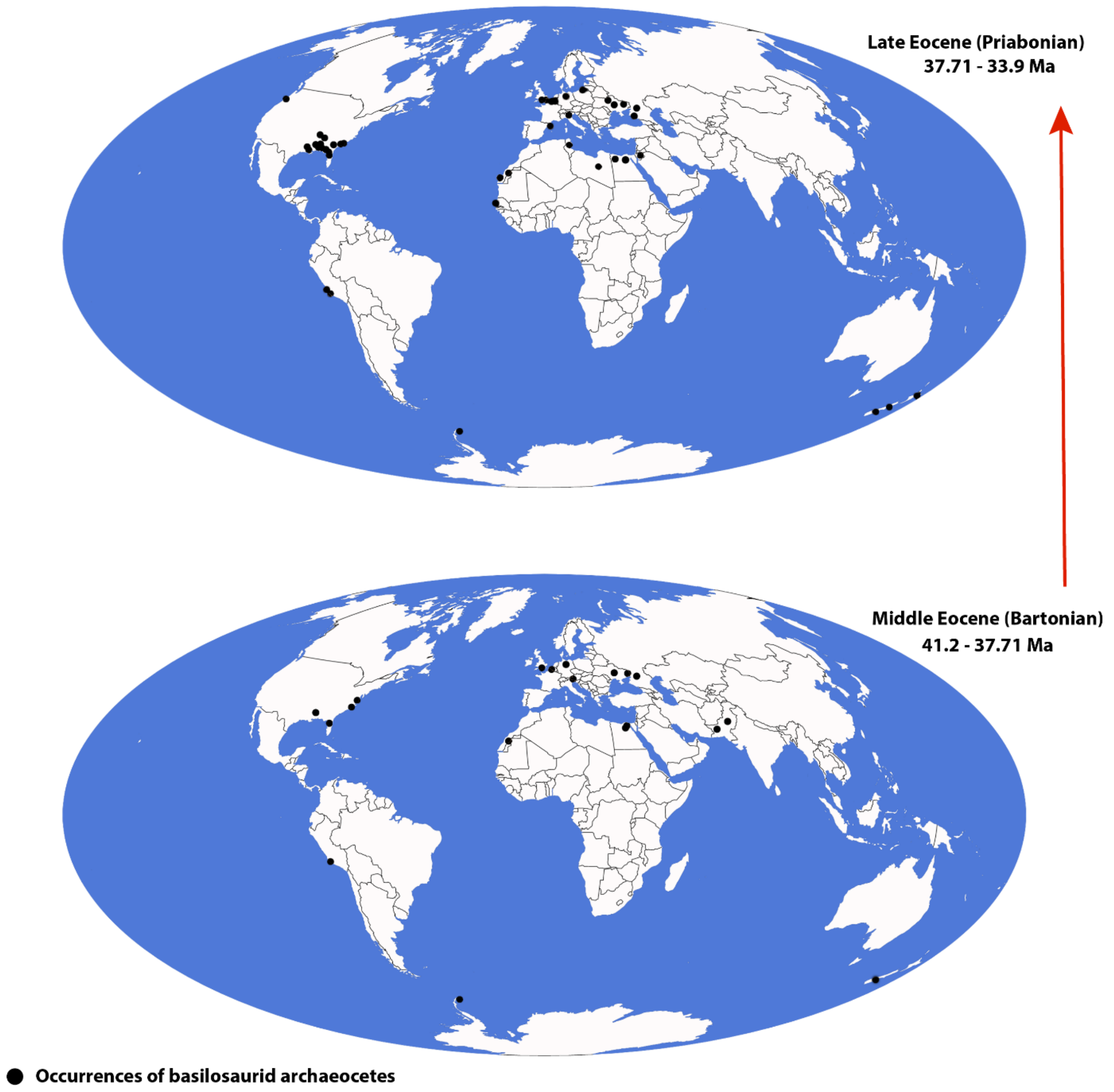

Supplementary Figure 22. Distribution of cetacean fossils from the middle Eocene into the late Eocene**.**

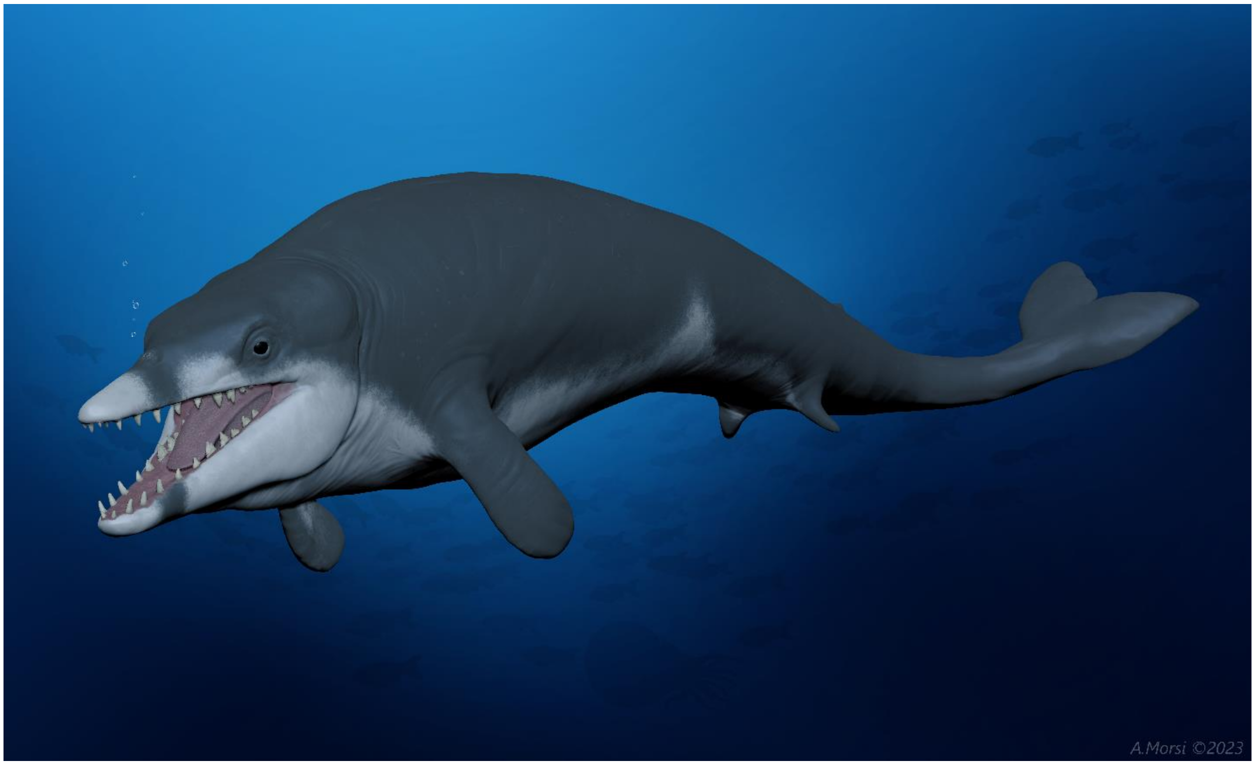

Supplementary Figure 23. Life Reconstruction of *Tutcetus rayanensis*, n. gen. et sp.: Depicting the extinct basilosaurid whale swimming in the Tethys Ocean of present-day Egypt, 41 million years ago. Illustration by Ahmed Morsi.

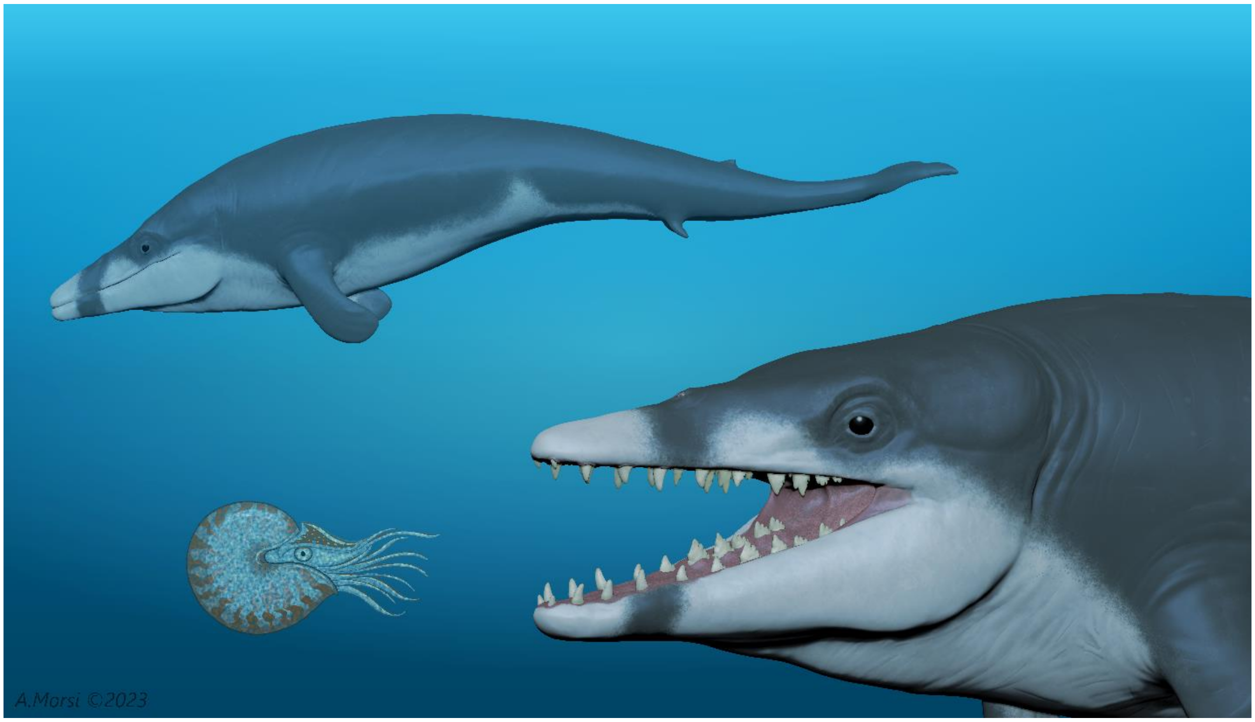

Supplementary Figure 24. Life reconstruction of *Tutcetus rayanensis*: A scene depicting two extinct basilosaurid whales, with the foreground individual preying on a nautilid cephalopod and another swimming in the background. Illustration by Ahmed Morsi.

Supplementary Tables

Supplementary Table 1. Institutional abbreviations

Abbreviation	Institution	Home country
**CGM**	Cairo Geological Museum	Egypt
**GMTSNUK**	Geological Museum of Taras Shevchenko National University of Kyiv	Ukraine
**GSM**	Georgia Southern Museum, Statesboro	USA
**GSP-UM**	Geological Survey of Pakistan-University of Michigan collection, Quetta, Balochistan	Pakistan
**KPG-M**	Université de Montpellier, Institut des Sciences de l’Evolution paleontology collection	France
**LUVP**	Lucknow University, Vertebrate Paleontology	India
**MNHN**	Muséum national d’Histoire naturelle, Paris	France
**MSNTUP**	Museo di Storia Naturale e del Territorio, Università di Pisa	Italy
**MUSM**	Departamento de Paleontologı´a de Vertebrados, Museo de Historia Natural de San Marcos collections, Lima	Peru
**MUVP**	Mansoura University Vertebrate Paleontology Center	Egypt
**NHML**	Natural History Museum, London	UK
**SCSM**	South Carolina State Museum, South Carolina	USA
**SMNS**	Staatliches Museum für Naturkunde, Stuttgart	Germany
**UM**	University of Michigan Museum of Paleontology	USA

Supplementary Table 2. Measurements of the holotype of *Tutcetus rayanensis*, n. gen. et sp., MUVP 501. All measurements are in millimeters. The asterisk (*) indicates an estimated measurement.

Dimension	Measurement
**Skull**	
Condylobasal skull length (CBL)	580*
From the occipital to the tip of the nasal	369.6
Occipital condyle width (OCW)	73.3
Bizygomatic width (BZW)	261.3*
Transverse diameter of supraorbital processes at posterior processes	203
Greatest outside dimensions across the nuchal crest	78.3
Greatest inside dimensions across the nuchal crest	45.6
Greatest diameter at outside margins of exoccipitals	253.4
Foramen magnum width	31.5
**Mandible**	
Dentary length	441
Depth at the coronoid process	122.8
Mandibular foramen length	138.8
Mandibular foramen height	73.4
Depth of dentary below canine	33.3
Diastema between I_2-I_3	14
Diastema between I_3-C_1	14.1
Diastema between C_1-P_l	18.3
Diastema between P_1-P_2	12.2
Diastema between P_2-P_3	12.1

Supplementary Table 3. Measurements (mm) of the deciduous and permanent teeth and their accessory cusps in *Tutcetus rayanensis*, n. gen. et sp., MUVP 501. Missing teeth or measurements that cannot be taken are indicated by blank cells.

Tooth		Length		Width		Height		Mesial denticles		Distal denticles	
		Left	Right	Left	Right	Left	Right	Left	Right	Left	Right
Deciduous lower teeth	dI_3		13.4		8.7		19.5		0		0
	dC_1	9.4	9.2	6.8	6.3	9.6	9.8	0	0	0	0
	dP_2	21.7	19.3	7.6	7.2	14.3	17.2	0	0	2	2
Upper permanent teeth	I^2		11.2		5.6				0		0
	P^3		28.3		7.5		21.2		0		2
	P^4		29.6		6.8		22.7		2		2
	M^1		22.2		6.7		18.1		2		3
	M^2		20.1		6.1		14.6		1		2
Lower permanent teeth	I_2	12.3		7.4		18.2		0		0	
	I_3	14.2	13.9	8.3	8	19.1	18.8	0	0	0	0
	C_1	13.7	11.7	7.3	7.1	21.6	20.6	0	0	0	0
	P_1	13.3		7.6				0		0	
	P_2	19.4		8.2		22.3		0		1	
	P_3		35.6	9.3	8.9	25.1	24.4		2	3	3
	P_4	37.3	36.2	8.3	10.5	24.8	24.9	2	2	3	3
	M_1	23.9	24.2	6.8	6.5	17.3	17.6	0	0	3	3
	M_2	25.1	25.3	7.7	7.1	18.2	18.3	0	0	3	3
	M_3	24.3	24.1	7.8	7.0	18.4	18.3	0	0	3	3

Supplementary Table 4. *Tutcetus rayanensis* dental eruption and wear status compared to dorudontine whales. Specimens are listed in the first column in order of age at death from youngest to oldest as determined by their dental eruption sequence. Teeth are listed across the first row in order that they erupt as determined by many dorudontine individuals. Each cell indicates the status of each tooth in each individual using the following code: 0, not present; 1, forming in the crypt; 2, less than half of the crown erupted; 3, greater than half the crown erupted; 4, erupted with no wear; 5, erupted with wear that has not broken the enamel surface; 6, moderate wear with patches of dentine exposed; 7, heavy wear with dentine swaths exposed; 8, very heavy wear with greater than fifty percent of the enamel worn away. A dash indicates that the tooth is not preserved in the specimen. A blank cell indicates that no replacement occurs. The form of the table is after Uhen[1].

Species	Specimen	M_1	M^1	dP_1	dP^1	M_2	M^2	M_3	P^4	P_4	P^3	I^1	P_3	I_1	P^2	I^2	P_2	I_2	P^1	I^3	P_1	I_3	C^1	C_1
Dorudon atrox	UM 100139	-	1	-	2	-	0	-	0	-	0	0	-	-	0	0	-	-	0	0	-	-	0	-
Dorudon atrox	UM 94811	-	4	-	4	-	0	-	0	-	0	0	-	-	0	0	-	-	0	0	-	-	0	-
Dorudon atrox	UM 83902	4	-	4	-	2	-	0	-	0	-	-	0	-	-	-	0	-	-	-	0	-	-	0
Dorudon atrox	UM 94814	4	4	4	4	3	0	0	0	0	0	0	0	0	0	0	0	0	0	0	0	0	0	0
Dorudon atrox	UM 93220	4	4	4	4	4	4	2	0	1	0	1	0	-	0	0	0	0	0	0	0	0	0	0
Dorudon atrox	UM 94796	4	-	-	-	4	-	4	-	3	-	-	0	-	-	-	-	-	-	-	-	-	-	-
Zygorhiza kochii	USNM 4748	-	-	-	-	-	-	-	-	-	-	-	2	-	-	-	2	-	-	-	-	1	-	1
Zygorhiza kochii	USNM 16639	4	4	4	4	4	4	4	4	4	3	-	3	-	2	-	2	-	-	-	1	-	1	-
Tutcetus rayanensis	**MUVP 501**	**4**	**4**			**4**	**4**	**4**	**4**	**4**	**4**	**-**	**4**	**-**	**-**	**4**	**3**	**-**	**-**	**-**	**4**	**-**	**-**	**3**
Dorudon atrox	UM 100146	6	-	-	-	6	-	5	-	6	-	-	-	-	-	-	-	-	-	-	5	-	-	-
Dorudon atrox	UM 101222	7	7	-	-	7	7	5	6	7	6	-	6	-	6	-	7	-	-	-	-	-	-	-
Dorudon atrox	UM 97506	7	-	-	-	7	-	7	-	5	-	-	-	-	-	-	-	-	-	-	-	-	-	-
Dorudon atrox	UM 101215	7	6	-	-	6	8	5	6	6	6	8	6	-	-	8	6	-	-	7	6	-	-	7

Supplementary Table 5. *Tutcetus rayanensis* fusion status compared to that of *Dorudon atrox* individuals. Specimens are listed in the first column in order of age at death from youngest to oldest as determined by fusion status. Sutures are listed across the first row in order that they fuse as determined by these individuals. Each cell indicates the status of each suture in each individual using the following code: 0, unfused; 1 partly fused; 2, fused. A dash indicates that the element is not preserved in the specimen. The form of the table is after Uhen[1]. The table presents abbreviations for various anatomical landmarks, including the atlas dorsal arch (dors. C1), the basioccipital/basisphenoid suture (BO/BS), the cervical (cerv. ep.), caudal (caud. ep.), thoracic (thor. ep.), and lumbar (lum. ep.) vertebrae epiphyses, the frontal/parietal suture (F/Pa), the nasal/frontal suture (N/F), the Occipital bones (Occipt.), the premaxilla/maxilla suture (Pm/Mx), the radius and ulna epiphyses (R/U), and the squamosal/parietal suture (SQ/PA).

Species	Specimen	SQ/PA	Occipt.	dors. C1	F/Pa	cerv. ep.	caud. ep.	thor. ep.	lum. ep.	BO/BS	Pm/Mx	N/F	R/U
Dorudon atrox	UM 100142	0	0	0	0	-	-	-	-	0	-	0	-
Dorudon atrox	UM 100139	-	1	-	2	-	-	-	-	2	-	0	-
Dorudon atrox	UM 94811	-	-	-	2	-	-	-	-	-	0	0	-
Dorudon atrox	UM 94814	2	2	2	2	2	-	0	-	2	0	0	-
Dorudon atrox	UM 93220	2	2	2	2	2	-	0	0	2	0	2	-
Tutcetus rayanensis	**MUVP 501**	**2**	**2**	**2**	**2**	**2**	**-**	**-**	**-**	**-**	**-**	**2**	**-**
Dorudon atrox	UM 101222	2	2	2	2	2	2	2	-	2	2	2	1
Dorudon atrox	UM 101215	2	2	2	2	2	2	2	2	2	-	-	2

Supplementary Table 6. Eruption sequence of dorudontine archaeocetes compared to *Tutcetus*. The sequence is read from left to right. Those teeth shown in the same column erupt at approximately the same time. D^D and D_D represent the upper and lower deciduous dentitions. The eruption sequence of dorudontine archaeocetes is from Uhen[31].

***Dorudon* and *Zygorhiza* eruption order**								
					I^1	I^2	I^3	C^1
**D^D**		M^1	dP^1	M^2	P^4	P^3	P^2	P^1
**D_D**	M_1	dP_1	M_2	M_3	P_4	P_3	P_2	P_1
					I_1	I_2	I_3	C_1

***Tutcetus* eruption order**								
					I^1	I^2	I^3	
**D^D**		M^1	P^1	M^2	P^4	P^3	C^1	P^2
**D_D**	M_1	P_1	M_2	M_3	P_4	P_3	C_1	P_2
					I_1	I_2	I_3	

Supplementary Table 7. Epiphyseal fusion stage of the holotype of *Tutcetus rayanensis* (MUVP 501) compared to the holotype of *Chrysocetus healyorum* (SCSM 87.195), juvenile *Dorudon atrox* (UM 93220), and juvenile *Zygorhiza kochii* (USNM 16639). The form of the table is after Uhen and Gingerich[32].

		Dorudon atrox **(UM 93220)**	*Chrysocetus healyorum* **(SCSM 87.195)**	*Zygorhiza kochii* **(USNM 16639)**	*Tutcetus rayanensis* **(MUVP 501)**
**Cranial**	Premaxilla-Maxilla	-	-	Complete	-
	Frontal-Parietal	Initial	Initial	-	Complete
	Basisphenoid-Basioccipital	-	-	Complete	-
	Squamosal-Parietal	Partial	Initial	Complete	Complete
	Parietal-Occipital	Partial	Initial	Complete	Complete
	Supraoccipital-Exoccipital	Partial	-	Complete	Complete
	Exoccipital- Basioccipital	Partial	-	Complete	-
	M2 eruption	Partial	Complete	Complete	Complete
	Last permanent tooth	-	Complete	Partial	Complete
**Atlas**	Atlas dorsal arch	Complete	Partial	Complete	Complete
	Atlas ventral arch	Complete	Partial	Complete	Complete

Supplementary Table 8. Posterior probabilities of reconstructed ancestral states for character 3 (skull length) using MBASR. Node labels are as in Supplementary Figure 15; nodes 86 and 87 are highlighted indicating the clade (87) containing *Tutcetus* and *Ocucajea*, and the more inclusive clade including *Chrysocetus* (86). Using the criteria that MBASR suggests for the cutoffs, the changes are as follows: Character 3 (Skull length): node 86 (1/2; i.e. variably moderate/elongate) to node 87 (1; moderate).

Node no.	Short	Moderate length	Elongate
**node48**	0.09176873	0.34989087	0.55834040
**node49**	0.07301977	0.35284952	0.57413071
**node50**	0.05815282	0.35566650	0.58618068
**node51**	0.10588947	0.35417943	0.53993110
**node52**	0.02021528	0.35024932	0.62953540
**node53**	0.00073462	0.04323381	0.95603157
**node54**	0.00036975	0.02660918	0.97302107
**node55**	0.00007730	0.01166748	0.98825522
**node56**	0.00062379	0.02620707	0.97316914
**node57**	0.02077987	0.41809175	0.56112838
**node58**	0.01815734	0.47686825	0.50497441
**node59**	0.02335386	0.47385652	0.50278962
**node60**	0.02114952	0.42844464	0.55040584
**node61**	0.01658663	0.38671379	0.59669958
**node62**	0.00931472	0.34514463	0.64554065
**node63**	0.01454805	0.27755524	0.70789671
**node64**	0.01214662	0.19878834	0.78906504
**node65**	0.05847776	0.31072565	0.63079659
**node66**	0.08966579	0.32395613	0.58637808
**node67**	0.00810474	0.36933734	0.62255792
**node68**	0.03142202	0.45311186	0.51546612
**node69**	0.04233864	0.49547218	0.46218918
**node70**	0.08758768	0.45746695	0.45494537
**node71**	0.19495490	0.38396888	0.42107622
**node72**	0.00543947	0.35304434	0.64151619
**node73**	0.00374058	0.35837860	0.63788082
**node74**	0.00949864	0.34539644	0.64510492
**node75**	0.01597478	0.30573985	0.67828537
**node76**	0.01820027	0.24895840	0.73284133
**node77**	0.01412294	0.18939747	0.79647959
**node78**	0.03805783	0.55968806	0.40225411
**node79**	0.12022502	0.60301621	0.27675877
**node80**	0.13705037	0.61328434	0.24966529
**node81**	0.26400816	0.51893462	0.21705722
**node82**	0.35289091	0.51006934	0.13703975
**node83**	0.37915846	0.58052318	0.04031836
**node84**	0.00885203	0.67668817	0.31445980
**node85**	0.00476778	0.39298790	0.60224432
**node86**	0.01829649	0.55418693	0.42751658
**node87**	0.02011031	0.71415358	0.26573611
**node88**	0.00384443	0.26918576	0.72696981
**node89**	0.00074272	0.09334177	0.90591551
**node90**	0.00033502	0.05898617	0.94067881
**node91**	0.00071649	0.08571228	0.91357123
**node92**	0.00234681	0.07988162	0.91777157
**node93**	0.03468055	0.72567399	0.23964546

Supplementary Table 9. Posterior probabilities of reconstructed ancestral states for character 6 [posterior margin of nasal in relation to the PMM (posterior margin of maxilla)] using MBASR. Node labels are as in Supplementary Figure 15; nodes 86 and 87 are highlighted indicating the clade (87) containing *Tutcetus* and *Ocucajea*, and the more inclusive clade including *Chrysocetus* (86). Using the criteria that MBASR suggests for the cutoffs, the changes are as follows: node 86 (variably posterior, anterior, or at same level as PMM) to node 87 (anterior or at same level as PMM)

Node No.	Posterior to PMM	Anterior or at the same level as PMM
**node48**	0.95877659	0.04122341
**node49**	0.97389066	0.02610934
**node50**	0.99597176	0.00402824
**node51**	0.99596005	0.00403995
**node52**	0.99813679	0.00186321
**node53**	0.91519379	0.08480621
**node54**	0.88161570	0.11838430
**node55**	0.86414581	0.13585419
**node56**	0.81999168	0.18000832
**node57**	0.99979161	0.00020839
**node58**	0.99992353	0.00007647
**node59**	0.99996768	0.00003232
**node60**	0.99997287	0.00002713
**node61**	0.99997648	0.00002352
**node62**	0.99992975	0.00007025
**node63**	0.99984133	0.00015867
**node64**	0.99980472	0.00019528
**node65**	0.99575647	0.00424353
**node66**	0.94226293	0.05773707
**node67**	0.98791420	0.01208580
**node68**	0.98313964	0.01686036
**node69**	0.98269564	0.01730436
**node70**	0.94337746	0.05662254
**node71**	0.85092562	0.14907438
**node72**	0.93876470	0.06123530
**node73**	0.59491099	0.40508901
**node74**	0.44930887	0.55069113
**node75**	0.45794279	0.54205721
**node76**	0.46070386	0.53929614
**node77**	0.46399108	0.53600892
**node78**	0.03224361	0.96775639
**node79**	0.01086007	0.98913993
**node80**	0.01087990	0.98912010
**node81**	0.00311166	0.99688834
**node82**	0.00195644	0.99804356
**node83**	0.00648008	0.99351992
**node84**	0.00068892	0.99931108
**node85**	0.55159500	0.44840500
**node86**	0.35853560	0.64146440
**node87**	0.22712565	0.77287435
**node88**	0.60376326	0.39623674
**node89**	0.97135761	0.02864239
**node90**	0.99816144	0.00183856
**node91**	0.99950627	0.00049373
**node92**	0.97772715	0.02227285
**node93**	0.99655452	0.00344548

Supplementary Table 10. Posterior probabilities of reconstructed ancestral states for character 16 (nasal process of the frontal) using MBASR. Node labels are as in Supplementary Figure 16; nodes 86 and 87 are highlighted indicating the clade (87) containing *Tutcetus* and *Ocucajea*, and the more inclusive clade including *Chrysocetus* (86). Using the criteria that MBASR suggests for the cutoffs, the changes are as follows: node 85 (2; frontal with a large nasal process) to node 86 (0/2; variably absent or frontal with a large nasal process), node 86 (0/2; variably absent or frontal with a large nasal process) to node 87 (0; absent).

Node No.	Absent	Present, frontal with a small (< 3 mm) process	Frontal with a large nasal process
**node48**	0.04609872	0.90773154	0.04616974
**node49**	0.02520978	0.94934541	0.02544481
**node50**	0.00704447	0.98551344	0.00744209
**node51**	0.00623528	0.98734303	0.00642169
**node52**	0.00184921	0.99577866	0.00237213
**node53**	0.01821898	0.94458872	0.03719230
**node54**	0.01675199	0.93719909	0.04604892
**node55**	0.01332881	0.93805245	0.04861874
**node56**	0.05931995	0.48064225	0.46003780
**node57**	0.00076084	0.99834793	0.00089123
**node58**	0.00007272	0.99984201	0.00008527
**node59**	0.00015294	0.99962974	0.00021732
**node60**	0.00027344	0.99922778	0.00049878
**node61**	0.00085276	0.99640201	0.00274523
**node62**	0.00405653	0.97543986	0.02050361
**node63**	0.00806413	0.97093254	0.02100333
**node64**	0.00997562	0.97280802	0.01721636
**node65**	0.07466563	0.84027917	0.08505520
**node66**	0.12458874	0.74330286	0.13210840
**node67**	0.02884433	0.49601003	0.47514564
**node68**	0.02526571	0.45313801	0.52159628
**node69**	0.02289585	0.44065850	0.53644565
**node70**	0.01381479	0.08419218	0.90199303
**node71**	0.03548680	0.05756591	0.90694729
**node72**	0.05955151	0.38053101	0.55991748
**node73**	0.09737652	0.34951020	0.55311328
**node74**	0.14200214	0.42714486	0.43085300
**node75**	0.15725857	0.41869855	0.42404288
**node76**	0.17859670	0.41068859	0.41071471
**node77**	0.21177071	0.38524329	0.40298600
**node78**	0.23826591	0.61542415	0.14630994
**node79**	0.75150737	0.16794846	0.08054417
**node80**	0.87550476	0.07876215	0.04573309
**node81**	0.96790097	0.01881058	0.01328845
**node82**	0.98344623	0.00881074	0.00774303
**node83**	0.97190346	0.01433929	0.01375725
**node84**	0.00571279	0.99010802	0.00417919
**node85**	0.16845424	0.22445772	0.60708804
**node86**	0.46595343	0.17800333	0.35604324
**node87**	0.62927119	0.11394249	0.25678632
**node88**	0.06895783	0.05692515	0.87411702
**node89**	0.07453574	0.04791924	0.87754502
**node90**	0.02420464	0.03823426	0.93756110
**node91**	0.01660379	0.05456279	0.92883342
**node92**	0.40189756	0.08305918	0.51504326
**node93**	0.00369091	0.99261235	0.00369674

Supplementary Table 11. Posterior probabilities of reconstructed ancestral states for character 19 (supraorbital process) using MBASR. Node labels are as in Supplementary Figure 17; nodes 86 and 87 are highlighted indicating the clade (87) containing *Tutcetus* and *Ocucajea*, and the more inclusive clade including *Chrysocetus* (86). Using the criteria that MBASR suggests for the cutoffs, the changes are as follows: node 86 (3; greatly enlarged) to node 87 (2; large).

Node No.	Absent	Present but small	Large	Greatly enlarged
**node48**	0.83072766	0.15872267	0.00989288	0.00065679
**node49**	0.84648055	0.14688689	0.00634398	0.00028858
**node50**	0.86635455	0.13112222	0.00250420	0.00001903
**node51**	0.98391085	0.01603758	0.00005130	0.00000027
**node52**	0.79010609	0.20480716	0.00507281	0.00001394
**node53**	0.97879584	0.02112457	0.00007932	0.00000027
**node54**	0.98855014	0.01138212	0.00006719	0.00000055
**node55**	0.99769470	0.00230287	0.00000243	0.00000000
**node56**	0.99918620	0.00081290	0.00000090	0.00000000
**node57**	0.31127156	0.58555350	0.10187671	0.00129823
**node58**	0.10211242	0.73263201	0.16321007	0.00204550
**node59**	0.00285094	0.14386291	0.81144511	0.04184104
**node60**	0.00017714	0.02799946	0.78537962	0.18644378
**node61**	0.00000080	0.00047150	0.10722203	0.89230567
**node62**	0.00000001	0.00001854	0.02987806	0.97010339
**node63**	0.00000005	0.00001723	0.01177008	0.98821264
**node64**	0.00000000	0.00000267	0.00310993	0.99688740
**node65**	0.00001352	0.00070007	0.03603036	0.96325605
**node66**	0.00005675	0.00143553	0.04971738	0.94879034
**node67**	0.00000001	0.00001577	0.02380958	0.97617464
**node68**	0.00000141	0.00025329	0.07682038	0.92292492
**node69**	0.00000041	0.00019515	0.09282361	0.90698083
**node70**	0.00000062	0.00031190	0.07386734	0.92582014
**node71**	0.00175078	0.07001541	0.51629346	0.41194035
**node72**	0.00000001	0.00001288	0.01597922	0.98400789
**node73**	0.00000001	0.00000726	0.01004932	0.98994341
**node74**	0.00000231	0.00017183	0.01820844	0.98161742
**node75**	0.00021335	0.00320031	0.05862635	0.93795999
**node76**	0.00091793	0.00968478	0.10566227	0.88373502
**node77**	0.00245963	0.01930250	0.14942889	0.82880898
**node78**	0.00000039	0.00002239	0.00561326	0.99436396
**node79**	0.00000014	0.00002129	0.00622352	0.99375505
**node80**	0.00000024	0.00001798	0.00519149	0.99479029
**node81**	0.00000007	0.00001571	0.00529752	0.99468670
**node82**	0.00000001	0.00000471	0.00314323	0.99685205
**node83**	0.00000001	0.00000263	0.00198910	0.99800826
**node84**	0.00000000	0.00000080	0.00101654	0.99898266
**node85**	0.00000001	0.00001012	0.01588851	0.98410136
**node86**	0.00000007	0.00005023	0.09527975	0.90466995
**node87**	0.00000020	0.00030967	0.92721218	0.07247795
**node88**	0.00000001	0.00000386	0.00304469	0.99695144
**node89**	0.00000000	0.00000106	0.00146046	0.99853848
**node90**	0.00000000	0.00000064	0.00095979	0.99903957
**node91**	0.00000000	0.00000084	0.00091146	0.99908770
**node92**	0.00000138	0.00010561	0.01121821	0.98867480
**node93**	0.05125668	0.86597549	0.08158749	0.00118034

Supplementary Table 12. Posterior probabilities of reconstructed ancestral states for character 23 (posterior edge of the postorbital process) using MBASR. Node labels are as in Supplementary Figure 17; nodes 86 and 87 are highlighted indicating the clade (87) containing *Tutcetus* and *Ocucajea*, and the more inclusive clade including *Chrysocetus* (86). Using the criteria that MBASR suggests for the cutoffs, the changes are as follows: node 86 (2; forms an acute angle with the sagittal crest) to node 87 (1; oriented at approximately 90º to the sagittal crest).

Node no.	Forms a highly obtuse angle with the sagittal crest	Oriented at approximately 90º to the sagittal crest	Forms an acute angle with the sagittal crest
**node48**	0.85685836	0.13810559	0.00503605
**node49**	0.86699266	0.13025345	0.00275389
**node50**	0.87831178	0.12095404	0.00073418
**node51**	0.98639808	0.01357814	0.00002378
**node52**	0.79142291	0.20826966	0.00030743
**node53**	0.90222497	0.09607430	0.00170073
**node54**	0.95319099	0.04585813	0.00095088
**node55**	0.99184671	0.00812550	0.00002779
**node56**	0.99162545	0.00831641	0.00005814
**node57**	0.05236149	0.94689440	0.00074411
**node58**	0.01032758	0.98918610	0.00048632
**node59**	0.00190825	0.99790210	0.00018965
**node60**	0.00044777	0.99941301	0.00013922
**node61**	0.00011040	0.99961843	0.00027117
**node62**	0.00004414	0.99879483	0.00116103
**node63**	0.00022799	0.99925985	0.00051216
**node64**	0.00037781	0.99919051	0.00043168
**node65**	0.00378327	0.99221976	0.00399697
**node66**	0.02178500	0.95624201	0.02197299
**node67**	0.00038053	0.95907543	0.04054404
**node68**	0.00153558	0.94604012	0.05242430
**node69**	0.00085282	0.94511048	0.05403670
**node70**	0.00072766	0.83923340	0.16003894
**node71**	0.00193570	0.98365017	0.01441413
**node72**	0.00064467	0.79874784	0.20060749
**node73**	0.00024406	0.34120659	0.65854935
**node74**	0.00223899	0.26055692	0.73720409
**node75**	0.01587299	0.26848782	0.71563919
**node76**	0.03005018	0.27652774	0.69342208
**node77**	0.04782479	0.29256384	0.65961137
**node78**	0.00047628	0.05713087	0.94239285
**node79**	0.00333613	0.09166470	0.90499917
**node80**	0.00541553	0.11791290	0.87667157
**node81**	0.81594480	0.16462114	0.01943406
**node82**	0.94161251	0.05805350	0.00033399
**node83**	0.91161543	0.08821040	0.00017417
**node84**	0.00000110	0.00150589	0.99849301
**node85**	0.00013917	0.26671230	0.73314853
**node86**	0.00014806	0.31005172	0.68980022
**node87**	0.00021362	0.97302206	0.02676432
**node88**	0.00004365	0.01944895	0.98050740
**node89**	0.00000284	0.00306417	0.99693299
**node90**	0.00000020	0.00053106	0.99946874
**node91**	0.00000020	0.00045242	0.99954738
**node92**	0.00004065	0.00701234	0.99294701
**node93**	0.01235933	0.98093891	0.00670176

Supplementary Table 13. Posterior probabilities of reconstructed ancestral states for character 101 (accessory denticles on P^3) using MBASR. Node labels are as in Supplementary Figure 19; nodes 86 and 87 are highlighted indicating the clade (87) containing *Tutcetus* and *Ocucajea*, and the more inclusive clade including *Chrysocetus* (86). Using the criteria that MBASR suggests for the cutoffs the changes are as follows: node 86 (3; equal number of denticles on both edges) to node 87 (2; more denticles on the distal edge).

Node no.	Tooth without denticles	More denticles on the mesial edge	More denticles on the distal edge	An equal number of denticles on both edges
**node48**	0.96072106	0.01309298	0.01309298	0.01309298
**node49**	0.98115292	0.00628236	0.00628236	0.00628236
**node50**	0.99899617	0.00033461	0.00033461	0.00033461
**node51**	0.99984931	0.00005023	0.00005023	0.00005023
**node52**	0.99994897	0.00001701	0.00001701	0.00001701
**node53**	0.99974278	0.00008574	0.00008574	0.00008574
**node54**	0.99893614	0.00035462	0.00035462	0.00035462
**node55**	0.99991114	0.00002962	0.00002962	0.00002962
**node56**	0.99989329	0.00003557	0.00003557	0.00003557
**node57**	0.99998077	0.00000641	0.00000641	0.00000641
**node58**	0.99999378	0.00000207	0.00000207	0.00000208
**node59**	0.99997894	0.00000699	0.00000699	0.00000708
**node60**	0.99996577	0.00001108	0.00001111	0.00001204
**node61**	0.99996554	0.00000844	0.00000873	0.00001729
**node62**	0.99974905	0.00002591	0.00003186	0.00019318
**node63**	0.99975229	0.00006972	0.00007111	0.00010688
**node64**	0.99769377	0.00076018	0.00076109	0.00078496
**node65**	0.99671281	0.00108788	0.00108872	0.00111059
**node66**	0.93491720	0.02168711	0.02168789	0.02170780
**node67**	0.97659010	0.00127699	0.00200240	0.02013051
**node68**	0.98404586	0.00170119	0.00210820	0.01214475
**node69**	0.98775884	0.00145463	0.00172039	0.00906614
**node70**	0.99873115	0.00021768	0.00024175	0.00080942
**node71**	0.99276305	0.00235033	0.00235659	0.00253003
**node72**	0.41984779	0.00968887	0.03083524	0.53962810
**node73**	0.06043872	0.00380491	0.03698027	0.89877610
**node74**	0.04854265	0.00611519	0.03445541	0.91088675
**node75**	0.05994166	0.01966366	0.04614099	0.87425369
**node76**	0.07308846	0.03589115	0.06023413	0.83078626
**node77**	0.08823352	0.05464820	0.07631880	0.78079948
**node78**	0.01226684	0.00364791	0.02350567	0.96057958
**node79**	0.01311793	0.00868540	0.05473711	0.92345956
**node80**	0.01337098	0.00952753	0.04936378	0.92773771
**node81**	0.05056201	0.04754490	0.08008156	0.82181153
**node82**	0.08377533	0.08135774	0.10748401	0.72738292
**node83**	0.10122876	0.09908380	0.12203549	0.67765195
**node84**	0.00900177	0.00459175	0.01381241	0.97259407
**node85**	0.01134381	0.00225286	0.05196538	0.93443795
**node86**	0.00637993	0.00194496	0.06981103	0.92186408
**node87**	0.00059512	0.00051008	0.98515355	0.01374125
**node88**	0.00414392	0.00673457	0.05274762	0.93637389
**node89**	0.00189438	0.01262251	0.01582032	0.96966279
**node90**	0.00216144	0.04779626	0.00819595	0.94184635
**node91**	0.00122721	0.96570136	0.00144234	0.03162909
**node92**	0.00270541	0.01012498	0.01036552	0.97680409
**node93**	0.99985609	0.00004797	0.00004797	0.00004797

Supplementary Table 14. Posterior probabilities of reconstructed ancestral states for character 113 (P^3 length) using MBASR. Node labels are as in Supplementary Figure 15; nodes 86 and 87 are highlighted indicating the clade (87) containing *Tutcetus* and *Ocucajea*, and the more inclusive clade including *Chrysocetus* (86). Using the criteria that MBASR suggests for the cutoffs, the changes are as follows: node 85 (2; longer than P^4) to node 86 (1/2; variably subequal to P^4 or longer than P^4), node 86 (1/2) to node 87 (1; subequal to P^4).

Node No.	Shorter than P4	Subequal to P4	Longer than P4
**node48**	0.02501230	0.04362084	0.93136686
**node49**	0.01351244	0.03096119	0.95552637
**node50**	0.00327620	0.02010203	0.97662177
**node51**	0.00301607	0.01020434	0.98677959
**node52**	0.00206876	0.02472415	0.97320709
**node53**	0.00325799	0.95815709	0.03858492
**node54**	0.00219727	0.98279563	0.01500710
**node55**	0.00025128	0.99852389	0.00122483
**node56**	0.00006471	0.99984070	0.00009459
**node57**	0.00107069	0.00925022	0.98967909
**node58**	0.00020150	0.00134486	0.99845364
**node59**	0.00035048	0.00098442	0.99866510
**node60**	0.00005688	0.00011573	0.99982739
**node61**	0.00002244	0.00002988	0.99994768
**node62**	0.00001534	0.00003486	0.99994980
**node63**	0.00007915	0.00008192	0.99983893
**node64**	0.00009128	0.00009165	0.99981707
**node65**	0.00231729	0.00231888	0.99536383
**node66**	0.03240171	0.03240314	0.93519515
**node67**	0.00025075	0.00212055	0.99762870
**node68**	0.00048018	0.00097269	0.99854713
**node69**	0.00017643	0.00030649	0.99951708
**node70**	0.00014437	0.00016048	0.99969515
**node71**	0.00484920	0.00485415	0.99029665
**node72**	0.00273308	0.04635902	0.95090790
**node73**	0.00241306	0.07228330	0.92530364
**node74**	0.01479618	0.14998973	0.83521409
**node75**	0.03337163	0.15977310	0.80685527
**node76**	0.05587167	0.17160773	0.77252060
**node77**	0.07990621	0.18472929	0.73536450
**node78**	0.01834710	0.33956662	0.64208628
**node79**	0.03691724	0.23520380	0.72787896
**node80**	0.03813144	0.20959339	0.75227517
**node81**	0.03270792	0.12098253	0.84630955
**node82**	0.08917102	0.15989408	0.75093490
**node83**	0.11631326	0.17860235	0.70508439
**node84**	0.00111630	0.98781921	0.01106449
**node85**	0.00306416	0.08422085	0.91271499
**node86**	0.01861356	0.40666595	0.57472049
**node87**	0.01763663	0.67092854	0.31143483
**node88**	0.00102403	0.00710878	0.99186719
**node89**	0.00127202	0.00487361	0.99385437
**node90**	0.00016819	0.00038711	0.99944470
**node91**	0.00008456	0.00010419	0.99981125
**node92**	0.03476179	0.03796269	0.92727552
**node93**	0.00015652	0.00022577	0.99961771

Supplementary Table 15. Posterior probabilities of reconstructed ancestral states for character 118 (enamel of premolars) using MBASR. Node labels are as in Supplementary Figure 20; nodes 86 and 87 are highlighted indicating the clade (87) containing *Tutcetus* and *Ocucajea*, and the more inclusive clade including *Chrysocetus* (86). Using the criteria that MBASR suggests for the cutoffs, the changes are as follows: node 85 (0; ornamented) to node 86 (1; smooth).

Node No.	Ornamented	Smooth
**node48**	0.96320824	0.03679176
**node49**	0.97843635	0.02156365
**node50**	0.99425755	0.00574245
**node51**	0.99528463	0.00471537
**node52**	0.99642717	0.00357283
**node53**	0.93163469	0.06836531
**node54**	0.89856115	0.10143885
**node55**	0.87888165	0.12111835
**node56**	0.84183296	0.15816704
**node57**	0.99855866	0.00144134
**node58**	0.99982372	0.00017628
**node59**	0.99991525	0.00008475
**node60**	0.99989517	0.00010483
**node61**	0.99956482	0.00043518
**node62**	0.99993530	0.00006470
**node63**	0.99983709	0.00016291
**node64**	0.99985281	0.00014719
**node65**	0.99566796	0.00433204
**node66**	0.95820992	0.04179008
**node67**	0.99990055	0.00009945
**node68**	0.99922208	0.00077792
**node69**	0.99972255	0.00027745
**node70**	0.99967702	0.00032298
**node71**	0.99062782	0.00937218
**node72**	0.99963243	0.00036757
**node73**	0.99881944	0.00118056
**node74**	0.99782192	0.00217808
**node75**	0.99117382	0.00882618
**node76**	0.98253808	0.01746192
**node77**	0.67012696	0.32987304
**node78**	0.99734975	0.00265025
**node79**	0.99769453	0.00230547
**node80**	0.99805345	0.00194655
**node81**	0.99194081	0.00805919
**node82**	0.92854878	0.07145122
**node83**	0.89671174	0.10328826
**node84**	0.99141582	0.00858418
**node85**	0.97813742	0.02186258
**node86**	0.05800837	0.94199163
**node87**	0.03487550	0.96512450
**node88**	0.99851177	0.00148823
**node89**	0.99976897	0.00023103
**node90**	0.99981209	0.00018791
**node91**	0.99984995	0.00015005
**node92**	0.99975677	0.00024323
**node93**	0.99968606	0.00031394

Supplementary Table 16. Posterior probabilities of reconstructed ancestral states for character 119 (prominent denticulate cingula on $P^{3\text{-}4}$) using MBASR. Node labels are as in Supplementary Figure 20; nodes 86 and 87 are highlighted indicating the clade (87) containing *Tutcetus* and *Ocucajea*, and the more inclusive clade including *Chrysocetus* (86). Using the criteria that MBASR suggests for the cutoffs, the changes are as follows: node 85 (0; absent) to node 86 (1; present).

Node No.	Absent	Present
**node48**	0.94556497	0.05443503
**node49**	0.96658569	0.03341431
**node50**	0.98764375	0.01235625
**node51**	0.98776061	0.01223939
**node52**	0.99099705	0.00900295
**node53**	0.89629398	0.10370602
**node54**	0.86364410	0.13635590
**node55**	0.83861477	0.16138523
**node56**	0.79218538	0.20781462
**node57**	0.99697390	0.00302610
**node58**	0.99948363	0.00051637
**node59**	0.99940476	0.00059524
**node60**	0.99882824	0.00117176
**node61**	0.99904198	0.00095802
**node62**	0.99992751	0.00007249
**node63**	0.99980076	0.00019924
**node64**	0.99964182	0.00035818
**node65**	0.99361114	0.00638886
**node66**	0.95428680	0.04571320
**node67**	0.99977051	0.00022949
**node68**	0.99871534	0.00128466
**node69**	0.99962598	0.00037402
**node70**	0.99946398	0.00053602
**node71**	0.98771076	0.01228924
**node72**	0.99507687	0.00492313
**node73**	0.98577592	0.01422408
**node74**	0.98073646	0.01926354
**node75**	0.95072771	0.04927229
**node76**	0.91784151	0.08215849
**node77**	0.93470347	0.06529653
**node78**	0.99344435	0.00655565
**node79**	0.96100528	0.03899472
**node80**	0.94514907	0.05485093
**node81**	0.52188334	0.47811666
**node82**	0.51765043	0.48234957
**node83**	0.51768639	0.48231361
**node84**	0.99930325	0.00069675
**node85**	0.76247781	0.23752219
**node86**	0.03402944	0.96597056
**node87**	0.01112955	0.98887045
**node88**	0.79217218	0.20782782
**node89**	0.99326260	0.00673740
**node90**	0.99866788	0.00133212
**node91**	0.99876912	0.00123088
**node92**	0.99824159	0.00175841
**node93**	0.99932054	0.00067946

Supplementary Table 17. Eocene archaeocete body mass (BM) estimates using the breadth across the occipital condyles (OCW).

Taxon	Age (Ma)	Specimen	OCW (mm)	BM (kg)	OCW reference
**Pakicetidae**					
Ichthyolestes pinfoldi	48	H-GSP 98134	43.6	37	Nummela et al.[33]
Nalacetus ratimitus	48	H-GSP 96386	47.1	47	Nummela et al.[33]
Pakicetus attocki	48	H-GSP 96231	52.7	67	Nummela et al.[33]
Pakicetus inachus	48	GSP-UM 084	53.7	71	Nummela et al.[33]
**Ambulocetidae**					
Ambulocetus natans	47.5	H-GSP 18507	79.3	239	Nummela et al.[33]
**Remingtonocetidae**					
Remingtonocetus harudiensis	46	IITR-SB 2770	87	320	Bajpai et al.[34]
Remingtonocetus sp.	46	IITR-SB 2529	95.7	432	Nummela et al.[33]
Dalanistes ahmedi	46.5	GSP-UM 3106	104.8	574	Waugh and Thewissen[35]
**Protocetidae**					
Rodhocetus kasrani	46.5	GSP-UM 3012	88.7	340	Waugh and Thewissen[35]
Artiocetus clavis	47	GSP-UM 3458	79.65	243	This study
Makaracetus bidens	43	GSP-UM 3570	77.65	224	Gingerich[36]
**Basilosauridae**					
Tutcetus rayanensis	41	MUVP 501	73.3	187	This study
Saghacetus osiris	34	BMNH 10228	91.8	379	Kellogg[2]
Zygorhiza kochii	37	USNM 16639	112	707	Kellogg[2]
Zygorhiza kochii	39	FMNH PM-459	120	877	Gingerich[17]
Dorudon atrox	38	UM 93220	105	577	Uhen[1]
Dorudon atrox	37	BMNH M 9266	107	613	Uhen[1]
Dorudon atrox	38	BMNH M 10173	111.8	703	Uhen[1]
Dorudon atrox	37	UM 100142	116	789	Uhen[1]
Dorudon atrox	37.5	CGM 42183	121	901	Uhen[1]
Dorudon atrox	37	UM 101215	121.6	915	Uhen[1]
Dorudon atrox	36	UM 101222	126	1022	Uhen[1]

Supplementary references

1. Uhen, M. D. Form, function, and anatomy of *Dorudon atrox* (Mammalia, Cetacea): an archaeocete from the middle to late Eocene of Egypt. *Univ. Michigan Mus. Paleontol. Papers Paleontol.* **34**, 1-222 (2004).
2. Kellogg, R. A review of the Archaeoceti. *Carnegie Inst. Washingt*. **482**, 1–366 (1936).

3. Dames, W. *Über Zeuglodonten aus Aegypten und die Beziehungen der Archaeoceten zu den übrigen Cetaceen*. (1894).
4. Gingerich, P. D., Amane, A. & Zouhri, S. Skull and partial skeleton of a new pachycetine genus (Cetacea, Basilosauridae) from the Aridal Formation, Bartonian middle Eocene, of southwestern Morocco. *PLOS ONE* **17,** e0276110 (2022).
5. Gingerich, P. D. *Stromerius nidensis*, new archaeocete (Mammalia, Cetacea) from the upper Eocene Qasr el-Sagha Formation, Fayum, Egypt. *Contrib. Mus. Paleontol., Univ. Mich*. **31**, 363–378 (2007).
6. Helal, S. & Holcová, K. Response of foraminiferal assemblages on the middle Eocene climatic optimum and following climatic transition in the shallow tropical sea (the south Fayoum area, Egypt). *Arabian Journal of Geosciences* **10**, 43 (2017). https://doi.org:10.1007/s12517-016-2818-7
7. Strougo, A., Faris, M., Abul-Nasr, R. A., Gingerich, P. D. & Haggag, M. A. Planktonic foraminifera and calcareous nannofossil biostratigraphy through the middle to late Eocene transition at Wadi Hitan, Fayum Province, Egypt. *Contrib. Mus. Paleontol., Univ. Mich*. **32**, 111–138 (2013).
8. Strougo, A. The Mokattamian stage: 125 years later. *Middle East Research Center, Ain Shams University, Cairo, Earth Science Series* **22**, 47-108 (2008).
9. Zalmout, I. & Gingerich, P. Late Eocene Sea Cows (Mammalia, Sirenia) From Wadi Al Hitan in The Western Desert of Fayum, Egypt. *Univ. Mich. Pap. Paleontol.* **37**, 1-158 (2012).
10. Peters, S., Antar, M., Zalmout, I. & Gingerich, P. Sequence stratigraphic control on preservation of late Eocene whales and other vertebrates at Wadi Al-Hitan, Egypt. *PALAIOS* **24**, 290-302 (2009). https://doi.org:10.2110/palo.2008.p08-080r
11. Andrews, C. W. *A Descriptive Catalogue of the Tertiary Vertebrata of the Fayum, Egypt* (1906).
12. Gingerich, P. D. Cetacea. *Cenozoic Mammals of Africa* 873–900 (2010). doi:10.1525/california/9780520257214.003.0045
13. Gingerich, P. D. Early Evolution of Whales. *Elwyn Simons: A Search for Origins* (eds John G. Fleagle & Christopher C. Gilbert) 107-124 (2008).
14. Gingerich, P. D. & Uhen, M. D. *Ancalecetus simonsi*, a new dorudontine archaeocete (Mammalia, Cetacea) from the early late Eocene of Wadi Hitan, Egypt. *Contrib. Mus. Paleontol., Univ. Mich*. **29**, 359–401 (1996).
15. Gingerich, P. D. Marine mammals (Cetacea and Sirenia) from the Eocene of Gebel Mokattam and Fayum, Egypt: stratigraphy, age, and paleoenvironments. *Univ. Mich. Pap. Paleontol.* **30**, 1–84 (1992).
16. Gohar, A. S. *et al.* A new protocetid whale offers clues to biogeography and feeding ecology in early cetacean evolution. *Proc. R. Soc. B* **288,** 20211368 (2021).
17. Gingerich, P. New partial skeleton and relative brain size in the late Eocene archaeocete *Zygorhiza Kochii* (Mammalia, Cetacea) from the Pachuta Marl of Alabama, with a note on contemporaneous *Pontogeneus Brachyspondylus*. *Contrib. Mus. Paleontol., Univ. Mich*. **32,** 161–188 (2015).
18. Kassegne, K. E. *et al.* First partial cranium of *Togocetus* from Kpogamé (Togo) and the protocetid diversity in the Togolese phosphate basin. *Annales de Paléontologie* **107**, 102488 (2021). https://doi.org:https://doi.org/10.1016/j.annpal.2021.102488
19. Bebej RM, Zalmout IS, Abed El-Aziz AA, Antar MSM, Gingerich PD. 2015 First remingtonocetid archaeocete (Mammalia, Cetacea) from the middle Eocene of Egypt with

implications for biogeography and locomotion in early cetacean evolution. *J. Paleontol*. **89**, 882–893. (doi:10.1017/jpa.2015.57)
20. Martínez-Cáceres, M., Lambert, O. & Muizon, C. de. The anatomy and phylogenetic affinities of *Cynthiacetus peruvianus*, a large *Dorudon*-like basilosaurid (Cetacea, Mammalia) from the Late Eocene of Peru. *Geodiversitas* **39,** 7–163 (2017).
21. O'Reilly, J. E. *et al.* Bayesian methods outperform parsimony but at the expense of precision in the estimation of phylogeny from discrete morphological data. *Biol. Letters* **12**, 20160081 (2016).
22. Puttick, M. N. *et al.* Uncertain-tree: discriminating among competing approaches to the phylogenetic analysis of phenotype data. *Proc. R. Soc. Lond. B* **284**, 20162290 (2017).
23. Geisler, J. H., Sanders, A. E. & Luo, Z.-X. A new protocetid whale (Cetacea: Archaeoceti) from the late middle Eocene of South Carolina. *American Museum Novitates* **3480,** 1 (2005).
24. Mourlam, M. J. & Orliac, M. J. Protocetid (Cetacea, Artiodactyla) bullae and petrosals from the middle Eocene locality of Kpogamé, Togo: new insights into the early history of cetacean hearing. *Journal of Systematic Palaeontology* **16,** 621–644 (2017).
25. Gingerich, P. D., Antar, M. S. M. & Zalmout, I. S. *Aegicetus gehennae*, a new late Eocene protocetid (Cetacea, Archaeoceti) from Wadi Al Hitan, Egypt, and the transition to tail-powered swimming in whales. *PLOS ONE* **14,** e0225391 (2019).
26. Steeman, M. E. A new baleen whale from the Late Miocene of Denmark and early mysticete hearing. *Palaeontology* **52**, 1169-1190 (2009).
27. Fordyce, R. E. & de Muizon, C. in *Secondary Adaptation of Tetrapods to Life in Water* (eds Mazin, J.-M. & de Buffre´nil, V.) 169–233 (Dr. Friedrich Pfeil, 2001).
28. Lambert, O. *et al.* Earliest mysticete from the late Eocene of Peru sheds new light on the origin of baleen whales. *Current Biology* **27,** (2017).
29. Muizon, C. de, Bianucci, G., Martínez-Cáceres, M. & Lambert, O. *Mystacodon selenensis*, the earliest known toothed Mysticete (Cetacea, Mammalia) from the late Eocene of Peru: Anatomy, phylogeny, and feeding adaptations. *Geodiversitas* **41,** 401 (2019).
30. Heritage, S. MBASR: Workflow-simplified ancestral state reconstruction of discrete traits with MrBayes in the R environment. *bioRxiv*, 2021.2001.2010.426107 (2021). https://doi.org:10.1101/2021.01.10.426107
31. Uhen, M. D. Replacement of deciduous first premolars and dental eruption in archaeocete whales. *Journal of Mammalogy* **81,** 123–133 (2000).
32. Uhen, M. D. & Gingerich, P. D. New genus of dorudontine archaeocete (Cetacea) from the middle-to-late Eocene of South Carolina. *Marine Mammal Science* **17,** 1–34 (2001).
33. Nummela, S., Hussain, S. T. & Thewissen, J. G. M. Cranial anatomy of Pakicetidae (Cetacea, Mammalia). *Journal of Vertebrate Paleontology* **26**, 746-759 (2006).
34. Bajpai, S., Thewissen, J. G. M. & Conley, R. W. Cranial anatomy of middle Eocene *Remingtonocetus* (Cetacea, Mammalia) from Kutch, India. *Journal of Paleontology* **85**, 703-718 (2011).
35. Waugh, D. A. & Thewissen, J. G. M. The pattern of brain-size change in the early evolution of cetaceans. *PLoS One* **16**, e0257803 (2021).
36. Gingerich, P., Zalmout, I., Ul-Haq, M. & Bhatti, M. *Makaracetus bidens*, a New Protocetid Archaeocete (Mammalia, Cetacea) from the Early Middle Eocene of Balochistan (Pakistan). *Contributions from the Museum of Paleontology, University of Michigan* **31** (2005).
