# Supplementary material for: A diminutive new basilosaurid whale reveals the trajectory of the cetacean life histories during the Eocene
Source: Commun Biol. 2023 Aug 10;6:707. doi: 10.1038/s42003-023-04986-w (PMC10415296; doi:10.1038/s42003-023-04986-w)
Supplement: Supplementary file 3 — Description of Additional Supplementary Files [file 42003_2023_4986_MOESM3_ESM.pdf]

Description of Additional Supplementary Files

File name: Supplementary Data 1

Description: Archaeocete-dominated matrix of Tutcetus rayanensis used in our phylogenetic analysis.

File name: Supplementary Data 2

Description: Pelagicete-dominated matrix of Tutcetus rayanensis used in our phylogenetic analysis.

File name: Supplementary Data 3

Description: Results from the ancestral state reconstructions (ASRs) on the allcompat trees derived from Bayesian tip-dating analyses.
