# Supplementary material for: A diminutive new basilosaurid whale reveals the trajectory of the cetacean life histories during the Eocene
Source: Commun Biol. 2023 Aug 10;6:707. doi: 10.1038/s42003-023-04986-w (PMC10415296; doi:10.1038/s42003-023-04986-w)
Supplement: Supplementary file 6 — Supplementary Data 3 [file 42003_2023_4986_MOESM6_ESM.zip › Supplementary Data 3/Supplementary Data 1_BTD_ASR/_traits_0001-0195_tree.plots.pdf]

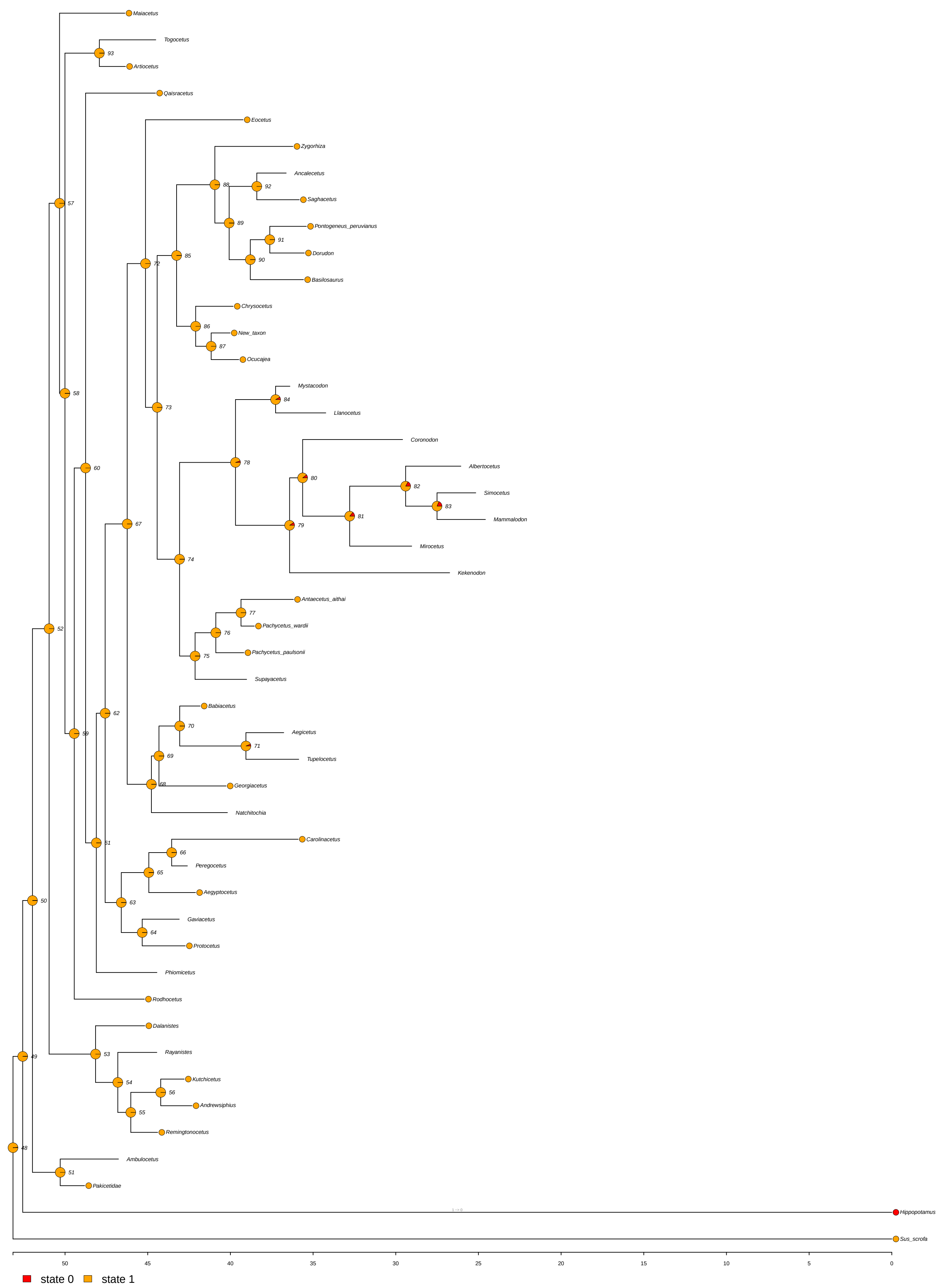
Maiacetus
Togocetus
93
Artiocetus
Qaisracetus
Eocetus
Zygorhiza
Ancalecetus
88
92
Saghacetus
57
Pontogeneus_peruvianus
89
91
Dorudon
72
85
90
Basilosaurus
Chrysocetus
86
New_taxon
87
Ocucajea
Mystacodon
84
58
73
Llanocetus
Coronodon
Albertocetus
60
78
80
82
Simocetus
83
67
79
81
Mammalodon
Mirocetus
74
Kekenodon
Antaecetus_aithai
77
Pachycetus_wardii
76
52
Pachycetus_paulsonii
75
Supayacetus
Babiacetus
62
70
Aegicetus
59
71
Tupelocetus
69
68
Georgiacetus
Natchitochia
Carolinacetus
61
66
Peregocetus
65
Aegyptocetus
50
63
Gaviacetus
64
Protocetus
Phiomicetus
Rodhocetus
Dalanistes
Rayanistes
49
53
54
Kutchicetus
56
Andrewsiphius
55
Remingtonocetus
48
Ambulocetus
51
Pakicetidae
Hippopotamus
Sus_scrofa
50
45
40
35
30
25
20
15
10
5
0
state 0
state 1

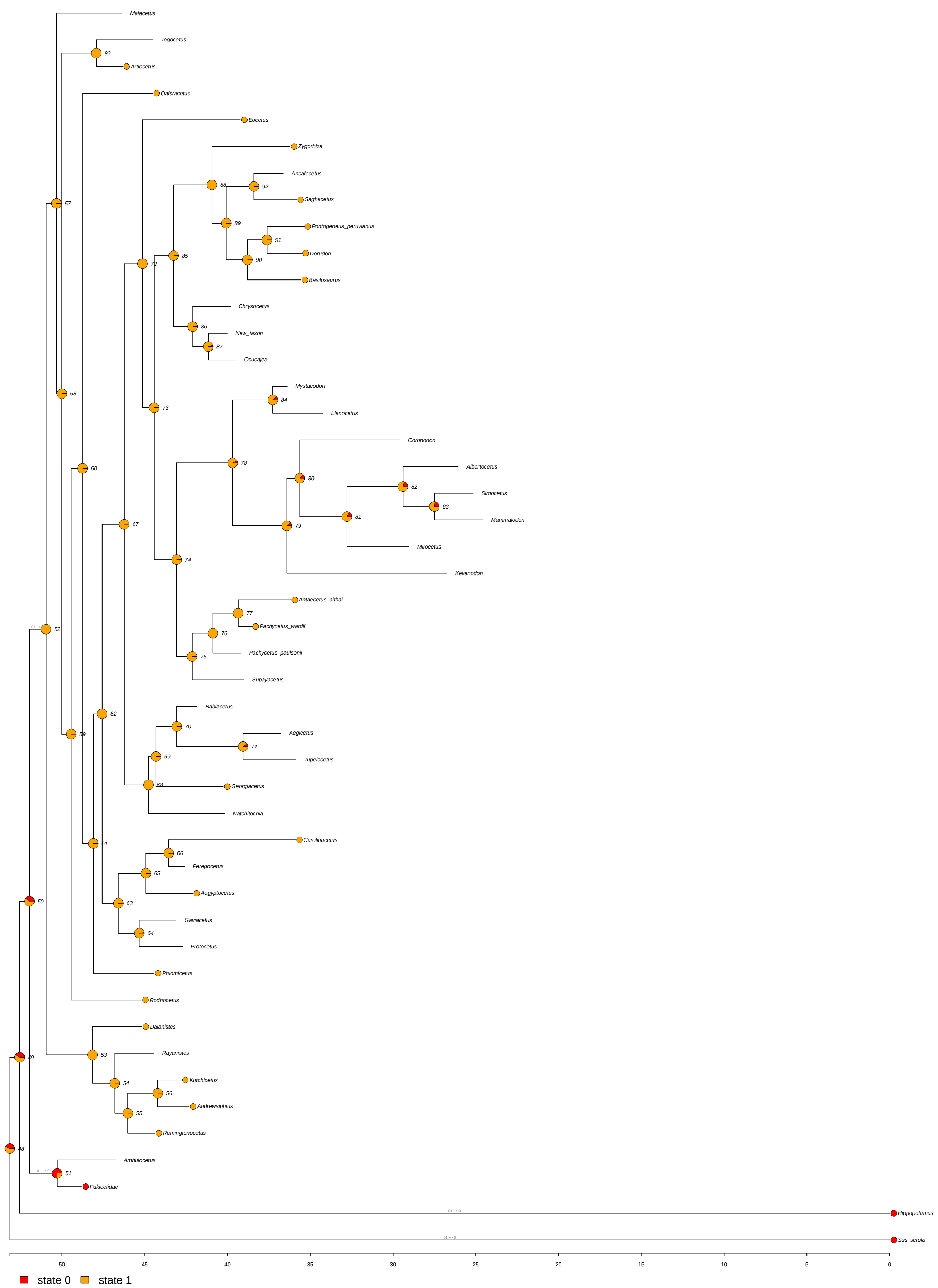

Maiacetus
Togocetus
93
Artiocetus
Qaisracetus
Eocetus
Zygorhiza
Ancalecetus
88
92
Saghacetus
89
Pontogeneus_peruvianus
91
Dorudon
90
Basilosaurus
72
85
Chrysocetus
86
New_taxon
87
Ocucajea
Mystacodon
84
Llanocetus
57
58
73
Coronodon
78
80
Albertocetus
82
Simocetus
83
Mammalodon
81
79
Mirocetus
60
67
74
Kekenodon
Antaecetus_aithai
77
Pachycetus_wardii
76
Pachycetus_paulsonii
75
Supayacetus
52
Babiacetus
62
70
Aegicetus
71
Tupelocetus
59
69
68
Georgiacetus
Natchitochia
Carolinacetus
66
Peregocetus
65
61
Aegyptocetus
50
63
Gaviacetus
64
Protocetus
Phiomicetus
Rodhocetus
Dalanistes
Rayanistes
53
49
54
Kutchicetus
56
Andrewsiphius
55
Remingtonocetus
48
Ambulocetus
51
Pakicetidae
Hippopotamus
Sus_scrofa
50
45
40
35
30
25
20
15
10
5
0
state 0
state 1

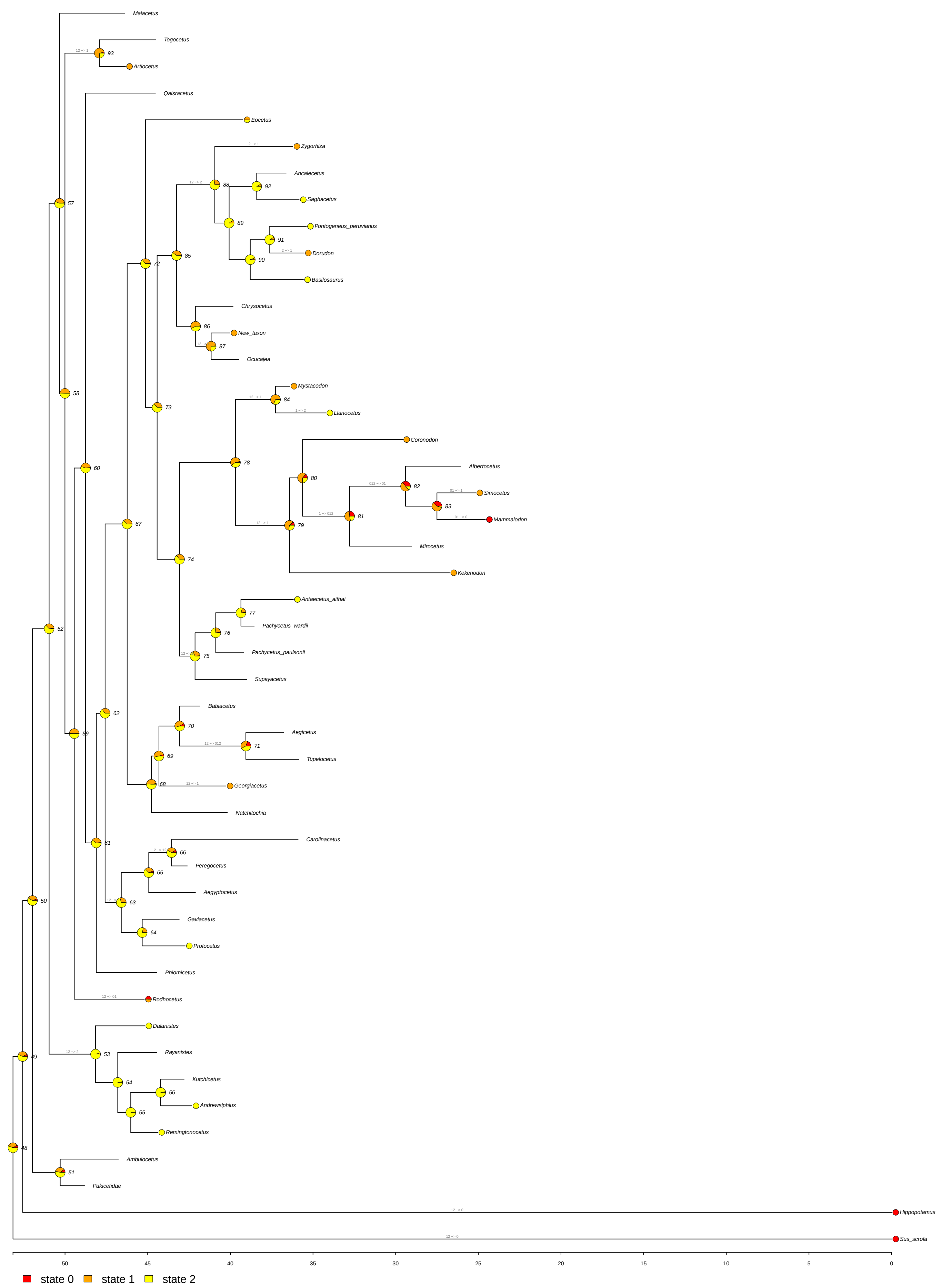
Maiacetus
Togocetus
Artiocetus
Qaisracetus
Eocetus
Zygorhiza
Ancalecetus
Saghacetus
Pontogeneus_peruvianus
Dorudon
Basilosaurus
Chrysocetus
New_taxon
Ocucajea
Mystacodon
Llanocetus
Coronodon
Albertocetus
Simocetus
Mammalodon
Mirocetus
Kekenodon
Antaecetus_aithai
Pachycetus_wardii
Pachycetus_paulsonii
Supayacetus
Babiacetus
Aegicetus
Tupelocetus
Georgiacetus
Natchitochia
Carolinacetus
Peregocetus
Aegyptocetus
Gaviacetus
Protocetus
Phiomicetus
Rodhocetus
Dalanistes
Rayanistes
Kutchicetus
Andrewsiphius
Remingtonocetus
Ambulocetus
Pakicetidae
Hippopotamus
Sus_scrofa
50 45 40 35 30 25 20 15 10 5 0
state 0
state 1
state 2

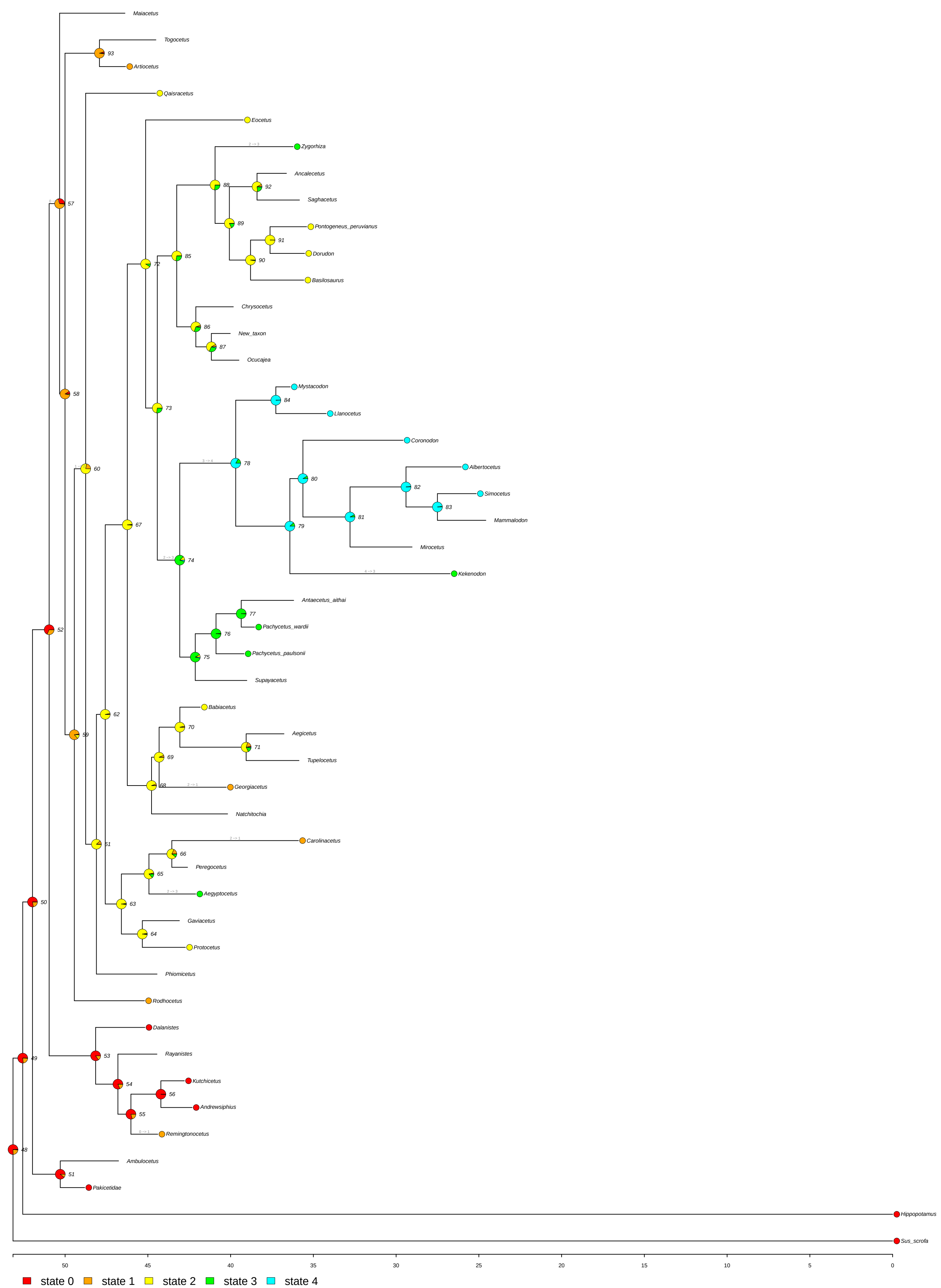

Maiacetus
Togocetus
93
Artiocetus
Qaisracetus
Eocetus
2 -> 3
Zygorhiza
Ancalecetus
88
92
Saghacetus
57
89
Pontogeneus_peruvianus
91
Dorudon
90
Basilosaurus
72
85
Chrysocetus
86
New_taxon
87
Ocucajea
Mystacodon
84
58
Llanocetus
73
Coronodon
3 -> 4
78
Albertocetus
60
80
82
Simocetus
67
83
81
Mammalodon
79
Mirocetus
2 -> 3
74
4 -> 3
Kekenodon
Antaecetus_aithai
77
52
Pachycetus_wardii
76
Pachycetus_paulsonii
75
Supayacetus
Babiacetus
62
70
59
Aegicetus
71
69
Tupelocetus
68
2 -> 1
Georgiacetus
Natchitochia
2 -> 1
Carolinacetus
61
66
Peregocetus
65
2 -> 3
Aegyptocetus
50
63
Gaviacetus
64
Protocetus
Phiomicetus
Rodhocetus
Dalanistes
Rayanistes
49
53
54
Kutchicetus
56
55
Andrewsiphius
0 -> 1
Remingtonocetus
48
Ambulocetus
51
Pakicetidae
Hippopotamus
Sus_scrofa
50
45
40
35
30
25
20
15
10
5
0
state 0
state 1
state 2
state 3
state 4

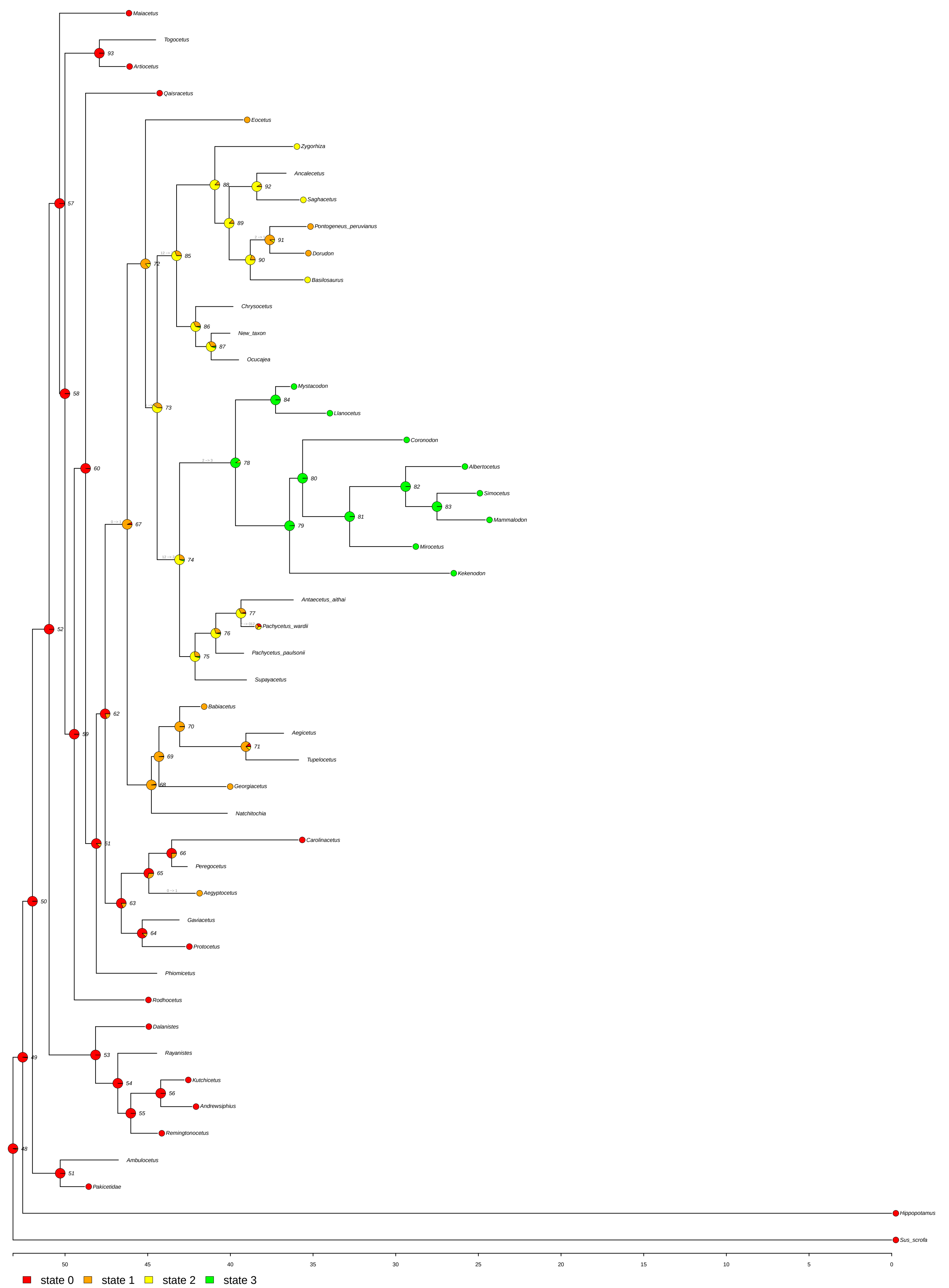

Maiacetus
Togocetus
93
Artiocetus
Qaisracetus
Eocetus
Zygorhiza
Ancalecetus
88
92
Saghacetus
89
Pontogeneus_peruvianus
91
Dorudon
90
Basilosaurus
72
85
Chrysocetus
86
New_taxon
87
Ocucajea
57
58
Mystacodon
84
Llanocetus
73
Coronodon
60
2 --> 3
78
Albertocetus
80
82
Simocetus
83
Mammalodon
81
79
Mirocetus
67
12 --> 2
74
Kekenodon
Antaecetus_aithai
77
Pachycetus_wardii
76
Pachycetus_paulsonii
75
Supayacetus
52
Babiacetus
62
70
Aegicetus
71
59
Tupelocetus
69
68
Georgiacetus
Natchitochia
Carolinacetus
61
66
Peregocetus
65
0 --> 1
Aegyptocetus
50
63
Gaviacetus
64
Protocetus
Phiomicetus
Rodhocetus
Dalanistes
53
Rayanistes
49
54
Kutchicetus
56
Andrewsiphius
55
Remingtonocetus
48
Ambulocetus
51
Pakicetidae
Hippopotamus
Sus_scrofa
50
45
40
35
30
25
20
15
10
5
0
state 0
state 1
state 2
state 3

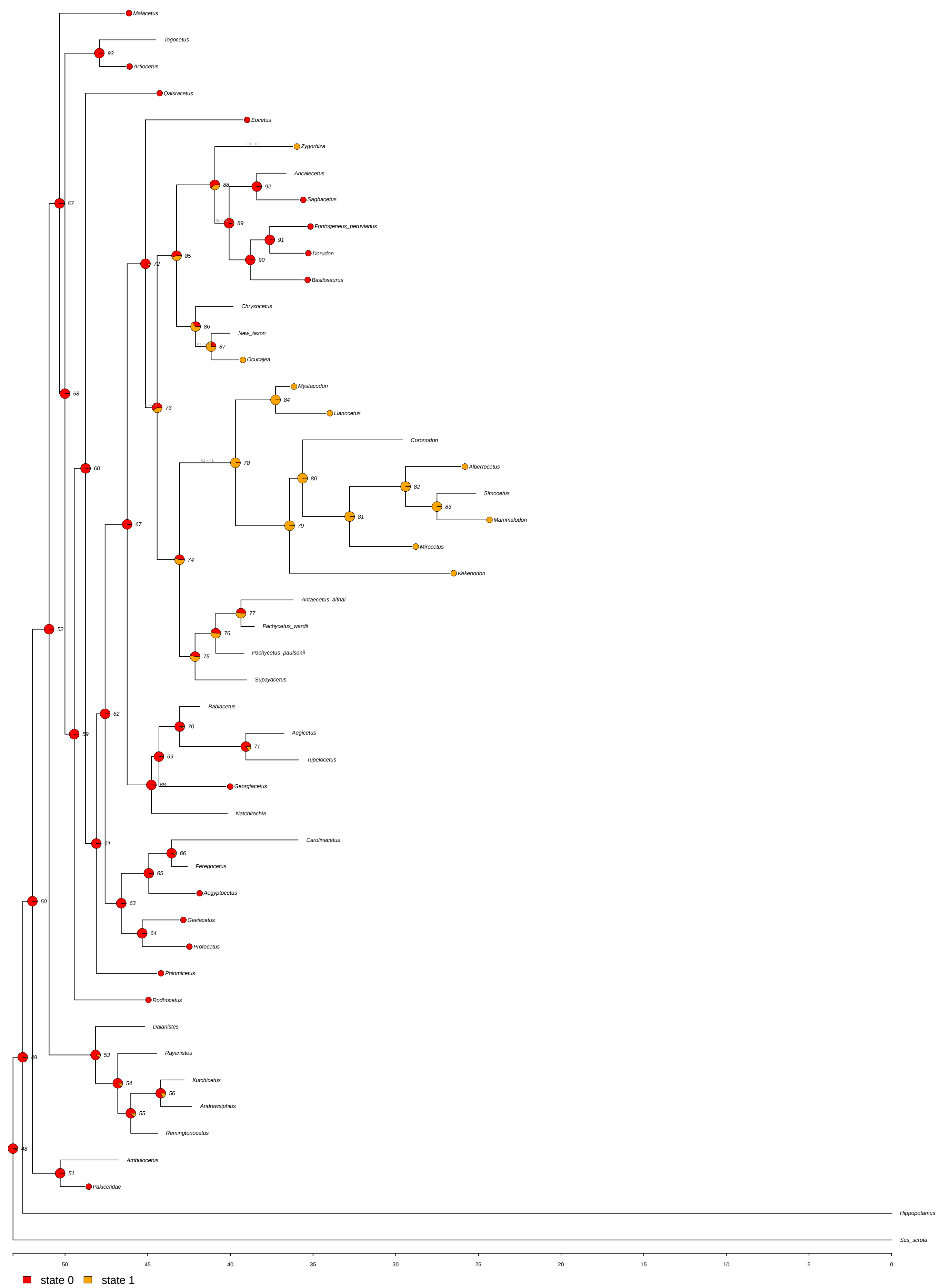
Maiacetus
Togocetus
Artiocetus
Qaisracetus
Eocetus
Zygorhiza
Ancalecetus
Saghacetus
Pontogeneus_peruvianus
Dorudon
Basilosaurus
Chrysocetus
New_taxon
Ocucajea
Mystacodon
Llanocetus
Coronodon
Albertocetus
Simocetus
Mammalodon
Mirocetus
Kekenodon
Antaecetus_aithai
Pachycetus_wardii
Pachycetus_paulsonii
Supayacetus
Babiacetus
Aegicetus
Tupelocetus
Georgiacetus
Natchitochia
Carolinacetus
Peregocetus
Aegyptocetus
Gaviacetus
Protocetus
Phiomicetus
Rodhocetus
Dalanistes
Rayanistes
Kutchicetus
Andrewsiphius
Remingtonocetus
Ambulocetus
Pakicetidae
Hippopotamus
Sus_scrofa
50
45
40
35
30
25
20
15
10
5
0
state 0
state 1

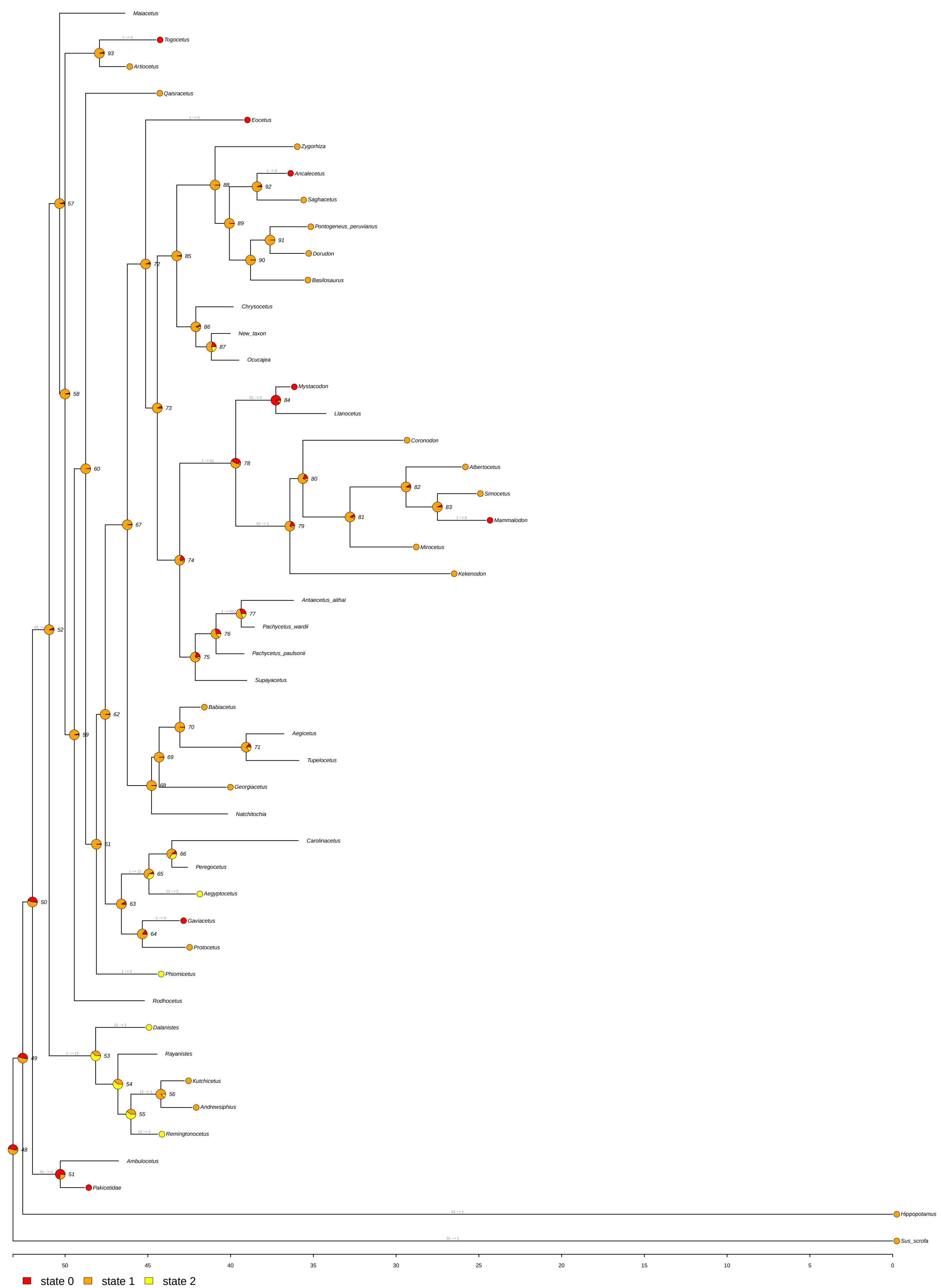

Maiacetus
Togocetus
93
Artiocetus
Qaisracetus
Eocetus
Zygorhiza
88
Ancalecetus
92
Saghacetus
89
Pontogeneus_peruvianus
91
Dorudon
90
Basilosaurus
57
72
85
Chrysocetus
86
New_taxon
87
Ocucajea
Mystacodon
84
Llanocetus
58
73
Coronodon
78
Albertocetus
80
60
82
Simocetus
83
Mammalodon
81
67
79
Mirocetus
74
Kekenodon
Antaecetus_aithai
77
Pachycetus_wardii
52
76
Pachycetus_paulsonii
75
Supayacetus
Babiacetus
62
70
Aegicetus
59
71
Tupelocetus
69
68
Georgiacetus
Natchitochia
Carolinacetus
61
66
Peregocetus
65
Aegyptocetus
50
63
Gaviacetus
64
Protocetus
Phiomicetus
Rodhocetus
Dalanistes
Rayanistes
53
49
54
Kutchicetus
56
55
Andrewsiphius
Remingtonocetus
48
Ambulocetus
51
Pakicetidae
Hippopotamus
Sus_scrofa
50
45
40
35
30
25
20
15
10
5
0
state 0
state 1
state 2

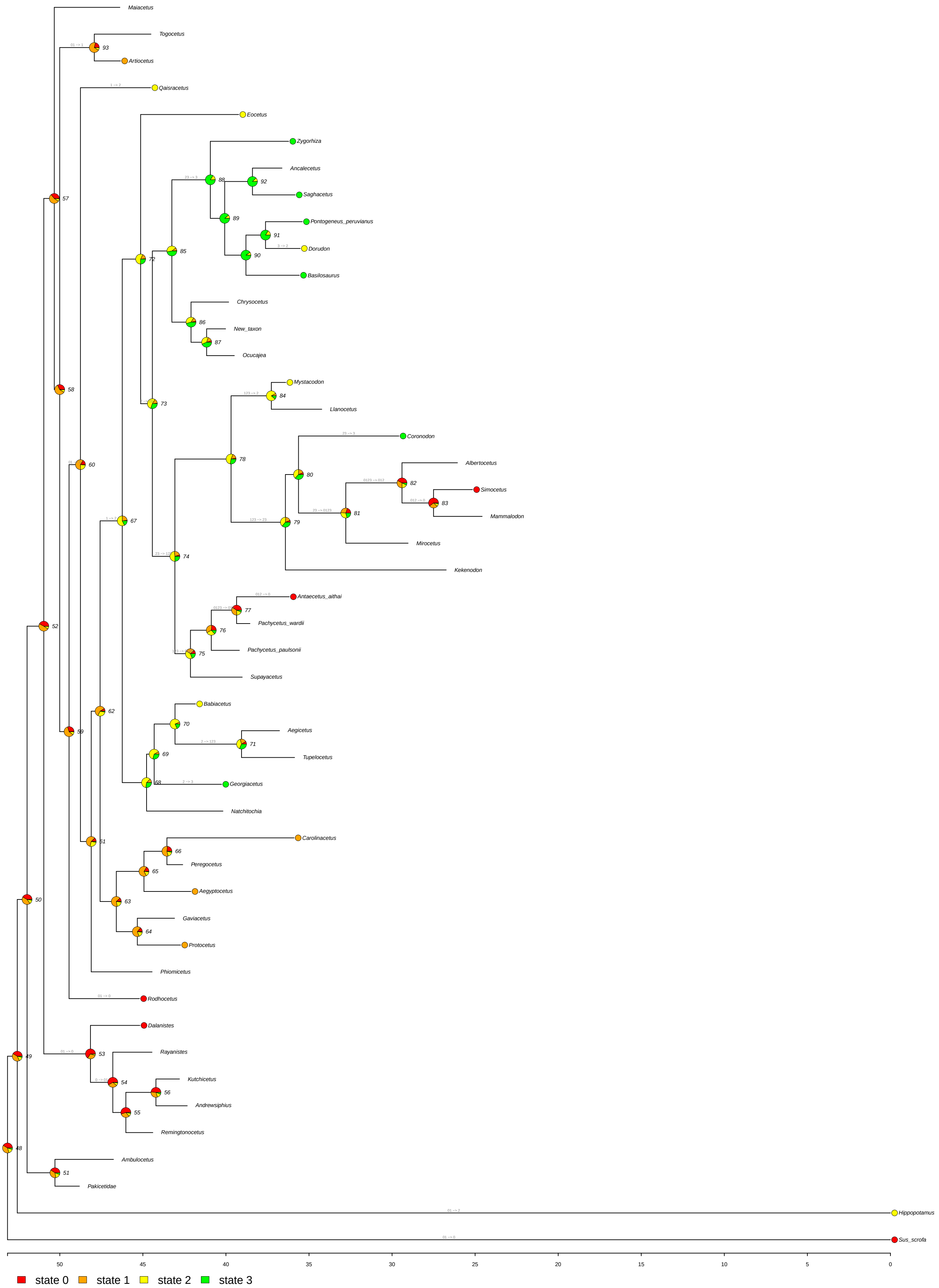
Maiacetus
Togocetus
Artiocetus
Qaisracetus
Eocetus
Zygorhiza
Ancalecetus
Saghacetus
Pontogeneus_peruvianus
Dorudon
Basilosaurus
Chrysocetus
New_taxon
Ocucajea
Mystacodon
Llanocetus
Coronodon
Albertocetus
Simocetus
Mammalodon
Mirocetus
Kekenodon
Antaecetus_aithai
Pachycetus_wardii
Pachycetus_paulsonii
Supayacetus
Babiacetus
Aegicetus
Tupelocetus
Georgiacetus
Natchitochia
Carolinacetus
Peregocetus
Aegyptocetus
Gaviacetus
Protocetus
Phiomicetus
Rodhocetus
Dalanistes
Rayanistes
Kutchicetus
Andrewsiphius
Remingtonocetus
Ambulocetus
Pakicetidae
Hippopotamus
Sus_scrofa
50
45
40
35
30
25
20
15
10
5
0
state 0
state 1
state 2
state 3

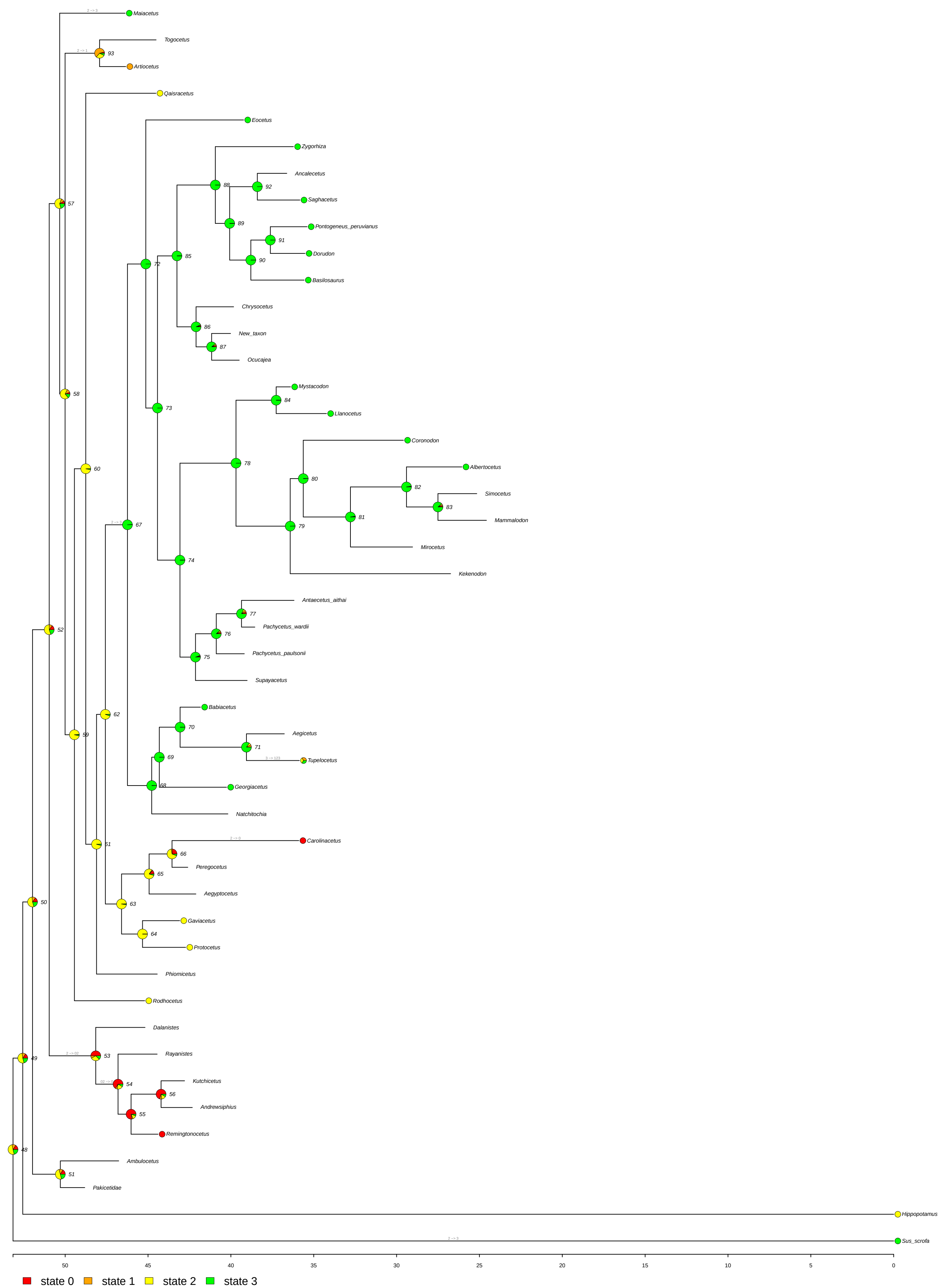

Maiacetus
Togocetus
Artiocetus
Qaisracetus
Eocetus
Zygorhiza
Ancalecetus
Saghacetus
Pontogeneus_peruvianus
Dorudon
Basilosaurus
Chrysocetus
New_taxon
Ocucajea
Mystacodon
Llanocetus
Coronodon
Albertocetus
Simocetus
Mammalodon
Mirocetus
Kekenodon
Antaecetus_aithai
Pachycetus_wardii
Pachycetus_paulsonii
Supayacetus
Babiacetus
Aegicetus
Tupelocetus
Georgiacetus
Natchitochia
Carolinacetus
Peregocetus
Aegyptocetus
Gaviacetus
Protocetus
Phiomicetus
Rodhocetus
Dalanistes
Rayanistes
Kutchicetus
Andrewsiphius
Remingtonocetus
Ambulocetus
Pakicetidae
Hippopotamus
Sus_scrofa
50 45 40 35 30 25 20 15 10 5 0
state 0
state 1
state 2
state 3

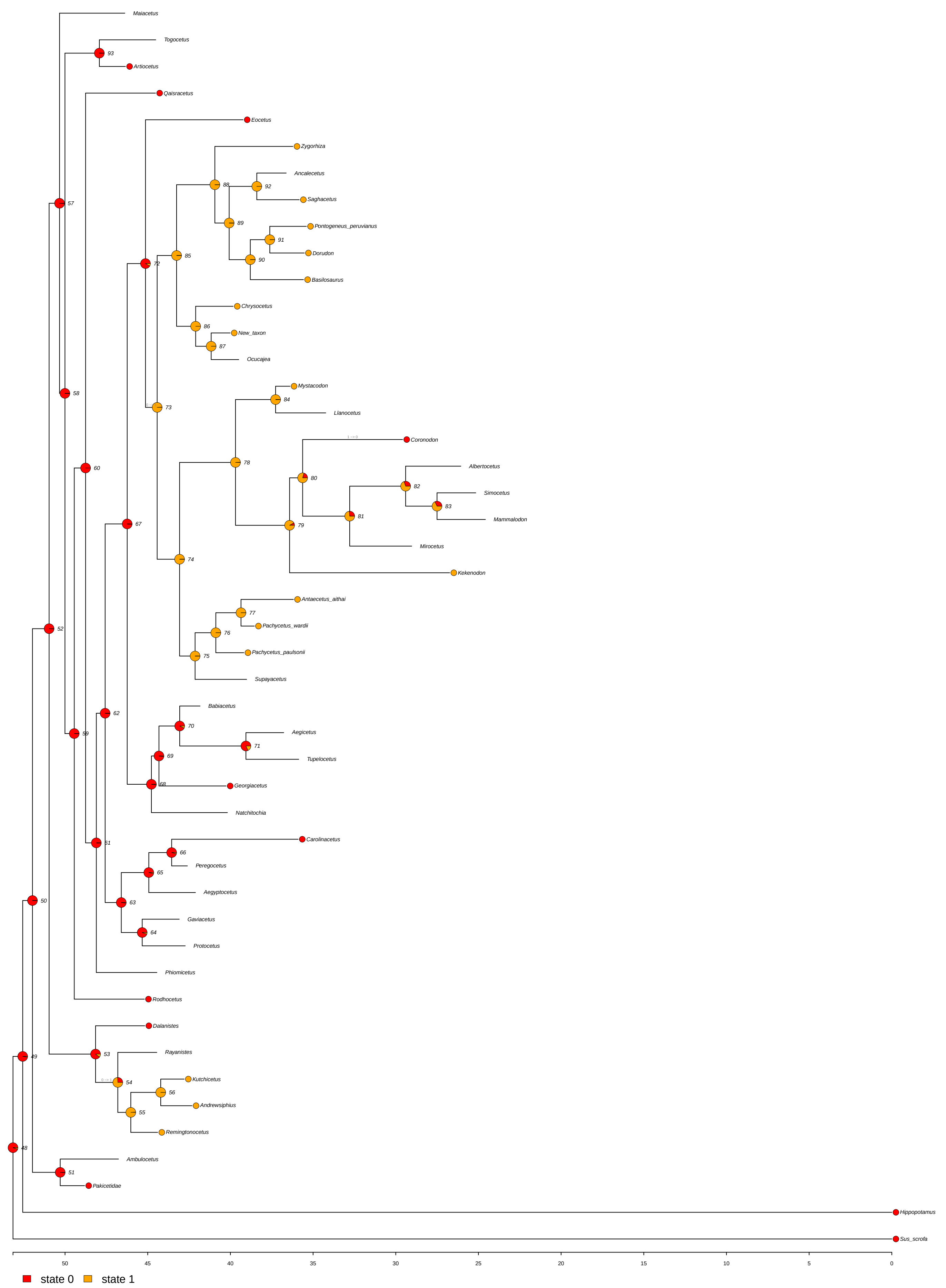

Maiacetus
Togocetus
Artiocetus
Qaisracetus
Eocetus
Zygorhiza
Ancalecetus
Saghacetus
Pontogeneus_peruvianus
Dorudon
Basilosaurus
Chrysocetus
New_taxon
Ocucajea
Mystacodon
Llanocetus
Coronodon
Albertocetus
Simocetus
Mammalodon
Mirocetus
Kekenodon
Antaecetus_aithai
Pachycetus_wardii
Pachycetus_paulsonii
Supayacetus
Babiacetus
Aegicetus
Tupelocetus
Georgiacetus
Natchitochia
Carolinacetus
Peregocetus
Aegyptocetus
Gaviacetus
Protocetus
Phiomicetus
Rodhocetus
Dalanistes
Rayanistes
Kutchicetus
Andrewsiphius
Remingtonocetus
Ambulocetus
Pakicetidae
Hippopotamus
Sus_scrofa
50
45
40
35
30
25
20
15
10
5
0
state 0
state 1

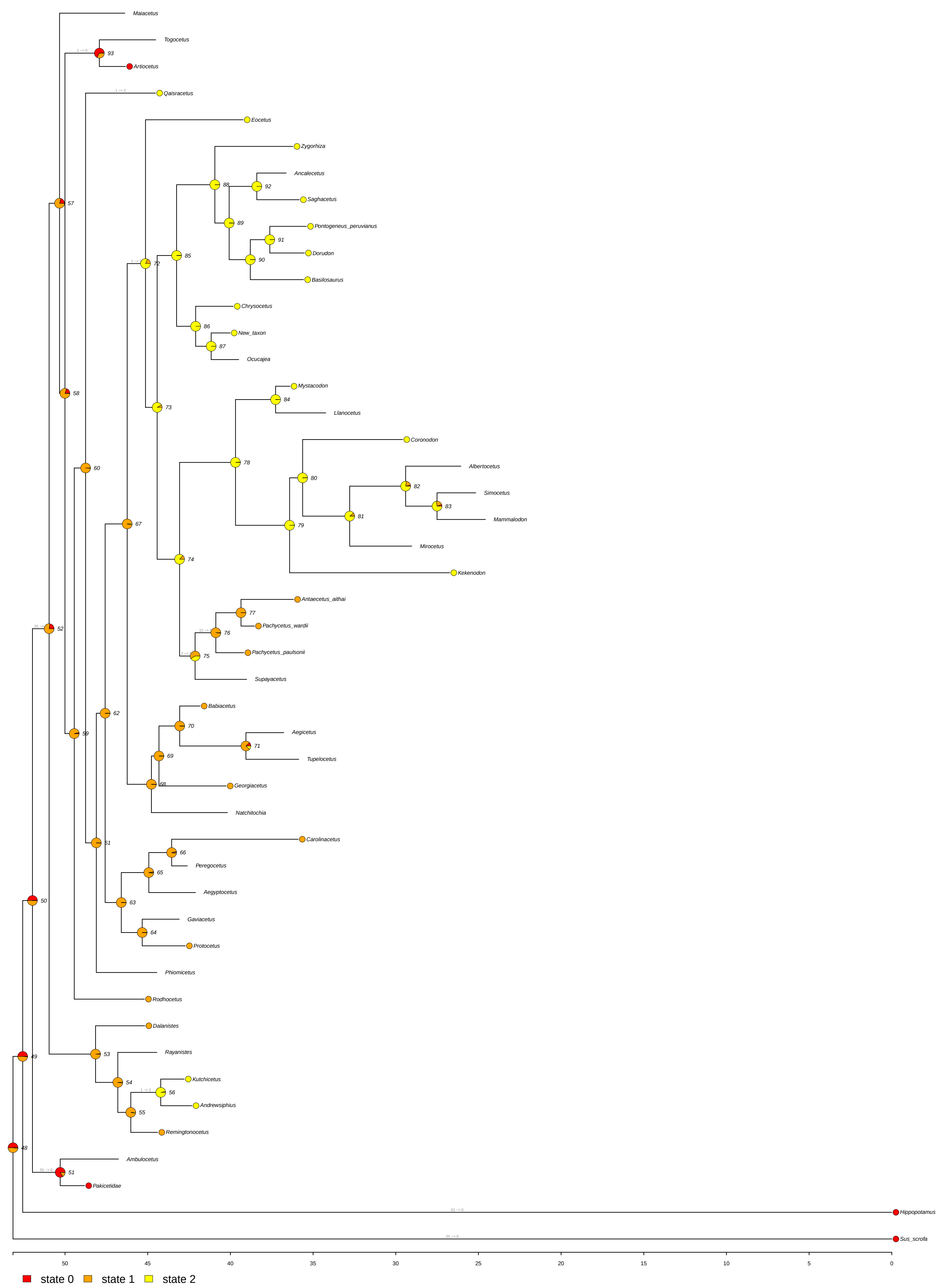

Maiacetus
Togocetus
93
Artiocetus
Qaisracetus
Eocetus
Zygorhiza
Ancalecetus
88
92
Saghacetus
89
Pontogeneus_peruvianus
91
Dorudon
90
Basilosaurus
72
85
Chrysocetus
86
New_taxon
87
Ocucajea
Mystacodon
84
Llanocetus
57
58
73
Coronodon
60
78
Albertocetus
80
82
Simocetus
83
Mammalodon
67
81
79
Mirocetus
74
Kekenodon
Antaecetus_aithai
77
Pachycetus_wardii
76
52
Pachycetus_paulsonii
75
Supayacetus
Babiacetus
62
70
Aegicetus
59
71
Tupelocetus
69
68
Georgiacetus
Natchitochia
Carolinacetus
66
Peregocetus
61
65
Aegyptocetus
50
63
Gaviacetus
64
Protocetus
Phiomicetus
Rodhocetus
Dalanistes
Rayanistes
53
49
54
Kutchicetus
56
55
Andrewsiphius
Remingtonocetus
48
Ambulocetus
51
Pakicetidae
Hippopotamus
Sus_scrofa
50
45
40
35
30
25
20
15
10
5
0
state 0
state 1
state 2

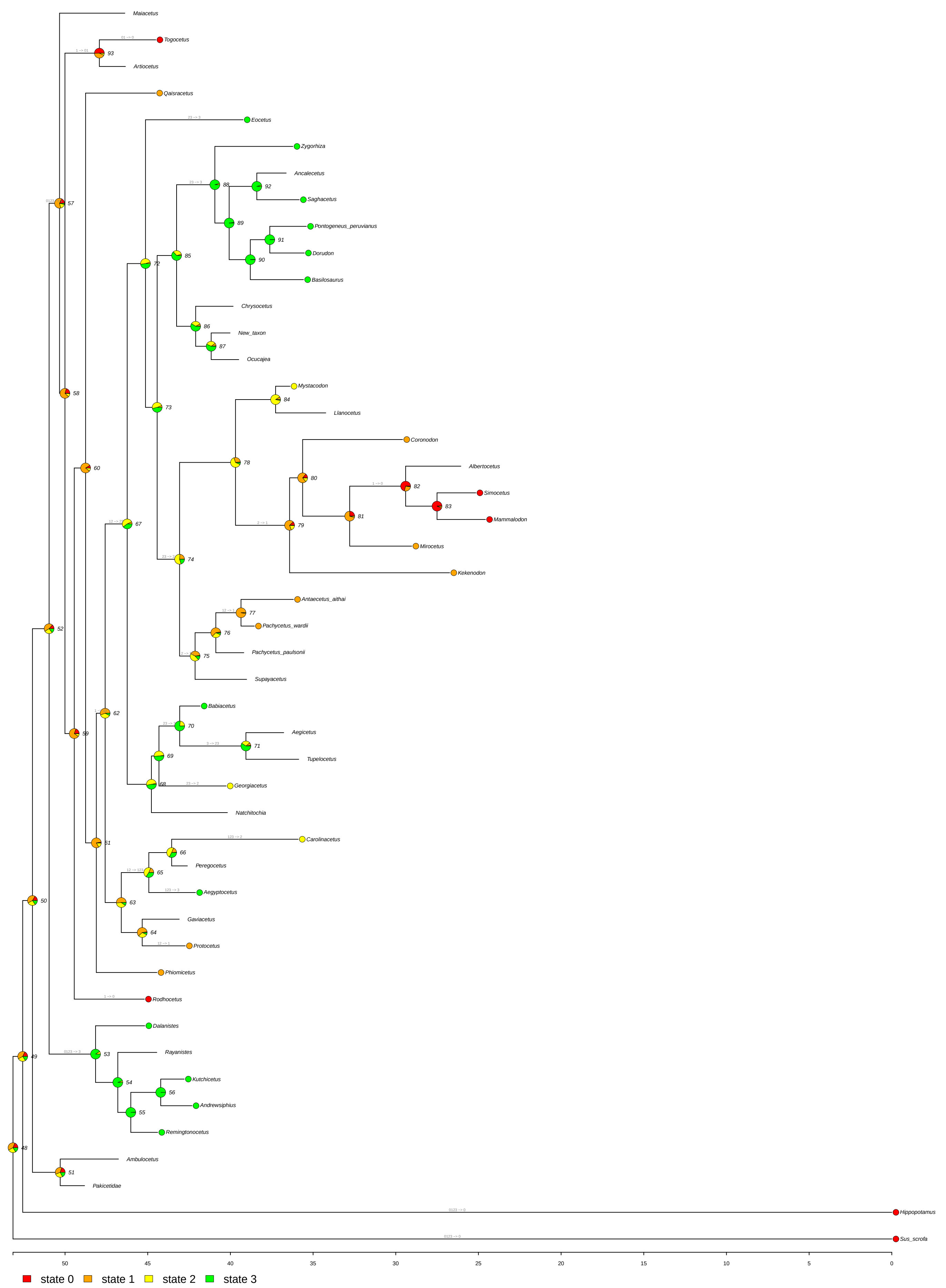

Maiacetus
Togocetus
Artiocetus
Qaisracetus
Eocetus
Zygorhiza
Ancalecetus
Saghacetus
Pontogeneus_peruvianus
Dorudon
Basilosaurus
Chrysocetus
New_taxon
Ocucajea
Mystacodon
Llanocetus
Coronodon
Albertocetus
Simocetus
Mammalodon
Mirocetus
Kekenodon
Antaecetus_aithai
Pachycetus_wardii
Pachycetus_paulsonii
Supayacetus
Babiacetus
Aegicetus
Tupelocetus
Georgiacetus
Natchitochia
Carolinacetus
Peregocetus
Aegyptocetus
Gaviacetus
Protocetus
Phiomicetus
Rodhocetus
Dalanistes
Rayanistes
Kutchicetus
Andrewsiphius
Remingtonocetus
Ambulocetus
Pakicetidae
Hippopotamus
Sus_scrofa
50
45
40
35
30
25
20
15
10
5
0
state 0
state 1
state 2
state 3

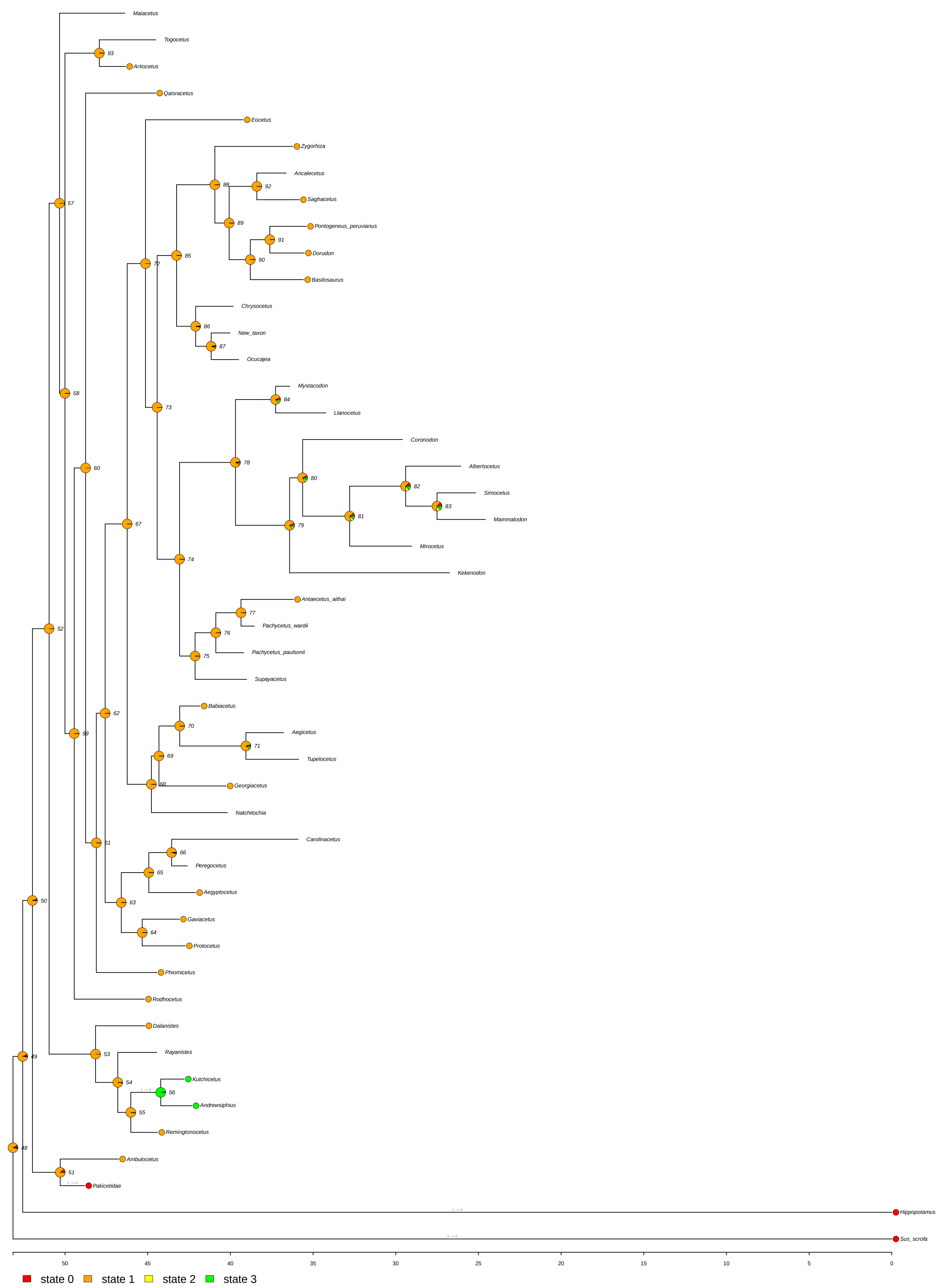
Maiacetus
Togocetus
93
Artiocetus
Qaisracetus
Eocetus
Zygorhiza
Ancalecetus
88
92
Saghacetus
Pontogeneus_peruvianus
89
91
Dorudon
90
Basilosaurus
57
72
85
Chrysocetus
86
New_taxon
87
Ocucajea
Mystacodon
84
Llanocetus
58
73
Coronodon
78
80
Albertocetus
82
Simocetus
83
Mammalodon
60
67
81
79
Mirocetus
74
Kekenodon
Antaecetus_aithai
77
Pachycetus_wardii
76
52
Pachycetus_paulsonii
75
Supayacetus
Babiacetus
62
70
Aegicetus
59
71
Tupelocetus
69
68
Georgiacetus
Natchitochia
Carolinacetus
61
66
Peregocetus
65
50
Aegyptocetus
63
Gaviacetus
64
Protocetus
Phiomicetus
Rodhocetus
Dalanistes
Rayanistes
53
49
54
Kutchicetus
56
Andrewsiphius
55
Remingtonocetus
48
Ambulocetus
51
Pakicetidae
Hippopotamus
Sus_scrofa
50
45
40
35
30
25
20
15
10
5
0
state 0
state 1
state 2
state 3

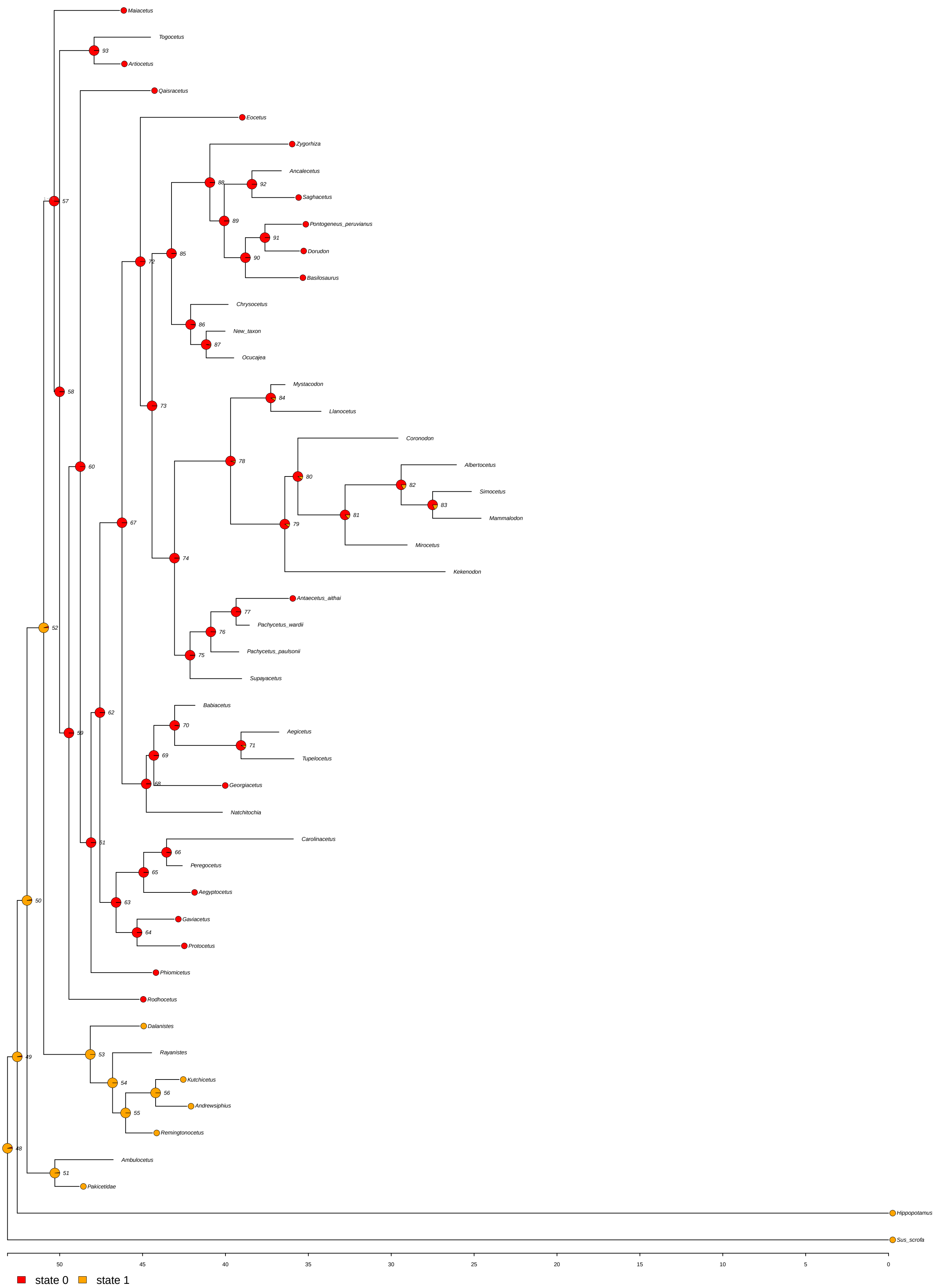

Maiacetus
Togocetus
93
Artiocetus
Qaisracetus
Eocetus
Zygorhiza
Ancalecetus
88
92
Saghacetus
57
Pontogeneus_peruvianus
89
91
Dorudon
72
85
90
Basilosaurus
Chrysocetus
86
New_taxon
87
Ocucajea
Mystacodon
84
58
Llanocetus
73
Coronodon
60
78
Albertocetus
80
82
Simocetus
83
67
81
Mammalodon
79
Mirocetus
74
Kekenodon
Antaecetus_aithai
77
Pachycetus_wardii
76
Pachycetus_paulsonii
52
75
Supayacetus
Babiacetus
62
70
59
Aegicetus
71
69
Tupelocetus
68
Georgiacetus
Natchitochia
Carolinacetus
61
66
Peregocetus
65
Aegyptocetus
50
63
Gaviacetus
64
Protocetus
Phiomicetus
Rodhocetus
Dalanistes
53
Rayanistes
49
54
Kutchicetus
56
55
Andrewsiphius
Remingtonocetus
48
Ambulocetus
51
Pakicetidae
Hippopotamus
Sus_scrofa
50
45
40
35
30
25
20
15
10
5
0
state 0
state 1

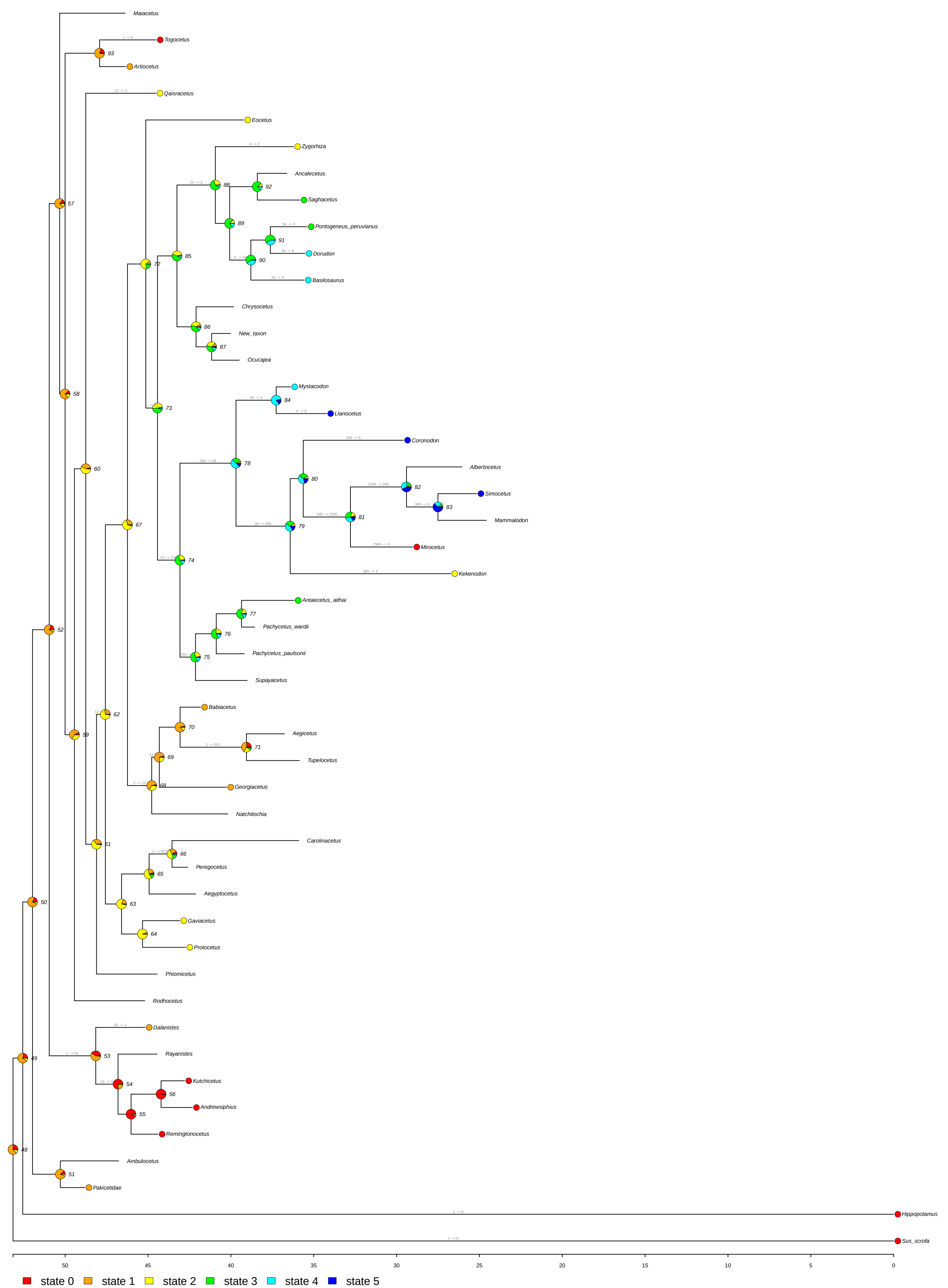

Maiacetus
Togocetus
Artiocetus
Qaisracetus
Eocetus
Zygorhiza
Ancalecetus
Saghacetus
Pontogeneus_peruvianus
Dorudon
Basilosaurus
Chrysocetus
New_taxon
Ocucajea
Mystacodon
Llanocetus
Coronodon
Albertocetus
Simocetus
Mammalodon
Mirocetus
Kekenodon
Antaecetus_aithai
Pachycetus_wardii
Pachycetus_paulsonii
Supayacetus
Babiacetus
Aegicetus
Tupelocetus
Georgiacetus
Natchitochia
Carolinacetus
Peregocetus
Aegyptocetus
Gaviacetus
Protocetus
Phiomicetus
Rodhocetus
Dalanistes
Rayanistes
Kutchicetus
Andrewsiphius
Remingtonocetus
Ambulocetus
Pakicetidae
Hippopotamus
Sus_scrofa
50
45
40
35
30
25
20
15
10
5
0
state 0
state 1
state 2
state 3
state 4
state 5

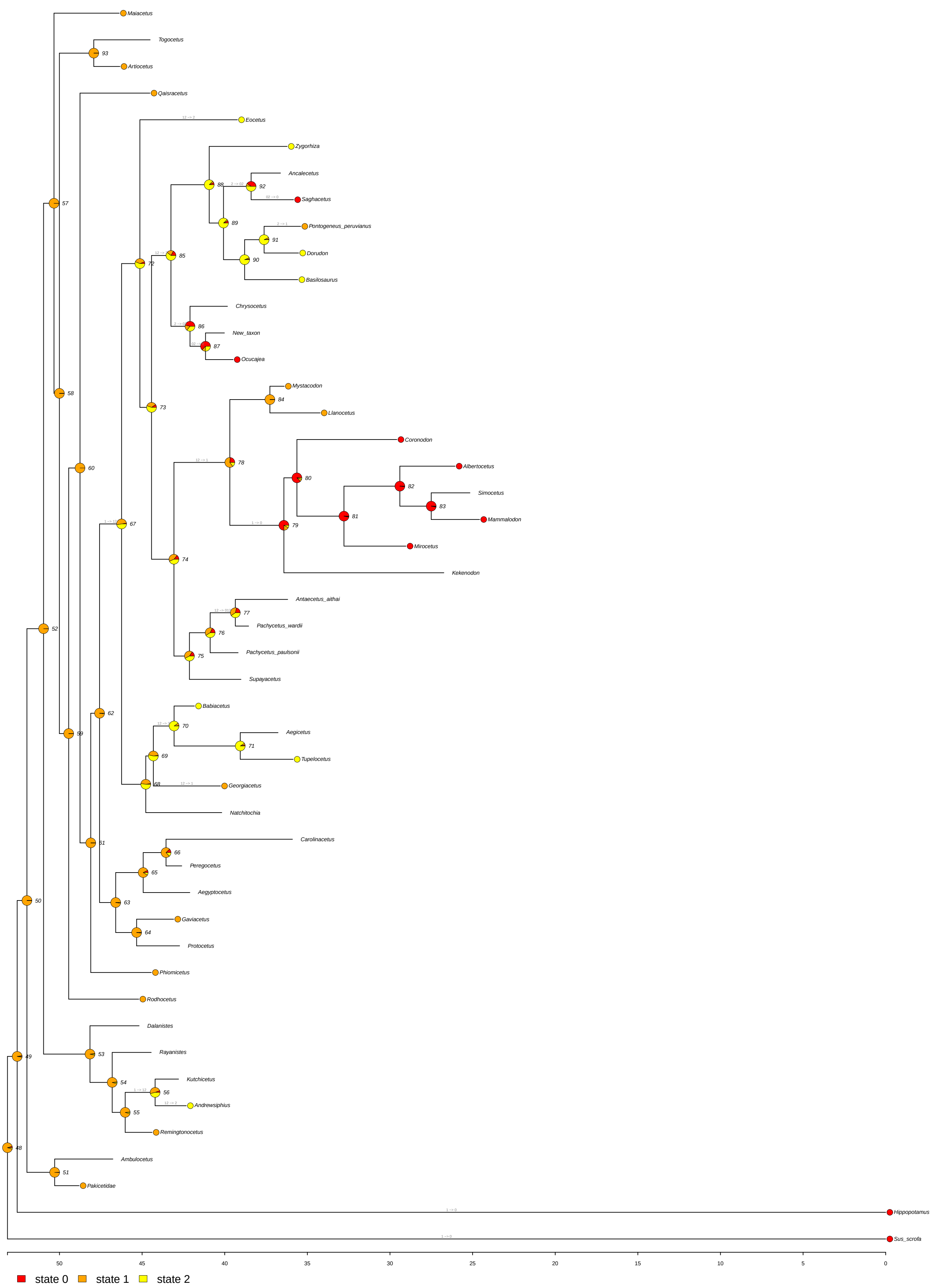

Maiacetus
Togocetus
Artiocetus
Qaisracetus
Eocetus
Zygorhiza
Ancalecetus
Saghacetus
Pontogeneus_peruvianus
Dorudon
Basilosaurus
Chrysocetus
New_taxon
Ocucajea
Mystacodon
Llanocetus
Coronodon
Albertocetus
Simocetus
Mammalodon
Mirocetus
Kekenodon
Antaecetus_aithai
Pachycetus_wardii
Pachycetus_paulsonii
Supayacetus
Babiacetus
Aegicetus
Tupelocetus
Georgiacetus
Natchitochia
Carolinacetus
Peregocetus
Aegyptocetus
Gaviacetus
Protocetus
Phiomicetus
Rodhocetus
Dalanistes
Rayanistes
Kutchicetus
Andrewsiphius
Remingtonocetus
Ambulocetus
Pakicetidae
Hippopotamus
Sus_scrofa
50
45
40
35
30
25
20
15
10
5
0
state 0
state 1
state 2

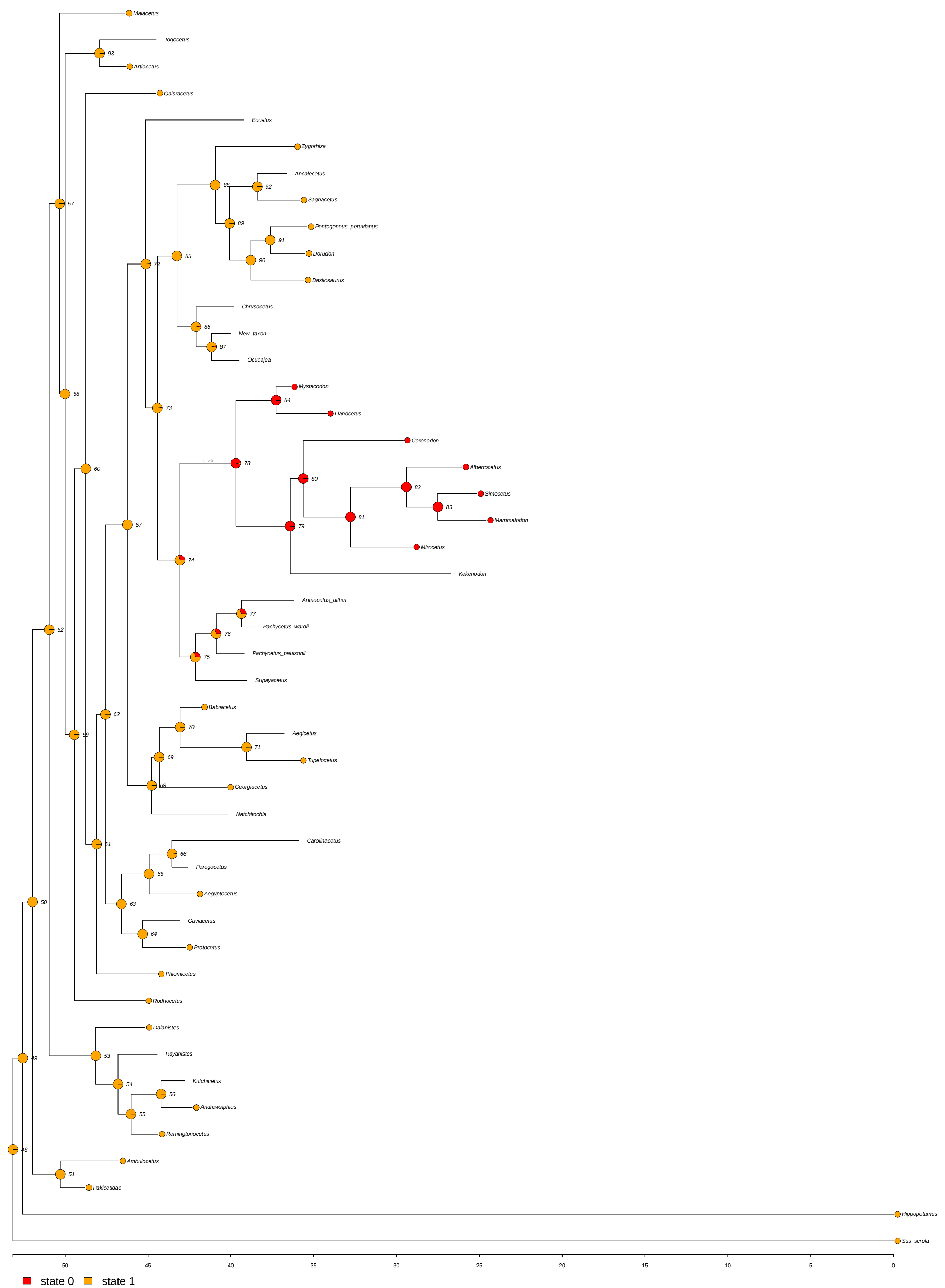

Maiacetus
Togocetus
93
Artiocetus
Qaisracetus
Eocetus
Zygorhiza
Ancalecetus
88
92
Saghacetus
57
Pontogeneus_peruvianus
89
91
Dorudon
85
72
90
Basilosaurus
Chrysocetus
86
New_taxon
87
Ocucajea
Mystacodon
84
58
Llanocetus
73
Coronodon
1 --> 0
78
60
Albertocetus
80
82
Simocetus
83
81
Mammalodon
67
79
Mirocetus
74
Kekenodon
Antaecetus_aithai
77
Pachycetus_wardii
76
52
Pachycetus_paulsonii
75
Supayacetus
Babiacetus
62
70
59
Aegicetus
71
Tupelocetus
69
68
Georgiacetus
Natchitochia
Carolinacetus
61
66
Peregocetus
65
50
Aegyptocetus
63
Gaviacetus
64
Protocetus
Phiomicetus
Rodhocetus
Dalanistes
Rayanistes
53
49
Kutchicetus
54
56
Andrewsiphius
55
Remingtonocetus
48
Ambulocetus
51
Pakicetidae
Hippopotamus
Sus_scrofa
50
45
40
35
30
25
20
15
10
5
0
state 0
state 1

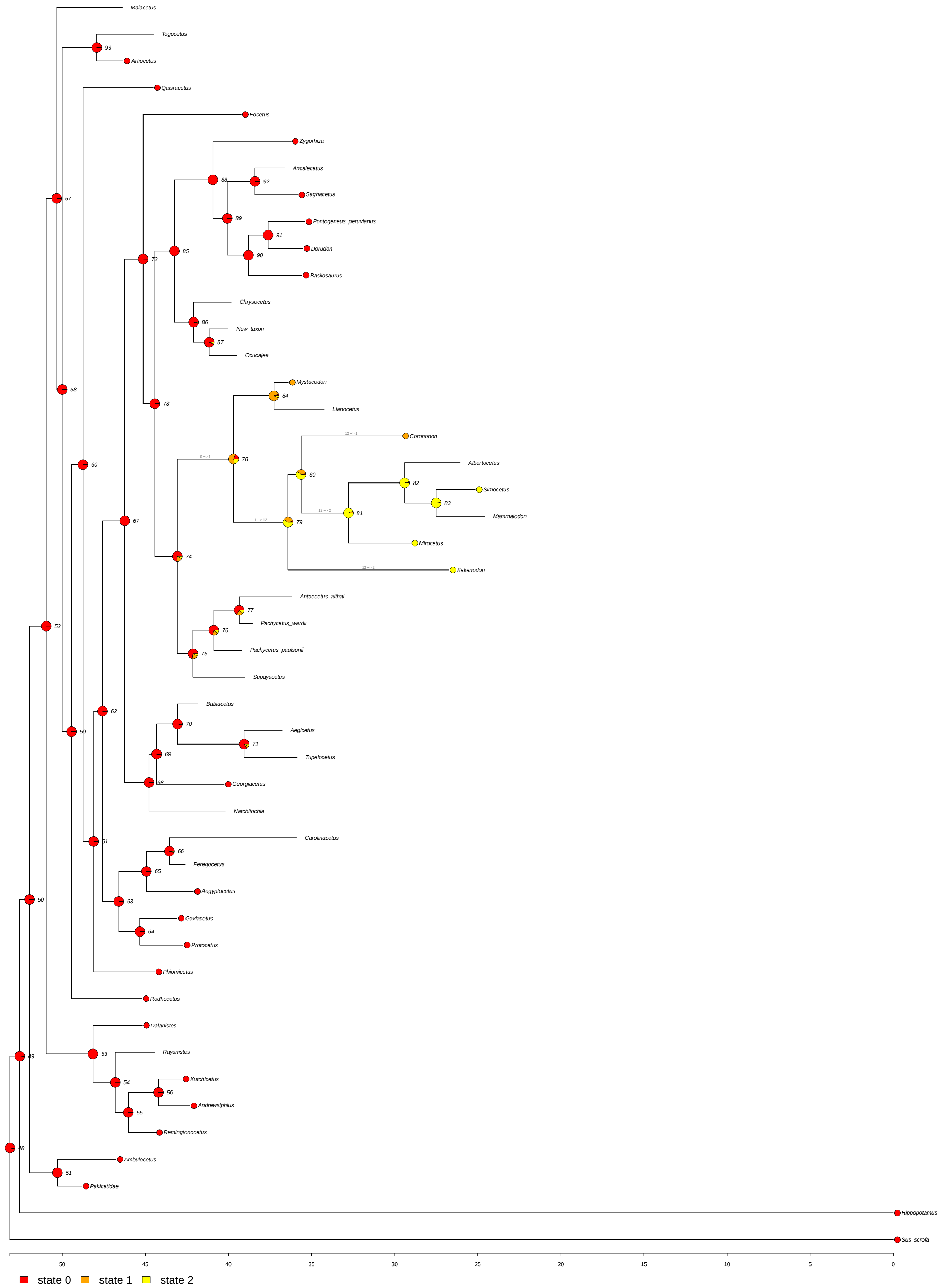

Maiacetus
Togocetus
93
Artiocetus
Qaisracetus
Eocetus
Zygorhiza
Ancalecetus
88
92
Saghacetus
Pontogeneus_peruvianus
89
91
Dorudon
90
Basilosaurus
57
72
85
Chrysocetus
86
New_taxon
87
Ocucajea
Mystacodon
84
Llanocetus
58
73
12 -> 1
Coronodon
0 -> 1
78
60
Albertocetus
80
82
Simocetus
83
12 -> 2
81
Mammalodon
67
1 -> 12
79
Mirocetus
74
12 -> 2
Kekenodon
Antaecetus_aithai
77
Pachycetus_wardii
76
52
Pachycetus_paulsonii
75
Supayacetus
Babiacetus
62
70
59
Aegicetus
71
Tupelocetus
69
68
Georgiacetus
Natchitochia
Carolinacetus
61
66
Peregocetus
65
Aegyptocetus
50
63
Gaviacetus
64
Protocetus
Phiomicetus
Rodhocetus
Dalanistes
Rayanistes
53
49
54
Kutchicetus
56
Andrewsiphius
55
Remingtonocetus
48
Ambulocetus
51
Pakicetidae
Hippopotamus
Sus_scrofa
50
45
40
35
30
25
20
15
10
5
0
state 0
state 1
state 2

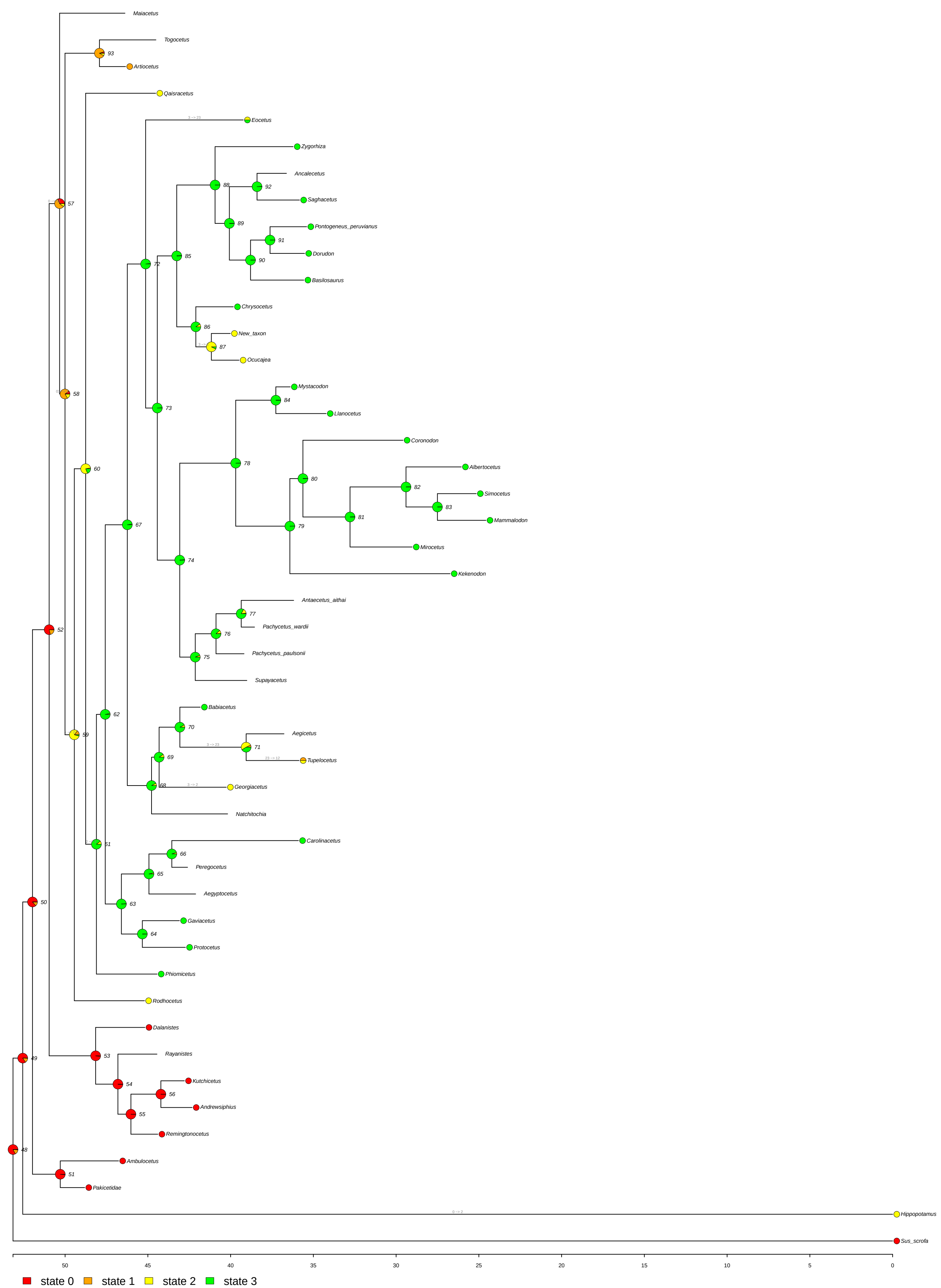
Maiacetus
Togocetus
Artiocetus
Qaisracetus
Eocetus
Zygorhiza
Ancalecetus
Saghacetus
Pontogeneus_peruvianus
Dorudon
Basilosaurus
Chrysocetus
New_taxon
Ocucajea
Mystacodon
Llanocetus
Coronodon
Albertocetus
Simocetus
Mammalodon
Mirocetus
Kekenodon
Antaecetus_aithai
Pachycetus_wardii
Pachycetus_paulsonii
Supayacetus
Babiacetus
Aegicetus
Tupelocetus
Georgiacetus
Natchitochia
Carolinacetus
Peregocetus
Aegyptocetus
Gaviacetus
Protocetus
Phiomicetus
Rodhocetus
Dalanistes
Rayanistes
Kutchicetus
Andrewsiphius
Remingtonocetus
Ambulocetus
Pakicetidae
Hippopotamus
Sus_scrofa
50
45
40
35
30
25
20
15
10
5
0
state 0
state 1
state 2
state 3

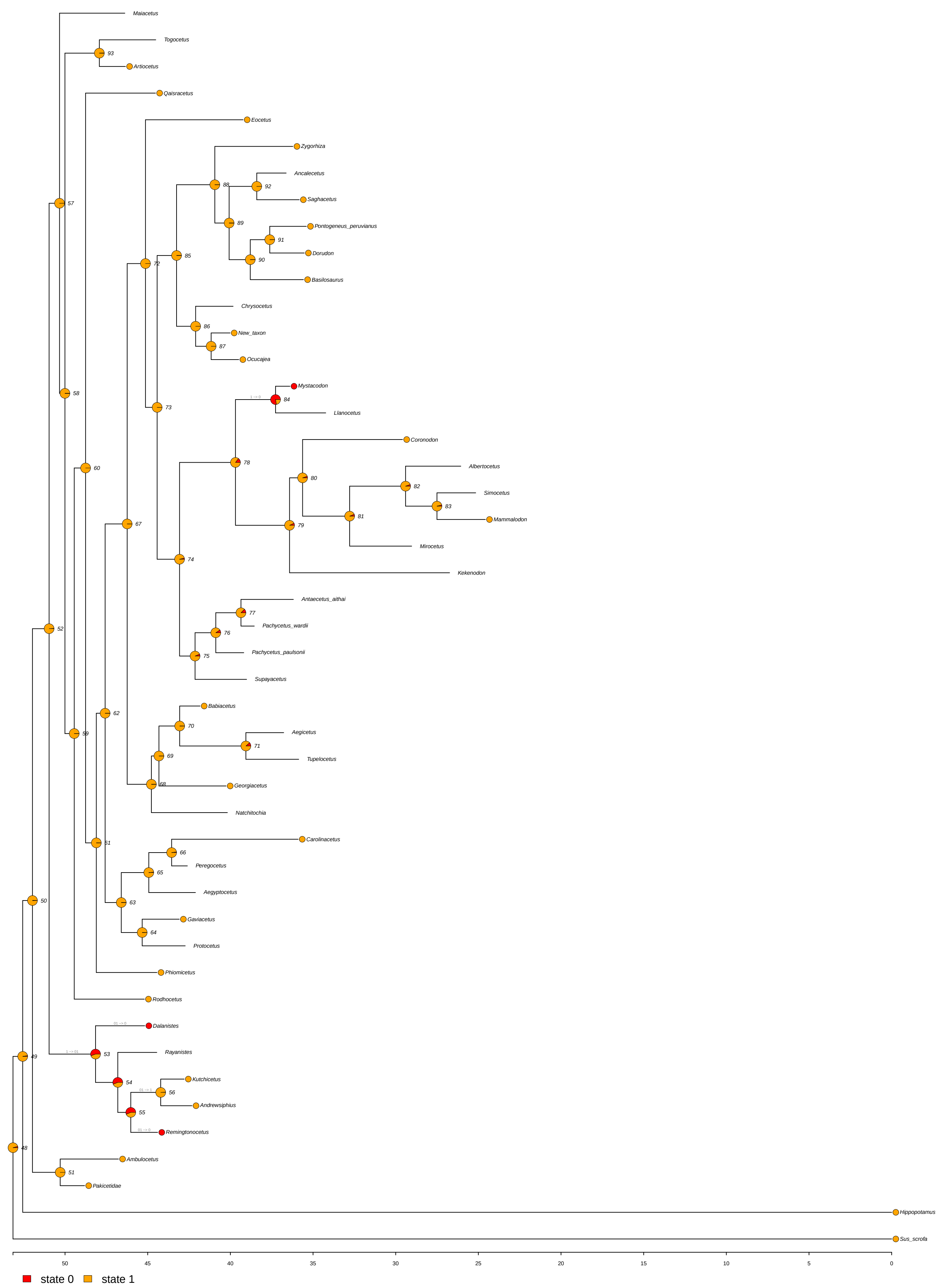

Maiacetus
Togocetus
Artiocetus
Qaisracetus
Eocetus
Zygorhiza
Ancalecetus
Saghacetus
Pontogeneus_peruvianus
Dorudon
Basilosaurus
Chrysocetus
New_taxon
Ocucajea
Mystacodon
Llanocetus
Coronodon
Albertocetus
Simocetus
Mammalodon
Mirocetus
Kekenodon
Antaecetus_aithai
Pachycetus_wardii
Pachycetus_paulsonii
Supayacetus
Babiacetus
Aegicetus
Tupelocetus
Georgiacetus
Natchitochia
Carolinacetus
Peregocetus
Aegyptocetus
Gaviacetus
Protocetus
Phiomicetus
Rodhocetus
Dalanistes
Rayanistes
Kutchicetus
Andrewsiphius
Remingtonocetus
Ambulocetus
Pakicetidae
Hippopotamus
Sus_scrofa
50
45
40
35
30
25
20
15
10
5
0
state 0
state 1

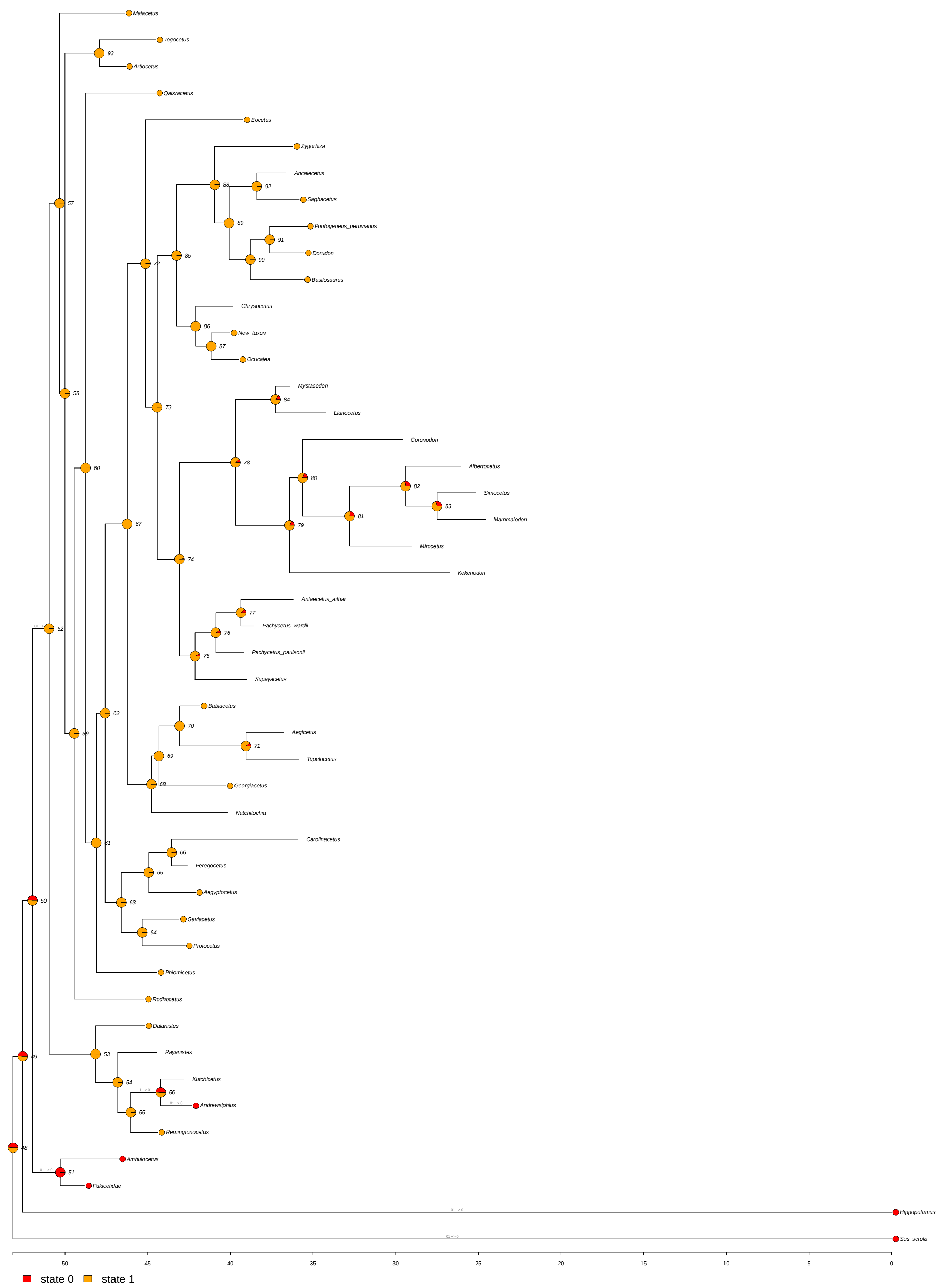
Maiacetus
Togocetus
Artiocetus
Qaisracetus
Eocetus
Zygorhiza
Ancalecetus
Saghacetus
Pontogeneus_peruvianus
Dorudon
Basilosaurus
Chrysocetus
New_taxon
Ocucajea
Mystacodon
Llanocetus
Coronodon
Albertocetus
Simocetus
Mammalodon
Mirocetus
Kekenodon
Antaecetus_aithai
Pachycetus_wardii
Pachycetus_paulsonii
Supayacetus
Babiacetus
Aegicetus
Tupelocetus
Georgiacetus
Natchitochia
Carolinacetus
Peregocetus
Aegyptocetus
Gaviacetus
Protocetus
Phiomicetus
Rodhocetus
Dalanistes
Rayanistes
Kutchicetus
Andrewsiphius
Remingtonocetus
Ambulocetus
Pakicetidae
Hippopotamus
Sus_scrofa
93
88
92
89
91
90
85
72
86
87
57
58
73
84
78
80
82
83
81
79
60
67
74
77
76
75
52
62
70
71
59
69
68
61
66
65
50
63
64
53
49
54
56
55
48
51
50
45
40
35
30
25
20
15
10
5
0
state 0
state 1

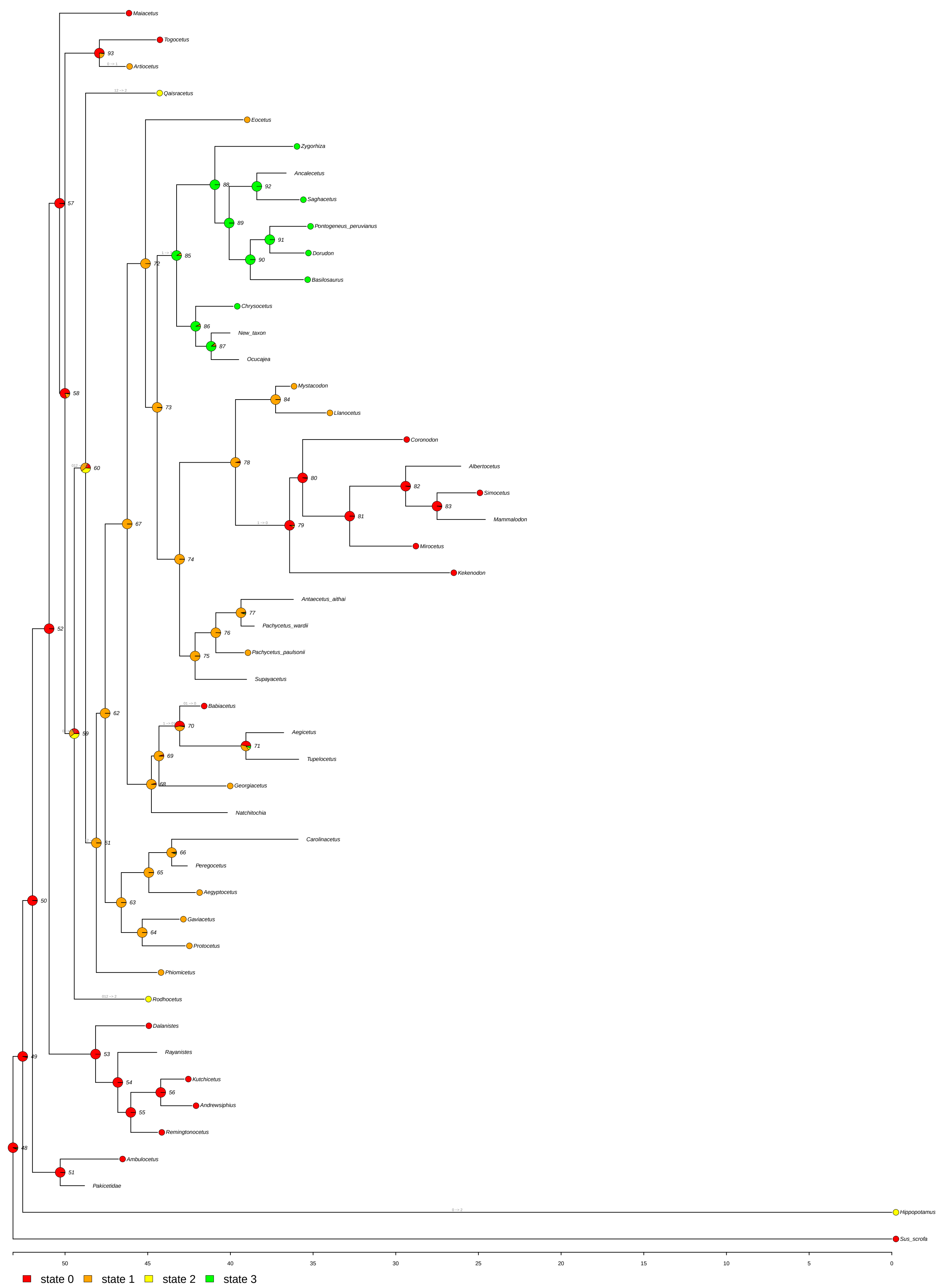

Maiacetus
Togocetus
Artiocetus
Qaisracetus
Eocetus
Zygorhiza
Ancalecetus
Saghacetus
Pontogeneus_peruvianus
Dorudon
Basilosaurus
Chrysocetus
New_taxon
Ocucajea
Mystacodon
Llanocetus
Coronodon
Albertocetus
Simocetus
Mammalodon
Mirocetus
Kekenodon
Antaecetus_aithai
Pachycetus_wardii
Pachycetus_paulsonii
Supayacetus
Babiacetus
Aegicetus
Tupelocetus
Georgiacetus
Natchitochia
Carolinacetus
Peregocetus
Aegyptocetus
Gaviacetus
Protocetus
Phiomicetus
Rodhocetus
Dalanistes
Rayanistes
Kutchicetus
Andrewsiphius
Remingtonocetus
Ambulocetus
Pakicetidae
Hippopotamus
Sus_scrofa
50
45
40
35
30
25
20
15
10
5
0
state 0
state 1
state 2
state 3

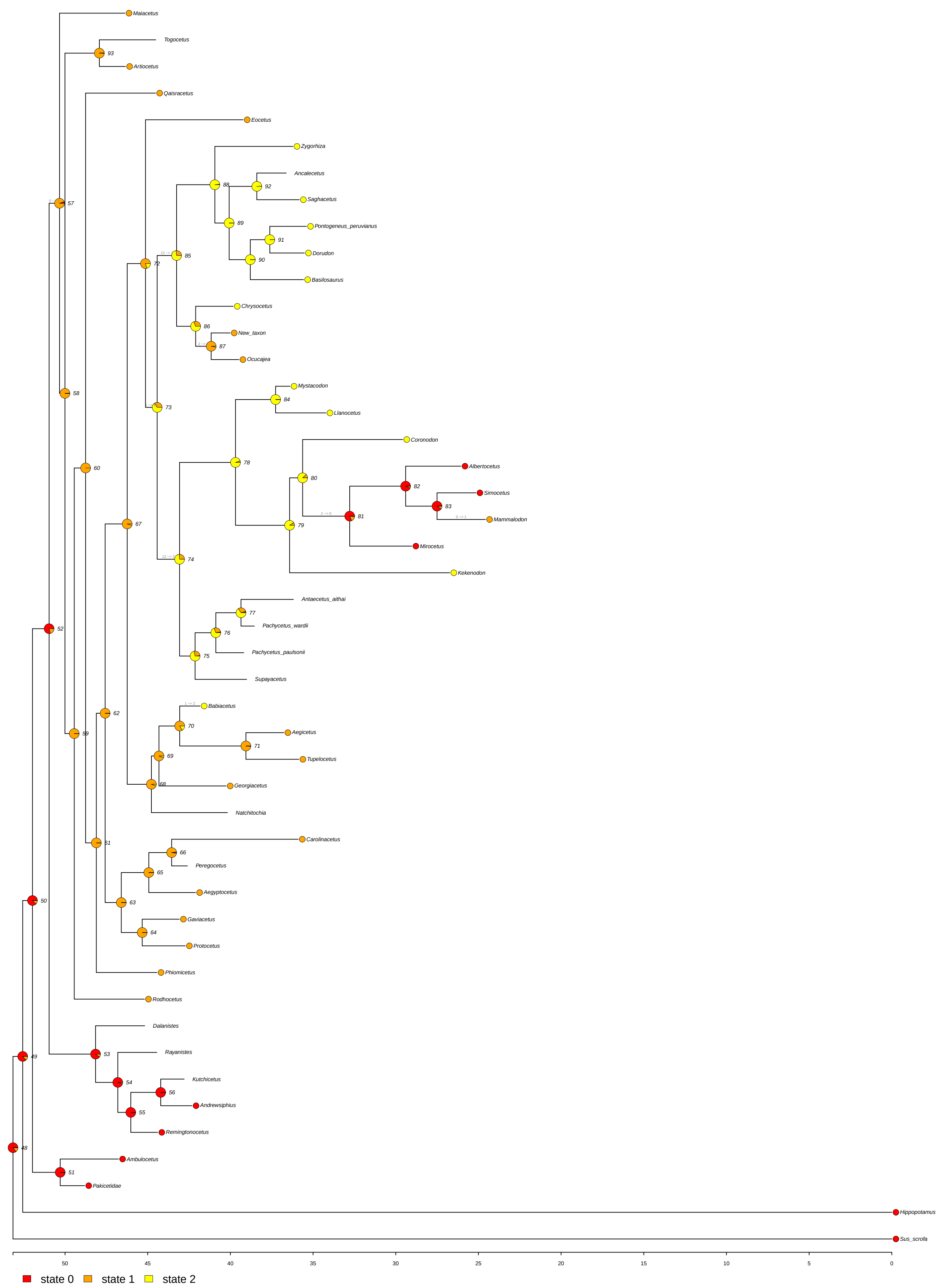

Maiacetus
Togocetus
93
Artiocetus
Qaisracetus
Eocetus
Zygorhiza
Ancalecetus
88
92
Saghacetus
89
Pontogeneus_peruvianus
91
Dorudon
90
Basilosaurus
57
72
85
Chrysocetus
86
New_taxon
87
Ocucajea
Mystacodon
84
Llanocetus
58
73
Coronodon
78
60
80
Albertocetus
82
Simocetus
83
Mammalodon
81
67
79
Mirocetus
74
Kekenodon
Antaecetus_aithai
77
Pachycetus_wardii
52
76
Pachycetus_paulsonii
75
Supayacetus
Babiacetus
62
70
Aegicetus
59
71
Tupelocetus
69
68
Georgiacetus
Natchitochia
Carolinacetus
61
66
Peregocetus
65
Aegyptocetus
50
63
Gaviacetus
64
Protocetus
Phiomicetus
Rodhocetus
Dalanistes
Rayanistes
53
49
54
Kutchicetus
56
Andrewsiphius
55
Remingtonocetus
48
Ambulocetus
51
Pakicetidae
Hippopotamus
Sus_scrofa
50
45
40
35
30
25
20
15
10
5
0
state 0
state 1
state 2

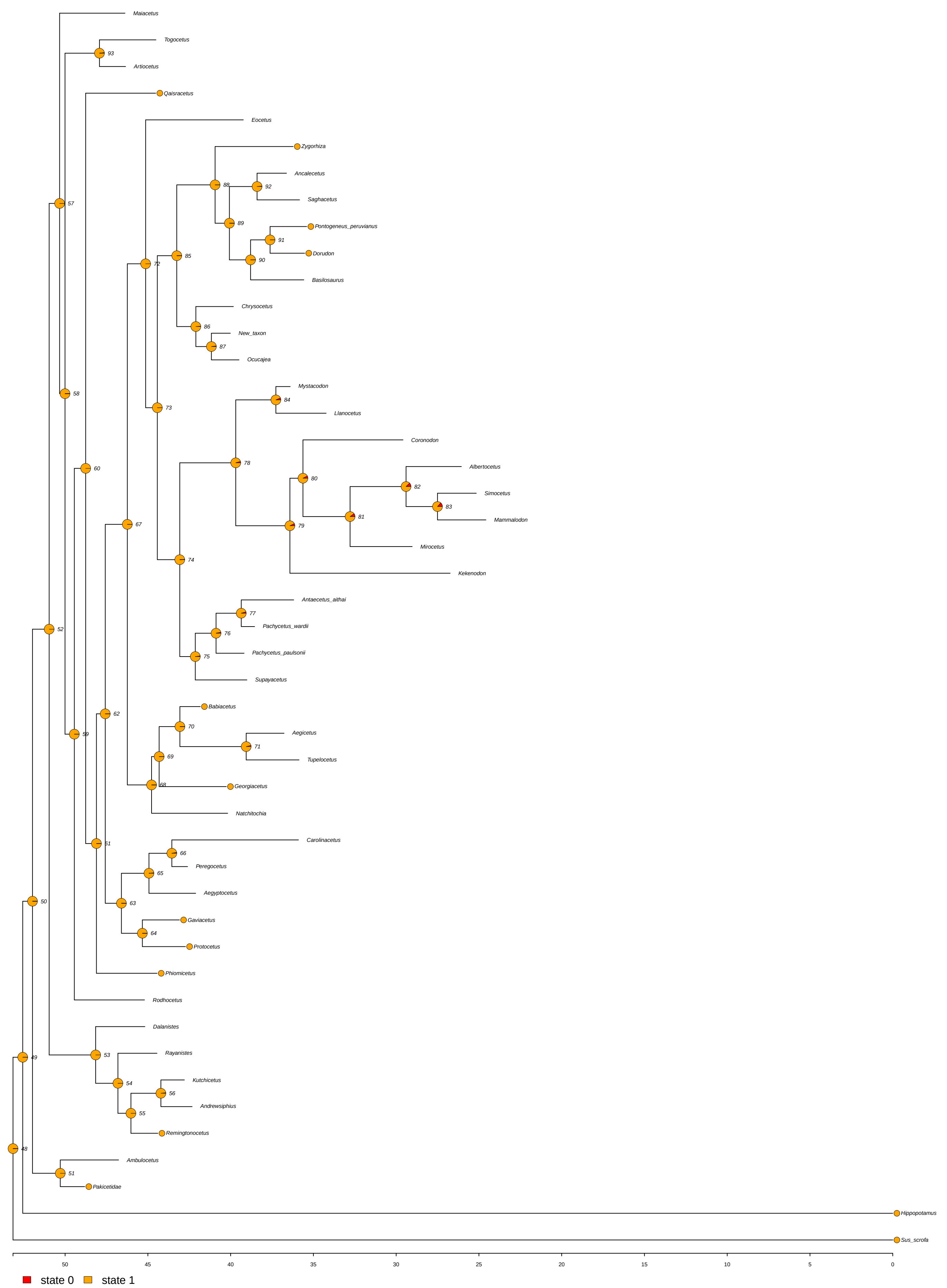

Maiacetus
Togocetus
93
Artiocetus
Qaisracetus
Eocetus
Zygorhiza
Ancalecetus
88
92
Saghacetus
89
Pontogeneus_peruvianus
91
Dorudon
90
Basilosaurus
57
72
85
Chrysocetus
86
New_taxon
87
Ocucajea
Mystacodon
84
Llanocetus
58
73
Coronodon
60
78
Albertocetus
80
82
Simocetus
83
Mammalodon
81
67
79
Mirocetus
74
Kekenodon
Antaecetus_aithai
77
Pachycetus_wardii
76
52
Pachycetus_paulsonii
75
Supayacetus
Babiacetus
62
70
Aegicetus
59
71
Tupelocetus
69
68
Georgiacetus
Natchitochia
Carolinacetus
61
66
Peregocetus
65
Aegyptocetus
50
63
Gaviacetus
64
Protocetus
Phiomicetus
Rodhocetus
Dalanistes
49
53
Rayanistes
54
Kutchicetus
56
Andrewsiphius
55
Remingtonocetus
48
Ambulocetus
51
Pakicetidae
Hippopotamus
Sus_scrofa
50
45
40
35
30
25
20
15
10
5
0
state 0
state 1

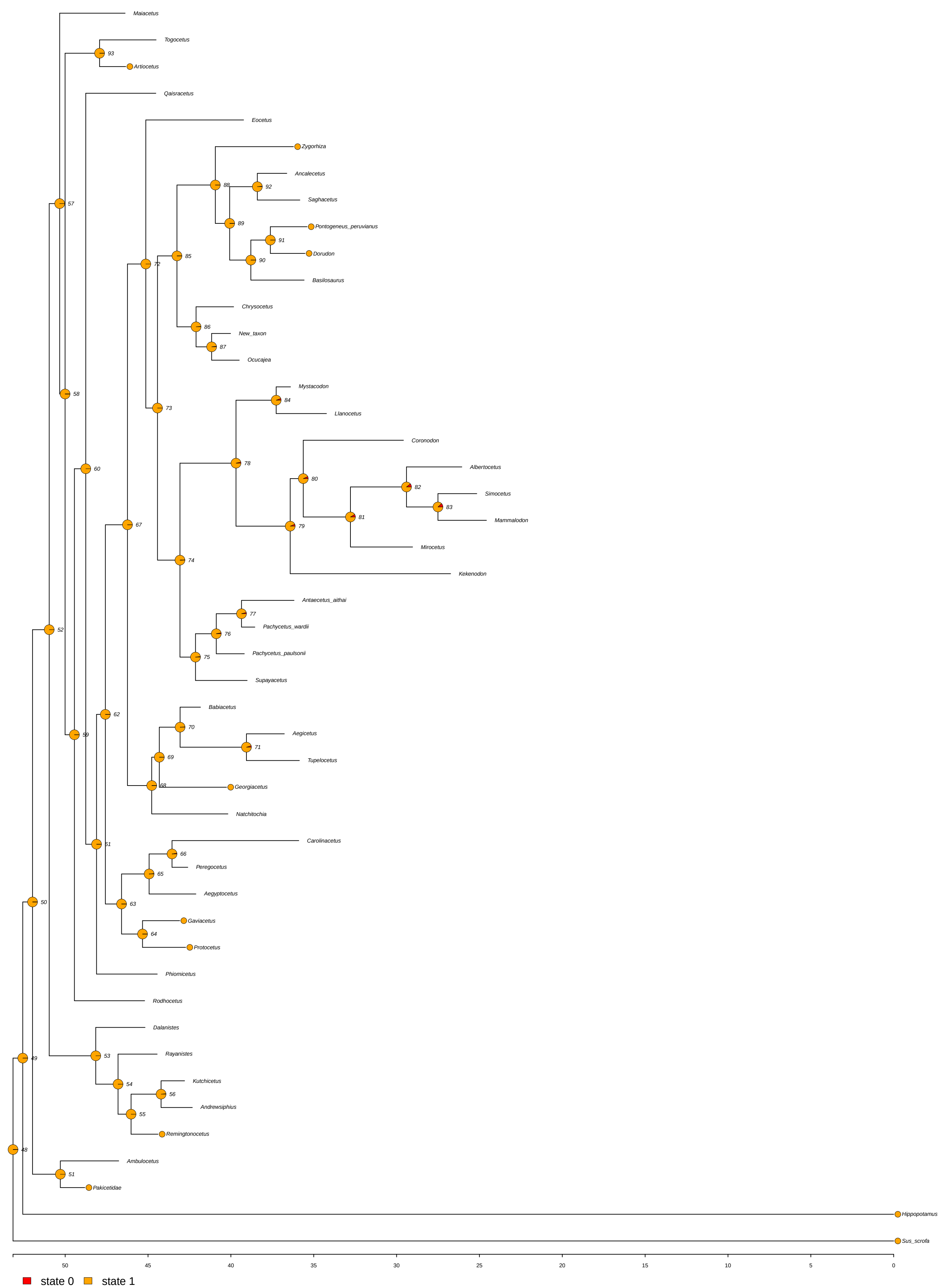

Maiacetus
Togocetus
Artiocetus
Qaisracetus
Eocetus
Zygorhiza
Ancalecetus
Saghacetus
Pontogeneus_peruvianus
Dorudon
Basilosaurus
Chrysocetus
New_taxon
Ocucajea
Mystacodon
Llanocetus
Coronodon
Albertocetus
Simocetus
Mammalodon
Mirocetus
Kekenodon
Antaecetus_aithai
Pachycetus_wardii
Pachycetus_paulsonii
Supayacetus
Babiacetus
Aegicetus
Tupelocetus
Georgiacetus
Natchitochia
Carolinacetus
Peregocetus
Aegyptocetus
Gaviacetus
Protocetus
Phiomicetus
Rodhocetus
Dalanistes
Rayanistes
Kutchicetus
Andrewsiphius
Remingtonocetus
Ambulocetus
Pakicetidae
Hippopotamus
Sus_scrofa
48
49
50
51
52
53
54
55
56
57
58
59
60
61
62
63
64
65
66
67
68
69
70
71
72
73
74
75
76
77
78
79
80
81
82
83
84
85
86
87
88
89
90
91
92
93
50
45
40
35
30
25
20
15
10
5
0
state 0
state 1

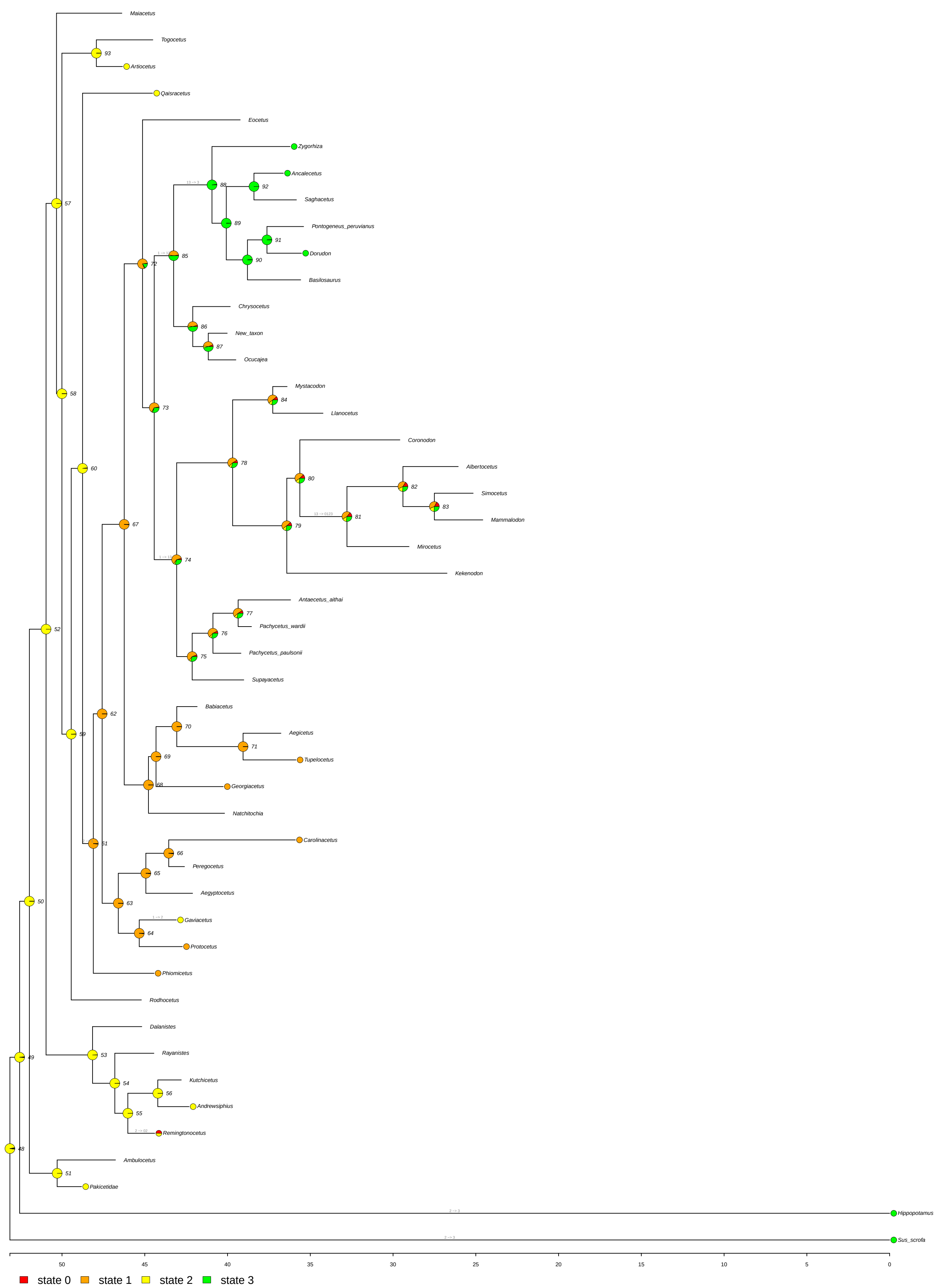

Maiacetus
Togocetus
Artiocetus
Qaisracetus
Eocetus
Zygorhiza
Ancalecetus
Saghacetus
Pontogeneus_peruvianus
Dorudon
Basilosaurus
Chrysocetus
New_taxon
Ocucajea
Mystacodon
Llanocetus
Coronodon
Albertocetus
Simocetus
Mammalodon
Mirocetus
Kekenodon
Antaecetus_aithai
Pachycetus_wardii
Pachycetus_paulsonii
Supayacetus
Babiacetus
Aegicetus
Tupelocetus
Georgiacetus
Natchitochia
Carolinacetus
Peregocetus
Aegyptocetus
Gaviacetus
Protocetus
Phiomicetus
Rodhocetus
Dalanistes
Rayanistes
Kutchicetus
Andrewsiphius
Remingtonocetus
Ambulocetus
Pakicetidae
Hippopotamus
Sus_scrofa
50
45
40
35
30
25
20
15
10
5
0
state 0
state 1
state 2
state 3

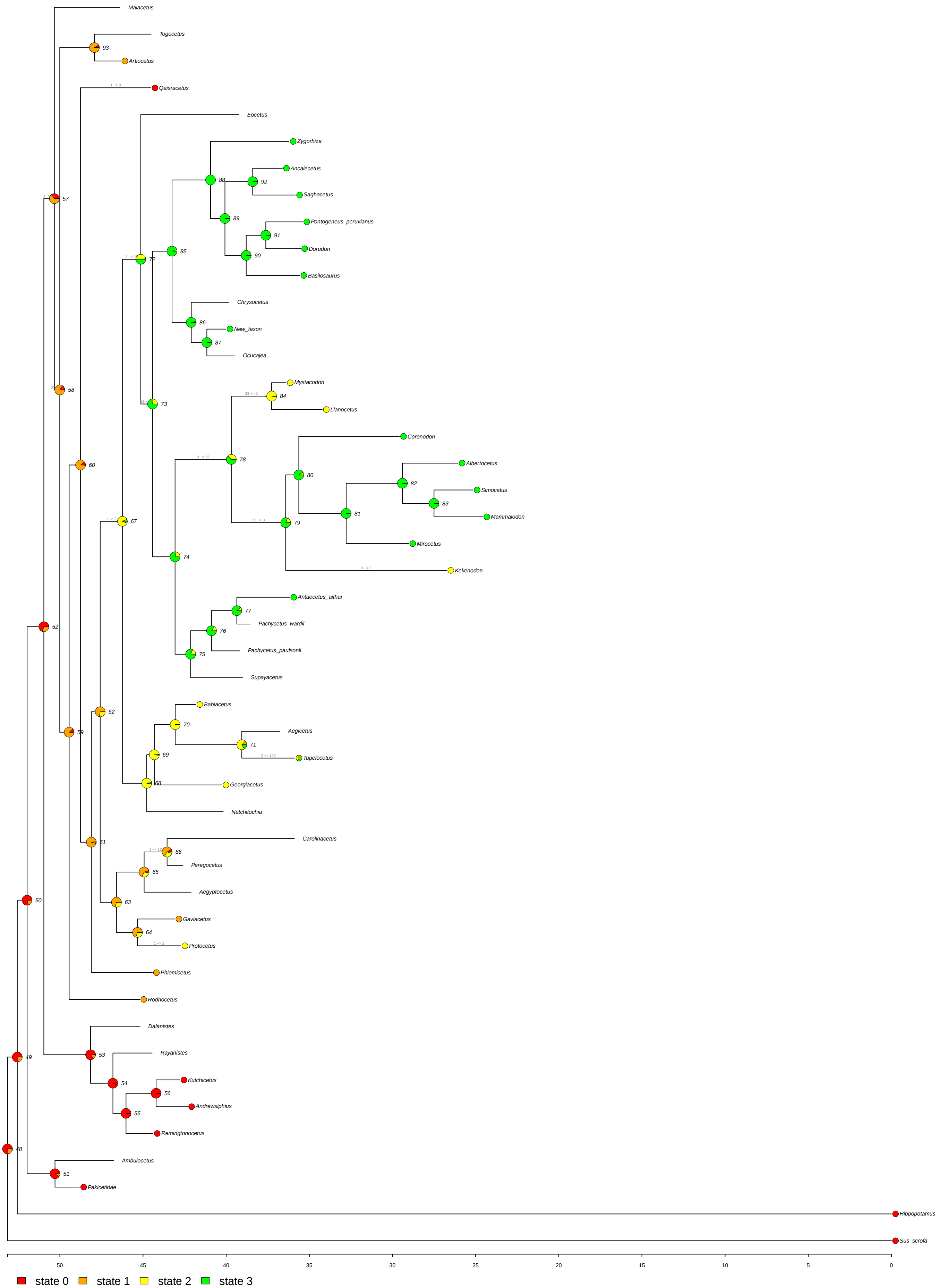
Maiacetus
Togocetus
93
Artiocetus
Qaisracetus
Eocetus
Zygorhiza
Ancalecetus
88
92
Saghacetus
57
89
Pontogeneus_peruvianus
91
Dorudon
72
85
90
Basilosaurus
Chrysocetus
86
New_taxon
87
Ocucajea
Mystacodon
84
58
Llanocetus
73
Coronodon
60
78
Albertocetus
80
82
Simocetus
83
81
Mammalodon
67
79
Mirocetus
74
Kekenodon
Antaecetus_aithai
77
Pachycetus_wardii
52
76
Pachycetus_paulsonii
75
Supayacetus
Babiacetus
62
59
70
Aegicetus
71
69
Tupelocetus
68
Georgiacetus
Natchitochia
Carolinacetus
61
66
Peregocetus
65
Aegyptocetus
50
63
Gaviacetus
64
Protocetus
Phiomicetus
Rodhocetus
Dalanistes
49
53
Rayanistes
54
Kutchicetus
56
55
Andrewsiphius
Remingtonocetus
48
Ambulocetus
51
Pakicetidae
Hippopotamus
Sus_scrofa
50
45
40
35
30
25
20
15
10
5
0
state 0
state 1
state 2
state 3

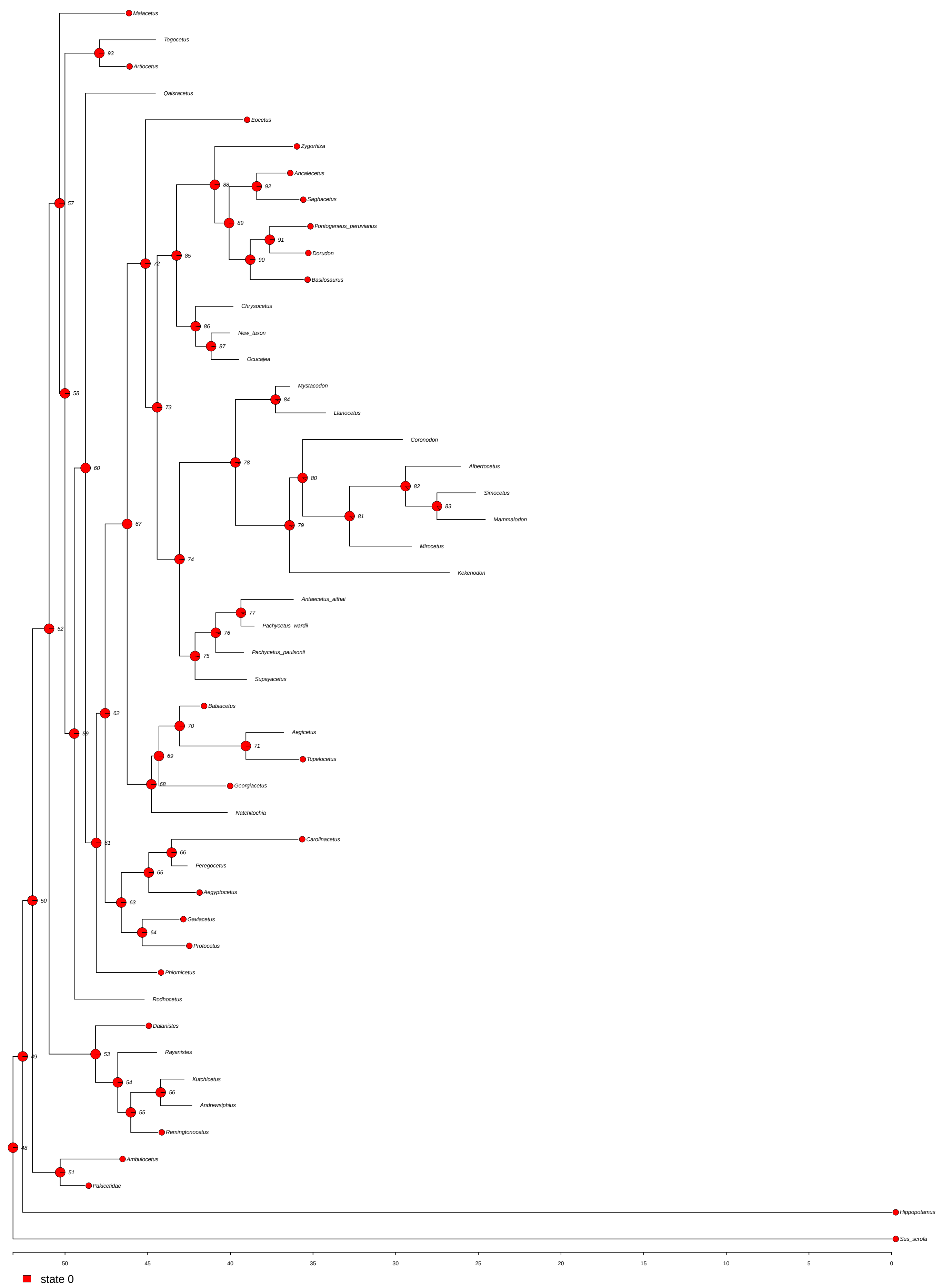

Maiacetus
Togocetus
93
Artiocetus
Qaisracetus
Eocetus
Zygorhiza
Ancalecetus
88
92
Saghacetus
57
Pontogeneus_peruvianus
89
91
Dorudon
90
Basilosaurus
72
85
Chrysocetus
86
New_taxon
87
Ocucajea
Mystacodon
58
84
Llanocetus
73
Coronodon
60
78
Albertocetus
80
82
Simocetus
83
Mammalodon
67
81
79
Mirocetus
74
Kekenodon
Antaecetus_aithai
77
Pachycetus_wardii
76
Pachycetus_paulsonii
52
75
Supayacetus
Babiacetus
62
70
Aegicetus
59
71
Tupelocetus
69
68
Georgiacetus
Natchitochia
Carolinacetus
61
66
Peregocetus
65
Aegyptocetus
50
63
Gaviacetus
64
Protocetus
Phiomicetus
Rodhocetus
Dalanistes
53
Rayanistes
49
54
Kutchicetus
56
55
Andrewsiphius
Remingtonocetus
48
Ambulocetus
51
Pakicetidae
Hippopotamus
Sus_scrofa
50
45
40
35
30
25
20
15
10
5
0
state 0

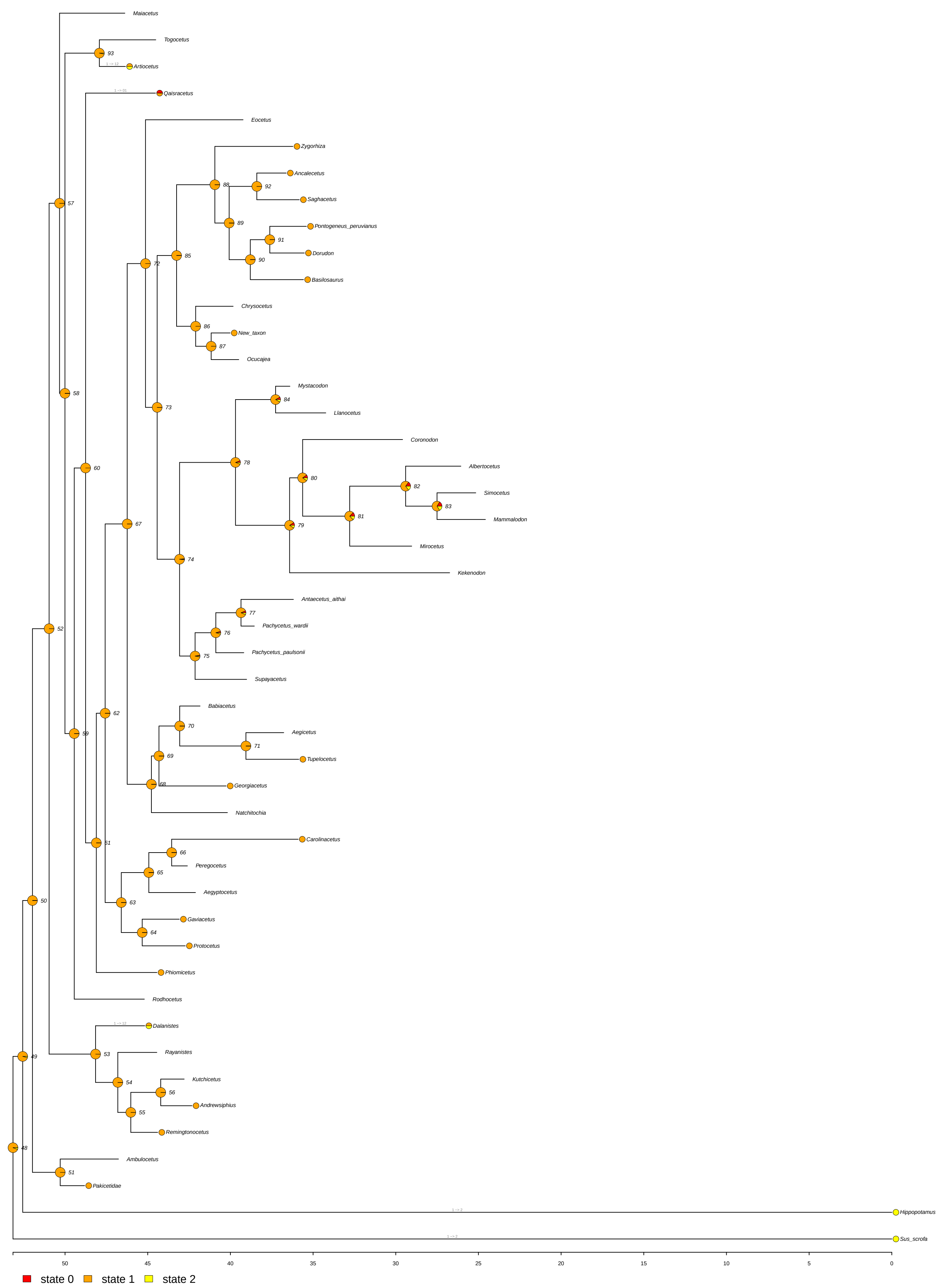

Maiacetus
Togocetus
93
Artiocetus
Qaisracetus
Eocetus
Zygorhiza
Ancalecetus
88
92
Saghacetus
57
Pontogeneus_peruvianus
89
91
Dorudon
90
72
85
Basilosaurus
Chrysocetus
86
New_taxon
87
Ocucajea
Mystacodon
84
58
Llanocetus
73
Coronodon
60
78
Albertocetus
80
82
Simocetus
83
81
Mammalodon
67
79
Mirocetus
74
Kekenodon
Antaecetus_aithai
77
Pachycetus_wardii
76
Pachycetus_paulsonii
52
75
Supayacetus
Babiacetus
62
70
Aegicetus
59
71
69
Tupelocetus
68
Georgiacetus
Natchitochia
Carolinacetus
61
66
Peregocetus
65
Aegyptocetus
50
63
Gaviacetus
64
Protocetus
Phiomicetus
Rodhocetus
Dalanistes
Rayanistes
49
53
Kutchicetus
54
56
Andrewsiphius
55
Remingtonocetus
48
Ambulocetus
51
Pakicetidae
Hippopotamus
Sus_scrofa
50
45
40
35
30
25
20
15
10
5
0
state 0
state 1
state 2

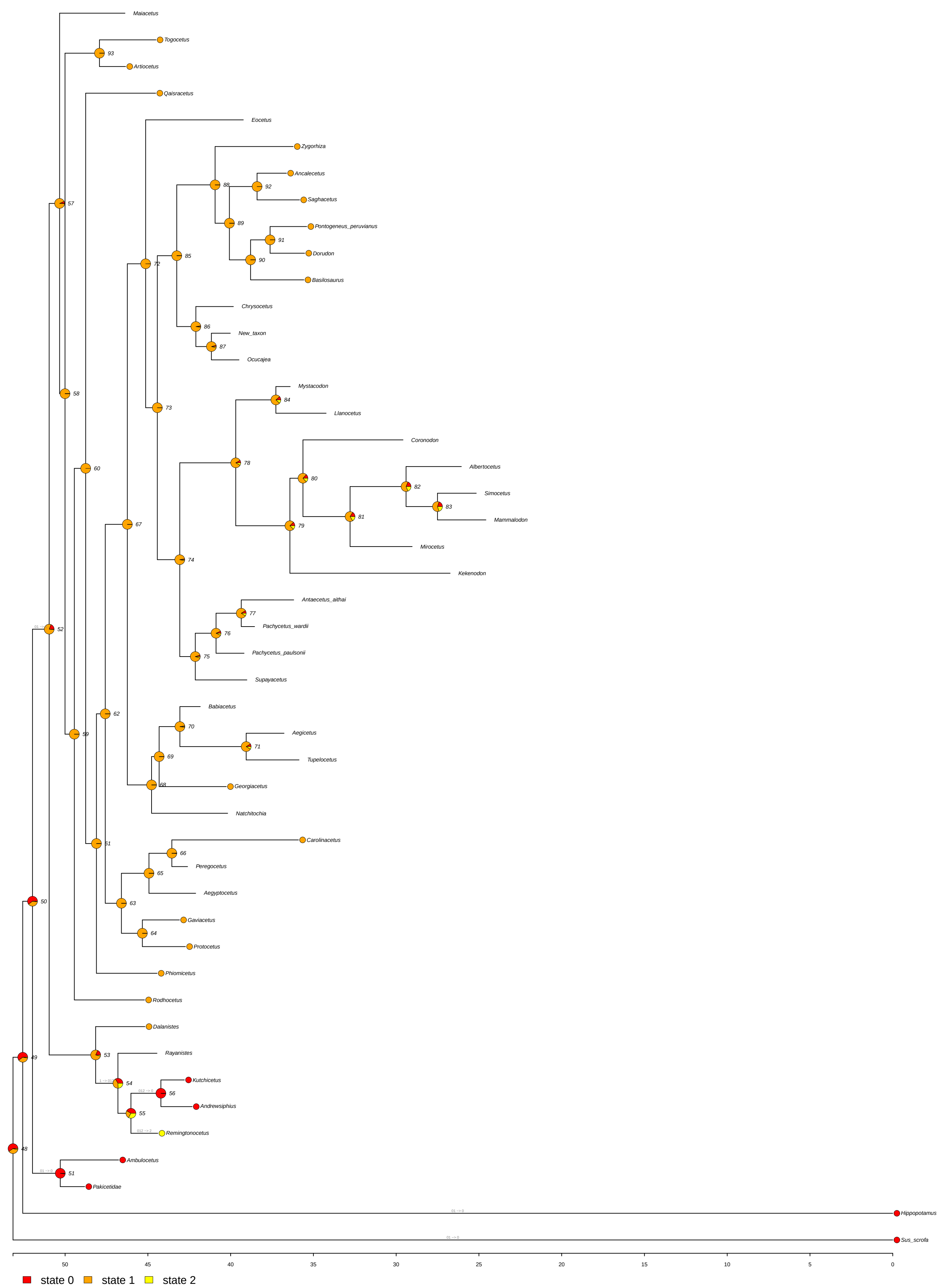

Maiacetus
Togocetus
Artiocetus
Qaisracetus
Eocetus
Zygorhiza
Ancalecetus
Saghacetus
Pontogeneus_peruvianus
Dorudon
Basilosaurus
Chrysocetus
New_taxon
Ocucajea
Mystacodon
Llanocetus
Coronodon
Albertocetus
Simocetus
Mammalodon
Mirocetus
Kekenodon
Antaecetus_aithai
Pachycetus_wardii
Pachycetus_paulsonii
Supayacetus
Babiacetus
Aegicetus
Tupelocetus
Georgiacetus
Natchitochia
Carolinacetus
Peregocetus
Aegyptocetus
Gaviacetus
Protocetus
Phiomicetus
Rodhocetus
Dalanistes
Rayanistes
Kutchicetus
Andrewsiphius
Remingtonocetus
Ambulocetus
Pakicetidae
Hippopotamus
Sus_scrofa
50
45
40
35
30
25
20
15
10
5
0
state 0
state 1
state 2

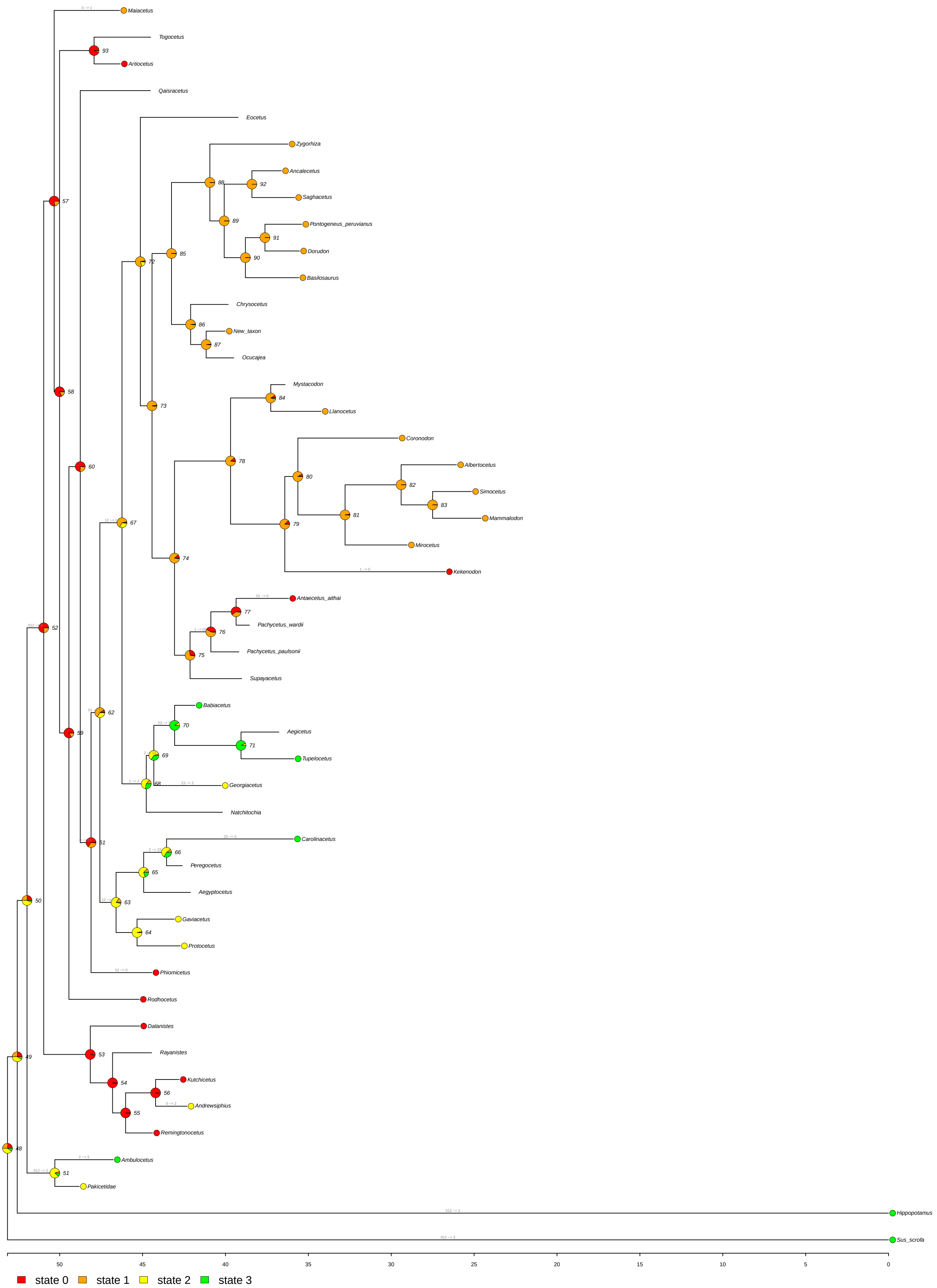

Maiacetus
Togocetus
Artiocetus
Qaisracetus
Eocetus
Zygorhiza
Ancalecetus
Saghacetus
Pontogeneus_peruvianus
Dorudon
Basilosaurus
Chrysocetus
New_taxon
Ocucajea
Mystacodon
Llanocetus
Coronodon
Albertocetus
Simocetus
Mammalodon
Mirocetus
Kekenodon
Antaecetus_aithai
Pachycetus_wardii
Pachycetus_paulsonii
Supayacetus
Babiacetus
Aegicetus
Tupelocetus
Georgiacetus
Natchitochia
Carolinacetus
Peregocetus
Aegyptocetus
Gaviacetus
Protocetus
Phiomicetus
Rodhocetus
Dalanistes
Rayanistes
Kutchicetus
Andrewsiphius
Remingtonocetus
Ambulocetus
Pakicetidae
Hippopotamus
Sus_scrofa
50
45
40
35
30
25
20
15
10
5
0
state 0
state 1
state 2
state 3

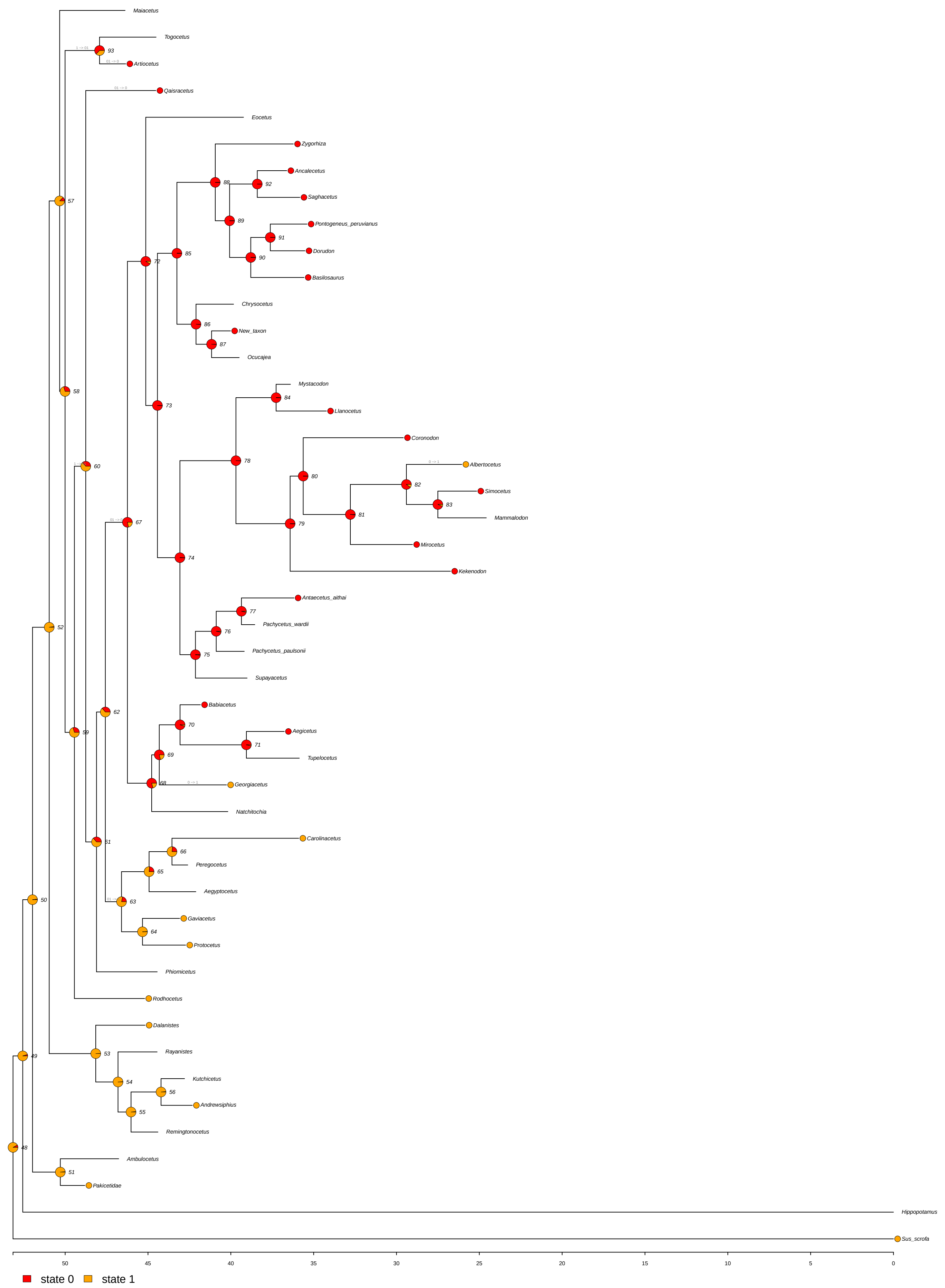

Maiacetus
Togocetus
93
Artiocetus
Qaisracetus
Eocetus
Zygorhiza
Ancalecetus
92
88
Saghacetus
89
Pontogeneus_peruvianus
91
Dorudon
90
Basilosaurus
72
85
57
Chrysocetus
86
New_taxon
87
Ocucajea
Mystacodon
84
58
Llanocetus
73
Coronodon
60
78
80
Albertocetus
82
Simocetus
83
Mammalodon
67
81
79
Mirocetus
74
Kekenodon
Antaecetus_aithai
77
Pachycetus_wardii
76
52
Pachycetus_paulsonii
75
Supayacetus
Babiacetus
62
70
59
Aegicetus
71
Tupelocetus
69
68
Georgiacetus
Natchitochia
Carolinacetus
61
66
Peregocetus
65
Aegyptocetus
50
63
Gaviacetus
64
Protocetus
Phiomicetus
Rodhocetus
Dalanistes
Rayanistes
53
49
54
Kutchicetus
56
Andrewsiphius
55
Remingtonocetus
48
Ambulocetus
51
Pakicetidae
Hippopotamus
Sus_scrofa
50
45
40
35
30
25
20
15
10
5
0
state 0
state 1

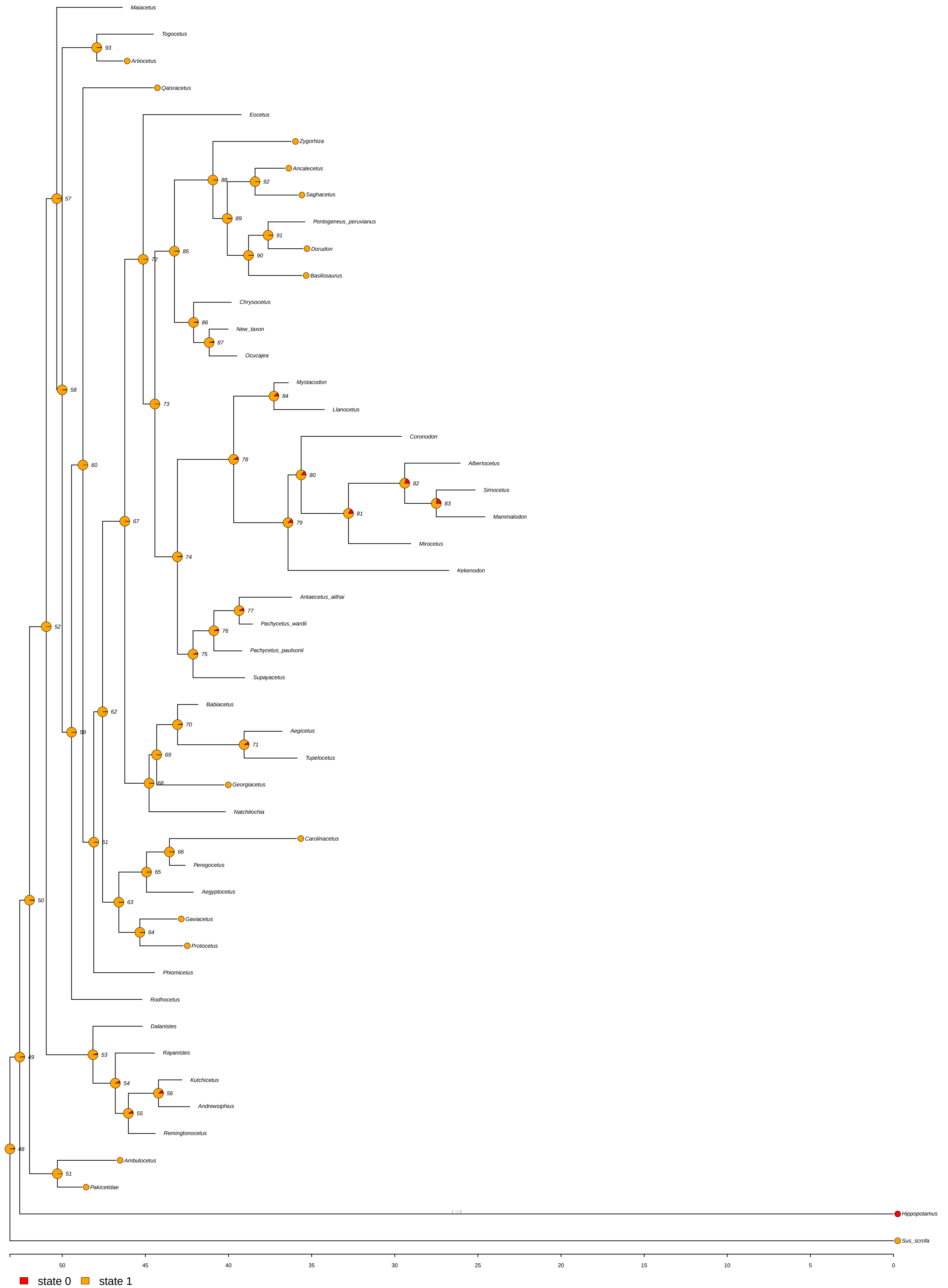
Maiacetus
Togocetus
Artiocetus
Qaisracetus
Eocetus
Zygorhiza
Ancalecetus
Saghacetus
Pontogeneus_peruvianus
Dorudon
Basilosaurus
Chrysocetus
New_taxon
Ocucajea
Mystacodon
Llanocetus
Coronodon
Albertocetus
Simocetus
Mammalodon
Mirocetus
Kekenodon
Antaecetus_aithai
Pachycetus_wardii
Pachycetus_paulsonii
Supayacetus
Babiacetus
Aegicetus
Tupelocetus
Georgiacetus
Natchitochia
Carolinacetus
Peregocetus
Aegyptocetus
Gaviacetus
Protocetus
Phiomicetus
Rodhocetus
Dalanistes
Rayanistes
Kutchicetus
Andrewsiphius
Remingtonocetus
Ambulocetus
Pakicetidae
Hippopotamus
Sus_scrofa
50
45
40
35
30
25
20
15
10
5
0
state 0
state 1

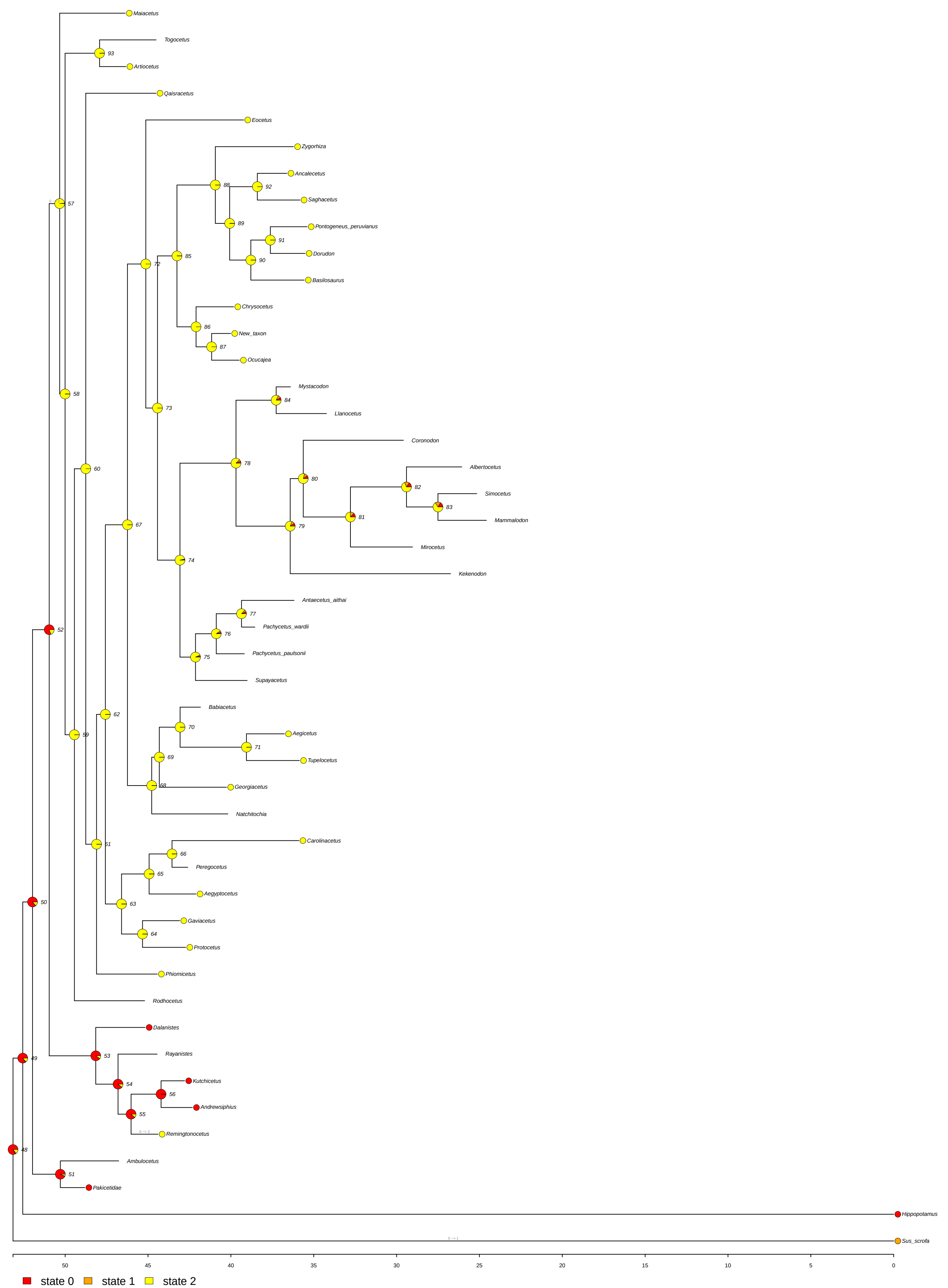

Maiacetus
Togocetus
93
Artiocetus
Qaisracetus
Eocetus
Zygorhiza
Ancalecetus
88
92
Saghacetus
57
89
Pontogeneus_peruvianus
91
Dorudon
90
72
85
Basilosaurus
Chrysocetus
86
New_taxon
87
Ocucajea
Mystacodon
84
58
73
Llanocetus
Coronodon
78
Albertocetus
60
80
82
Simocetus
83
81
Mammalodon
67
79
Mirocetus
74
Kekenodon
Antaecetus_aithai
77
Pachycetus_wardii
52
76
Pachycetus_paulsonii
75
Supayacetus
Babiacetus
62
70
Aegicetus
59
71
69
Tupelocetus
68
Georgiacetus
Natchitochia
Carolinacetus
61
66
Peregocetus
65
Aegyptocetus
50
63
Gaviacetus
64
Protocetus
Phiomicetus
Rodhocetus
Dalanistes
Rayanistes
49
53
Kutchicetus
54
56
Andrewsiphius
55
0 -> 2
Remingtonocetus
48
Ambulocetus
51
Pakicetidae
Hippopotamus
0 -> 1
Sus_scrofa
50
45
40
35
30
25
20
15
10
5
0
state 0
state 1
state 2

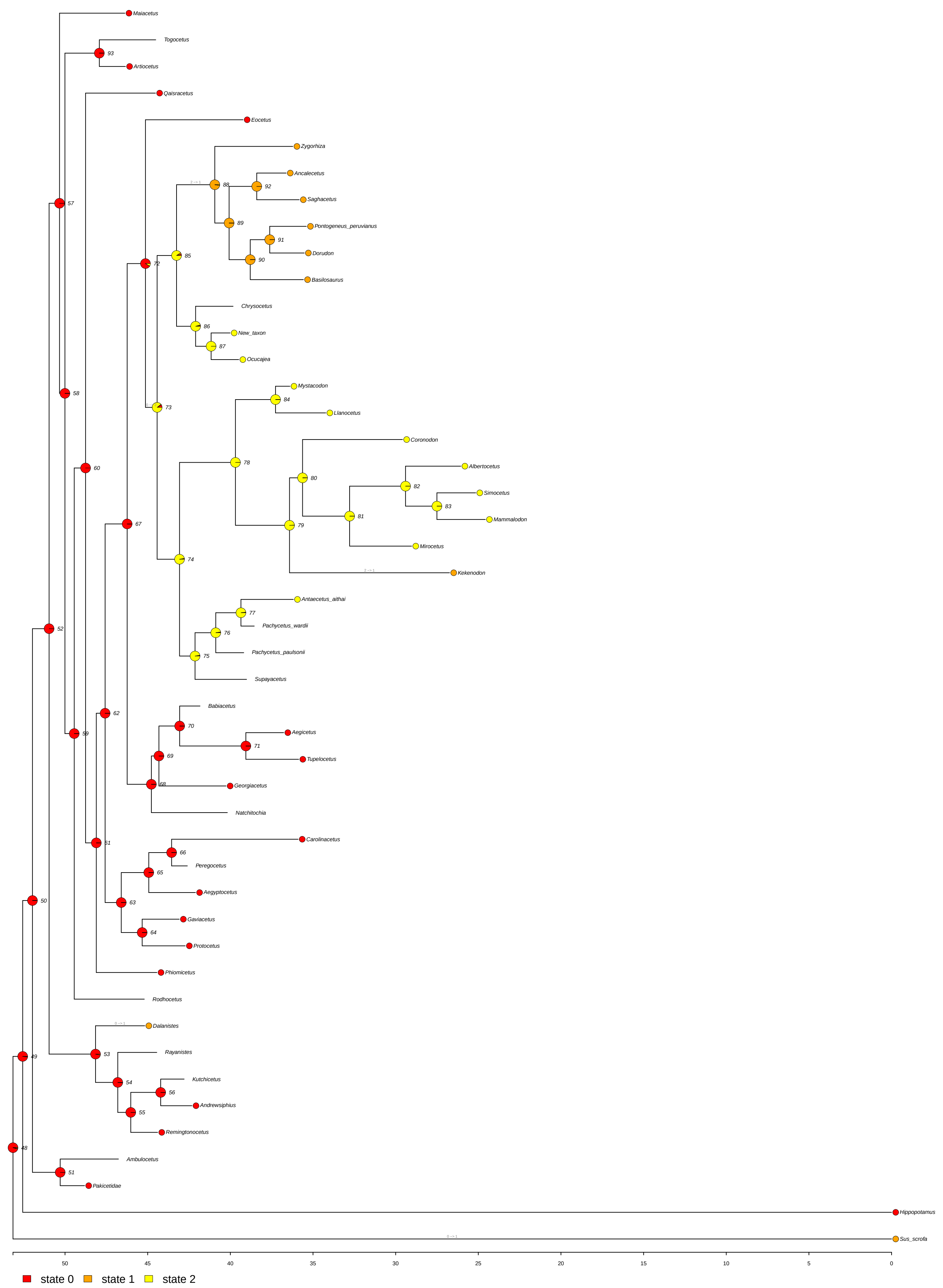

Maiacetus
Togocetus
93
Artiocetus
Qaisracetus
Eocetus
Zygorhiza
88
Ancalecetus
92
Saghacetus
89
Pontogeneus_peruvianus
91
Dorudon
90
Basilosaurus
72
85
Chrysocetus
86
New_taxon
87
Ocucajea
57
Mystacodon
84
Llanocetus
58
73
Coronodon
78
Albertocetus
80
82
Simocetus
83
Mammalodon
81
60
67
79
Mirocetus
74
Kekenodon
Antaecetus_aithai
77
Pachycetus_wardii
76
Pachycetus_paulsonii
75
Supayacetus
52
Babiacetus
62
70
59
Aegicetus
71
Tupelocetus
69
68
Georgiacetus
Natchitochia
Carolinacetus
61
66
Peregocetus
65
Aegyptocetus
50
63
Gaviacetus
64
Protocetus
Phiomicetus
Rodhocetus
Dalanistes
53
Rayanistes
49
54
Kutchicetus
56
Andrewsiphius
55
Remingtonocetus
48
Ambulocetus
51
Pakicetidae
Hippopotamus
Sus_scrofa
50
45
40
35
30
25
20
15
10
5
0
state 0
state 1
state 2

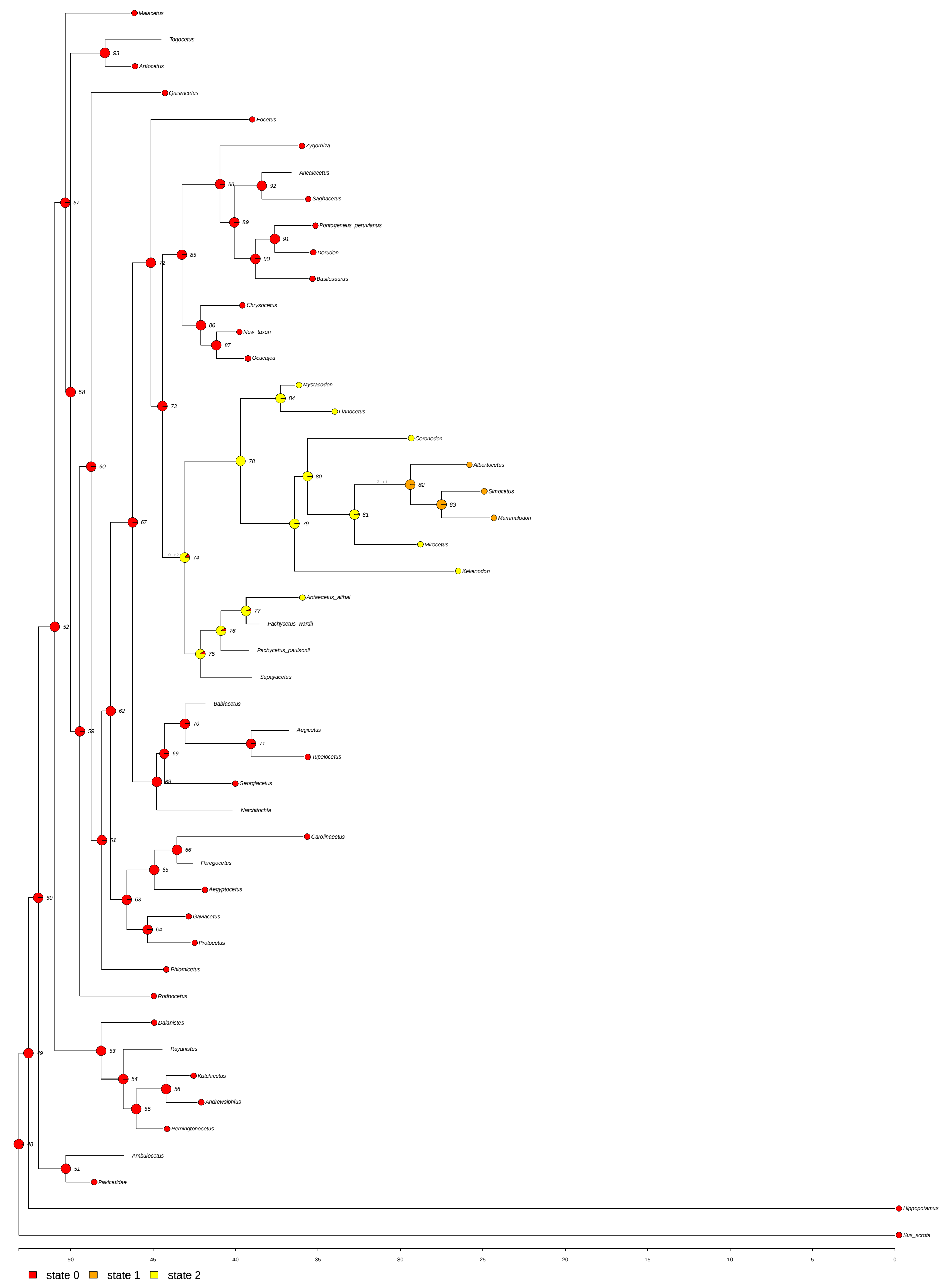
Maiacetus
Togocetus
Artiocetus
Qaisracetus
Eocetus
Zygorhiza
Ancalecetus
Saghacetus
Pontogeneus_peruvianus
Dorudon
Basilosaurus
Chrysocetus
New_taxon
Ocucajea
Mystacodon
Llanocetus
Coronodon
Albertocetus
Simocetus
Mammalodon
Mirocetus
Kekenodon
Antaecetus_aithai
Pachycetus_wardii
Pachycetus_paulsonii
Supayacetus
Babiacetus
Aegicetus
Tupelocetus
Georgiacetus
Natchitochia
Carolinacetus
Peregocetus
Aegyptocetus
Gaviacetus
Protocetus
Phiomicetus
Rodhocetus
Dalanistes
Rayanistes
Kutchicetus
Andrewsiphius
Remingtonocetus
Ambulocetus
Pakicetidae
Hippopotamus
Sus_scrofa
50
45
40
35
30
25
20
15
10
5
0
state 0
state 1
state 2

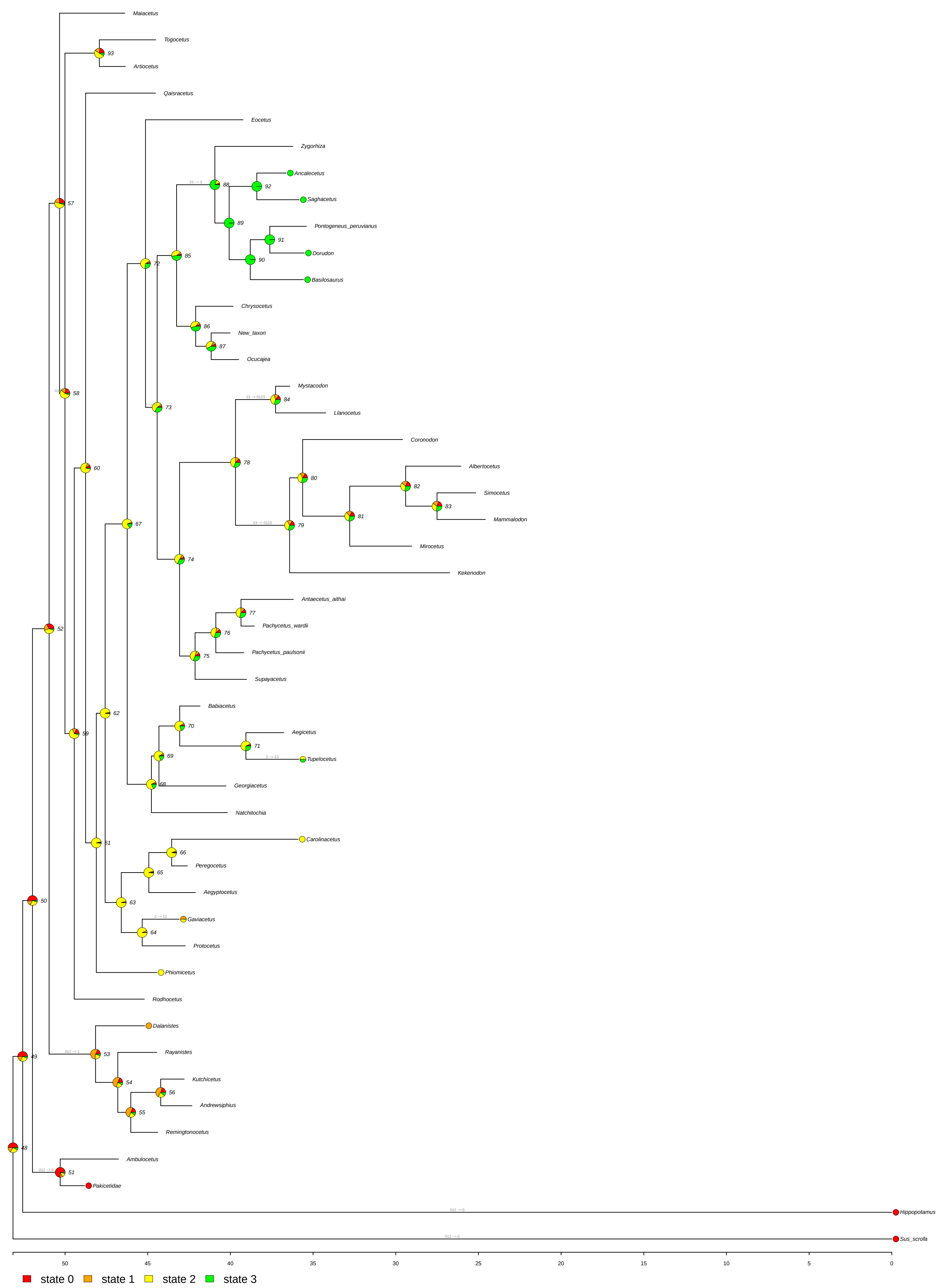
Maiacetus
Togocetus
93
Artiocetus
Qaisracetus
Eocetus
Zygorhiza
Ancalecetus
88
92
Saghacetus
89
Pontogeneus_peruvianus
91
Dorudon
90
Basilosaurus
72
85
Chrysocetus
86
New_taxon
87
Ocucajea
57
58
73
Mystacodon
84
Llanocetus
Coronodon
60
78
Albertocetus
80
82
Simocetus
83
Mammalodon
67
81
79
Mirocetus
74
Kekenodon
Antaecetus_aithai
77
Pachycetus_wardii
76
52
Pachycetus_paulsonii
75
Supayacetus
Babiacetus
62
70
Aegicetus
59
71
69
Tupelocetus
68
Georgiacetus
Natchitochia
61
Carolinacetus
66
Peregocetus
65
Aegyptocetus
50
63
Gaviacetus
64
Protocetus
Phiomicetus
Rodhocetus
Dalanistes
Rayanistes
49
53
54
Kutchicetus
56
Andrewsiphius
55
Remingtonocetus
48
Ambulocetus
51
Pakicetidae
Hippopotamus
Sus_scrofa
50
45
40
35
30
25
20
15
10
5
0
state 0
state 1
state 2
state 3

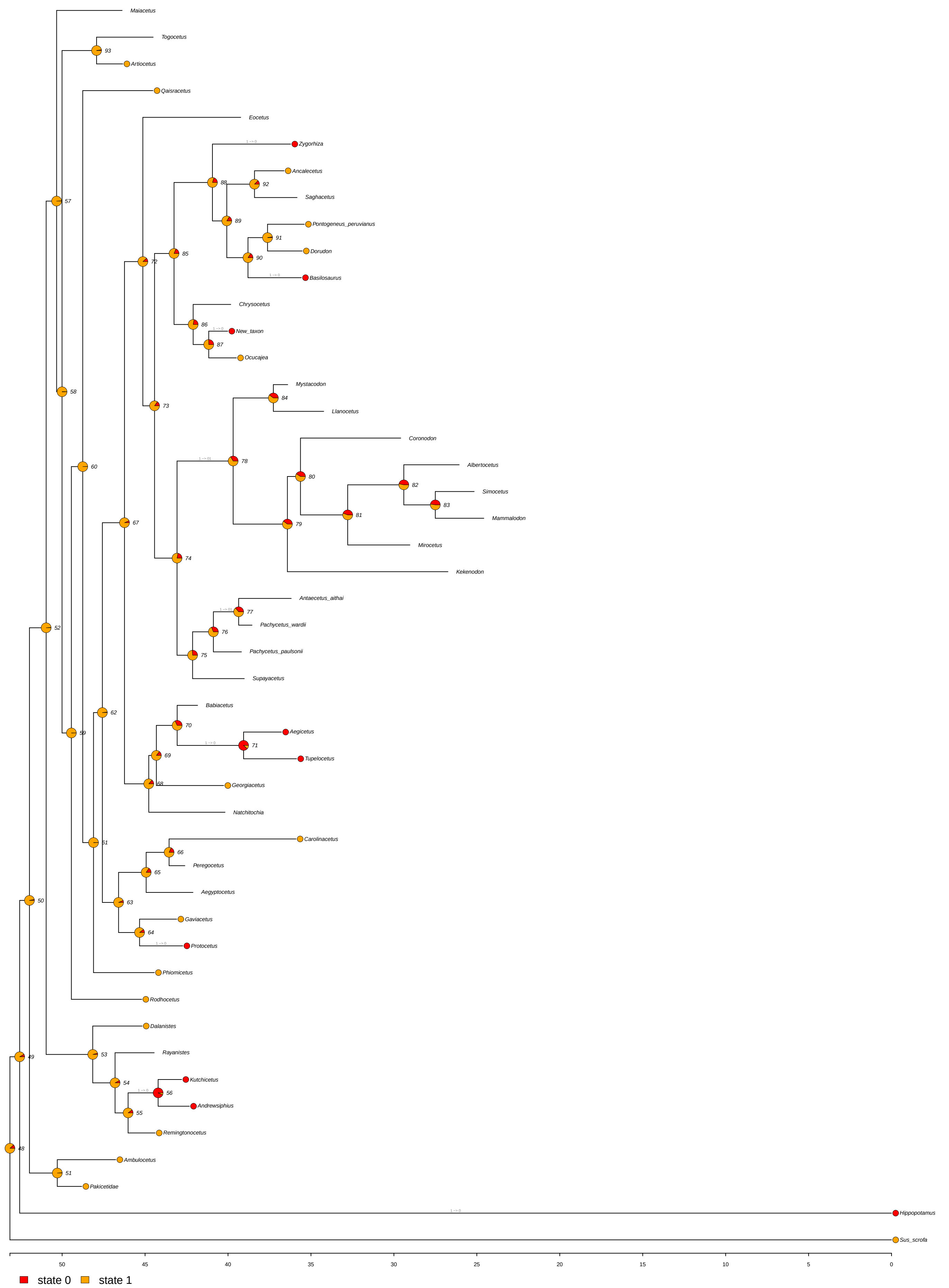

Maiacetus
Togocetus
93
Artiocetus
Qaisracetus
Eocetus
1 -> 0
Zygorhiza
Ancalecetus
88
92
Saghacetus
89
Pontogeneus_peruvianus
91
57
Dorudon
72
85
90
1 -> 0
Basilosaurus
Chrysocetus
86
1 -> 0
New_taxon
87
Ocucajea
Mystacodon
84
58
73
Llanocetus
Coronodon
60
1 -> 01
78
Albertocetus
80
82
Simocetus
83
67
81
Mammalodon
79
Mirocetus
74
Kekenodon
Antaecetus_aithai
1 -> 01
77
Pachycetus_wardii
52
76
Pachycetus_paulsonii
75
Supayacetus
Babiacetus
62
70
Aegicetus
59
1 -> 0
71
69
Tupelocetus
68
Georgiacetus
Natchitochia
61
Carolinacetus
66
Peregocetus
65
Aegyptocetus
50
63
Gaviacetus
64
1 -> 0
Protocetus
Phiomicetus
Rodhocetus
Dalanistes
49
53
Rayanistes
Kutchicetus
54
1 -> 0
56
55
Andrewsiphius
Remingtonocetus
48
Ambulocetus
51
Pakicetidae
1 -> 0
Hippopotamus
Sus_scrofa
50
45
40
35
30
25
20
15
10
5
0
state 0
state 1

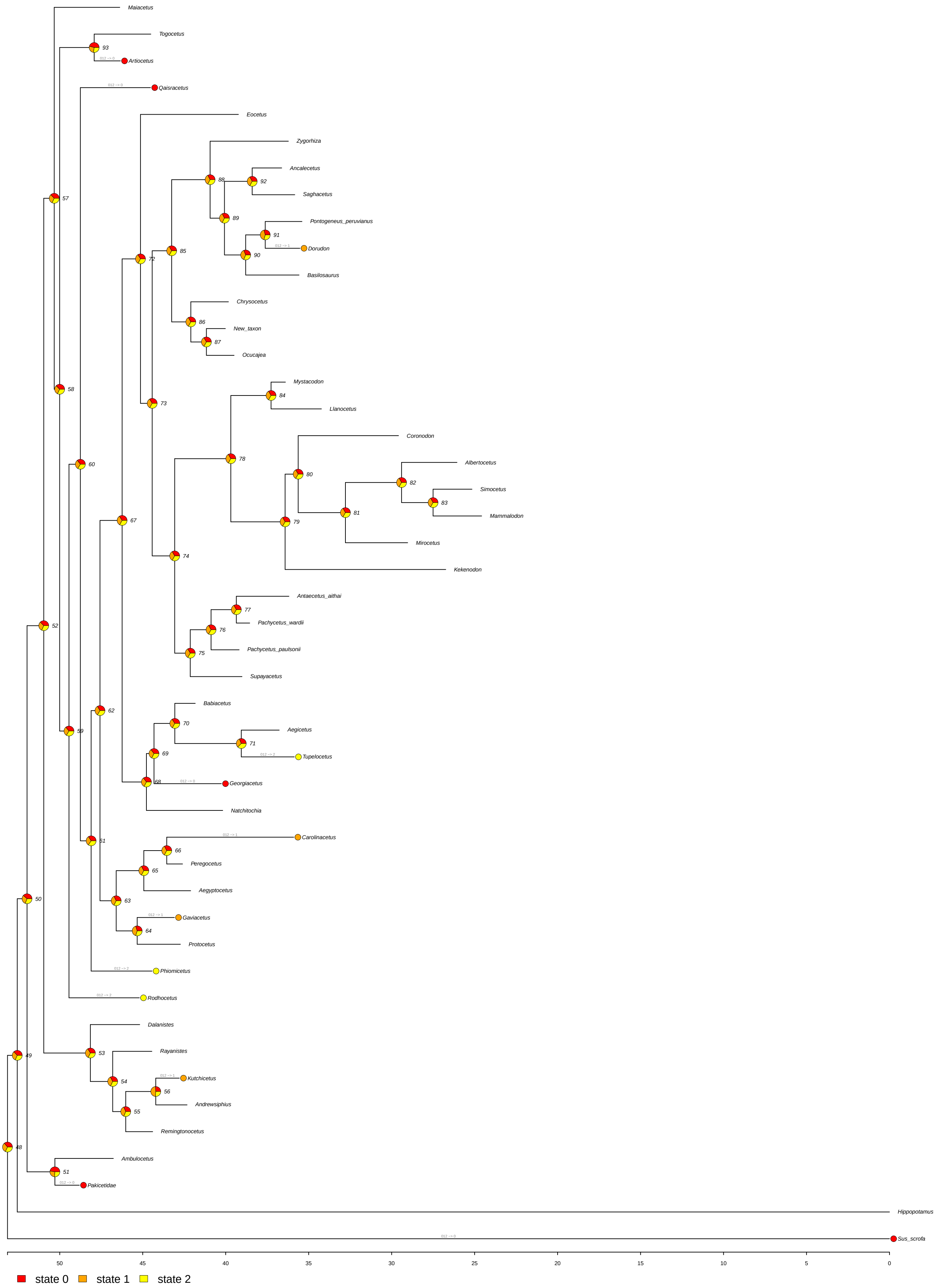

Maiacetus
Togocetus
Artiocetus
Qaisracetus
Eocetus
Zygorhiza
Ancalecetus
Saghacetus
Pontogeneus_peruvianus
Dorudon
Basilosaurus
Chrysocetus
New_taxon
Ocucajea
Mystacodon
Llanocetus
Coronodon
Albertocetus
Simocetus
Mammalodon
Mirocetus
Kekenodon
Antaecetus_aithai
Pachycetus_wardii
Pachycetus_paulsonii
Supayacetus
Babiacetus
Aegicetus
Tupelocetus
Georgiacetus
Natchitochia
Carolinacetus
Peregocetus
Aegyptocetus
Gaviacetus
Protocetus
Phiomicetus
Rodhocetus
Dalanistes
Rayanistes
Kutchicetus
Andrewsiphius
Remingtonocetus
Ambulocetus
Pakicetidae
Hippopotamus
Sus_scrofa
50
45
40
35
30
25
20
15
10
5
0
state 0
state 1
state 2

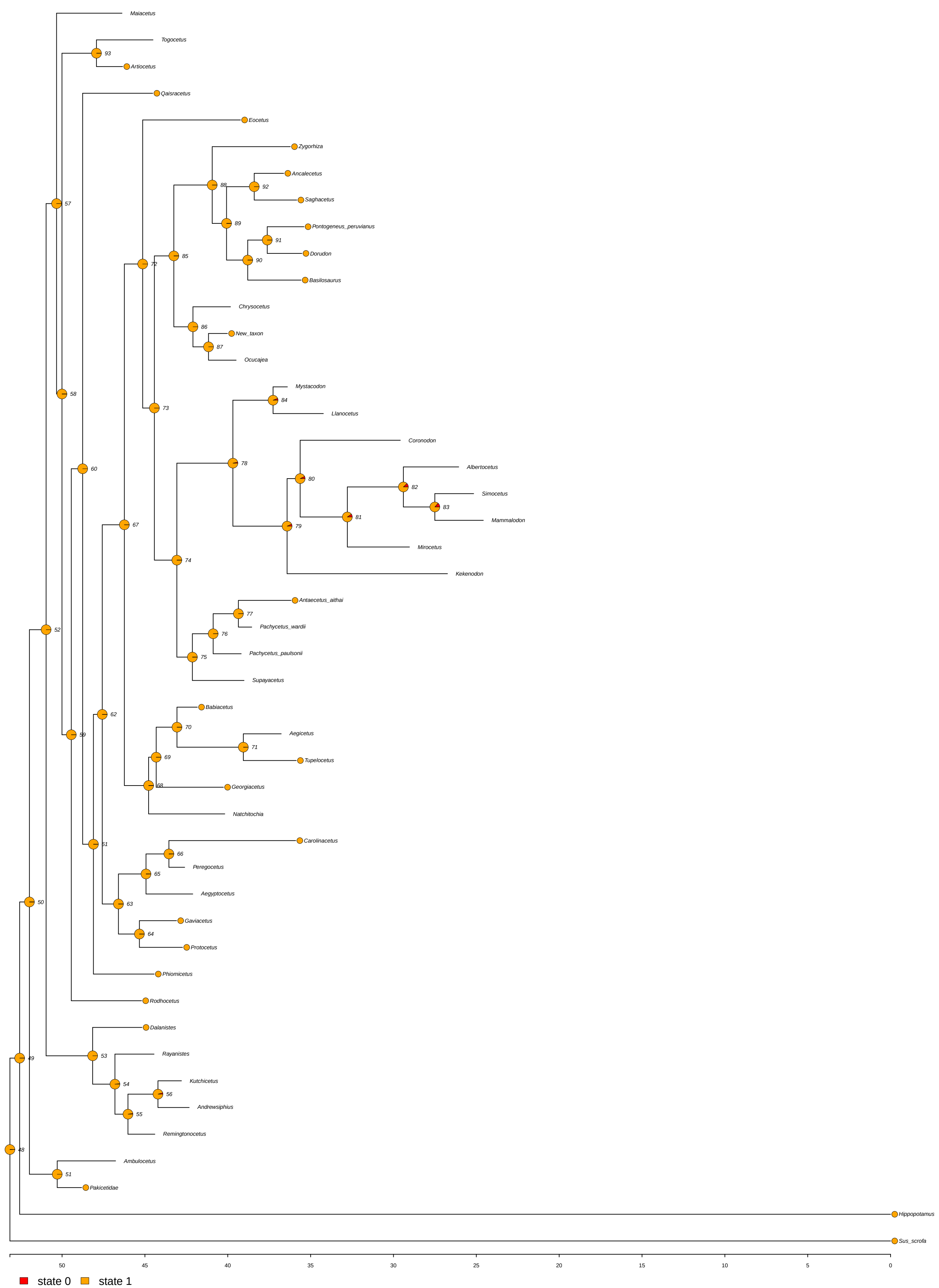
Maiacetus
Togocetus
Artiocetus
Qaisracetus
Eocetus
Zygorhiza
Ancalecetus
Saghacetus
Pontogeneus_peruvianus
Dorudon
Basilosaurus
Chrysocetus
New_taxon
Ocucajea
Mystacodon
Llanocetus
Coronodon
Albertocetus
Simocetus
Mammalodon
Mirocetus
Kekenodon
Antaecetus_aithai
Pachycetus_wardii
Pachycetus_paulsonii
Supayacetus
Babiacetus
Aegicetus
Tupelocetus
Georgiacetus
Natchitochia
Carolinacetus
Peregocetus
Aegyptocetus
Gaviacetus
Protocetus
Phiomicetus
Rodhocetus
Dalanistes
Rayanistes
Kutchicetus
Andrewsiphius
Remingtonocetus
Ambulocetus
Pakicetidae
Hippopotamus
Sus_scrofa
93
57
88
92
89
91
90
72
85
86
87
58
84
73
78
60
80
82
83
81
67
79
74
77
76
75
62
70
59
71
69
68
61
66
65
63
64
53
54
56
55
50
49
48
51
52
50
45
40
35
30
25
20
15
10
5
0
state 0
state 1

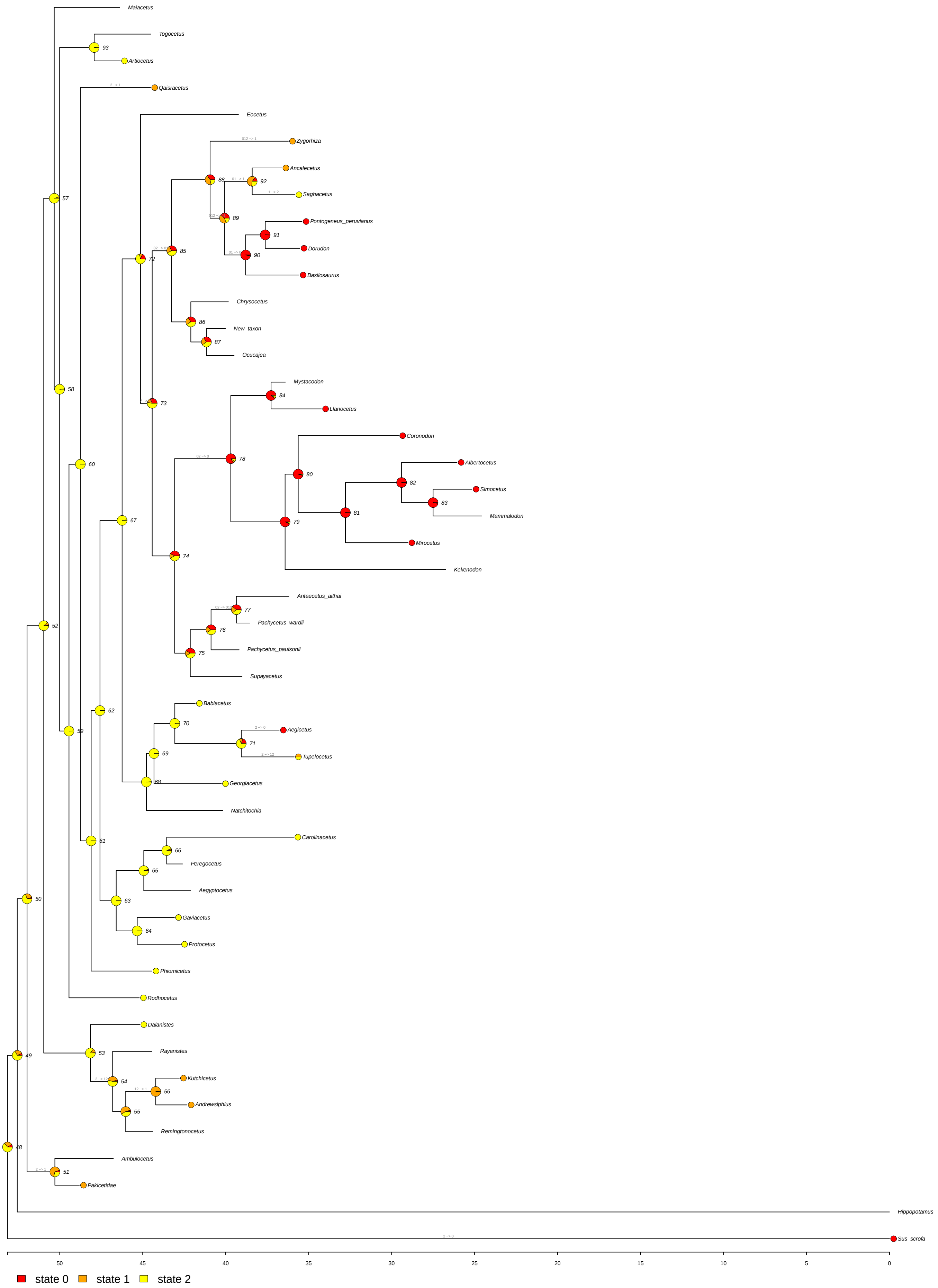
Maiacetus
Togocetus
Artiocetus
Qaisracetus
Eocetus
Zygorhiza
Ancalecetus
Saghacetus
Pontogeneus_peruvianus
Dorudon
Basilosaurus
Chrysocetus
New_taxon
Ocucajea
Mystacodon
Llanocetus
Coronodon
Albertocetus
Simocetus
Mammalodon
Mirocetus
Kekenodon
Antaecetus_aithai
Pachycetus_wardii
Pachycetus_paulsonii
Supayacetus
Babiacetus
Aegicetus
Tupelocetus
Georgiacetus
Natchitochia
Carolinacetus
Peregocetus
Aegyptocetus
Gaviacetus
Protocetus
Phiomicetus
Rodhocetus
Dalanistes
Rayanistes
Kutchicetus
Andrewsiphius
Remingtonocetus
Ambulocetus
Pakicetidae
Hippopotamus
Sus_scrofa
50
45
40
35
30
25
20
15
10
5
0
state 0
state 1
state 2

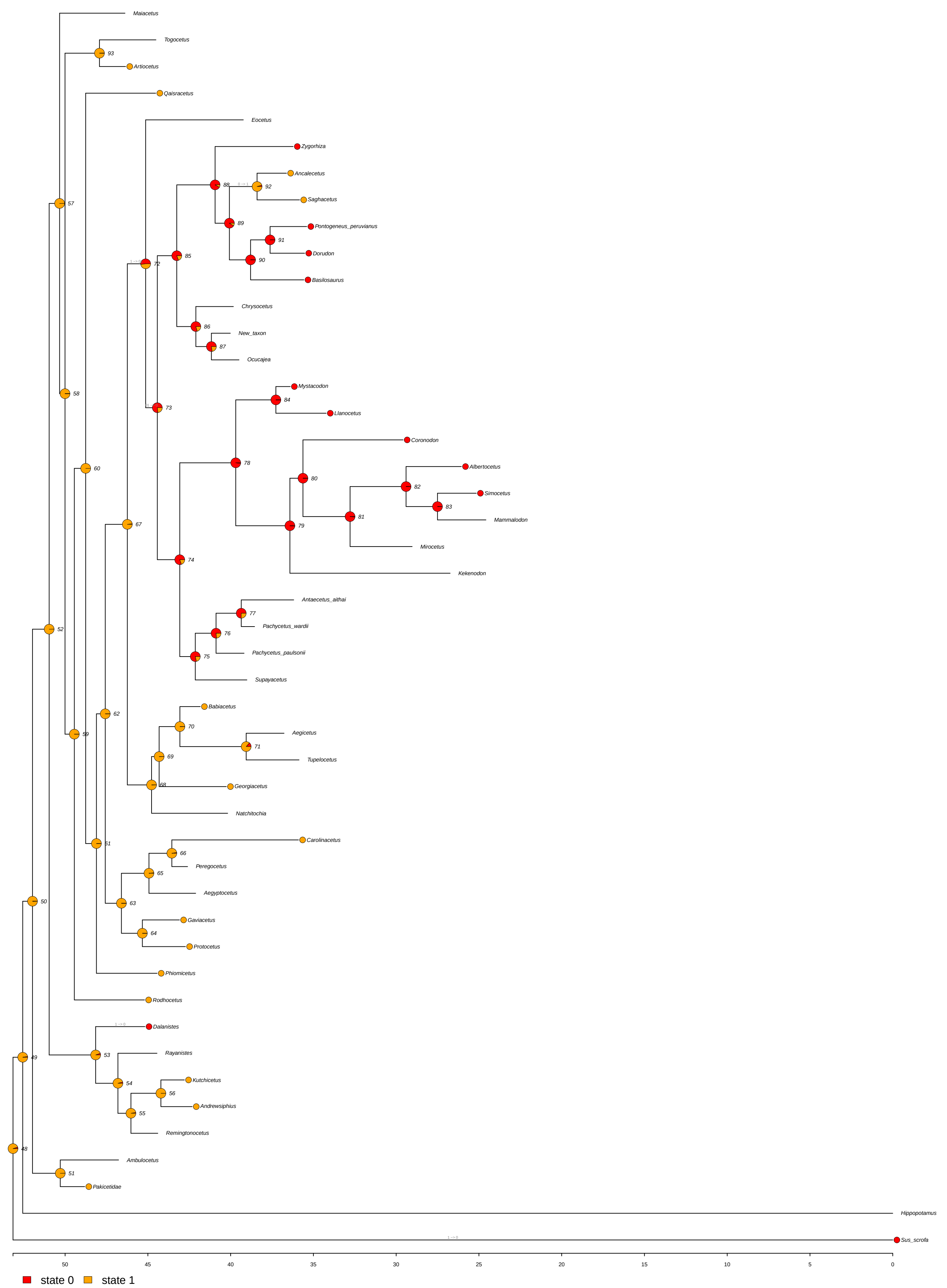

Maiacetus
Togocetus
Artiocetus
Qaisracetus
Eocetus
Zygorhiza
Ancalecetus
Saghacetus
Pontogeneus_peruvianus
Dorudon
Basilosaurus
Chrysocetus
New_taxon
Ocucajea
Mystacodon
Llanocetus
Coronodon
Albertocetus
Simocetus
Mammalodon
Mirocetus
Kekenodon
Antaecetus_aithai
Pachycetus_wardii
Pachycetus_paulsonii
Supayacetus
Babiacetus
Aegicetus
Tupelocetus
Georgiacetus
Natchitochia
Carolinacetus
Peregocetus
Aegyptocetus
Gaviacetus
Protocetus
Phiomicetus
Rodhocetus
Dalanistes
Rayanistes
Kutchicetus
Andrewsiphius
Remingtonocetus
Ambulocetus
Pakicetidae
Hippopotamus
Sus_scrofa
93
57
88
92
89
91
90
85
72
86
87
58
73
84
78
80
82
83
81
79
60
67
74
77
76
75
62
70
71
69
59
68
61
66
65
63
64
50
53
49
54
56
55
48
51
52
0 -> 1
1 -> 0
50
45
40
35
30
25
20
15
10
5
0
state 0
state 1

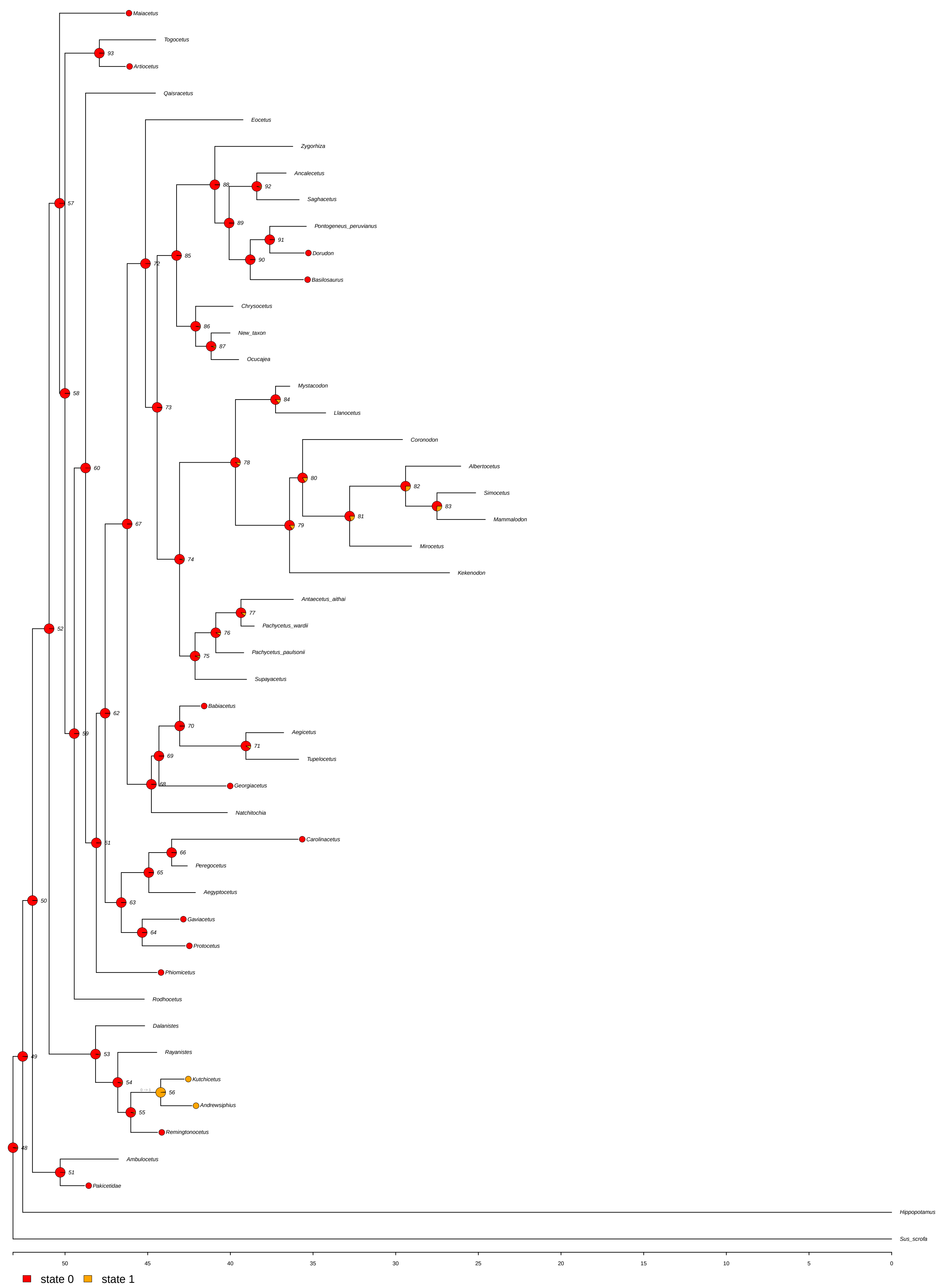

Maiacetus
Togocetus
93
Artiocetus
Qaisracetus
Eocetus
Zygorhiza
Ancalecetus
88
92
Saghacetus
89
Pontogeneus_peruvianus
91
Dorudon
90
Basilosaurus
57
72
85
Chrysocetus
86
New_taxon
87
Ocucajea
Mystacodon
84
Llanocetus
58
73
Coronodon
60
78
Albertocetus
80
82
Simocetus
83
Mammalodon
81
67
79
Mirocetus
74
Kekenodon
Antaecetus_aithai
77
Pachycetus_wardii
52
76
Pachycetus_paulsonii
75
Supayacetus
Babiacetus
62
70
Aegicetus
59
71
Tupelocetus
69
68
Georgiacetus
Natchitochia
61
Carolinacetus
66
Peregocetus
65
Aegyptocetus
50
63
Gaviacetus
64
Protocetus
Phiomicetus
Rodhocetus
Dalanistes
53
Rayanistes
49
54
Kutchicetus
56
Andrewsiphius
55
Remingtonocetus
48
Ambulocetus
51
Pakicetidae
Hippopotamus
Sus_scrofa
50
45
40
35
30
25
20
15
10
5
0
state 0
state 1

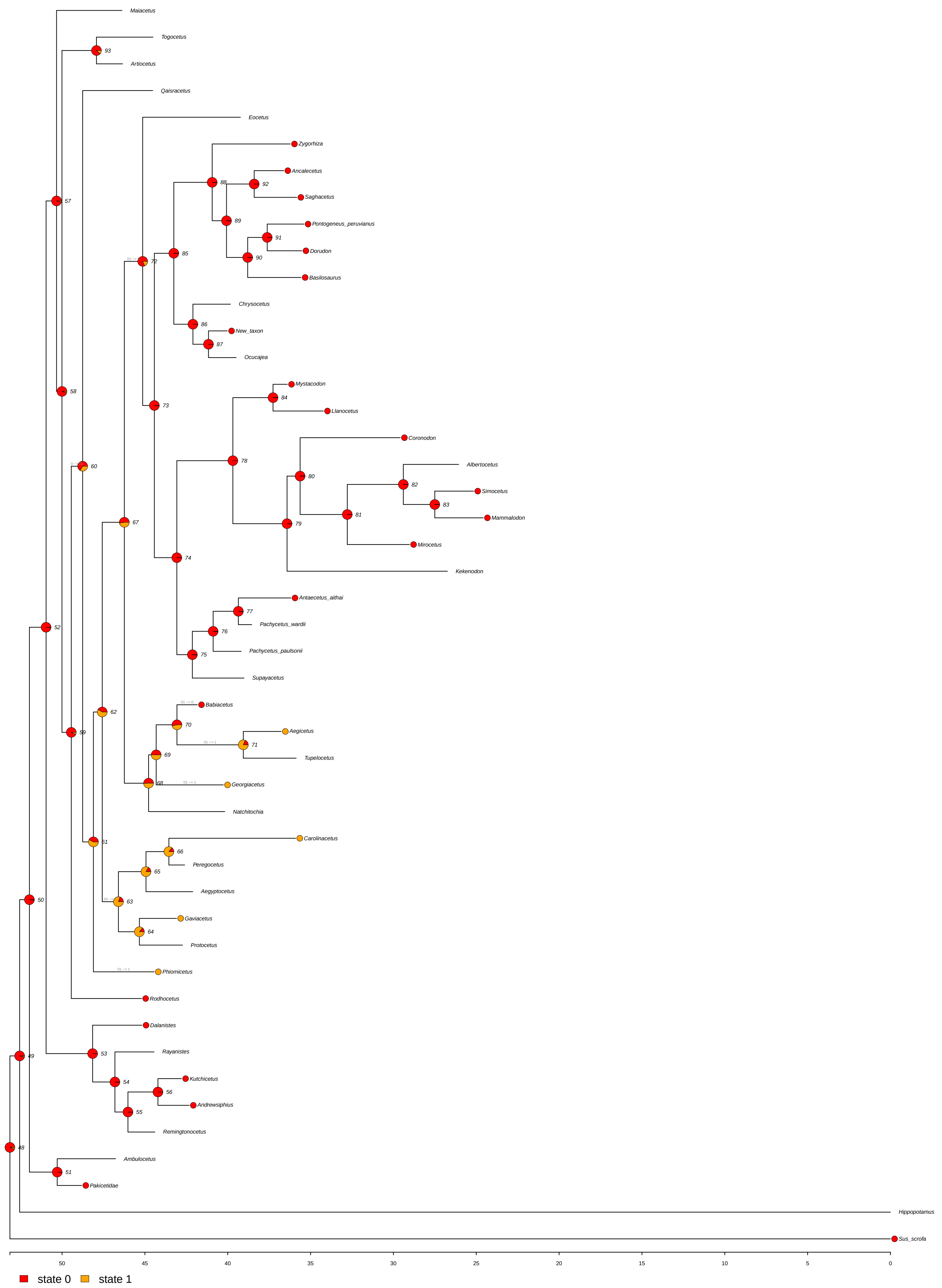
Maiacetus
Togocetus
93
Artiocetus
Qaisracetus
Eocetus
Zygorhiza
Ancalecetus
88
92
Saghacetus
57
Pontogeneus_peruvianus
89
91
Dorudon
72
85
90
Basilosaurus
Chrysocetus
86
New_taxon
87
Ocucajea
Mystacodon
84
58
73
Llanocetus
Coronodon
60
78
Albertocetus
80
82
Simocetus
83
67
81
Mammalodon
79
Mirocetus
74
Kekenodon
Antaecetus_aithai
77
Pachycetus_wardii
52
76
Pachycetus_paulsonii
75
Supayacetus
Babiacetus
62
70
Aegicetus
59
71
69
Tupelocetus
68
Georgiacetus
Natchitochia
61
Carolinacetus
66
Peregocetus
65
Aegyptocetus
50
63
Gaviacetus
64
Protocetus
Phiomicetus
Rodhocetus
Dalanistes
53
Rayanistes
49
54
Kutchicetus
56
Andrewsiphius
55
Remingtonocetus
48
Ambulocetus
51
Pakicetidae
Hippopotamus
Sus_scrofa
50
45
40
35
30
25
20
15
10
5
0
state 0
state 1

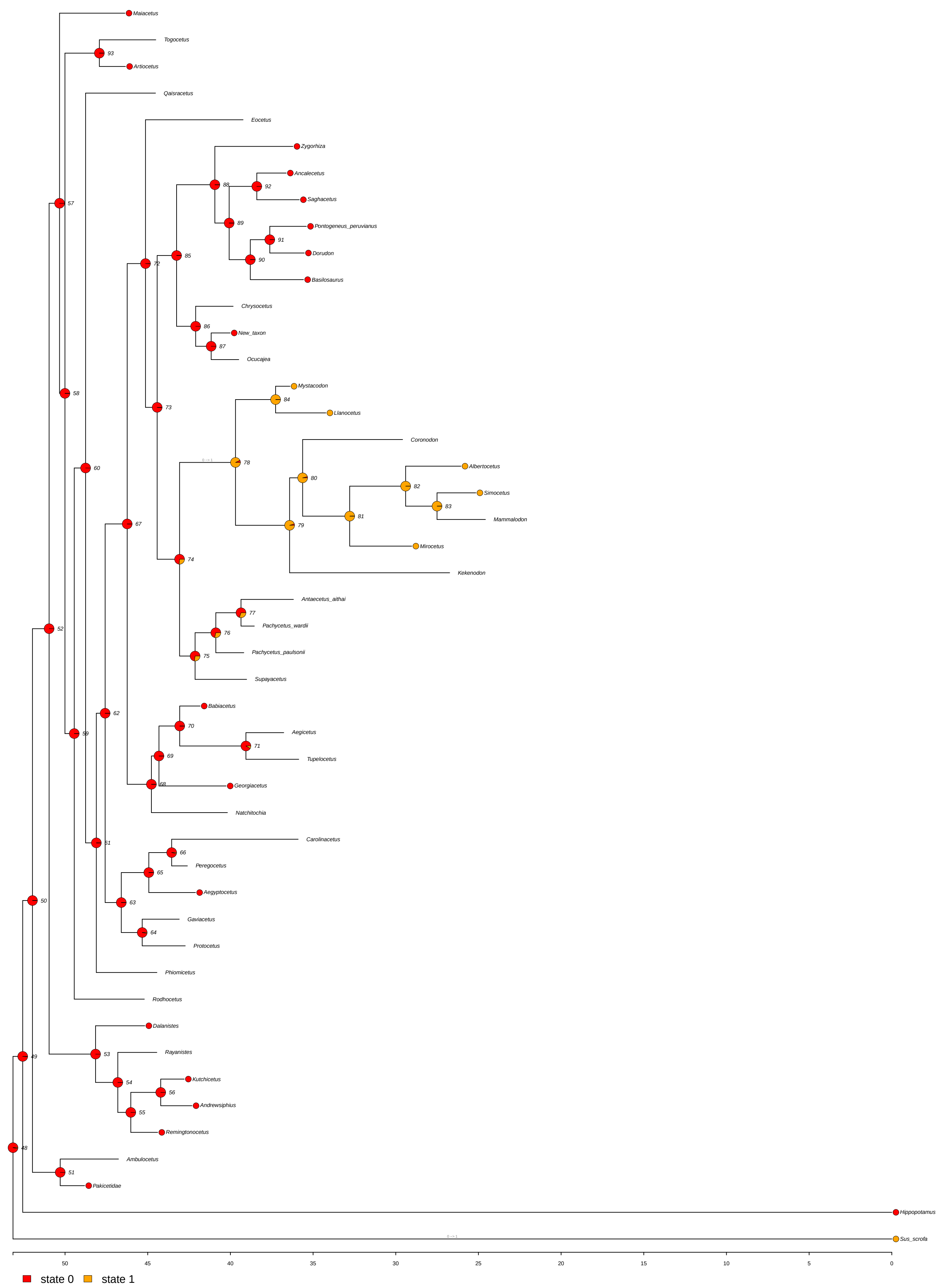
Maiacetus
Togocetus
93
Artiocetus
Qaisracetus
Eocetus
Zygorhiza
Ancalecetus
88
92
Saghacetus
57
Pontogeneus_peruvianus
89
91
Dorudon
72
85
90
Basilosaurus
Chrysocetus
86
New_taxon
87
Ocucajea
Mystacodon
58
84
73
Llanocetus
Coronodon
0 --> 1
78
Albertocetus
60
80
82
Simocetus
83
67
81
Mammalodon
79
Mirocetus
74
Kekenodon
Antaecetus_aithai
77
Pachycetus_wardii
52
76
Pachycetus_paulsonii
75
Supayacetus
Babiacetus
62
Aegicetus
59
70
71
Tupelocetus
69
68
Georgiacetus
Natchitochia
Carolinacetus
61
66
Peregocetus
65
Aegyptocetus
50
63
Gaviacetus
64
Protocetus
Phiomicetus
Rodhocetus
Dalanistes
53
Rayanistes
49
54
Kutchicetus
56
55
Andrewsiphius
Remingtonocetus
48
Ambulocetus
51
Pakicetidae
Hippopotamus
0 --> 1
Sus_scrofa
50
45
40
35
30
25
20
15
10
5
0
state 0
state 1

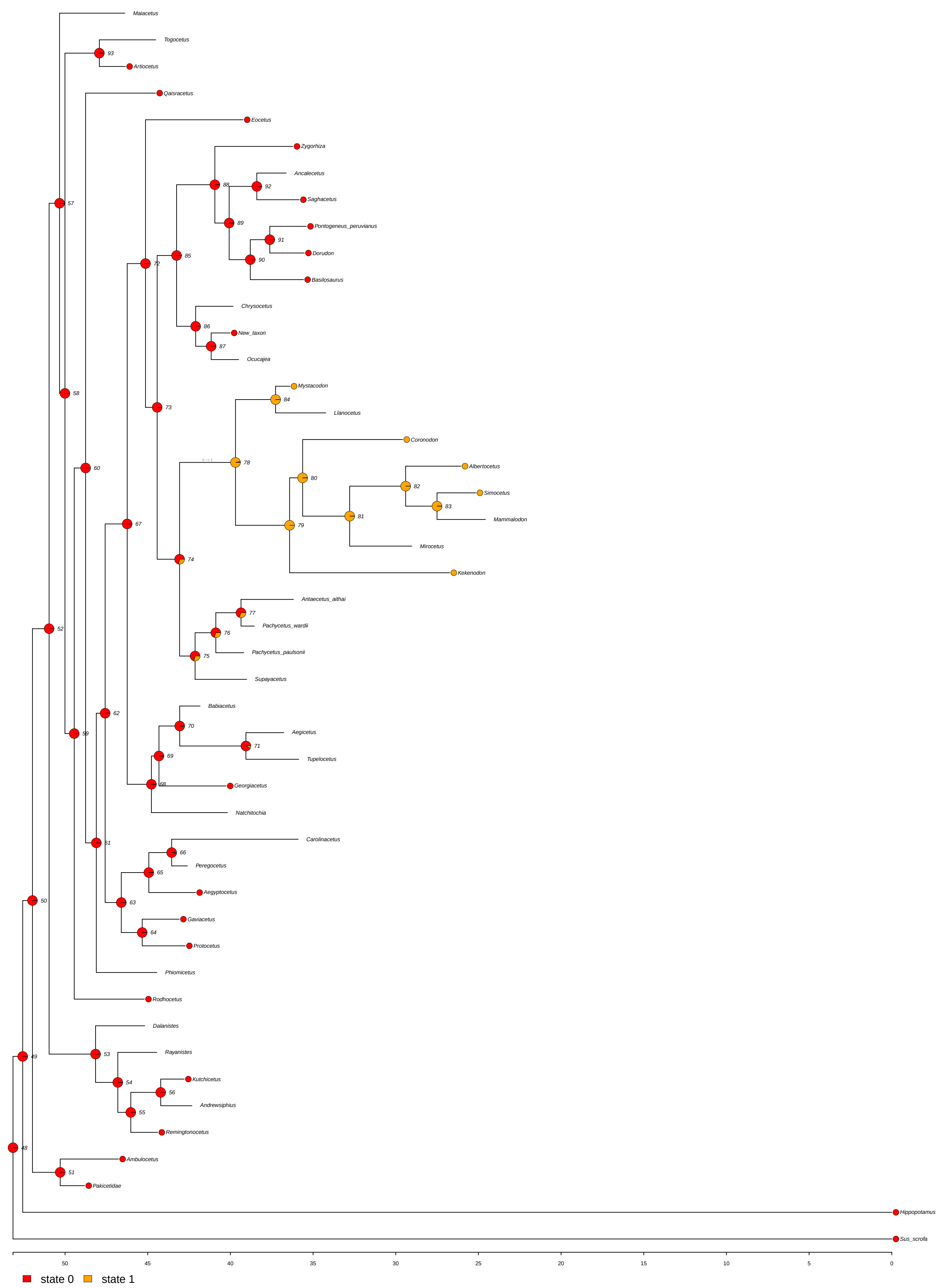
Maiacetus
Togocetus
93
Artiocetus
Qaisracetus
Eocetus
Zygorhiza
Ancalecetus
88
92
Saghacetus
89
Pontogeneus_peruvianus
91
Dorudon
90
Basilosaurus
57
72
85
Chrysocetus
86
New_taxon
87
Ocucajea
Mystacodon
84
Llanocetus
58
73
Coronodon
0 --> 1
78
60
80
Albertocetus
82
Simocetus
83
67
81
Mammalodon
79
Mirocetus
74
Kekenodon
Antaecetus_aithai
77
Pachycetus_wardii
52
76
Pachycetus_paulsonii
75
Supayacetus
Babiacetus
62
70
Aegicetus
59
71
Tupelocetus
69
68
Georgiacetus
Natchitochia
Carolinacetus
61
66
Peregocetus
65
Aegyptocetus
50
63
Gaviacetus
64
Protocetus
Phiomicetus
Rodhocetus
Dalanistes
Rayanistes
53
49
54
Kutchicetus
56
Andrewsiphius
55
Remingtonocetus
48
Ambulocetus
51
Pakicetidae
Hippopotamus
Sus_scrofa
50
45
40
35
30
25
20
15
10
5
0
state 0
state 1

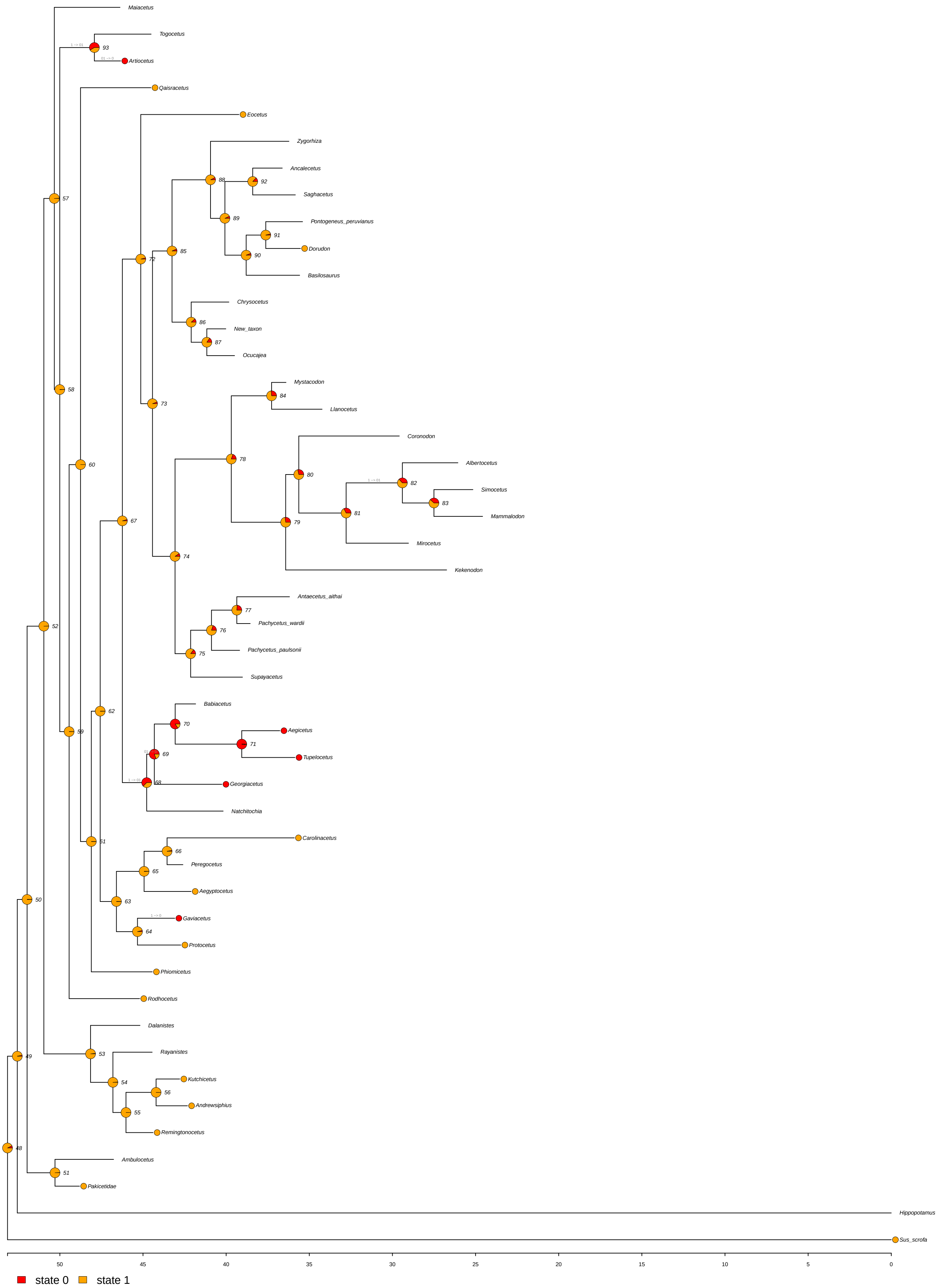
Maiacetus
Togocetus
Artiocetus
Qaisracetus
Eocetus
Zygorhiza
Ancalecetus
Saghacetus
Pontogeneus_peruvianus
Dorudon
Basilosaurus
Chrysocetus
New_taxon
Ocucajea
Mystacodon
Llanocetus
Coronodon
Albertocetus
Simocetus
Mammalodon
Mirocetus
Kekenodon
Antaecetus_aithai
Pachycetus_wardii
Pachycetus_paulsonii
Supayacetus
Babiacetus
Aegicetus
Tupelocetus
Georgiacetus
Natchitochia
Carolinacetus
Peregocetus
Aegyptocetus
Gaviacetus
Protocetus
Phiomicetus
Rodhocetus
Dalanistes
Rayanistes
Kutchicetus
Andrewsiphius
Remingtonocetus
Ambulocetus
Pakicetidae
Hippopotamus
Sus_scrofa
50
45
40
35
30
25
20
15
10
5
0
state 0
state 1

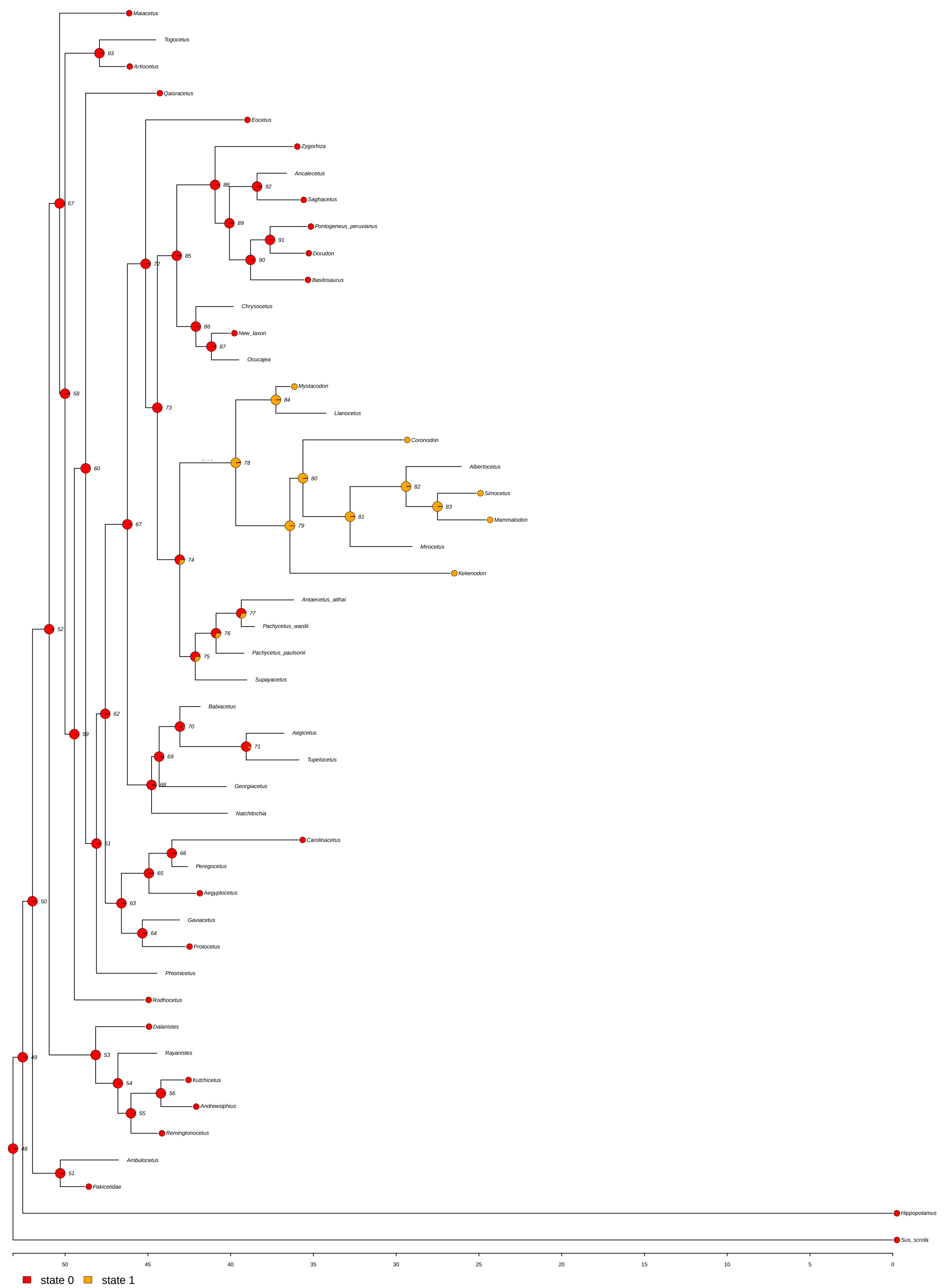
Maiacetus
Togocetus
Artiocetus
Qaisracetus
Eocetus
Zygorhiza
Ancalecetus
Saghacetus
Pontogeneus_peruvianus
Dorudon
Basilosaurus
Chrysocetus
New_taxon
Ocucajea
Mystacodon
Llanocetus
Coronodon
Albertocetus
Simocetus
Mammalodon
Mirocetus
Kekenodon
Antaecetus_aithai
Pachycetus_wardii
Pachycetus_paulsonii
Supayacetus
Babiacetus
Aegicetus
Tupelocetus
Georgiacetus
Natchitochia
Carolinacetus
Peregocetus
Aegyptocetus
Gaviacetus
Protocetus
Phiomicetus
Rodhocetus
Dalanistes
Rayanistes
Kutchicetus
Andrewsiphius
Remingtonocetus
Ambulocetus
Pakicetidae
Hippopotamus
Sus_scrofa
50
45
40
35
30
25
20
15
10
5
0
state 0
state 1

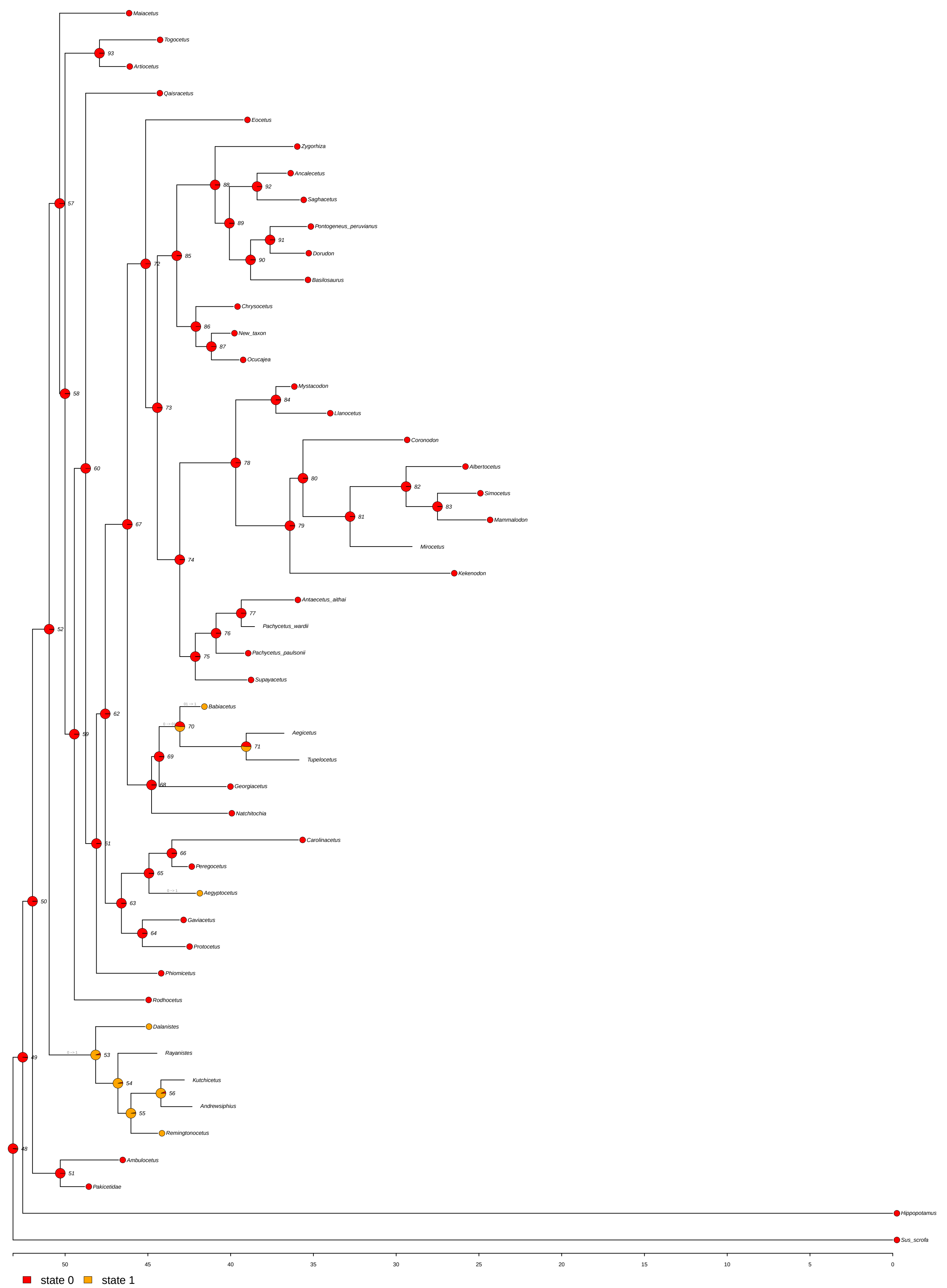

Maiacetus
Togocetus
93
Artiocetus
Qaisracetus
Eocetus
Zygorhiza
Ancalecetus
88
92
Saghacetus
89
Pontogeneus_peruvianus
91
Dorudon
90
Basilosaurus
57
72
85
Chrysocetus
86
New_taxon
87
Ocucajea
Mystacodon
84
Llanocetus
58
73
Coronodon
60
78
80
Albertocetus
82
Simocetus
83
Mammalodon
81
67
79
Mirocetus
74
Kekenodon
Antaecetus_aithai
77
Pachycetus_wardii
52
76
Pachycetus_paulsonii
75
Supayacetus
Babiacetus
62
70
Aegicetus
59
71
Tupelocetus
69
68
Georgiacetus
Natchitochia
Carolinacetus
66
61
Peregocetus
65
Aegyptocetus
50
63
Gaviacetus
64
Protocetus
Phiomicetus
Rodhocetus
Dalanistes
53
Rayanistes
49
54
Kutchicetus
56
55
Andrewsiphius
Remingtonocetus
48
Ambulocetus
51
Pakicetidae
Hippopotamus
Sus_scrofa
50
45
40
35
30
25
20
15
10
5
0
state 0
state 1

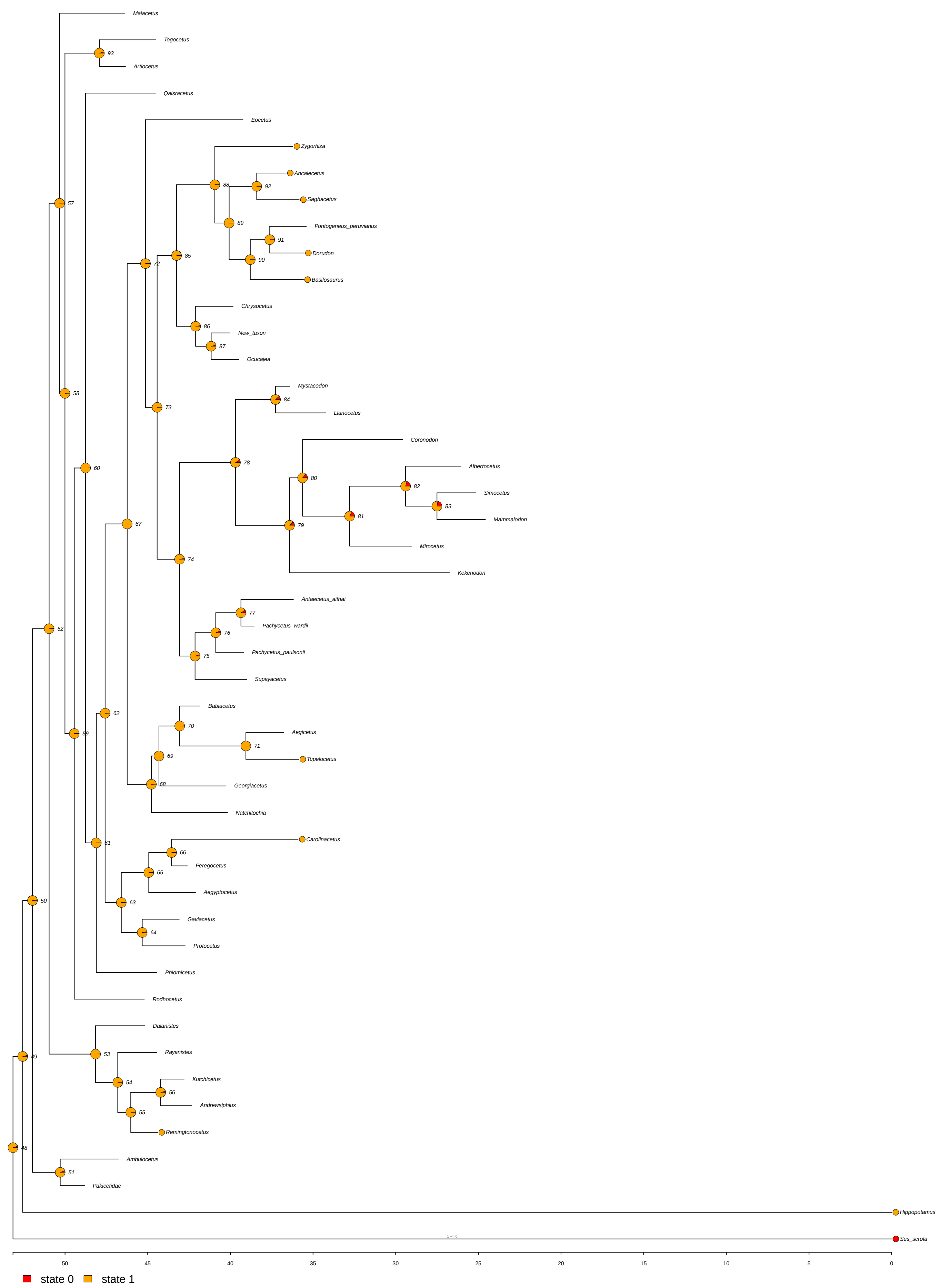
Maiacetus
Togocetus
93
Artiocetus
Qaisracetus
Eocetus
Zygorhiza
Ancalecetus
88
92
Saghacetus
89
Pontogeneus_peruvianus
91
Dorudon
90
Basilosaurus
72
85
Chrysocetus
86
New_taxon
87
Ocucajea
Mystacodon
84
Llanocetus
58
73
Coronodon
78
Albertocetus
80
82
Simocetus
83
81
Mammalodon
79
Mirocetus
Kekenodon
74
Antaecetus_aithai
77
Pachycetus_wardii
76
Pachycetus_paulsonii
75
Supayacetus
57
60
67
52
Babiacetus
70
Aegicetus
71
Tupelocetus
62
59
69
68
Georgiacetus
Natchitochia
Carolinacetus
66
Peregocetus
65
Aegyptocetus
61
63
Gaviacetus
64
Protocetus
Phiomicetus
Rodhocetus
50
Dalanistes
Rayanistes
53
Kutchicetus
54
56
Andrewsiphius
55
Remingtonocetus
49
48
Ambulocetus
51
Pakicetidae
Hippopotamus
Sus_scrofa
50
45
40
35
30
25
20
15
10
5
0
state 0
state 1

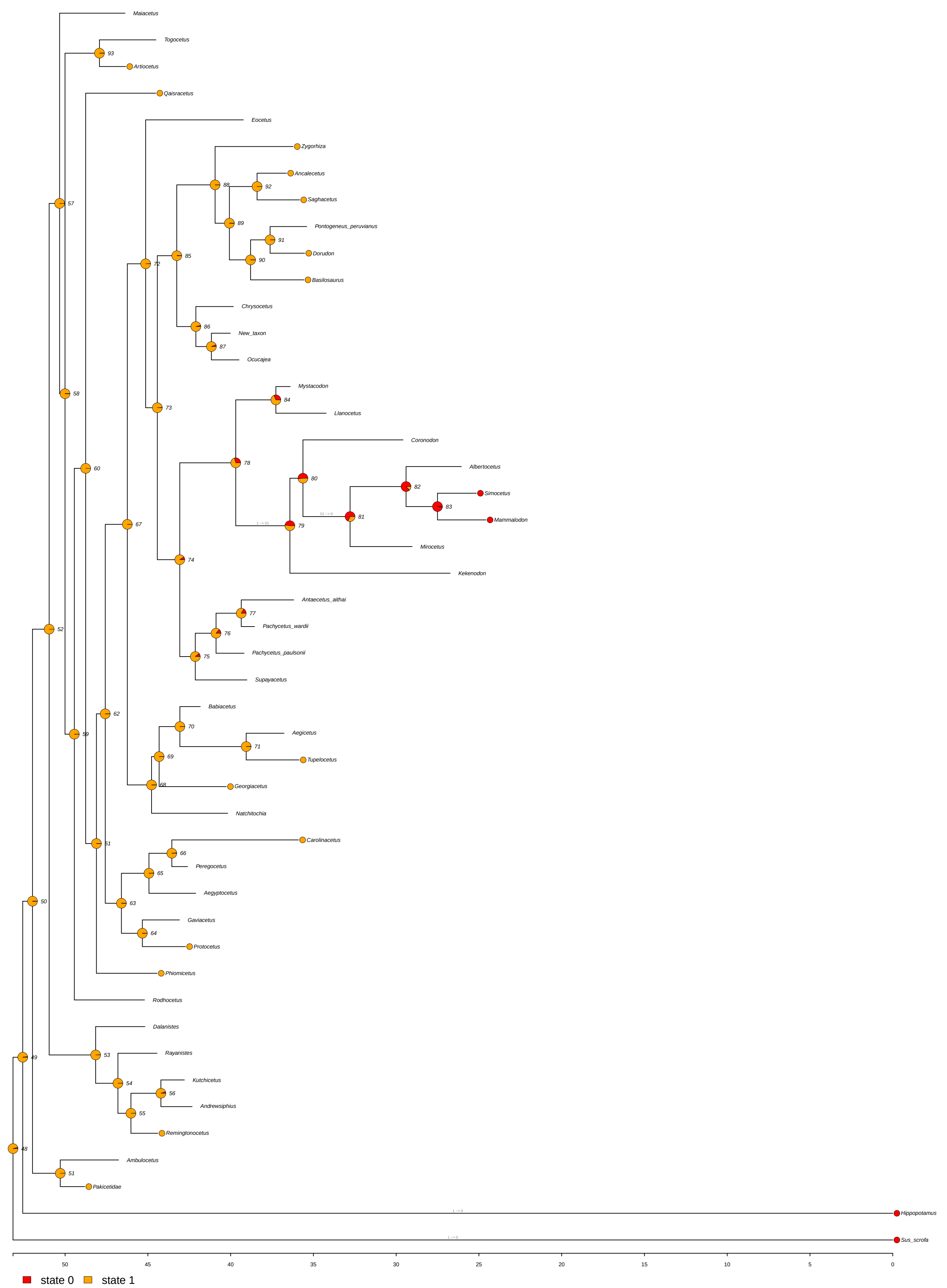

Maiacetus
Togocetus
93
Artiocetus
Qaisracetus
Eocetus
Zygorhiza
Ancalecetus
88
92
Saghacetus
89
Pontogeneus_peruvianus
91
Dorudon
90
Basilosaurus
72
85
Chrysocetus
86
New_taxon
87
Ocucajea
57
58
Mystacodon
84
Llanocetus
73
Coronodon
78
80
Albertocetus
82
Simocetus
83
Mammalodon
81
79
Mirocetus
Kekenodon
74
60
67
Antaecetus_aithai
77
Pachycetus_wardii
76
Pachycetus_paulsonii
75
Supayacetus
52
Babiacetus
62
70
Aegicetus
59
71
Tupelocetus
69
68
Georgiacetus
Natchitochia
Carolinacetus
61
66
Peregocetus
65
Aegyptocetus
63
Gaviacetus
64
Protocetus
Phiomicetus
Rodhocetus
Dalanistes
53
Rayanistes
54
Kutchicetus
56
Andrewsiphius
55
Remingtonocetus
49
50
48
Ambulocetus
51
Pakicetidae
Hippopotamus
Sus_scrofa
50
45
40
35
30
25
20
15
10
5
0
state 0
state 1

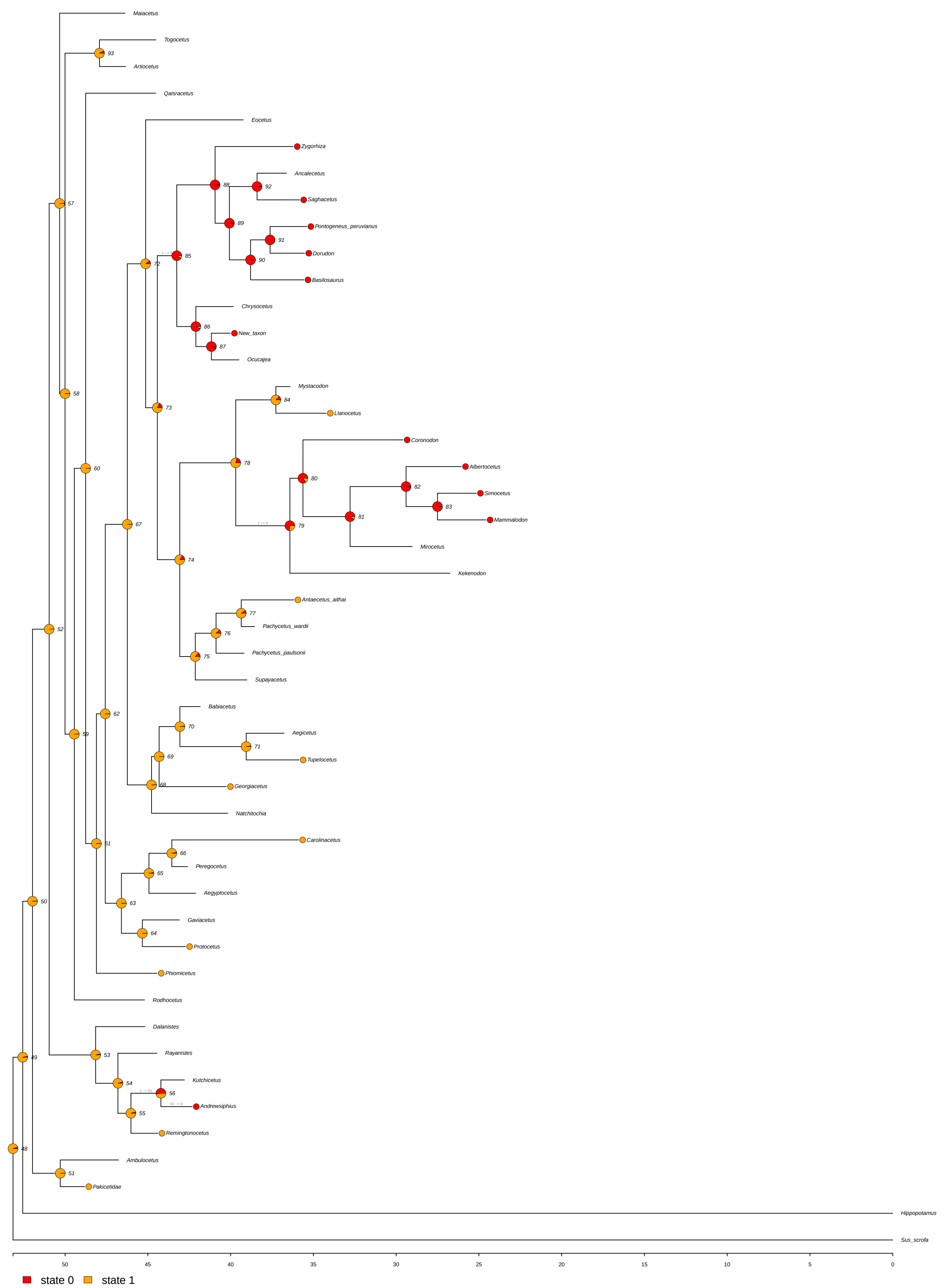
Maiacetus
Togocetus
93
Artiocetus
Qaisracetus
Eocetus
Zygorhiza
Ancalecetus
88
92
Saghacetus
57
89
Pontogeneus_peruvianus
91
1 --> 0
85
Dorudon
72
90
Basilosaurus
Chrysocetus
86
New_taxon
87
Ocucajea
Mystacodon
84
58
73
Llanocetus
Coronodon
60
78
Albertocetus
80
82
Simocetus
83
81
Mammalodon
67
1 --> 0
79
Mirocetus
74
Kekenodon
Antaecetus_aithai
77
52
76
Pachycetus_wardii
Pachycetus_paulsonii
75
Supayacetus
Babiacetus
62
70
Aegicetus
59
71
69
Tupelocetus
68
Georgiacetus
Natchitochia
Carolinacetus
61
66
Peregocetus
65
Aegyptocetus
50
63
Gaviacetus
64
Protocetus
Phiomicetus
Rodhocetus
Dalanistes
Rayanistes
49
53
Kutchicetus
54
1 --> 01
56
01 --> 0
Andrewsiphius
55
Remingtonocetus
48
Ambulocetus
51
Pakicetidae
Hippopotamus
Sus_scrofa
50
45
40
35
30
25
20
15
10
5
0
state 0
state 1

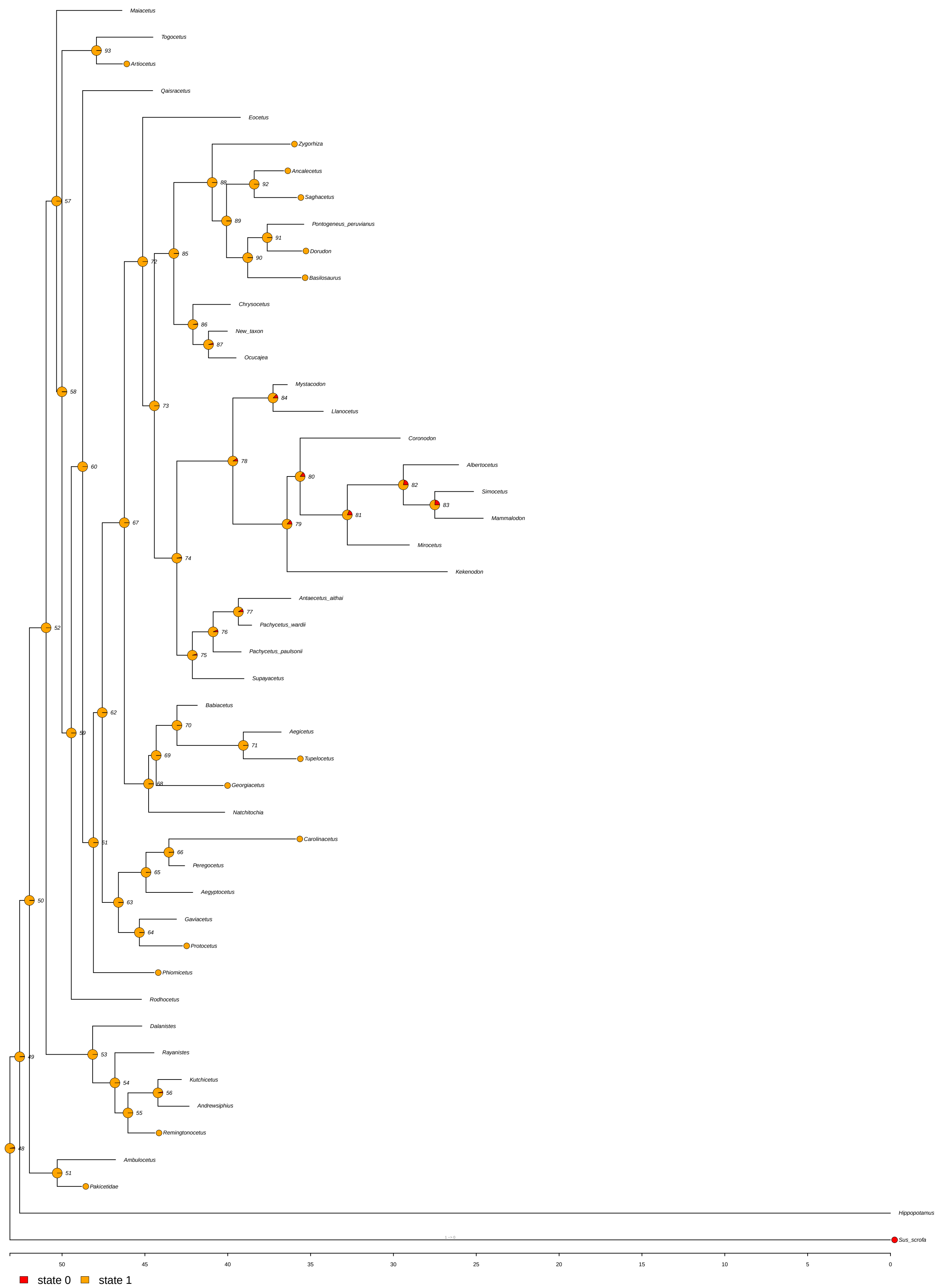
Maiacetus
Togocetus
Artiocetus
Qaisracetus
Eocetus
Zygorhiza
Ancalecetus
Saghacetus
Pontogeneus_peruvianus
Dorudon
Basilosaurus
Chrysocetus
New_taxon
Ocucajea
Mystacodon
Llanocetus
Coronodon
Albertocetus
Simocetus
Mammalodon
Mirocetus
Kekenodon
Antaecetus_aithai
Pachycetus_wardii
Pachycetus_paulsonii
Supayacetus
Babiacetus
Aegicetus
Tupelocetus
Georgiacetus
Natchitochia
Carolinacetus
Peregocetus
Aegyptocetus
Gaviacetus
Protocetus
Phiomicetus
Rodhocetus
Dalanistes
Rayanistes
Kutchicetus
Andrewsiphius
Remingtonocetus
Ambulocetus
Pakicetidae
Hippopotamus
Sus_scrofa
50
45
40
35
30
25
20
15
10
5
0
state 0
state 1

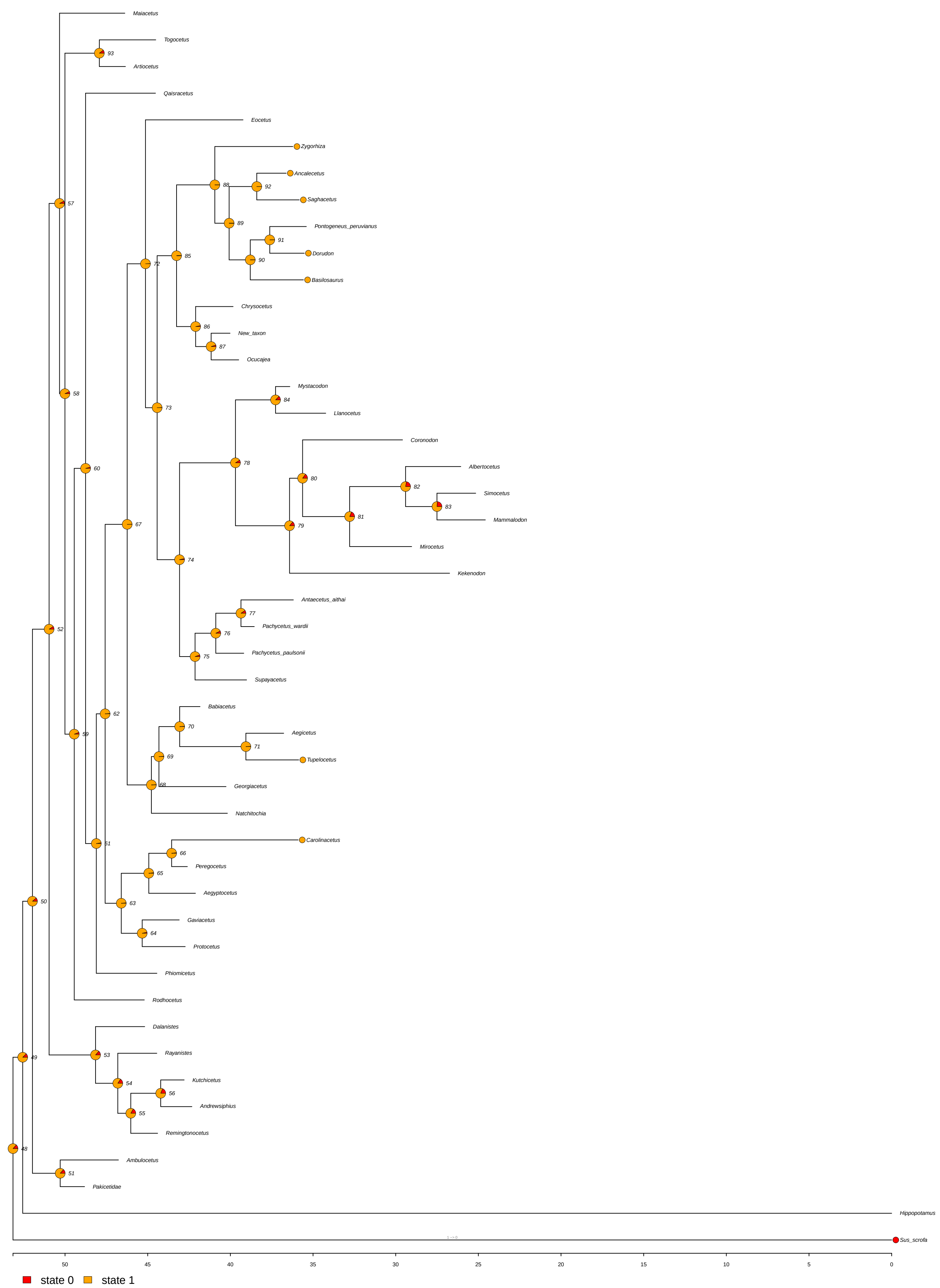
Maiacetus
Togocetus
Artiocetus
Qaisracetus
Eocetus
Zygorhiza
Ancalecetus
Saghacetus
Pontogeneus_peruvianus
Dorudon
Basilosaurus
Chrysocetus
New_taxon
Ocucajea
Mystacodon
Llanocetus
Coronodon
Albertocetus
Simocetus
Mammalodon
Mirocetus
Kekenodon
Antaecetus_aithai
Pachycetus_wardii
Pachycetus_paulsonii
Supayacetus
Babiacetus
Aegicetus
Tupelocetus
Georgiacetus
Natchitochia
Carolinacetus
Peregocetus
Aegyptocetus
Gaviacetus
Protocetus
Phiomicetus
Rodhocetus
Dalanistes
Rayanistes
Kutchicetus
Andrewsiphius
Remingtonocetus
Ambulocetus
Pakicetidae
Hippopotamus
Sus_scrofa
50
45
40
35
30
25
20
15
10
5
0
state 0
state 1

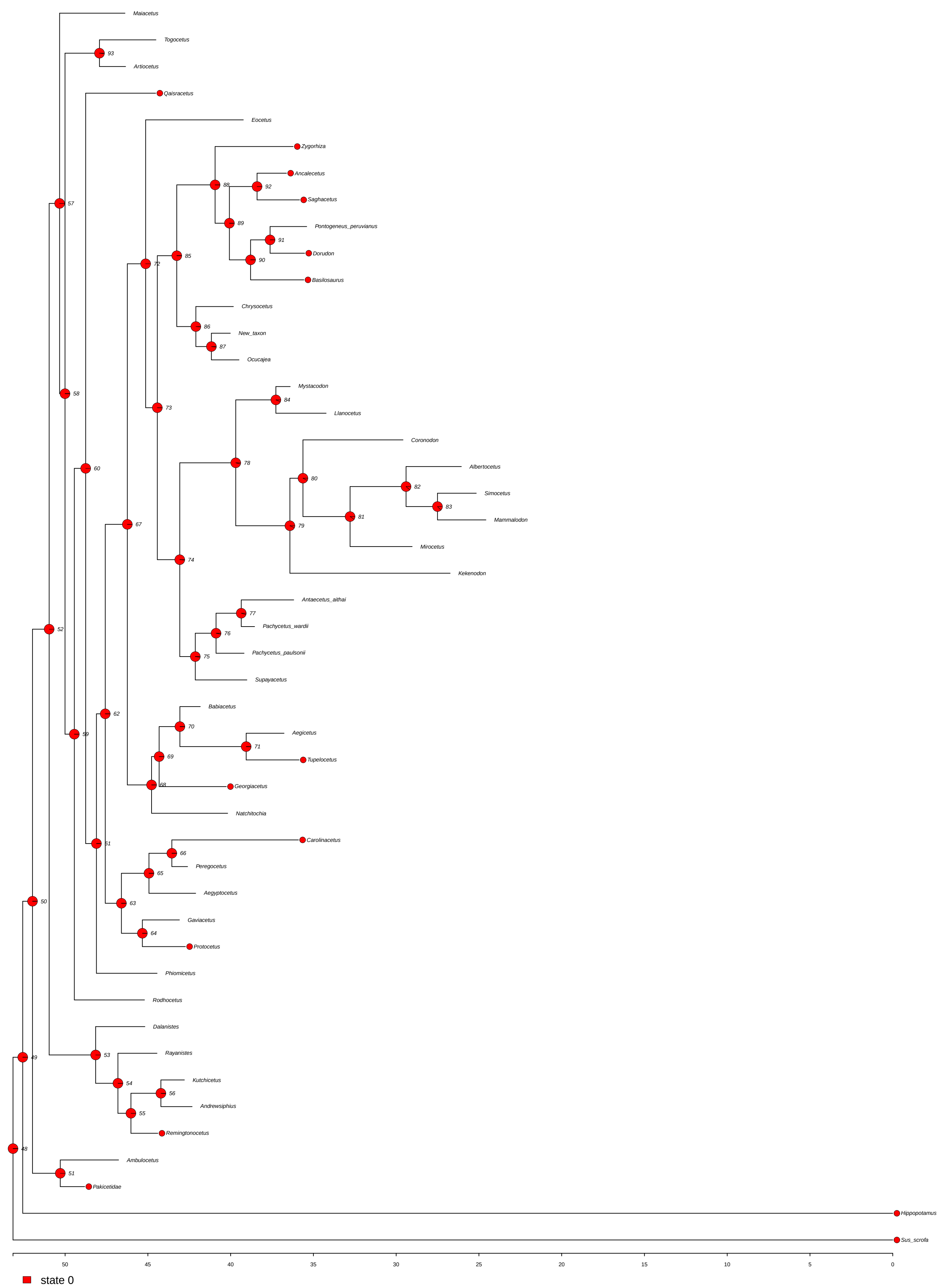

Maiacetus
Togocetus
Artiocetus
Qaisracetus
Eocetus
Zygorhiza
Ancalecetus
Saghacetus
Pontogeneus_peruvianus
Dorudon
Basilosaurus
Chrysocetus
New_taxon
Ocucajea
Mystacodon
Llanocetus
Coronodon
Albertocetus
Simocetus
Mammalodon
Mirocetus
Kekenodon
Antaecetus_aithai
Pachycetus_wardii
Pachycetus_paulsonii
Supayacetus
Babiacetus
Aegicetus
Tupelocetus
Georgiacetus
Natchitochia
Carolinacetus
Peregocetus
Aegyptocetus
Gaviacetus
Protocetus
Phiomicetus
Rodhocetus
Dalanistes
Rayanistes
Kutchicetus
Andrewsiphius
Remingtonocetus
Ambulocetus
Pakicetidae
Hippopotamus
Sus_scrofa
93
57
88
92
89
91
90
72
85
86
87
58
73
84
78
80
82
83
81
79
60
67
74
77
76
75
52
62
59
70
71
69
68
61
66
65
63
64
50
53
54
56
55
49
48
51
50
45
40
35
30
25
20
15
10
5
0
state 0

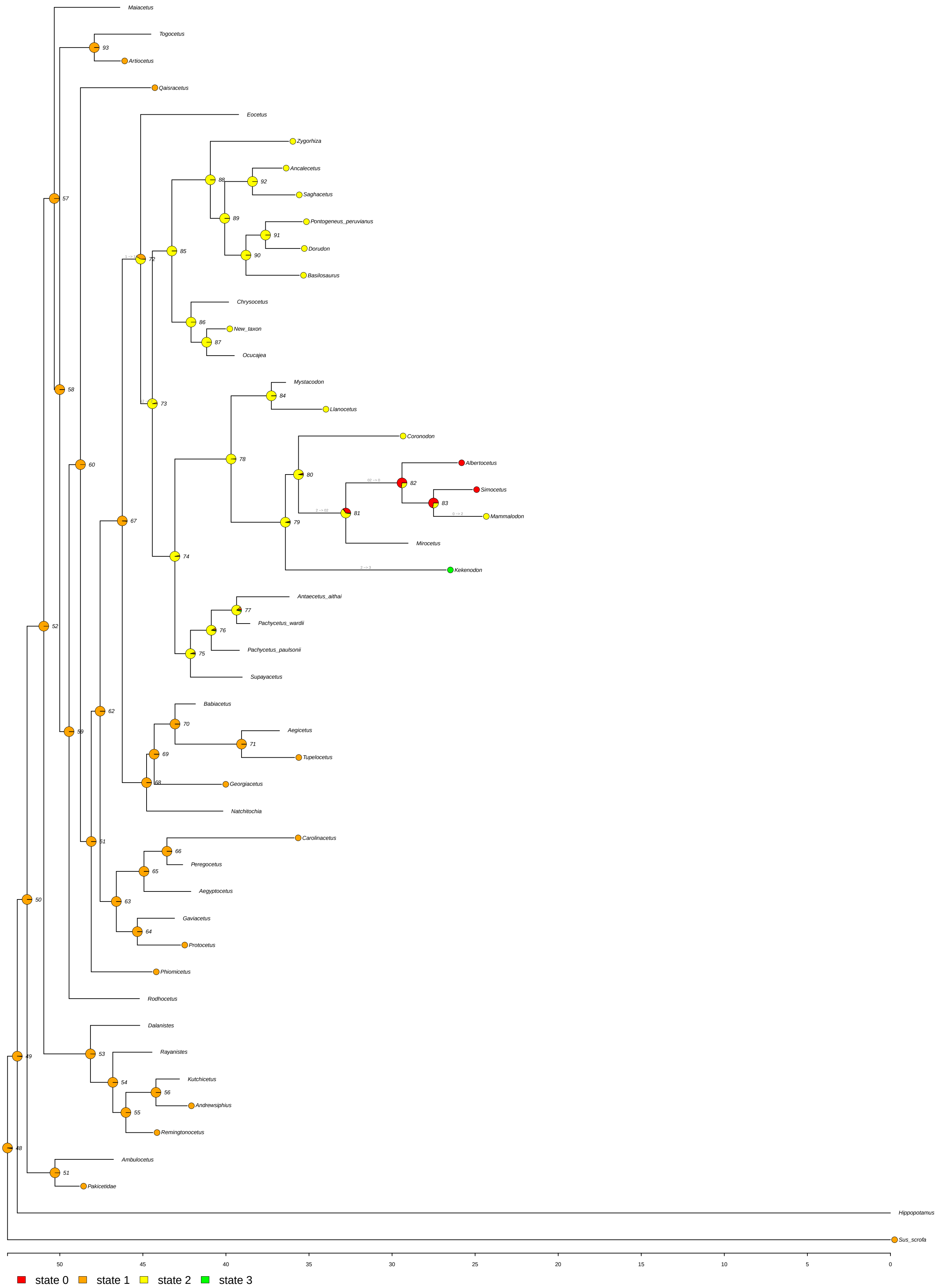

Maiacetus
Togocetus
93
Artiocetus
Qaisracetus
Eocetus
Zygorhiza
Ancalecetus
88
92
Saghacetus
Pontogeneus_peruvianus
89
91
Dorudon
90
Basilosaurus
72
85
Chrysocetus
86
New_taxon
87
Ocucajea
Mystacodon
84
58
Llanocetus
73
Coronodon
60
78
Albertocetus
80
02 -> 0
82
Simocetus
2 -> 02
83
Mammalodon
67
81
0 -> 2
79
Mirocetus
74
2 -> 3
Kekenodon
Antaecetus_aithai
77
Pachycetus_wardii
52
76
Pachycetus_paulsonii
75
Supayacetus
Babiacetus
62
70
Aegicetus
59
71
69
Tupelocetus
68
Georgiacetus
Natchitochia
Carolinacetus
61
66
Peregocetus
65
Aegyptocetus
50
63
Gaviacetus
64
Protocetus
Phiomicetus
Rodhocetus
Dalanistes
53
Rayanistes
49
54
Kutchicetus
56
Andrewsiphius
55
Remingtonocetus
48
Ambulocetus
51
Pakicetidae
Hippopotamus
Sus_scrofa
50
45
40
35
30
25
20
15
10
5
0
state 0
state 1
state 2
state 3

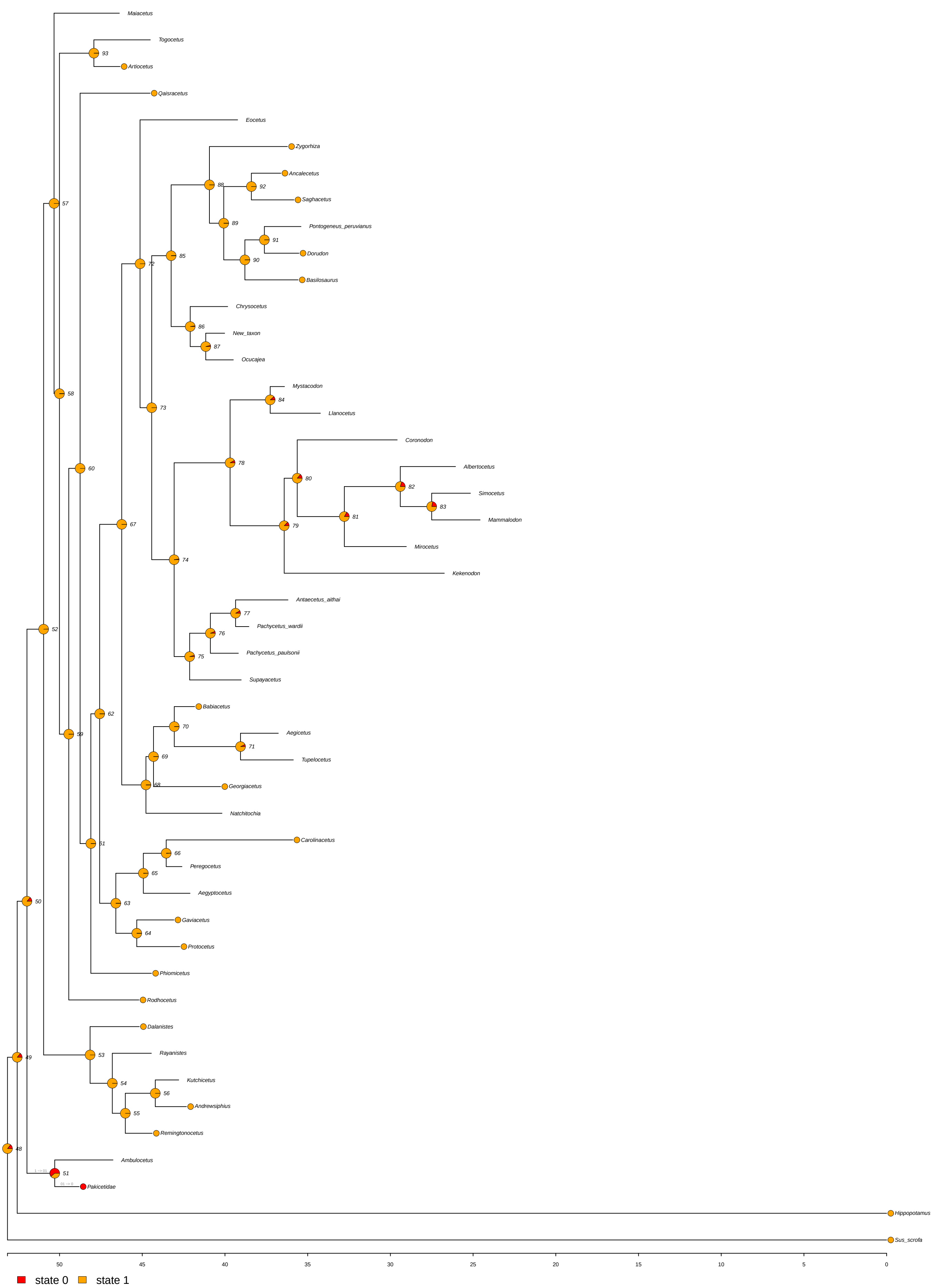

Maiacetus
Togocetus
Artiocetus
Qaisracetus
Eocetus
Zygorhiza
Ancalecetus
Saghacetus
Pontogeneus_peruvianus
Dorudon
Basilosaurus
Chrysocetus
New_taxon
Ocucajea
Mystacodon
Llanocetus
Coronodon
Albertocetus
Simocetus
Mammalodon
Mirocetus
Kekenodon
Antaecetus_aithai
Pachycetus_wardii
Pachycetus_paulsonii
Supayacetus
Babiacetus
Aegicetus
Tupelocetus
Georgiacetus
Natchitochia
Carolinacetus
Peregocetus
Aegyptocetus
Gaviacetus
Protocetus
Phiomicetus
Rodhocetus
Dalanistes
Rayanistes
Kutchicetus
Andrewsiphius
Remingtonocetus
Ambulocetus
Pakicetidae
Hippopotamus
Sus_scrofa
50
45
40
35
30
25
20
15
10
5
0
state 0
state 1

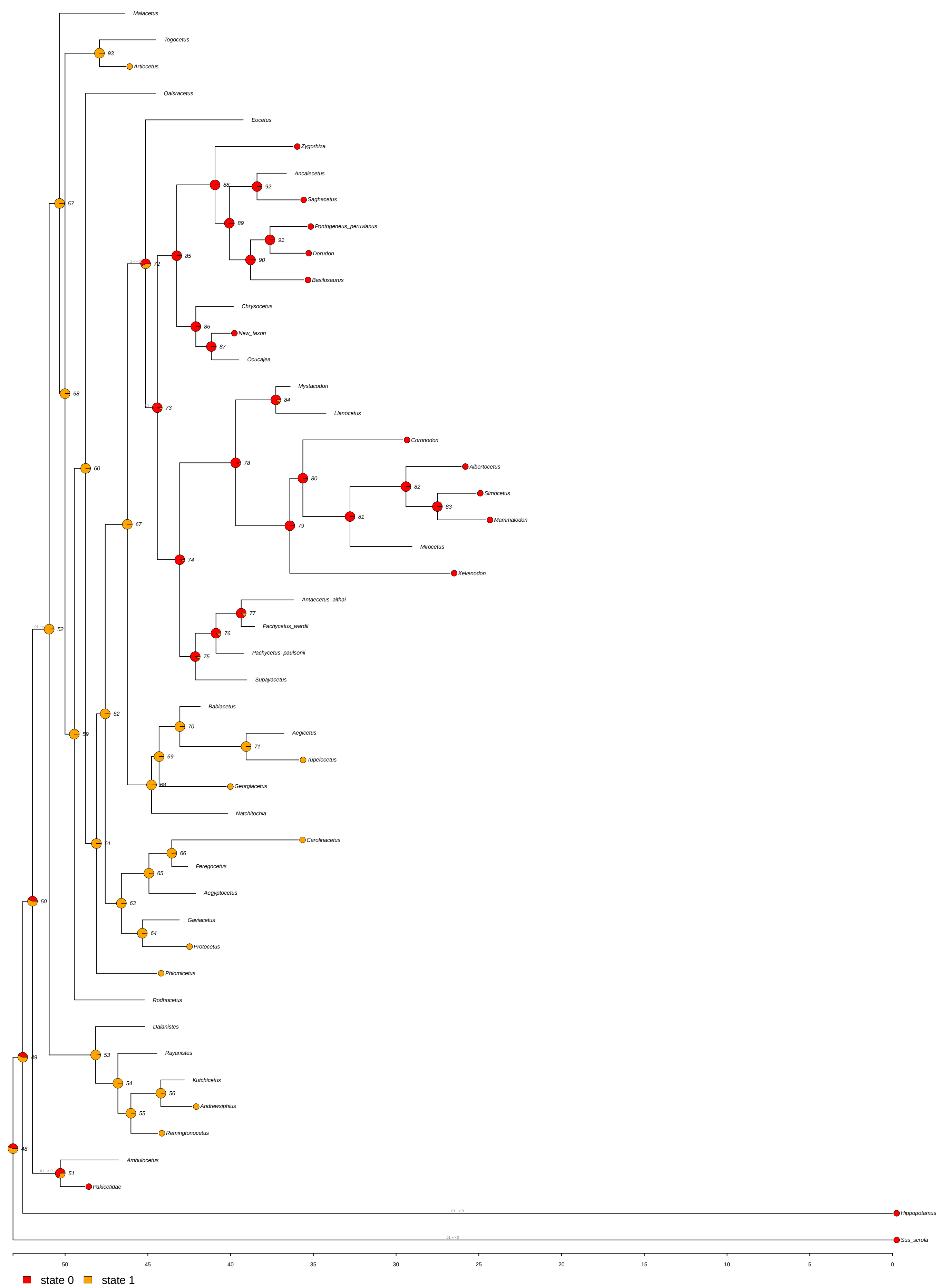
Maiacetus
Togocetus
Artiocetus
Qaisracetus
Eocetus
Zygorhiza
Ancalecetus
Saghacetus
Pontogeneus_peruvianus
Dorudon
Basilosaurus
Chrysocetus
New_taxon
Ocucajea
Mystacodon
Llanocetus
Coronodon
Albertocetus
Simocetus
Mammalodon
Mirocetus
Kekenodon
Antaecetus_aithai
Pachycetus_wardii
Pachycetus_paulsonii
Supayacetus
Babiacetus
Aegicetus
Tupelocetus
Georgiacetus
Natchitochia
Carolinacetus
Peregocetus
Aegyptocetus
Gaviacetus
Protocetus
Phiomicetus
Rodhocetus
Dalanistes
Rayanistes
Kutchicetus
Andrewsiphius
Remingtonocetus
Ambulocetus
Pakicetidae
Hippopotamus
Sus_scrofa
50
45
40
35
30
25
20
15
10
5
0
state 0
state 1

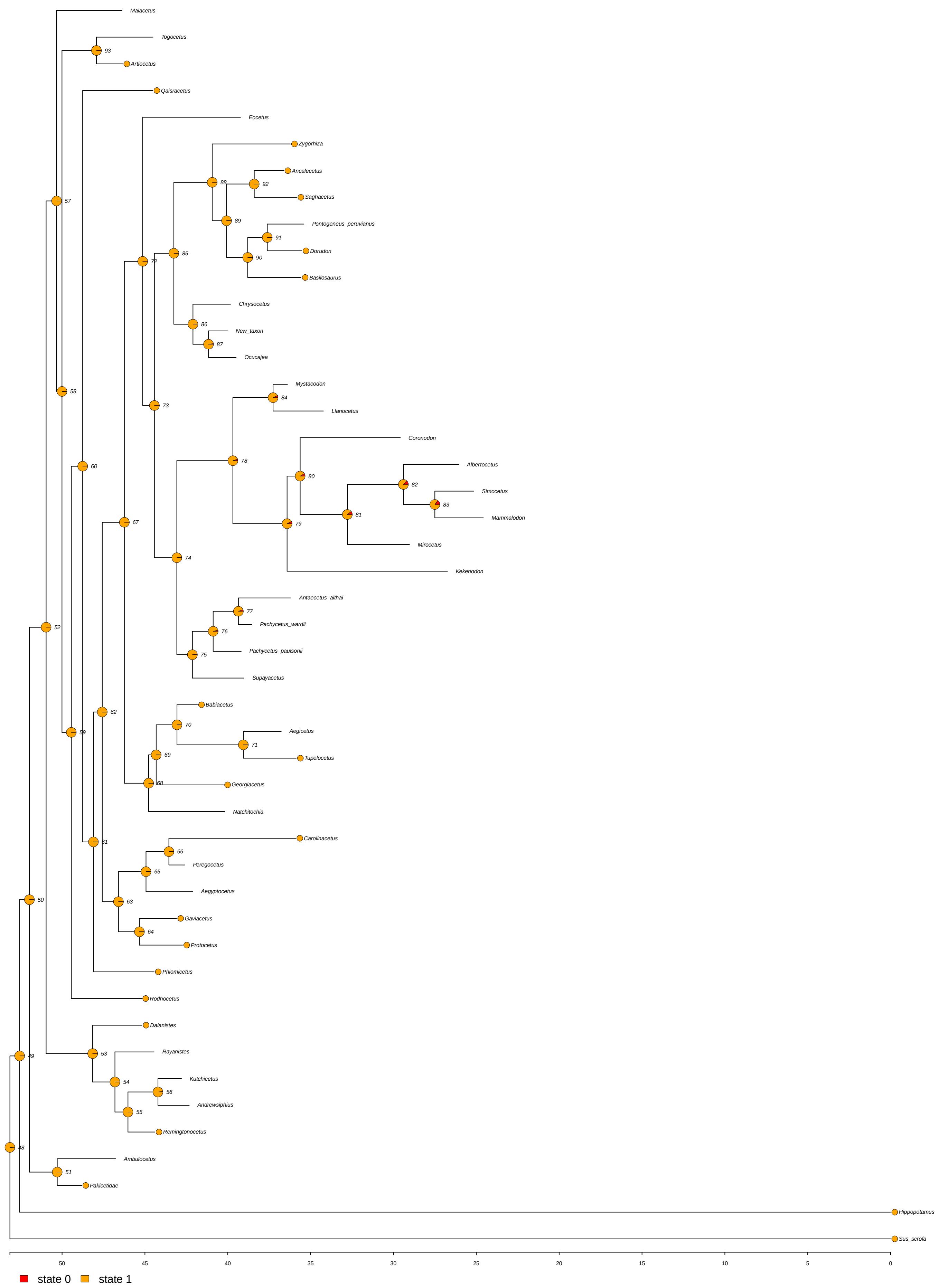

Maiacetus
Togocetus
93
Artiocetus
Qaisracetus
Eocetus
Zygorhiza
Ancalecetus
88
92
Saghacetus
Pontogeneus_peruvianus
89
91
Dorudon
90
Basilosaurus
57
72
85
Chrysocetus
86
New_taxon
87
Ocucajea
Mystacodon
84
58
73
Llanocetus
Coronodon
60
78
Albertocetus
80
82
Simocetus
83
Mammalodon
81
67
79
Mirocetus
74
Kekenodon
Antaecetus_aithai
77
76
Pachycetus_wardii
52
Pachycetus_paulsonii
75
Supayacetus
Babiacetus
62
70
Aegicetus
59
71
69
Tupelocetus
68
Georgiacetus
Natchitochia
Carolinacetus
61
66
Peregocetus
65
Aegyptocetus
50
63
Gaviacetus
64
Protocetus
Phiomicetus
Rodhocetus
Dalanistes
49
53
Rayanistes
54
Kutchicetus
56
Andrewsiphius
55
Remingtonocetus
48
Ambulocetus
51
Pakicetidae
Hippopotamus
Sus_scrofa
50
45
40
35
30
25
20
15
10
5
0
state 0
state 1

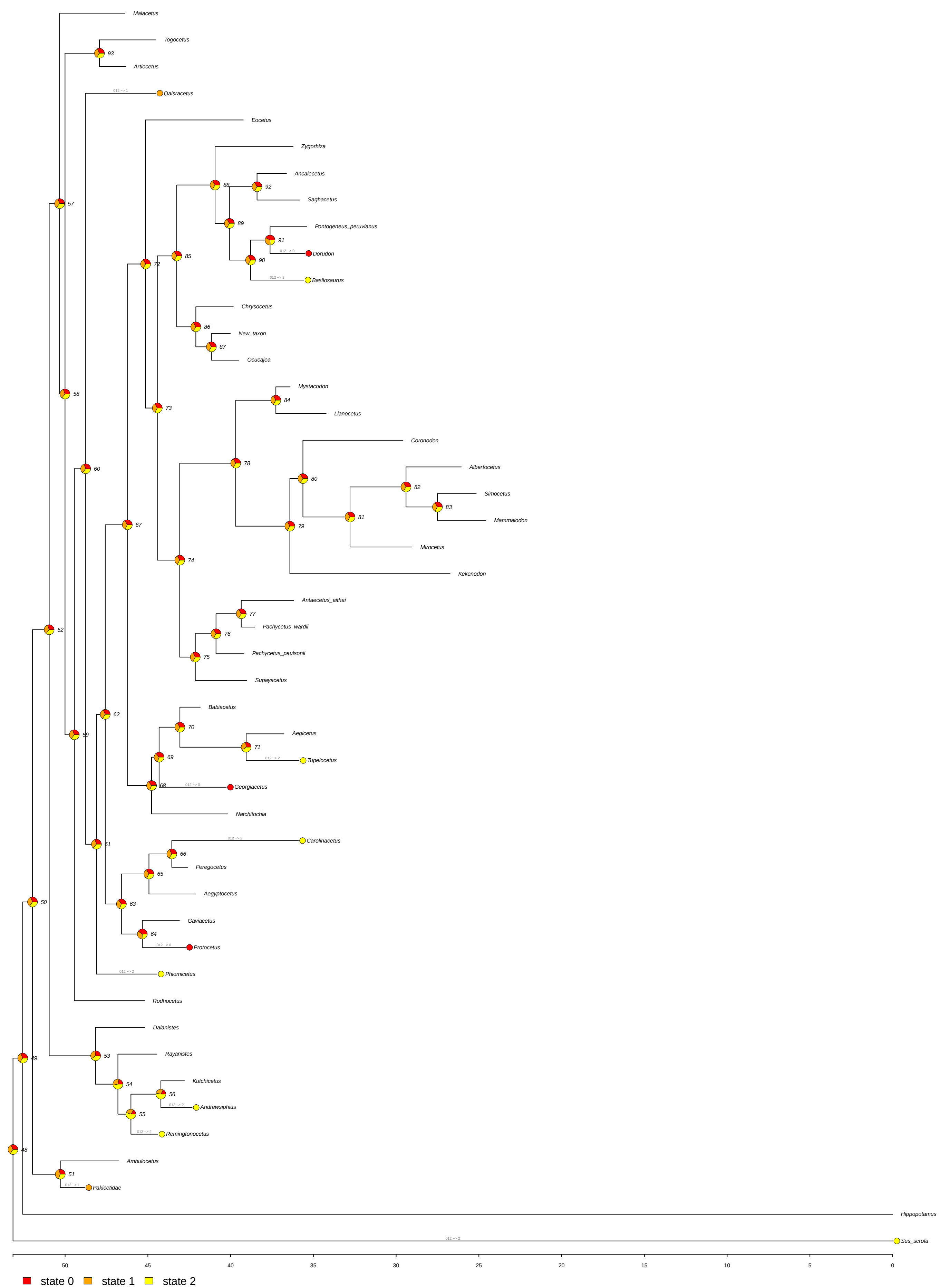

Maiacetus
Togocetus
93
Artiocetus
Qaisracetus
Eocetus
Zygorhiza
Ancalecetus
88
92
Saghacetus
57
89
Pontogeneus_peruvianus
91
Dorudon
85
90
72
Basilosaurus
Chrysocetus
86
New_taxon
87
Ocucajea
Mystacodon
84
58
73
Llanocetus
Coronodon
60
78
Albertocetus
80
82
Simocetus
83
Mammalodon
67
79
81
Mirocetus
74
Kekenodon
Antaecetus_aithai
77
Pachycetus_wardii
52
76
Pachycetus_paulsonii
75
Supayacetus
Babiacetus
62
59
70
Aegicetus
71
69
Tupelocetus
68
Georgiacetus
Natchitochia
61
Carolinacetus
66
Peregocetus
65
Aegyptocetus
50
63
Gaviacetus
64
Protocetus
Phiomicetus
Rodhocetus
Dalanistes
53
Rayanistes
49
54
Kutchicetus
56
Andrewsiphius
55
Remingtonocetus
48
Ambulocetus
51
Pakicetidae
Hippopotamus
Sus_scrofa
50
45
40
35
30
25
20
15
10
5
0
state 0
state 1
state 2

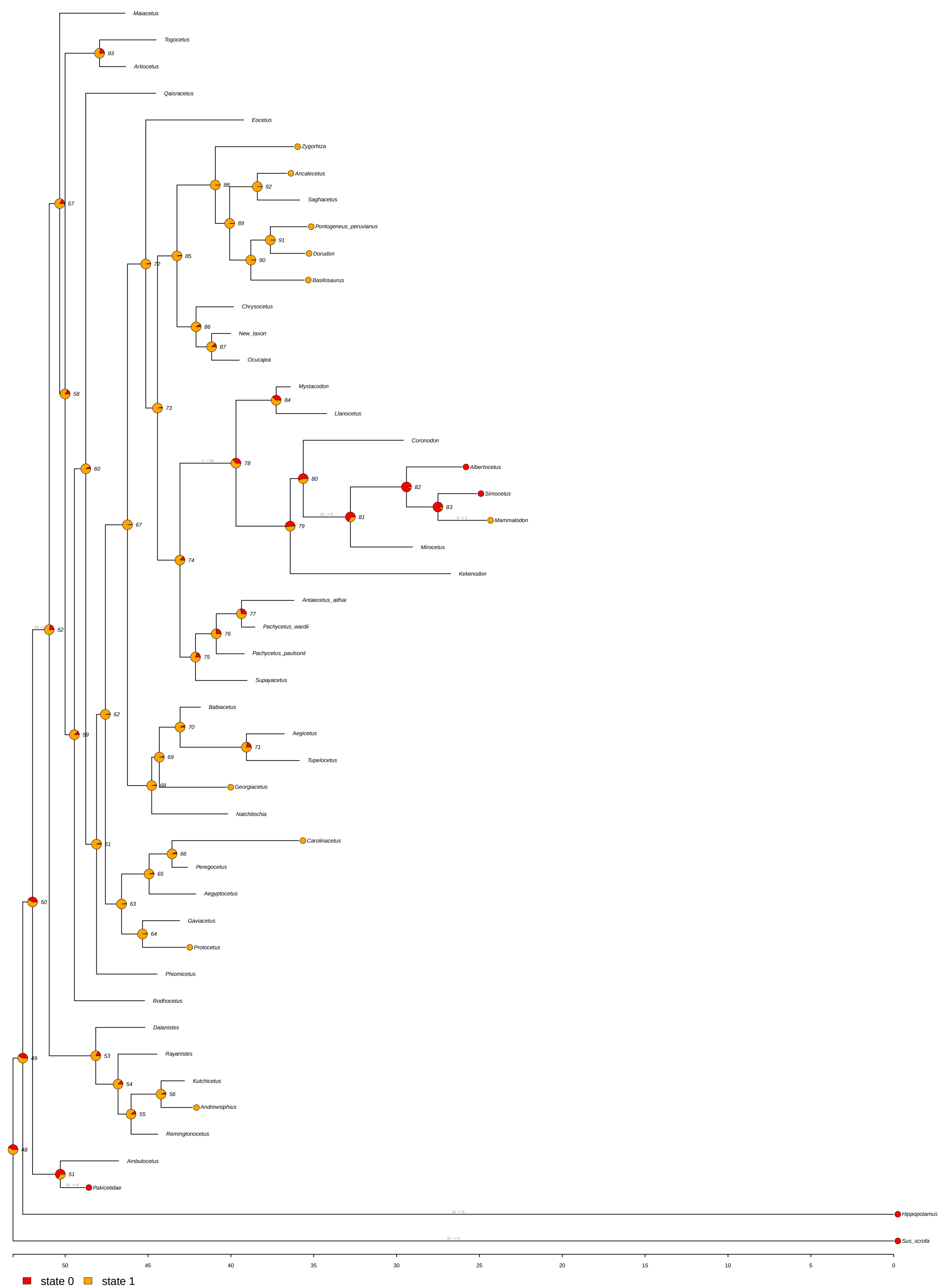

Maiacetus
Togocetus
93
Artiocetus
Qaisracetus
Eocetus
Zygorhiza
Ancalecetus
88
92
Saghacetus
Pontogeneus_peruvianus
89
91
Dorudon
90
Basilosaurus
72
85
57
Chrysocetus
86
New_taxon
87
Ocucajea
Mystacodon
84
58
Llanocetus
73
Coronodon
60
1 -> 01
78
Albertocetus
80
82
Simocetus
83
01 -> 0
81
0 -> 1
Mammalodon
67
79
Mirocetus
74
Kekenodon
Antaecetus_aithai
77
52
76
Pachycetus_wardii
Pachycetus_paulsonii
75
Supayacetus
Babiacetus
62
70
Aegicetus
59
71
Tupelocetus
69
68
Georgiacetus
Natchitochia
Carolinacetus
61
66
Peregocetus
65
50
Aegyptocetus
63
Gaviacetus
64
Protocetus
Phiomicetus
Rodhocetus
Dalanistes
53
Rayanistes
49
54
Kutchicetus
56
55
Andrewsiphius
Remingtonocetus
48
Ambulocetus
51
01 -> 0
Pakicetidae
01 -> 0
Hippopotamus
01 -> 0
Sus_scrofa
50
45
40
35
30
25
20
15
10
5
0
state 0
state 1

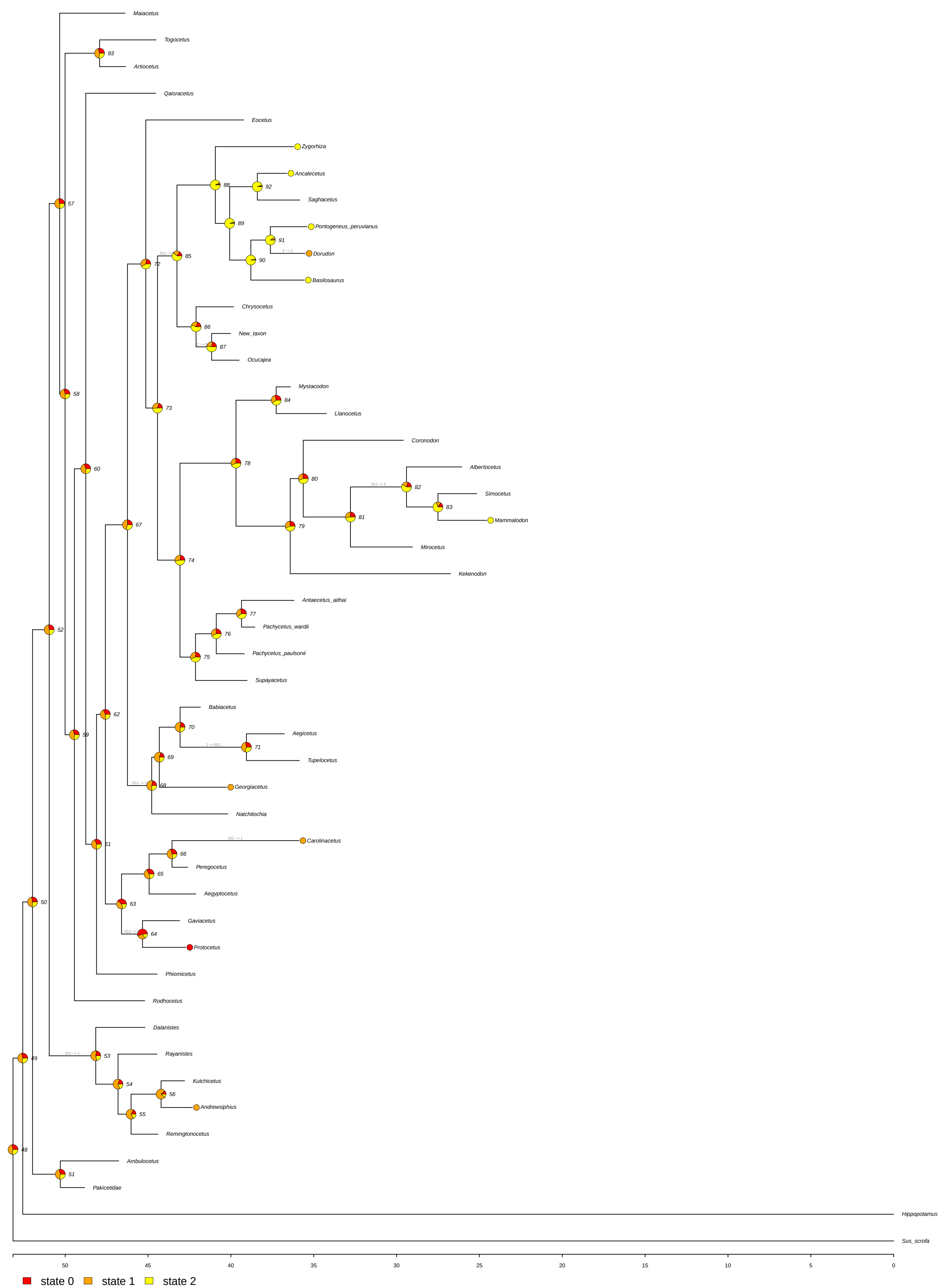
Maiacetus
Togocetus
93
Artiocetus
Qaisracetus
Eocetus
Zygorhiza
Ancalecetus
88
92
Saghacetus
89
Pontogeneus_peruvianus
91
2 -> 1
Dorudon
72
012 ->
85
90
Basilosaurus
Chrysocetus
86
New_taxon
87
Ocucajea
57
Mystacodon
84
Llanocetus
58
73
Coronodon
60
78
Albertocetus
80
012 -> 2
82
Simocetus
83
Mammalodon
81
67
79
Mirocetus
74
Kekenodon
Antaecetus_aithai
77
Pachycetus_wardii
76
52
Pachycetus_paulsonii
75
Supayacetus
Babiacetus
62
70
Aegicetus
1 -> 012
71
59
Tupelocetus
69
012 ->
68
Georgiacetus
Natchitochia
012 -> 1
Carolinacetus
61
66
Peregocetus
65
Aegyptocetus
50
63
Gaviacetus
012 ->
64
Protocetus
Phiomicetus
Rodhocetus
Dalanistes
012 -> 1
53
49
Rayanistes
54
Kutchicetus
56
55
Andrewsiphius
Remingtonocetus
48
Ambulocetus
51
Pakicetidae
Hippopotamus
Sus_scrofa
50
45
40
35
30
25
20
15
10
5
0
state 0
state 1
state 2

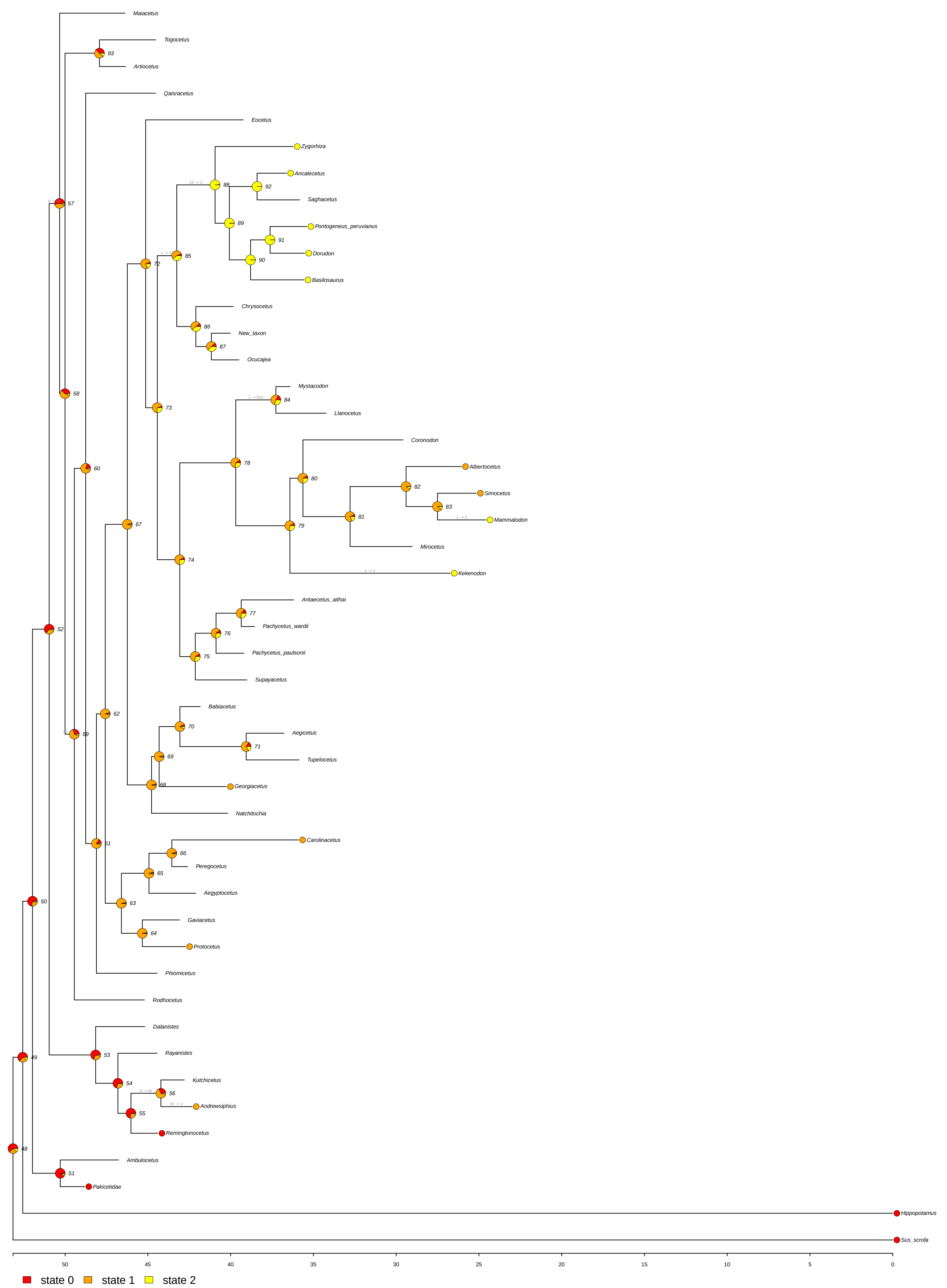

Maiacetus
Togocetus
Artiocetus
Qaisracetus
Eocetus
Zygorhiza
Ancalecetus
Saghacetus
Pontogeneus_peruvianus
Dorudon
Basilosaurus
Chrysocetus
New_taxon
Ocucajea
Mystacodon
Llanocetus
Coronodon
Albertocetus
Simocetus
Mammalodon
Mirocetus
Kekenodon
Antaecetus_aithai
Pachycetus_wardii
Pachycetus_paulsonii
Supayacetus
Babiacetus
Aegicetus
Tupelocetus
Georgiacetus
Natchitochia
Carolinacetus
Peregocetus
Aegyptocetus
Gaviacetus
Protocetus
Phiomicetus
Rodhocetus
Dalanistes
Rayanistes
Kutchicetus
Andrewsiphius
Remingtonocetus
Ambulocetus
Pakicetidae
Hippopotamus
Sus_scrofa
50
45
40
35
30
25
20
15
10
5
0
state 0
state 1
state 2

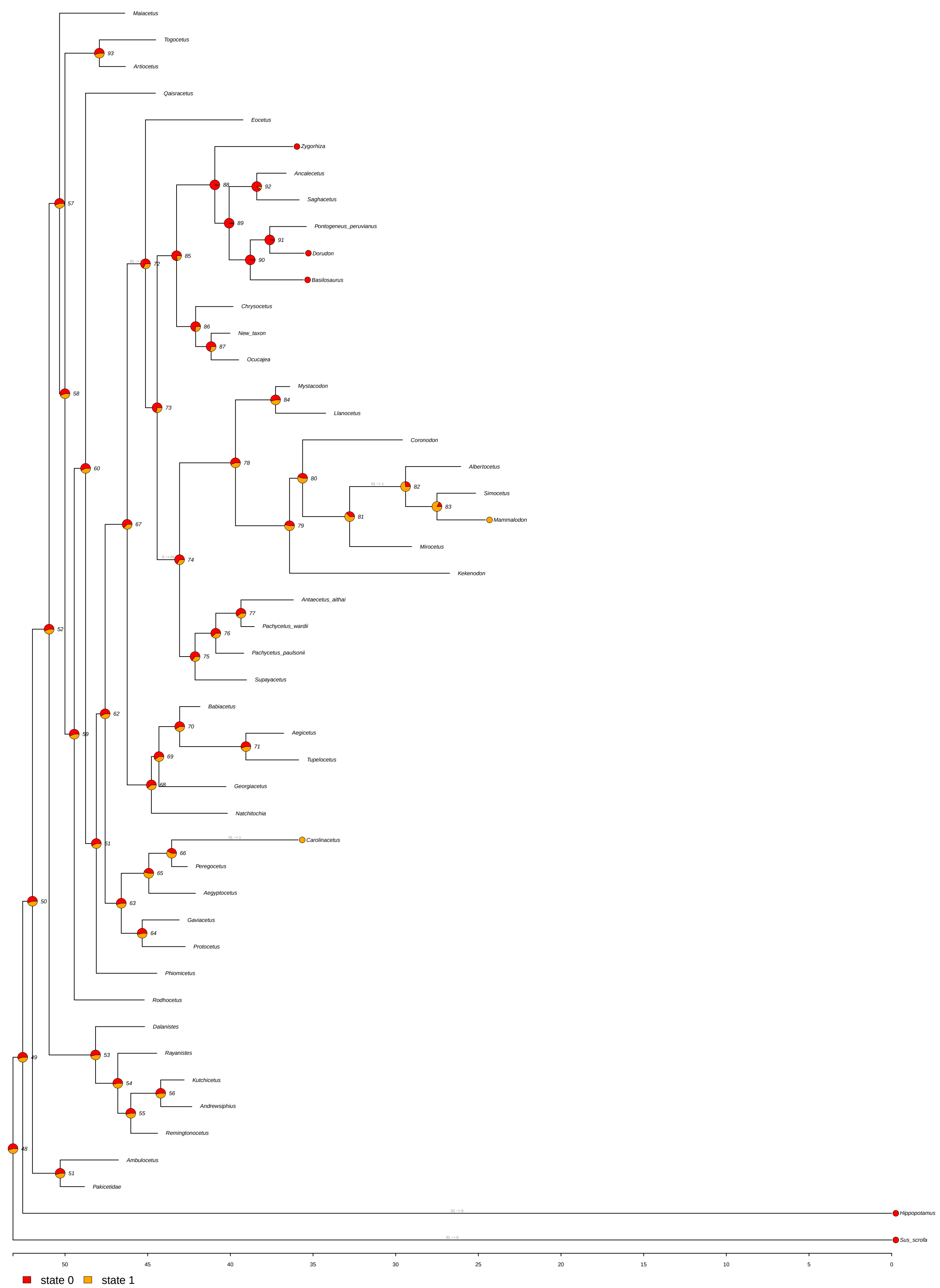

Maiacetus
Togocetus
Artiocetus
Qaisracetus
Eocetus
Zygorhiza
Ancalecetus
Saghacetus
Pontogeneus_peruvianus
Dorudon
Basilosaurus
Chrysocetus
New_taxon
Ocucajea
Mystacodon
Llanocetus
Coronodon
Albertocetus
Simocetus
Mammalodon
Mirocetus
Kekenodon
Antaecetus_aithai
Pachycetus_wardii
Pachycetus_paulsonii
Supayacetus
Babiacetus
Aegicetus
Tupelocetus
Georgiacetus
Natchitochia
Carolinacetus
Peregocetus
Aegyptocetus
Gaviacetus
Protocetus
Phiomicetus
Rodhocetus
Dalanistes
Rayanistes
Kutchicetus
Andrewsiphius
Remingtonocetus
Ambulocetus
Pakicetidae
Hippopotamus
Sus_scrofa
50
45
40
35
30
25
20
15
10
5
0
state 0
state 1

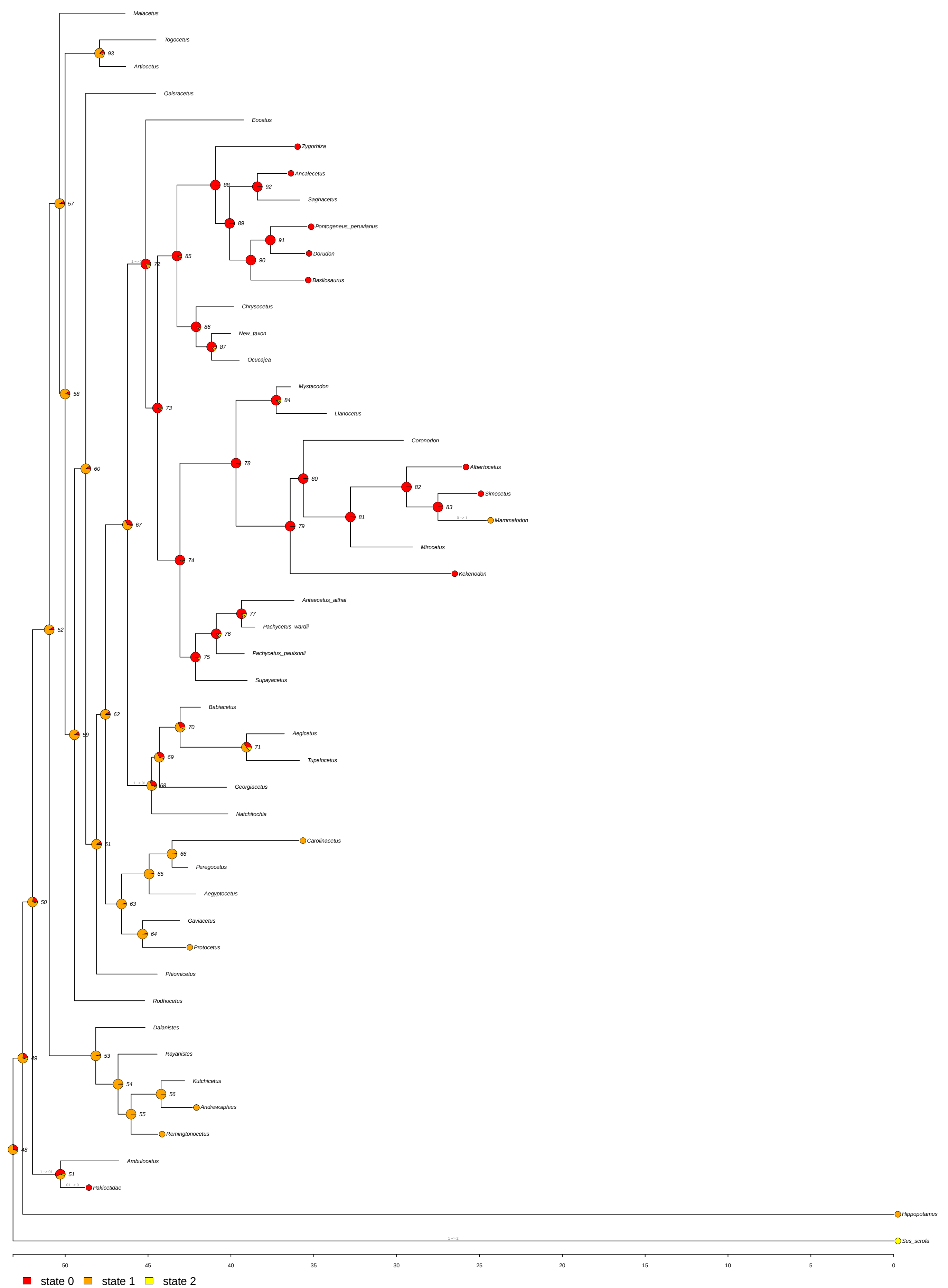
Maiacetus
Togocetus
93
Artiocetus
Qaisracetus
Eocetus
Zygorhiza
Ancalecetus
88
92
Saghacetus
89
Pontogeneus_peruvianus
91
Dorudon
90
85
72
Basilosaurus
Chrysocetus
86
New_taxon
87
Ocucajea
57
Mystacodon
84
Llanocetus
58
73
Coronodon
Albertocetus
60
78
80
82
Simocetus
83
Mammalodon
67
79
81
Mirocetus
74
Kekenodon
Antaecetus_aithai
77
52
76
Pachycetus_wardii
Pachycetus_paulsonii
75
Supayacetus
Babiacetus
62
70
Aegicetus
59
71
69
Tupelocetus
68
Georgiacetus
Natchitochia
Carolinacetus
61
66
Peregocetus
65
Aegyptocetus
50
63
Gaviacetus
64
Protocetus
Phiomicetus
Rodhocetus
Dalanistes
53
Rayanistes
49
54
Kutchicetus
56
55
Andrewsiphius
Remingtonocetus
48
Ambulocetus
51
Pakicetidae
Hippopotamus
Sus_scrofa
50
45
40
35
30
25
20
15
10
5
0
state 0
state 1
state 2

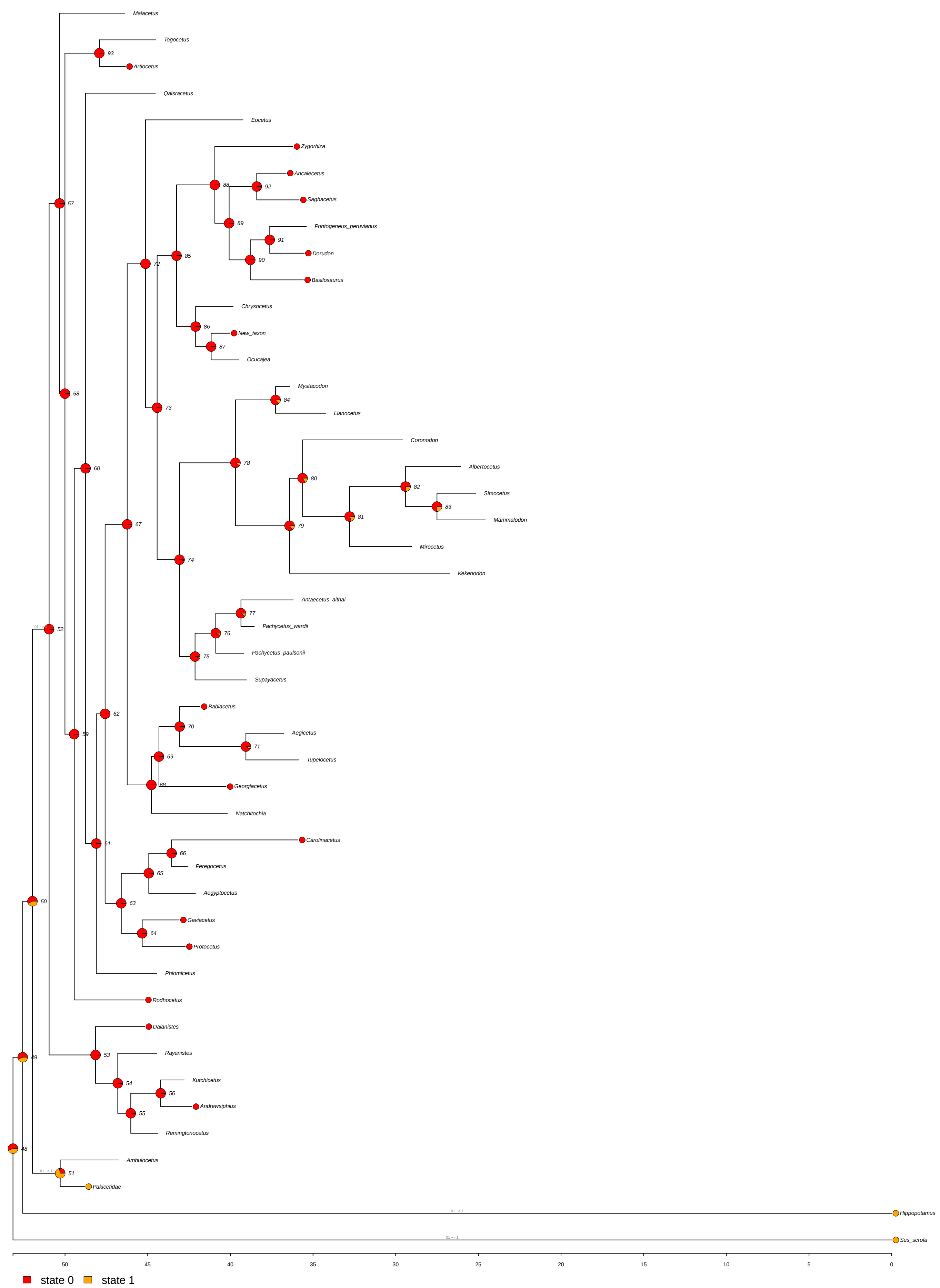
Maiacetus
Togocetus
93
Artiocetus
Qaisracetus
Eocetus
Zygorhiza
Ancalecetus
88
92
Saghacetus
89
Pontogeneus_peruvianus
91
Dorudon
90
57
72
85
Basilosaurus
Chrysocetus
86
New_taxon
87
Ocucajea
Mystacodon
84
Llanocetus
58
73
Coronodon
78
Albertocetus
80
82
Simocetus
83
60
Mammalodon
81
67
79
Mirocetus
74
Kekenodon
Antaecetus_aithai
77
52
76
Pachycetus_wardii
Pachycetus_paulsonii
75
Supayacetus
Babiacetus
62
70
Aegicetus
59
71
Tupelocetus
69
68
Georgiacetus
Natchitochia
Carolinacetus
61
66
Peregocetus
65
Aegyptocetus
50
63
Gaviacetus
64
Protocetus
Phiomicetus
Rodhocetus
Dalanistes
53
Rayanistes
49
54
Kutchicetus
56
Andrewsiphius
55
Remingtonocetus
48
Ambulocetus
51
Pakicetidae
Hippopotamus
Sus_scrofa
50
45
40
35
30
25
20
15
10
5
0
state 0
state 1

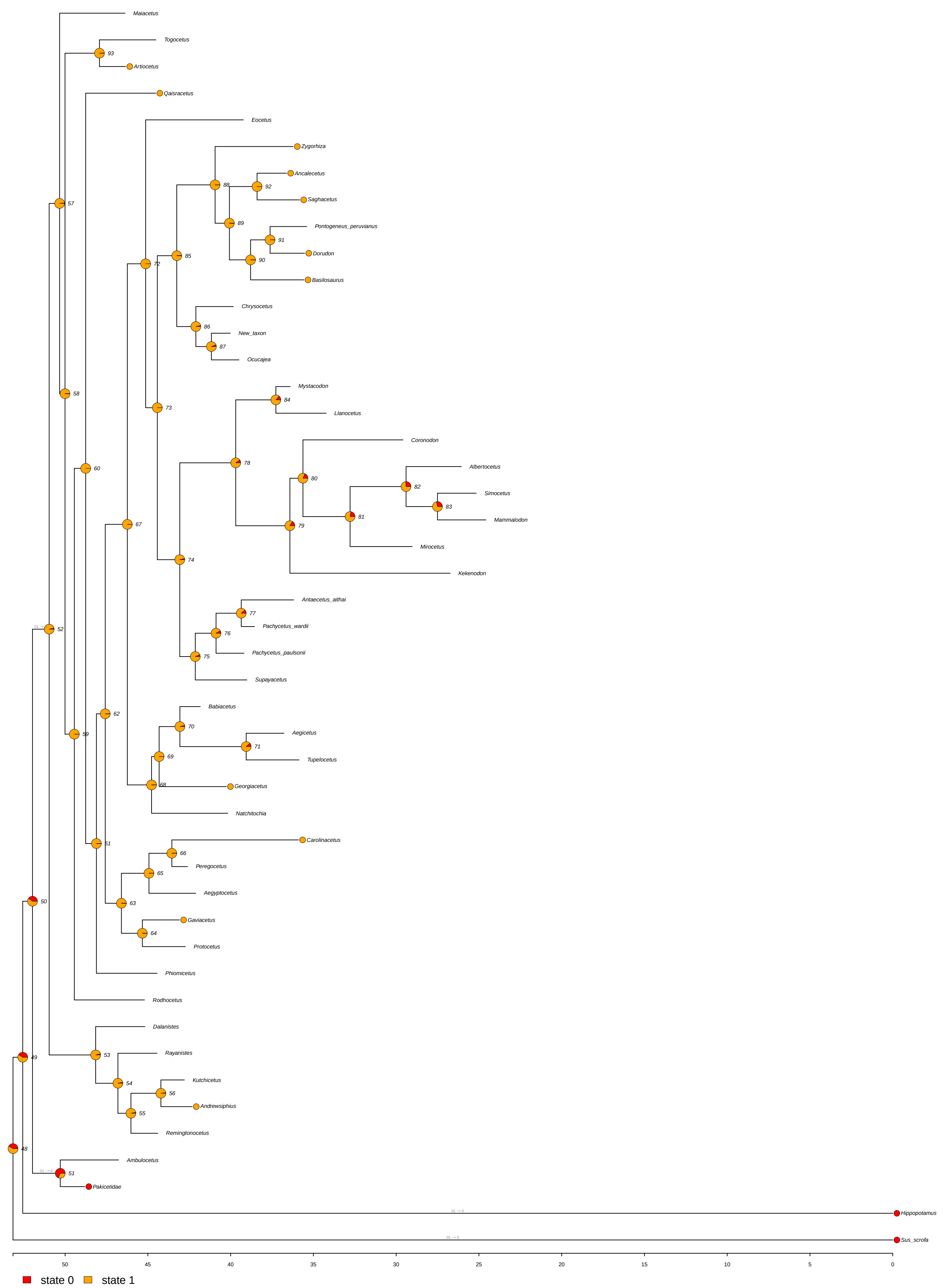
Maiacetus
Togocetus
93
Artiocetus
Qaisracetus
Eocetus
Zygorhiza
Ancalecetus
88
92
Saghacetus
57
Pontogeneus_peruvianus
89
91
Dorudon
90
85
72
Basilosaurus
Chrysocetus
86
New_taxon
87
Ocucajea
Mystacodon
58
84
Llanocetus
73
Coronodon
60
78
Albertocetus
80
82
Simocetus
83
81
Mammalodon
67
79
Mirocetus
74
Kekenodon
Antaecetus_aithai
77
Pachycetus_wardii
52
76
Pachycetus_paulsonii
75
Supayacetus
Babiacetus
62
70
Aegicetus
59
71
Tupelocetus
69
68
Georgiacetus
Natchitochia
Carolinacetus
61
66
Peregocetus
65
50
Aegyptocetus
63
Gaviacetus
64
Protocetus
Phiomicetus
Rodhocetus
Dalanistes
53
Rayanistes
49
54
Kutchicetus
56
55
Andrewsiphius
Remingtonocetus
48
Ambulocetus
51
Pakicetidae
Hippopotamus
Sus_scrofa
50
45
40
35
30
25
20
15
10
5
0
state 0
state 1

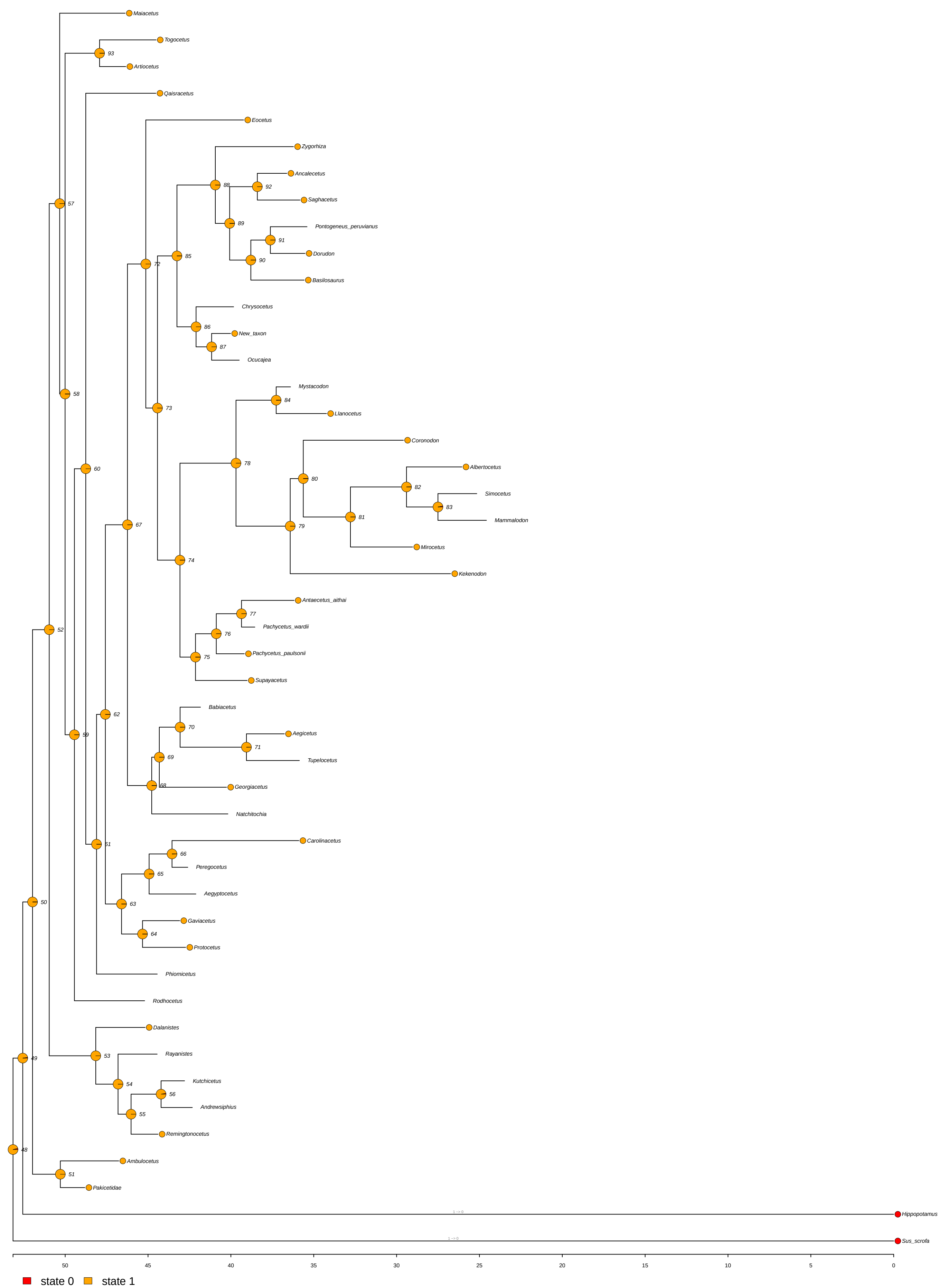

Maiacetus
Togocetus
93
Artiocetus
Qaisracetus
Eocetus
Zygorhiza
Ancalecetus
88
92
Saghacetus
Pontogeneus_peruvianus
89
91
Dorudon
90
Basilosaurus
57
72
85
Chrysocetus
86
New_taxon
87
Ocucajea
Mystacodon
84
Llanocetus
58
73
Coronodon
78
Albertocetus
60
80
82
Simocetus
83
Mammalodon
67
81
79
Mirocetus
74
Kekenodon
Antaecetus_aithai
77
Pachycetus_wardii
52
76
Pachycetus_paulsonii
75
Supayacetus
Babiacetus
62
70
Aegicetus
59
71
69
Tupelocetus
68
Georgiacetus
Natchitochia
Carolinacetus
61
66
Peregocetus
65
Aegyptocetus
50
63
Gaviacetus
64
Protocetus
Phiomicetus
Rodhocetus
Dalanistes
53
Rayanistes
49
54
Kutchicetus
56
Andrewsiphius
55
Remingtonocetus
48
Ambulocetus
51
Pakicetidae
1 -> 0
Hippopotamus
1 -> 0
Sus_scrofa
50
45
40
35
30
25
20
15
10
5
0
state 0
state 1

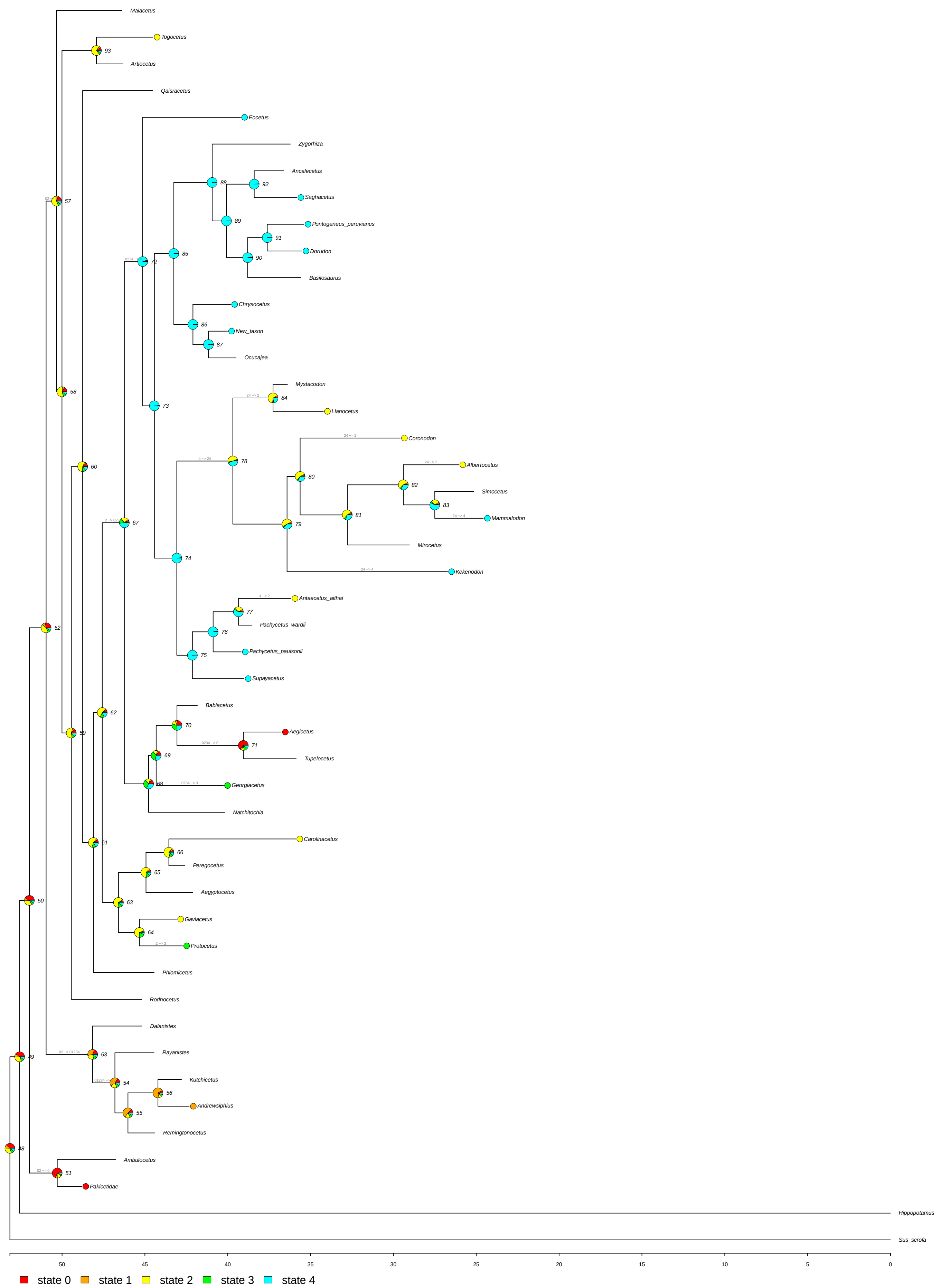

Maiacetus
Togocetus
93
Artiocetus
Qaisracetus
Eocetus
Zygorhiza
Ancalecetus
88
92
Saghacetus
Pontogeneus_peruvianus
89
91
Dorudon
90
Basilosaurus
57
72
85
Chrysocetus
86
New_taxon
87
Ocucajea
Mystacodon
84
Llanocetus
58
73
Coronodon
60
78
Albertocetus
80
82
Simocetus
83
Mammalodon
81
67
79
Mirocetus
74
Kekenodon
Antaecetus_aithai
77
Pachycetus_wardii
52
76
Pachycetus_paulsonii
75
Supayacetus
Babiacetus
62
70
Aegicetus
59
71
Tupelocetus
69
68
Georgiacetus
Natchitochia
61
Carolinacetus
66
Peregocetus
65
Aegyptocetus
50
63
Gaviacetus
64
Protocetus
Phiomicetus
Rodhocetus
Dalanistes
Rayanistes
53
54
Kutchicetus
56
Andrewsiphius
55
Remingtonocetus
48
Ambulocetus
51
Pakicetidae
Hippopotamus
Sus_scrofa
50
45
40
35
30
25
20
15
10
5
0
state 0
state 1
state 2
state 3
state 4

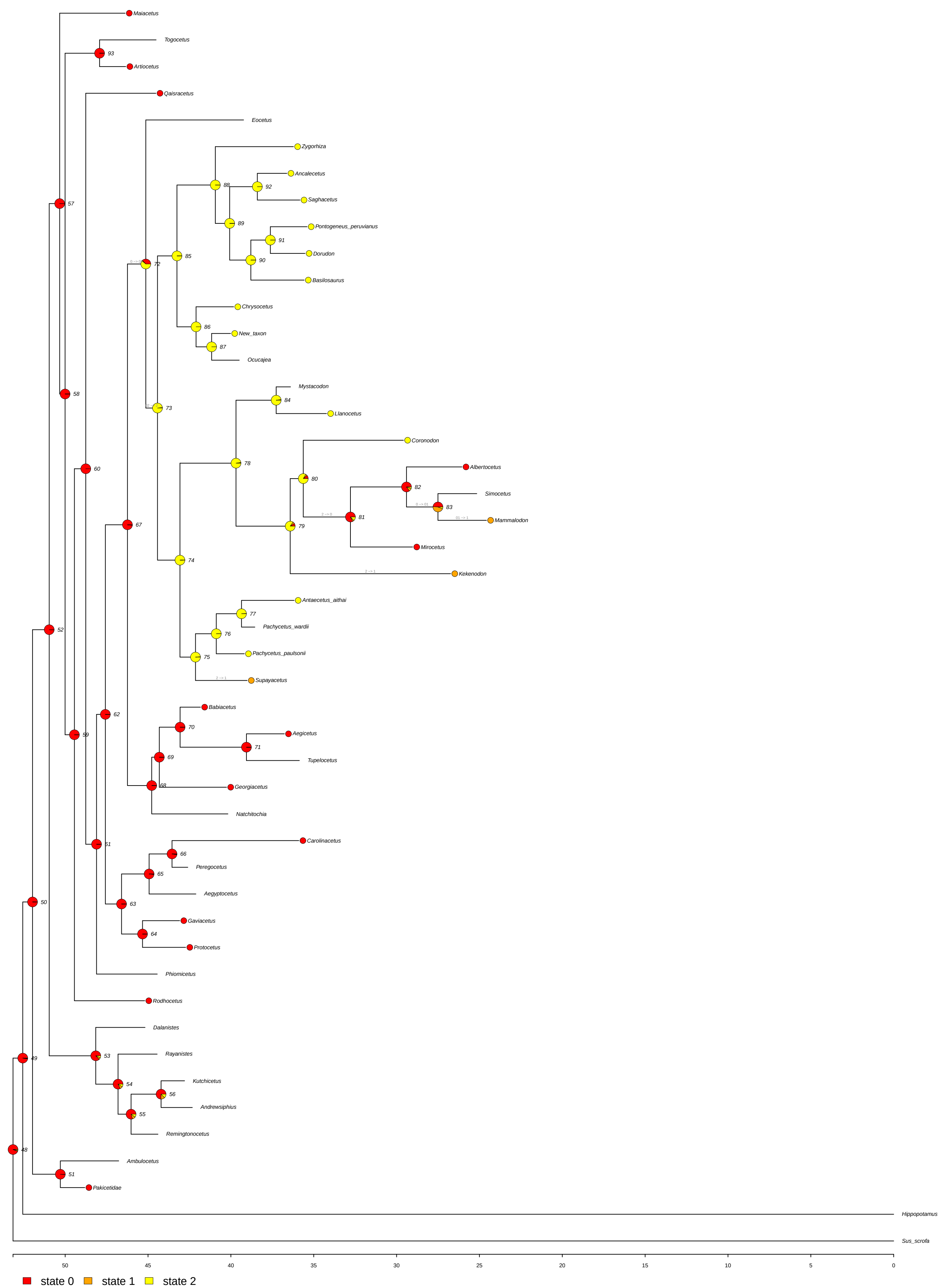
Maiacetus
Togocetus
93
Artiocetus
Qaisracetus
Eocetus
Zygorhiza
Ancalecetus
Saghacetus
Pontogeneus_peruvianus
Dorudon
Basilosaurus
Chrysocetus
New_taxon
Ocucajea
Mystacodon
Llanocetus
Coronodon
Albertocetus
Simocetus
Mammalodon
Mirocetus
Kekenodon
Antaecetus_aithai
Pachycetus_wardii
Pachycetus_paulsonii
Supayacetus
Babiacetus
Aegicetus
Tupelocetus
Georgiacetus
Natchitochia
Carolinacetus
Peregocetus
Aegyptocetus
Gaviacetus
Protocetus
Phiomicetus
Rodhocetus
Dalanistes
Rayanistes
Kutchicetus
Andrewsiphius
Remingtonocetus
Ambulocetus
Pakicetidae
Hippopotamus
Sus_scrofa
50
45
40
35
30
25
20
15
10
5
0
state 0
state 1
state 2

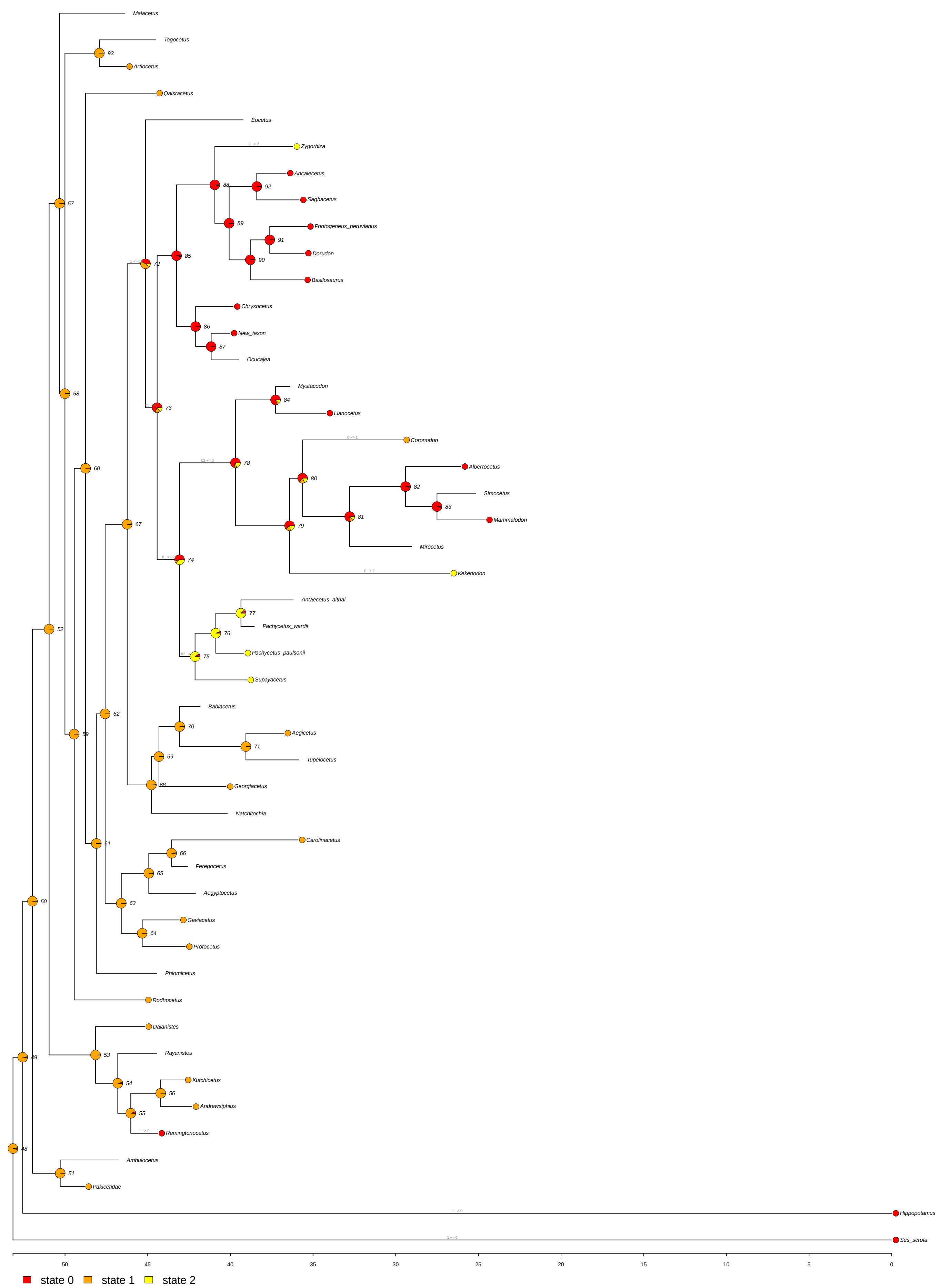

Maiacetus
Togocetus
93
Artiocetus
Qaisracetus
Eocetus
0 -> 2
Zygorhiza
Ancalecetus
88
92
Saghacetus
Pontogeneus_peruvianus
89
91
Dorudon
90
Basilosaurus
1 -> 0
72
85
Chrysocetus
86
New_taxon
87
Ocucajea
Mystacodon
84
Llanocetus
73
0 -> 1
Coronodon
02 -> 0
78
Albertocetus
80
82
Simocetus
83
Mammalodon
81
79
Mirocetus
0 -> 02
74
0 -> 2
Kekenodon
Antaecetus_aithai
77
Pachycetus_wardii
76
Pachycetus_paulsonii
75
Supayacetus
57
58
60
67
52
59
62
Babiacetus
70
Aegicetus
71
Tupelocetus
69
68
Georgiacetus
Natchitochia
61
Carolinacetus
66
Peregocetus
65
Aegyptocetus
63
Gaviacetus
64
Protocetus
Phiomicetus
Rodhocetus
50
Dalanistes
49
53
Rayanistes
54
Kutchicetus
56
Andrewsiphius
55
1 -> 0
Remingtonocetus
48
Ambulocetus
51
Pakicetidae
1 -> 0
Hippopotamus
1 -> 0
Sus_scrofa
50
45
40
35
30
25
20
15
10
5
0
state 0
state 1
state 2

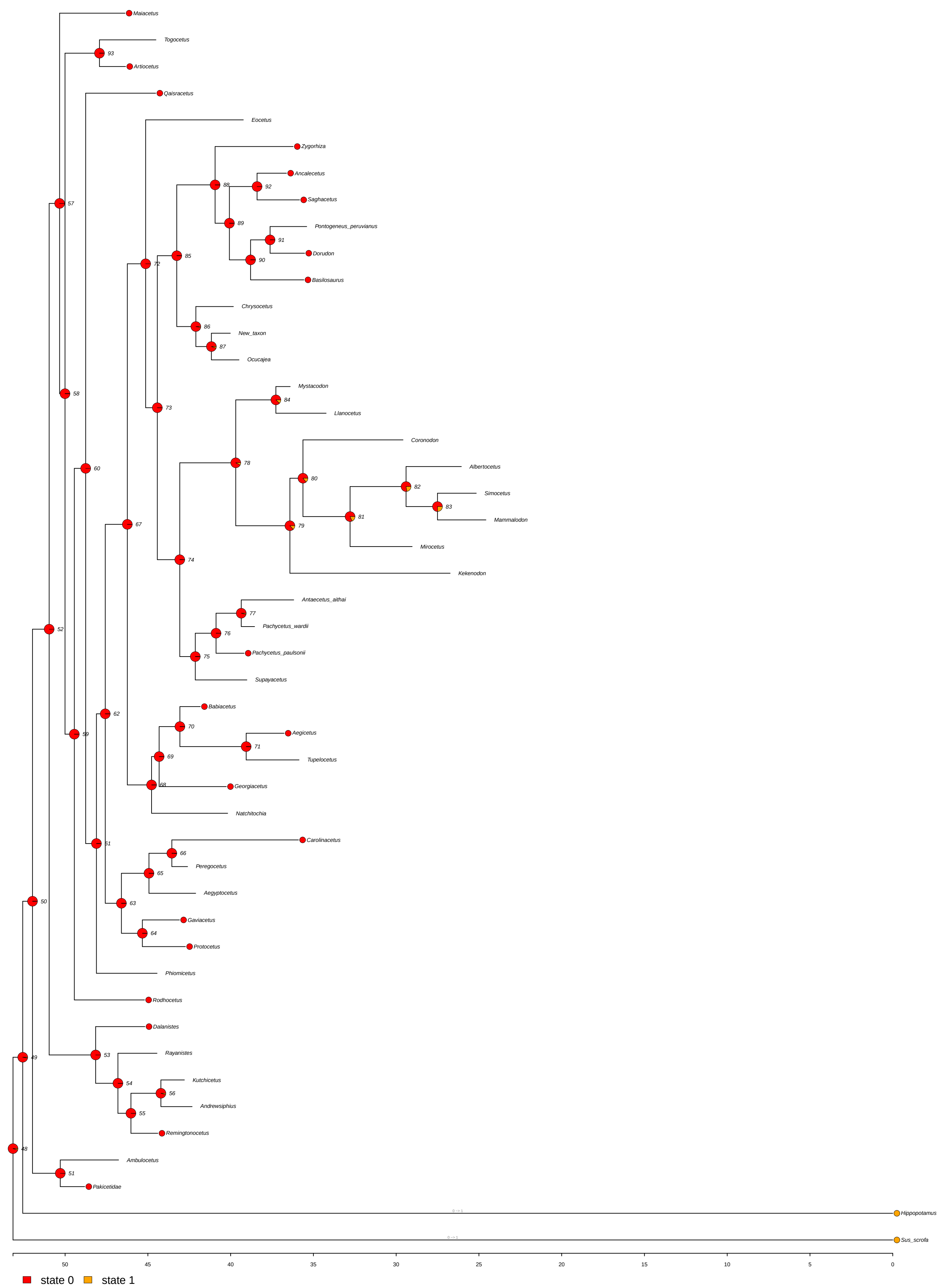
Maiacetus
Togocetus
93
Artiocetus
Qaisracetus
Eocetus
Zygorhiza
Ancalecetus
88
92
Saghacetus
89
Pontogeneus_peruvianus
91
Dorudon
90
Basilosaurus
57
72
85
Chrysocetus
86
New_taxon
87
Ocucajea
Mystacodon
84
Llanocetus
58
73
Coronodon
Albertocetus
78
80
82
Simocetus
83
Mammalodon
81
79
Mirocetus
60
67
74
Kekenodon
Antaecetus_aithai
77
Pachycetus_wardii
76
Pachycetus_paulsonii
75
Supayacetus
52
Babiacetus
62
70
Aegicetus
59
71
Tupelocetus
69
68
Georgiacetus
Natchitochia
Carolinacetus
61
66
Peregocetus
65
Aegyptocetus
50
63
Gaviacetus
64
Protocetus
Phiomicetus
Rodhocetus
Dalanistes
49
53
Rayanistes
Kutchicetus
54
56
Andrewsiphius
55
Remingtonocetus
48
Ambulocetus
51
Pakicetidae
0 -> 1
Hippopotamus
0 -> 1
Sus_scrofa
50
45
40
35
30
25
20
15
10
5
0
state 0
state 1

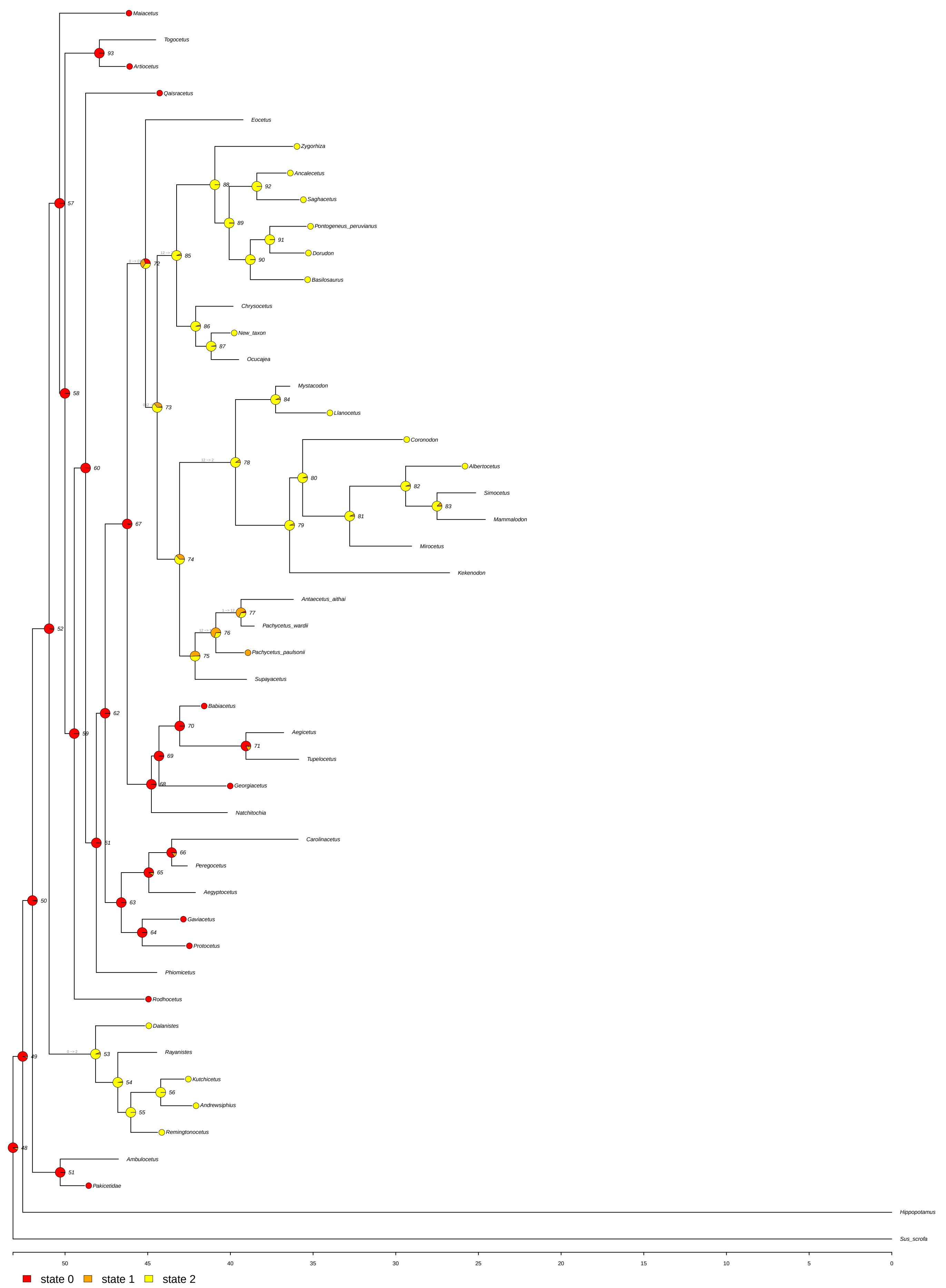

Maiacetus
Togocetus
93
Artiocetus
Qaisracetus
Eocetus
Zygorhiza
Ancalecetus
88
92
Saghacetus
89
Pontogeneus_peruvianus
91
Dorudon
85
90
72
Basilosaurus
Chrysocetus
86
New_taxon
87
Ocucajea
Mystacodon
84
Llanocetus
73
Coronodon
78
Albertocetus
80
82
Simocetus
83
Mammalodon
81
79
Mirocetus
74
Kekenodon
Antaecetus_aithai
77
Pachycetus_wardii
76
Pachycetus_paulsonii
75
Supayacetus
Babiacetus
70
Aegicetus
71
Tupelocetus
69
68
Georgiacetus
Natchitochia
Carolinacetus
66
Peregocetus
65
Aegyptocetus
63
Gaviacetus
64
Protocetus
Phiomicetus
Rodhocetus
Dalanistes
53
Rayanistes
54
Kutchicetus
56
Andrewsiphius
55
Remingtonocetus
48
Ambulocetus
51
Pakicetidae
Hippopotamus
Sus_scrofa
57
58
60
67
62
59
61
52
50
49
0 -> 2
12 -> 2
1 -> 12
50
45
40
35
30
25
20
15
10
5
0
state 0
state 1
state 2

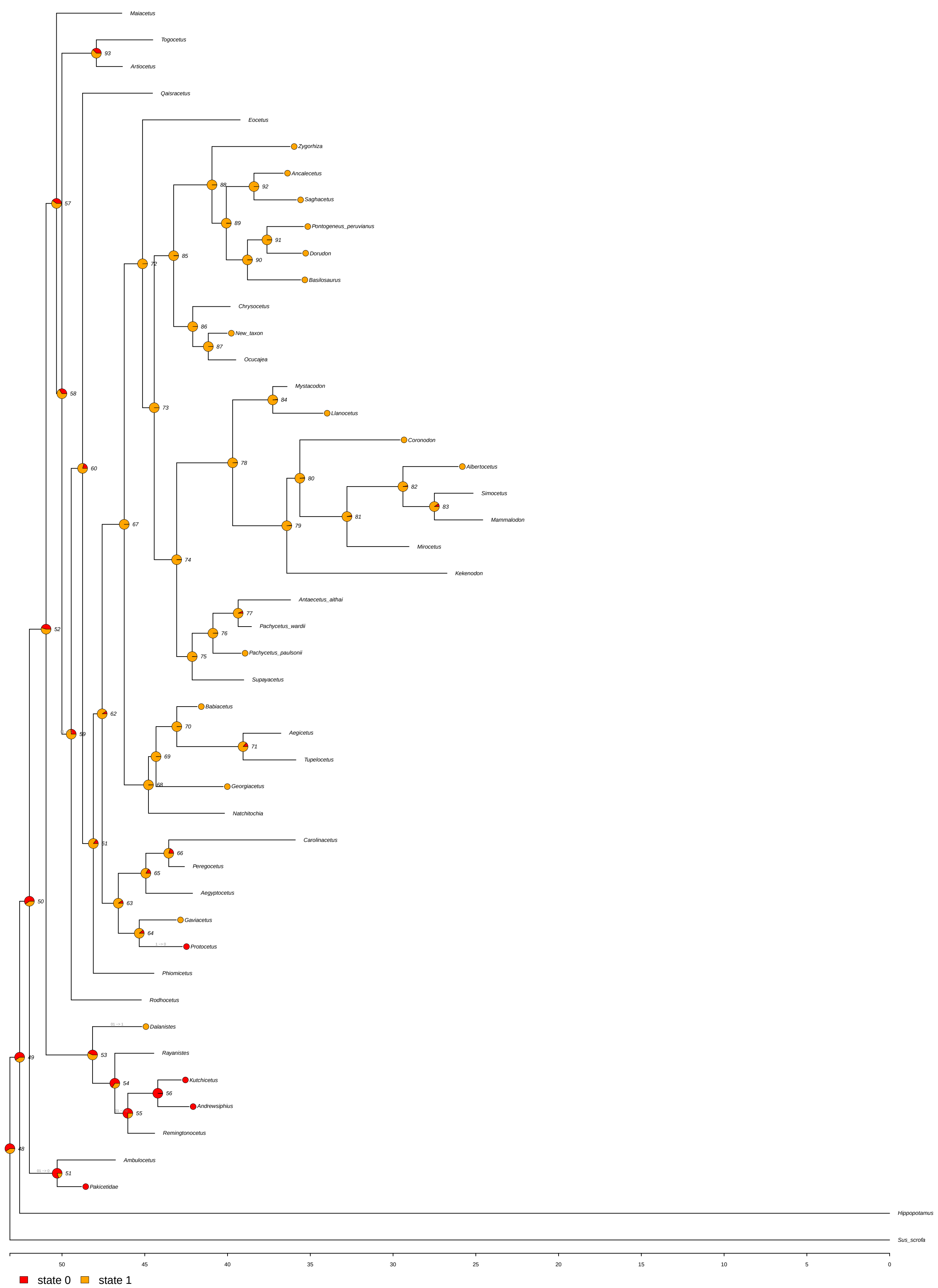

Maiacetus
Togocetus
93
Artiocetus
Qaisracetus
Eocetus
Zygorhiza
Ancalecetus
88
92
Saghacetus
Pontogeneus_peruvianus
89
91
Dorudon
90
Basilosaurus
57
72
85
Chrysocetus
86
New_taxon
87
Ocucajea
Mystacodon
84
Llanocetus
58
73
Coronodon
60
78
Albertocetus
80
82
Simocetus
83
67
81
Mammalodon
79
Mirocetus
74
Kekenodon
Antaecetus_aithai
77
52
Pachycetus_wardii
76
Pachycetus_paulsonii
75
Supayacetus
Babiacetus
62
70
Aegicetus
59
71
Tupelocetus
69
Georgiacetus
68
Natchitochia
Carolinacetus
61
66
Peregocetus
65
50
Aegyptocetus
63
Gaviacetus
64
1 -> 0
Protocetus
Phiomicetus
Rodhocetus
01 -> 1
Dalanistes
Rayanistes
49
53
Kutchicetus
54
56
Andrewsiphius
55
Remingtonocetus
48
Ambulocetus
01 -> 0
51
Pakicetidae
Hippopotamus
Sus_scrofa
50
45
40
35
30
25
20
15
10
5
0
state 0
state 1

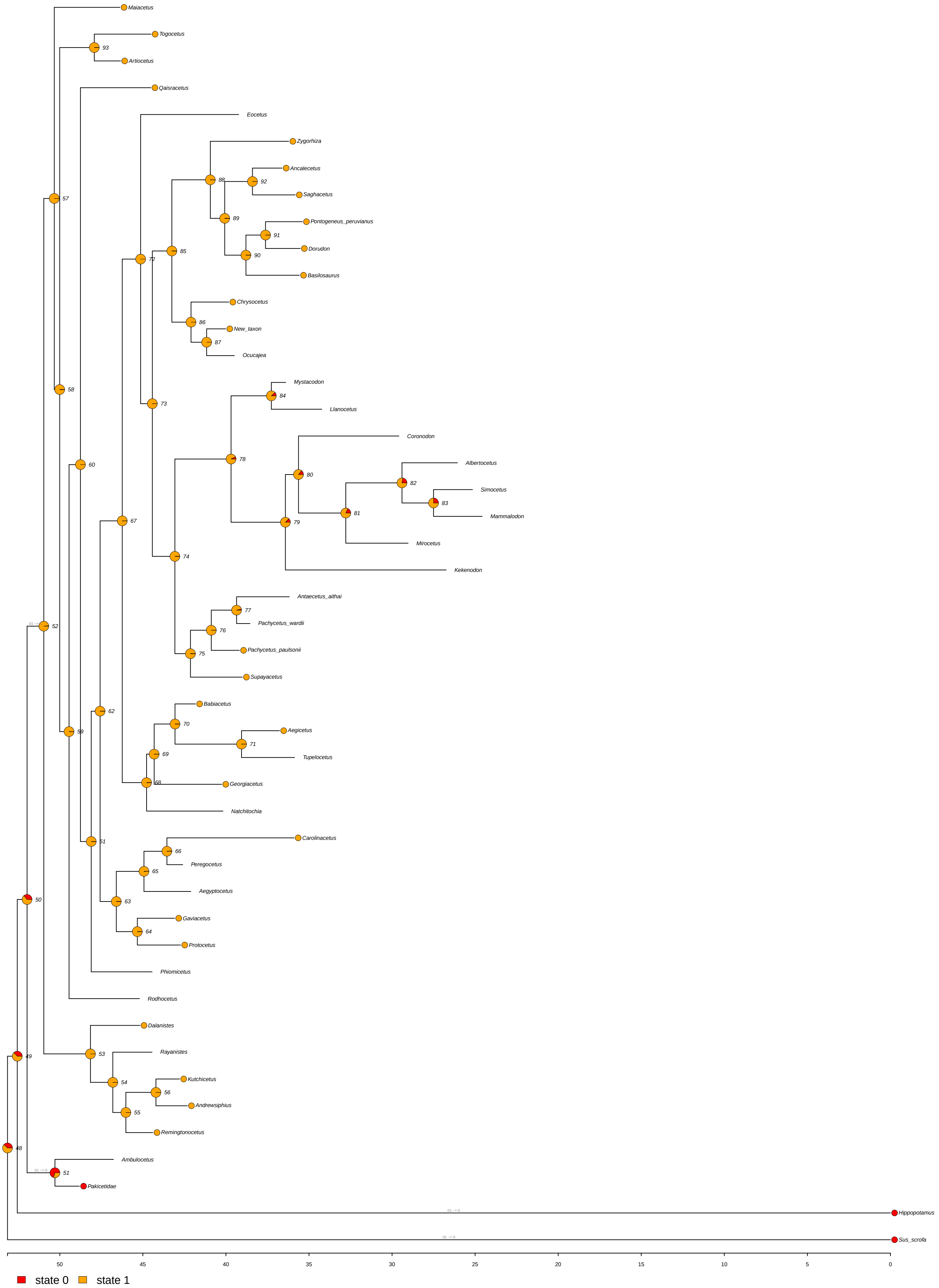

Maiacetus
Togocetus
93
Artiocetus
Qaisracetus
Eocetus
Zygorhiza
Ancalecetus
88
92
Saghacetus
89
Pontogeneus_peruvianus
91
Dorudon
90
Basilosaurus
72
85
Chrysocetus
86
New_taxon
87
Ocucajea
Mystacodon
84
Llanocetus
58
73
Coronodon
60
78
Albertocetus
80
82
Simocetus
83
81
Mammalodon
67
79
Mirocetus
74
Kekenodon
Antaecetus_aithai
77
Pachycetus_wardii
52
76
Pachycetus_paulsonii
75
Supayacetus
Babiacetus
62
70
Aegicetus
59
71
Tupelocetus
69
68
Georgiacetus
Natchitochia
61
Carolinacetus
66
Peregocetus
65
50
Aegyptocetus
63
Gaviacetus
64
Protocetus
Phiomicetus
Rodhocetus
Dalanistes
Rayanistes
53
49
54
Kutchicetus
56
Andrewsiphius
55
Remingtonocetus
48
Ambulocetus
51
Pakicetidae
Hippopotamus
Sus_scrofa
50
45
40
35
30
25
20
15
10
5
0
state 0
state 1

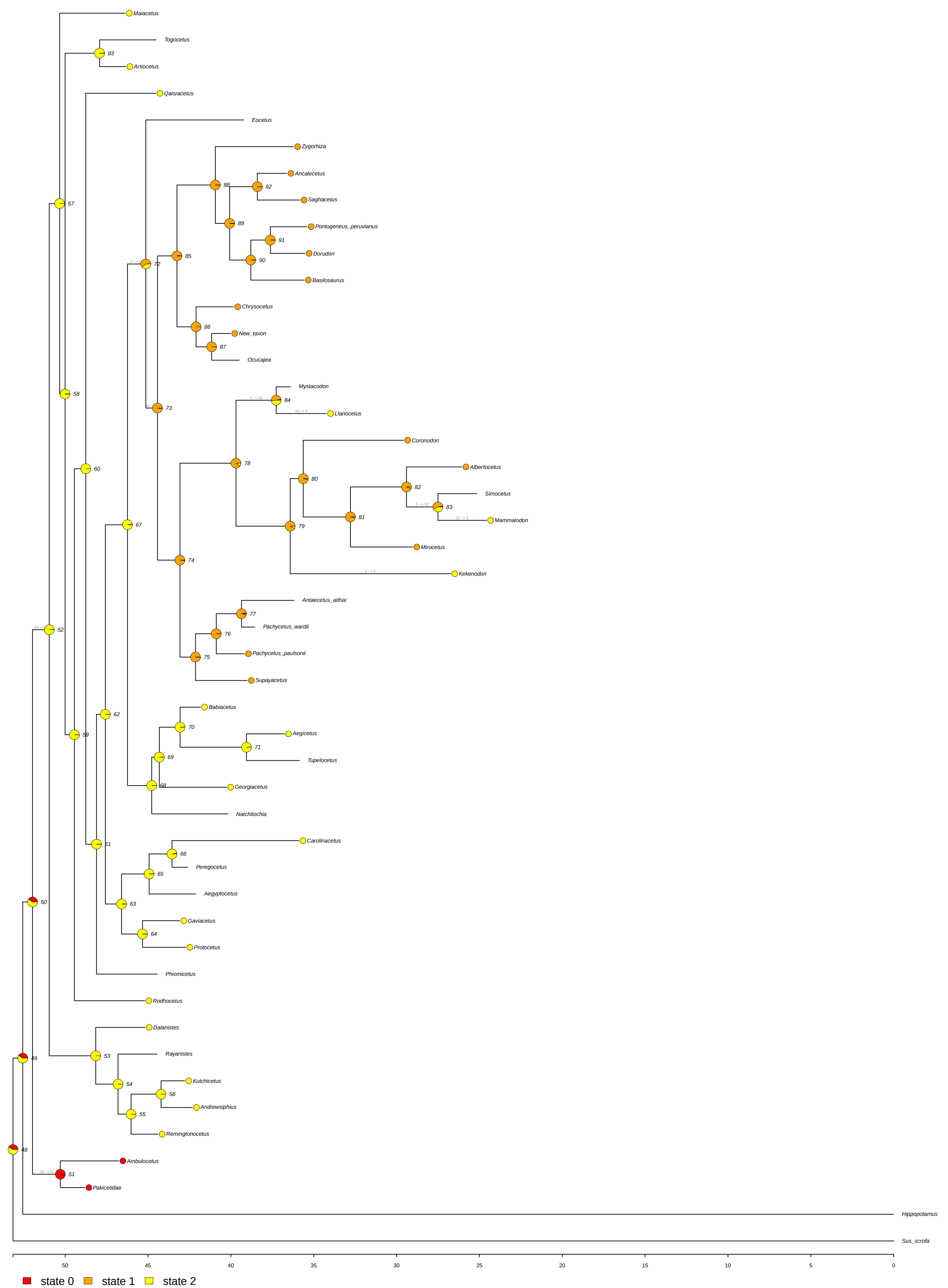

Maiacetus
Togocetus
Artiocetus
Qaisracetus
Eocetus
Zygorhiza
Ancalecetus
Saghacetus
Pontogeneus_peruvianus
Dorudon
Basilosaurus
Chrysocetus
New_taxon
Ocucajea
Mystacodon
Llanocetus
Coronodon
Albertocetus
Simocetus
Mammalodon
Mirocetus
Kekenodon
Antaecetus_aithai
Pachycetus_wardii
Pachycetus_paulsonii
Supayacetus
Babiacetus
Aegicetus
Tupelocetus
Georgiacetus
Natchitochia
Carolinacetus
Peregocetus
Aegyptocetus
Gaviacetus
Protocetus
Phiomicetus
Rodhocetus
Dalanistes
Rayanistes
Kutchicetus
Andrewsiphius
Remingtonocetus
Ambulocetus
Pakicetidae
Hippopotamus
Sus_scrofa
50
45
40
35
30
25
20
15
10
5
0
state 0
state 1
state 2

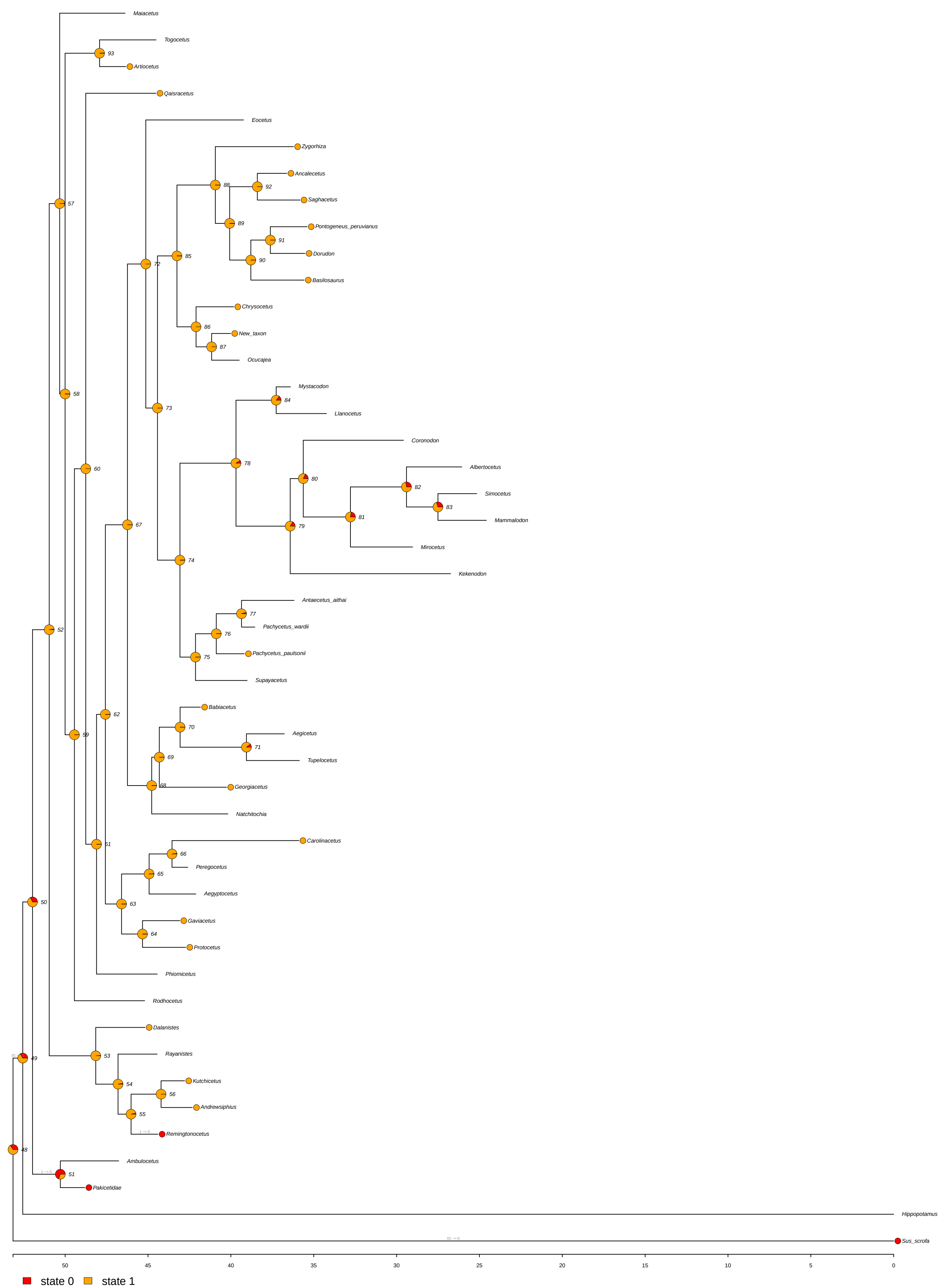

Maiacetus
Togocetus
93
Artiocetus
Qaisracetus
Eocetus
Zygorhiza
Ancalecetus
88
92
Saghacetus
Pontogeneus_peruvianus
89
91
Dorudon
90
Basilosaurus
72
85
Chrysocetus
86
New_taxon
87
Ocucajea
57
Mystacodon
84
Llanocetus
58
73
Coronodon
78
60
Albertocetus
80
82
Simocetus
83
Mammalodon
81
67
79
Mirocetus
74
Kekenodon
Antaecetus_aithai
77
Pachycetus_wardii
52
76
Pachycetus_paulsonii
75
Supayacetus
Babiacetus
62
70
Aegicetus
59
71
Tupelocetus
69
68
Georgiacetus
Natchitochia
Carolinacetus
61
66
Peregocetus
65
50
Aegyptocetus
63
Gaviacetus
64
Protocetus
Phiomicetus
Rodhocetus
Dalanistes
Rayanistes
53
49
Kutchicetus
54
56
Andrewsiphius
55
Remingtonocetus
48
Ambulocetus
51
Pakicetidae
Hippopotamus
Sus_scrofa
50
45
40
35
30
25
20
15
10
5
0
state 0
state 1

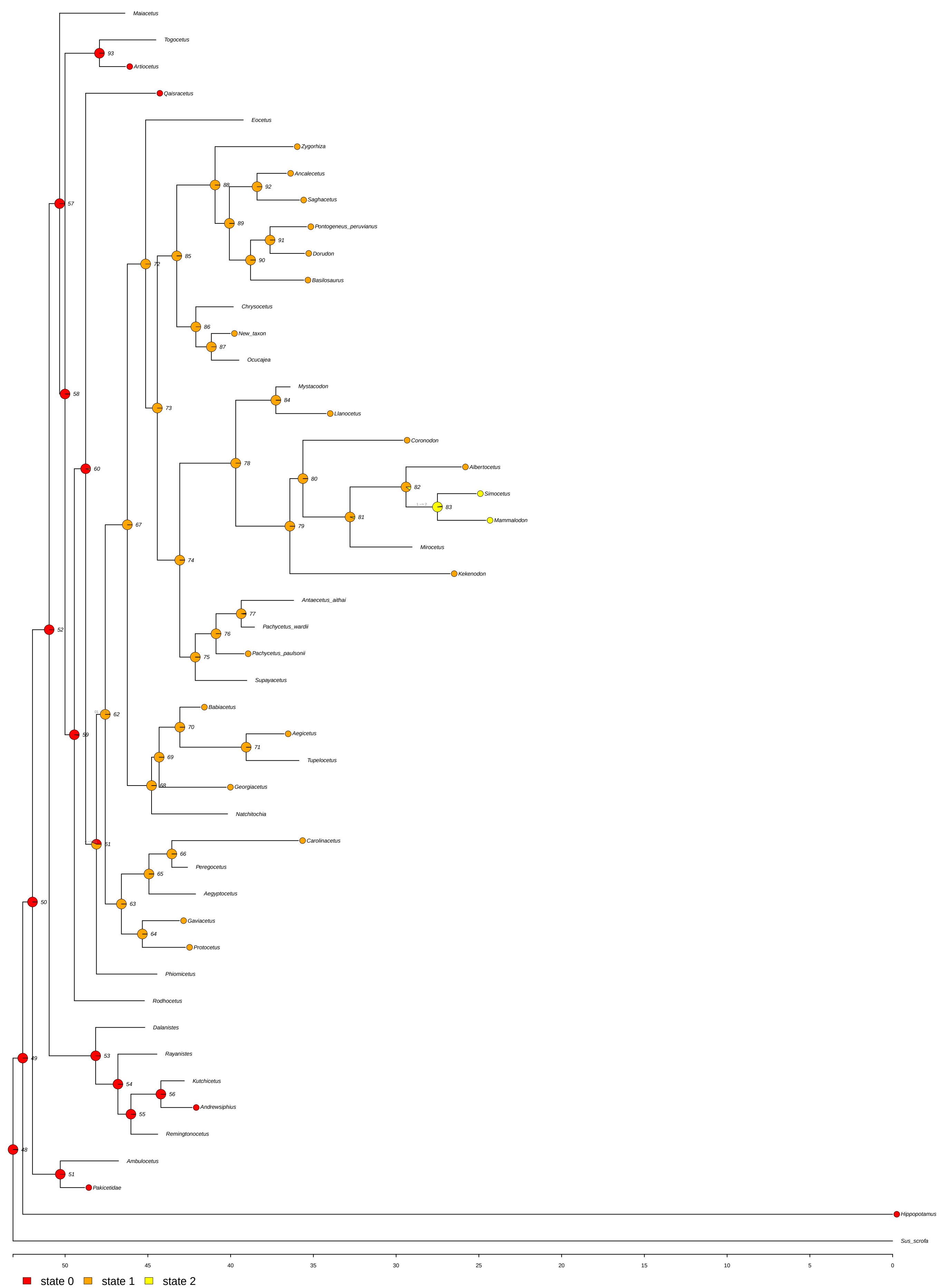

Maiacetus
Togocetus
Artiocetus
Qaisracetus
Eocetus
Zygorhiza
Ancalecetus
Saghacetus
Pontogeneus_peruvianus
Dorudon
Basilosaurus
Chrysocetus
New_taxon
Ocucajea
Mystacodon
Llanocetus
Coronodon
Albertocetus
Simocetus
Mammalodon
Mirocetus
Kekenodon
Antaecetus_aithai
Pachycetus_wardii
Pachycetus_paulsonii
Supayacetus
Babiacetus
Aegicetus
Tupelocetus
Georgiacetus
Natchitochia
Carolinacetus
Peregocetus
Aegyptocetus
Gaviacetus
Protocetus
Phiomicetus
Rodhocetus
Dalanistes
Rayanistes
Kutchicetus
Andrewsiphius
Remingtonocetus
Ambulocetus
Pakicetidae
Hippopotamus
Sus_scrofa
93
57
58
60
59
52
50
49
48
51
53
54
55
56
61
62
63
64
65
66
67
68
69
70
71
72
73
74
75
76
77
78
79
80
81
82
83
84
85
86
87
88
89
90
91
92
1 -> 2
50
45
40
35
30
25
20
15
10
5
0
state 0
state 1
state 2

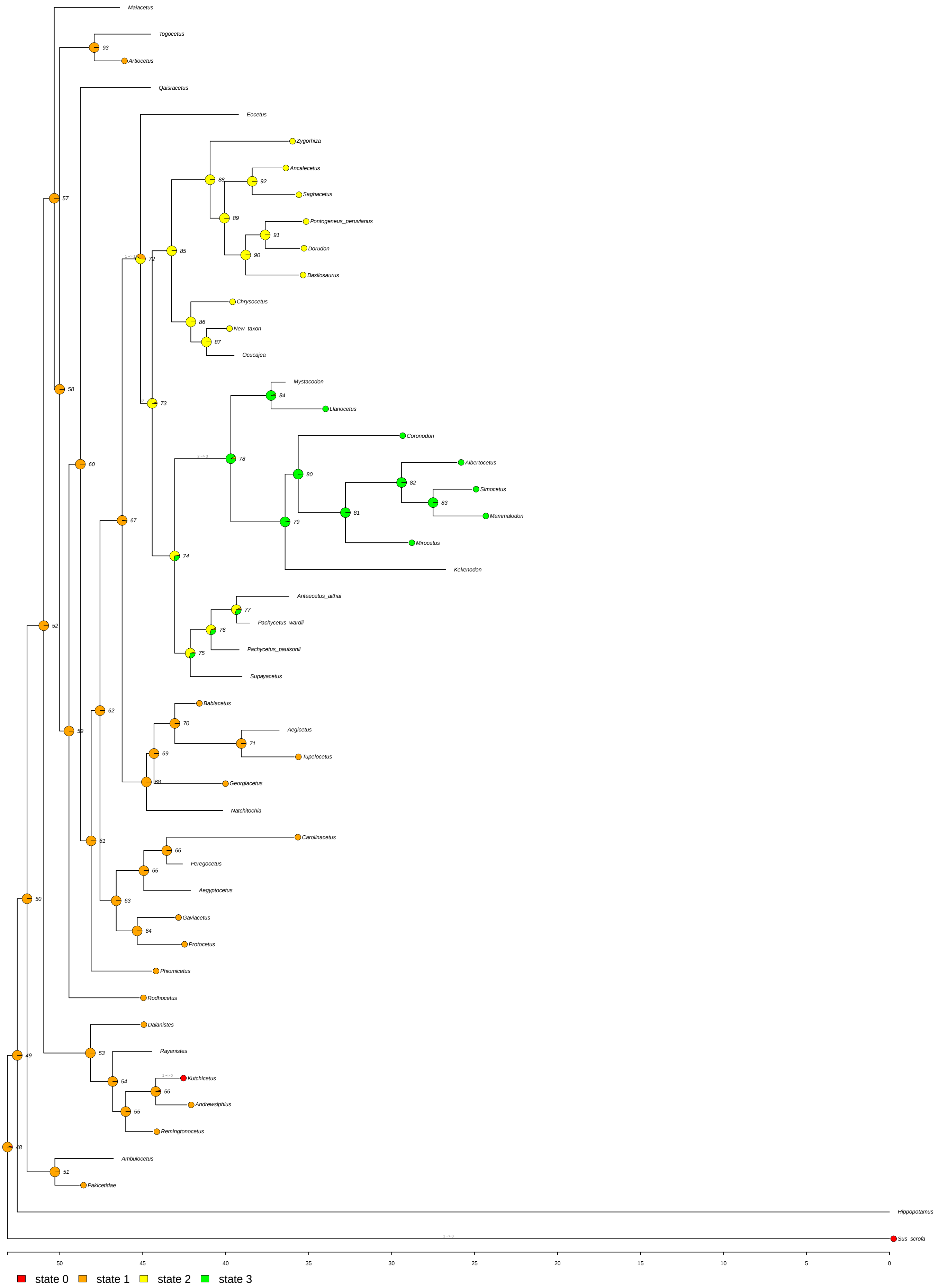
Maiacetus
Togocetus
Artiocetus
Qaisracetus
Eocetus
Zygorhiza
Ancalecetus
Saghacetus
Pontogeneus_peruvianus
Dorudon
Basilosaurus
Chrysocetus
New_taxon
Ocucajea
Mystacodon
Llanocetus
Coronodon
Albertocetus
Simocetus
Mammalodon
Mirocetus
Kekenodon
Antaecetus_aithai
Pachycetus_wardii
Pachycetus_paulsonii
Supayacetus
Babiacetus
Aegicetus
Tupelocetus
Georgiacetus
Natchitochia
Carolinacetus
Peregocetus
Aegyptocetus
Gaviacetus
Protocetus
Phiomicetus
Rodhocetus
Dalanistes
Rayanistes
Kutchicetus
Andrewsiphius
Remingtonocetus
Ambulocetus
Pakicetidae
Hippopotamus
Sus_scrofa
50 45 40 35 30 25 20 15 10 5 0
state 0
state 1
state 2
state 3

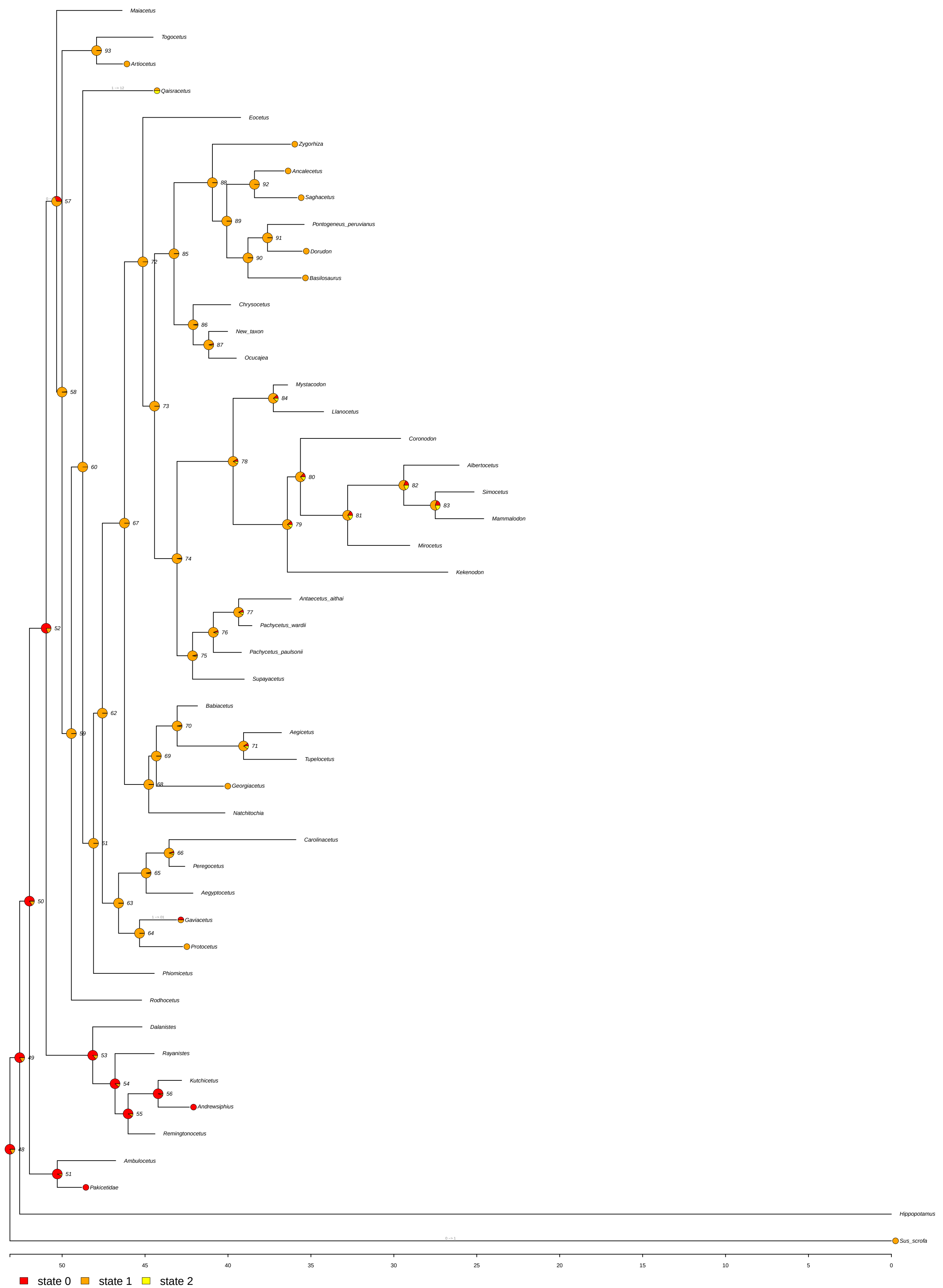
Maiacetus
Togocetus
Artiocetus
Qaisracetus
Eocetus
Zygorhiza
Ancalecetus
Saghacetus
Pontogeneus_peruvianus
Dorudon
Basilosaurus
Chrysocetus
New_taxon
Ocucajea
Mystacodon
Llanocetus
Coronodon
Albertocetus
Simocetus
Mammalodon
Mirocetus
Kekenodon
Antaecetus_aithai
Pachycetus_wardii
Pachycetus_paulsonii
Supayacetus
Babiacetus
Aegicetus
Tupelocetus
Georgiacetus
Natchitochia
Carolinacetus
Peregocetus
Aegyptocetus
Gaviacetus
Protocetus
Phiomicetus
Rodhocetus
Dalanistes
Rayanistes
Kutchicetus
Andrewsiphius
Remingtonocetus
Ambulocetus
Pakicetidae
Hippopotamus
Sus_scrofa
50 45 40 35 30 25 20 15 10 5 0
state 0
state 1
state 2

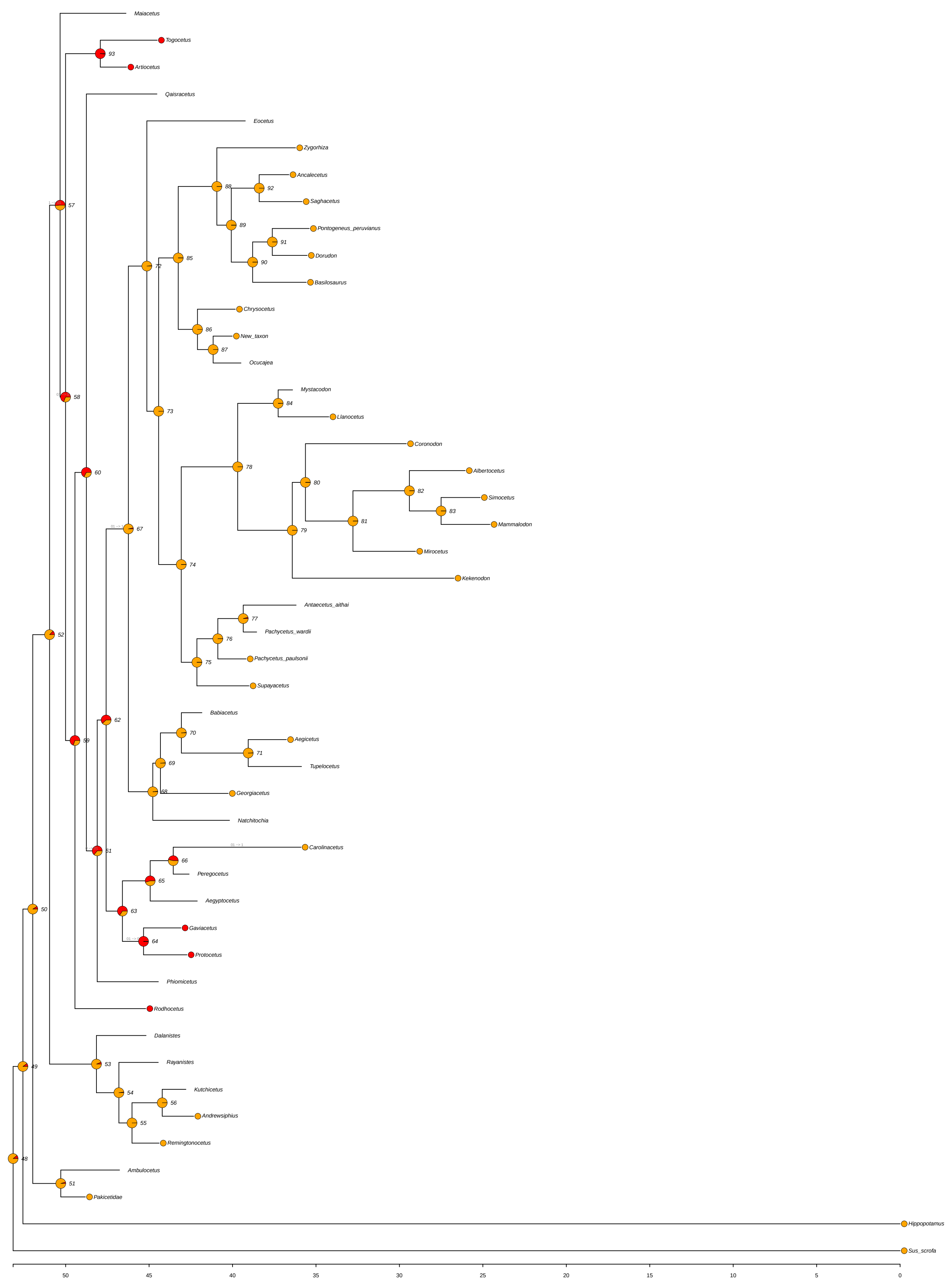
Maiacetus
Togocetus
Artiocetus
Qaisracetus
Eocetus
Zygorhiza
Ancalecetus
Saghacetus
Pontogeneus_peruvianus
Dorudon
Basilosaurus
Chrysocetus
New_taxon
Ocucajea
Mystacodon
Llanocetus
Coronodon
Albertocetus
Simocetus
Mammalodon
Mirocetus
Kekenodon
Antaecetus_aithai
Pachycetus_wardii
Pachycetus_paulsonii
Supayacetus
Babiacetus
Aegicetus
Tupelocetus
Georgiacetus
Natchitochia
Carolinacetus
Peregocetus
Aegyptocetus
Gaviacetus
Protocetus
Phiomicetus
Rodhocetus
Dalanistes
Rayanistes
Kutchicetus
Andrewsiphius
Remingtonocetus
Ambulocetus
Pakicetidae
Hippopotamus
Sus_scrofa
50
45
40
35
30
25
20
15
10
5
0
state 0
state 1

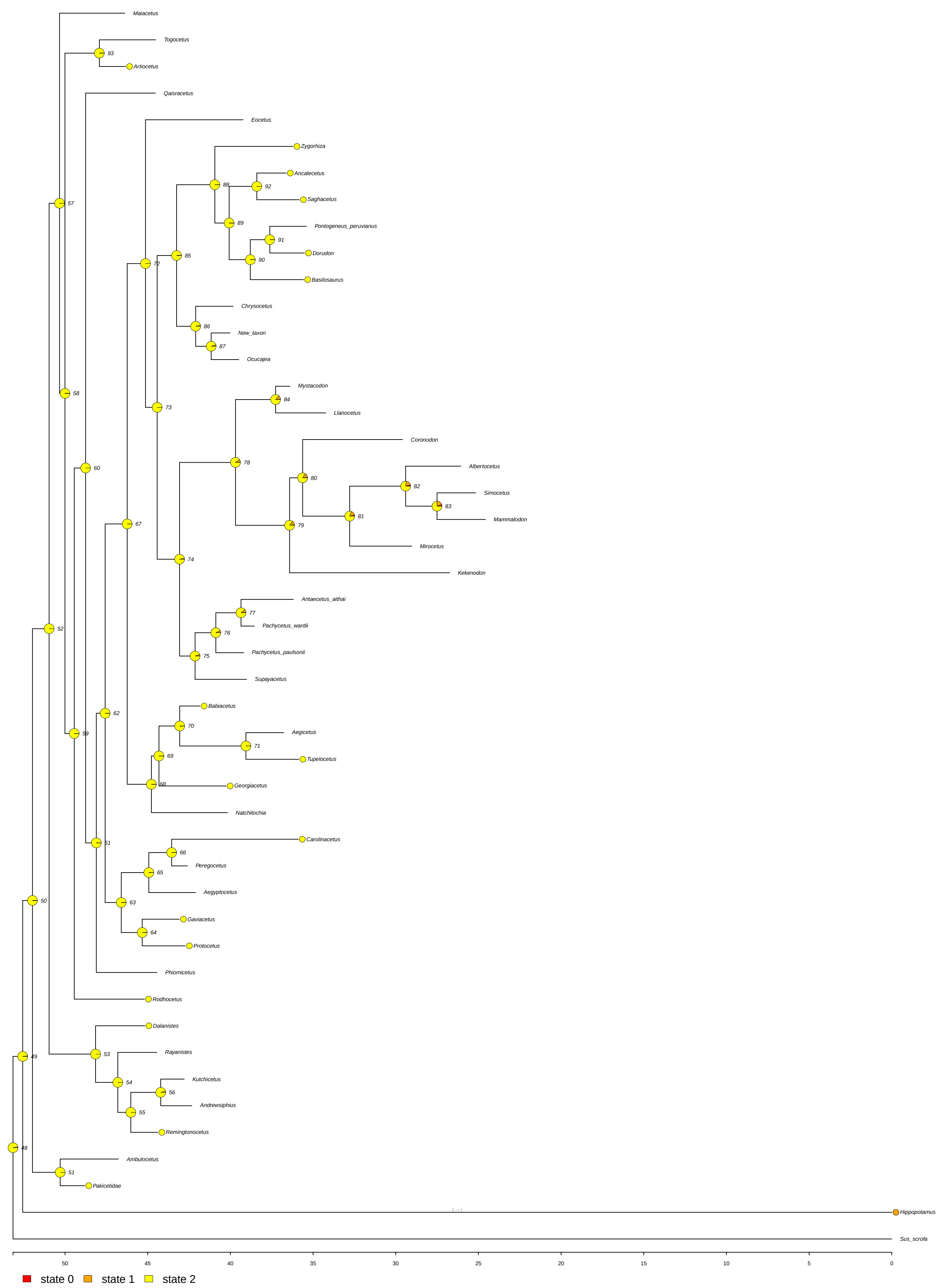

Maiacetus
Togocetus
93
Artiocetus
Qaisracetus
Eocetus
Zygorhiza
Ancalecetus
88
92
Saghacetus
89
Pontogeneus_peruvianus
91
Dorudon
90
Basilosaurus
72
85
Chrysocetus
86
New_taxon
87
Ocucajea
57
58
Mystacodon
84
Llanocetus
73
Coronodon
60
78
Albertocetus
80
82
Simocetus
83
81
Mammalodon
67
79
Mirocetus
74
Kekenodon
Antaecetus_aithai
77
Pachycetus_wardii
52
76
Pachycetus_paulsonii
75
Supayacetus
Babiacetus
62
70
Aegicetus
59
71
Tupelocetus
69
68
Georgiacetus
Natchitochia
Carolinacetus
61
66
Peregocetus
65
Aegyptocetus
50
63
Gaviacetus
64
Protocetus
Phiomicetus
Rodhocetus
Dalanistes
49
53
Rayanistes
54
Kutchicetus
56
Andrewsiphius
55
Remingtonocetus
48
Ambulocetus
51
Pakicetidae
Hippopotamus
Sus_scrofa
50
45
40
35
30
25
20
15
10
5
0
state 0
state 1
state 2

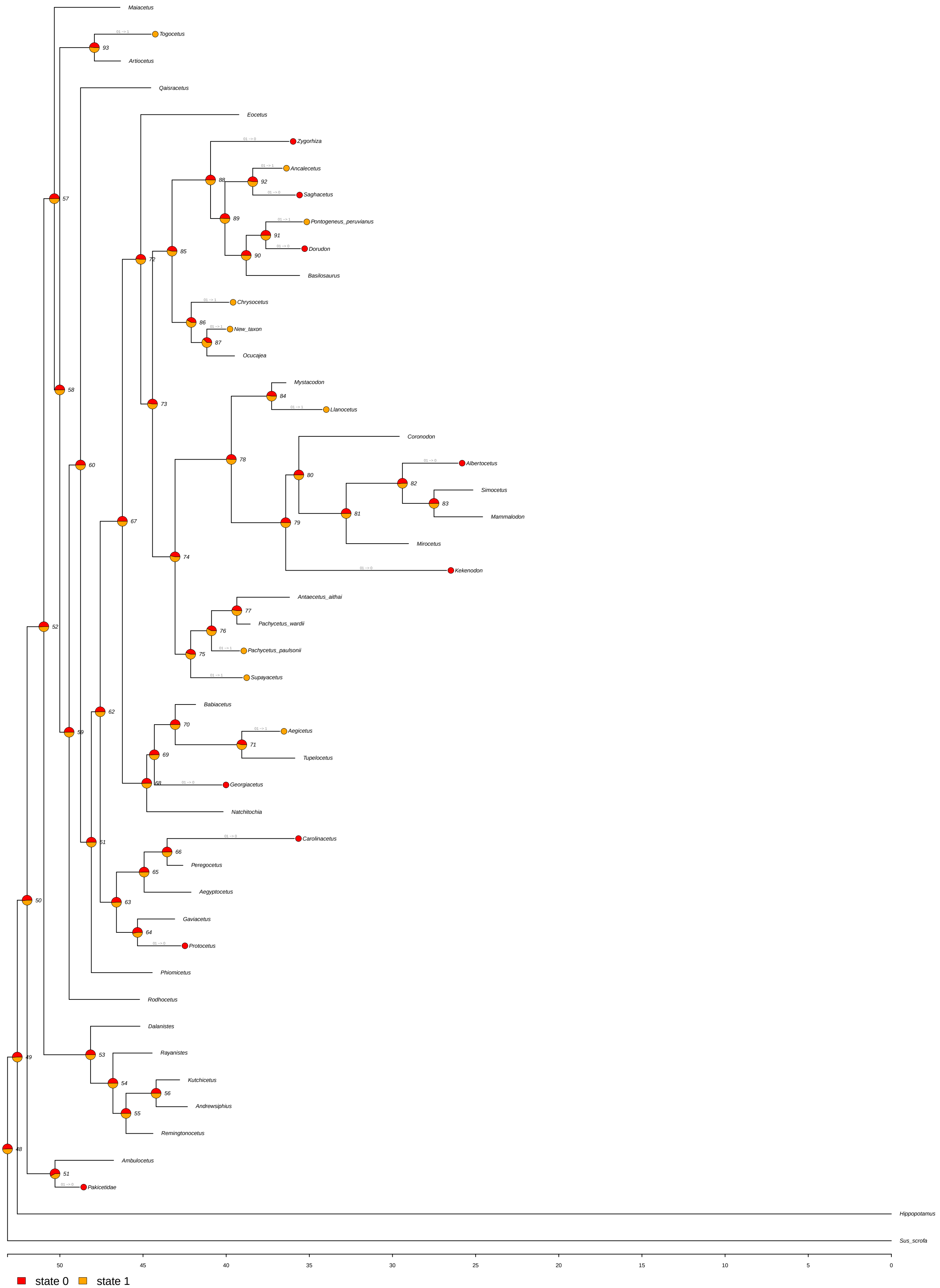
Maiacetus
Togocetus
Artiocetus
Qaisracetus
Eocetus
Zygorhiza
Ancalecetus
Saghacetus
Pontogeneus_peruvianus
Dorudon
Basilosaurus
Chrysocetus
New_taxon
Ocucajea
Mystacodon
Llanocetus
Coronodon
Albertocetus
Simocetus
Mammalodon
Mirocetus
Kekenodon
Antaecetus_aithai
Pachycetus_wardii
Pachycetus_paulsonii
Supayacetus
Babiacetus
Aegicetus
Tupelocetus
Georgiacetus
Natchitochia
Carolinacetus
Peregocetus
Aegyptocetus
Gaviacetus
Protocetus
Phiomicetus
Rodhocetus
Dalanistes
Rayanistes
Kutchicetus
Andrewsiphius
Remingtonocetus
Ambulocetus
Pakicetidae
Hippopotamus
Sus_scrofa
50 45 40 35 30 25 20 15 10 5 0
state 0
state 1

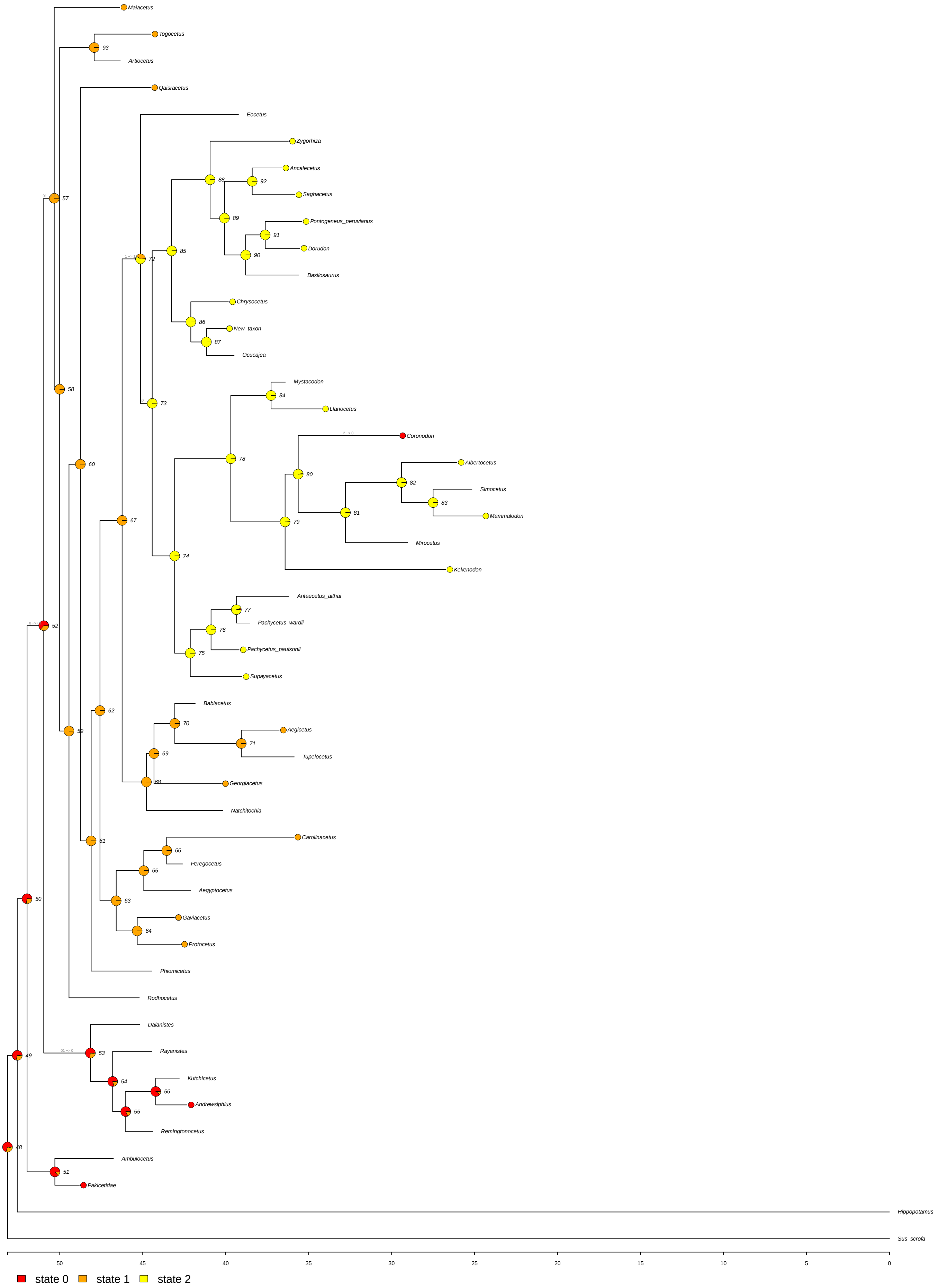
Maiacetus
Togocetus
93
Artiocetus
Qaisracetus
Eocetus
Zygorhiza
Ancalecetus
88
92
Saghacetus
89
Pontogeneus_peruvianus
91
Dorudon
85
90
72
Basilosaurus
Chrysocetus
86
New_taxon
87
Ocucajea
Mystacodon
84
Llanocetus
73
2 --> 0
Coronodon
78
80
Albertocetus
82
Simocetus
83
Mammalodon
81
79
Mirocetus
74
Kekenodon
Antaecetus_aithai
77
Pachycetus_wardii
76
Pachycetus_paulsonii
75
Supayacetus
Babiacetus
70
Aegicetus
71
Tupelocetus
69
68
Georgiacetus
Natchitochia
Carolinacetus
66
Peregocetus
65
Aegyptocetus
63
Gaviacetus
64
Protocetus
Phiomicetus
Rodhocetus
Dalanistes
01 --> 0
53
Rayanistes
54
Kutchicetus
56
Andrewsiphius
55
Remingtonocetus
48
Ambulocetus
51
Pakicetidae
Hippopotamus
Sus_scrofa
57
58
60
67
52
62
59
61
50
49
50
45
40
35
30
25
20
15
10
5
0
state 0
state 1
state 2

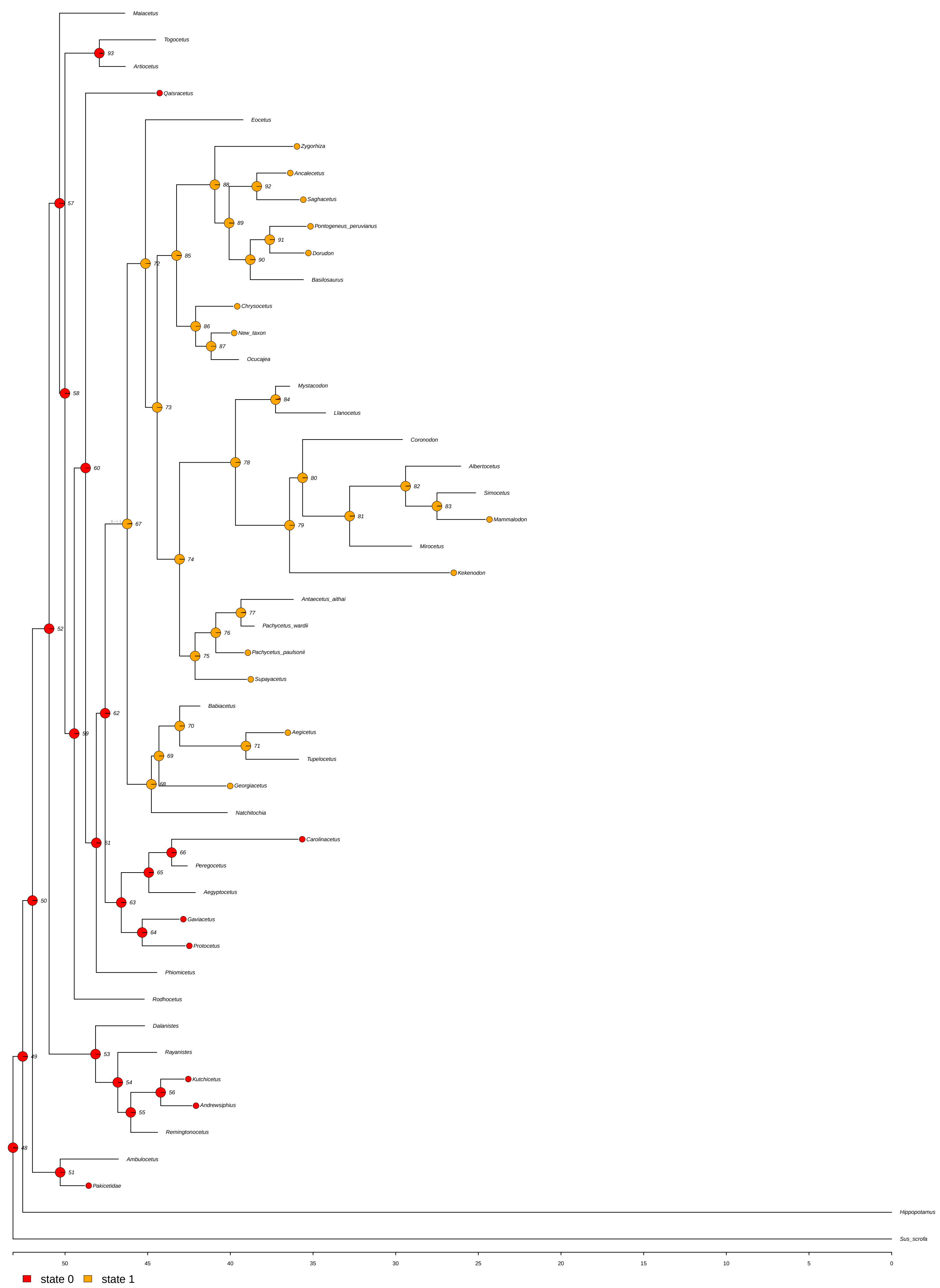

Maiacetus
Togocetus
93
Artiocetus
Qaisracetus
Eocetus
Zygorhiza
Ancalecetus
88
92
Saghacetus
Pontogeneus_peruvianus
89
91
Dorudon
90
Basilosaurus
57
72
85
Chrysocetus
86
New_taxon
87
Ocucajea
Mystacodon
84
58
Llanocetus
73
Coronodon
60
78
80
Albertocetus
82
Simocetus
83
Mammalodon
81
67
79
Mirocetus
74
Kekenodon
Antaecetus_aithai
77
76
Pachycetus_wardii
52
Pachycetus_paulsonii
75
Supayacetus
Babiacetus
62
70
Aegicetus
59
71
Tupelocetus
69
68
Georgiacetus
Natchitochia
Carolinacetus
61
66
Peregocetus
65
Aegyptocetus
50
63
Gaviacetus
64
Protocetus
Phiomicetus
Rodhocetus
Dalanistes
53
Rayanistes
49
54
Kutchicetus
56
Andrewsiphius
55
Remingtonocetus
48
Ambulocetus
51
Pakicetidae
Hippopotamus
Sus_scrofa
50
45
40
35
30
25
20
15
10
5
0
state 0
state 1

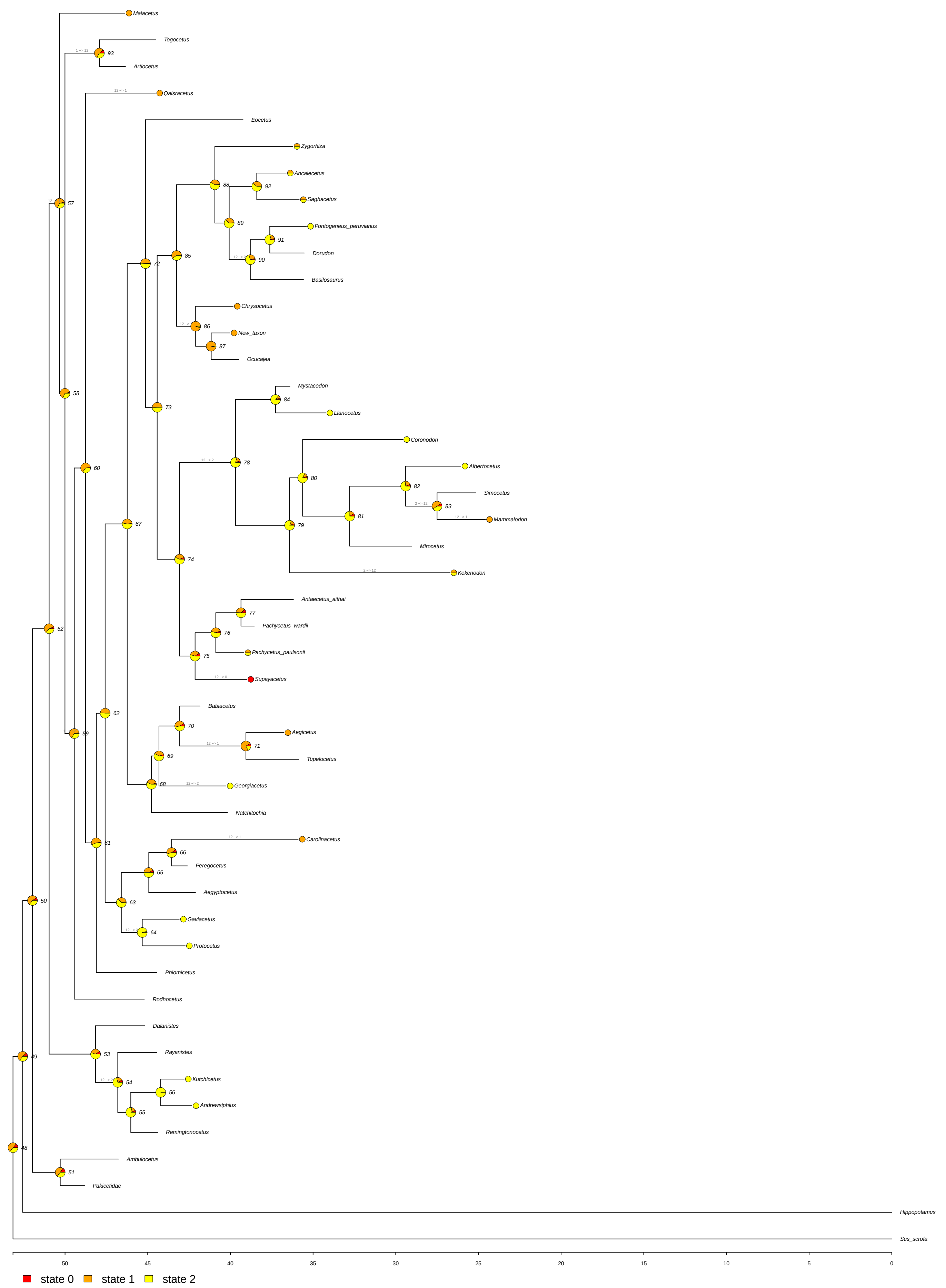

Maiacetus
Togocetus
93
Artiocetus
Qaisracetus
Eocetus
Zygorhiza
Ancalecetus
88
92
Saghacetus
57
Pontogeneus_peruvianus
89
91
Dorudon
72
85
90
Basilosaurus
Chrysocetus
86
New_taxon
87
Ocucajea
Mystacodon
58
84
73
Llanocetus
Coronodon
60
78
Albertocetus
80
82
Simocetus
83
67
79
81
Mammalodon
Mirocetus
74
Kekenodon
Antaecetus_aithai
77
52
Pachycetus_wardii
76
Pachycetus_paulsonii
75
Supayacetus
Babiacetus
62
59
70
Aegicetus
71
69
Tupelocetus
68
Georgiacetus
Natchitochia
61
Carolinacetus
66
Peregocetus
65
50
Aegyptocetus
63
Gaviacetus
64
Protocetus
Phiomicetus
Rodhocetus
Dalanistes
53
Rayanistes
49
54
Kutchicetus
56
Andrewsiphius
55
Remingtonocetus
48
Ambulocetus
51
Pakicetidae
Hippopotamus
Sus_scrofa
50
45
40
35
30
25
20
15
10
5
0
state 0
state 1
state 2

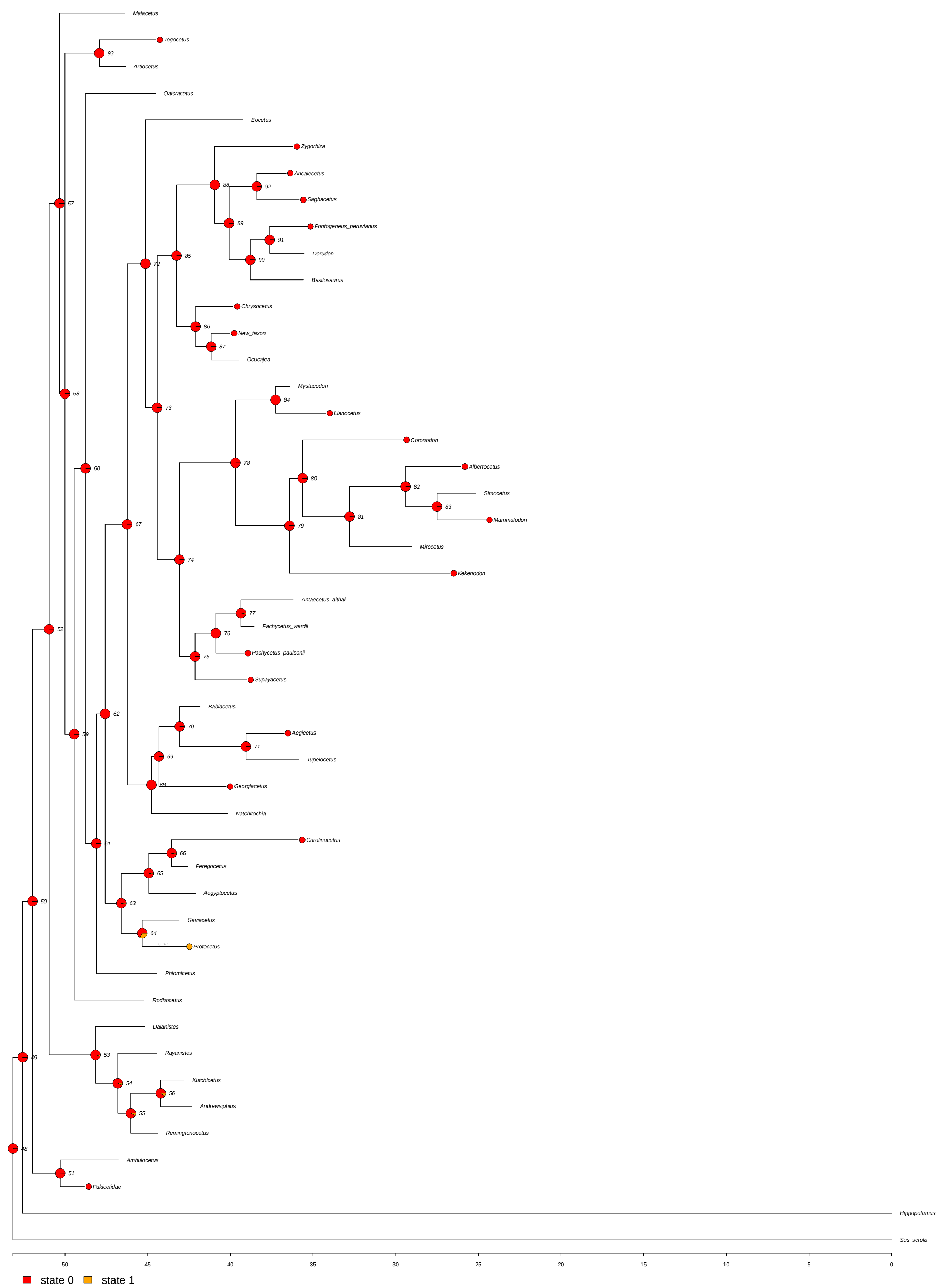

Maiacetus
Togocetus
93
Artiocetus
Qaisracetus
Eocetus
Zygorhiza
Ancalecetus
88
92
Saghacetus
Pontogeneus_peruvianus
89
91
Dorudon
90
Basilosaurus
57
72
85
Chrysocetus
86
New_taxon
87
Ocucajea
Mystacodon
84
58
Llanocetus
73
Coronodon
60
78
Albertocetus
80
82
Simocetus
67
83
81
Mammalodon
79
Mirocetus
74
Kekenodon
Antaecetus_aithai
77
52
Pachycetus_wardii
76
Pachycetus_paulsonii
75
Supayacetus
Babiacetus
62
70
Aegicetus
59
71
Tupelocetus
69
68
Georgiacetus
Natchitochia
Carolinacetus
61
66
Peregocetus
65
Aegyptocetus
50
63
Gaviacetus
64
Protocetus
Phiomicetus
Rodhocetus
Dalanistes
53
Rayanistes
49
Kutchicetus
54
56
Andrewsiphius
55
Remingtonocetus
48
Ambulocetus
51
Pakicetidae
Hippopotamus
Sus_scrofa
50
45
40
35
30
25
20
15
10
5
0
state 0
state 1

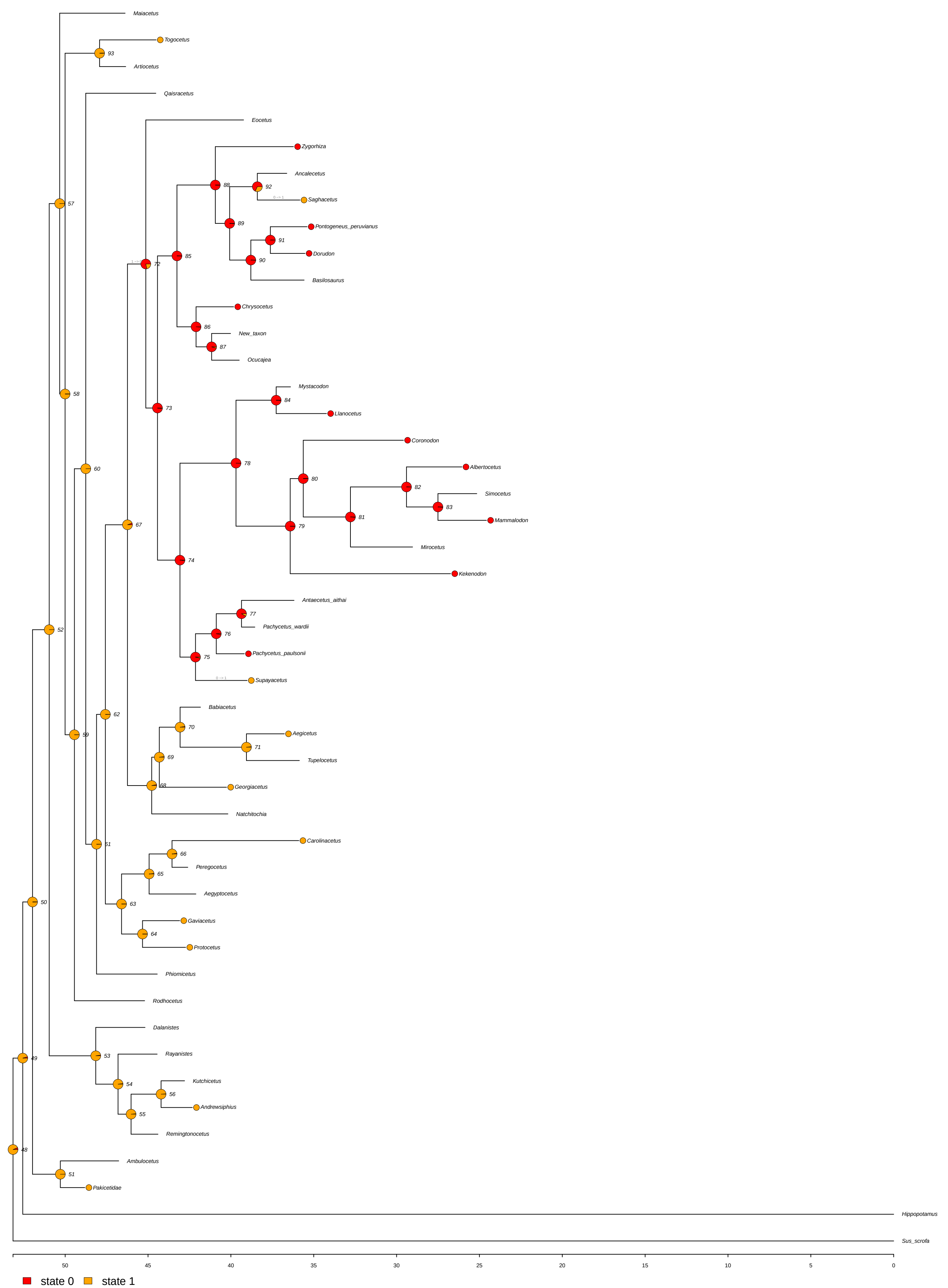
Maiacetus
Togocetus
Artiocetus
Qaisracetus
Eocetus
Zygorhiza
Ancalecetus
Saghacetus
Pontogeneus_peruvianus
Dorudon
Basilosaurus
Chrysocetus
New_taxon
Ocucajea
Mystacodon
Llanocetus
Coronodon
Albertocetus
Simocetus
Mammalodon
Mirocetus
Kekenodon
Antaecetus_aithai
Pachycetus_wardii
Pachycetus_paulsonii
Supayacetus
Babiacetus
Aegicetus
Tupelocetus
Georgiacetus
Natchitochia
Carolinacetus
Peregocetus
Aegyptocetus
Gaviacetus
Protocetus
Phiomicetus
Rodhocetus
Dalanistes
Rayanistes
Kutchicetus
Andrewsiphius
Remingtonocetus
Ambulocetus
Pakicetidae
Hippopotamus
Sus_scrofa
50
45
40
35
30
25
20
15
10
5
0
state 0
state 1

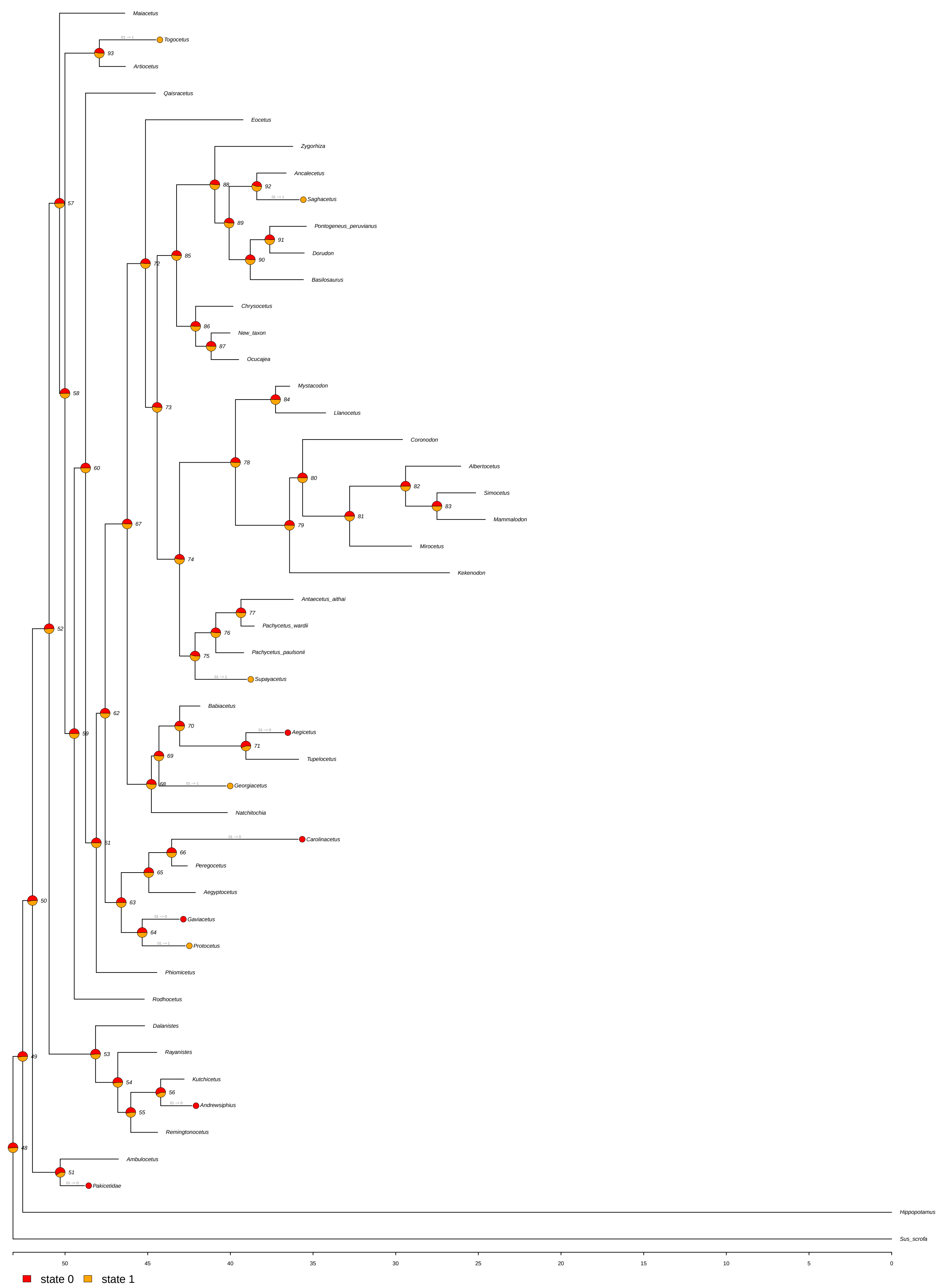

Maiacetus
Togocetus
93
Artiocetus
Qaisracetus
Eocetus
Zygorhiza
Ancalecetus
88
92
Saghacetus
Pontogeneus_peruvianus
89
91
Dorudon
90
Basilosaurus
57
72
85
Chrysocetus
86
New_taxon
87
Ocucajea
Mystacodon
84
Llanocetus
58
73
Coronodon
Albertocetus
60
78
80
82
Simocetus
83
Mammalodon
67
81
79
Mirocetus
74
Kekenodon
Antaecetus_aithai
77
Pachycetus_wardii
76
52
Pachycetus_paulsonii
75
Supayacetus
Babiacetus
62
59
70
Aegicetus
71
Tupelocetus
69
68
Georgiacetus
Natchitochia
Carolinacetus
61
66
Peregocetus
65
Aegyptocetus
50
63
Gaviacetus
64
Protocetus
Phiomicetus
Rodhocetus
Dalanistes
Rayanistes
53
49
Kutchicetus
54
56
Andrewsiphius
55
Remingtonocetus
48
Ambulocetus
51
Pakicetidae
Hippopotamus
Sus_scrofa
50
45
40
35
30
25
20
15
10
5
0
state 0
state 1

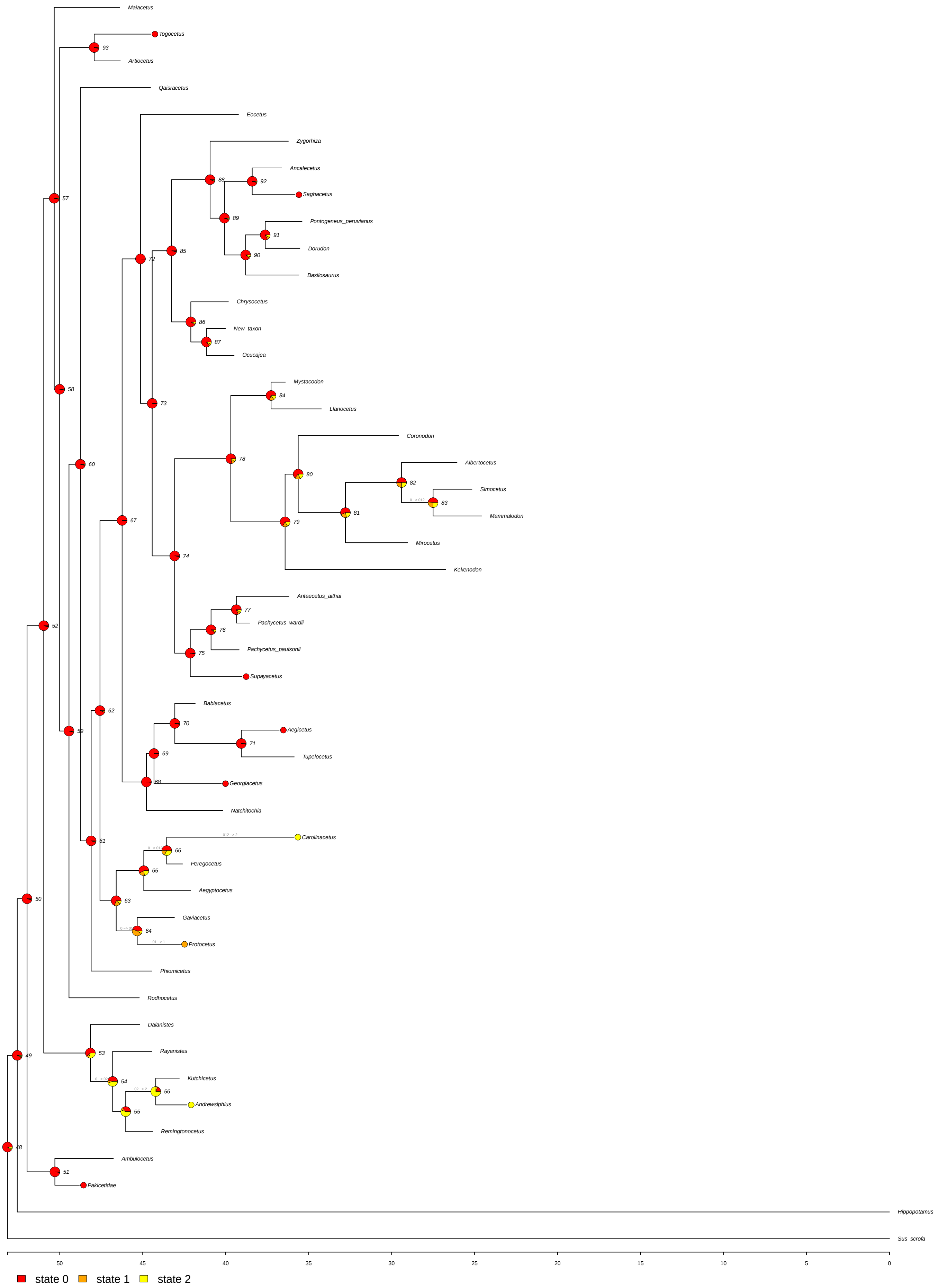

Maiacetus
Togocetus
93
Artiocetus
Qaisracetus
Eocetus
Zygorhiza
Ancalecetus
92
88
Saghacetus
57
89
Pontogeneus_peruvianus
91
Dorudon
90
Basilosaurus
72
85
Chrysocetus
86
New_taxon
87
Ocucajea
Mystacodon
84
Llanocetus
58
73
Coronodon
60
78
Albertocetus
80
82
Simocetus
0 -> 012
83
Mammalodon
81
67
79
Mirocetus
74
Kekenodon
Antaecetus_aithai
77
Pachycetus_wardii
76
52
Pachycetus_paulsonii
75
Supayacetus
Babiacetus
62
59
70
Aegicetus
71
69
Tupelocetus
68
Georgiacetus
Natchitochia
61
012 -> 2
Carolinacetus
66
Peregocetus
65
Aegyptocetus
50
63
Gaviacetus
64
01 -> 1
Protocetus
Phiomicetus
Rodhocetus
Dalanistes
53
49
Rayanistes
54
Kutchicetus
02 -> 2
56
Andrewsiphius
55
Remingtonocetus
48
Ambulocetus
51
Pakicetidae
Hippopotamus
Sus_scrofa
50
45
40
35
30
25
20
15
10
5
0
state 0
state 1
state 2

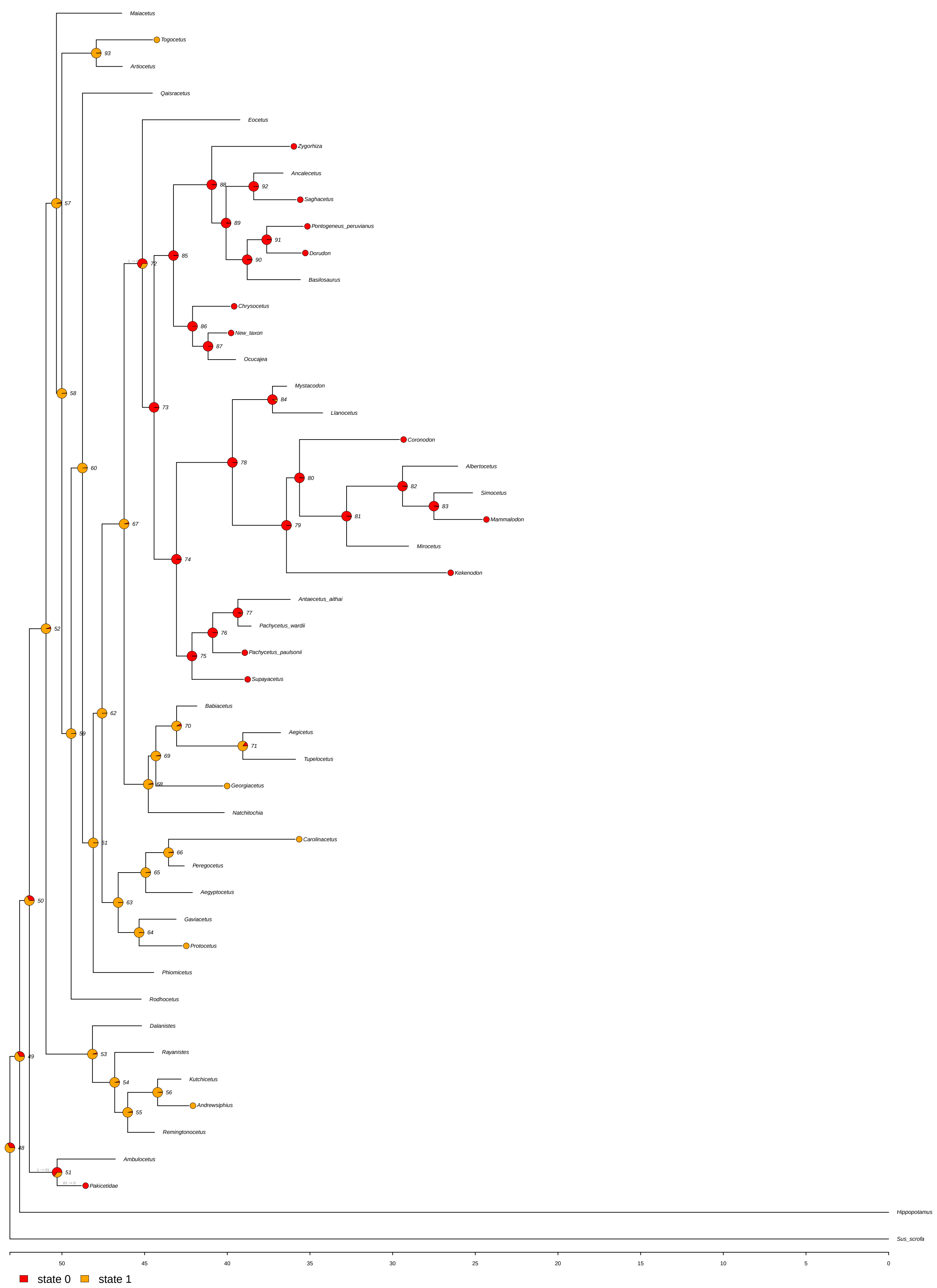

Maiacetus
Togocetus
93
Artiocetus
Qaisracetus
Eocetus
Zygorhiza
88
Ancalecetus
92
Saghacetus
89
Pontogeneus_peruvianus
91
Dorudon
90
Basilosaurus
72
85
57
Chrysocetus
86
New_taxon
87
Ocucajea
Mystacodon
84
Llanocetus
58
73
Coronodon
78
Albertocetus
80
82
Simocetus
60
83
Mammalodon
81
67
79
Mirocetus
74
Kekenodon
Antaecetus_aithai
77
Pachycetus_wardii
52
76
Pachycetus_paulsonii
75
Supayacetus
Babiacetus
62
70
Aegicetus
59
71
Tupelocetus
69
68
Georgiacetus
Natchitochia
Carolinacetus
61
66
Peregocetus
65
Aegyptocetus
50
63
Gaviacetus
64
Protocetus
Phiomicetus
Rodhocetus
Dalanistes
Rayanistes
49
53
Kutchicetus
54
56
Andrewsiphius
55
Remingtonocetus
48
Ambulocetus
51
Pakicetidae
Hippopotamus
Sus_scrofa
50
45
40
35
30
25
20
15
10
5
0
state 0
state 1

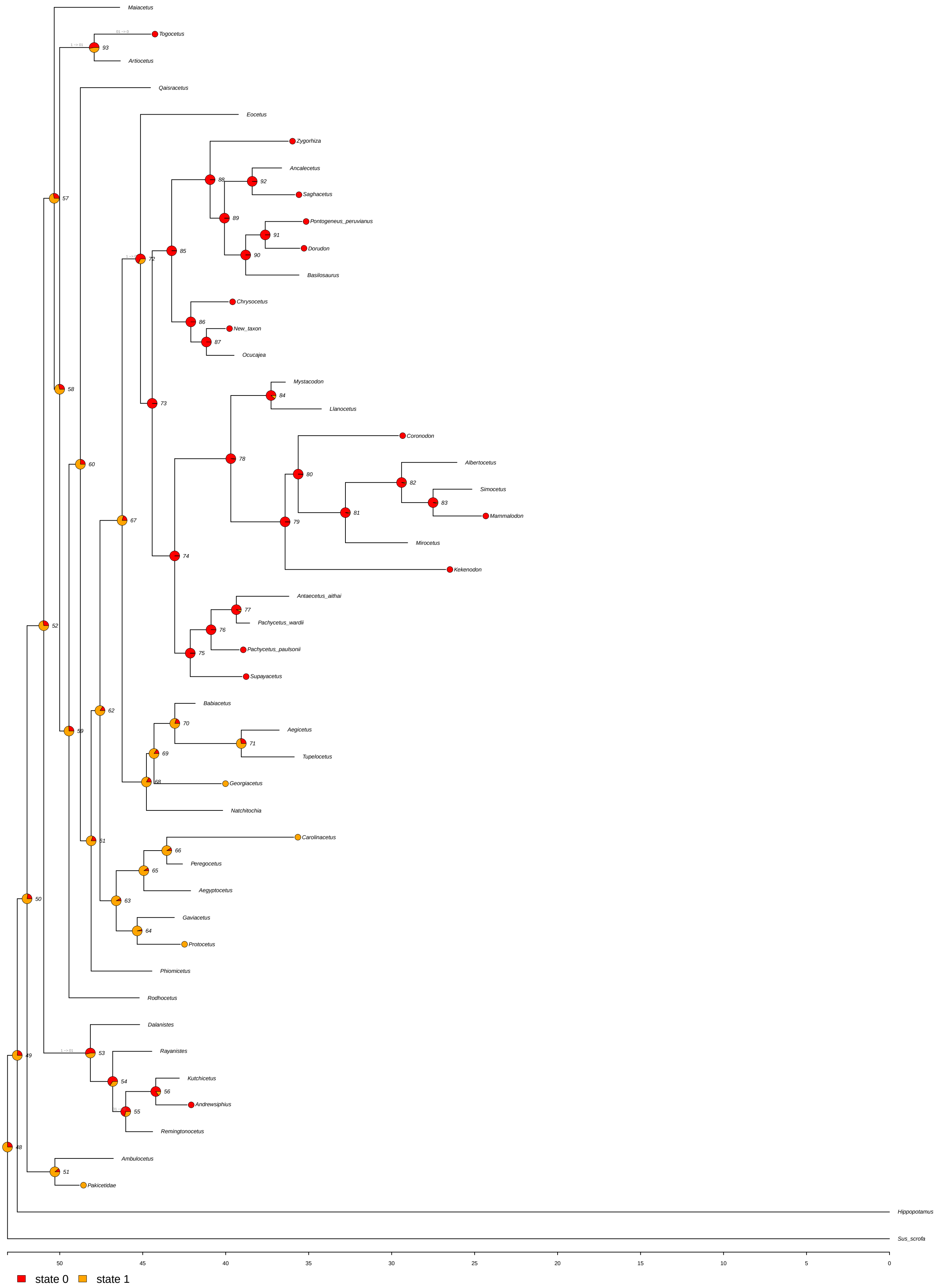

Maiacetus
Togocetus
Artiocetus
Qaisracetus
Eocetus
Zygorhiza
Ancalecetus
Saghacetus
Pontogeneus_peruvianus
Dorudon
Basilosaurus
Chrysocetus
New_taxon
Ocucajea
Mystacodon
Llanocetus
Coronodon
Albertocetus
Simocetus
Mammalodon
Mirocetus
Kekenodon
Antaecetus_aithai
Pachycetus_wardii
Pachycetus_paulsonii
Supayacetus
Babiacetus
Aegicetus
Tupelocetus
Georgiacetus
Natchitochia
Carolinacetus
Peregocetus
Aegyptocetus
Gaviacetus
Protocetus
Phiomicetus
Rodhocetus
Dalanistes
Rayanistes
Kutchicetus
Andrewsiphius
Remingtonocetus
Ambulocetus
Pakicetidae
Hippopotamus
Sus_scrofa
50
45
40
35
30
25
20
15
10
5
0
state 0
state 1

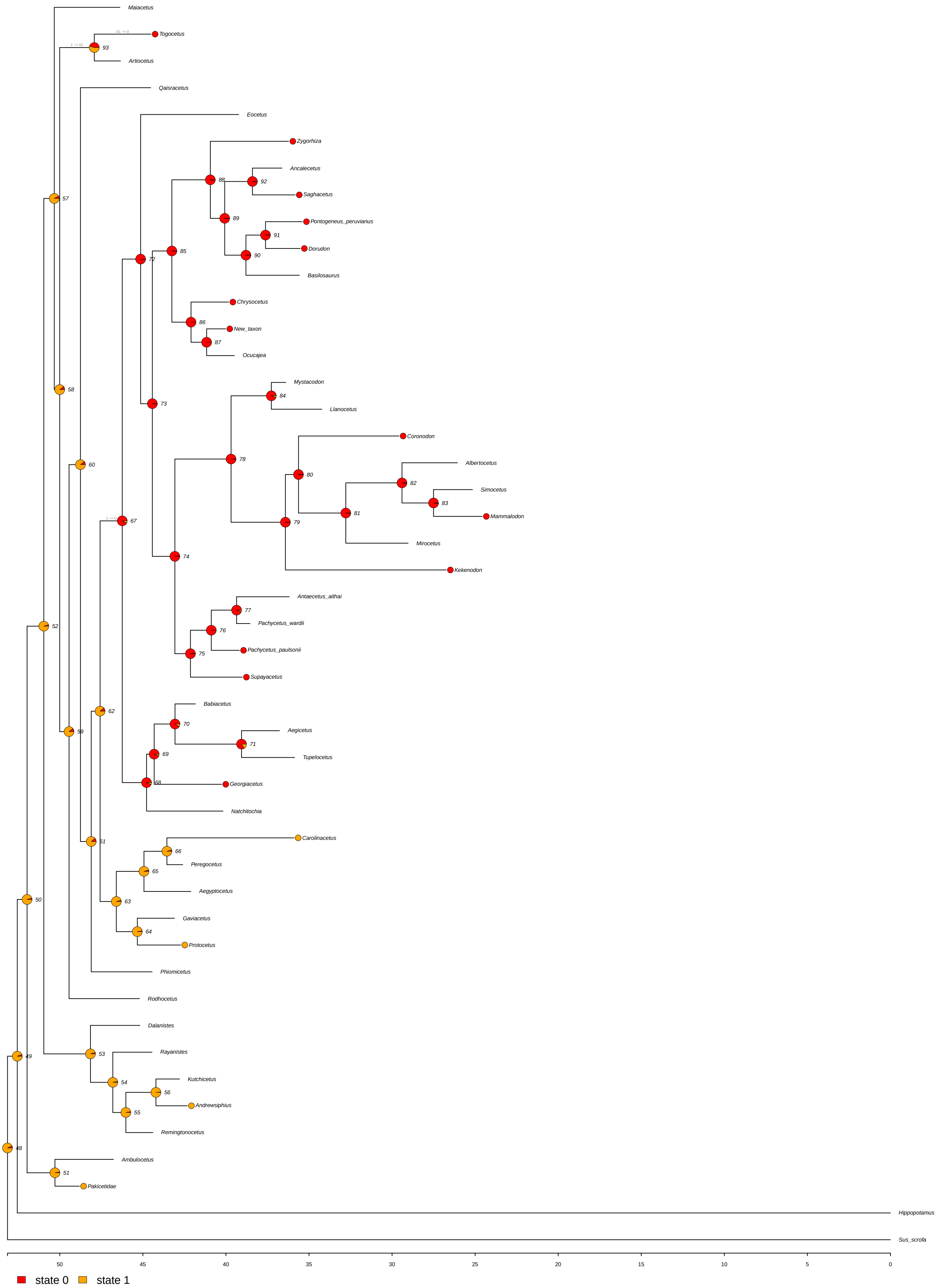
Maiacetus
Togocetus
Artiocetus
Qaisracetus
Eocetus
Zygorhiza
Ancalecetus
Saghacetus
Pontogeneus_peruvianus
Dorudon
Basilosaurus
Chrysocetus
New_taxon
Ocucajea
Mystacodon
Llanocetus
Coronodon
Albertocetus
Simocetus
Mammalodon
Mirocetus
Kekenodon
Antaecetus_aithai
Pachycetus_wardii
Pachycetus_paulsonii
Supayacetus
Babiacetus
Aegicetus
Tupelocetus
Georgiacetus
Natchitochia
Carolinacetus
Peregocetus
Aegyptocetus
Gaviacetus
Protocetus
Phiomicetus
Rodhocetus
Dalanistes
Rayanistes
Kutchicetus
Andrewsiphius
Remingtonocetus
Ambulocetus
Pakicetidae
Hippopotamus
Sus_scrofa
50
45
40
35
30
25
20
15
10
5
0
state 0
state 1

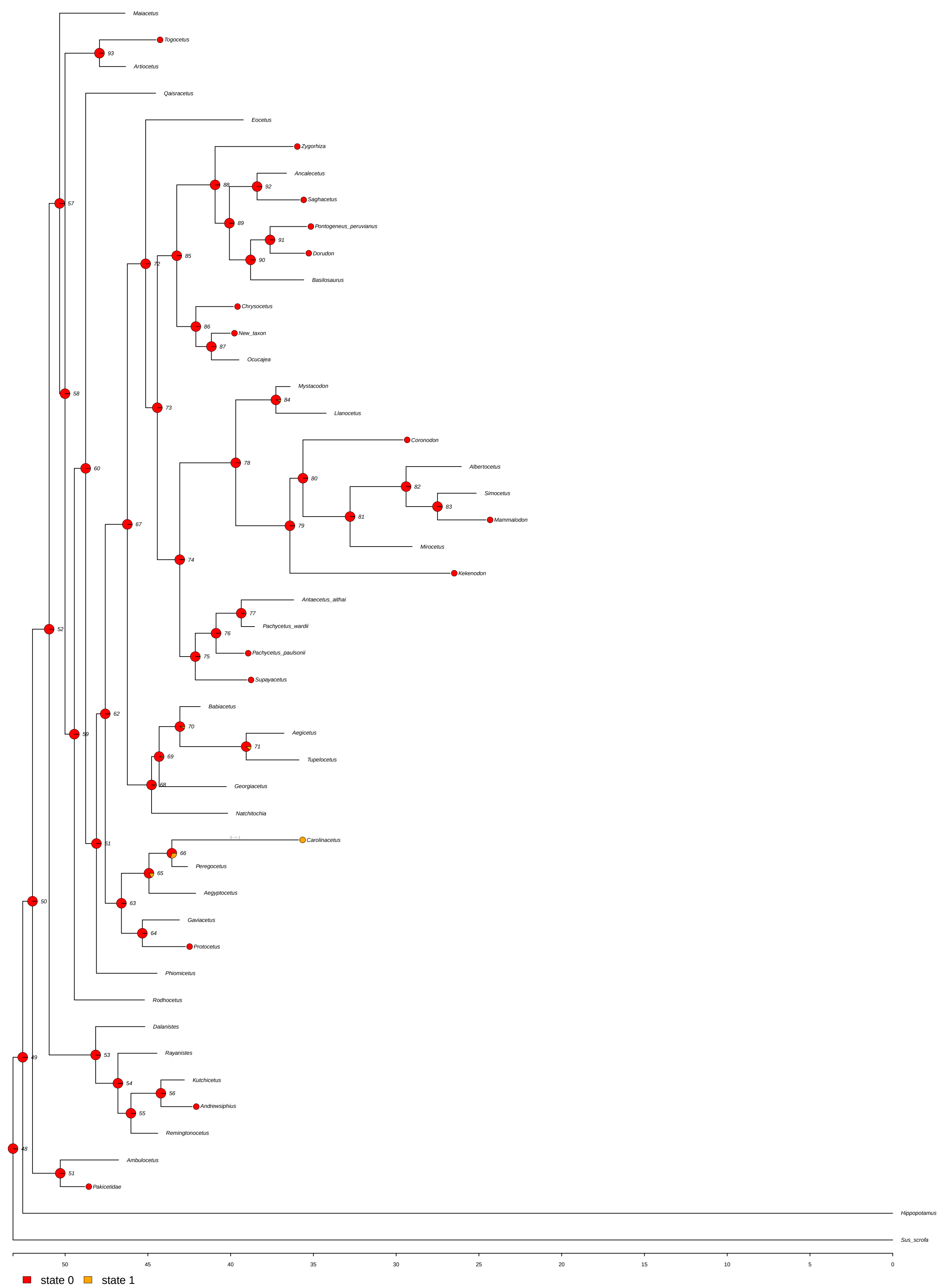

Maiacetus
Togocetus
93
Artiocetus
Qaisracetus
Eocetus
Zygorhiza
Ancalecetus
88
92
Saghacetus
Pontogeneus_peruvianus
89
91
Dorudon
90
57
Basilosaurus
72
85
Chrysocetus
86
New_taxon
87
Ocucajea
Mystacodon
84
Llanocetus
58
73
Coronodon
78
Albertocetus
80
82
Simocetus
83
Mammalodon
81
60
67
79
Mirocetus
74
Kekenodon
Antaecetus_aithai
77
Pachycetus_wardii
76
52
Pachycetus_paulsonii
75
Supayacetus
Babiacetus
62
70
Aegicetus
59
71
Tupelocetus
69
68
Georgiacetus
Natchitochia
0 --> 1
Carolinacetus
61
66
Peregocetus
65
Aegyptocetus
50
63
Gaviacetus
64
Protocetus
Phiomicetus
Rodhocetus
Dalanistes
53
Rayanistes
49
54
Kutchicetus
56
Andrewsiphius
55
Remingtonocetus
48
Ambulocetus
51
Pakicetidae
Hippopotamus
Sus_scrofa
50
45
40
35
30
25
20
15
10
5
0
state 0
state 1

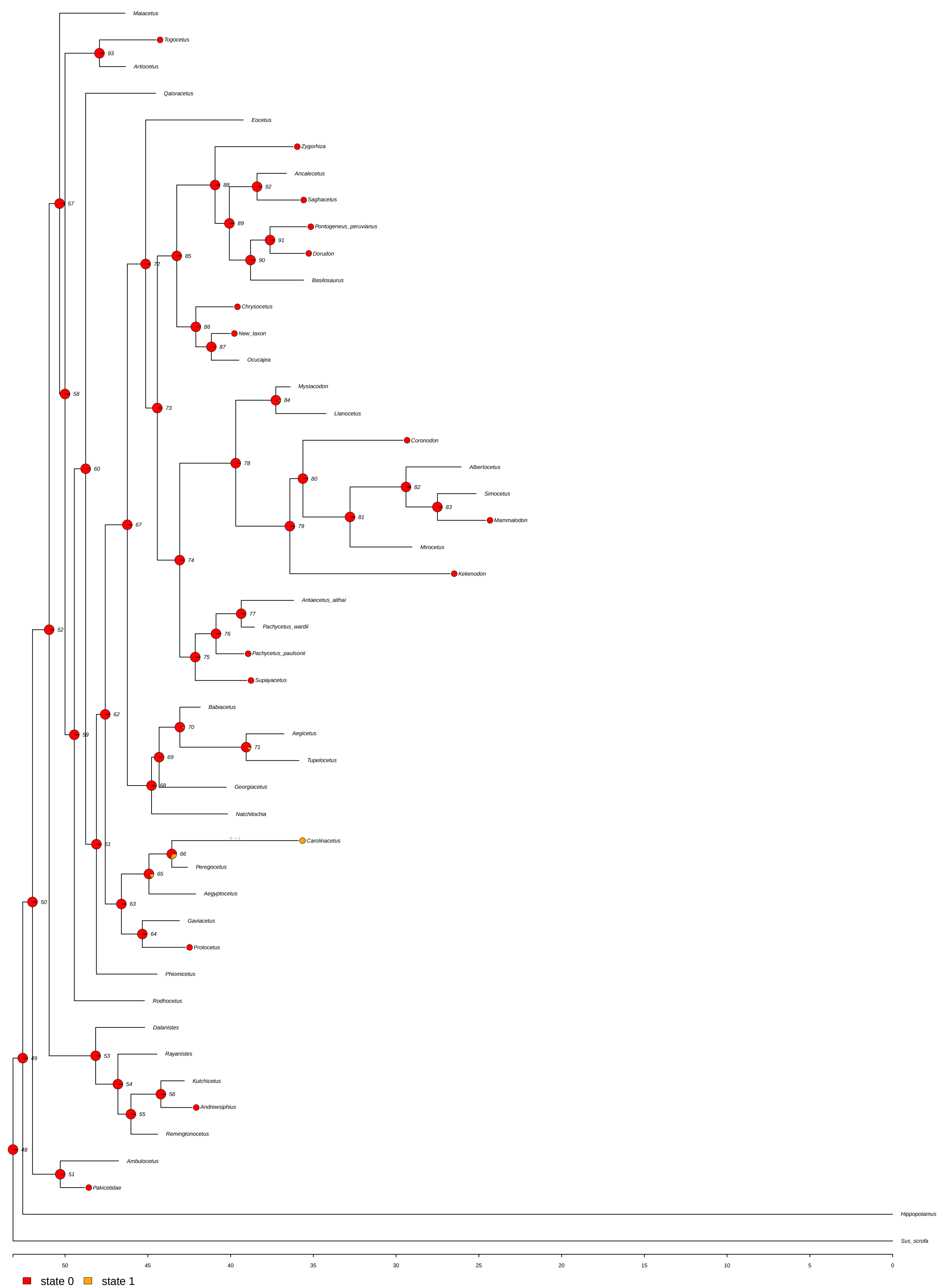

Maiacetus
Togocetus
Artiocetus
Qaisracetus
Eocetus
Zygorhiza
Ancalecetus
Saghacetus
Pontogeneus_peruvianus
Dorudon
Basilosaurus
Chrysocetus
New_taxon
Ocucajea
Mystacodon
Llanocetus
Coronodon
Albertocetus
Simocetus
Mammalodon
Mirocetus
Kekenodon
Antaecetus_aithai
Pachycetus_wardii
Pachycetus_paulsonii
Supayacetus
Babiacetus
Aegicetus
Tupelocetus
Georgiacetus
Natchitochia
Carolinacetus
Peregocetus
Aegyptocetus
Gaviacetus
Protocetus
Phiomicetus
Rodhocetus
Dalanistes
Rayanistes
Kutchicetus
Andrewsiphius
Remingtonocetus
Ambulocetus
Pakicetidae
Hippopotamus
Sus_scrofa
50
45
40
35
30
25
20
15
10
5
0
state 0
state 1

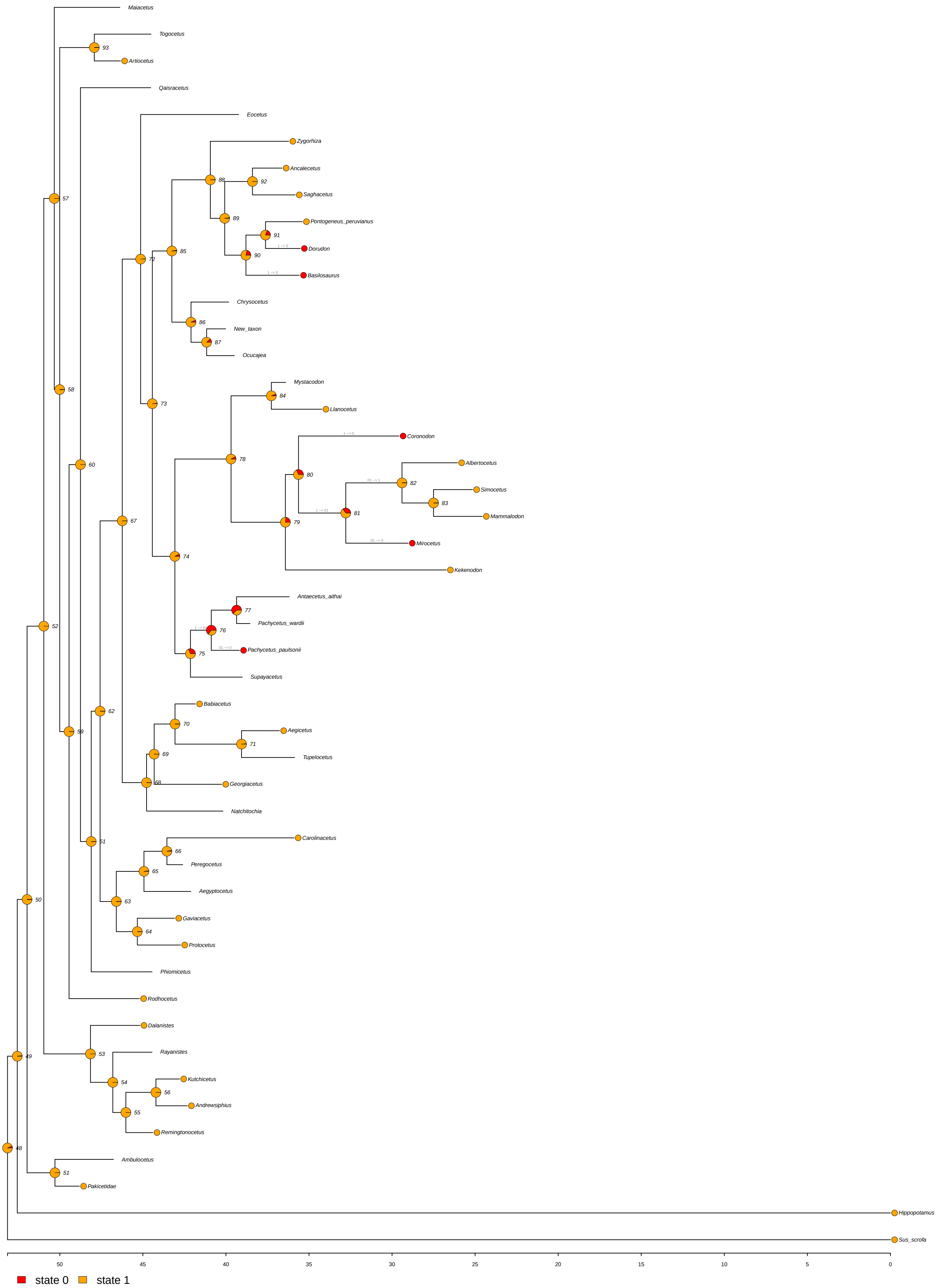

Maiacetus
Togocetus
93
Artiocetus
Qaisracetus
Eocetus
Zygorhiza
Ancalecetus
88
92
Saghacetus
89
Pontogeneus_peruvianus
91
1 -> 0
Dorudon
90
1 -> 0
Basilosaurus
57
72
85
Chrysocetus
86
New_taxon
87
Ocucajea
Mystacodon
84
Llanocetus
58
73
1 -> 0
Coronodon
Albertocetus
60
78
80
01 -> 1
82
Simocetus
83
1 -> 01
81
Mammalodon
67
79
01 -> 0
Mirocetus
74
Kekenodon
Antaecetus_aithai
77
52
1 -> 01
76
Pachycetus_wardii
01 -> 0
Pachycetus_paulsonii
75
Supayacetus
Babiacetus
62
70
59
Aegicetus
71
69
Tupelocetus
68
Georgiacetus
Natchitochia
61
Carolinacetus
66
Peregocetus
65
50
Aegyptocetus
63
Gaviacetus
64
Protocetus
Phiomicetus
Rodhocetus
Dalanistes
49
53
Rayanistes
54
Kutchicetus
56
55
Andrewsiphius
Remingtonocetus
48
Ambulocetus
51
Pakicetidae
Hippopotamus
Sus_scrofa
50
45
40
35
30
25
20
15
10
5
0
state 0
state 1

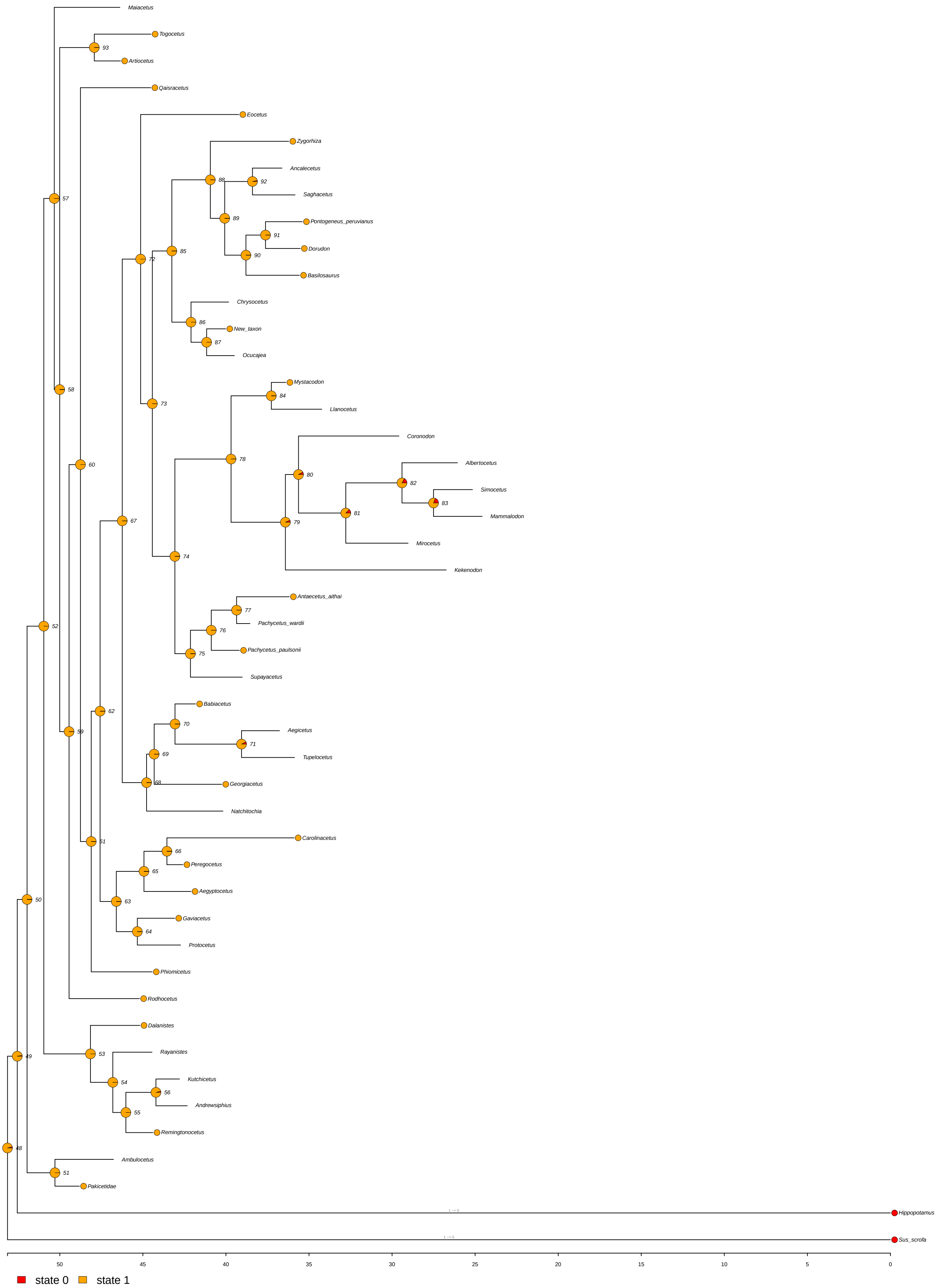

Maiacetus
Togocetus
93
Artiocetus
Qaisracetus
Eocetus
Zygorhiza
Ancalecetus
88
92
Saghacetus
Pontogeneus_peruvianus
89
91
Dorudon
90
Basilosaurus
57
72
85
Chrysocetus
86
New_taxon
87
Ocucajea
Mystacodon
84
Llanocetus
58
73
Coronodon
Albertocetus
60
78
80
82
Simocetus
83
Mammalodon
67
79
81
Mirocetus
74
Kekenodon
Antaecetus_aithai
77
Pachycetus_wardii
76
52
Pachycetus_paulsonii
75
Supayacetus
Babiacetus
62
70
Aegicetus
59
71
Tupelocetus
69
68
Georgiacetus
Natchitochia
Carolinacetus
61
66
Peregocetus
65
Aegyptocetus
50
63
Gaviacetus
64
Protocetus
Phiomicetus
Rodhocetus
Dalanistes
Rayanistes
49
53
54
Kutchicetus
56
Andrewsiphius
55
Remingtonocetus
48
Ambulocetus
51
Pakicetidae
Hippopotamus
Sus_scrofa
50
45
40
35
30
25
20
15
10
5
0
state 0
state 1

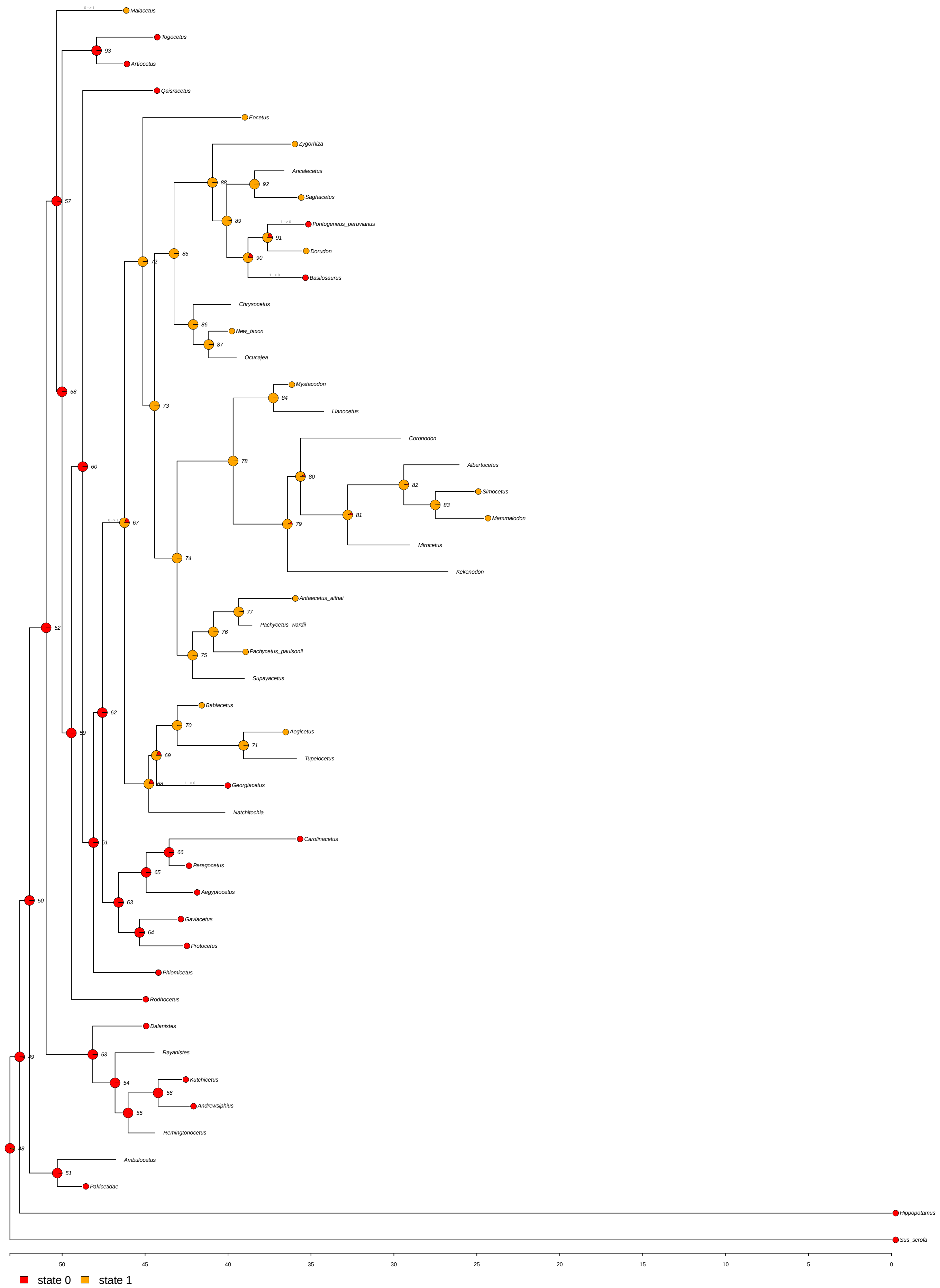

0 -> 1
Maiacetus
Togocetus
93
Artiocetus
Qaisracetus
Eocetus
Zygorhiza
Ancalecetus
88
92
Saghacetus
57
1 -> 0
Pontogeneus_peruvianus
89
91
72
85
90
Dorudon
1 -> 0
Basilosaurus
Chrysocetus
86
New_taxon
87
Ocucajea
Mystacodon
84
58
73
Llanocetus
Coronodon
60
78
Albertocetus
80
82
Simocetus
83
81
Mammalodon
0 -> 1
67
79
Mirocetus
74
Kekenodon
Antaecetus_aithai
77
Pachycetus_wardii
52
76
Pachycetus_paulsonii
75
Supayacetus
Babiacetus
62
70
59
Aegicetus
71
Tupelocetus
69
1 -> 0
68
Georgiacetus
Natchitochia
61
Carolinacetus
66
Peregocetus
65
50
Aegyptocetus
63
Gaviacetus
64
Protocetus
Phiomicetus
Rodhocetus
Dalanistes
49
53
Rayanistes
54
Kutchicetus
56
Andrewsiphius
55
Remingtonocetus
48
Ambulocetus
51
Pakicetidae
Hippopotamus
Sus_scrofa
50
45
40
35
30
25
20
15
10
5
0
state 0
state 1

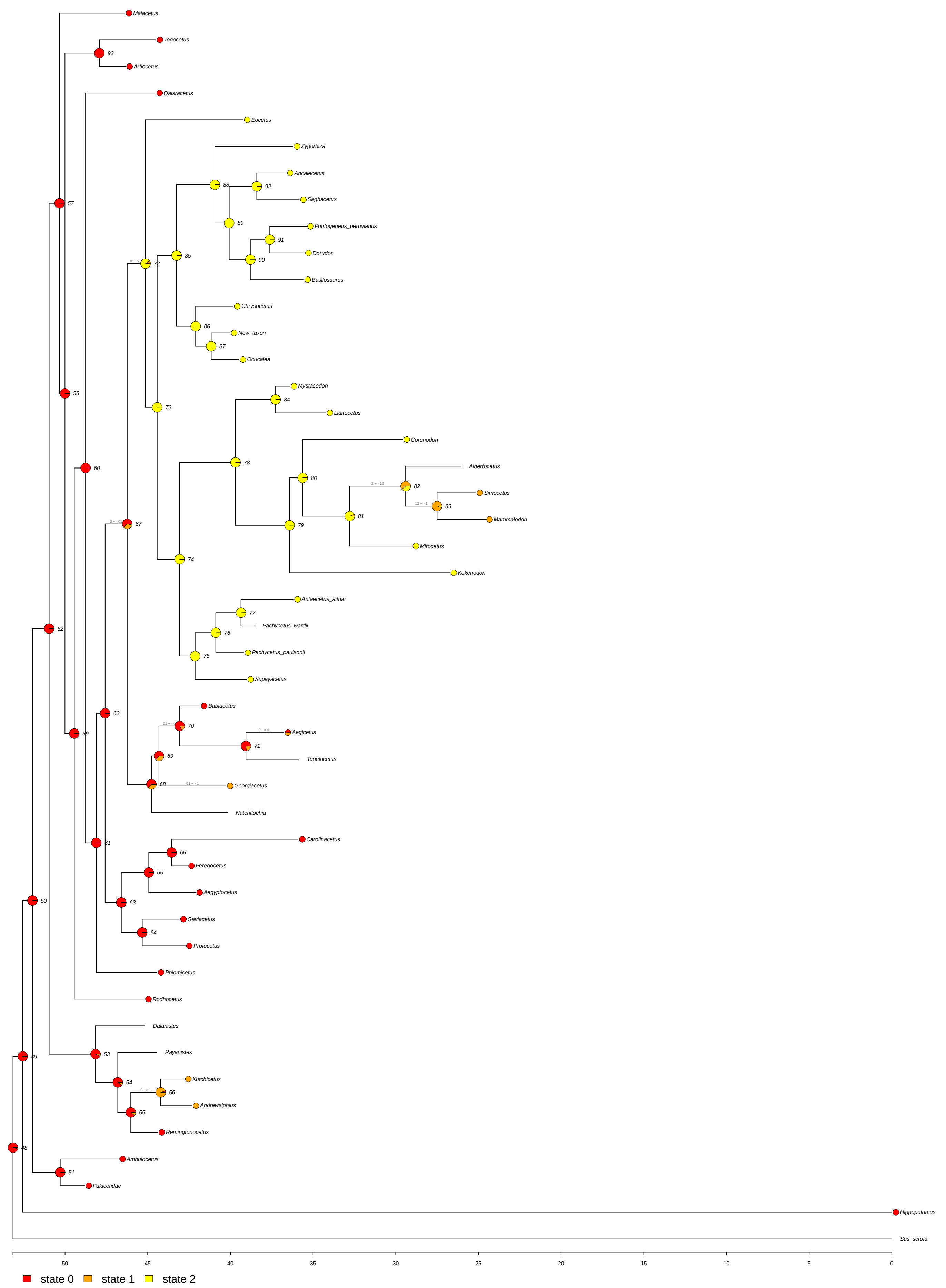

Maiacetus
Togocetus
93
Artiocetus
Qaisracetus
Eocetus
Zygorhiza
Ancalecetus
88
92
Saghacetus
89
Pontogeneus_peruvianus
91
Dorudon
90
Basilosaurus
72
85
Chrysocetus
86
New_taxon
87
Ocucajea
57
58
Mystacodon
84
Llanocetus
73
Coronodon
60
Albertocetus
78
80
2 -> 12
82
Simocetus
12 -> 1
83
Mammalodon
81
67
79
Mirocetus
74
Kekenodon
Antaecetus_aithai
77
Pachycetus_wardii
76
52
Pachycetus_paulsonii
75
Supayacetus
Babiacetus
62
70
0 -> 01
Aegicetus
59
71
Tupelocetus
69
68
01 -> 1
Georgiacetus
Natchitochia
Carolinacetus
61
66
Peregocetus
65
Aegyptocetus
50
63
Gaviacetus
64
Protocetus
Phiomicetus
Rodhocetus
Dalanistes
53
Rayanistes
49
54
Kutchicetus
0 -> 1
56
55
Andrewsiphius
Remingtonocetus
48
Ambulocetus
51
Pakicetidae
Hippopotamus
Sus_scrofa
50
45
40
35
30
25
20
15
10
5
0
state 0
state 1
state 2

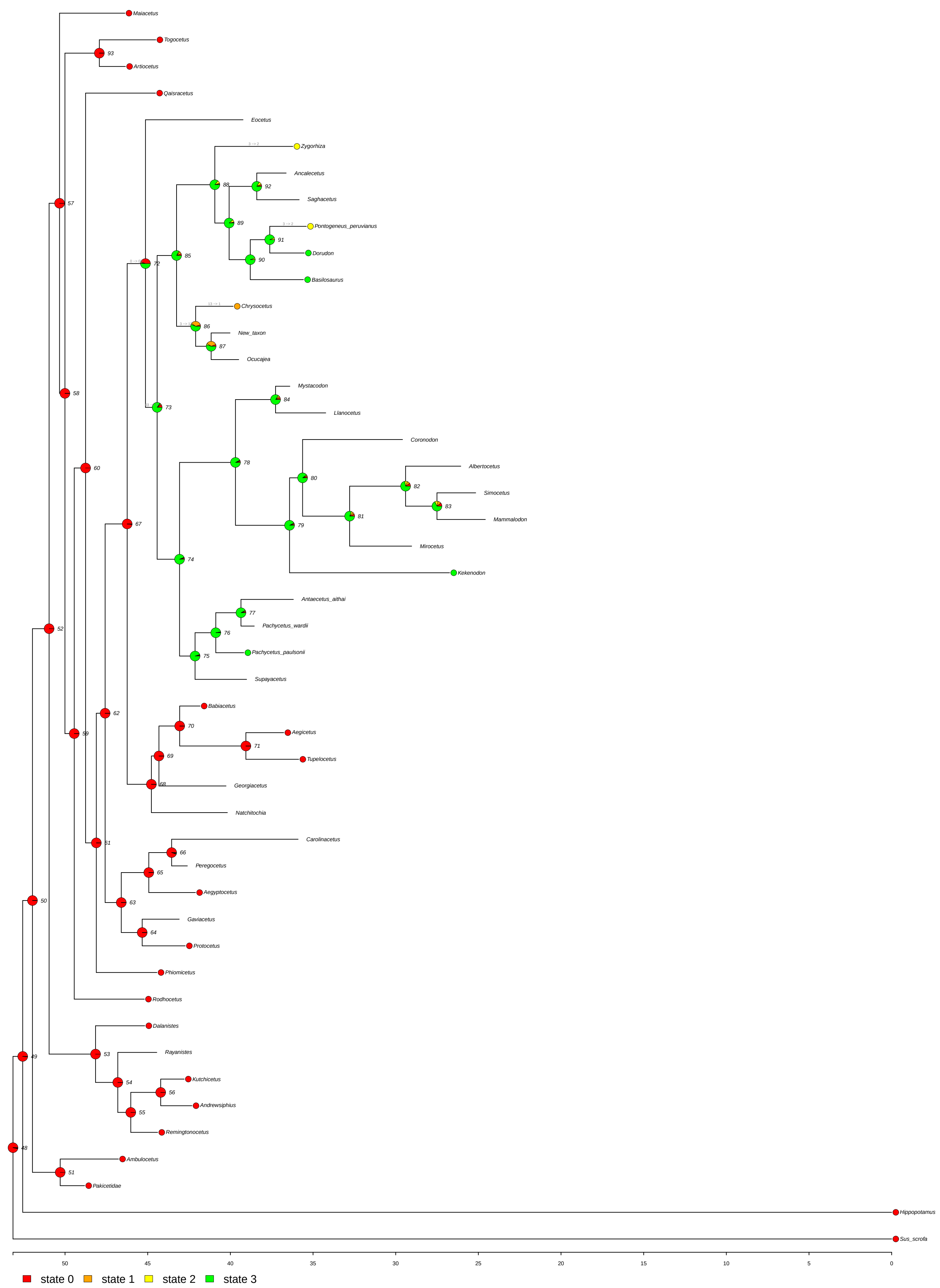

Maiacetus
Togocetus
93
Artiocetus
Qaisracetus
Eocetus
Zygorhiza
3 -> 2
Ancalecetus
88
92
Saghacetus
89
Pontogeneus_peruvianus
3 -> 2
91
Dorudon
90
Basilosaurus
57
72
85
Chrysocetus
86
New_taxon
87
Ocucajea
Mystacodon
84
Llanocetus
58
73
Coronodon
78
Albertocetus
60
80
82
Simocetus
83
Mammalodon
67
81
79
Mirocetus
74
Kekenodon
Antaecetus_aithai
77
Pachycetus_wardii
76
Pachycetus_paulsonii
75
Supayacetus
52
Babiacetus
62
70
Aegicetus
59
71
Tupelocetus
69
68
Georgiacetus
Natchitochia
Carolinacetus
61
66
Peregocetus
65
Aegyptocetus
50
63
Gaviacetus
64
Protocetus
Phiomicetus
Rodhocetus
Dalanistes
Rayanistes
53
49
54
Kutchicetus
56
Andrewsiphius
55
Remingtonocetus
48
Ambulocetus
51
Pakicetidae
Hippopotamus
Sus_scrofa
50
45
40
35
30
25
20
15
10
5
0
state 0
state 1
state 2
state 3

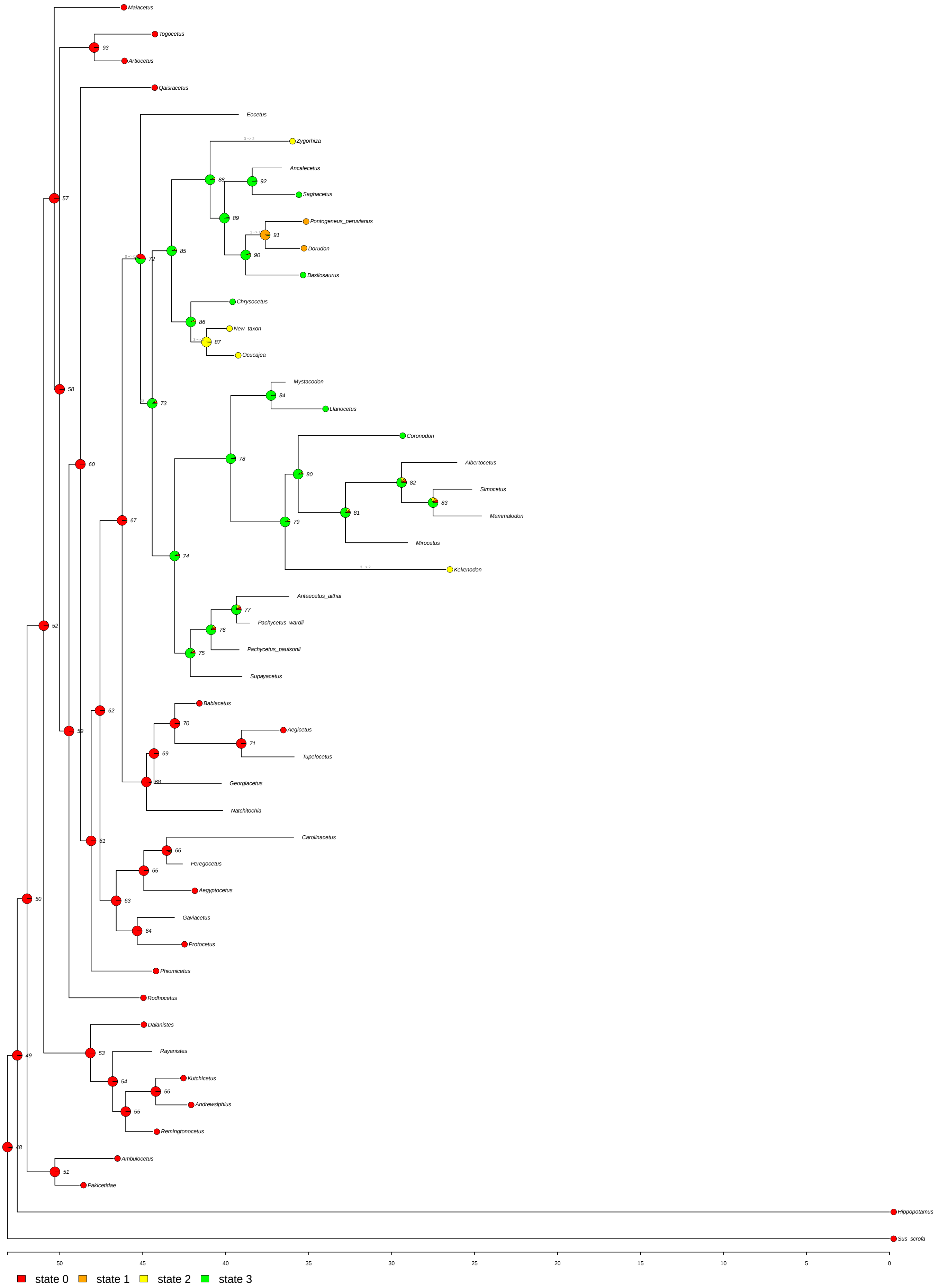
Maiacetus
Togocetus
Artiocetus
Qaisracetus
Eocetus
Zygorhiza
Ancalecetus
Saghacetus
Pontogeneus_peruvianus
Dorudon
Basilosaurus
Chrysocetus
New_taxon
Ocucajea
Mystacodon
Llanocetus
Coronodon
Albertocetus
Simocetus
Mammalodon
Mirocetus
Kekenodon
Antaecetus_aithai
Pachycetus_wardii
Pachycetus_paulsonii
Supayacetus
Babiacetus
Aegicetus
Tupelocetus
Georgiacetus
Natchitochia
Carolinacetus
Peregocetus
Aegyptocetus
Gaviacetus
Protocetus
Phiomicetus
Rodhocetus
Dalanistes
Rayanistes
Kutchicetus
Andrewsiphius
Remingtonocetus
Ambulocetus
Pakicetidae
Hippopotamus
Sus_scrofa
50
45
40
35
30
25
20
15
10
5
0
state 0
state 1
state 2
state 3

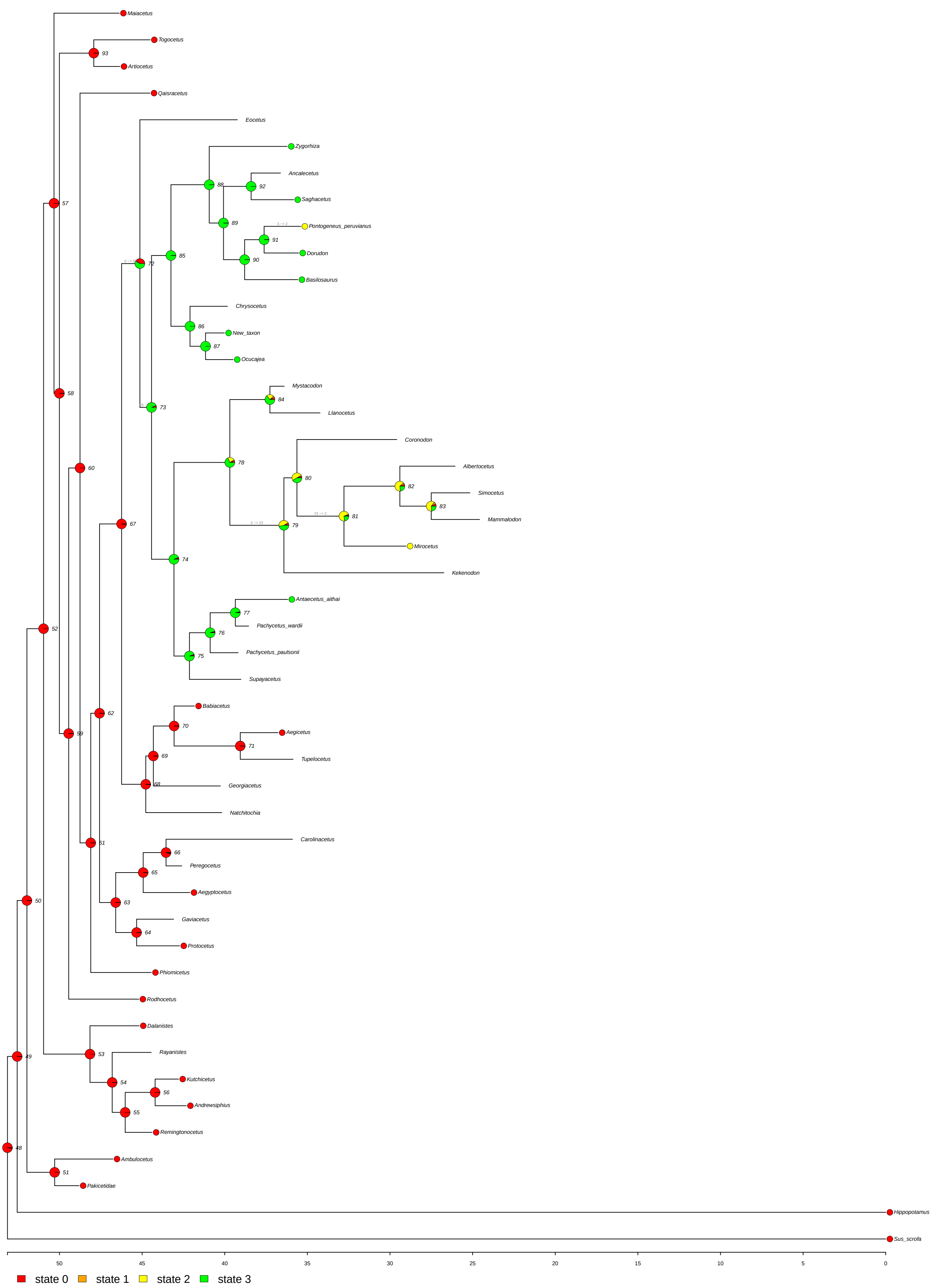
Maiacetus
Togocetus
93
Artiocetus
Qaisracetus
Eocetus
Zygorhiza
Ancalecetus
88
92
Saghacetus
89
3 -> 2
Pontogeneus_peruvianus
91
Dorudon
72
85
90
Basilosaurus
57
Chrysocetus
86
New_taxon
87
Ocucajea
Mystacodon
84
58
Llanocetus
73
Coronodon
Albertocetus
60
78
80
82
Simocetus
83
23 -> 2
81
Mammalodon
67
3 -> 23
79
Mirocetus
74
Kekenodon
Antaecetus_aithai
77
Pachycetus_wardii
76
Pachycetus_paulsonii
52
75
Supayacetus
Babiacetus
62
70
Aegicetus
59
71
Tupelocetus
69
68
Georgiacetus
Natchitochia
Carolinacetus
61
66
Peregocetus
65
Aegyptocetus
50
63
Gaviacetus
64
Protocetus
Phiomicetus
Rodhocetus
Dalanistes
53
Rayanistes
49
54
Kutchicetus
56
Andrewsiphius
55
Remingtonocetus
48
Ambulocetus
51
Pakicetidae
Hippopotamus
Sus_scrofa
50
45
40
35
30
25
20
15
10
5
0
state 0
state 1
state 2
state 3

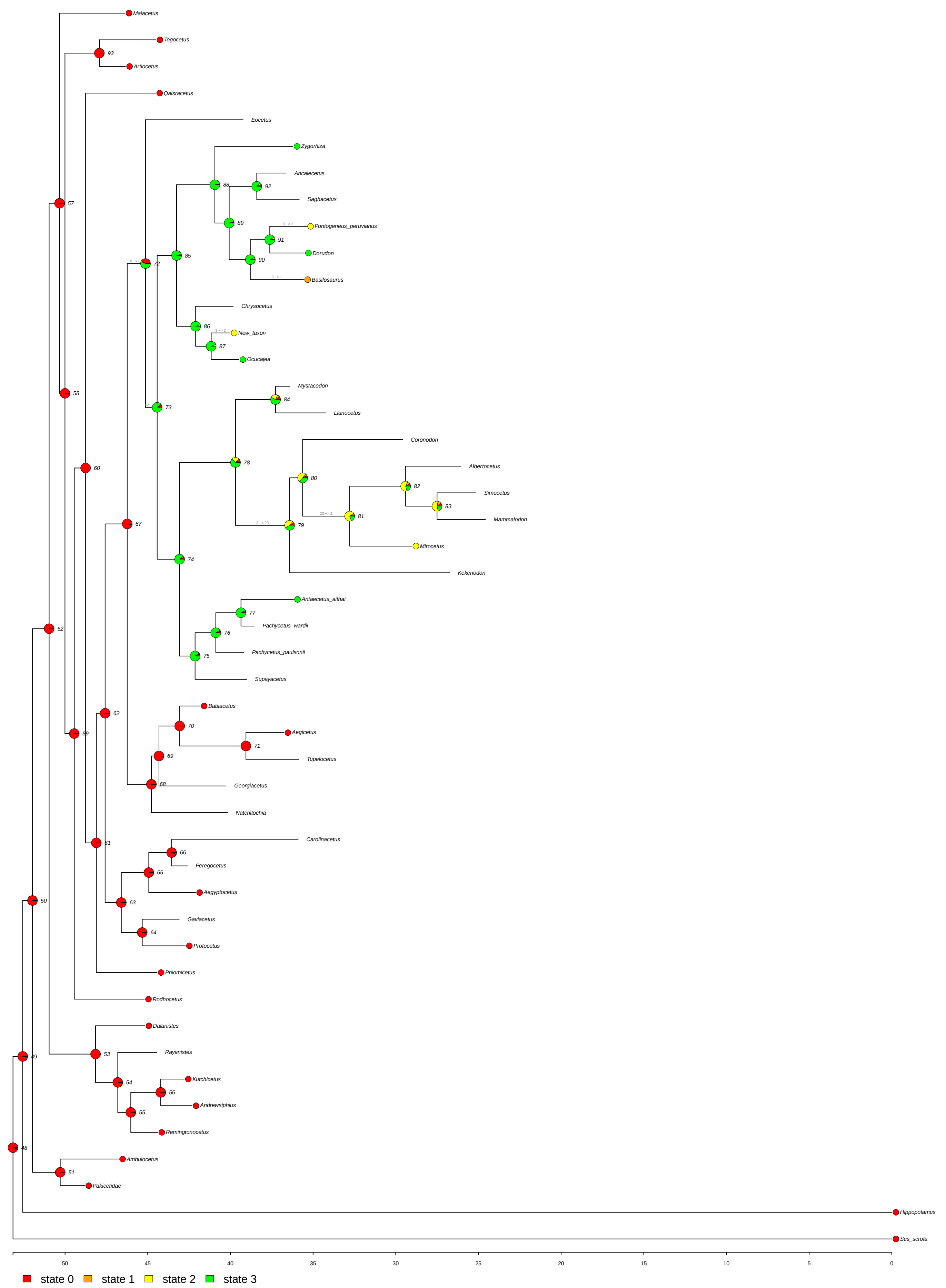

Maiacetus
Togocetus
Artiocetus
Qaisracetus
Eocetus
Zygorhiza
Ancalecetus
Saghacetus
Pontogeneus_peruvianus
Dorudon
Basilosaurus
Chrysocetus
New_taxon
Ocucajea
Mystacodon
Llanocetus
Coronodon
Albertocetus
Simocetus
Mammalodon
Mirocetus
Kekenodon
Antaecetus_aithai
Pachycetus_wardii
Pachycetus_paulsonii
Supayacetus
Babiacetus
Aegicetus
Tupelocetus
Georgiacetus
Natchitochia
Carolinacetus
Peregocetus
Aegyptocetus
Gaviacetus
Protocetus
Phiomicetus
Rodhocetus
Dalanistes
Rayanistes
Kutchicetus
Andrewsiphius
Remingtonocetus
Ambulocetus
Pakicetidae
Hippopotamus
Sus_scrofa
50
45
40
35
30
25
20
15
10
5
0
state 0
state 1
state 2
state 3

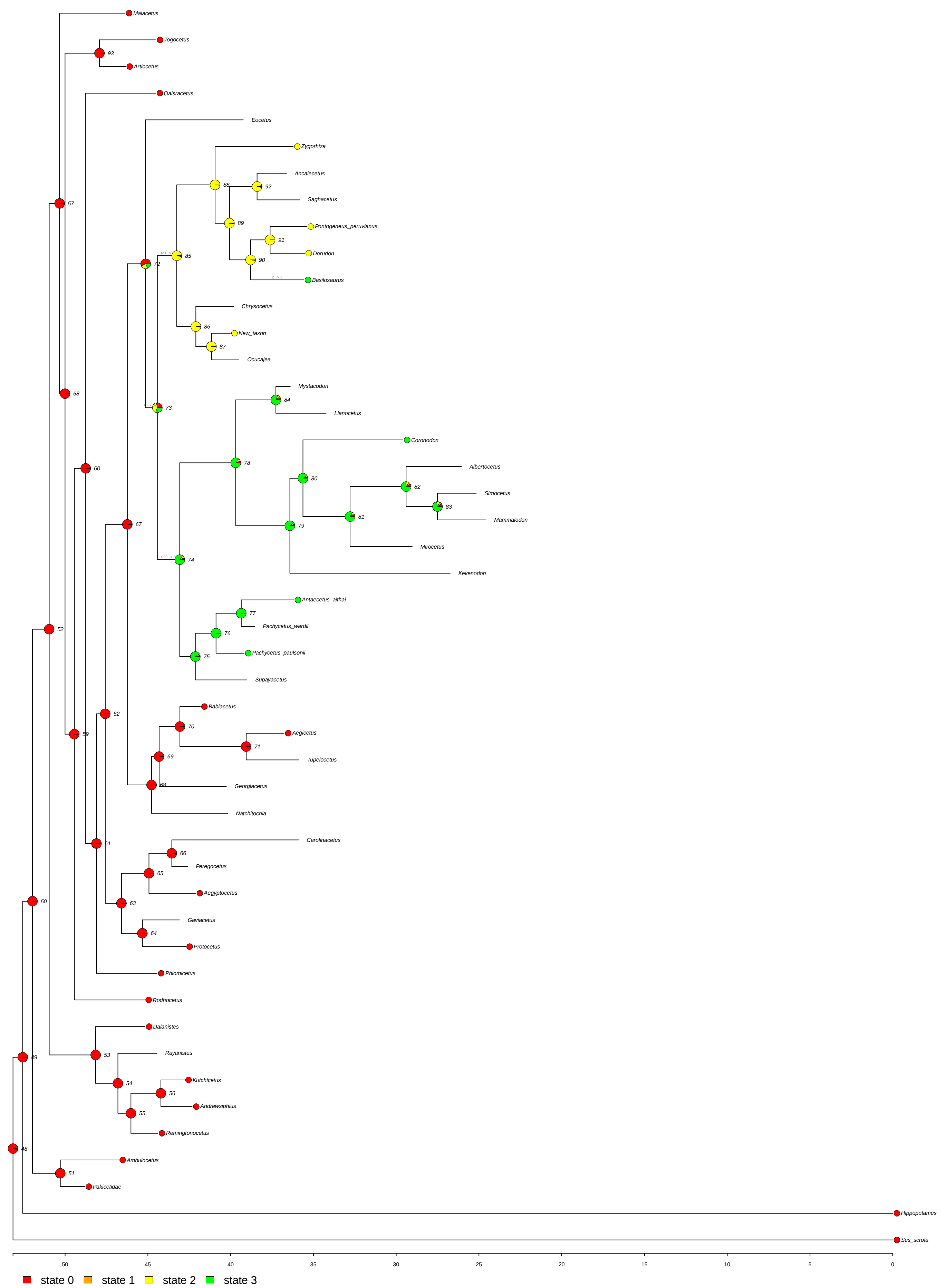
Maiacetus
Togocetus
93
Artiocetus
Qaisracetus
Eocetus
Zygorhiza
Ancalecetus
88
92
Saghacetus
89
Pontogeneus_peruvianus
91
Dorudon
90
72
85
Basilosaurus
Chrysocetus
86
New_taxon
87
Ocucajea
57
58
Mystacodon
84
Llanocetus
73
Coronodon
78
60
Albertocetus
80
82
Simocetus
83
Mammalodon
81
67
79
Mirocetus
74
Kekenodon
Antaecetus_aithai
77
Pachycetus_wardii
76
Pachycetus_paulsonii
75
52
Supayacetus
Babiacetus
62
70
Aegicetus
59
71
Tupelocetus
69
68
Georgiacetus
Natchitochia
Carolinacetus
61
66
Peregocetus
65
Aegyptocetus
50
63
Gaviacetus
64
Protocetus
Phiomicetus
Rodhocetus
Dalanistes
Rayanistes
49
53
54
Kutchicetus
56
Andrewsiphius
55
Remingtonocetus
48
Ambulocetus
51
Pakicetidae
Hippopotamus
Sus_scrofa
50
45
40
35
30
25
20
15
10
5
0
state 0
state 1
state 2
state 3

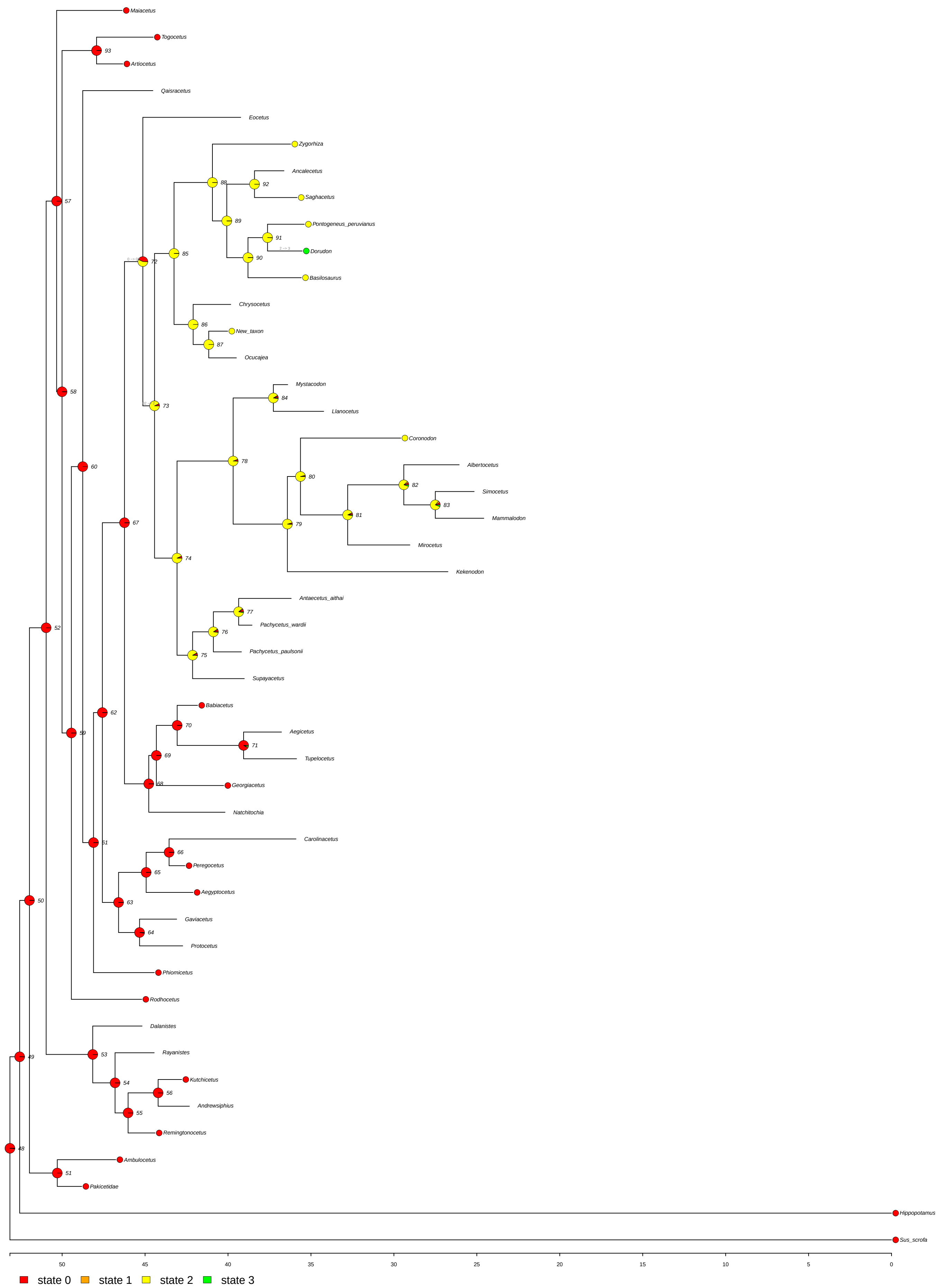

Maiacetus
Togocetus
Artiocetus
Qaisracetus
Eocetus
Zygorhiza
Ancalecetus
Saghacetus
Pontogeneus_peruvianus
Dorudon
Basilosaurus
Chrysocetus
New_taxon
Ocucajea
Mystacodon
Llanocetus
Coronodon
Albertocetus
Simocetus
Mammalodon
Mirocetus
Kekenodon
Antaecetus_aithai
Pachycetus_wardii
Pachycetus_paulsonii
Supayacetus
Babiacetus
Aegicetus
Tupelocetus
Georgiacetus
Natchitochia
Carolinacetus
Peregocetus
Aegyptocetus
Gaviacetus
Protocetus
Phiomicetus
Rodhocetus
Dalanistes
Rayanistes
Kutchicetus
Andrewsiphius
Remingtonocetus
Ambulocetus
Pakicetidae
Hippopotamus
Sus_scrofa
50 45 40 35 30 25 20 15 10 5 0
state 0
state 1
state 2
state 3

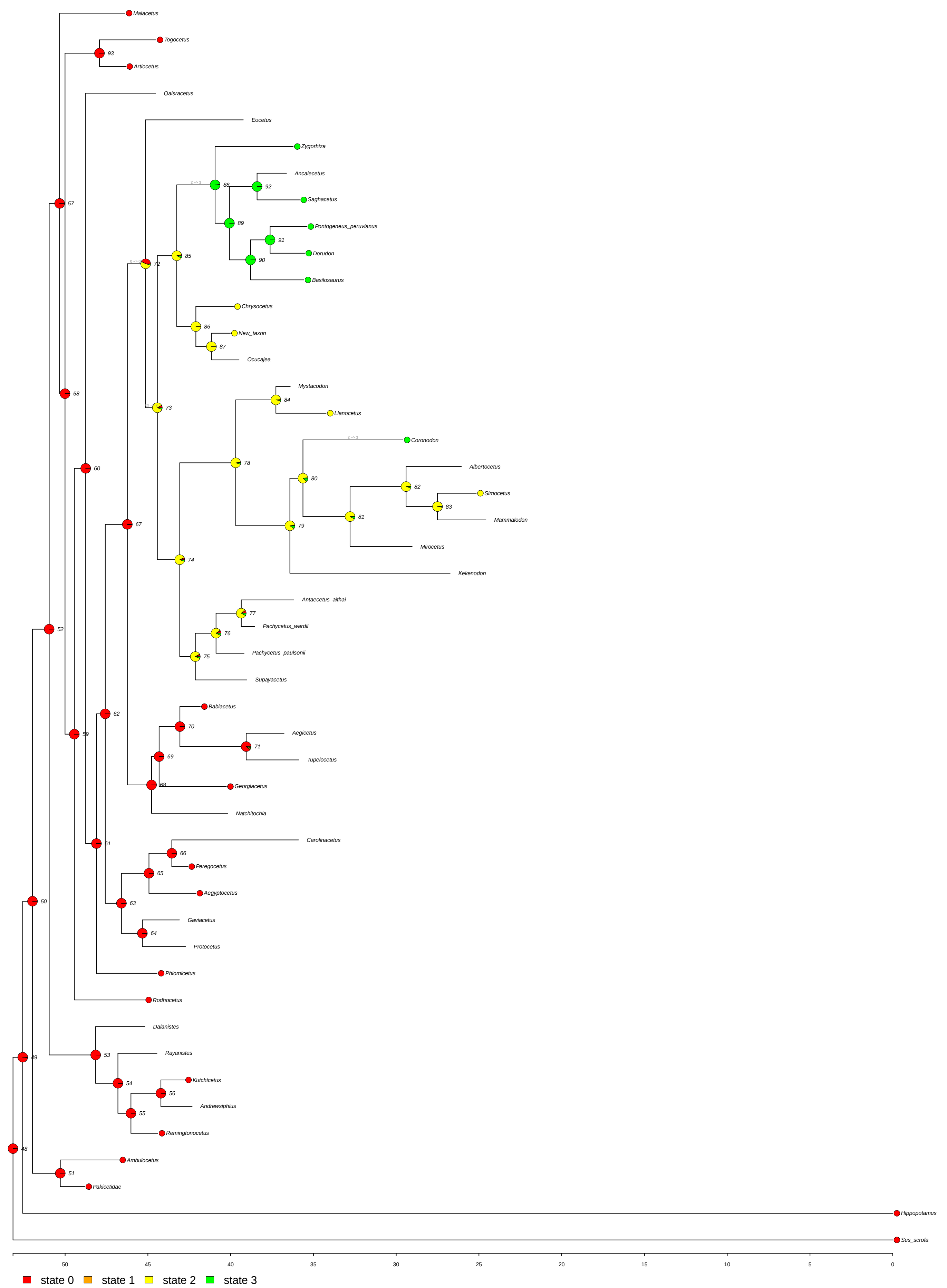

Maiacetus
Togocetus
Artiocetus
Qaisracetus
Eocetus
Zygorhiza
Ancalecetus
Saghacetus
Pontogeneus_peruvianus
Dorudon
Basilosaurus
Chrysocetus
New_taxon
Ocucajea
Mystacodon
Llanocetus
Coronodon
Albertocetus
Simocetus
Mammalodon
Mirocetus
Kekenodon
Antaecetus_aithai
Pachycetus_wardii
Pachycetus_paulsonii
Supayacetus
Babiacetus
Aegicetus
Tupelocetus
Georgiacetus
Natchitochia
Carolinacetus
Peregocetus
Aegyptocetus
Gaviacetus
Protocetus
Phiomicetus
Rodhocetus
Dalanistes
Rayanistes
Kutchicetus
Andrewsiphius
Remingtonocetus
Ambulocetus
Pakicetidae
Hippopotamus
Sus_scrofa
50
45
40
35
30
25
20
15
10
5
0
state 0
state 1
state 2
state 3

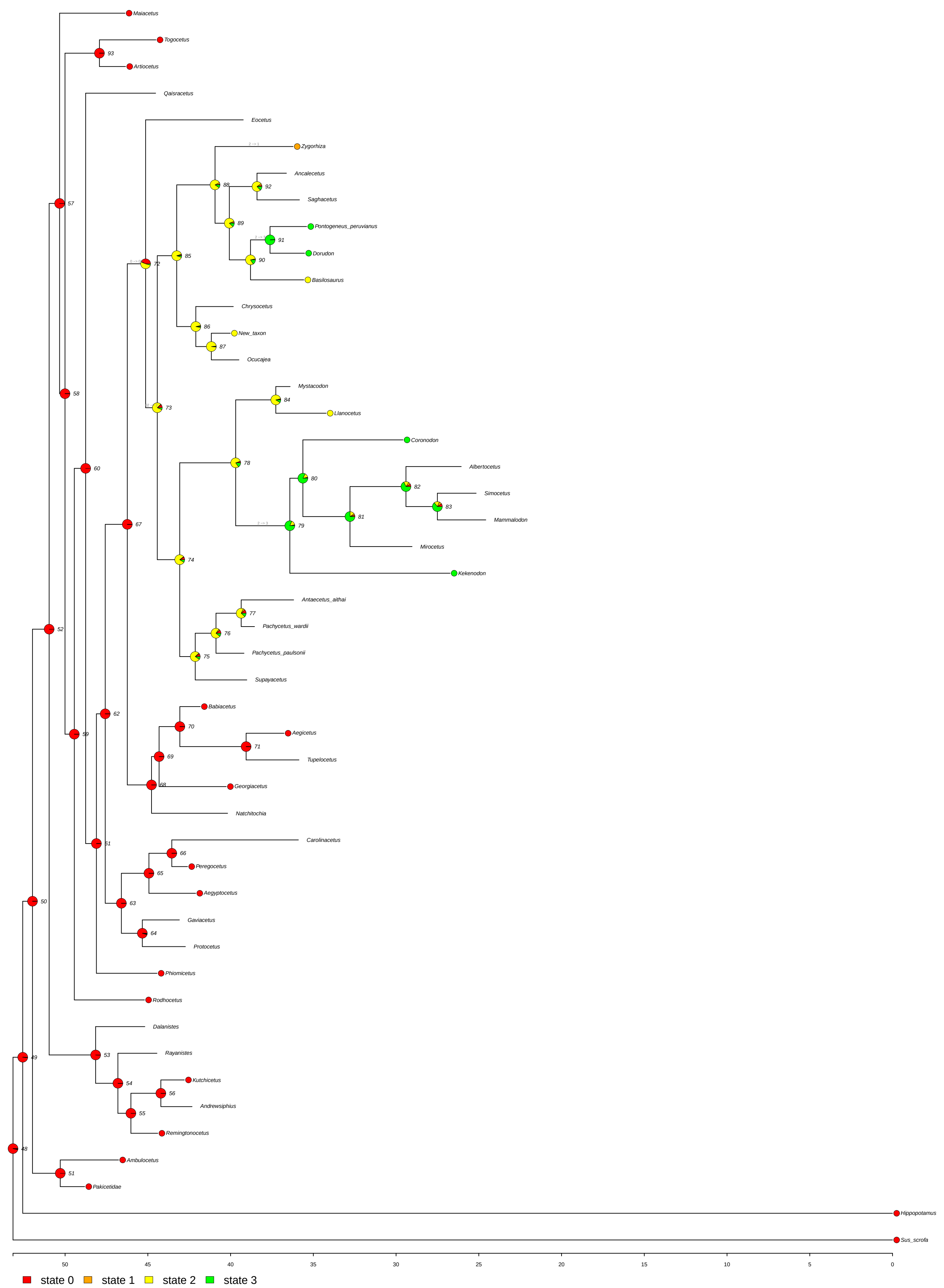

Maiacetus
Togocetus
93
Artiocetus
Qaisracetus
Eocetus
2 --> 1
Zygorhiza
Ancalecetus
88
92
Saghacetus
Pontogeneus_peruvianus
89
91
Dorudon
72
85
90
Basilosaurus
Chrysocetus
86
New_taxon
87
Ocucajea
57
Mystacodon
84
58
Llanocetus
73
Coronodon
60
78
Albertocetus
80
82
Simocetus
83
67
2 --> 3
81
Mammalodon
79
Mirocetus
74
Kekenodon
52
Antaecetus_aithai
77
Pachycetus_wardii
76
Pachycetus_paulsonii
75
Supayacetus
Babiacetus
62
70
59
Aegicetus
71
Tupelocetus
69
68
Georgiacetus
Natchitochia
Carolinacetus
61
66
Peregocetus
65
Aegyptocetus
63
Gaviacetus
64
Protocetus
Phiomicetus
Rodhocetus
Dalanistes
49
53
Rayanistes
54
Kutchicetus
56
Andrewsiphius
55
Remingtonocetus
48
Ambulocetus
51
Pakicetidae
Hippopotamus
Sus_scrofa
50
45
40
35
30
25
20
15
10
5
0
state 0
state 1
state 2
state 3

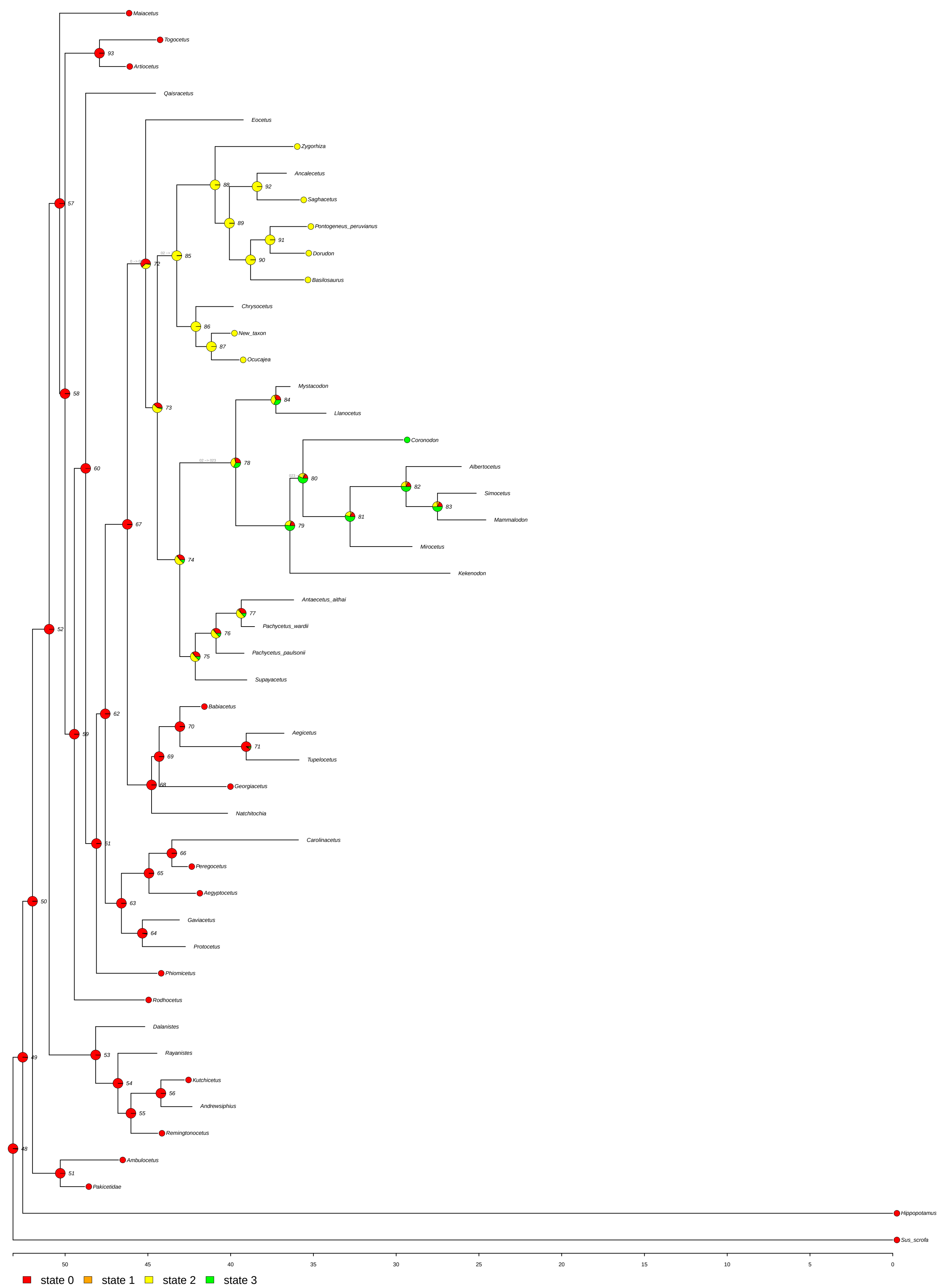

Maiacetus
Togocetus
93
Artiocetus
Qaisracetus
Eocetus
Zygorhiza
Ancalecetus
88
92
Saghacetus
89
Pontogeneus_peruvianus
91
Dorudon
90
85
72
Basilosaurus
Chrysocetus
86
New_taxon
87
Ocucajea
Mystacodon
84
Llanocetus
73
Coronodon
78
02 --> 023
80
Albertocetus
82
Simocetus
83
81
Mammalodon
67
79
Mirocetus
74
Kekenodon
Antaecetus_aithai
77
Pachycetus_wardii
76
Pachycetus_paulsonii
75
Supayacetus
Babiacetus
62
70
59
Aegicetus
71
Tupelocetus
69
68
Georgiacetus
Natchitochia
Carolinacetus
61
66
Peregocetus
65
Aegyptocetus
63
Gaviacetus
64
Protocetus
Phiomicetus
Rodhocetus
Dalanistes
Rayanistes
53
Kutchicetus
54
56
Andrewsiphius
55
Remingtonocetus
48
Ambulocetus
51
Pakicetidae
Hippopotamus
Sus_scrofa
57
58
60
52
50
49
50
45
40
35
30
25
20
15
10
5
0
state 0
state 1
state 2
state 3

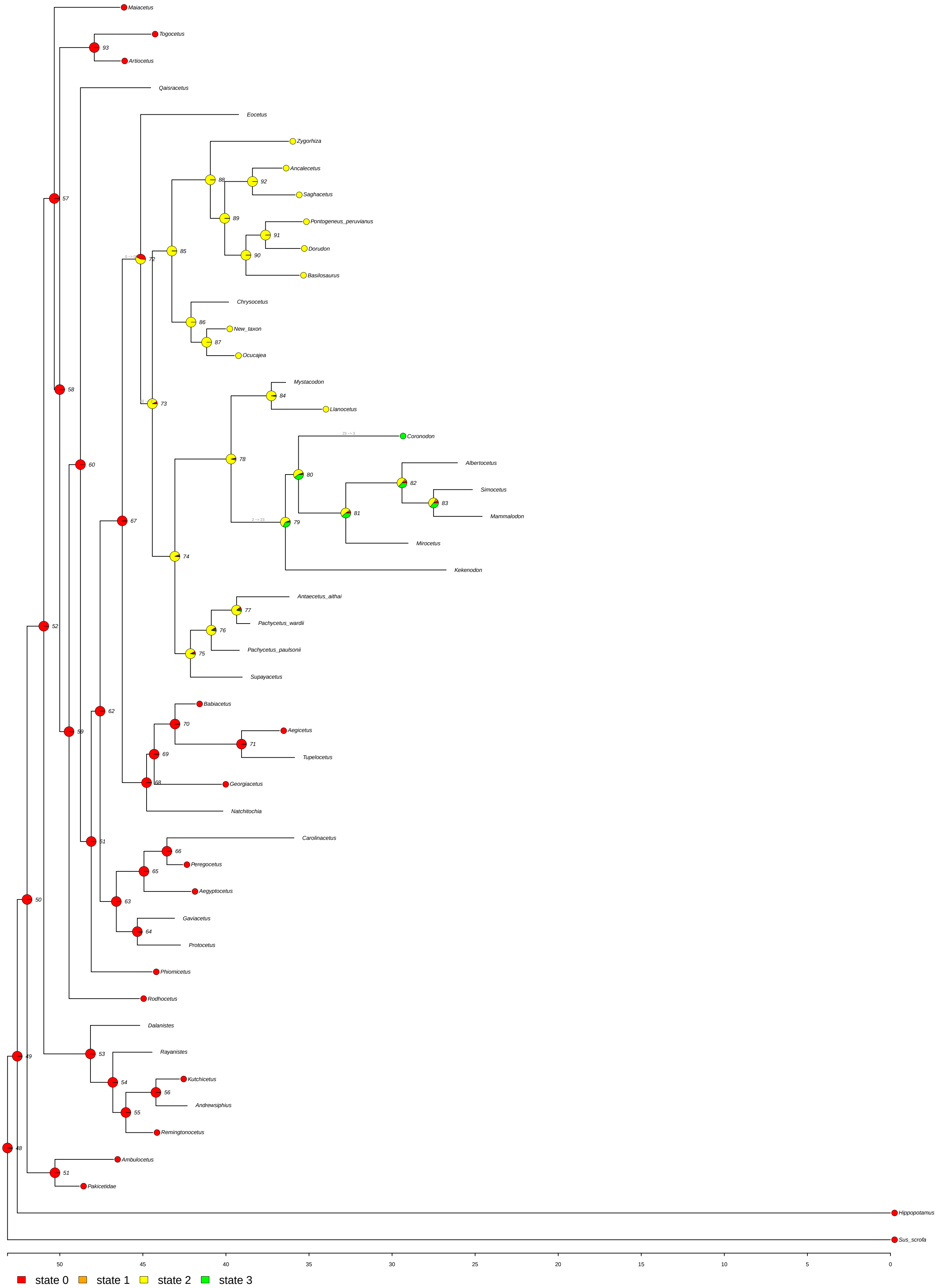

Maiacetus
Togocetus
93
Artiocetus
Qaisracetus
Eocetus
Zygorhiza
Ancalecetus
88
92
Saghacetus
89
Pontogeneus_peruvianus
91
Dorudon
90
Basilosaurus
72
85
57
Chrysocetus
86
New_taxon
87
Ocucajea
Mystacodon
84
Llanocetus
58
73
Coronodon
78
Albertocetus
80
82
Simocetus
83
Mammalodon
81
79
Mirocetus
67
74
Kekenodon
Antaecetus_aithai
77
Pachycetus_wardii
76
52
Pachycetus_paulsonii
75
Supayacetus
Babiacetus
62
70
59
Aegicetus
71
Tupelocetus
69
68
Georgiacetus
Natchitochia
Carolinacetus
61
66
Peregocetus
65
50
Aegyptocetus
63
Gaviacetus
64
Protocetus
Phiomicetus
Rodhocetus
Dalanistes
53
Rayanistes
49
54
Kutchicetus
56
55
Andrewsiphius
Remingtonocetus
48
Ambulocetus
51
Pakicetidae
Hippopotamus
Sus_scrofa
50
45
40
35
30
25
20
15
10
5
0
state 0
state 1
state 2
state 3

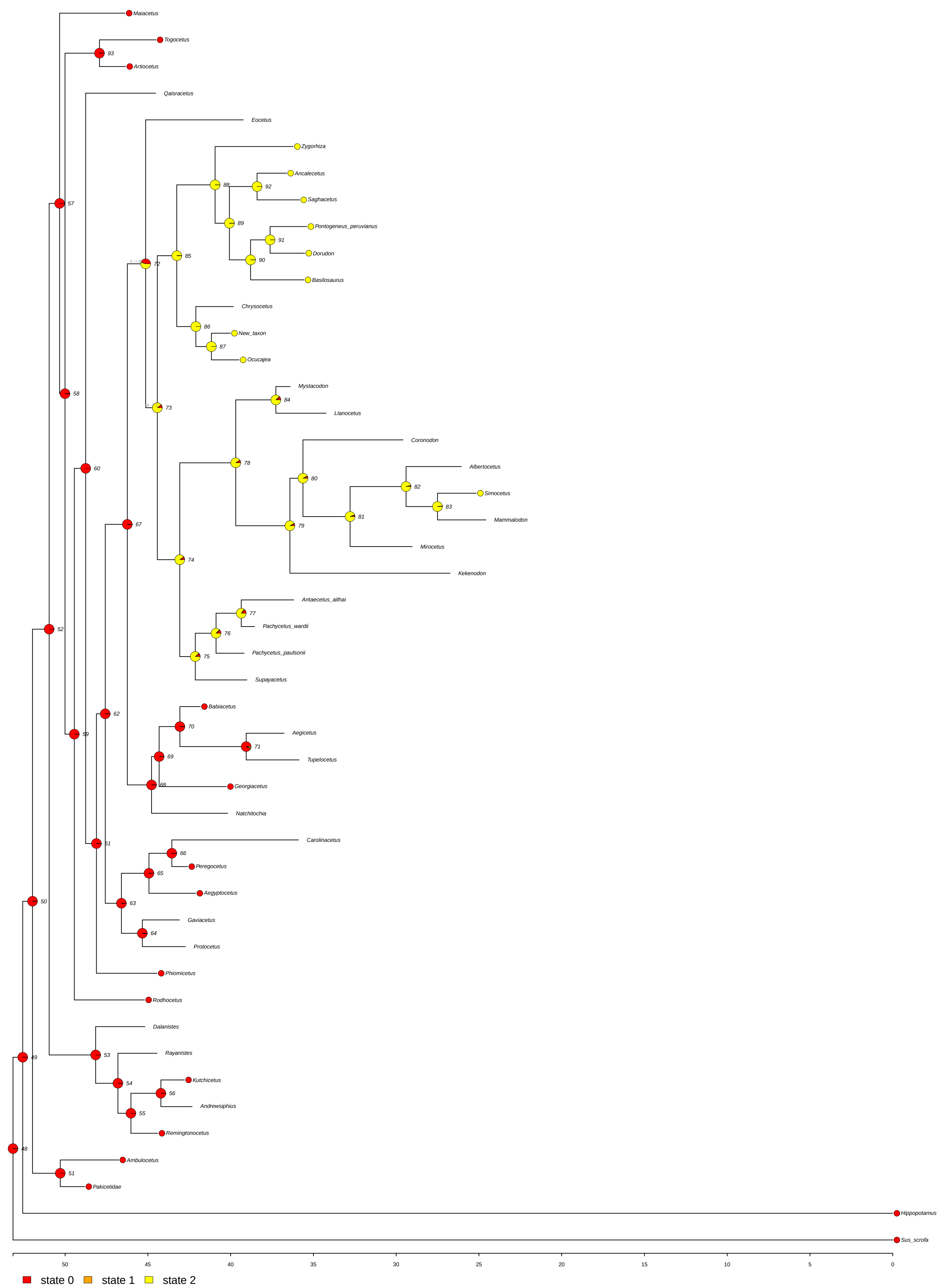

Maiacetus
Togocetus
93
Artiocetus
Qaisracetus
Eocetus
Zygorhiza
88
Ancalecetus
92
Saghacetus
89
Pontogeneus_peruvianus
91
Dorudon
90
Basilosaurus
85
72
57
Chrysocetus
86
New_taxon
87
Ocucajea
Mystacodon
84
Llanocetus
58
73
Coronodon
78
Albertocetus
80
82
Simocetus
83
Mammalodon
81
79
60
67
Mirocetus
Kekenodon
74
Antaecetus_aithai
77
Pachycetus_wardii
76
Pachycetus_paulsonii
75
52
Supayacetus
Babiacetus
62
70
Aegicetus
59
71
Tupelocetus
69
68
Georgiacetus
Natchitochia
Carolinacetus
61
66
Peregocetus
65
Aegyptocetus
50
63
Gaviacetus
64
Protocetus
Phiomicetus
Rodhocetus
Dalanistes
53
Rayanistes
49
54
Kutchicetus
56
Andrewsiphius
55
Remingtonocetus
48
Ambulocetus
51
Pakicetidae
Hippopotamus
Sus_scrofa
50
45
40
35
30
25
20
15
10
5
0
state 0
state 1
state 2

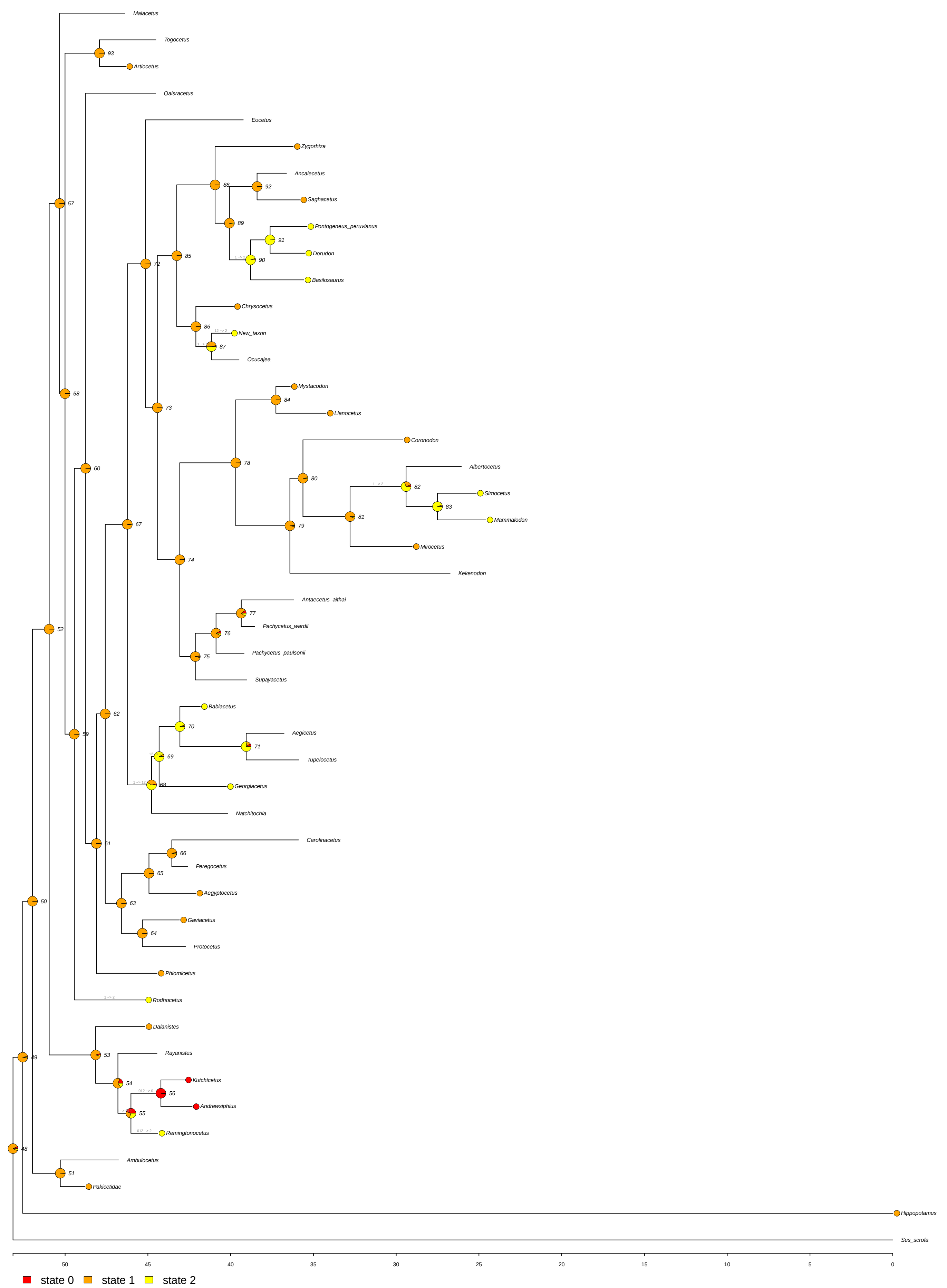

Maiacetus
Togocetus
93
Artiocetus
Qaisracetus
Eocetus
Zygorhiza
Ancalecetus
88
92
Saghacetus
89
Pontogeneus_peruvianus
91
Dorudon
1 -> 2
90
Basilosaurus
57
72
85
Chrysocetus
86
12 -> 2
New_taxon
87
Ocucajea
Mystacodon
84
58
73
Llanocetus
Coronodon
60
78
Albertocetus
80
1 -> 2
82
Simocetus
83
67
81
Mammalodon
79
Mirocetus
74
Kekenodon
Antaecetus_aithai
77
Pachycetus_wardii
52
76
Pachycetus_paulsonii
75
Supayacetus
Babiacetus
62
70
59
Aegicetus
71
Tupelocetus
69
1 -> 12
68
Georgiacetus
Natchitochia
Carolinacetus
61
66
Peregocetus
65
50
Aegyptocetus
63
Gaviacetus
64
Protocetus
Phiomicetus
1 -> 2
Rodhocetus
Dalanistes
Rayanistes
49
53
Kutchicetus
54
012 -> 0
56
Andrewsiphius
55
012 -> 2
Remingtonocetus
48
Ambulocetus
51
Pakicetidae
Hippopotamus
Sus_scrofa
50
45
40
35
30
25
20
15
10
5
0
state 0
state 1
state 2

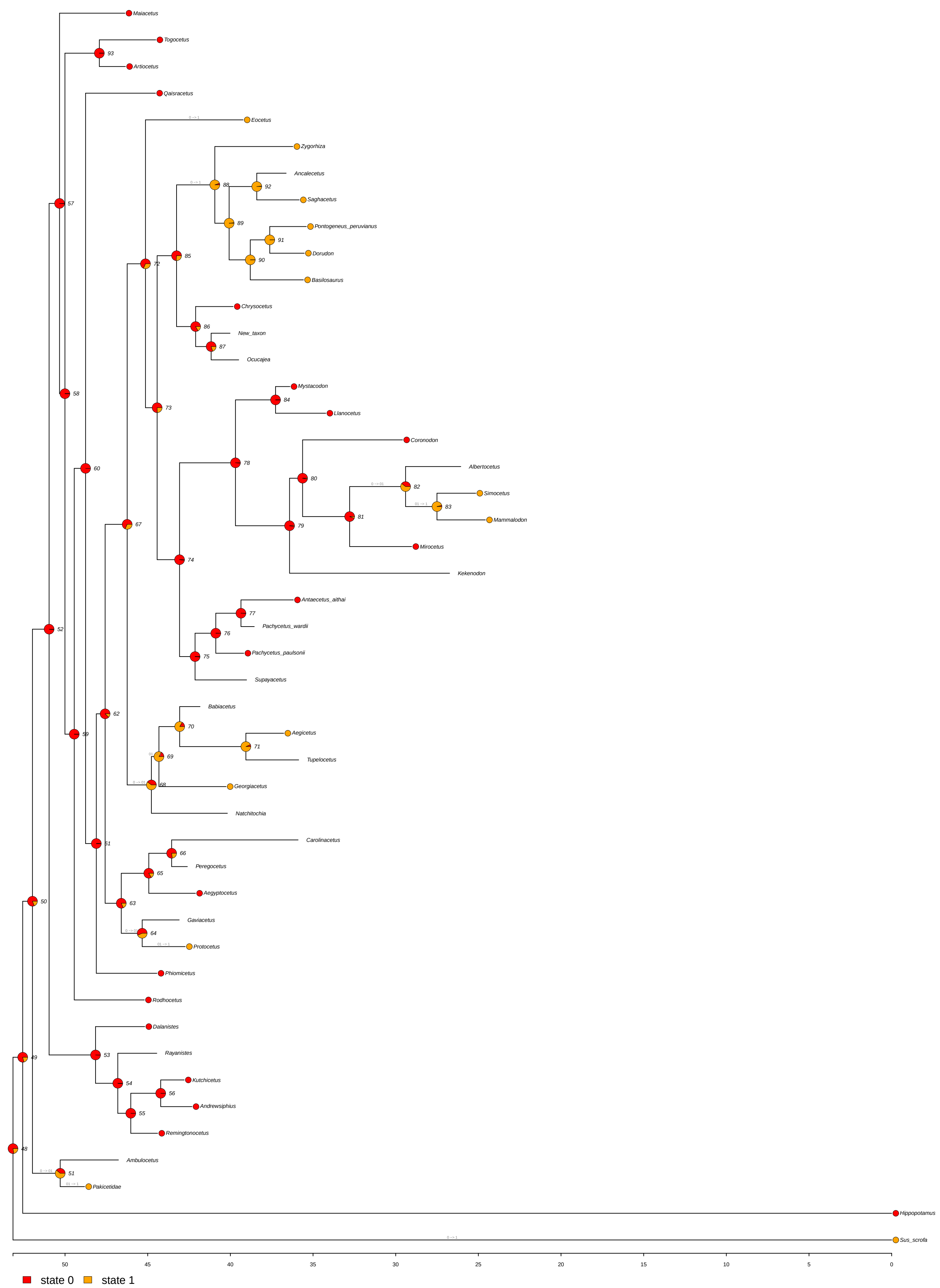
Maiacetus
Togocetus
Artiocetus
Qaisracetus
Eocetus
Zygorhiza
Ancalecetus
Saghacetus
Pontogeneus_peruvianus
Dorudon
Basilosaurus
Chrysocetus
New_taxon
Ocucajea
Mystacodon
Llanocetus
Coronodon
Albertocetus
Simocetus
Mammalodon
Mirocetus
Kekenodon
Antaecetus_aithai
Pachycetus_wardii
Pachycetus_paulsonii
Supayacetus
Babiacetus
Aegicetus
Tupelocetus
Georgiacetus
Natchitochia
Carolinacetus
Peregocetus
Aegyptocetus
Gaviacetus
Protocetus
Phiomicetus
Rodhocetus
Dalanistes
Rayanistes
Kutchicetus
Andrewsiphius
Remingtonocetus
Ambulocetus
Pakicetidae
Hippopotamus
Sus_scrofa
50
45
40
35
30
25
20
15
10
5
0
state 0
state 1

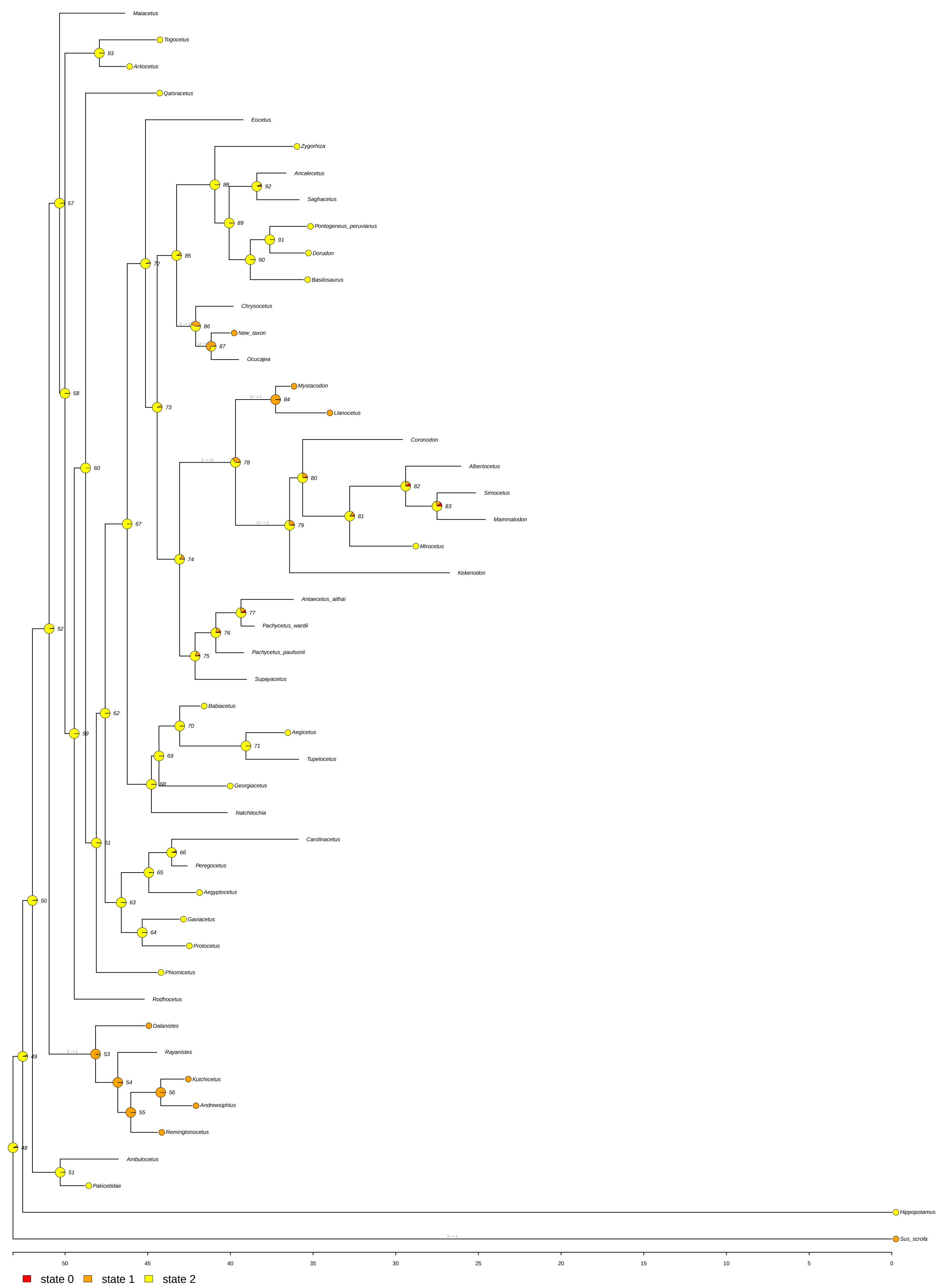

Maiacetus
Togocetus
93
Artiocetus
Qaisracetus
Eocetus
Zygorhiza
Ancalecetus
88
92
Saghacetus
Pontogeneus_peruvianus
89
91
Dorudon
90
Basilosaurus
57
72
85
Chrysocetus
86
New_taxon
87
Ocucajea
Mystacodon
84
Llanocetus
58
73
Coronodon
78
Albertocetus
80
82
Simocetus
83
Mammalodon
60
81
67
79
Mirocetus
74
Kekenodon
Antaecetus_aithai
77
Pachycetus_wardii
52
76
Pachycetus_paulsonii
75
Supayacetus
Babiacetus
62
70
Aegicetus
59
71
Tupelocetus
69
68
Georgiacetus
Natchitochia
Carolinacetus
61
66
Peregocetus
65
Aegyptocetus
50
63
Gaviacetus
64
Protocetus
Phiomicetus
Rodhocetus
Dalanistes
49
53
Rayanistes
54
Kutchicetus
56
Andrewsiphius
55
Remingtonocetus
48
Ambulocetus
51
Pakicetidae
Hippopotamus
Sus_scrofa
50
45
40
35
30
25
20
15
10
5
0
state 0
state 1
state 2

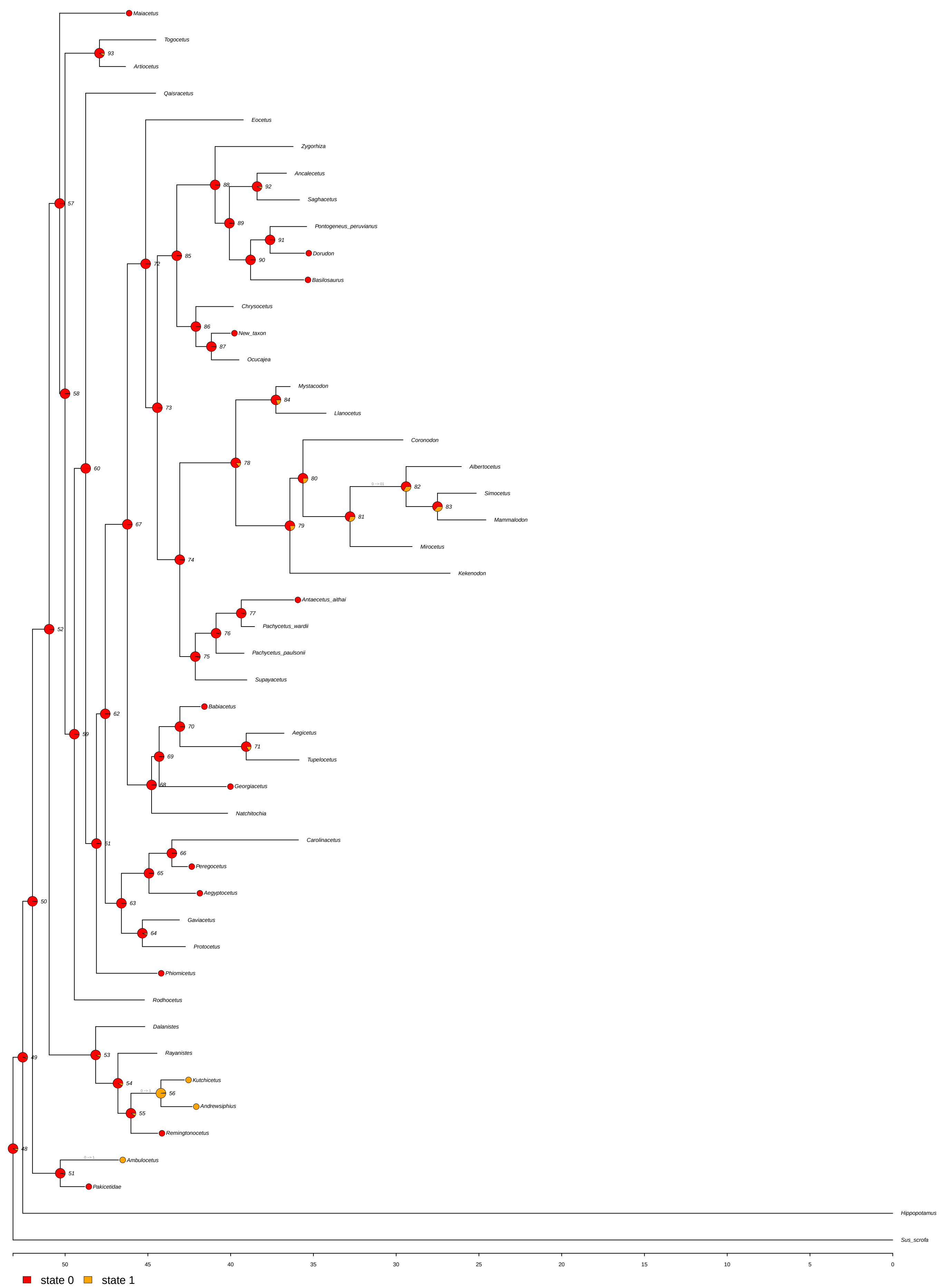

Maiacetus
Togocetus
Artiocetus
Qaisracetus
Eocetus
Zygorhiza
Ancalecetus
Saghacetus
Pontogeneus_peruvianus
Dorudon
Basilosaurus
Chrysocetus
New_taxon
Ocucajea
Mystacodon
Llanocetus
Coronodon
Albertocetus
Simocetus
Mammalodon
Mirocetus
Kekenodon
Antaecetus_aithai
Pachycetus_wardii
Pachycetus_paulsonii
Supayacetus
Babiacetus
Aegicetus
Tupelocetus
Georgiacetus
Natchitochia
Carolinacetus
Peregocetus
Aegyptocetus
Gaviacetus
Protocetus
Phiomicetus
Rodhocetus
Dalanistes
Rayanistes
Kutchicetus
Andrewsiphius
Remingtonocetus
Ambulocetus
Pakicetidae
Hippopotamus
Sus_scrofa
48
49
50
51
52
53
54
55
56
57
58
59
60
61
62
63
64
65
66
67
68
69
70
71
72
73
74
75
76
77
78
79
80
81
82
83
84
85
86
87
88
89
90
91
92
93
50
45
40
35
30
25
20
15
10
5
0
state 0
state 1

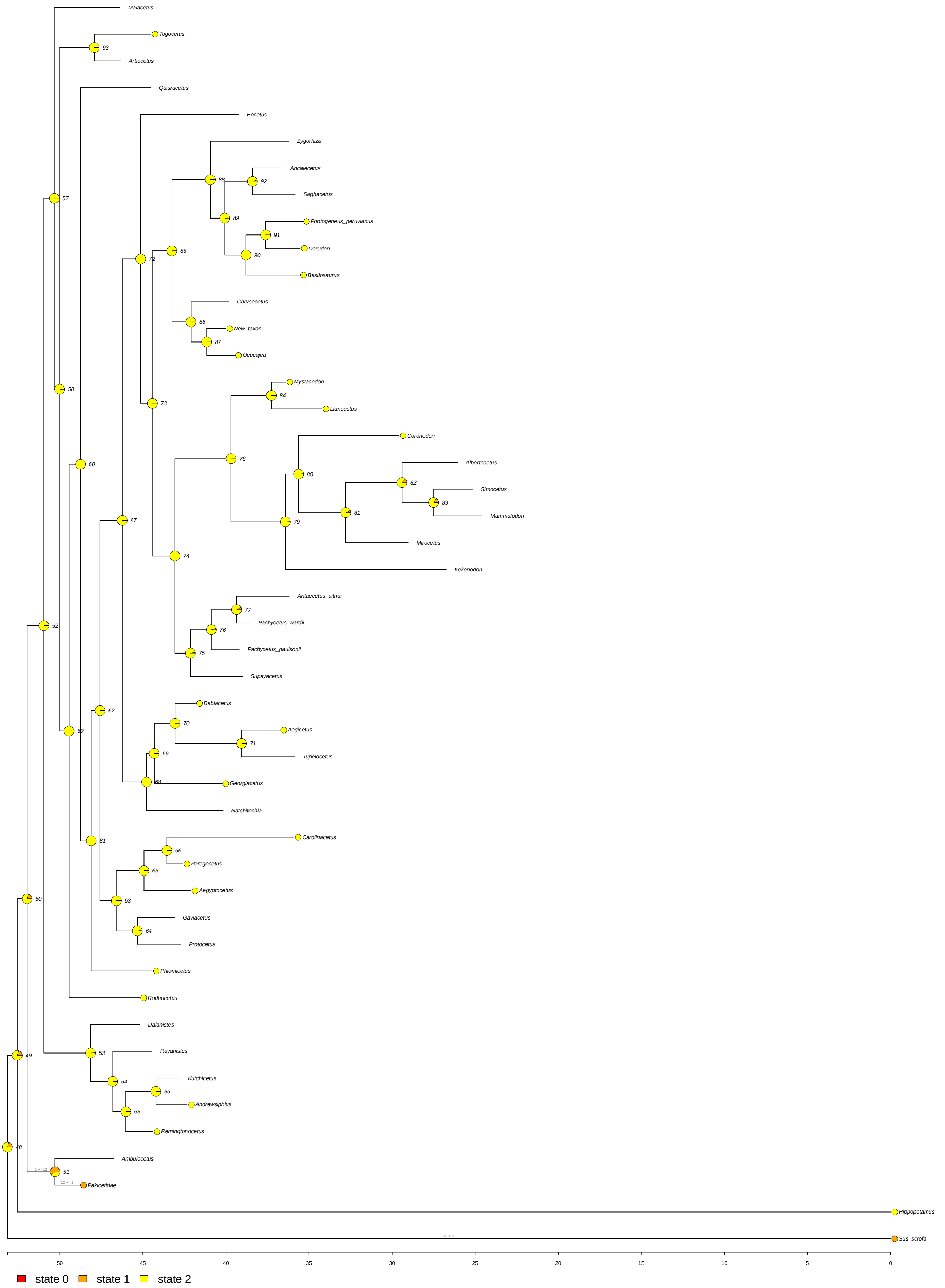

Maiacetus
Togocetus
Artiocetus
Qaisracetus
Eocetus
Zygorhiza
Ancalecetus
Saghacetus
Pontogeneus_peruvianus
Dorudon
Basilosaurus
Chrysocetus
New_taxon
Ocucajea
Mystacodon
Llanocetus
Coronodon
Albertocetus
Simocetus
Mammalodon
Mirocetus
Kekenodon
Antaecetus_aithai
Pachycetus_wardii
Pachycetus_paulsonii
Supayacetus
Babiacetus
Aegicetus
Tupelocetus
Georgiacetus
Natchitochia
Carolinacetus
Peregocetus
Aegyptocetus
Gaviacetus
Protocetus
Phiomicetus
Rodhocetus
Dalanistes
Rayanistes
Kutchicetus
Andrewsiphius
Remingtonocetus
Ambulocetus
Pakicetidae
Hippopotamus
Sus_scrofa
50
45
40
35
30
25
20
15
10
5
0
state 0
state 1
state 2

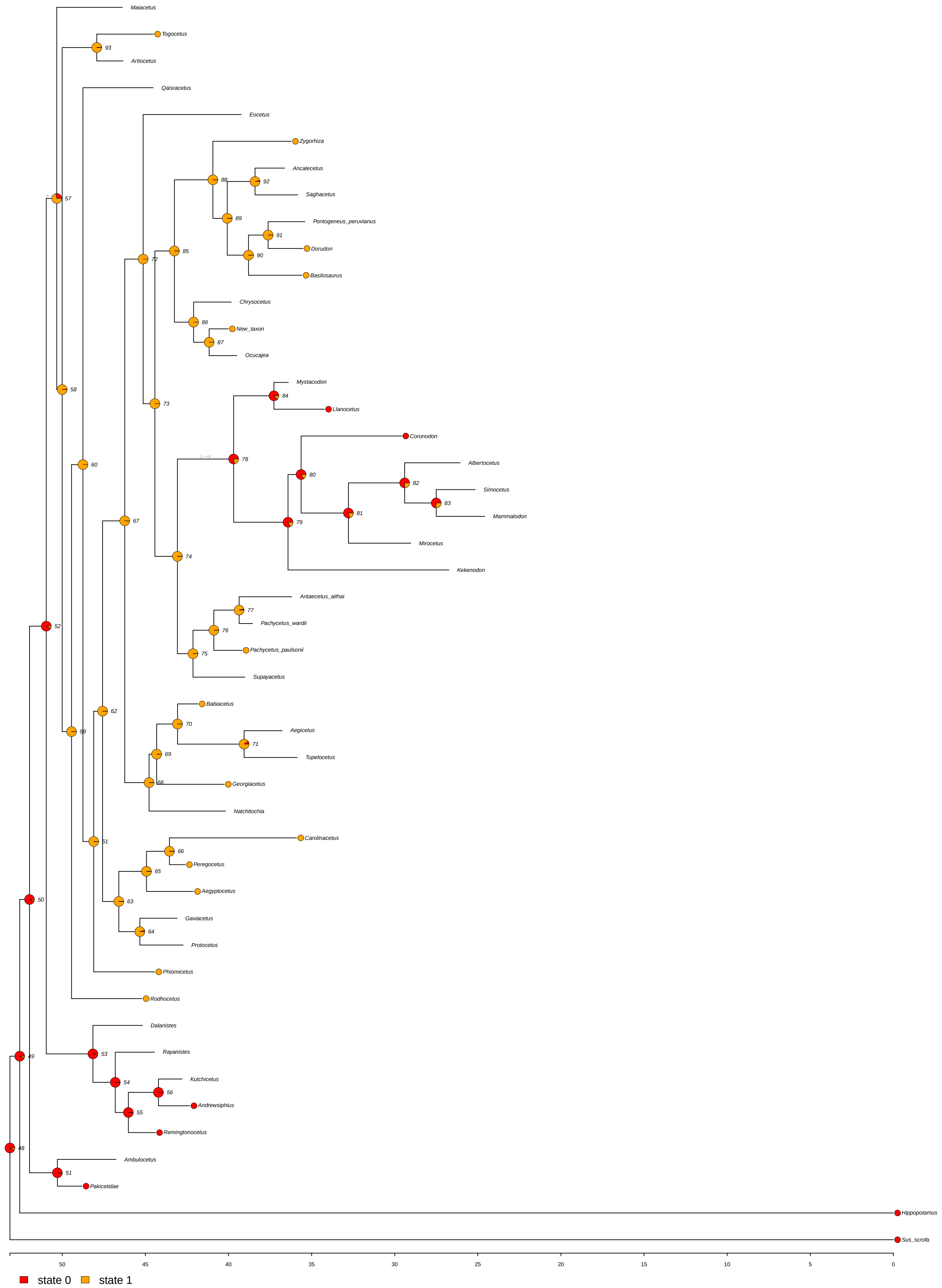

Maiacetus
Togocetus
93
Artiocetus
Qaisracetus
Eocetus
Zygorhiza
Ancalecetus
88
92
Saghacetus
Pontogeneus_peruvianus
89
91
Dorudon
90
Basilosaurus
57
72
85
Chrysocetus
86
New_taxon
87
Ocucajea
Mystacodon
84
Llanocetus
58
73
Coronodon
1 --> 0
78
80
Albertocetus
82
Simocetus
83
Mammalodon
81
60
67
79
Mirocetus
74
Kekenodon
Antaecetus_aithai
77
Pachycetus_wardii
76
52
Pachycetus_paulsonii
75
Supayacetus
Babiacetus
62
70
Aegicetus
59
71
Tupelocetus
69
68
Georgiacetus
Natchitochia
Carolinacetus
61
66
Peregocetus
65
Aegyptocetus
50
63
Gaviacetus
64
Protocetus
Phiomicetus
Rodhocetus
Dalanistes
49
53
Rayanistes
Kutchicetus
54
56
Andrewsiphius
55
Remingtonocetus
48
Ambulocetus
51
Pakicetidae
Hippopotamus
Sus_scrofa
50
45
40
35
30
25
20
15
10
5
0
state 0
state 1

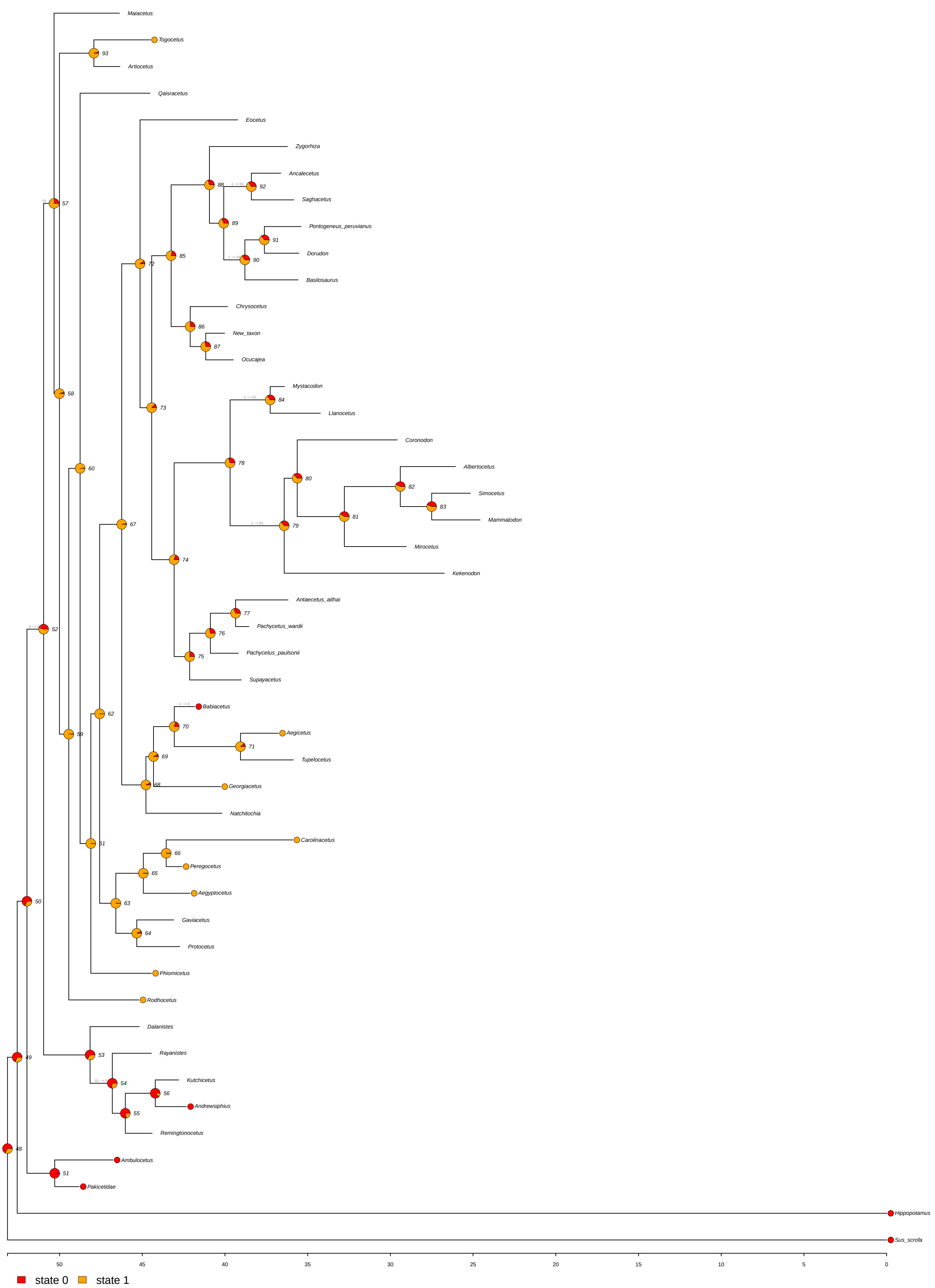
Maiacetus
Togocetus
93
Artiocetus
Qaisracetus
Eocetus
Zygorhiza
Ancalecetus
88
92
Saghacetus
89
Pontogeneus_peruvianus
91
57
85
72
Dorudon
90
Basilosaurus
Chrysocetus
86
New_taxon
87
Ocucajea
Mystacodon
84
58
73
Llanocetus
Coronodon
60
78
80
Albertocetus
82
Simocetus
83
67
79
81
Mammalodon
Mirocetus
74
Kekenodon
Antaecetus_aithai
77
Pachycetus_wardii
52
76
Pachycetus_paulsonii
75
Supayacetus
Babiacetus
62
70
Aegicetus
59
71
Tupelocetus
69
68
Georgiacetus
Natchitochia
61
Carolinacetus
66
Peregocetus
65
50
63
Aegyptocetus
Gaviacetus
64
Protocetus
Phiomicetus
Rodhocetus
Dalanistes
49
53
Rayanistes
54
Kutchicetus
56
55
Andrewsiphius
Remingtonocetus
48
Ambulocetus
51
Pakicetidae
Hippopotamus
Sus_scrofa
50
45
40
35
30
25
20
15
10
5
0
state 0
state 1

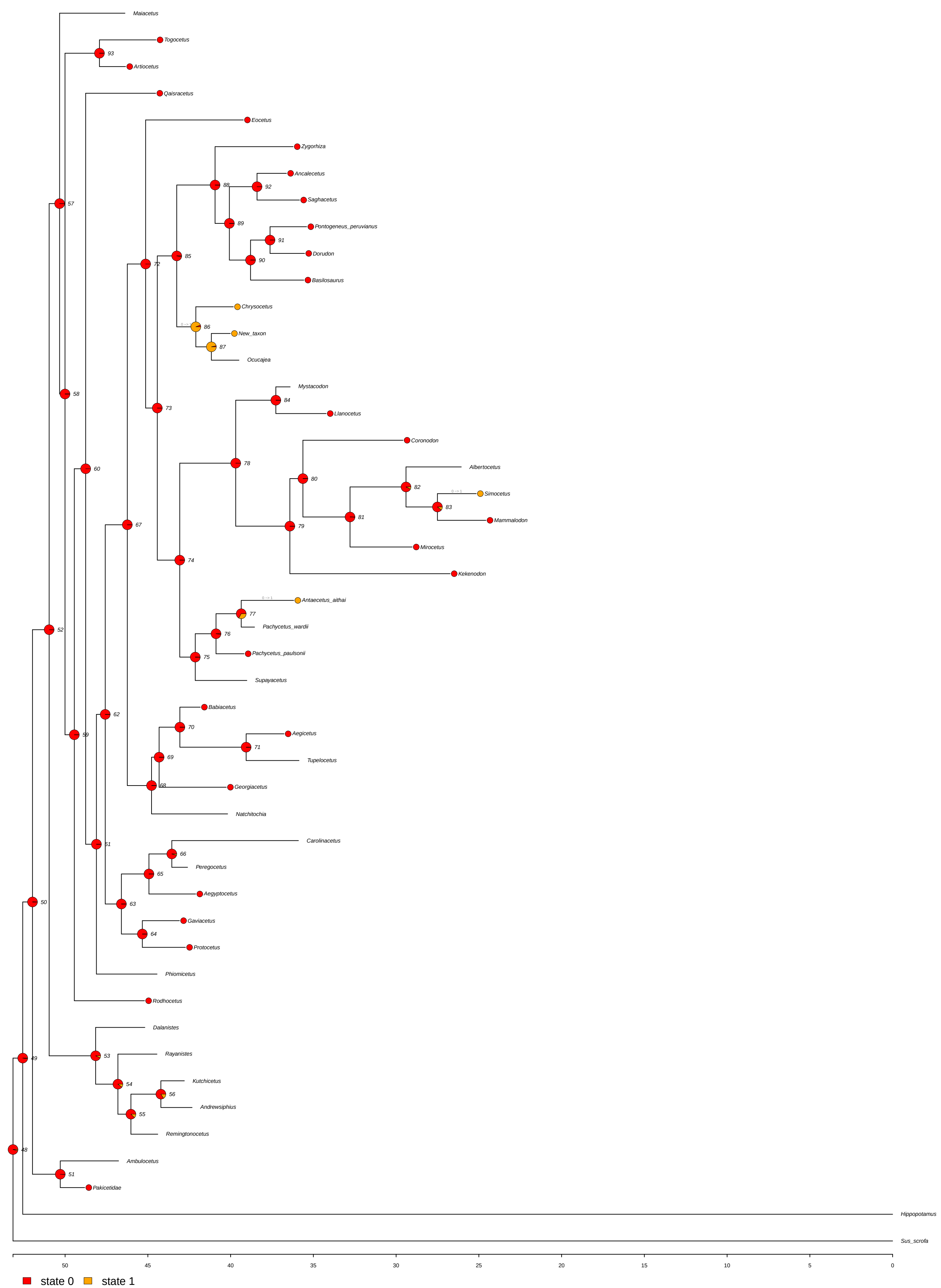

Maiacetus
Togocetus
93
Artiocetus
Qaisracetus
Eocetus
Zygorhiza
Ancalecetus
88
92
Saghacetus
Pontogeneus_peruvianus
89
91
Dorudon
90
Basilosaurus
57
72
85
Chrysocetus
86
New_taxon
87
Ocucajea
Mystacodon
84
Llanocetus
58
73
Coronodon
60
78
Albertocetus
80
82
Simocetus
83
67
81
Mammalodon
79
Mirocetus
74
Kekenodon
Antaecetus_aithai
77
Pachycetus_wardii
52
76
Pachycetus_paulsonii
75
Supayacetus
Babiacetus
62
70
59
Aegicetus
71
Tupelocetus
69
68
Georgiacetus
Natchitochia
Carolinacetus
61
66
Peregocetus
65
Aegyptocetus
50
63
Gaviacetus
64
Protocetus
Phiomicetus
Rodhocetus
Dalanistes
53
Rayanistes
49
Kutchicetus
54
56
Andrewsiphius
55
Remingtonocetus
48
Ambulocetus
51
Pakicetidae
Hippopotamus
Sus_scrofa
50
45
40
35
30
25
20
15
10
5
0
state 0
state 1

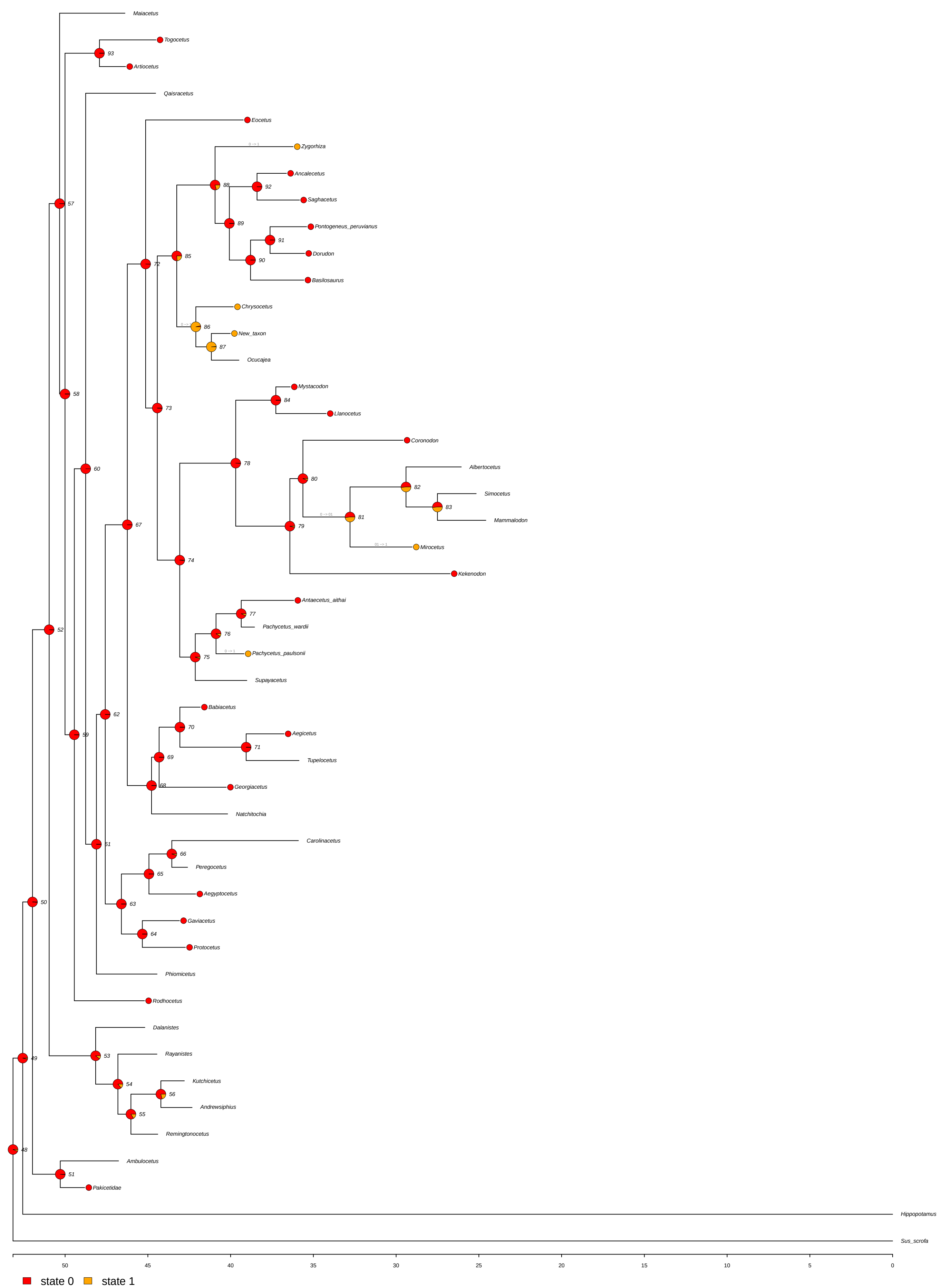
Maiacetus
Togocetus
93
Artiocetus
Qaisracetus
Eocetus
0 -> 1
Zygorhiza
Ancalecetus
88
92
Saghacetus
89
Pontogeneus_peruvianus
91
Dorudon
90
Basilosaurus
57
72
85
Chrysocetus
86
New_taxon
87
Ocucajea
Mystacodon
84
Llanocetus
58
73
Coronodon
78
Albertocetus
60
80
82
Simocetus
83
0 -> 01
81
Mammalodon
67
79
01 -> 1
Mirocetus
74
Kekenodon
Antaecetus_aithai
77
Pachycetus_wardii
76
52
0 -> 1
Pachycetus_paulsonii
75
Supayacetus
Babiacetus
62
70
Aegicetus
59
71
Tupelocetus
69
68
Georgiacetus
Natchitochia
Carolinacetus
61
66
Peregocetus
65
Aegyptocetus
50
63
Gaviacetus
64
Protocetus
Phiomicetus
Rodhocetus
Dalanistes
Rayanistes
53
49
54
Kutchicetus
56
Andrewsiphius
55
Remingtonocetus
48
Ambulocetus
51
Pakicetidae
Hippopotamus
Sus_scrofa
50
45
40
35
30
25
20
15
10
5
0
state 0
state 1

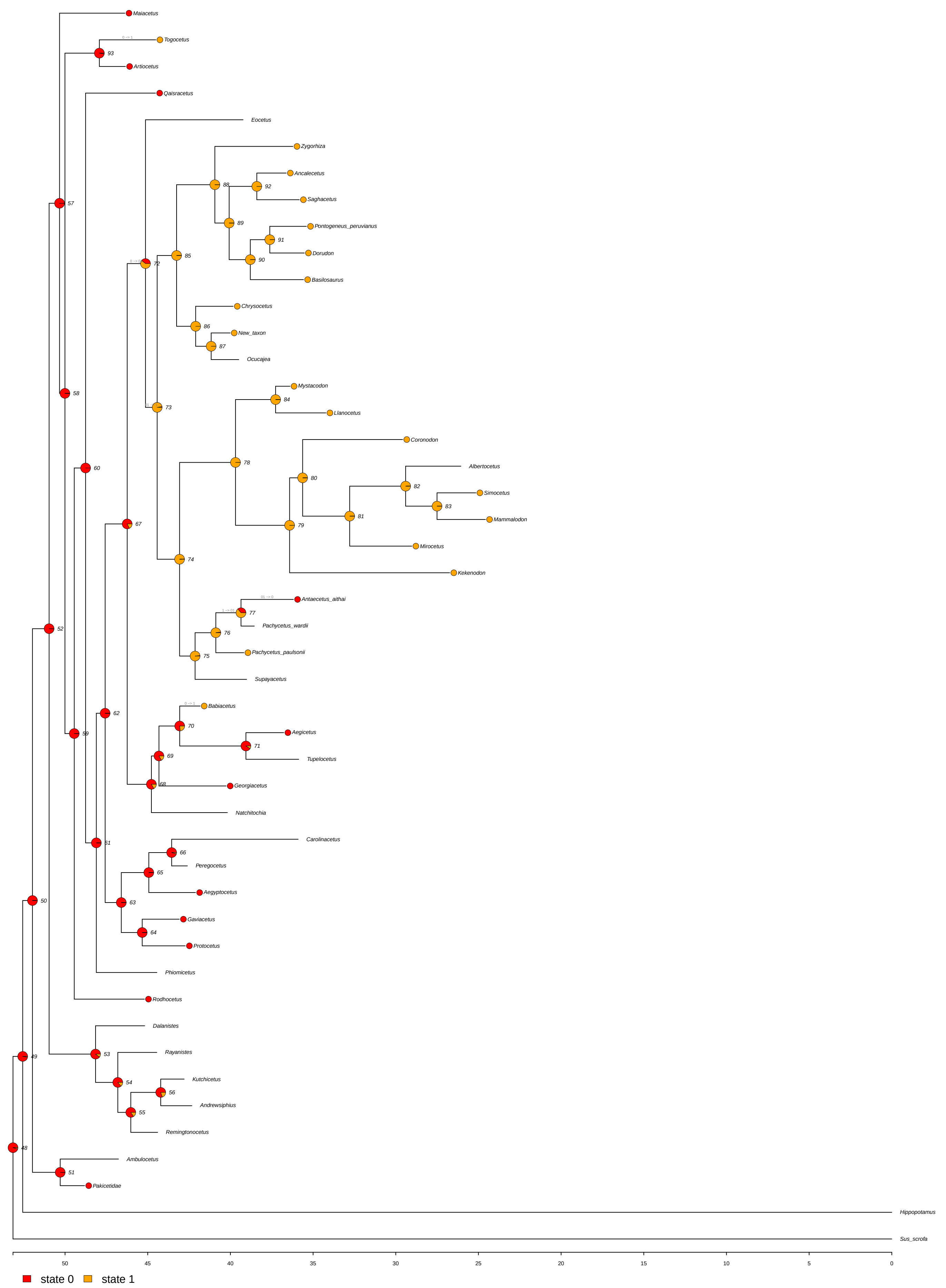
Maiacetus
Togocetus
Artiocetus
Qaisracetus
Eocetus
Zygorhiza
Ancalecetus
Saghacetus
Pontogeneus_peruvianus
Dorudon
Basilosaurus
Chrysocetus
New_taxon
Ocucajea
Mystacodon
Llanocetus
Coronodon
Albertocetus
Simocetus
Mammalodon
Mirocetus
Kekenodon
Antaecetus_aithai
Pachycetus_wardii
Pachycetus_paulsonii
Supayacetus
Babiacetus
Aegicetus
Tupelocetus
Georgiacetus
Natchitochia
Carolinacetus
Peregocetus
Aegyptocetus
Gaviacetus
Protocetus
Phiomicetus
Rodhocetus
Dalanistes
Rayanistes
Kutchicetus
Andrewsiphius
Remingtonocetus
Ambulocetus
Pakicetidae
Hippopotamus
Sus_scrofa
50
45
40
35
30
25
20
15
10
5
0
state 0
state 1

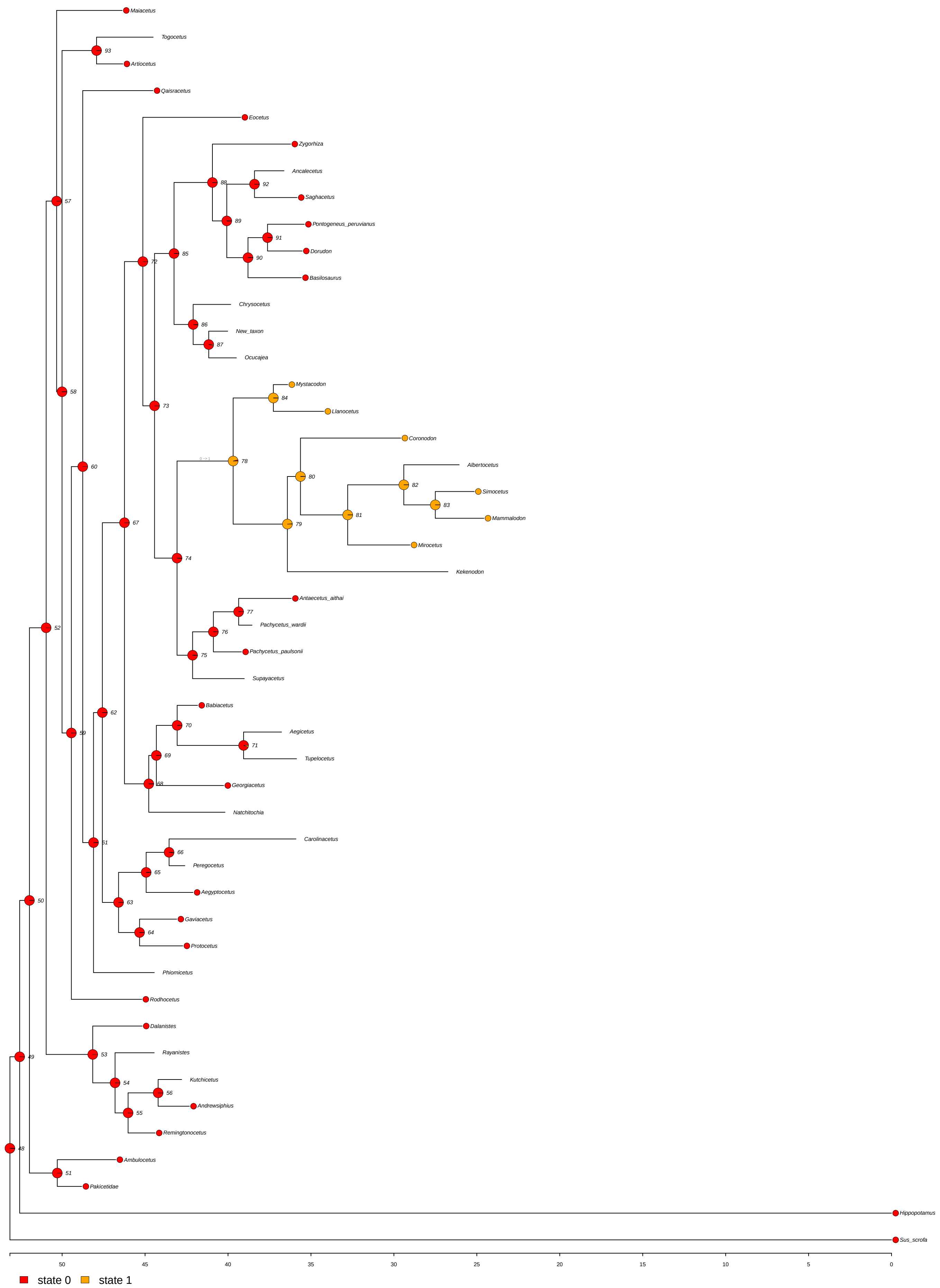

Maiacetus
Togocetus
Artiocetus
Qaisracetus
Eocetus
Zygorhiza
Ancalecetus
Saghacetus
Pontogeneus_peruvianus
Dorudon
Basilosaurus
Chrysocetus
New_taxon
Ocucajea
Mystacodon
Llanocetus
Coronodon
Albertocetus
Simocetus
Mammalodon
Mirocetus
Kekenodon
Antaecetus_aithai
Pachycetus_wardii
Pachycetus_paulsonii
Supayacetus
Babiacetus
Aegicetus
Tupelocetus
Georgiacetus
Natchitochia
Carolinacetus
Peregocetus
Aegyptocetus
Gaviacetus
Protocetus
Phiomicetus
Rodhocetus
Dalanistes
Rayanistes
Kutchicetus
Andrewsiphius
Remingtonocetus
Ambulocetus
Pakicetidae
Hippopotamus
Sus_scrofa
0 --> 1
50
45
40
35
30
25
20
15
10
5
0
state 0
state 1

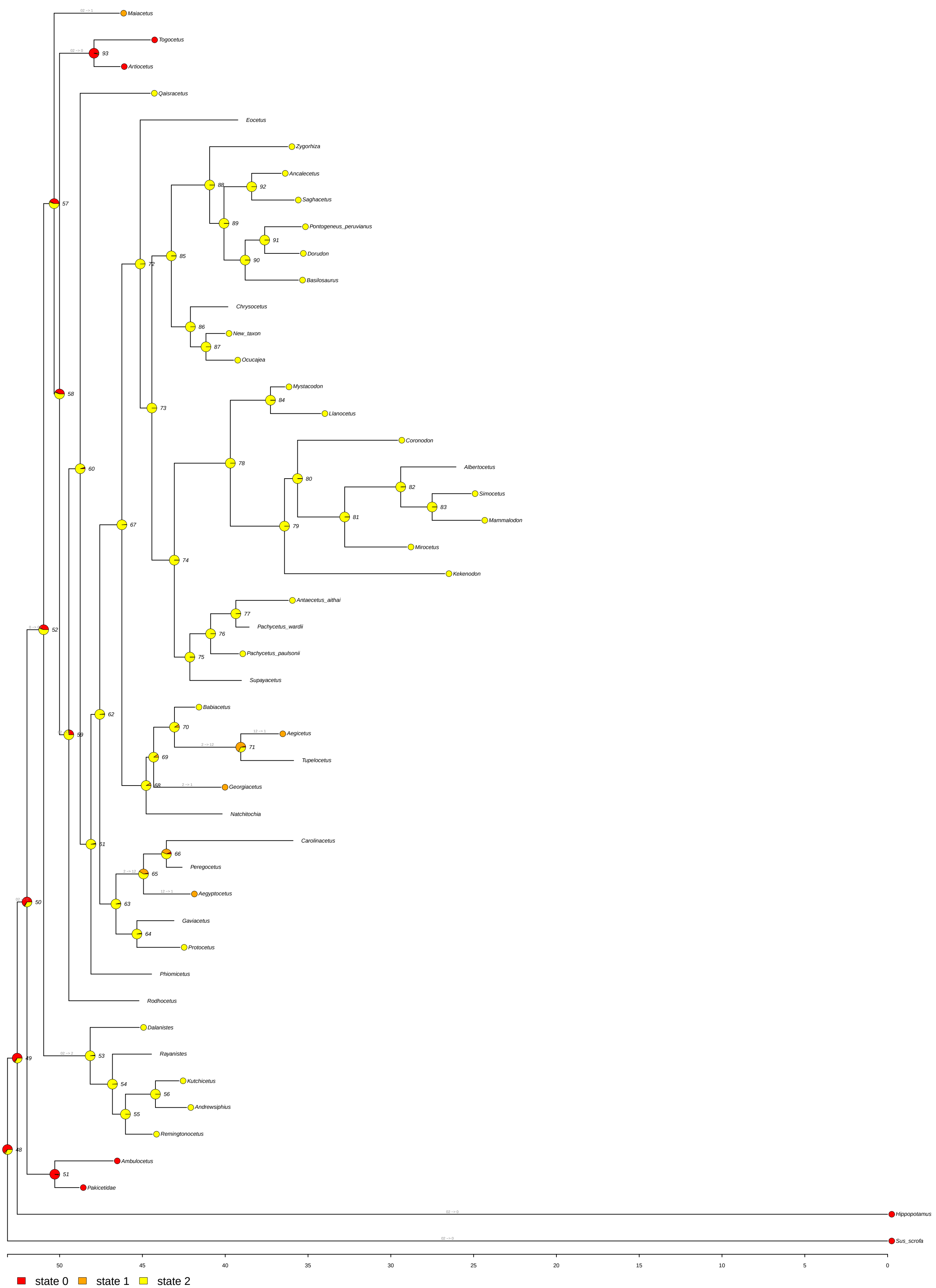

Maiacetus
Togocetus
93
Artiocetus
Qaisracetus
Eocetus
Zygorhiza
Ancalecetus
88
92
Saghacetus
Pontogeneus_peruvianus
89
91
Dorudon
90
Basilosaurus
57
72
85
Chrysocetus
86
New_taxon
87
Ocucajea
Mystacodon
58
84
73
Llanocetus
Coronodon
60
78
80
Albertocetus
82
Simocetus
83
81
67
Mammalodon
79
Mirocetus
74
Kekenodon
Antaecetus_aithai
77
52
Pachycetus_wardii
76
Pachycetus_paulsonii
75
Supayacetus
Babiacetus
62
70
Aegicetus
59
71
69
Tupelocetus
68
Georgiacetus
Natchitochia
61
Carolinacetus
66
Peregocetus
65
Aegyptocetus
50
63
Gaviacetus
64
Protocetus
Phiomicetus
Rodhocetus
Dalanistes
Rayanistes
49
53
54
Kutchicetus
56
Andrewsiphius
55
Remingtonocetus
48
Ambulocetus
51
Pakicetidae
Hippopotamus
Sus_scrofa
50
45
40
35
30
25
20
15
10
5
0
state 0
state 1
state 2

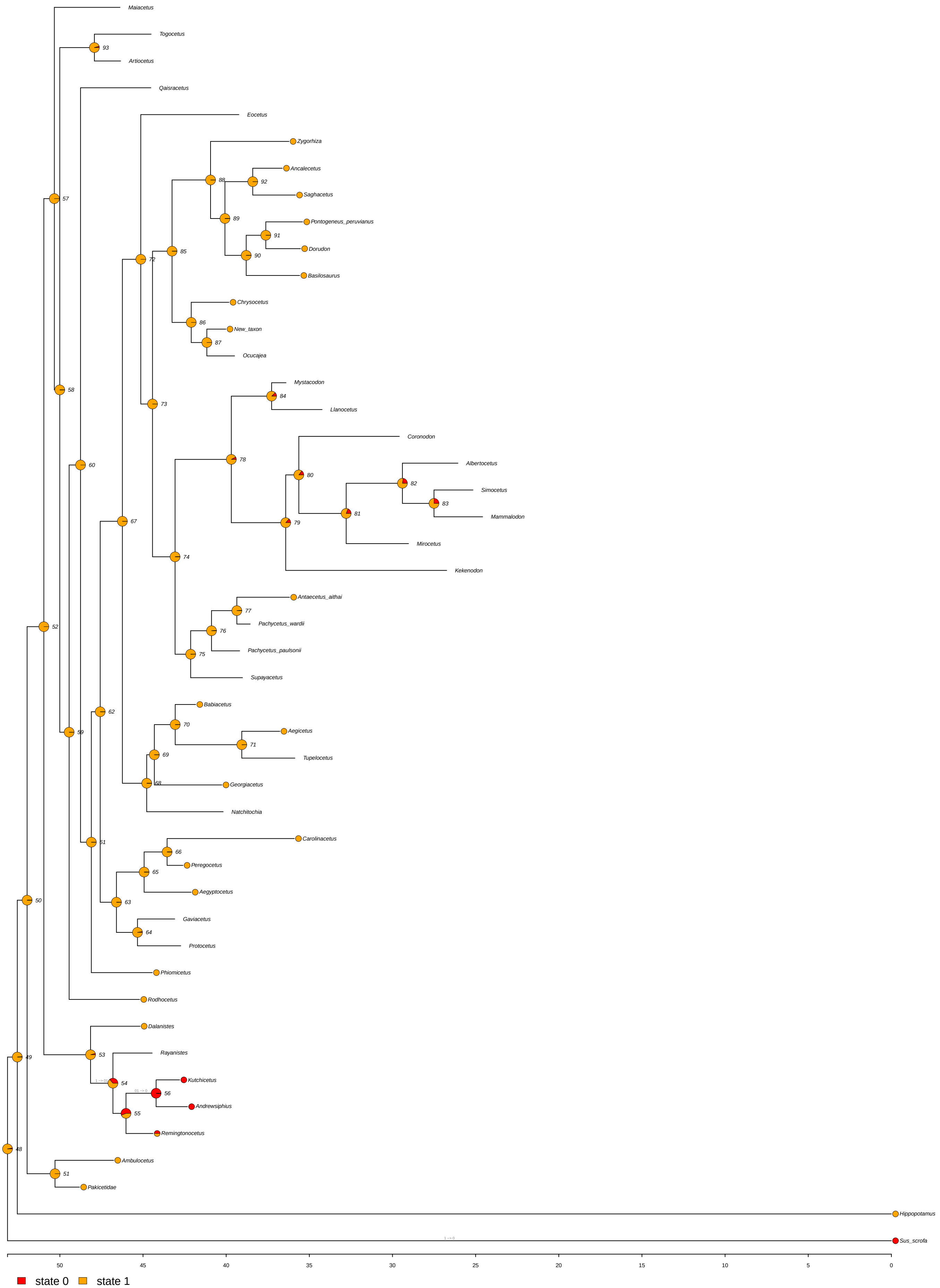

Maiacetus
Togocetus
93
Artiocetus
Qaisracetus
Eocetus
Zygorhiza
Ancalecetus
88
92
Saghacetus
57
Pontogeneus_peruvianus
89
91
Dorudon
90
Basilosaurus
72
85
Chrysocetus
86
New_taxon
87
Ocucajea
Mystacodon
84
58
Llanocetus
73
Coronodon
60
78
Albertocetus
80
82
Simocetus
83
81
Mammalodon
67
79
Mirocetus
74
Kekenodon
Antaecetus_aithai
77
Pachycetus_wardii
76
52
Pachycetus_paulsonii
75
Supayacetus
Babiacetus
62
70
Aegicetus
59
71
Tupelocetus
69
68
Georgiacetus
Natchitochia
Carolinacetus
61
66
Peregocetus
65
Aegyptocetus
50
63
Gaviacetus
64
Protocetus
Phiomicetus
Rodhocetus
Dalanistes
49
53
Rayanistes
Kutchicetus
54
56
Andrewsiphius
55
Remingtonocetus
48
Ambulocetus
51
Pakicetidae
Hippopotamus
Sus_scrofa
50
45
40
35
30
25
20
15
10
5
0
state 0
state 1

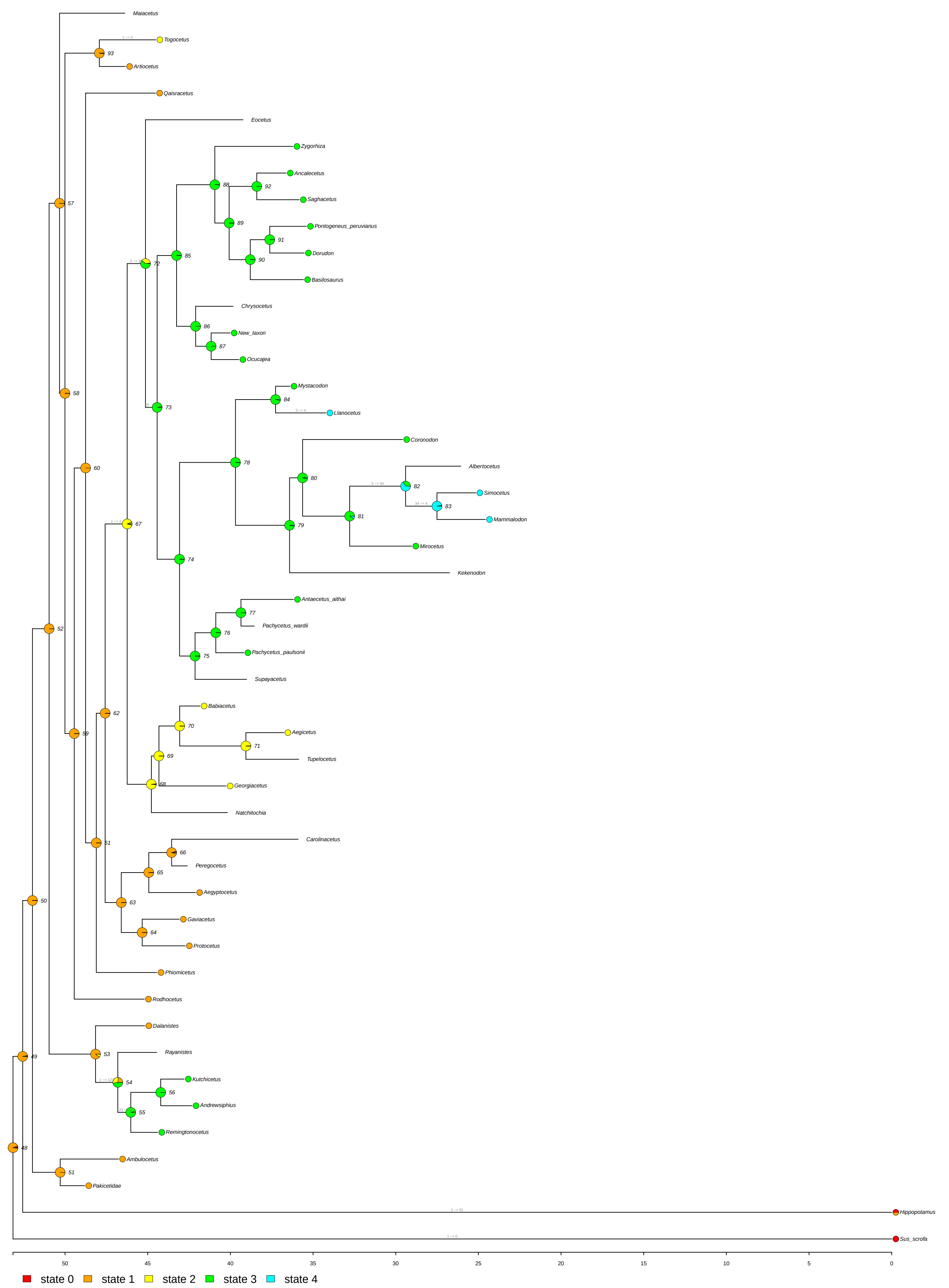

Maiacetus
Togocetus
93
Artiocetus
Qaisracetus
Eocetus
Zygorhiza
Ancalecetus
88
92
Saghacetus
89
Pontogeneus_peruvianus
91
Dorudon
90
Basilosaurus
57
72
85
Chrysocetus
86
New_taxon
87
Ocucajea
Mystacodon
84
Llanocetus
58
73
Coronodon
78
Albertocetus
80
82
Simocetus
83
Mammalodon
81
60
67
79
Mirocetus
74
Kekenodon
Antaecetus_aithai
77
Pachycetus_wardii
52
76
Pachycetus_paulsonii
75
Supayacetus
Babiacetus
62
70
Aegicetus
59
71
Tupelocetus
69
68
Georgiacetus
Natchitochia
Carolinacetus
61
66
Peregocetus
65
50
Aegyptocetus
63
Gaviacetus
64
Protocetus
Phiomicetus
Rodhocetus
Dalanistes
Rayanistes
53
49
54
Kutchicetus
56
Andrewsiphius
55
Remingtonocetus
48
Ambulocetus
51
Pakicetidae
Hippopotamus
Sus_scrofa
50
45
40
35
30
25
20
15
10
5
0
state 0
state 1
state 2
state 3
state 4

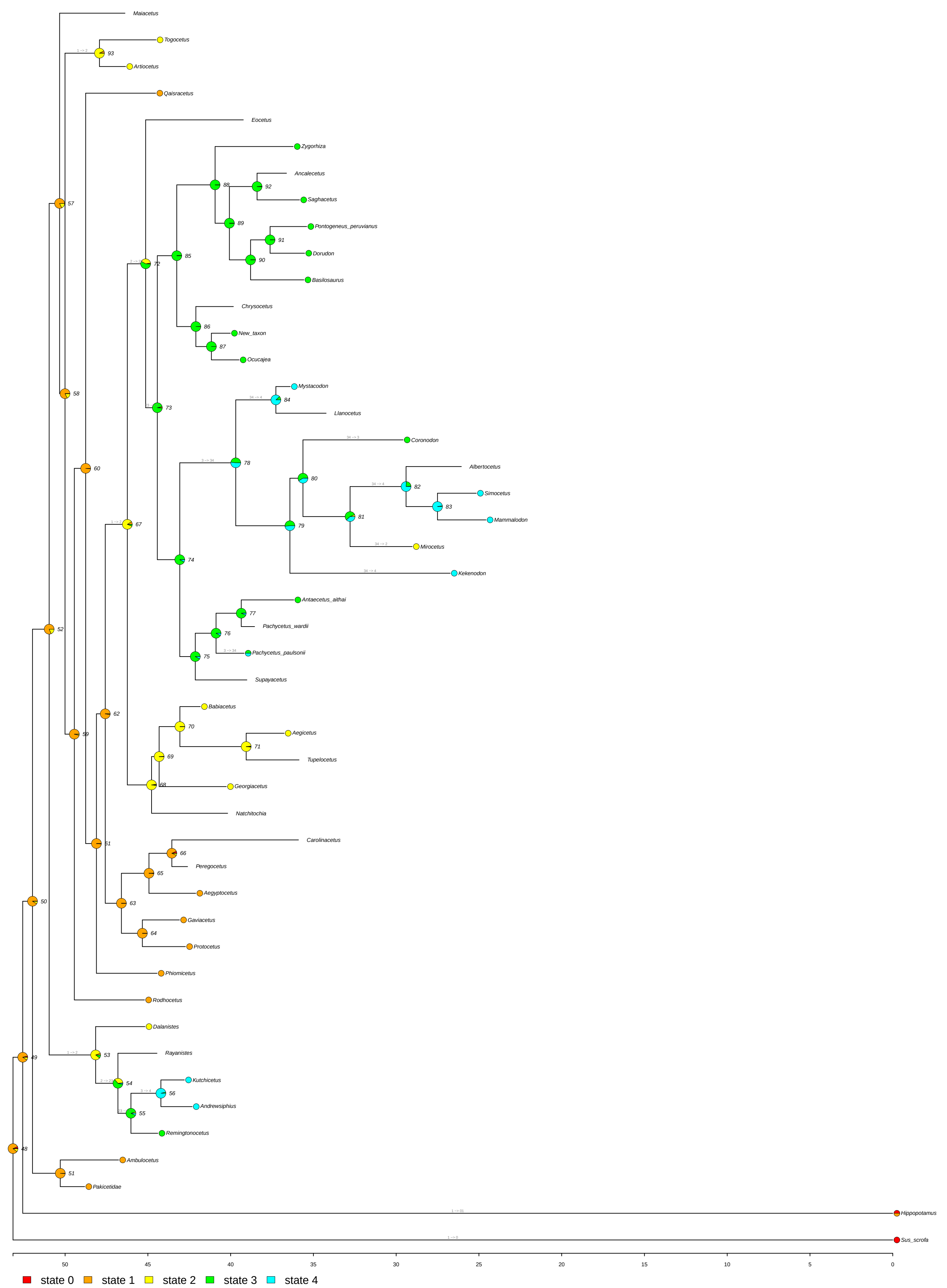
Maiacetus
Togocetus
Artiocetus
Qaisracetus
Eocetus
Zygorhiza
Ancalecetus
Saghacetus
Pontogeneus_peruvianus
Dorudon
Basilosaurus
Chrysocetus
New_taxon
Ocucajea
Mystacodon
Llanocetus
Coronodon
Albertocetus
Simocetus
Mammalodon
Mirocetus
Kekenodon
Antaecetus_aithai
Pachycetus_wardii
Pachycetus_paulsonii
Supayacetus
Babiacetus
Aegicetus
Tupelocetus
Georgiacetus
Natchitochia
Carolinacetus
Peregocetus
Aegyptocetus
Gaviacetus
Protocetus
Phiomicetus
Rodhocetus
Dalanistes
Rayanistes
Kutchicetus
Andrewsiphius
Remingtonocetus
Ambulocetus
Pakicetidae
Hippopotamus
Sus_scrofa
50
45
40
35
30
25
20
15
10
5
0
state 0
state 1
state 2
state 3
state 4

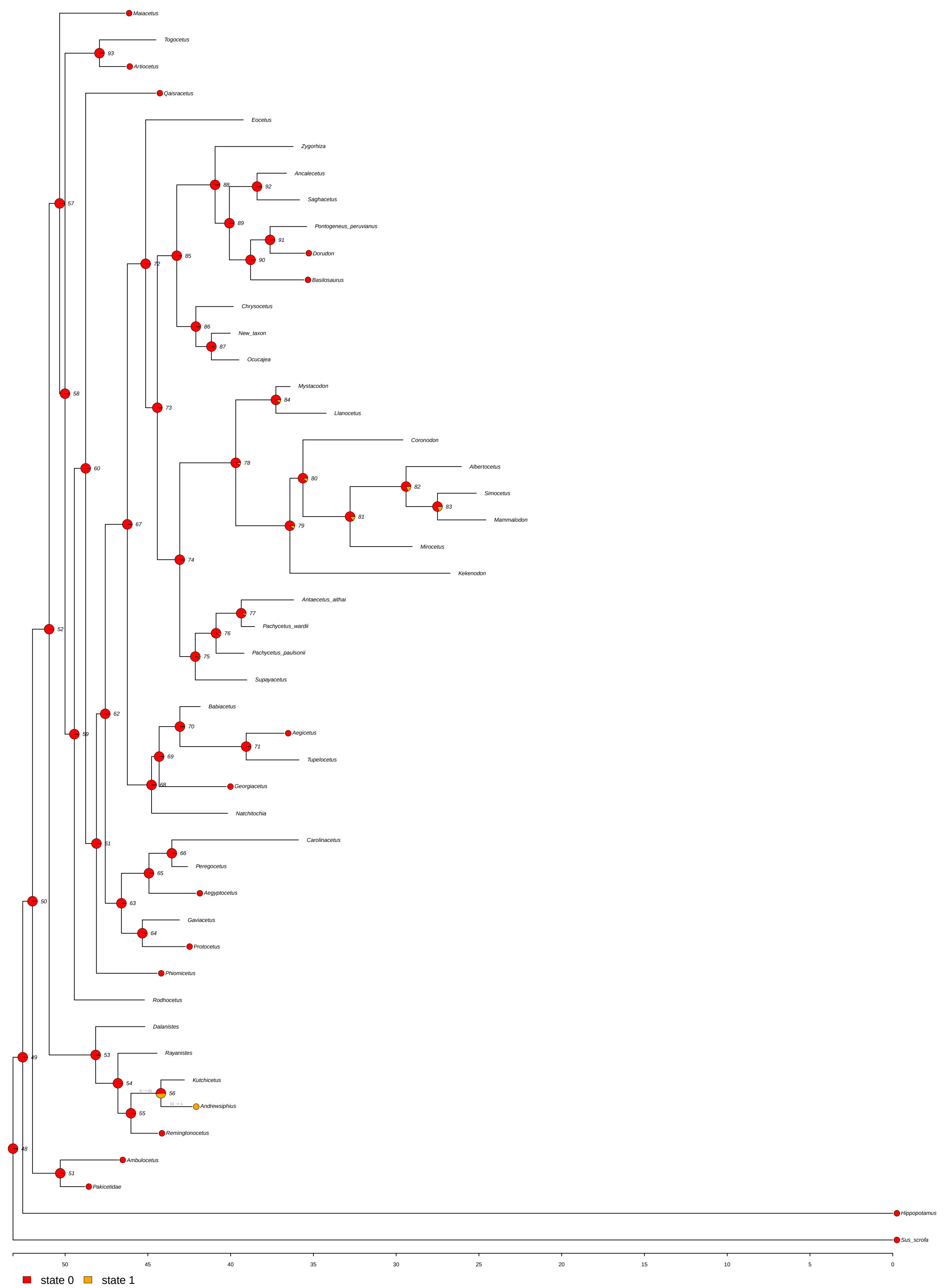
Maiacetus
Togocetus
Artiocetus
Qaisracetus
Eocetus
Zygorhiza
Ancalecetus
Saghacetus
Pontogeneus_peruvianus
Dorudon
Basilosaurus
Chrysocetus
New_taxon
Ocucajea
Mystacodon
Llanocetus
Coronodon
Albertocetus
Simocetus
Mammalodon
Mirocetus
Kekenodon
Antaecetus_aithai
Pachycetus_wardii
Pachycetus_paulsonii
Supayacetus
Babiacetus
Aegicetus
Tupelocetus
Georgiacetus
Natchitochia
Carolinacetus
Peregocetus
Aegyptocetus
Gaviacetus
Protocetus
Phiomicetus
Rodhocetus
Dalanistes
Rayanistes
Kutchicetus
Andrewsiphius
Remingtonocetus
Ambulocetus
Pakicetidae
Hippopotamus
Sus_scrofa
50
45
40
35
30
25
20
15
10
5
0
state 0
state 1

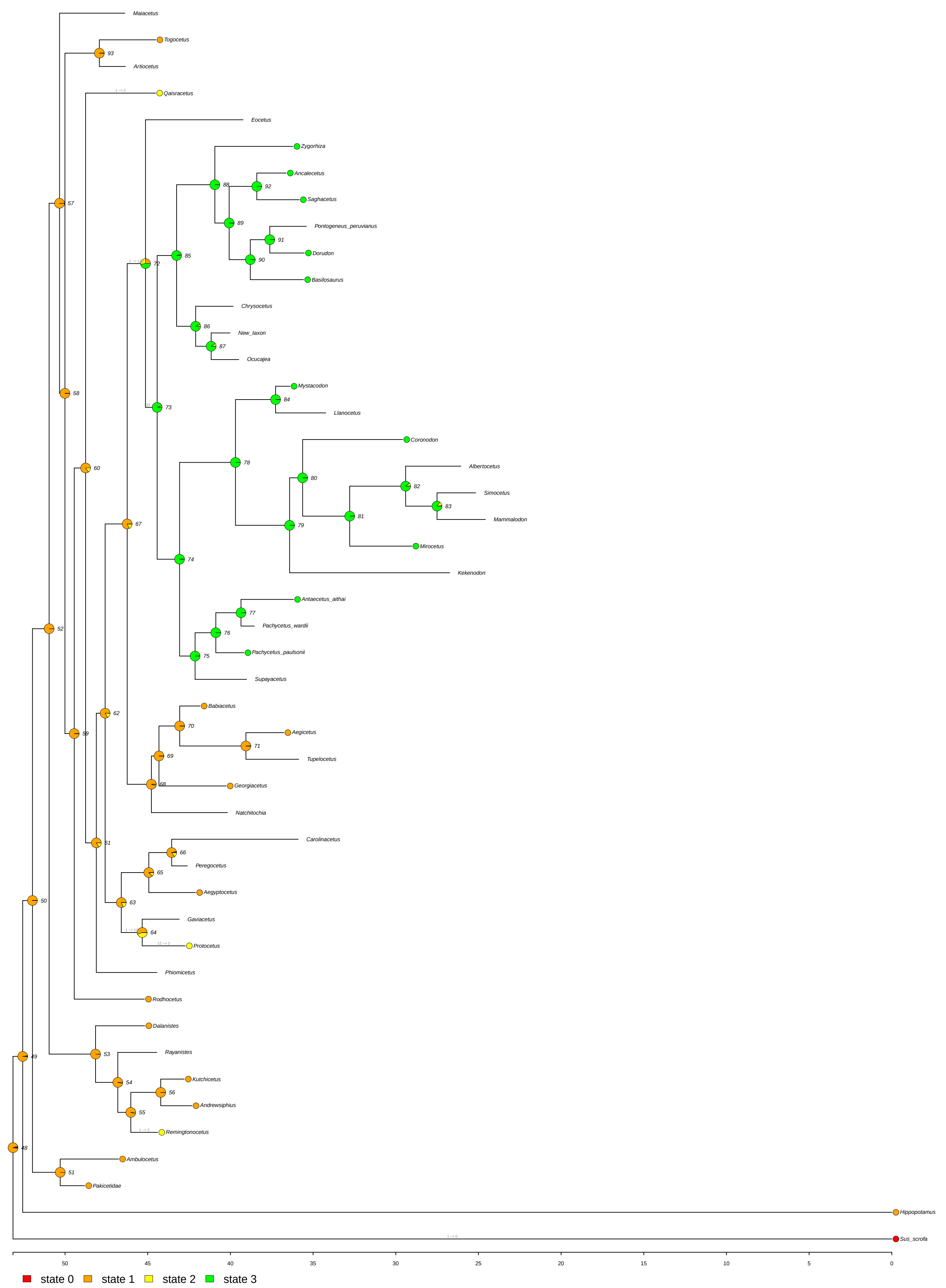

Maiacetus
Togocetus
93
Artiocetus
1 -> 2
Qaisracetus
Eocetus
Zygorhiza
Ancalecetus
88
92
Saghacetus
89
Pontogeneus_peruvianus
91
Dorudon
85
90
72
Basilosaurus
57
Chrysocetus
86
New_taxon
87
Ocucajea
Mystacodon
58
84
73
Llanocetus
Coronodon
78
60
Albertocetus
80
82
Simocetus
83
Mammalodon
81
67
79
Mirocetus
74
Kekenodon
Antaecetus_aithai
77
Pachycetus_wardii
76
52
Pachycetus_paulsonii
75
Supayacetus
Babiacetus
62
70
Aegicetus
59
71
Tupelocetus
69
68
Georgiacetus
Natchitochia
Carolinacetus
61
66
Peregocetus
65
Aegyptocetus
50
63
Gaviacetus
64
Protocetus
Phiomicetus
Rodhocetus
Dalanistes
Rayanistes
53
49
54
Kutchicetus
56
Andrewsiphius
55
1 -> 2
Remingtonocetus
48
Ambulocetus
51
Pakicetidae
Hippopotamus
1 -> 0
Sus_scrofa
50
45
40
35
30
25
20
15
10
5
0
state 0
state 1
state 2
state 3

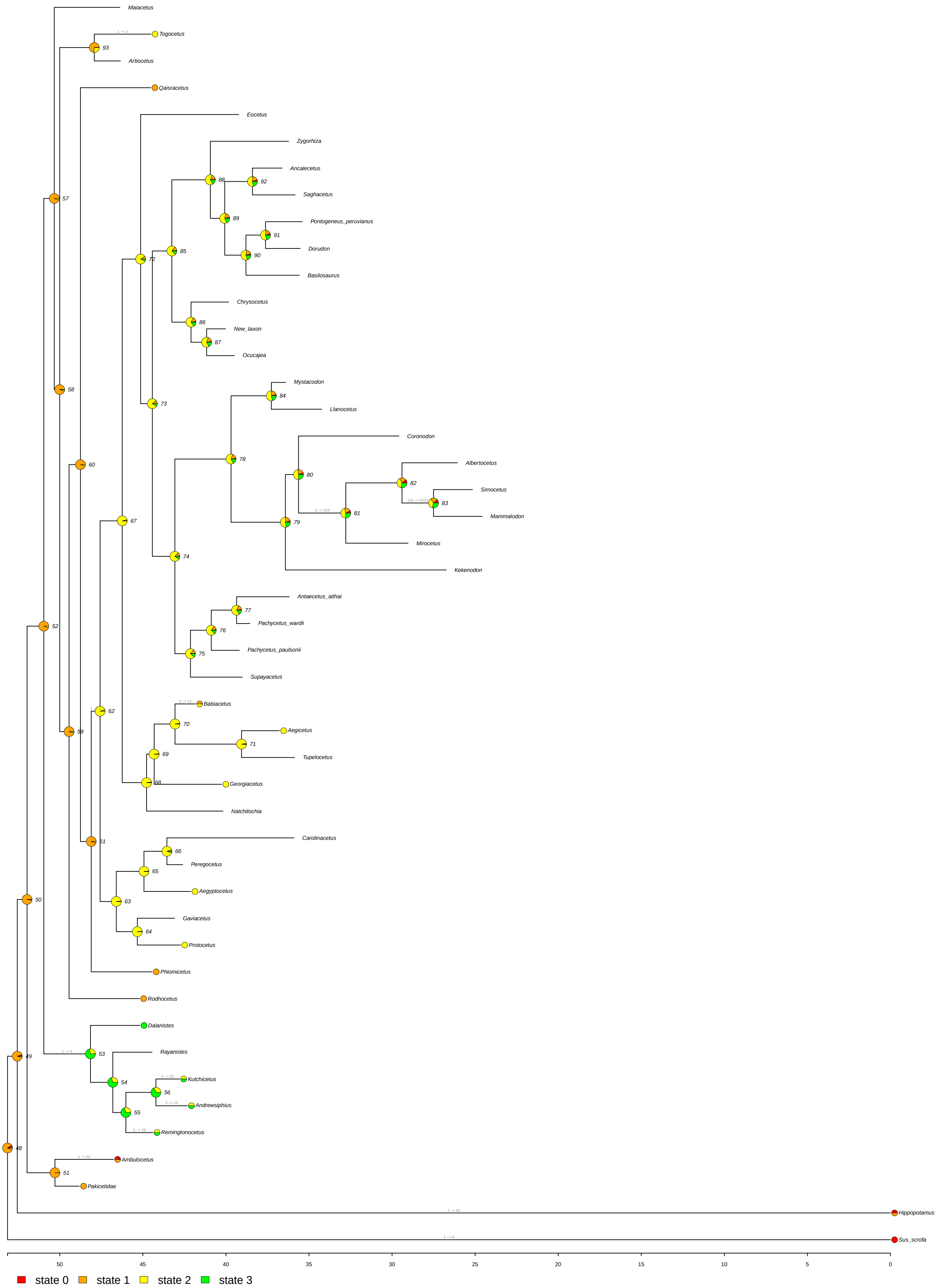

Maiacetus
Togocetus
93
Artiocetus
Qaisracetus
Eocetus
Zygorhiza
Ancalecetus
88
92
Saghacetus
89
Pontogeneus_peruvianus
91
Dorudon
90
Basilosaurus
57
72
85
Chrysocetus
86
New_taxon
87
Ocucajea
Mystacodon
84
Llanocetus
58
73
Coronodon
78
60
Albertocetus
80
82
Simocetus
83
Mammalodon
81
79
67
Mirocetus
74
Kekenodon
Antaecetus_aithai
77
Pachycetus_wardii
76
52
Pachycetus_paulsonii
75
Supayacetus
Babiacetus
62
70
Aegicetus
59
71
Tupelocetus
69
68
Georgiacetus
Natchitochia
Carolinacetus
61
66
Peregocetus
65
50
Aegyptocetus
63
Gaviacetus
64
Protocetus
Phiomicetus
Rodhocetus
Dalanistes
Rayanistes
49
53
54
Kutchicetus
56
Andrewsiphius
55
Remingtonocetus
48
Ambulocetus
51
Pakicetidae
Hippopotamus
Sus_scrofa
50
45
40
35
30
25
20
15
10
5
0
state 0
state 1
state 2
state 3

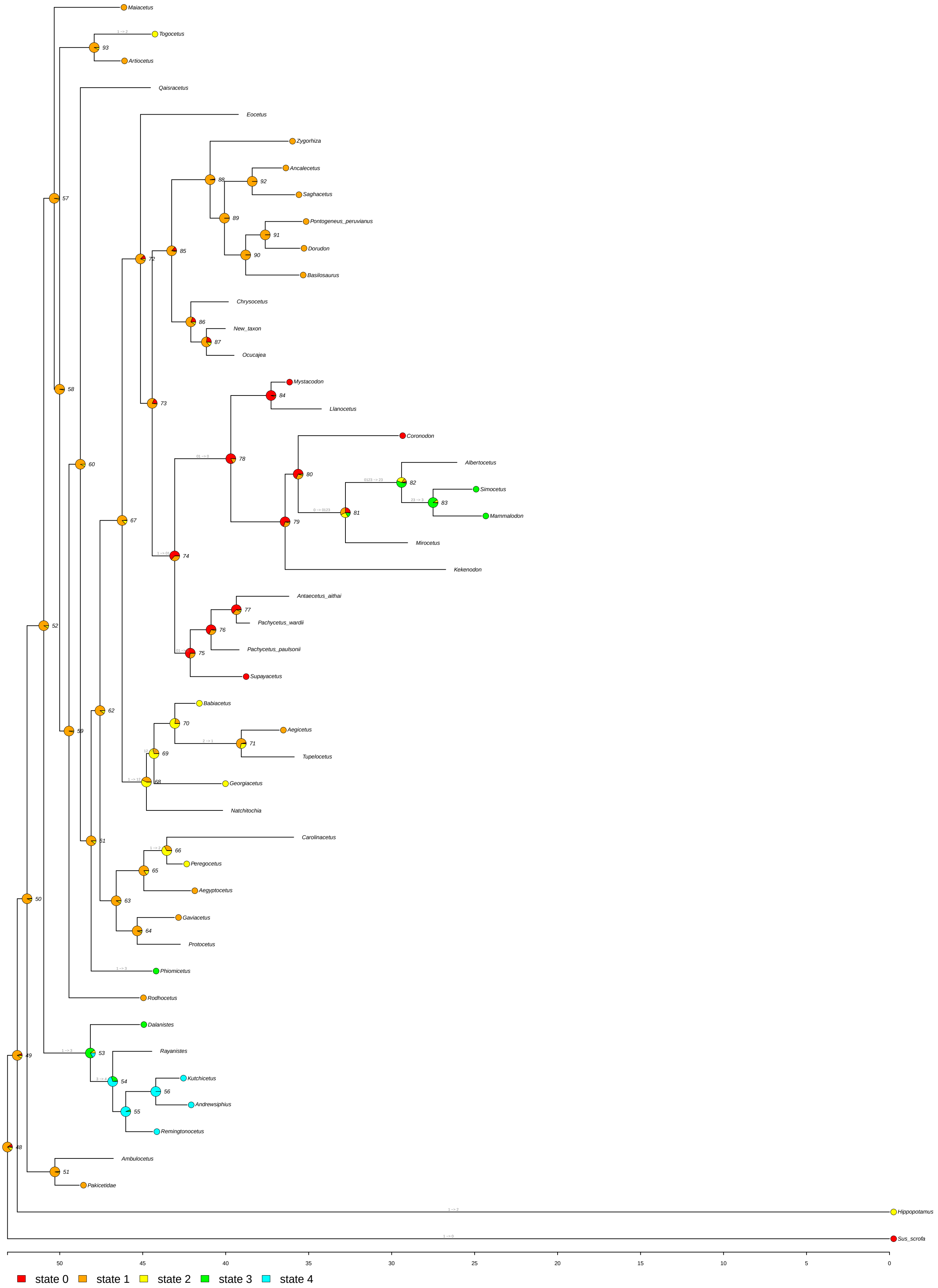

Maiacetus
Togocetus
Artiocetus
Qaisracetus
Eocetus
Zygorhiza
Ancalecetus
Saghacetus
Pontogeneus_peruvianus
Dorudon
Basilosaurus
Chrysocetus
New_taxon
Ocucajea
Mystacodon
Llanocetus
Coronodon
Albertocetus
Simocetus
Mammalodon
Mirocetus
Kekenodon
Antaecetus_aithai
Pachycetus_wardii
Pachycetus_paulsonii
Supayacetus
Babiacetus
Aegicetus
Tupelocetus
Georgiacetus
Natchitochia
Carolinacetus
Peregocetus
Aegyptocetus
Gaviacetus
Protocetus
Phiomicetus
Rodhocetus
Dalanistes
Rayanistes
Kutchicetus
Andrewsiphius
Remingtonocetus
Ambulocetus
Pakicetidae
Hippopotamus
Sus_scrofa
50
45
40
35
30
25
20
15
10
5
0
state 0
state 1
state 2
state 3
state 4

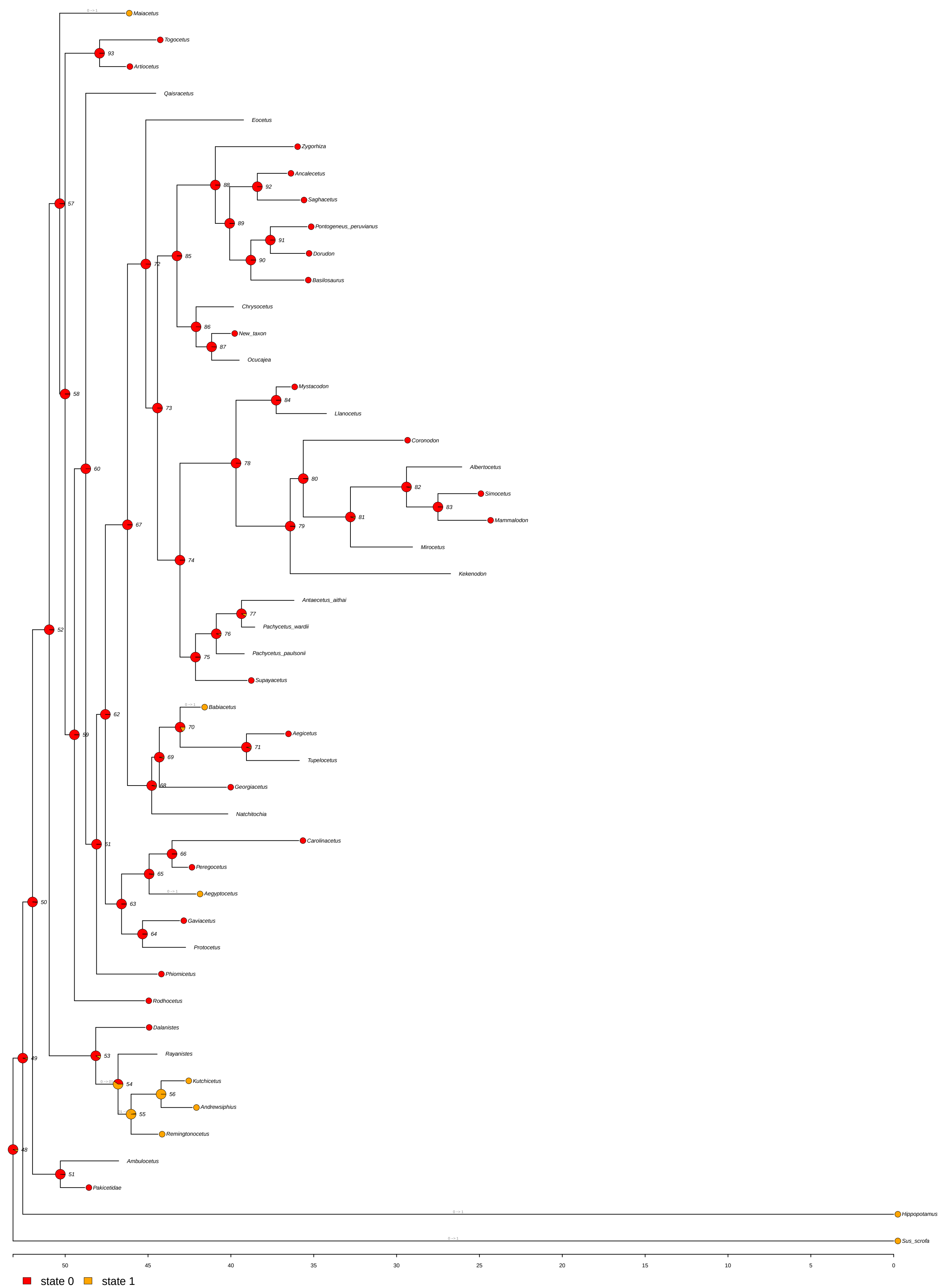

Maiacetus
Togocetus
Artiocetus
Qaisracetus
Eocetus
Zygorhiza
Ancalecetus
Saghacetus
Pontogeneus_peruvianus
Dorudon
Basilosaurus
Chrysocetus
New_taxon
Ocucajea
Mystacodon
Llanocetus
Coronodon
Albertocetus
Simocetus
Mammalodon
Mirocetus
Kekenodon
Antaecetus_aithai
Pachycetus_wardii
Pachycetus_paulsonii
Supayacetus
Babiacetus
Aegicetus
Tupelocetus
Georgiacetus
Natchitochia
Carolinacetus
Peregocetus
Aegyptocetus
Gaviacetus
Protocetus
Phiomicetus
Rodhocetus
Dalanistes
Rayanistes
Kutchicetus
Andrewsiphius
Remingtonocetus
Ambulocetus
Pakicetidae
Hippopotamus
Sus_scrofa
50
45
40
35
30
25
20
15
10
5
0
state 0
state 1

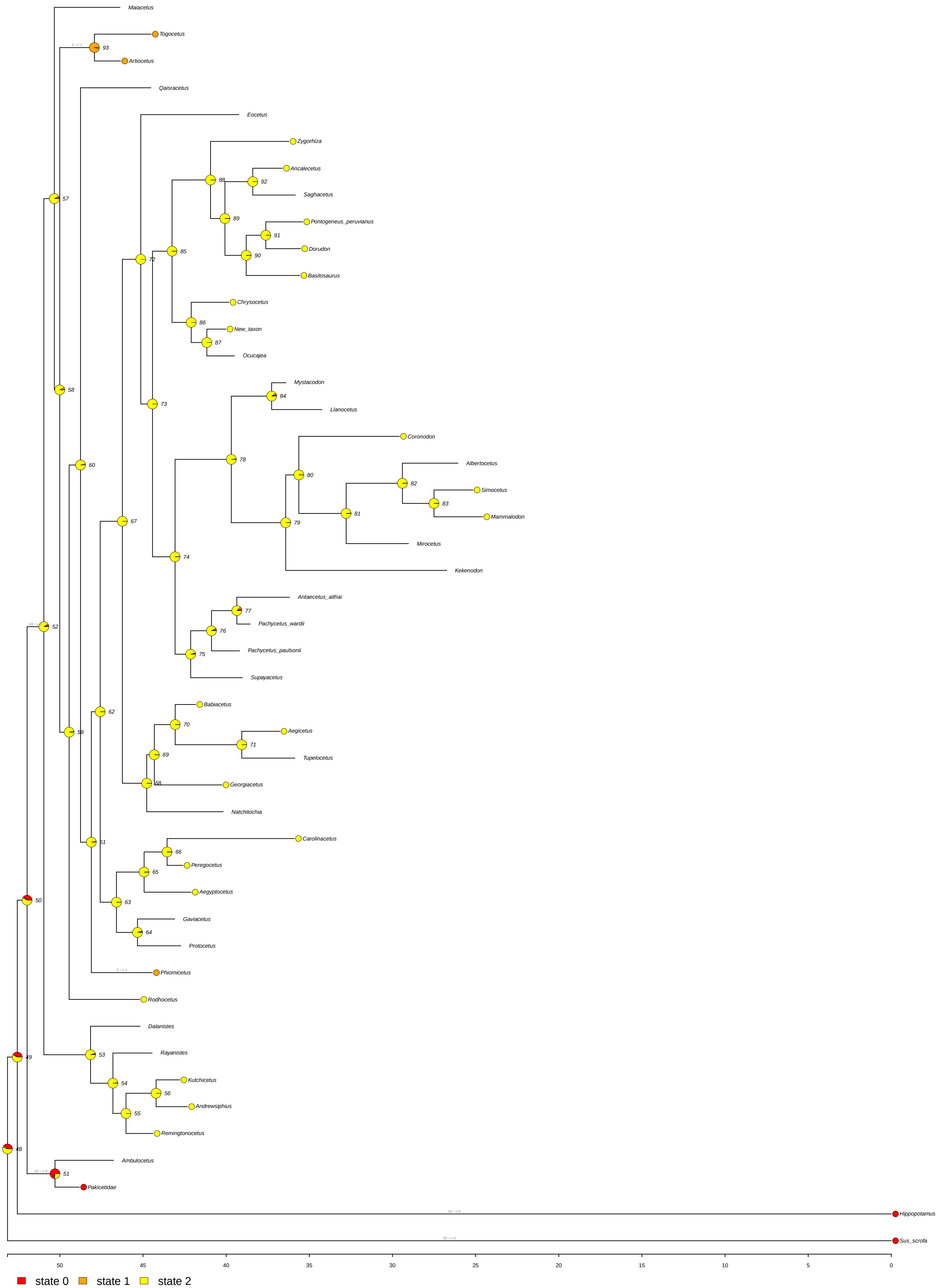

Maiacetus
Togocetus
93
Artiocetus
Qaisracetus
Eocetus
Zygorhiza
Ancalecetus
88
92
Saghacetus
Pontogeneus_peruvianus
89
91
Dorudon
90
Basilosaurus
72
85
Chrysocetus
86
New_taxon
87
Ocucajea
Mystacodon
84
Llanocetus
73
Coronodon
78
80
Albertocetus
82
Simocetus
83
Mammalodon
81
Mirocetus
79
67
74
Kekenodon
Antaecetus_aithai
77
Pachycetus_wardii
76
Pachycetus_paulsonii
75
Supayacetus
57
58
60
52
62
Babiacetus
70
Aegicetus
71
Tupelocetus
69
59
68
Georgiacetus
Natchitochia
Carolinacetus
61
66
Peregocetus
65
Aegyptocetus
50
63
Gaviacetus
64
Protocetus
Phiomicetus
Rodhocetus
Dalanistes
53
Rayanistes
49
54
Kutchicetus
56
55
Andrewsiphius
Remingtonocetus
48
Ambulocetus
51
Pakicetidae
Hippopotamus
Sus_scrofa
50
45
40
35
30
25
20
15
10
5
0
state 0
state 1
state 2

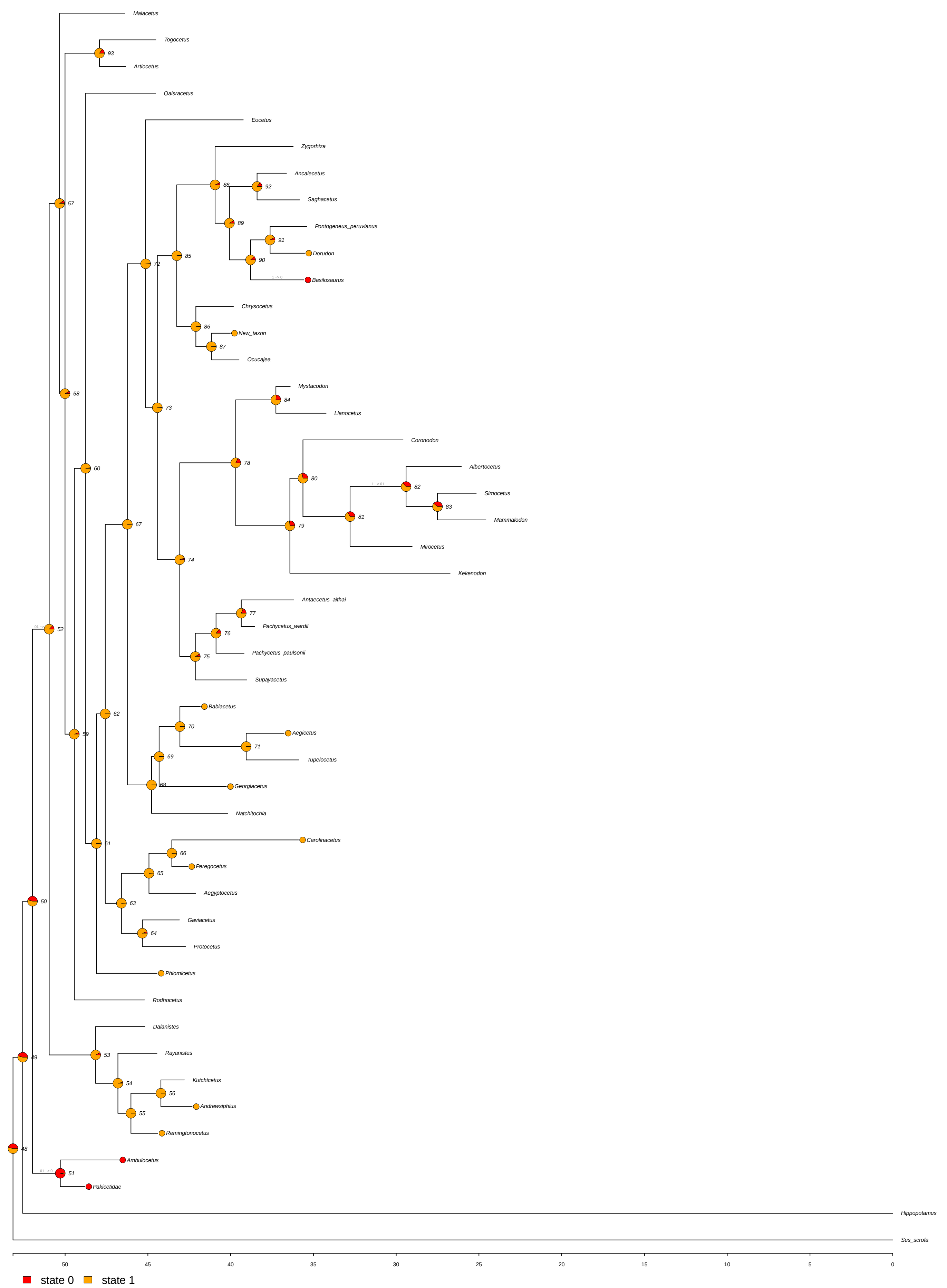

Maiacetus
Togocetus
Artiocetus
Qaisracetus
Eocetus
Zygorhiza
Ancalecetus
Saghacetus
Pontogeneus_peruvianus
Dorudon
Basilosaurus
Chrysocetus
New_taxon
Ocucajea
Mystacodon
Llanocetus
Coronodon
Albertocetus
Simocetus
Mammalodon
Mirocetus
Kekenodon
Antaecetus_aithai
Pachycetus_wardii
Pachycetus_paulsonii
Supayacetus
Babiacetus
Aegicetus
Tupelocetus
Georgiacetus
Natchitochia
Carolinacetus
Peregocetus
Aegyptocetus
Gaviacetus
Protocetus
Phiomicetus
Rodhocetus
Dalanistes
Rayanistes
Kutchicetus
Andrewsiphius
Remingtonocetus
Ambulocetus
Pakicetidae
Hippopotamus
Sus_scrofa
1 -> 0
1 -> 01
01 -> 0
50
45
40
35
30
25
20
15
10
5
0
state 0
state 1

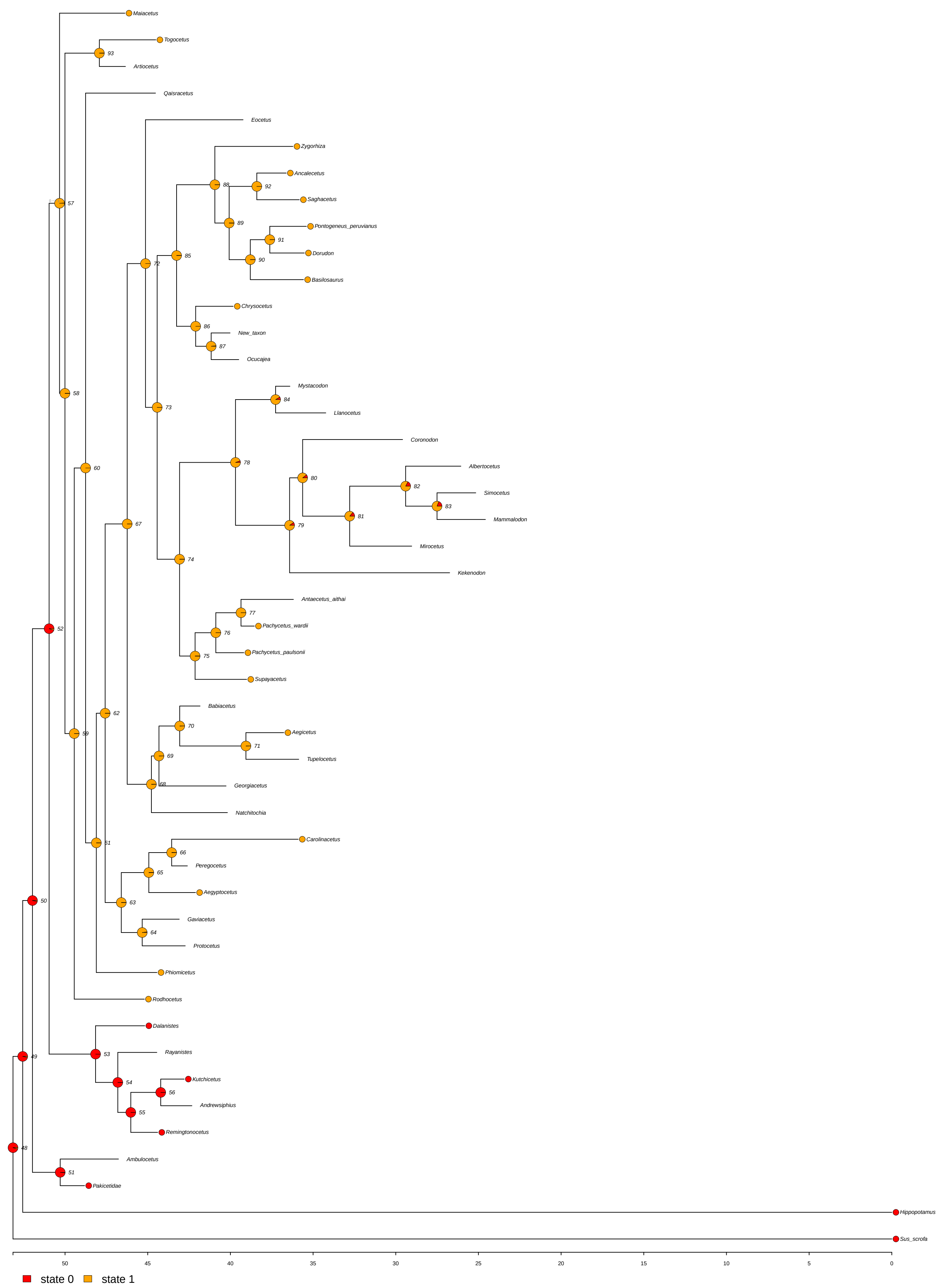

Maiacetus
Togocetus
93
Artiocetus
Qaisracetus
Eocetus
Zygorhiza
Ancalecetus
88
92
Saghacetus
57
Pontogeneus_peruvianus
89
91
Dorudon
90
Basilosaurus
72
85
Chrysocetus
86
New_taxon
87
Ocucajea
Mystacodon
84
58
Llanocetus
73
Coronodon
78
80
Albertocetus
60
82
Simocetus
83
81
Mammalodon
67
79
Mirocetus
74
Kekenodon
Antaecetus_aithai
77
52
Pachycetus_wardii
76
Pachycetus_paulsonii
75
Supayacetus
Babiacetus
62
70
Aegicetus
59
71
Tupelocetus
69
68
Georgiacetus
Natchitochia
Carolinacetus
61
66
Peregocetus
65
Aegyptocetus
50
63
Gaviacetus
64
Protocetus
Phiomicetus
Rodhocetus
Dalanistes
53
Rayanistes
49
Kutchicetus
54
56
Andrewsiphius
55
Remingtonocetus
48
Ambulocetus
51
Pakicetidae
Hippopotamus
Sus_scrofa
50
45
40
35
30
25
20
15
10
5
0
state 0
state 1

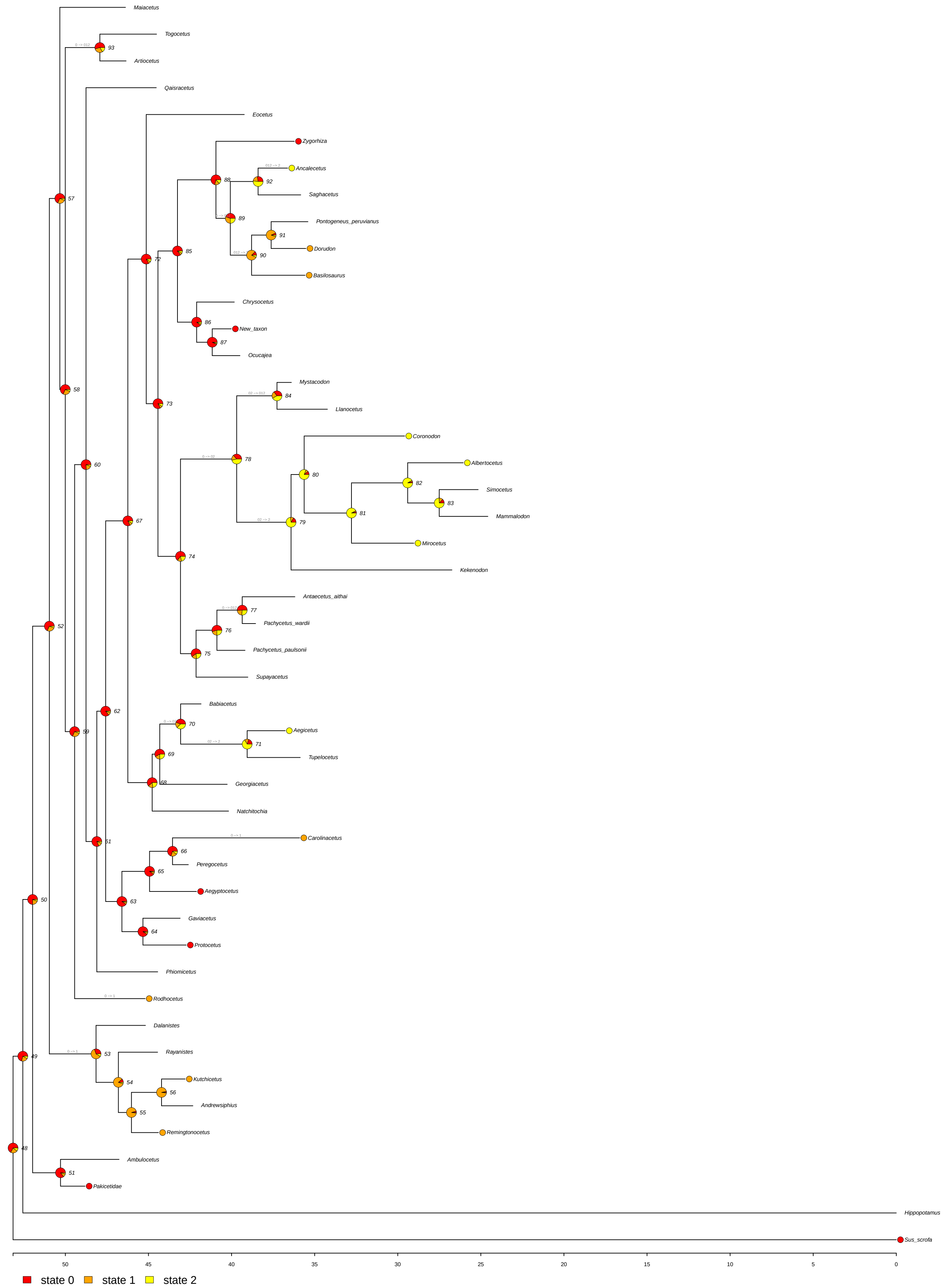

Maiacetus
Togocetus
Artiocetus
Qaisracetus
Eocetus
Zygorhiza
Ancalecetus
Saghacetus
Pontogeneus_peruvianus
Dorudon
Basilosaurus
Chrysocetus
New_taxon
Ocucajea
Mystacodon
Llanocetus
Coronodon
Albertocetus
Simocetus
Mammalodon
Mirocetus
Kekenodon
Antaecetus_aithai
Pachycetus_wardii
Pachycetus_paulsonii
Supayacetus
Babiacetus
Aegicetus
Tupelocetus
Georgiacetus
Natchitochia
Carolinacetus
Peregocetus
Aegyptocetus
Gaviacetus
Protocetus
Phiomicetus
Rodhocetus
Dalanistes
Rayanistes
Kutchicetus
Andrewsiphius
Remingtonocetus
Ambulocetus
Pakicetidae
Hippopotamus
Sus_scrofa
50
45
40
35
30
25
20
15
10
5
0
state 0
state 1
state 2

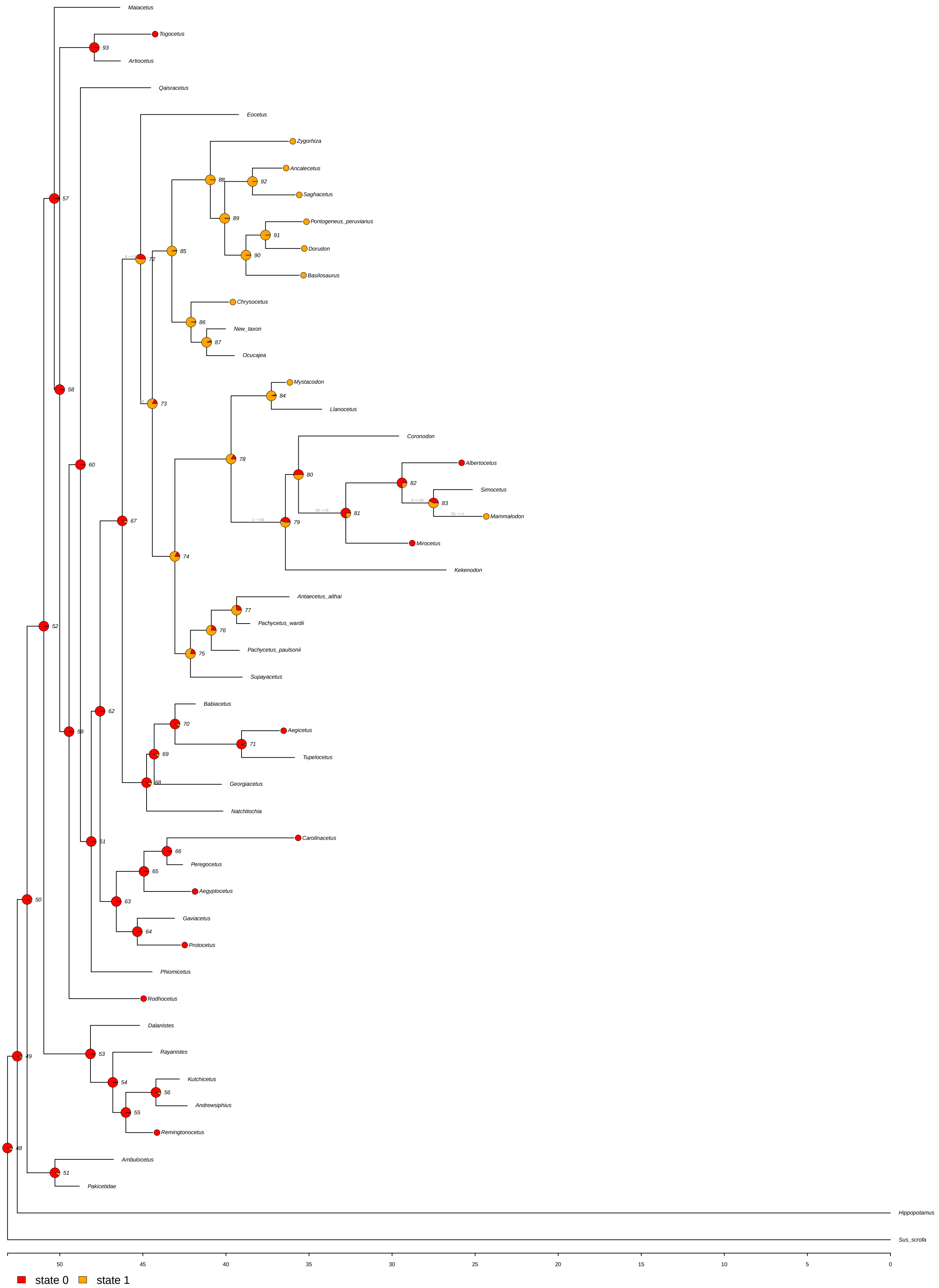

Maiacetus
Togocetus
93
Artiocetus
Qaisracetus
Eocetus
Zygorhiza
Ancalecetus
88
92
Saghacetus
Pontogeneus_peruvianus
89
91
Dorudon
90
Basilosaurus
72
85
57
Chrysocetus
86
New_taxon
87
Ocucajea
Mystacodon
84
Llanocetus
58
73
Coronodon
78
Albertocetus
60
80
82
Simocetus
83
81
Mammalodon
67
79
Mirocetus
74
Kekenodon
Antaecetus_aithai
77
Pachycetus_wardii
52
76
Pachycetus_paulsonii
75
Supayacetus
Babiacetus
62
70
Aegicetus
59
71
Tupelocetus
69
68
Georgiacetus
Natchitochia
Carolinacetus
61
66
Peregocetus
65
Aegyptocetus
50
63
Gaviacetus
64
Protocetus
Phiomicetus
Rodhocetus
Dalanistes
53
Rayanistes
49
54
Kutchicetus
56
Andrewsiphius
55
Remingtonocetus
48
Ambulocetus
51
Pakicetidae
Hippopotamus
Sus_scrofa
50
45
40
35
30
25
20
15
10
5
0
state 0
state 1

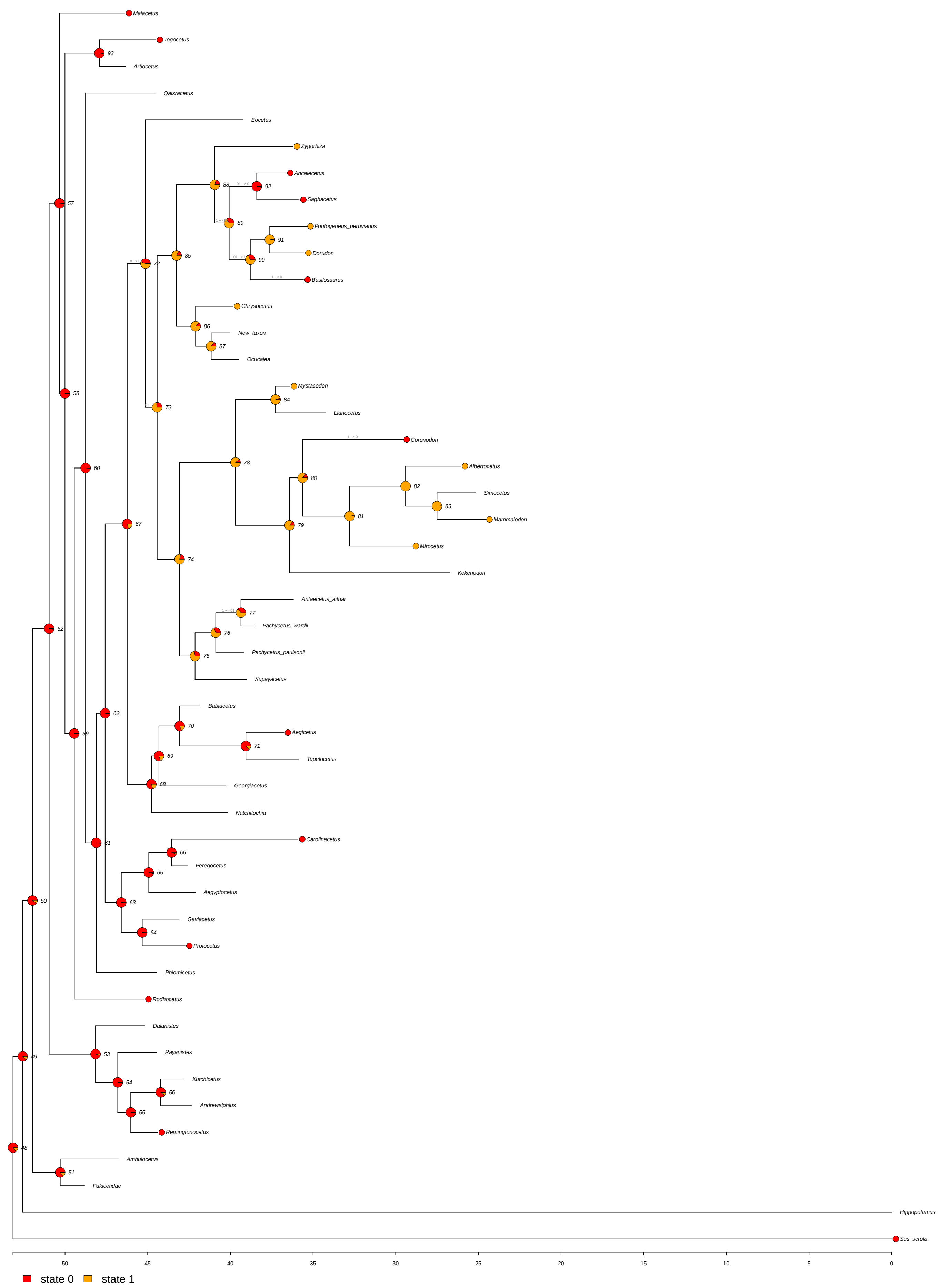

Maiacetus
Togocetus
93
Artiocetus
Qaisracetus
Eocetus
Zygorhiza
Ancalecetus
88
92
Saghacetus
57
89
Pontogeneus_peruvianus
91
Dorudon
85
90
72
Basilosaurus
Chrysocetus
86
New_taxon
87
Ocucajea
Mystacodon
84
58
73
Llanocetus
Coronodon
78
Albertocetus
80
60
82
Simocetus
83
81
Mammalodon
67
79
Mirocetus
74
Kekenodon
Antaecetus_aithai
77
Pachycetus_wardii
76
52
Pachycetus_paulsonii
75
Supayacetus
Babiacetus
62
70
Aegicetus
59
71
Tupelocetus
69
68
Georgiacetus
Natchitochia
Carolinacetus
61
66
Peregocetus
65
Aegyptocetus
50
63
Gaviacetus
64
Protocetus
Phiomicetus
Rodhocetus
Dalanistes
53
Rayanistes
49
54
Kutchicetus
56
Andrewsiphius
55
Remingtonocetus
48
Ambulocetus
51
Pakicetidae
Hippopotamus
Sus_scrofa
50
45
40
35
30
25
20
15
10
5
0
state 0
state 1

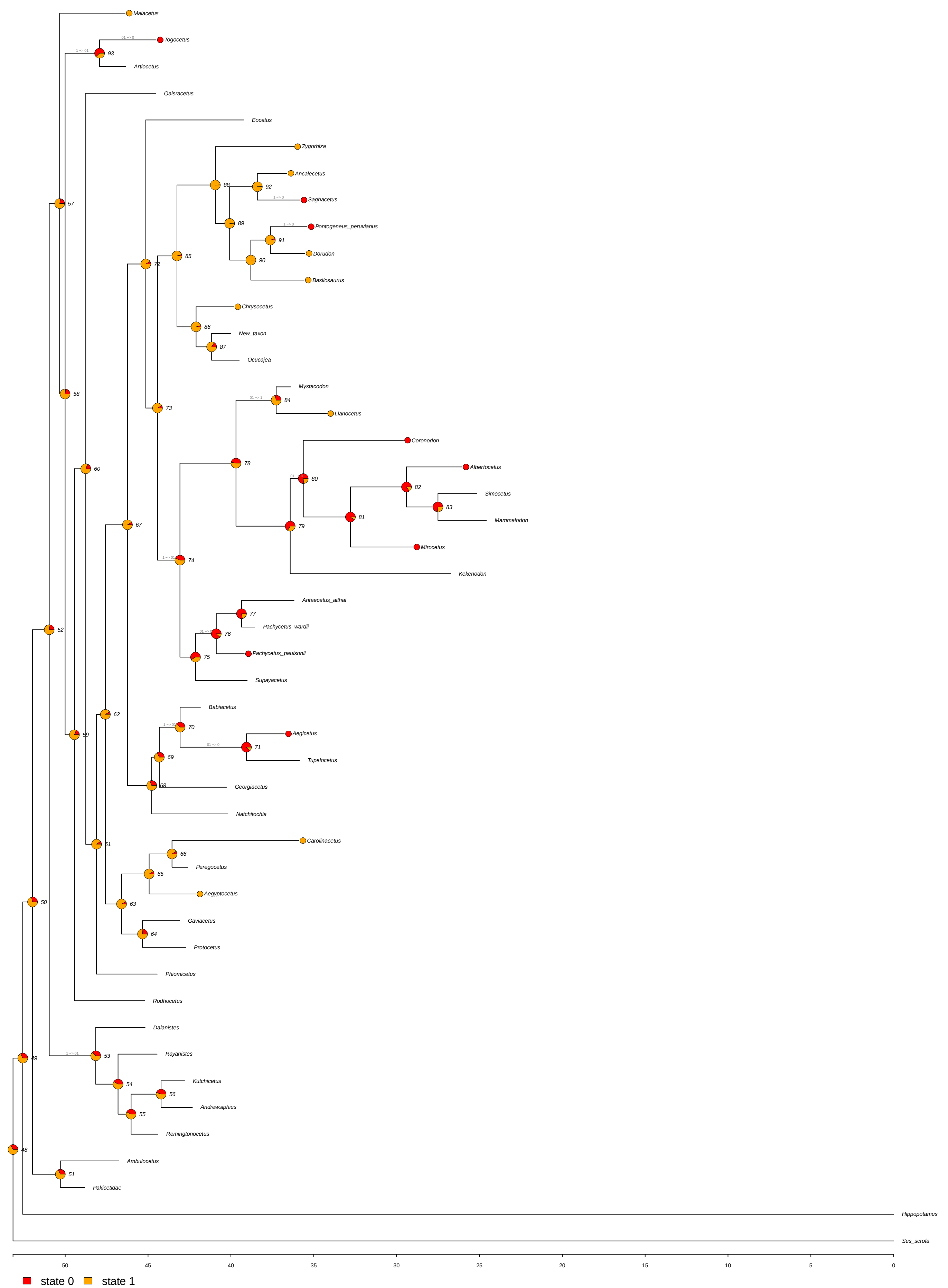

Maiacetus
01 -> 0
Togocetus
1 -> 01
93
Artiocetus
Qaisracetus
Eocetus
Zygorhiza
Ancalecetus
88
92
1 -> 0
Saghacetus
57
89
1 -> 0
Pontogeneus_peruvianus
91
Dorudon
72
85
90
Basilosaurus
Chrysocetus
86
New_taxon
87
Ocucajea
Mystacodon
01 -> 1
84
58
73
Llanocetus
Coronodon
Albertocetus
60
78
80
82
Simocetus
83
Mammalodon
67
81
79
Mirocetus
1 -> 01
74
Kekenodon
Antaecetus_aithai
77
01 -> 0
76
Pachycetus_wardii
52
75
Pachycetus_paulsonii
Supayacetus
Babiacetus
62
1 -> 01
70
Aegicetus
59
01 -> 0
71
Tupelocetus
69
68
Georgiacetus
Natchitochia
Carolinacetus
61
66
Peregocetus
65
Aegyptocetus
50
63
Gaviacetus
64
Protocetus
Phiomicetus
Rodhocetus
Dalanistes
1 -> 01
53
Rayanistes
49
54
Kutchicetus
56
55
Andrewsiphius
Remingtonocetus
48
Ambulocetus
51
Pakicetidae
Hippopotamus
Sus_scrofa
50
45
40
35
30
25
20
15
10
5
0
state 0
state 1

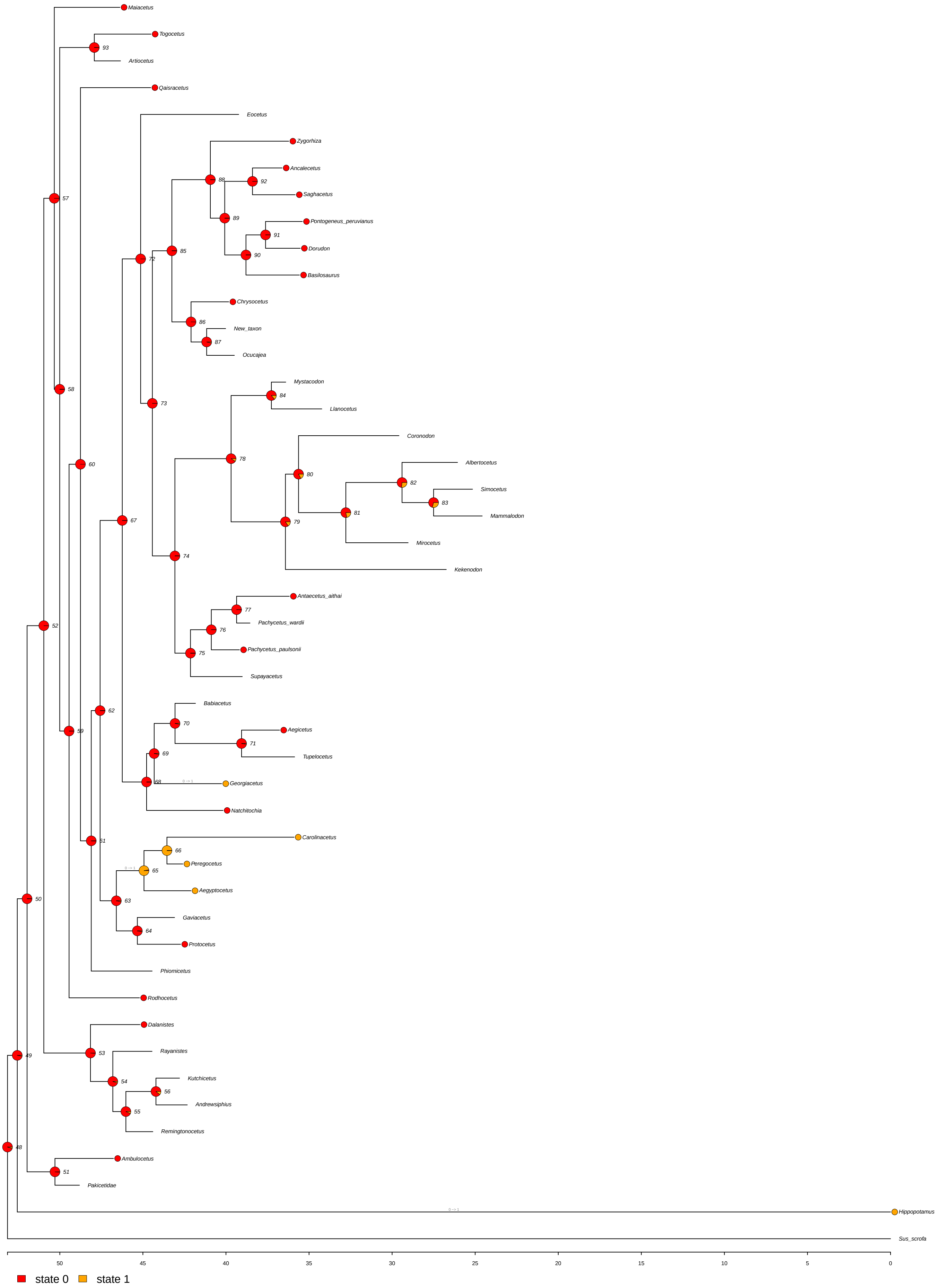
Maiacetus
Togocetus
93
Artiocetus
Qaisracetus
Eocetus
Zygorhiza
Ancalecetus
88
92
Saghacetus
89
Pontogeneus_peruvianus
91
Dorudon
90
Basilosaurus
57
72
85
Chrysocetus
86
New_taxon
87
Ocucajea
Mystacodon
84
Llanocetus
58
73
Coronodon
60
78
Albertocetus
80
82
Simocetus
83
Mammalodon
81
67
79
Mirocetus
74
Kekenodon
Antaecetus_aithai
77
Pachycetus_wardii
76
52
Pachycetus_paulsonii
75
Supayacetus
Babiacetus
62
70
Aegicetus
59
71
Tupelocetus
69
68
0 -> 1
Georgiacetus
Natchitochia
Carolinacetus
61
66
Peregocetus
0 -> 1
65
Aegyptocetus
50
63
Gaviacetus
64
Protocetus
Phiomicetus
Rodhocetus
Dalanistes
Rayanistes
53
49
54
Kutchicetus
56
Andrewsiphius
55
Remingtonocetus
48
Ambulocetus
51
Pakicetidae
0 -> 1
Hippopotamus
Sus_scrofa
50
45
40
35
30
25
20
15
10
5
0
state 0
state 1

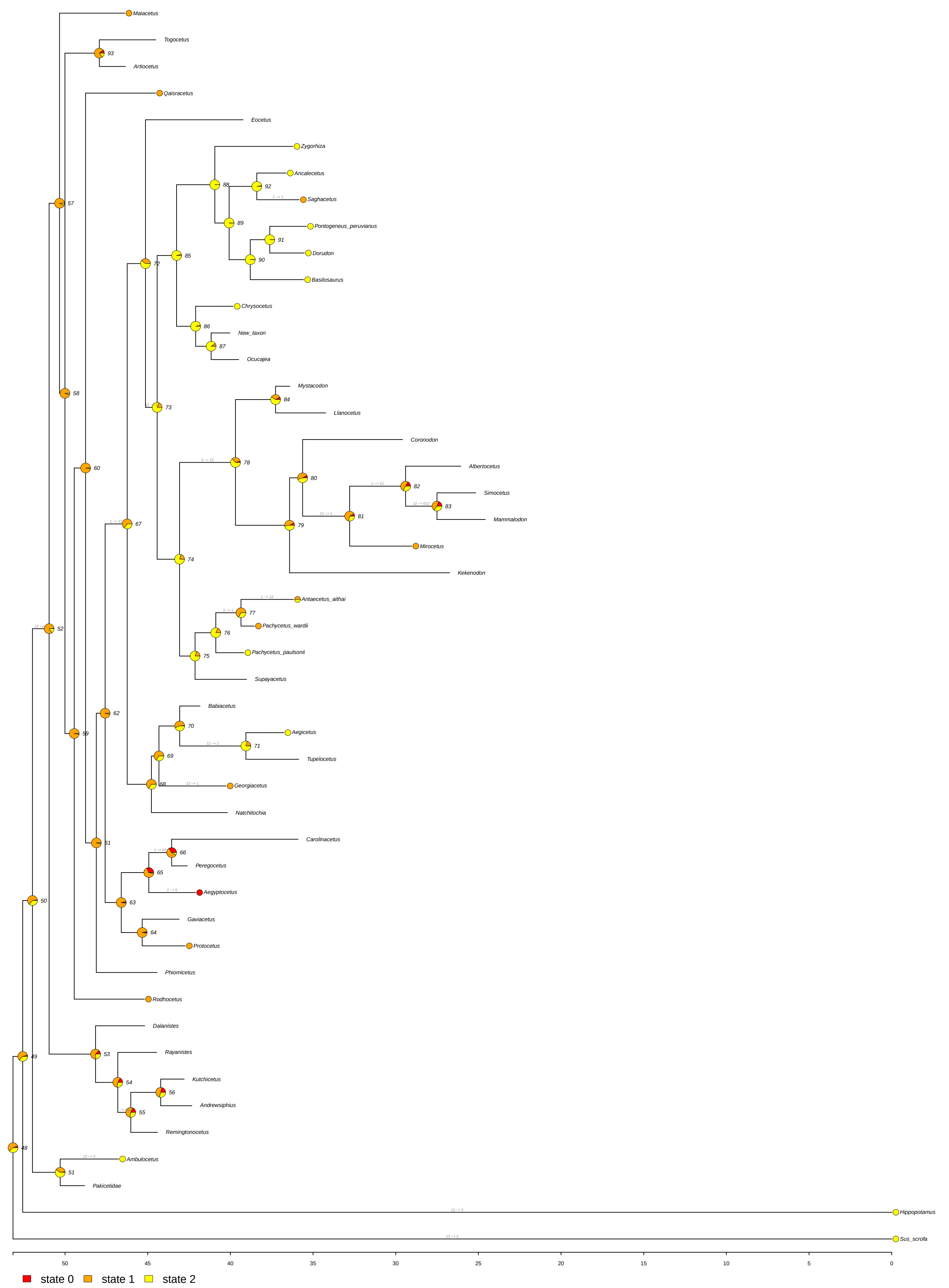
Maiacetus
Togocetus
93
Artiocetus
Qaisracetus
Eocetus
Zygorhiza
Ancalecetus
88
92
Saghacetus
Pontogeneus_peruvianus
89
91
Dorudon
90
Basilosaurus
85
72
Chrysocetus
86
New_taxon
87
Ocucajea
57
Mystacodon
84
Llanocetus
58
73
Coronodon
78
Albertocetus
80
82
Simocetus
83
81
Mammalodon
60
67
79
Mirocetus
74
Kekenodon
Antaecetus_aithai
77
Pachycetus_wardii
76
Pachycetus_paulsonii
52
75
Supayacetus
Babiacetus
62
70
Aegicetus
59
71
Tupelocetus
69
68
Georgiacetus
Natchitochia
Carolinacetus
66
61
Peregocetus
65
Aegyptocetus
50
63
Gaviacetus
64
Protocetus
Phiomicetus
Rodhocetus
Dalanistes
53
Rayanistes
49
54
Kutchicetus
56
Andrewsiphius
55
Remingtonocetus
48
Ambulocetus
51
Pakicetidae
Hippopotamus
Sus_scrofa
50
45
40
35
30
25
20
15
10
5
0
state 0
state 1
state 2

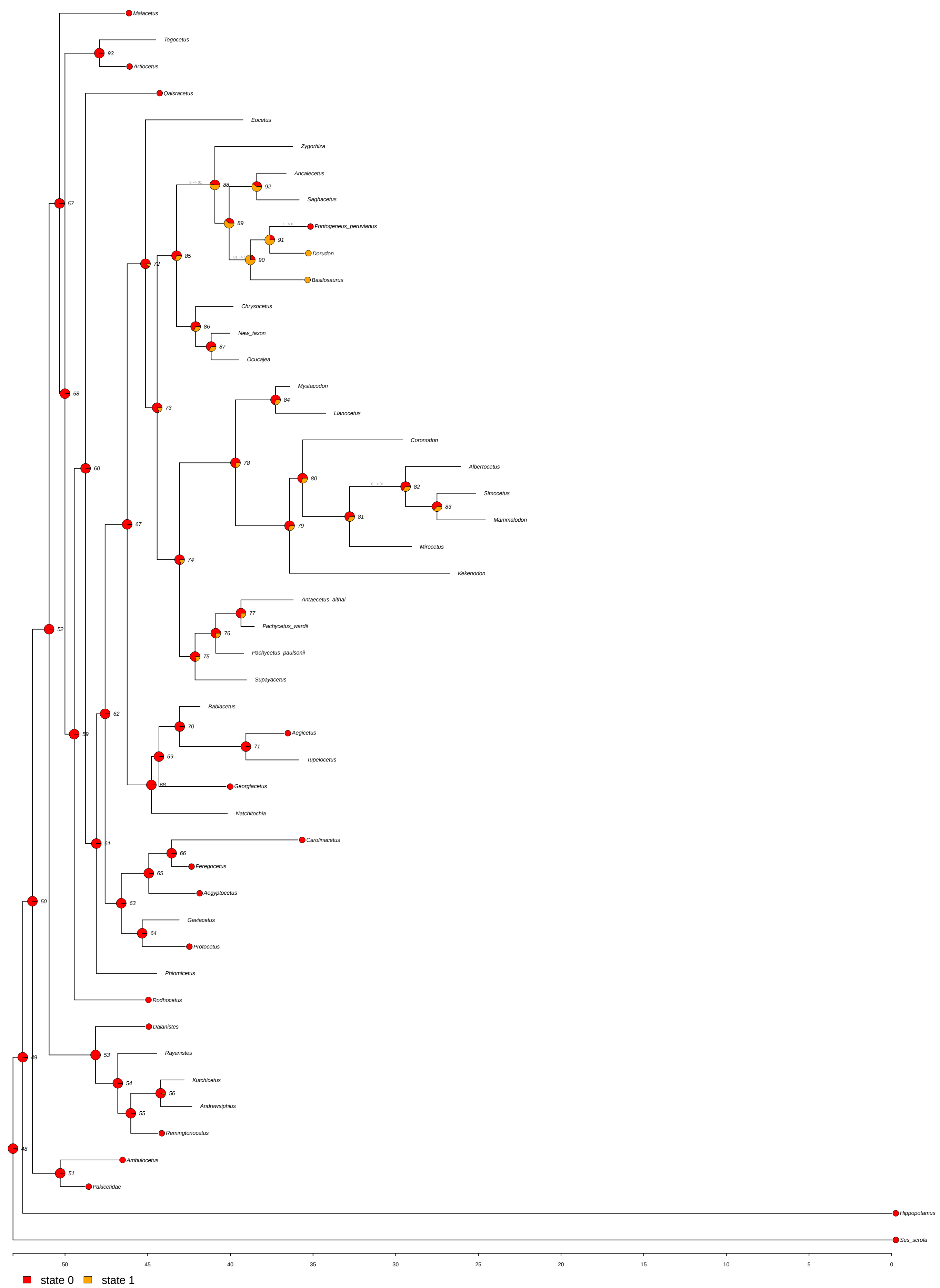

Maiacetus
Togocetus
Artiocetus
Qaisracetus
Eocetus
Zygorhiza
Ancalecetus
Saghacetus
Pontogeneus_peruvianus
Dorudon
Basilosaurus
Chrysocetus
New_taxon
Ocucajea
Mystacodon
Llanocetus
Coronodon
Albertocetus
Simocetus
Mammalodon
Mirocetus
Kekenodon
Antaecetus_aithai
Pachycetus_wardii
Pachycetus_paulsonii
Supayacetus
Babiacetus
Aegicetus
Tupelocetus
Georgiacetus
Natchitochia
Carolinacetus
Peregocetus
Aegyptocetus
Gaviacetus
Protocetus
Phiomicetus
Rodhocetus
Dalanistes
Rayanistes
Kutchicetus
Andrewsiphius
Remingtonocetus
Ambulocetus
Pakicetidae
Hippopotamus
Sus_scrofa
50
45
40
35
30
25
20
15
10
5
0
state 0
state 1

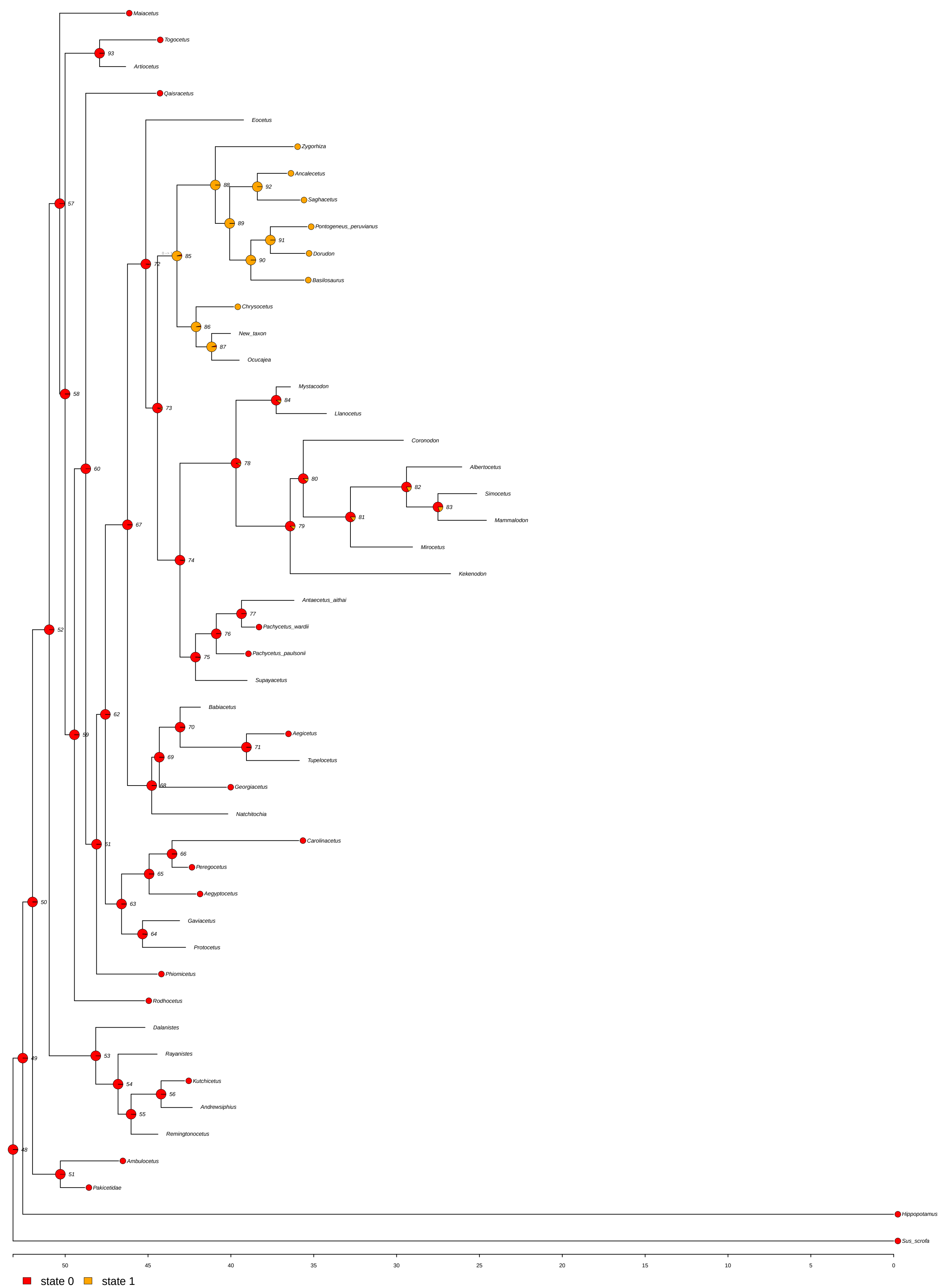
Maiacetus
Togocetus
93
Artiocetus
Qaisracetus
Eocetus
Zygorhiza
Ancalecetus
88
92
Saghacetus
Pontogeneus_peruvianus
89
91
Dorudon
90
Basilosaurus
85
72
57
Chrysocetus
86
New_taxon
87
Ocucajea
Mystacodon
84
Llanocetus
58
73
Coronodon
78
Albertocetus
80
82
Simocetus
83
81
Mammalodon
79
Mirocetus
60
67
74
Kekenodon
Antaecetus_aithai
77
Pachycetus_wardii
76
Pachycetus_paulsonii
75
52
Supayacetus
Babiacetus
62
70
Aegicetus
59
71
Tupelocetus
69
68
Georgiacetus
Natchitochia
Carolinacetus
61
66
Peregocetus
65
Aegyptocetus
50
63
Gaviacetus
64
Protocetus
Phiomicetus
Rodhocetus
Dalanistes
53
Rayanistes
49
54
Kutchicetus
56
55
Andrewsiphius
Remingtonocetus
48
Ambulocetus
51
Pakicetidae
Hippopotamus
Sus_scrofa
50
45
40
35
30
25
20
15
10
5
0
state 0
state 1

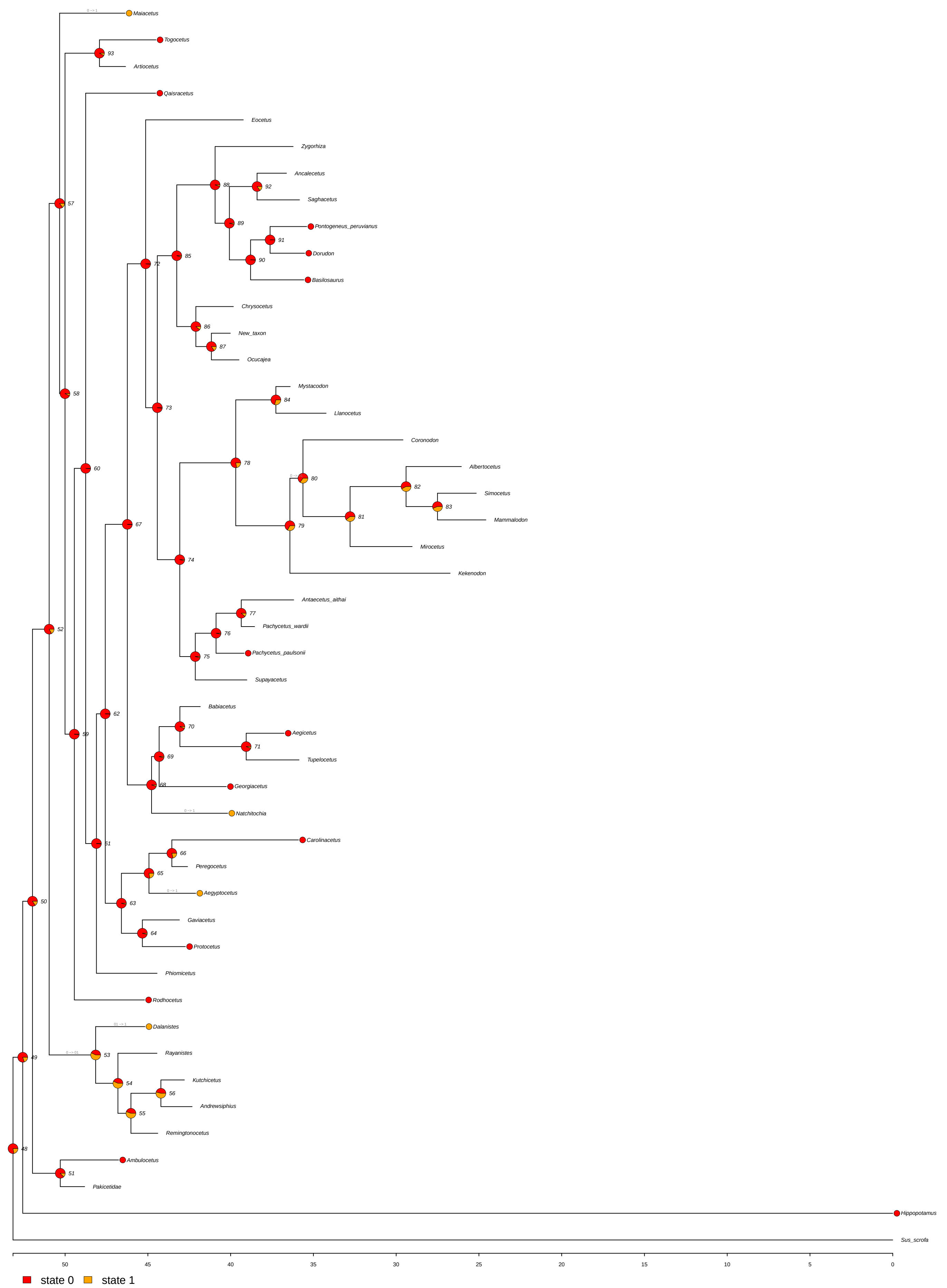

Maiacetus
Togocetus
Artiocetus
Qaisracetus
Eocetus
Zygorhiza
Ancalecetus
Saghacetus
Pontogeneus_peruvianus
Dorudon
Basilosaurus
Chrysocetus
New_taxon
Ocucajea
Mystacodon
Llanocetus
Coronodon
Albertocetus
Simocetus
Mammalodon
Mirocetus
Kekenodon
Antaecetus_aithai
Pachycetus_wardii
Pachycetus_paulsonii
Supayacetus
Babiacetus
Aegicetus
Tupelocetus
Georgiacetus
Natchitochia
Carolinacetus
Peregocetus
Aegyptocetus
Gaviacetus
Protocetus
Phiomicetus
Rodhocetus
Dalanistes
Rayanistes
Kutchicetus
Andrewsiphius
Remingtonocetus
Ambulocetus
Pakicetidae
Hippopotamus
Sus_scrofa
50 45 40 35 30 25 20 15 10 5 0
state 0
state 1

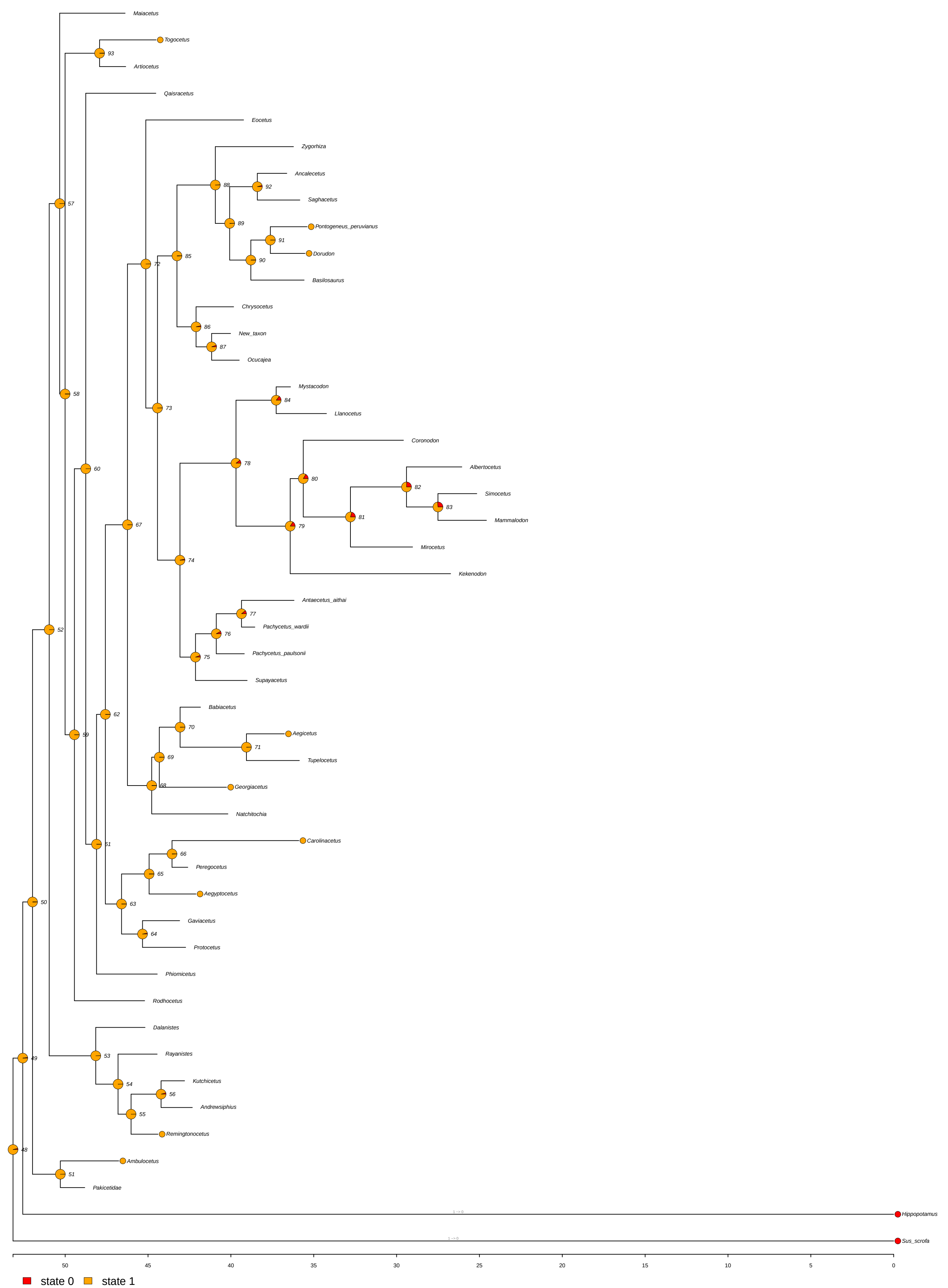

Maiacetus
Togocetus
93
Artiocetus
Qaisracetus
Eocetus
Zygorhiza
Ancalecetus
88
92
Saghacetus
Pontogeneus_peruvianus
89
91
Dorudon
90
Basilosaurus
57
72
85
Chrysocetus
86
New_taxon
87
Ocucajea
Mystacodon
84
58
Llanocetus
73
Coronodon
Albertocetus
78
80
60
82
Simocetus
83
Mammalodon
81
79
67
Mirocetus
74
Kekenodon
Antaecetus_aithai
77
Pachycetus_wardii
76
52
Pachycetus_paulsonii
75
Supayacetus
Babiacetus
62
59
70
Aegicetus
71
69
Tupelocetus
68
Georgiacetus
Natchitochia
Carolinacetus
61
66
Peregocetus
65
Aegyptocetus
50
63
Gaviacetus
64
Protocetus
Phiomicetus
Rodhocetus
Dalanistes
Rayanistes
53
49
54
Kutchicetus
56
Andrewsiphius
55
Remingtonocetus
48
Ambulocetus
51
Pakicetidae
1 -> 0
Hippopotamus
1 -> 0
Sus_scrofa
50
45
40
35
30
25
20
15
10
5
0
state 0
state 1

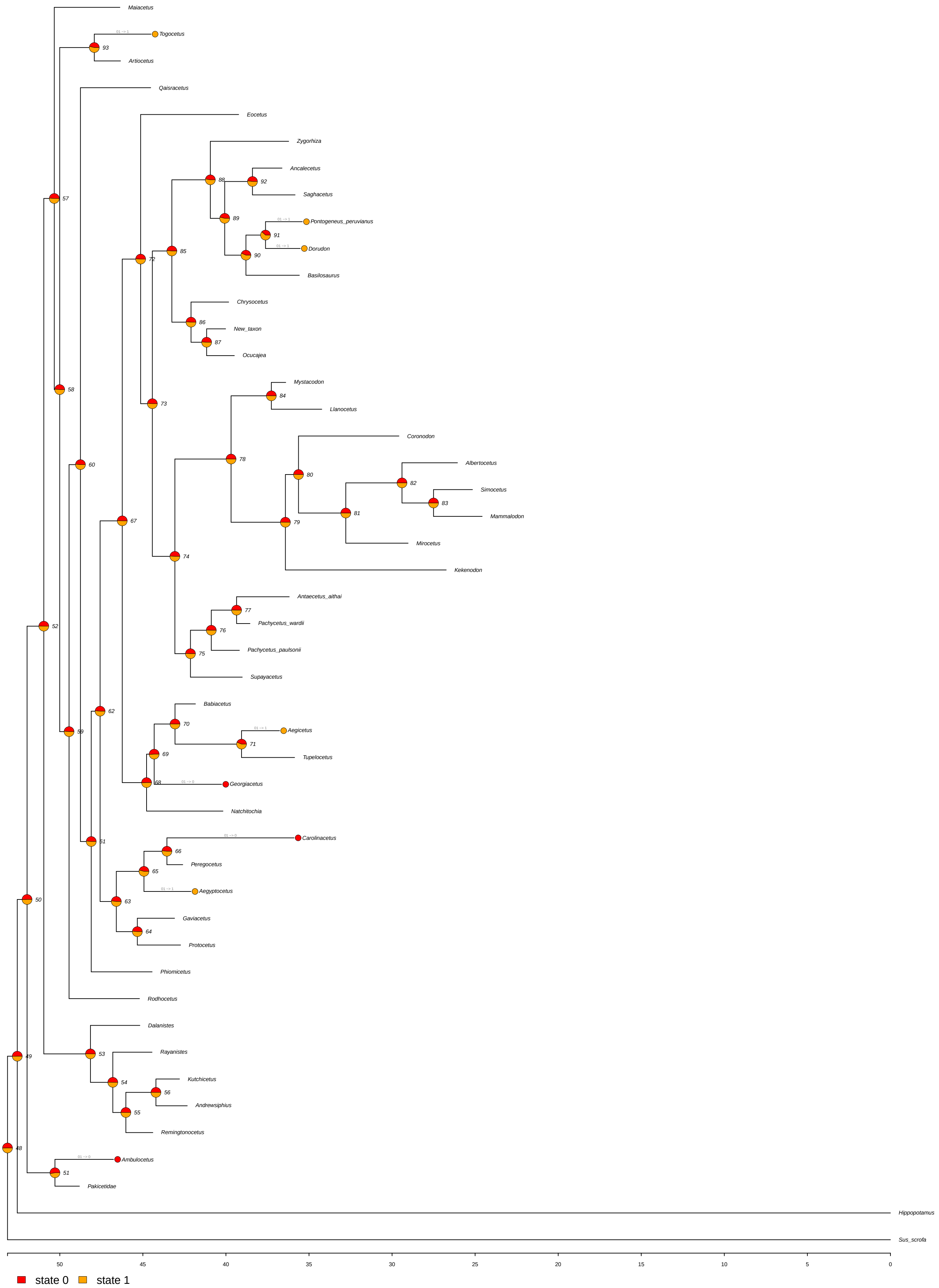

Maiacetus
Togocetus
Artiocetus
Qaisracetus
Eocetus
Zygorhiza
Ancalecetus
Saghacetus
Pontogeneus_peruvianus
Dorudon
Basilosaurus
Chrysocetus
New_taxon
Ocucajea
Mystacodon
Llanocetus
Coronodon
Albertocetus
Simocetus
Mammalodon
Mirocetus
Kekenodon
Antaecetus_aithai
Pachycetus_wardii
Pachycetus_paulsonii
Supayacetus
Babiacetus
Aegicetus
Tupelocetus
Georgiacetus
Natchitochia
Carolinacetus
Peregocetus
Aegyptocetus
Gaviacetus
Protocetus
Phiomicetus
Rodhocetus
Dalanistes
Rayanistes
Kutchicetus
Andrewsiphius
Remingtonocetus
Ambulocetus
Pakicetidae
Hippopotamus
Sus_scrofa
50
45
40
35
30
25
20
15
10
5
0
state 0
state 1

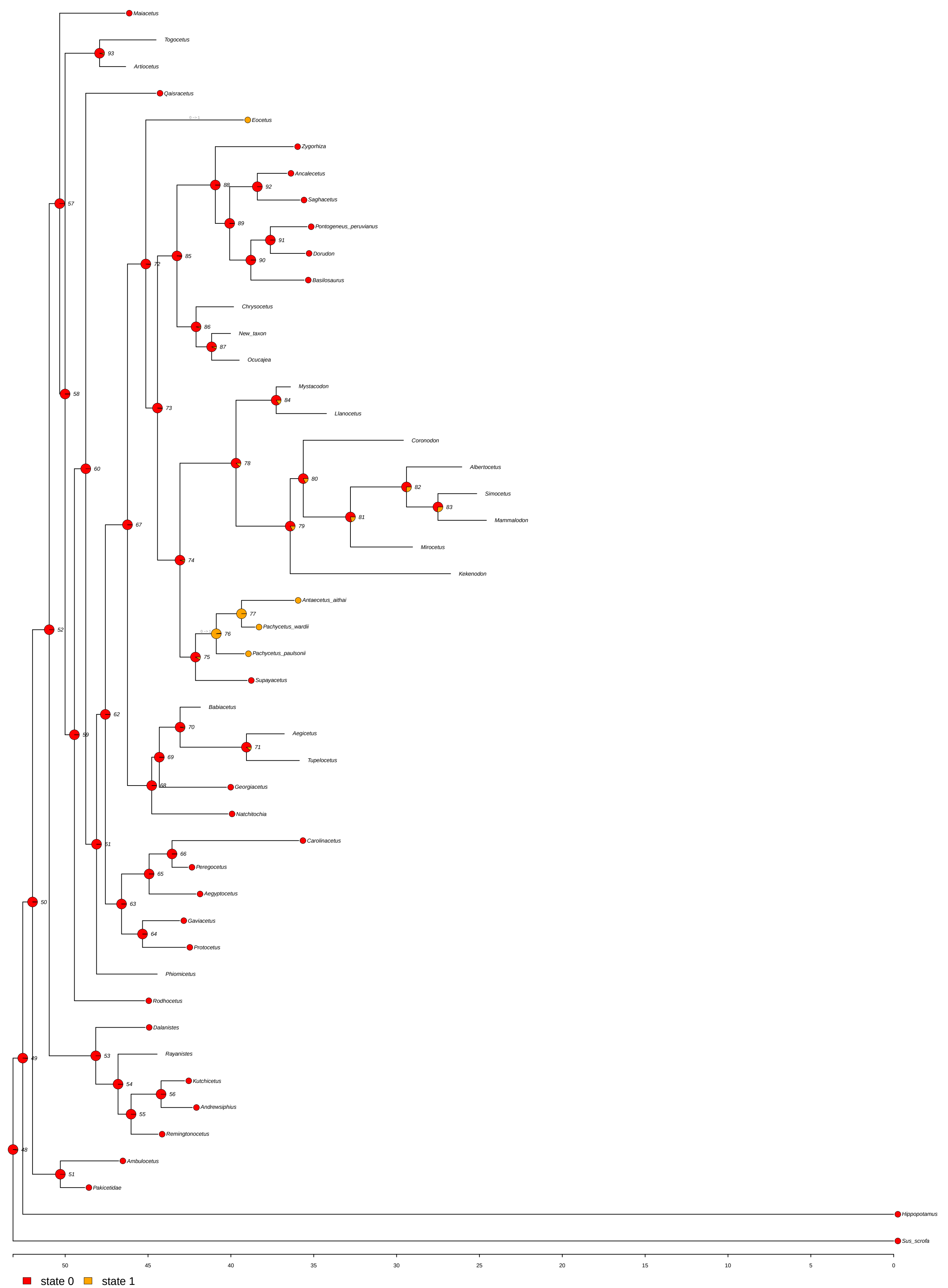
Maiacetus
Togocetus
Artiocetus
Qaisracetus
Eocetus
Zygorhiza
Ancalecetus
Saghacetus
Pontogeneus_peruvianus
Dorudon
Basilosaurus
Chrysocetus
New_taxon
Ocucajea
Mystacodon
Llanocetus
Coronodon
Albertocetus
Simocetus
Mammalodon
Mirocetus
Kekenodon
Antaecetus_aithai
Pachycetus_wardii
Pachycetus_paulsonii
Supayacetus
Babiacetus
Aegicetus
Tupelocetus
Georgiacetus
Natchitochia
Carolinacetus
Peregocetus
Aegyptocetus
Gaviacetus
Protocetus
Phiomicetus
Rodhocetus
Dalanistes
Rayanistes
Kutchicetus
Andrewsiphius
Remingtonocetus
Ambulocetus
Pakicetidae
Hippopotamus
Sus_scrofa
50
45
40
35
30
25
20
15
10
5
0
state 0
state 1

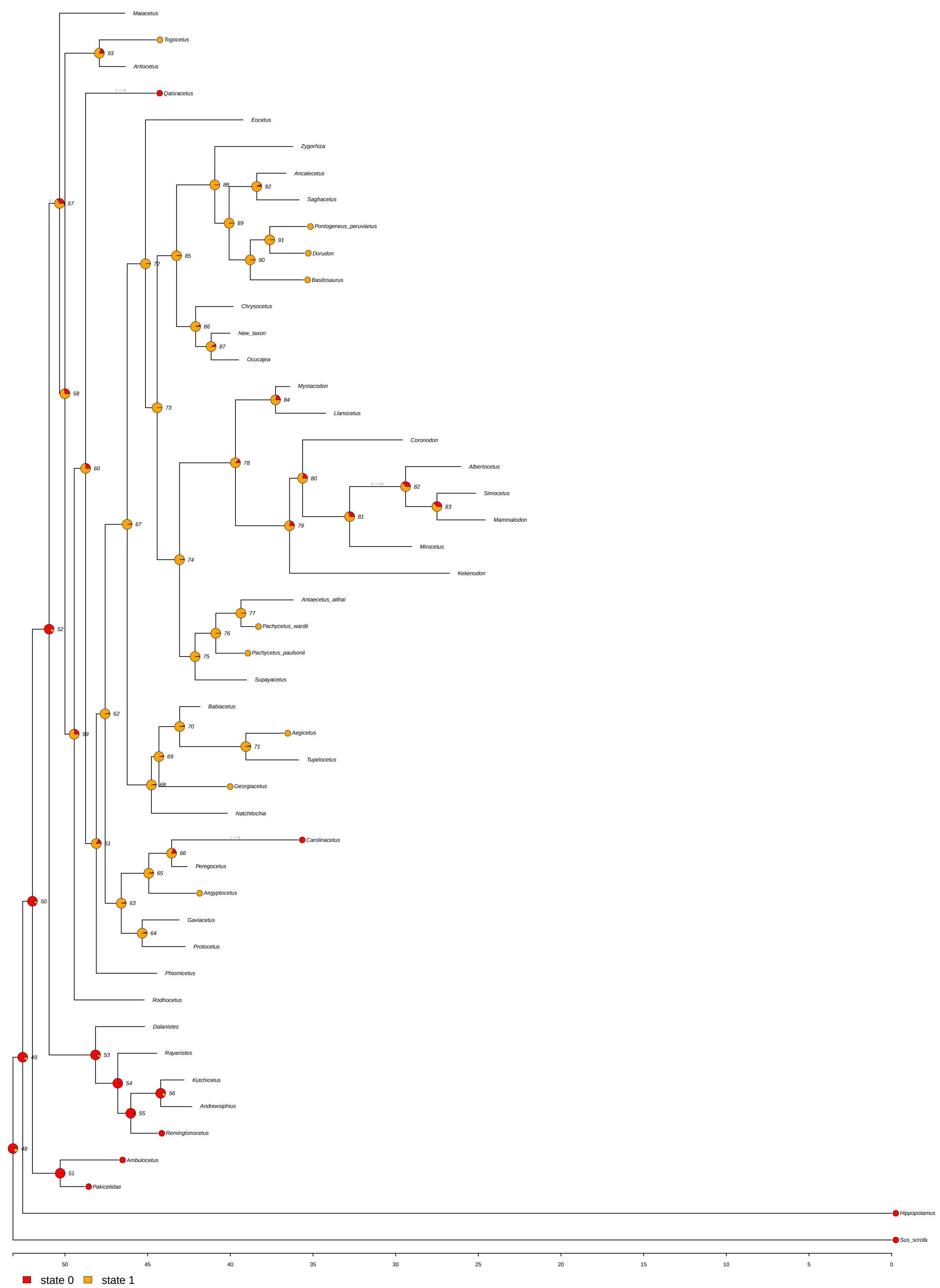

Maiacetus
Togocetus
93
Artiocetus
Qaisracetus
Eocetus
Zygorhiza
Ancalecetus
88
92
Saghacetus
57
89
Pontogeneus_peruvianus
91
Dorudon
90
72
85
Basilosaurus
Chrysocetus
86
New_taxon
87
Ocucajea
Mystacodon
84
58
Llanocetus
73
Coronodon
60
78
Albertocetus
80
82
Simocetus
83
81
Mammalodon
67
79
Mirocetus
74
Kekenodon
Antaecetus_aithai
77
Pachycetus_wardii
52
76
Pachycetus_paulsonii
75
Supayacetus
Babiacetus
62
70
Aegicetus
59
71
69
Tupelocetus
68
Georgiacetus
Natchitochia
Carolinacetus
61
66
Peregocetus
65
Aegyptocetus
50
63
Gaviacetus
64
Protocetus
Phiomicetus
Rodhocetus
Dalanistes
53
Rayanistes
49
54
Kutchicetus
56
Andrewsiphius
55
Remingtonocetus
48
Ambulocetus
51
Pakicetidae
Hippopotamus
Sus_scrofa
50
45
40
35
30
25
20
15
10
5
0
state 0
state 1

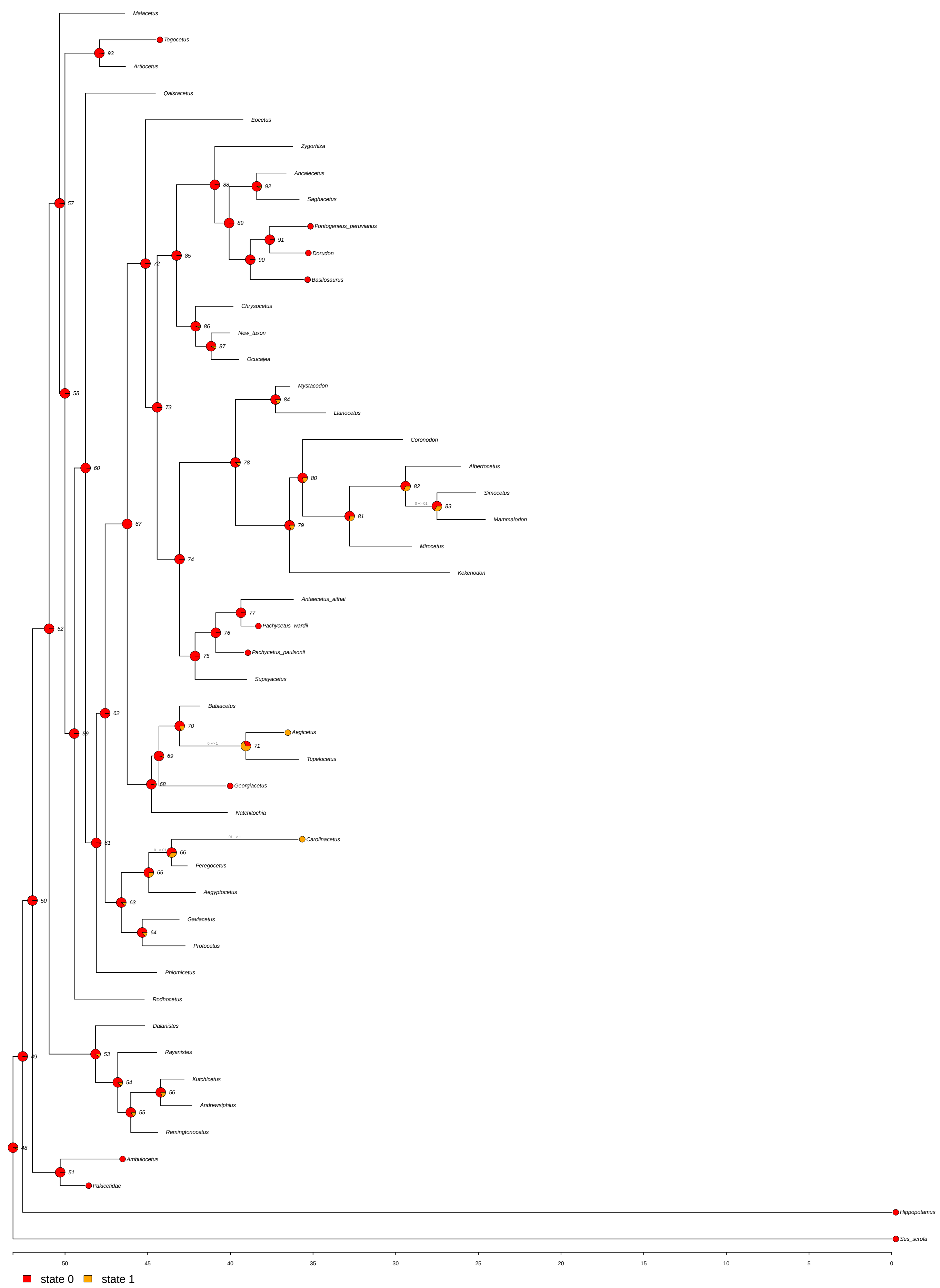

Maiacetus
Togocetus
Artiocetus
Qaisracetus
Eocetus
Zygorhiza
Ancalecetus
Saghacetus
Pontogeneus_peruvianus
Dorudon
Basilosaurus
Chrysocetus
New_taxon
Ocucajea
Mystacodon
Llanocetus
Coronodon
Albertocetus
Simocetus
Mammalodon
Mirocetus
Kekenodon
Antaecetus_aithai
Pachycetus_wardii
Pachycetus_paulsonii
Supayacetus
Babiacetus
Aegicetus
Tupelocetus
Georgiacetus
Natchitochia
Carolinacetus
Peregocetus
Aegyptocetus
Gaviacetus
Protocetus
Phiomicetus
Rodhocetus
Dalanistes
Rayanistes
Kutchicetus
Andrewsiphius
Remingtonocetus
Ambulocetus
Pakicetidae
Hippopotamus
Sus_scrofa
50
45
40
35
30
25
20
15
10
5
0
state 0
state 1

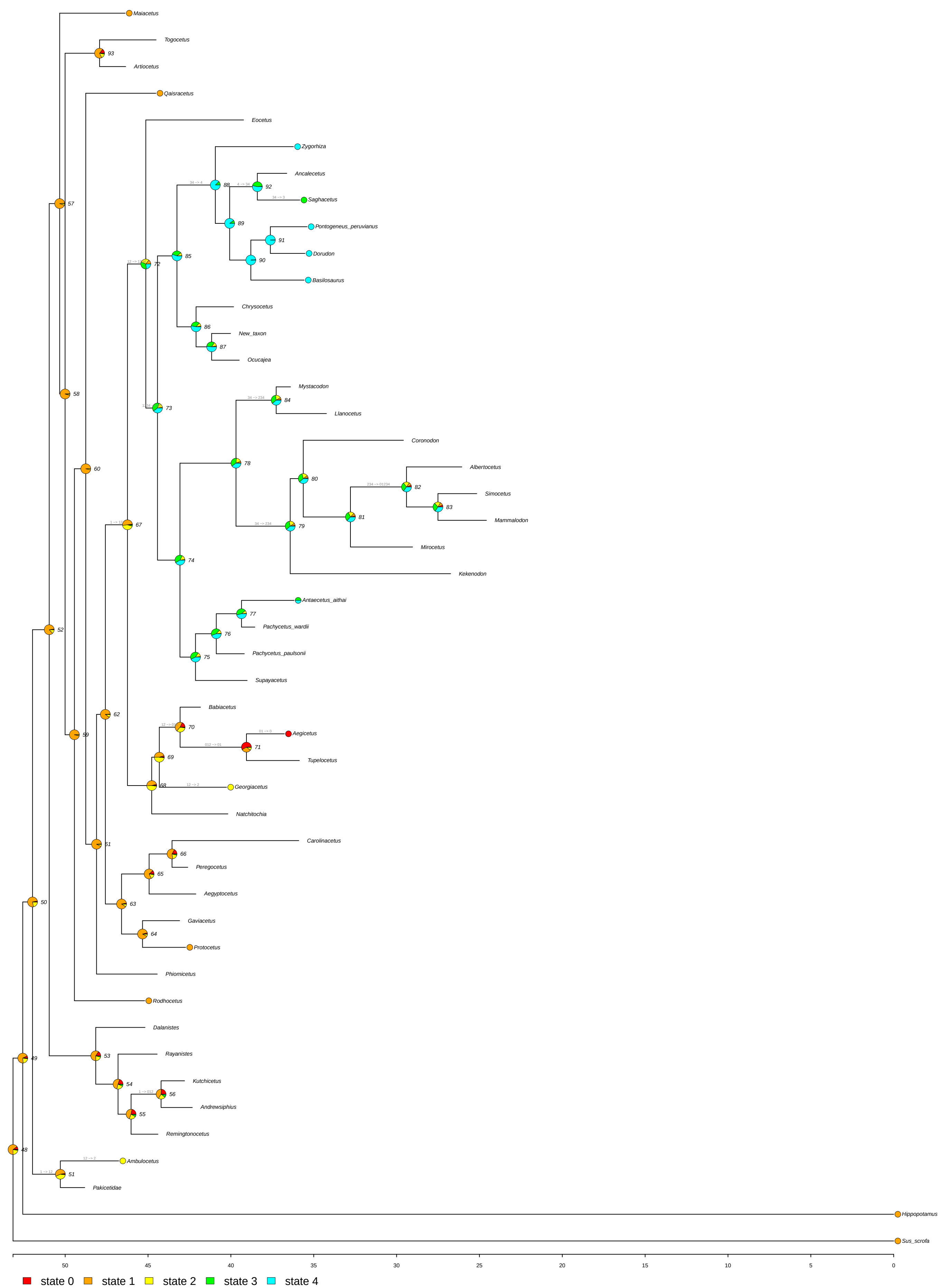
Maiacetus
Togocetus
Artiocetus
Qaisracetus
Eocetus
Zygorhiza
Ancalecetus
Saghacetus
Pontogeneus_peruvianus
Dorudon
Basilosaurus
Chrysocetus
New_taxon
Ocucajea
Mystacodon
Llanocetus
Coronodon
Albertocetus
Simocetus
Mammalodon
Mirocetus
Kekenodon
Antaecetus_aithai
Pachycetus_wardii
Pachycetus_paulsonii
Supayacetus
Babiacetus
Aegicetus
Tupelocetus
Georgiacetus
Natchitochia
Carolinacetus
Peregocetus
Aegyptocetus
Gaviacetus
Protocetus
Phiomicetus
Rodhocetus
Dalanistes
Rayanistes
Kutchicetus
Andrewsiphius
Remingtonocetus
Ambulocetus
Pakicetidae
Hippopotamus
Sus_scrofa
50
45
40
35
30
25
20
15
10
5
0
state 0
state 1
state 2
state 3
state 4

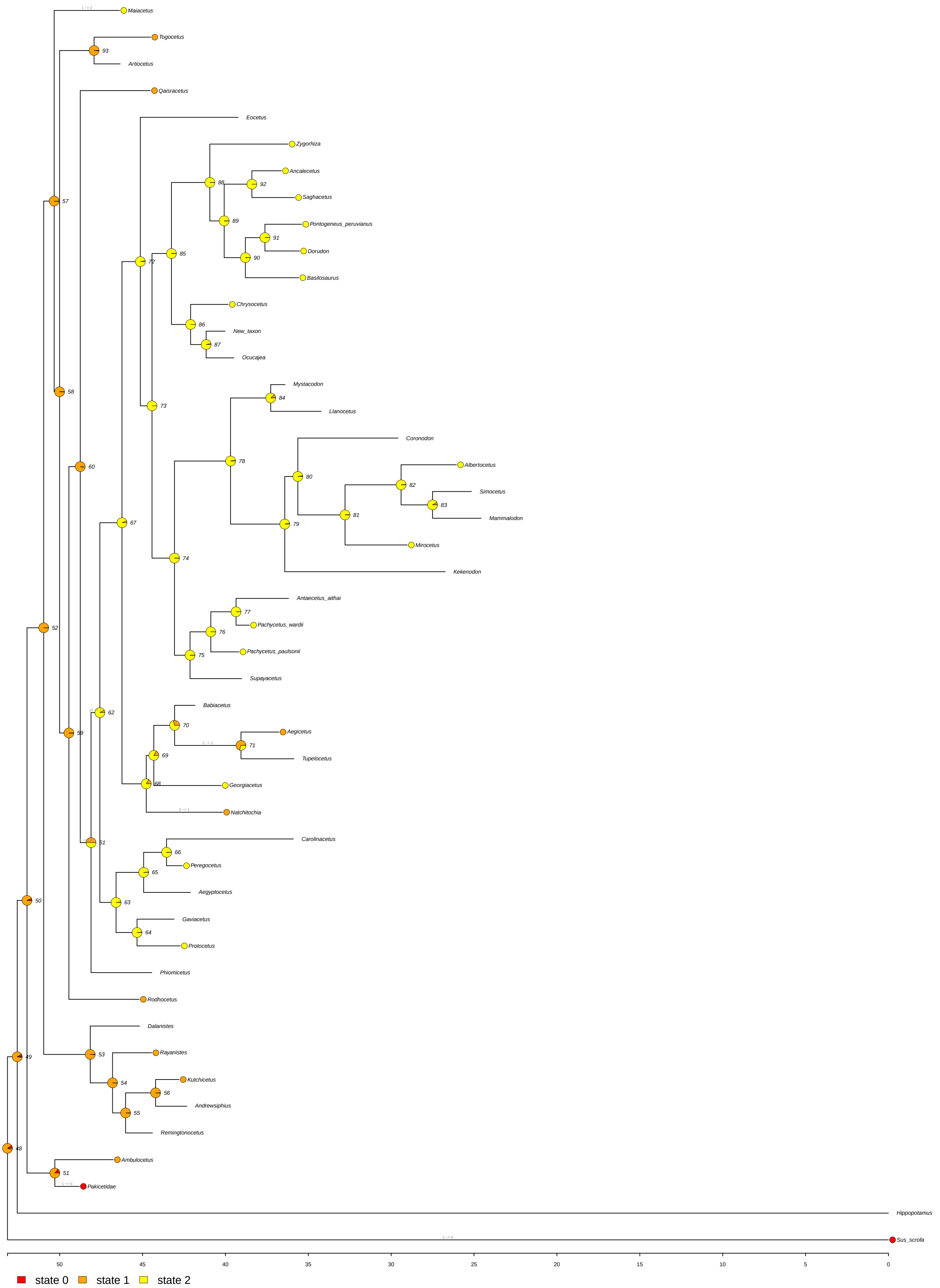
Maiacetus
Togocetus
Artiocetus
Qaisracetus
Eocetus
Zygorhiza
Ancalecetus
Saghacetus
Pontogeneus_peruvianus
Dorudon
Basilosaurus
Chrysocetus
New_taxon
Ocucajea
Mystacodon
Llanocetus
Coronodon
Albertocetus
Simocetus
Mammalodon
Mirocetus
Kekenodon
Antaecetus_aithai
Pachycetus_wardii
Pachycetus_paulsonii
Supayacetus
Babiacetus
Aegicetus
Tupelocetus
Georgiacetus
Natchitochia
Carolinacetus
Peregocetus
Aegyptocetus
Gaviacetus
Protocetus
Phiomicetus
Rodhocetus
Dalanistes
Rayanistes
Kutchicetus
Andrewsiphius
Remingtonocetus
Ambulocetus
Pakicetidae
Hippopotamus
Sus_scrofa
50
45
40
35
30
25
20
15
10
5
0
state 0
state 1
state 2

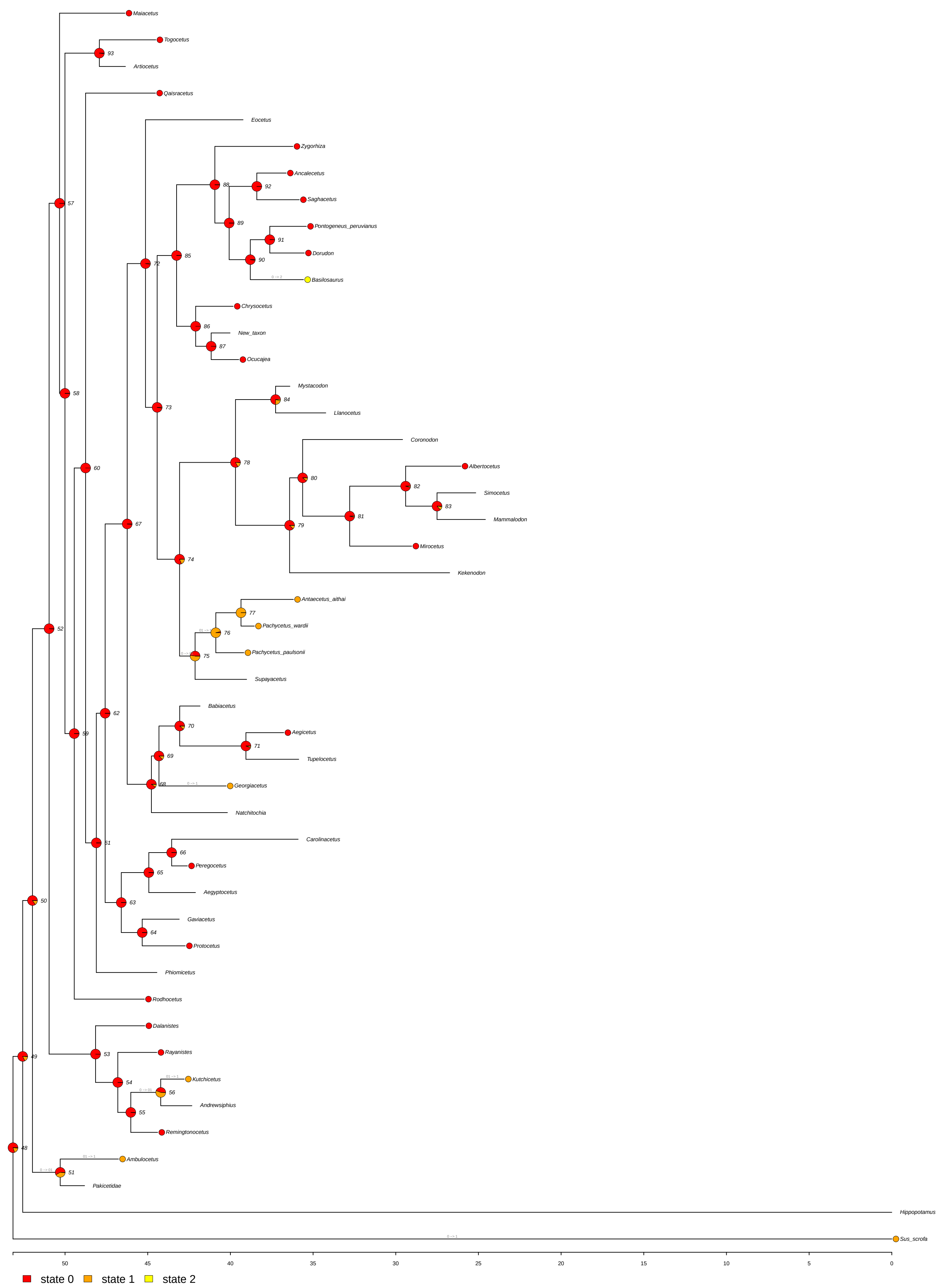
Maiacetus
Togocetus
93
Artiocetus
Qaisracetus
Eocetus
Zygorhiza
Ancalecetus
88
92
Saghacetus
89
Pontogeneus_peruvianus
91
Dorudon
90
0 -> 2
Basilosaurus
57
Chrysocetus
86
New_taxon
87
Ocucajea
72
85
Mystacodon
84
Llanocetus
58
73
Coronodon
Albertocetus
78
80
82
Simocetus
83
Mammalodon
60
81
79
Mirocetus
67
74
Kekenodon
Antaecetus_aithai
77
Pachycetus_wardii
52
76
Pachycetus_paulsonii
75
Supayacetus
Babiacetus
62
70
Aegicetus
59
71
Tupelocetus
69
68
0 -> 1
Georgiacetus
Natchitochia
Carolinacetus
61
66
Peregocetus
65
Aegyptocetus
50
63
Gaviacetus
64
Protocetus
Phiomicetus
Rodhocetus
Dalanistes
53
Rayanistes
49
54
01 -> 1
Kutchicetus
0 -> 01
56
Andrewsiphius
55
Remingtonocetus
48
01 -> 1
Ambulocetus
0 -> 01
51
Pakicetidae
Hippopotamus
0 -> 1
Sus_scrofa
50
45
40
35
30
25
20
15
10
5
0
state 0
state 1
state 2

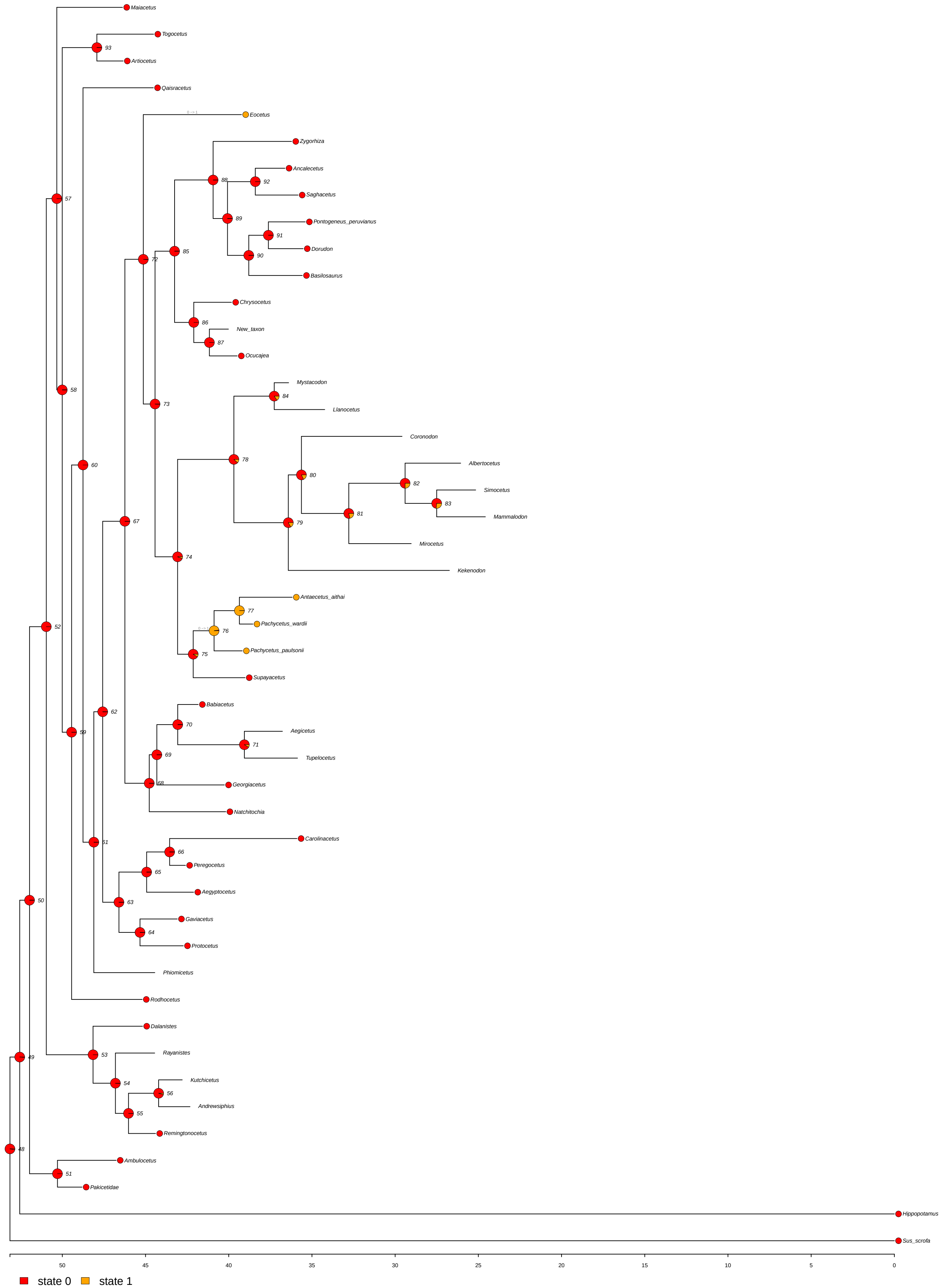

Maiacetus
Togocetus
93
Artiocetus
Qaisracetus
0 -> 1
Eocetus
Zygorhiza
Ancalecetus
88
92
Saghacetus
89
Pontogeneus_peruvianus
91
Dorudon
90
Basilosaurus
57
72
85
Chrysocetus
86
New_taxon
87
Ocucajea
Mystacodon
84
Llanocetus
58
73
Coronodon
78
Albertocetus
60
80
82
Simocetus
83
Mammalodon
81
67
79
Mirocetus
74
Kekenodon
Antaecetus_aithai
77
0 -> 1
76
Pachycetus_wardii
52
Pachycetus_paulsonii
75
Supayacetus
Babiacetus
62
70
Aegicetus
59
71
Tupelocetus
69
68
Georgiacetus
Natchitochia
Carolinacetus
66
Peregocetus
65
61
Aegyptocetus
50
63
Gaviacetus
64
Protocetus
Phiomicetus
Rodhocetus
Dalanistes
53
Rayanistes
49
54
Kutchicetus
56
55
Andrewsiphius
Remingtonocetus
48
Ambulocetus
51
Pakicetidae
Hippopotamus
Sus_scrofa
50
45
40
35
30
25
20
15
10
5
0
state 0
state 1

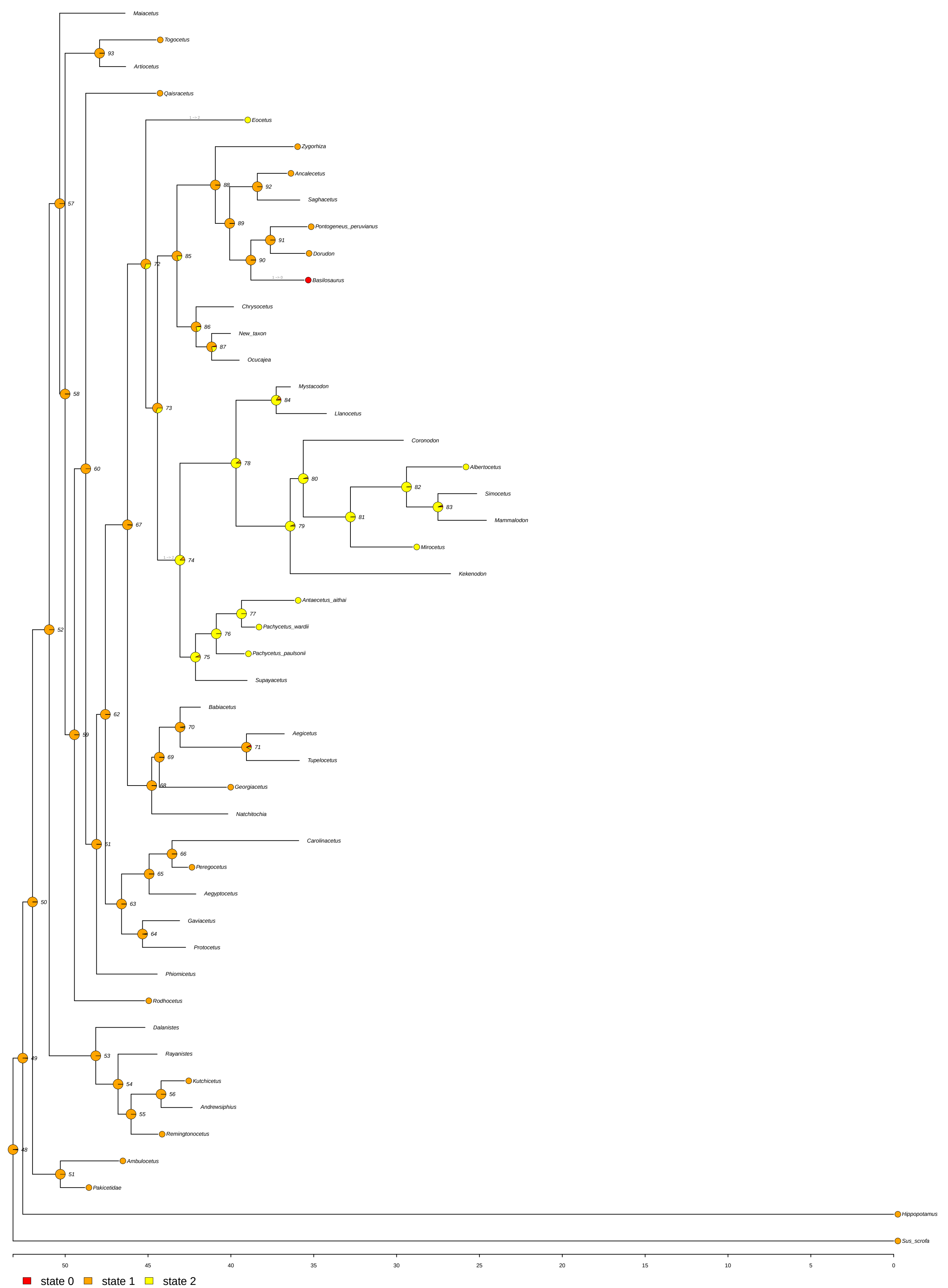
Maiacetus
Togocetus
Artiocetus
Qaisracetus
Eocetus
Zygorhiza
Ancalecetus
Saghacetus
Pontogeneus_peruvianus
Dorudon
Basilosaurus
Chrysocetus
New_taxon
Ocucajea
Mystacodon
Llanocetus
Coronodon
Albertocetus
Simocetus
Mammalodon
Mirocetus
Kekenodon
Antaecetus_aithai
Pachycetus_wardii
Pachycetus_paulsonii
Supayacetus
Babiacetus
Aegicetus
Tupelocetus
Georgiacetus
Natchitochia
Carolinacetus
Peregocetus
Aegyptocetus
Gaviacetus
Protocetus
Phiomicetus
Rodhocetus
Dalanistes
Rayanistes
Kutchicetus
Andrewsiphius
Remingtonocetus
Ambulocetus
Pakicetidae
Hippopotamus
Sus_scrofa
1 -> 2
1 -> 0
50
45
40
35
30
25
20
15
10
5
0
state 0
state 1
state 2

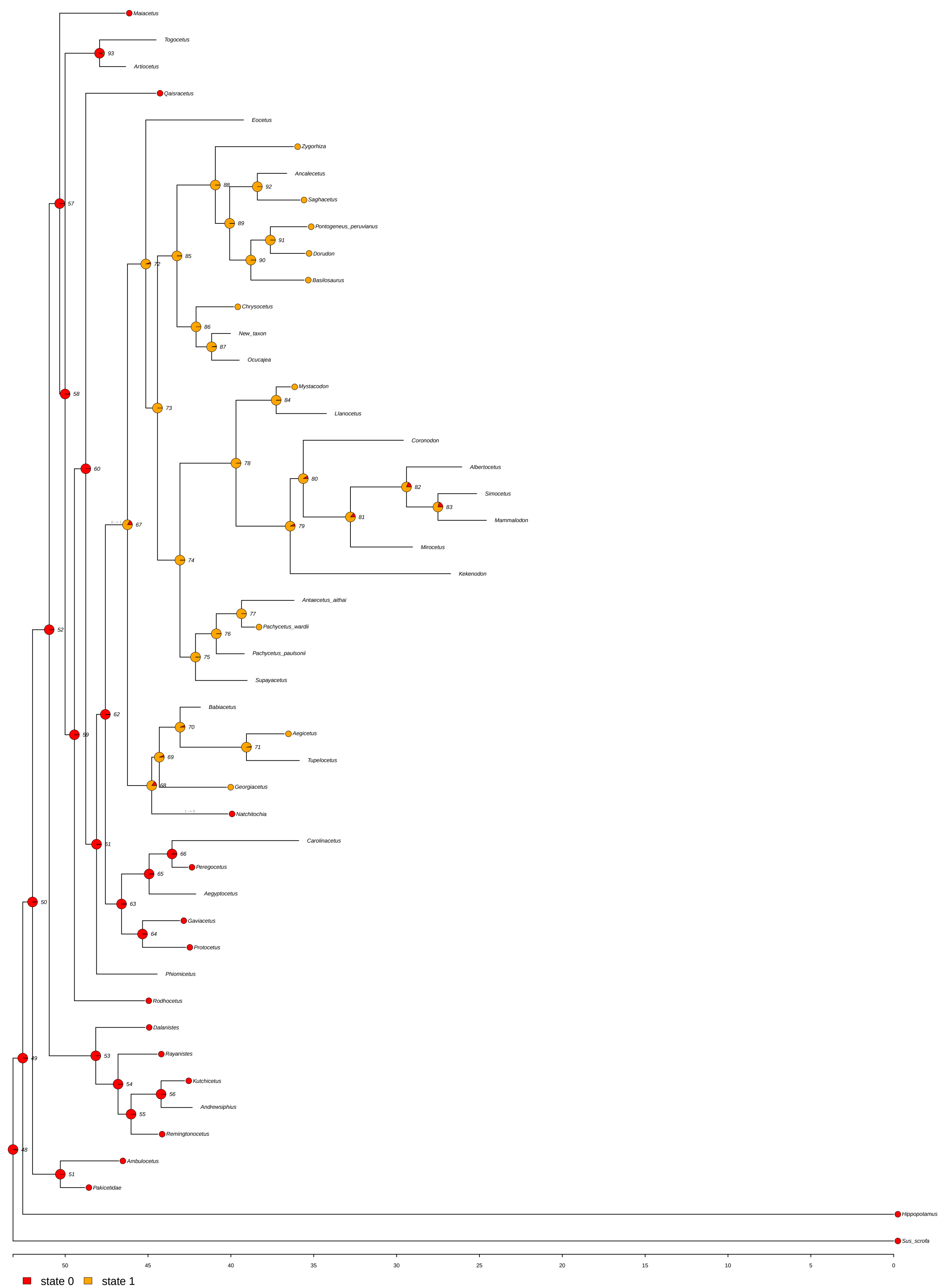

Maiacetus
Togocetus
93
Artiocetus
Qaisracetus
Eocetus
Zygorhiza
Ancalecetus
88
92
Saghacetus
57
Pontogeneus_peruvianus
89
91
Dorudon
72
85
90
Basilosaurus
Chrysocetus
86
New_taxon
87
Ocucajea
Mystacodon
84
58
73
Llanocetus
Coronodon
60
78
Albertocetus
80
82
Simocetus
83
81
Mammalodon
0 --> 1
67
79
Mirocetus
74
Kekenodon
Antaecetus_aithai
77
Pachycetus_wardii
76
52
Pachycetus_paulsonii
75
Supayacetus
Babiacetus
62
70
Aegicetus
59
71
Tupelocetus
69
68
Georgiacetus
1 --> 0
Natchitochia
61
Carolinacetus
66
Peregocetus
65
Aegyptocetus
50
63
Gaviacetus
64
Protocetus
Phiomicetus
Rodhocetus
Dalanistes
Rayanistes
53
49
Kutchicetus
54
56
Andrewsiphius
55
Remingtonocetus
48
Ambulocetus
51
Pakicetidae
Hippopotamus
Sus_scrofa
50
45
40
35
30
25
20
15
10
5
0
state 0
state 1

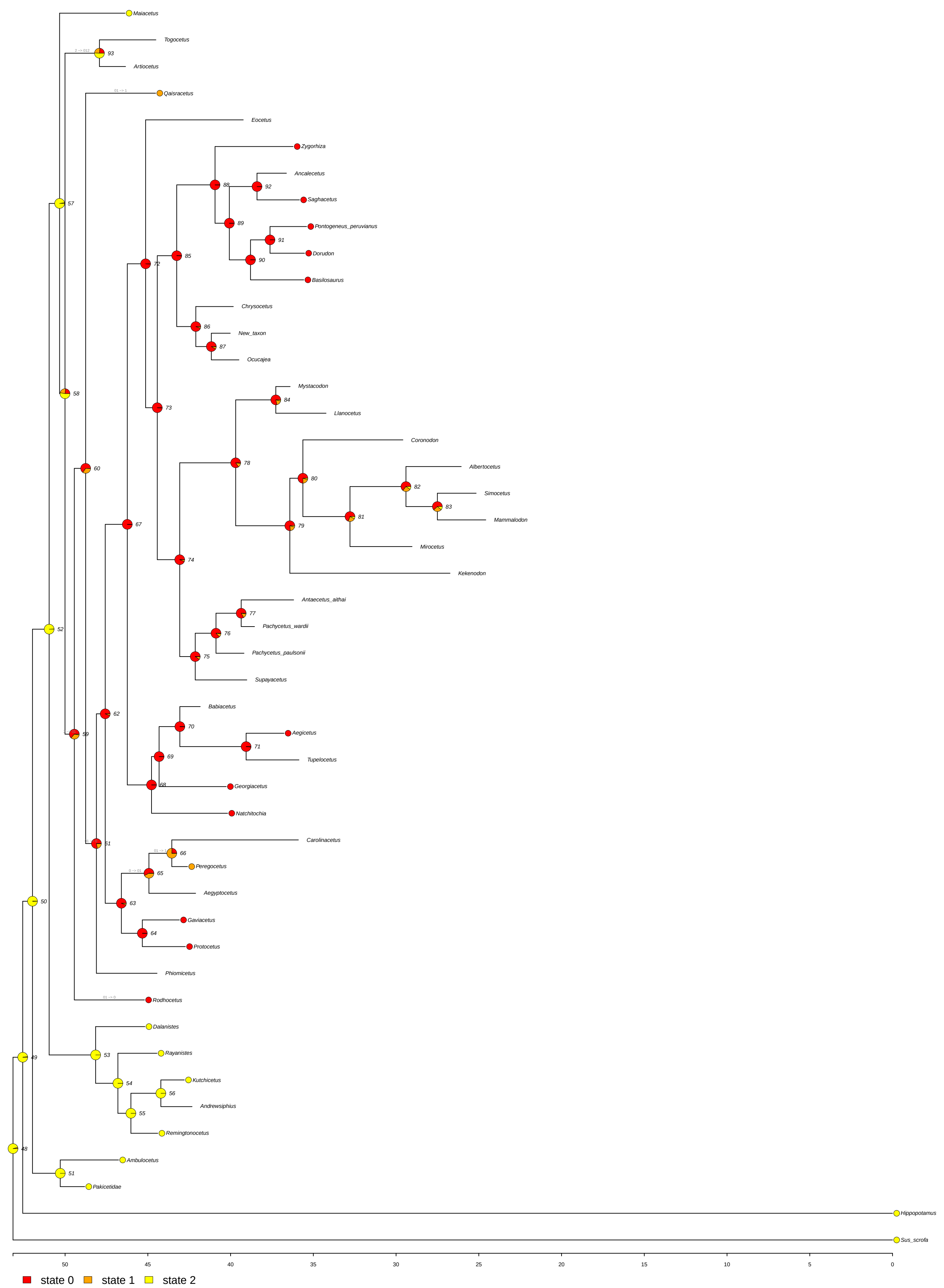

Maiacetus
Togocetus
2 -> 012
93
Artiocetus
01 -> 1
Qaisracetus
Eocetus
Zygorhiza
Ancalecetus
88
92
Saghacetus
89
Pontogeneus_peruvianus
91
Dorudon
90
Basilosaurus
57
72
85
Chrysocetus
86
New_taxon
87
Ocucajea
Mystacodon
84
58
Llanocetus
73
Coronodon
60
78
Albertocetus
80
82
Simocetus
83
81
Mammalodon
67
79
Mirocetus
74
Kekenodon
Antaecetus_aithai
77
Pachycetus_wardii
76
Pachycetus_paulsonii
52
75
Supayacetus
Babiacetus
62
70
59
Aegicetus
71
69
Tupelocetus
68
Georgiacetus
Natchitochia
Carolinacetus
61
01 -> 1
66
Peregocetus
0 -> 01
65
Aegyptocetus
50
63
Gaviacetus
64
Protocetus
Phiomicetus
01 -> 0
Rodhocetus
Dalanistes
53
Rayanistes
49
54
Kutchicetus
56
Andrewsiphius
55
Remingtonocetus
48
Ambulocetus
51
Pakicetidae
Hippopotamus
Sus_scrofa
50
45
40
35
30
25
20
15
10
5
0
state 0
state 1
state 2

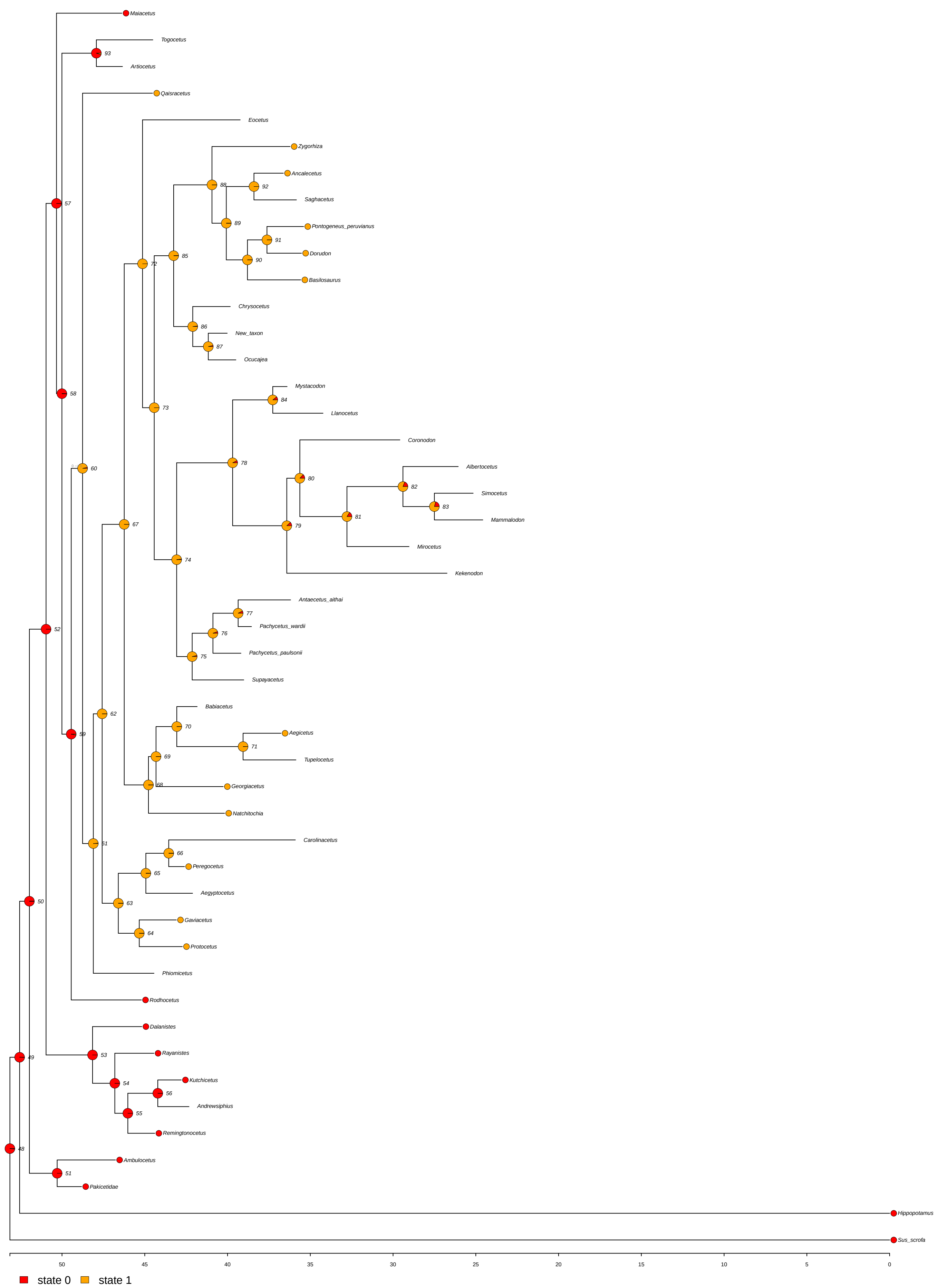

Maiacetus
Togocetus
93
Artiocetus
Qaisracetus
Eocetus
Zygorhiza
Ancalecetus
88
92
Saghacetus
Pontogeneus_peruvianus
89
91
Dorudon
90
Basilosaurus
72
85
57
Chrysocetus
86
New_taxon
87
Ocucajea
Mystacodon
84
Llanocetus
58
73
Coronodon
Albertocetus
60
78
80
82
Simocetus
83
Mammalodon
67
81
79
Mirocetus
74
Kekenodon
Antaecetus_aithai
77
Pachycetus_wardii
52
76
Pachycetus_paulsonii
75
Supayacetus
Babiacetus
62
70
Aegicetus
59
71
Tupelocetus
69
68
Georgiacetus
Natchitochia
61
Carolinacetus
66
Peregocetus
65
Aegyptocetus
50
63
Gaviacetus
64
Protocetus
Phiomicetus
Rodhocetus
Dalanistes
53
Rayanistes
49
54
Kutchicetus
56
Andrewsiphius
55
Remingtonocetus
48
Ambulocetus
51
Pakicetidae
Hippopotamus
Sus_scrofa
50
45
40
35
30
25
20
15
10
5
0
state 0
state 1

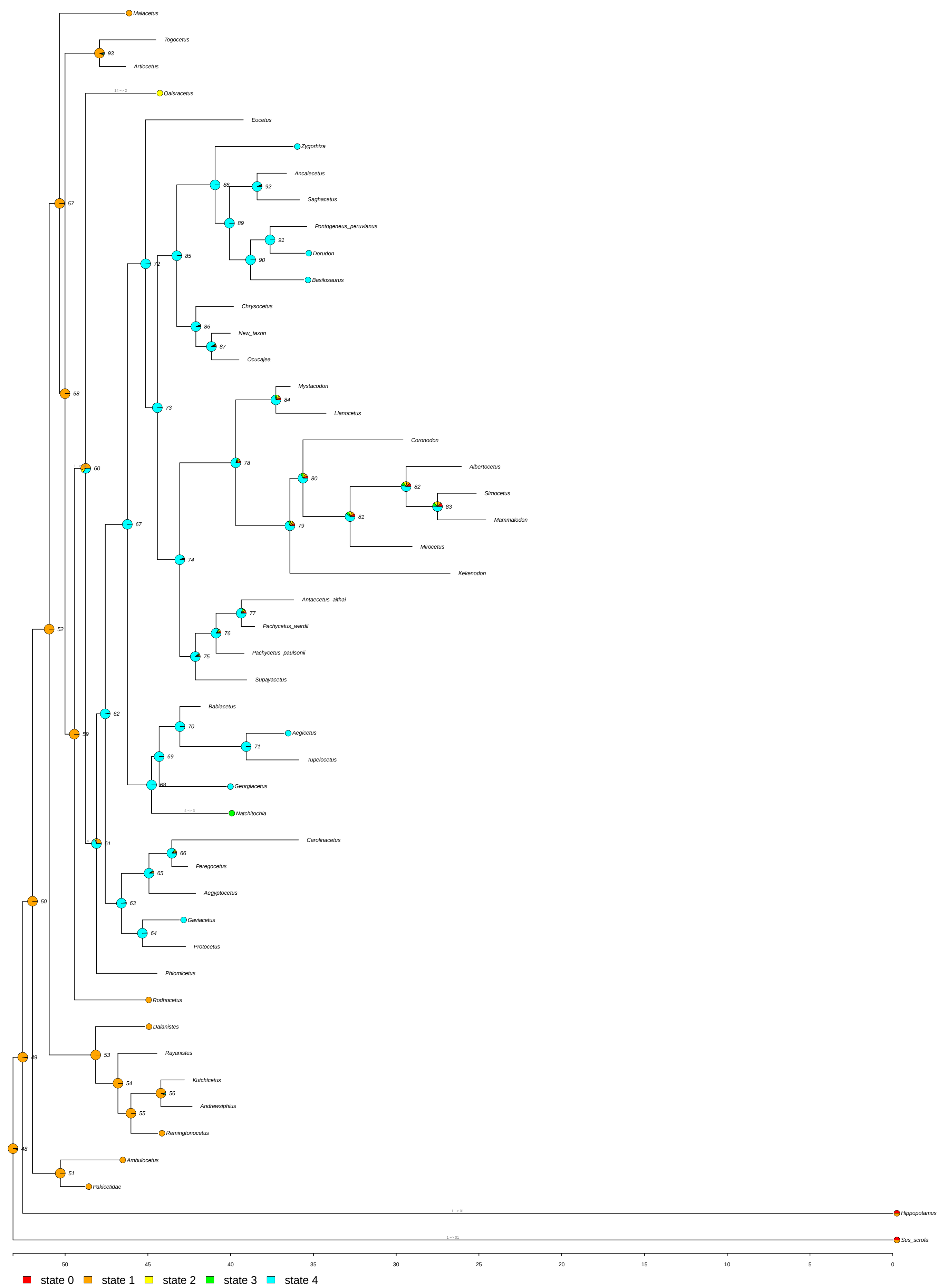
Maiacetus
Togocetus
Artiocetus
Qaisracetus
Eocetus
Zygorhiza
Ancalecetus
Saghacetus
Pontogeneus_peruvianus
Dorudon
Basilosaurus
Chrysocetus
New_taxon
Ocucajea
Mystacodon
Llanocetus
Coronodon
Albertocetus
Simocetus
Mammalodon
Mirocetus
Kekenodon
Antaecetus_aithai
Pachycetus_wardii
Pachycetus_paulsonii
Supayacetus
Babiacetus
Aegicetus
Tupelocetus
Georgiacetus
Natchitochia
Carolinacetus
Peregocetus
Aegyptocetus
Gaviacetus
Protocetus
Phiomicetus
Rodhocetus
Dalanistes
Rayanistes
Kutchicetus
Andrewsiphius
Remingtonocetus
Ambulocetus
Pakicetidae
Hippopotamus
Sus_scrofa
50
45
40
35
30
25
20
15
10
5
0
state 0
state 1
state 2
state 3
state 4

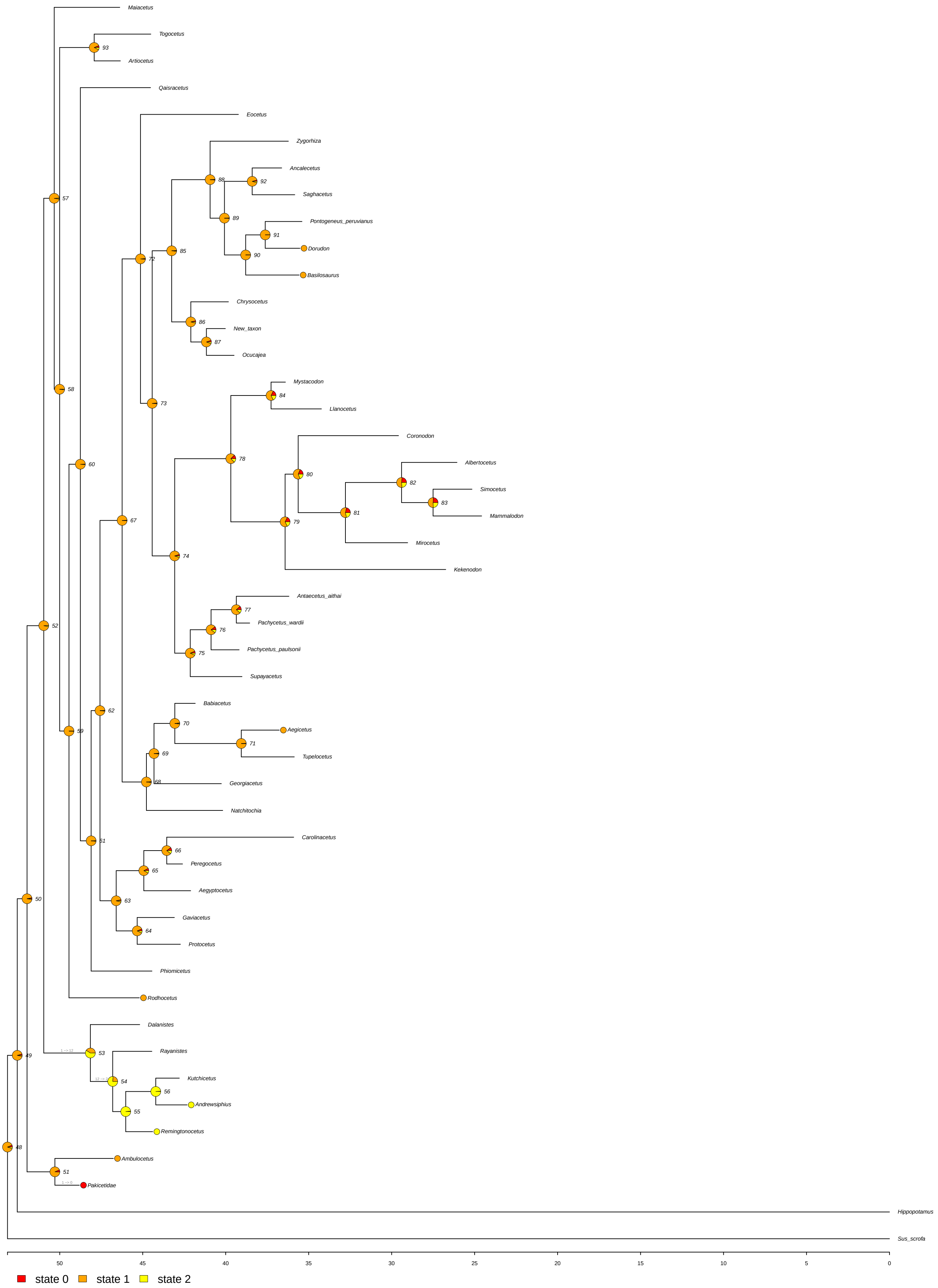

Maiacetus
Togocetus
93
Artiocetus
Qaisracetus
Eocetus
Zygorhiza
Ancalecetus
88
92
Saghacetus
Pontogeneus_peruvianus
89
91
Dorudon
72
85
90
Basilosaurus
Chrysocetus
86
New_taxon
87
Ocucajea
Mystacodon
84
58
Llanocetus
73
Coronodon
Albertocetus
60
78
80
82
Simocetus
83
67
81
Mammalodon
79
Mirocetus
74
Kekenodon
Antaecetus_aithai
77
Pachycetus_wardii
52
76
Pachycetus_paulsonii
75
Supayacetus
Babiacetus
62
70
Aegicetus
59
71
Tupelocetus
69
68
Georgiacetus
Natchitochia
Carolinacetus
61
66
Peregocetus
65
Aegyptocetus
50
63
Gaviacetus
64
Protocetus
Phiomicetus
Rodhocetus
Dalanistes
1 -> 12
53
Rayanistes
49
54
Kutchicetus
56
Andrewsiphius
55
Remingtonocetus
48
Ambulocetus
51
1 -> 0
Pakicetidae
Hippopotamus
Sus_scrofa
50
45
40
35
30
25
20
15
10
5
0
state 0
state 1
state 2

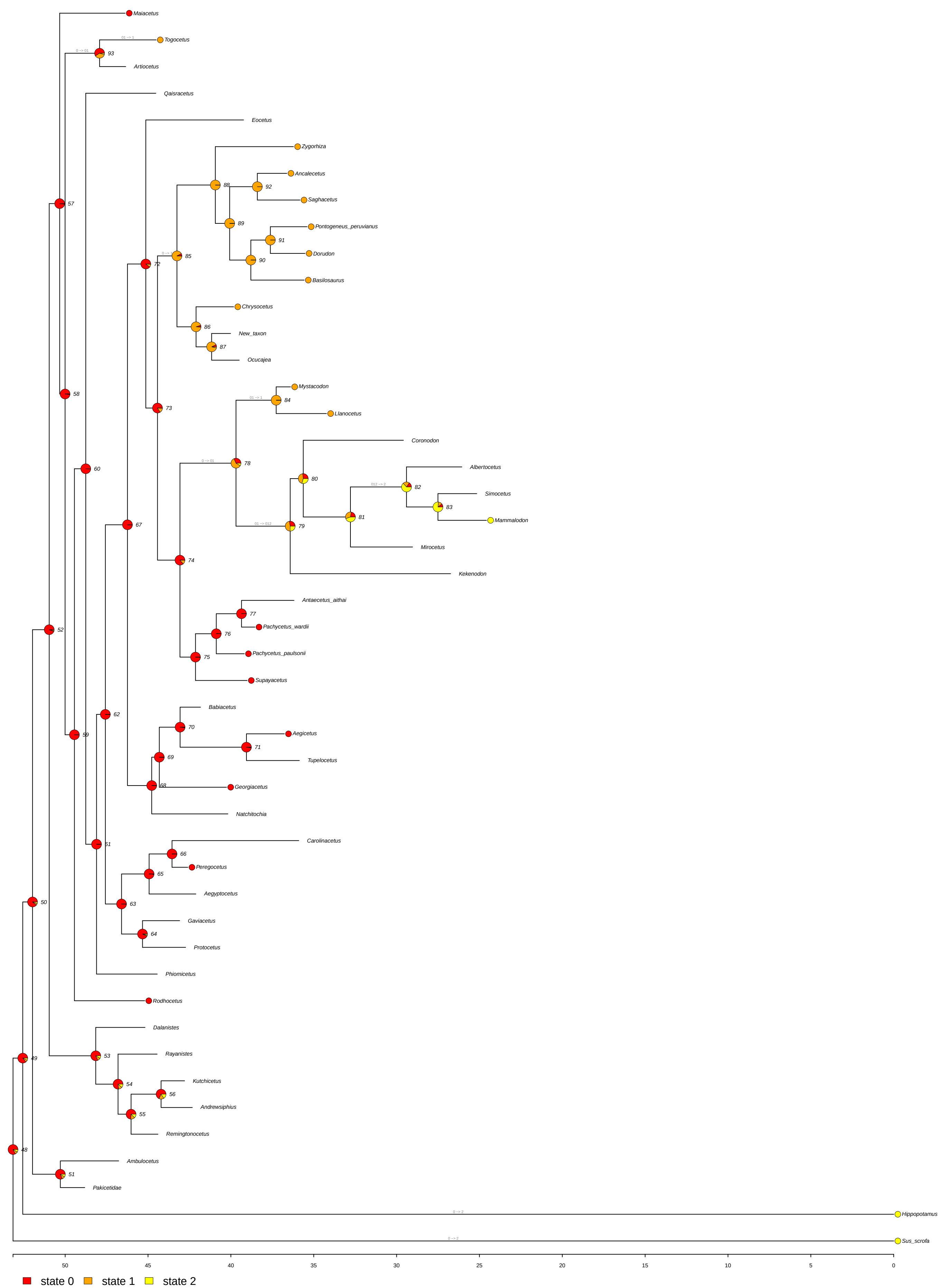
Maiacetus
Togocetus
Artiocetus
Qaisracetus
Eocetus
Zygorhiza
Ancalecetus
Saghacetus
Pontogeneus_peruvianus
Dorudon
Basilosaurus
Chrysocetus
New_taxon
Ocucajea
Mystacodon
Llanocetus
Coronodon
Albertocetus
Simocetus
Mammalodon
Mirocetus
Kekenodon
Antaecetus_aithai
Pachycetus_wardii
Pachycetus_paulsonii
Supayacetus
Babiacetus
Aegicetus
Tupelocetus
Georgiacetus
Natchitochia
Carolinacetus
Peregocetus
Aegyptocetus
Gaviacetus
Protocetus
Phiomicetus
Rodhocetus
Dalanistes
Rayanistes
Kutchicetus
Andrewsiphius
Remingtonocetus
Ambulocetus
Pakicetidae
Hippopotamus
Sus_scrofa
50 45 40 35 30 25 20 15 10 5 0
state 0
state 1
state 2

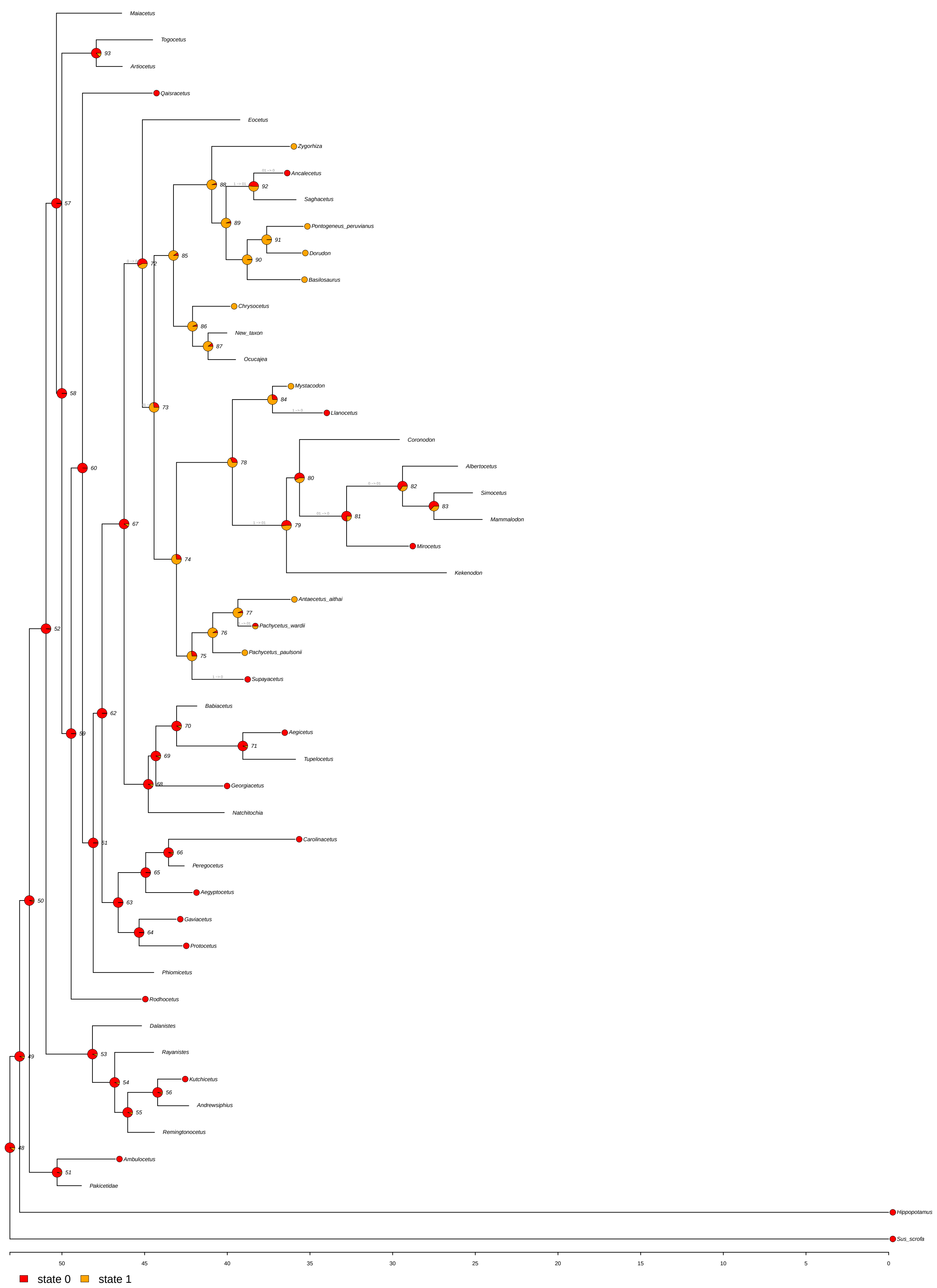

Maiacetus
Togocetus
Artiocetus
Qaisracetus
Eocetus
Zygorhiza
Ancalecetus
Saghacetus
Pontogeneus_peruvianus
Dorudon
Basilosaurus
Chrysocetus
New_taxon
Ocucajea
Mystacodon
Llanocetus
Coronodon
Albertocetus
Simocetus
Mammalodon
Mirocetus
Kekenodon
Antaecetus_aithai
Pachycetus_wardii
Pachycetus_paulsonii
Supayacetus
Babiacetus
Aegicetus
Tupelocetus
Georgiacetus
Natchitochia
Carolinacetus
Peregocetus
Aegyptocetus
Gaviacetus
Protocetus
Phiomicetus
Rodhocetus
Dalanistes
Rayanistes
Kutchicetus
Andrewsiphius
Remingtonocetus
Ambulocetus
Pakicetidae
Hippopotamus
Sus_scrofa
50
45
40
35
30
25
20
15
10
5
0
state 0
state 1

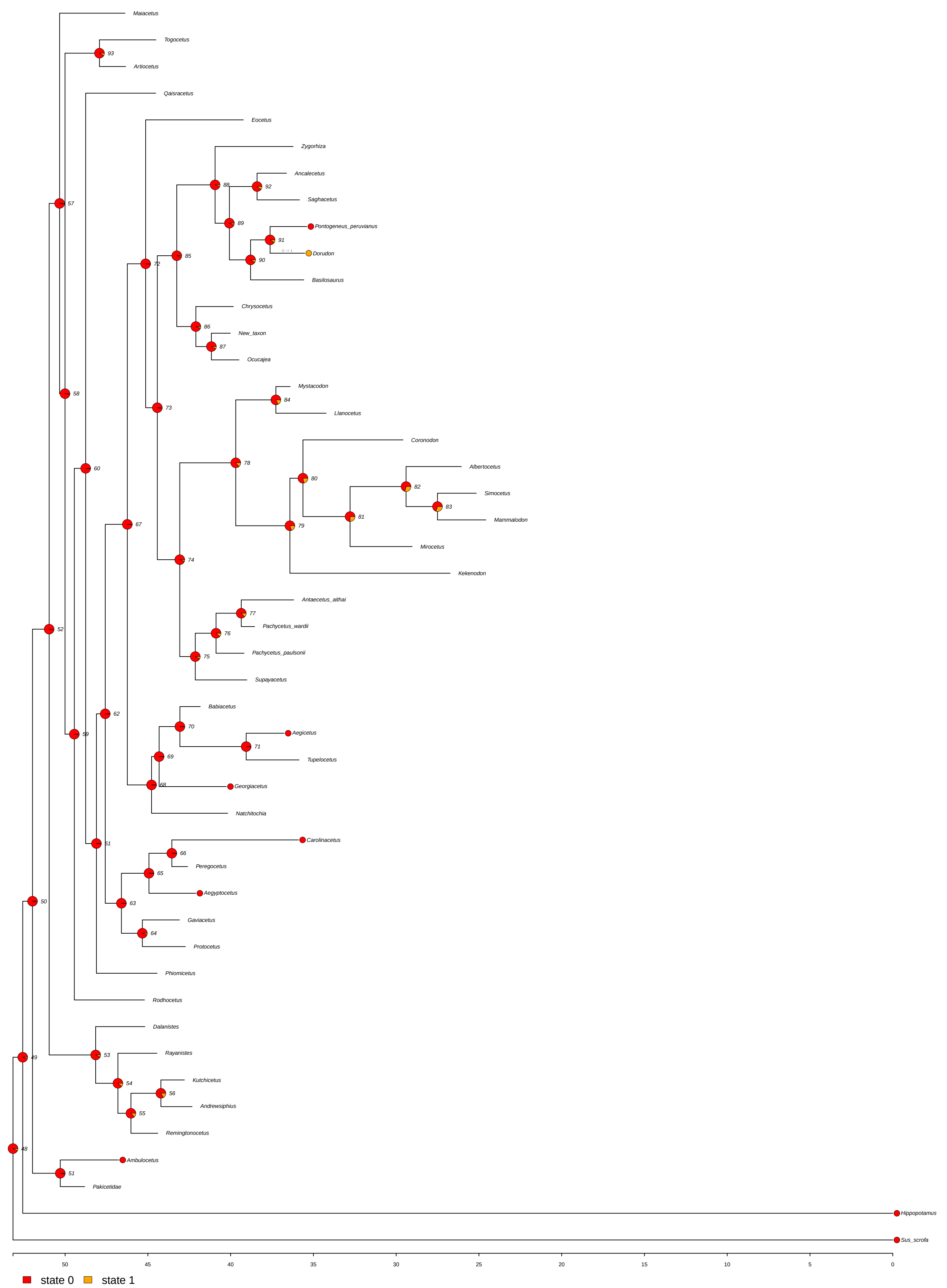
Maiacetus
Togocetus
93
Artiocetus
Qaisracetus
Eocetus
Zygorhiza
Ancalecetus
92
Saghacetus
88
89
Pontogeneus_peruvianus
91
Dorudon
90
Basilosaurus
85
72
Chrysocetus
86
New_taxon
87
Ocucajea
Mystacodon
84
Llanocetus
Coronodon
78
80
Albertocetus
82
Simocetus
83
Mammalodon
81
79
Mirocetus
Kekenodon
74
73
57
58
60
67
Antaecetus_aithai
77
Pachycetus_wardii
76
Pachycetus_paulsonii
75
Supayacetus
52
Babiacetus
62
70
Aegicetus
71
Tupelocetus
59
69
68
Georgiacetus
Natchitochia
Carolinacetus
66
Peregocetus
61
65
Aegyptocetus
50
63
Gaviacetus
64
Protocetus
Phiomicetus
Rodhocetus
Dalanistes
Rayanistes
53
49
54
Kutchicetus
56
Andrewsiphius
55
Remingtonocetus
48
Ambulocetus
51
Pakicetidae
Hippopotamus
Sus_scrofa
50
45
40
35
30
25
20
15
10
5
0
state 0
state 1

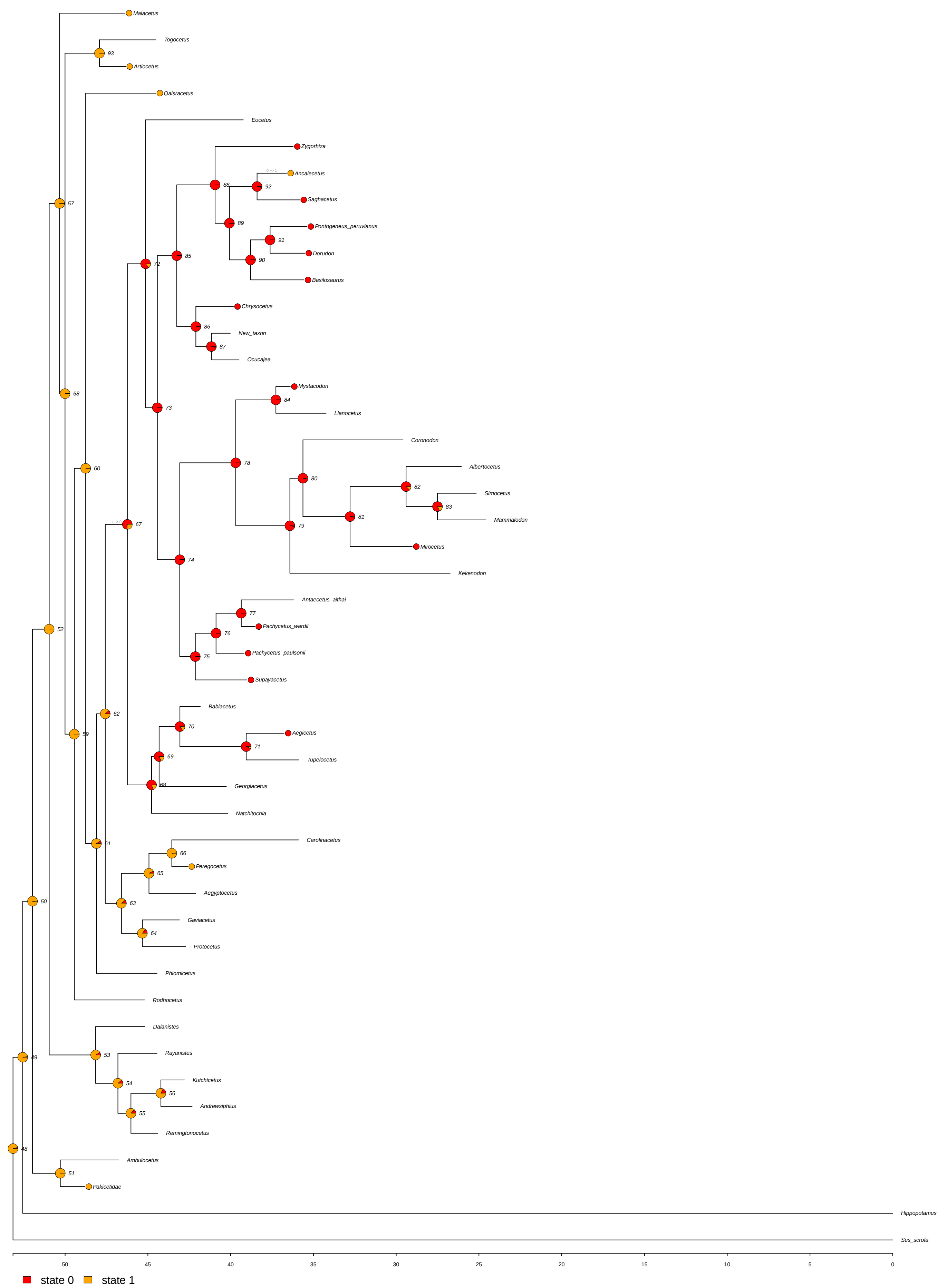

Maiacetus
Togocetus
93
Artiocetus
Qaisracetus
Eocetus
Zygorhiza
0 -> 1
Ancalecetus
88
92
Saghacetus
Pontogeneus_peruvianus
89
91
Dorudon
72
85
90
Basilosaurus
57
Chrysocetus
86
New_taxon
87
Ocucajea
Mystacodon
84
58
73
Llanocetus
Coronodon
60
78
Albertocetus
80
82
Simocetus
83
1 -> 0
67
81
Mammalodon
79
Mirocetus
74
Kekenodon
Antaecetus_aithai
77
Pachycetus_wardii
52
76
Pachycetus_paulsonii
75
Supayacetus
Babiacetus
62
59
70
Aegicetus
71
Tupelocetus
69
68
Georgiacetus
Natchitochia
61
Carolinacetus
66
Peregocetus
65
50
Aegyptocetus
63
Gaviacetus
64
Protocetus
Phiomicetus
Rodhocetus
Dalanistes
53
Rayanistes
49
54
Kutchicetus
56
Andrewsiphius
55
Remingtonocetus
48
Ambulocetus
51
Pakicetidae
Hippopotamus
Sus_scrofa
50
45
40
35
30
25
20
15
10
5
0
state 0
state 1

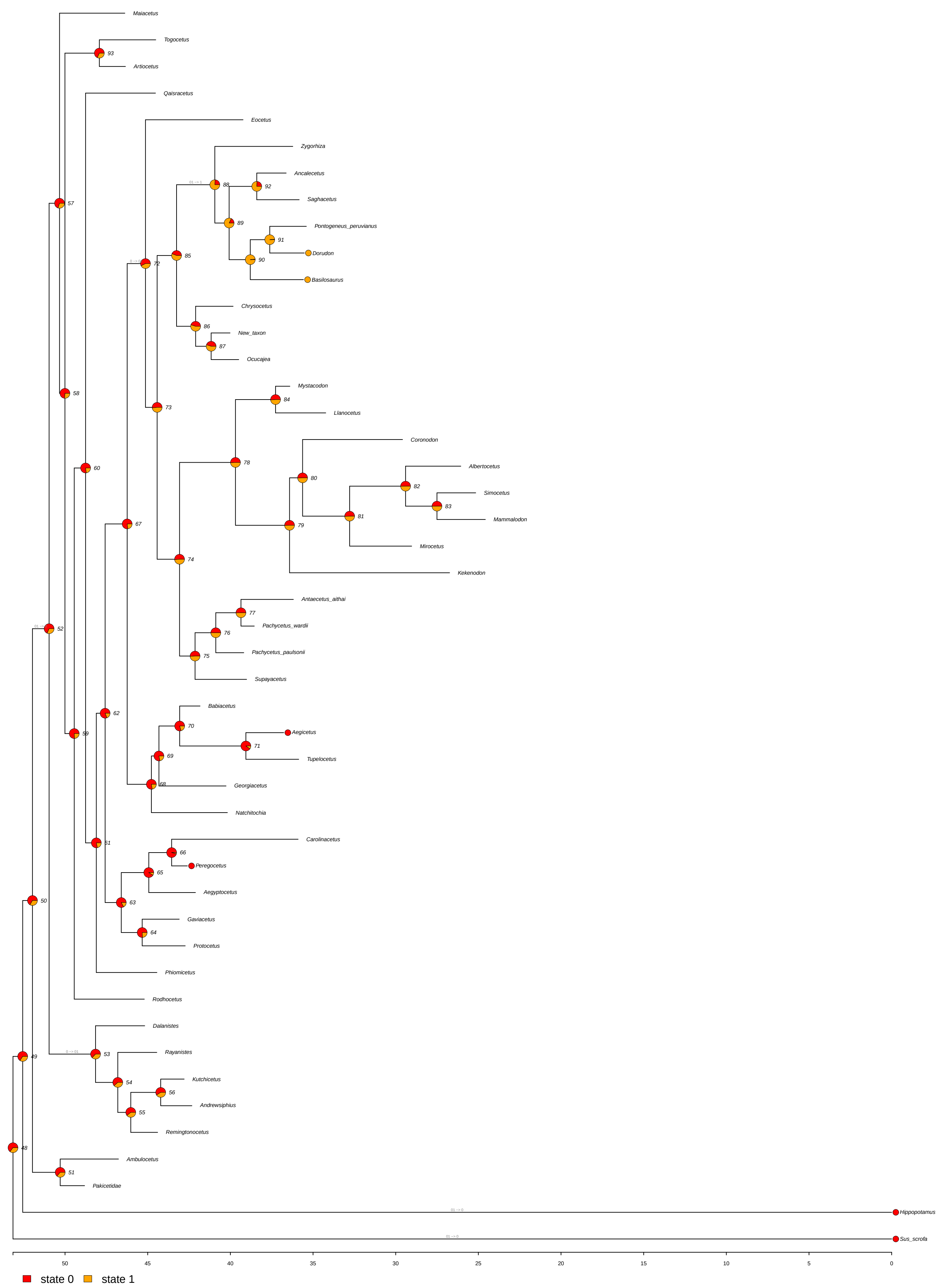

Maiacetus
Togocetus
93
Artiocetus
Qaisracetus
Eocetus
Zygorhiza
Ancalecetus
88
92
Saghacetus
89
Pontogeneus_peruvianus
91
Dorudon
90
Basilosaurus
72
85
Chrysocetus
86
New_taxon
87
Ocucajea
57
Mystacodon
84
Llanocetus
58
73
Coronodon
78
Albertocetus
80
82
Simocetus
83
Mammalodon
81
60
67
79
Mirocetus
74
Kekenodon
Antaecetus_aithai
77
Pachycetus_wardii
52
76
Pachycetus_paulsonii
75
Supayacetus
Babiacetus
62
70
Aegicetus
59
71
Tupelocetus
69
68
Georgiacetus
Natchitochia
Carolinacetus
61
66
Peregocetus
65
Aegyptocetus
50
63
Gaviacetus
64
Protocetus
Phiomicetus
Rodhocetus
Dalanistes
Rayanistes
49
53
Kutchicetus
54
56
Andrewsiphius
55
Remingtonocetus
48
Ambulocetus
51
Pakicetidae
Hippopotamus
Sus_scrofa
50
45
40
35
30
25
20
15
10
5
0
state 0
state 1

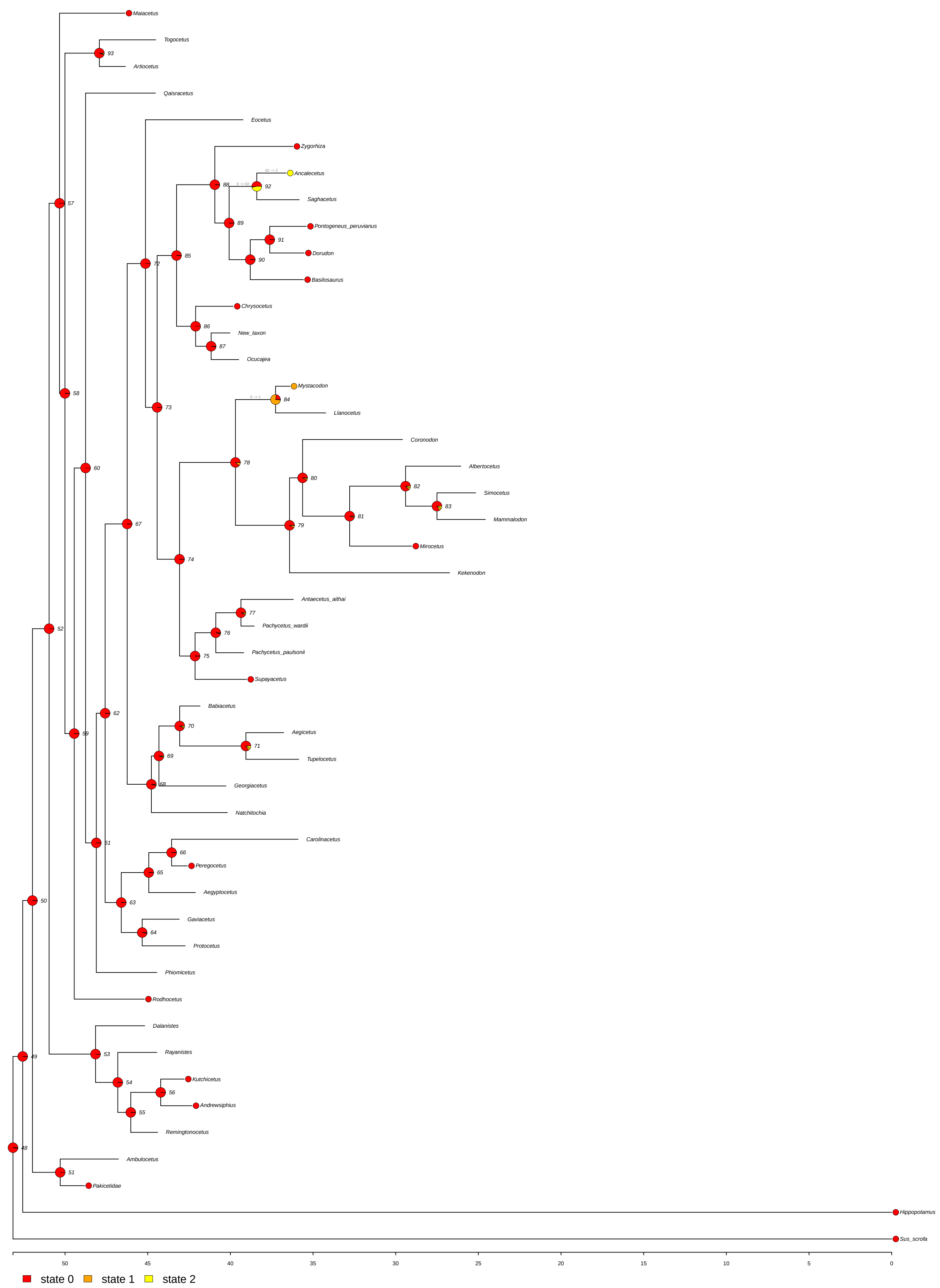

Maiacetus
Togocetus
93
Artiocetus
Qaisracetus
Eocetus
Zygorhiza
Ancalecetus
88
92
Saghacetus
89
Pontogeneus_peruvianus
91
Dorudon
90
Basilosaurus
72
85
Chrysocetus
86
New_taxon
87
Ocucajea
57
58
Mystacodon
84
Llanocetus
73
Coronodon
78
80
Albertocetus
82
Simocetus
83
Mammalodon
81
79
Mirocetus
74
Kekenodon
Antaecetus_aithai
77
Pachycetus_wardii
76
Pachycetus_paulsonii
75
Supayacetus
60
67
52
Babiacetus
70
Aegicetus
71
Tupelocetus
69
62
59
68
Georgiacetus
Natchitochia
Carolinacetus
66
Peregocetus
65
61
Aegyptocetus
50
63
Gaviacetus
64
Protocetus
Phiomicetus
Rodhocetus
Dalanistes
53
Rayanistes
49
54
Kutchicetus
56
Andrewsiphius
55
Remingtonocetus
48
Ambulocetus
51
Pakicetidae
Hippopotamus
Sus_scrofa
50
45
40
35
30
25
20
15
10
5
0
state 0
state 1
state 2

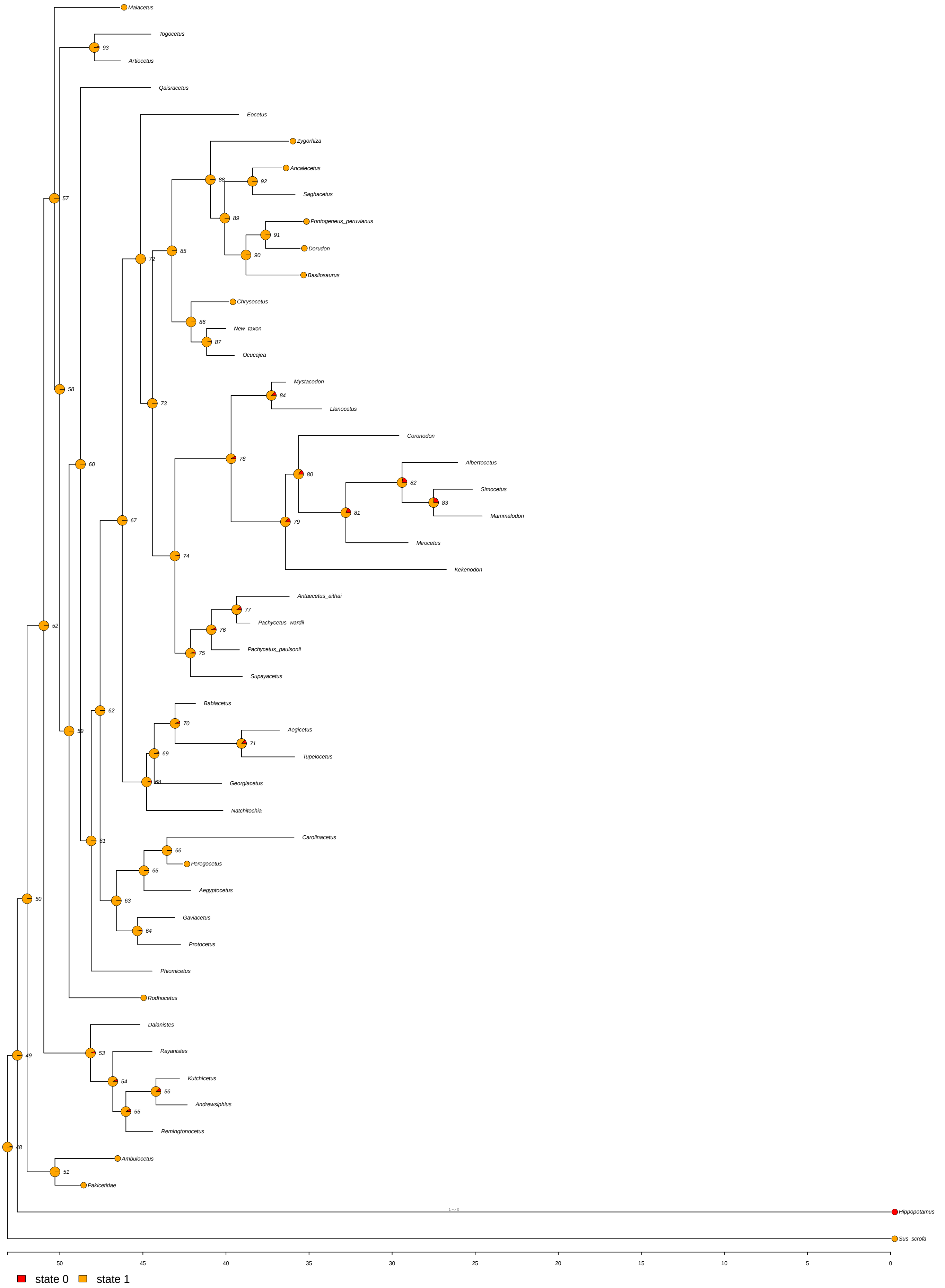

Maiacetus
Togocetus
93
Artiocetus
Qaisracetus
Eocetus
Zygorhiza
Ancalecetus
88
92
Saghacetus
89
Pontogeneus_peruvianus
91
Dorudon
90
Basilosaurus
72
85
Chrysocetus
86
New_taxon
87
Ocucajea
57
Mystacodon
84
Llanocetus
58
73
Coronodon
78
Albertocetus
80
82
Simocetus
83
Mammalodon
81
79
Mirocetus
60
67
74
Kekenodon
Antaecetus_aithai
77
Pachycetus_wardii
76
Pachycetus_paulsonii
75
52
Supayacetus
Babiacetus
62
70
Aegicetus
59
71
Tupelocetus
69
68
Georgiacetus
Natchitochia
Carolinacetus
61
66
Peregocetus
65
Aegyptocetus
50
63
Gaviacetus
64
Protocetus
Phiomicetus
Rodhocetus
Dalanistes
Rayanistes
53
49
54
Kutchicetus
56
Andrewsiphius
55
Remingtonocetus
48
Ambulocetus
51
Pakicetidae
Hippopotamus
Sus_scrofa
50
45
40
35
30
25
20
15
10
5
0
state 0
state 1

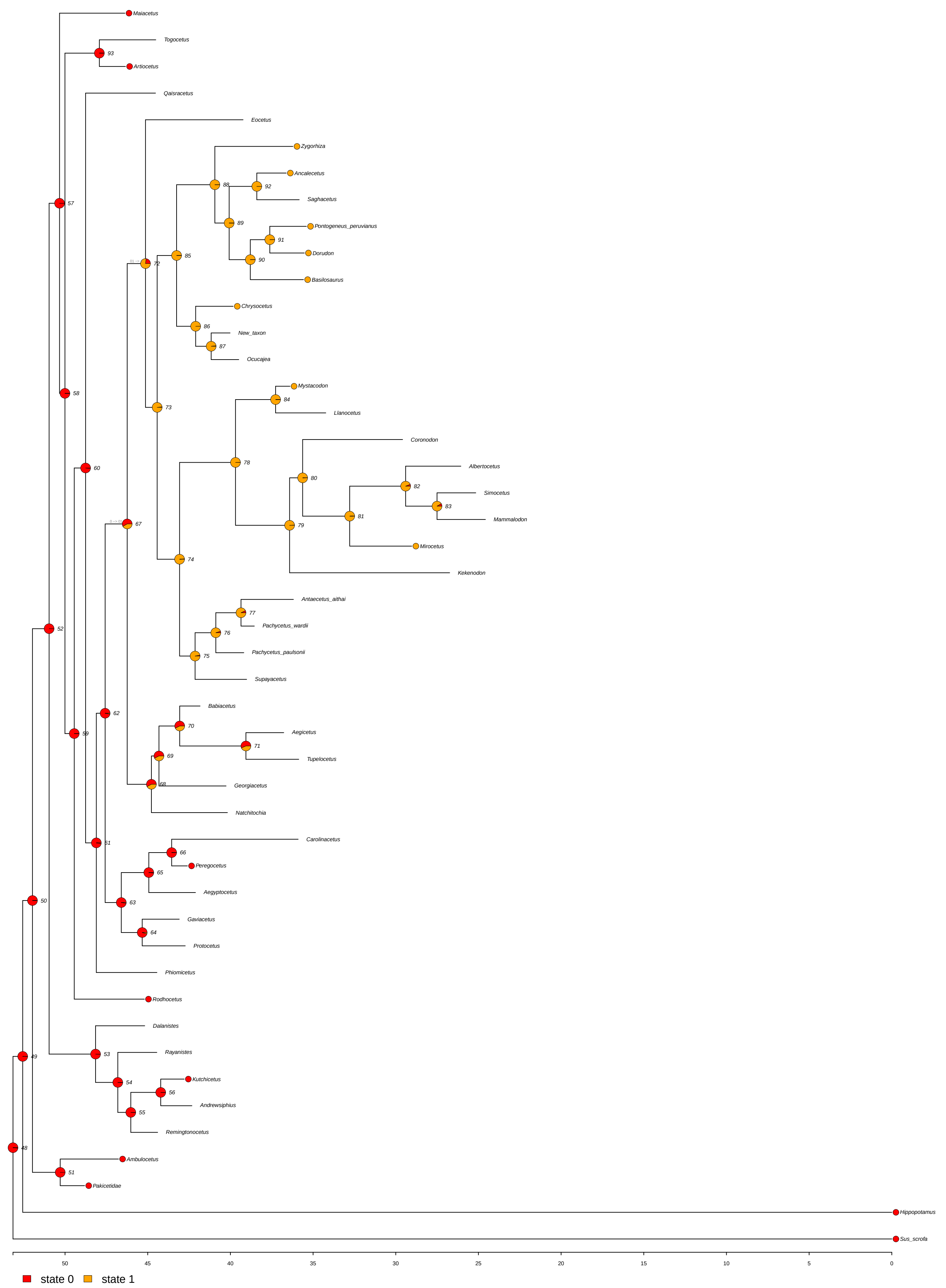

Maiacetus
Togocetus
93
Artiocetus
Qaisracetus
Eocetus
Zygorhiza
Ancalecetus
88
92
Saghacetus
Pontogeneus_peruvianus
89
91
Dorudon
90
85
72
Basilosaurus
Chrysocetus
86
New_taxon
87
Ocucajea
57
Mystacodon
84
58
Llanocetus
73
Coronodon
60
78
Albertocetus
80
82
Simocetus
83
81
Mammalodon
67
79
Mirocetus
74
Kekenodon
Antaecetus_aithai
77
52
76
Pachycetus_wardii
Pachycetus_paulsonii
75
Supayacetus
Babiacetus
62
70
59
Aegicetus
71
Tupelocetus
69
68
Georgiacetus
Natchitochia
Carolinacetus
61
66
Peregocetus
65
Aegyptocetus
50
63
Gaviacetus
64
Protocetus
Phiomicetus
Rodhocetus
Dalanistes
Rayanistes
53
49
54
Kutchicetus
56
Andrewsiphius
55
Remingtonocetus
48
Ambulocetus
51
Pakicetidae
Hippopotamus
Sus_scrofa
50
45
40
35
30
25
20
15
10
5
0
state 0
state 1

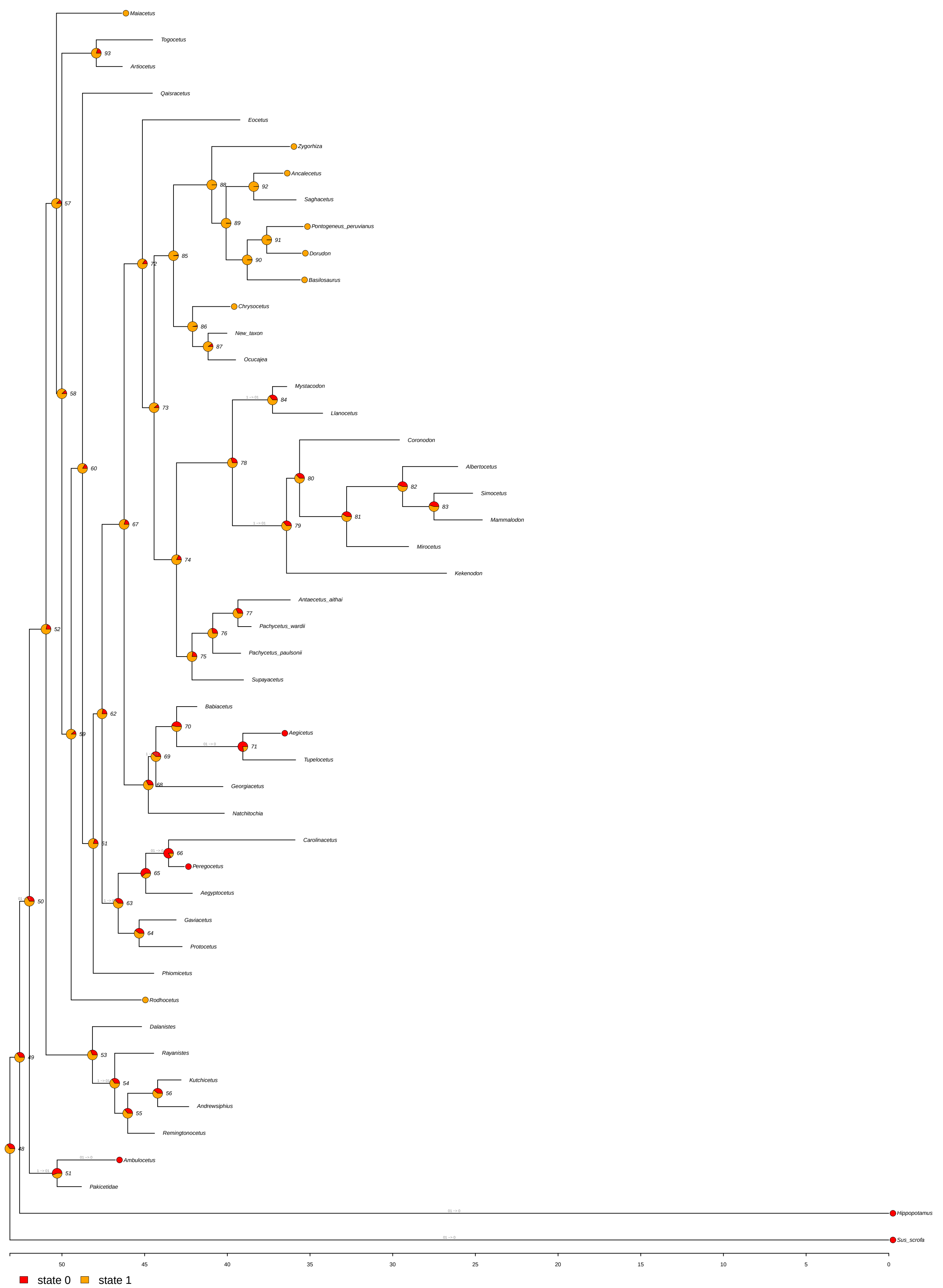
Maiacetus
Togocetus
93
Artiocetus
Qaisracetus
Eocetus
Zygorhiza
Ancalecetus
88
92
Saghacetus
Pontogeneus_peruvianus
89
91
Dorudon
90
Basilosaurus
57
72
85
Chrysocetus
New_taxon
86
87
Ocucajea
Mystacodon
84
Llanocetus
58
73
Coronodon
Albertocetus
60
78
80
82
Simocetus
83
Mammalodon
67
79
81
Mirocetus
74
Kekenodon
Antaecetus_aithai
77
Pachycetus_wardii
52
76
Pachycetus_paulsonii
75
Supayacetus
Babiacetus
62
70
Aegicetus
59
71
Tupelocetus
69
68
Georgiacetus
Natchitochia
Carolinacetus
61
66
Peregocetus
65
Aegyptocetus
50
63
Gaviacetus
64
Protocetus
Phiomicetus
Rodhocetus
Dalanistes
53
Rayanistes
49
Kutchicetus
54
56
Andrewsiphius
55
Remingtonocetus
48
Ambulocetus
51
Pakicetidae
Hippopotamus
Sus_scrofa
50
45
40
35
30
25
20
15
10
5
0
state 0
state 1

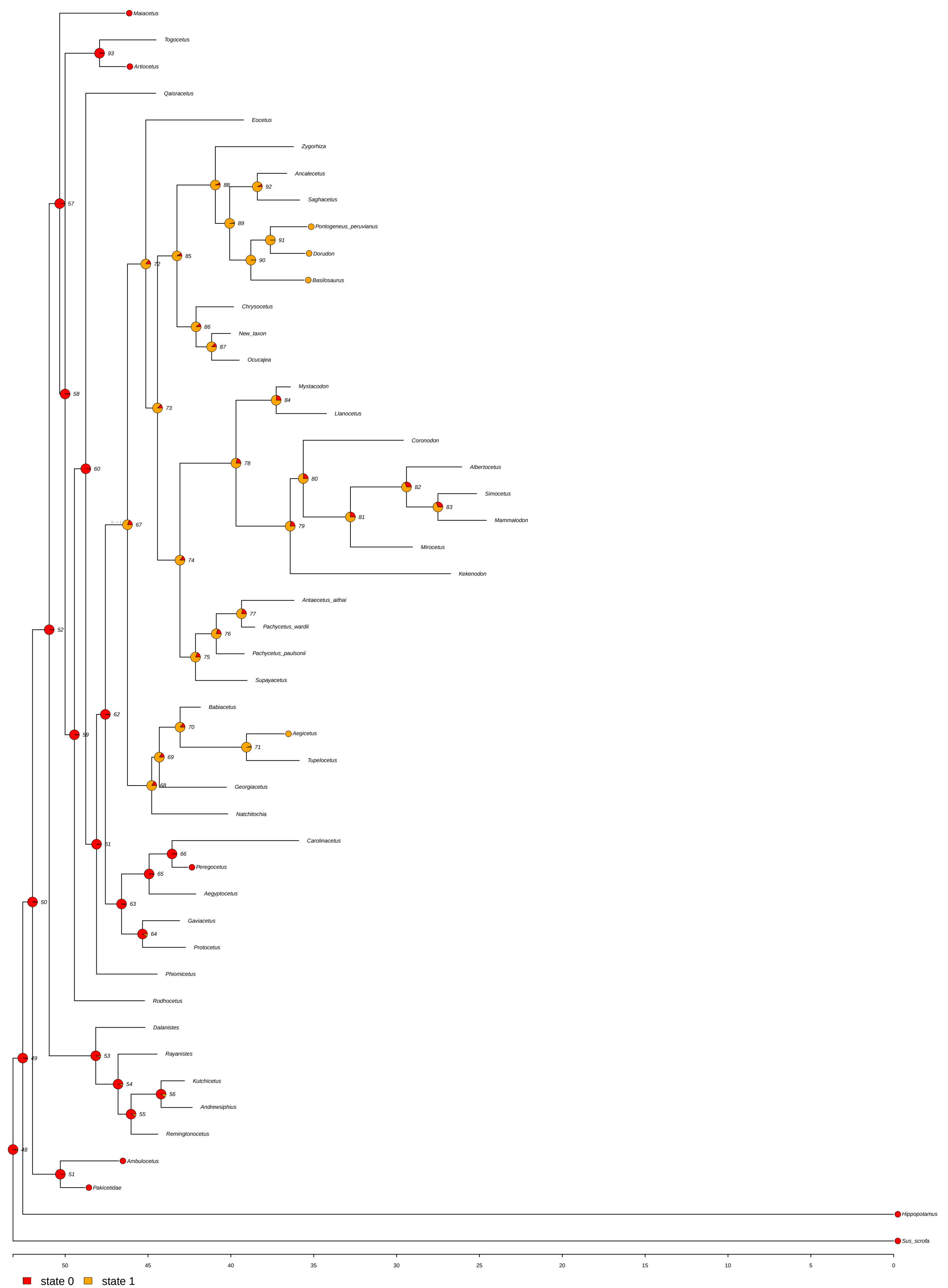

Maiacetus
Togocetus
93
Artiocetus
Qaisracetus
Eocetus
Zygorhiza
Ancalecetus
88
92
Saghacetus
89
Pontogeneus_peruvianus
91
Dorudon
90
Basilosaurus
72
85
57
Chrysocetus
86
New_taxon
87
Ocucajea
Mystacodon
84
Llanocetus
58
73
Coronodon
78
60
80
Albertocetus
82
Simocetus
83
Mammalodon
81
67
79
Mirocetus
74
Kekenodon
Antaecetus_aithai
77
Pachycetus_wardii
76
Pachycetus_paulsonii
52
75
Supayacetus
Babiacetus
62
70
Aegicetus
59
71
Tupelocetus
69
68
Georgiacetus
Natchitochia
Carolinacetus
61
66
Peregocetus
65
Aegyptocetus
50
63
Gaviacetus
64
Protocetus
Phiomicetus
Rodhocetus
Dalanistes
53
Rayanistes
49
54
Kutchicetus
56
Andrewsiphius
55
Remingtonocetus
48
Ambulocetus
51
Pakicetidae
Hippopotamus
Sus_scrofa
50
45
40
35
30
25
20
15
10
5
0
state 0
state 1

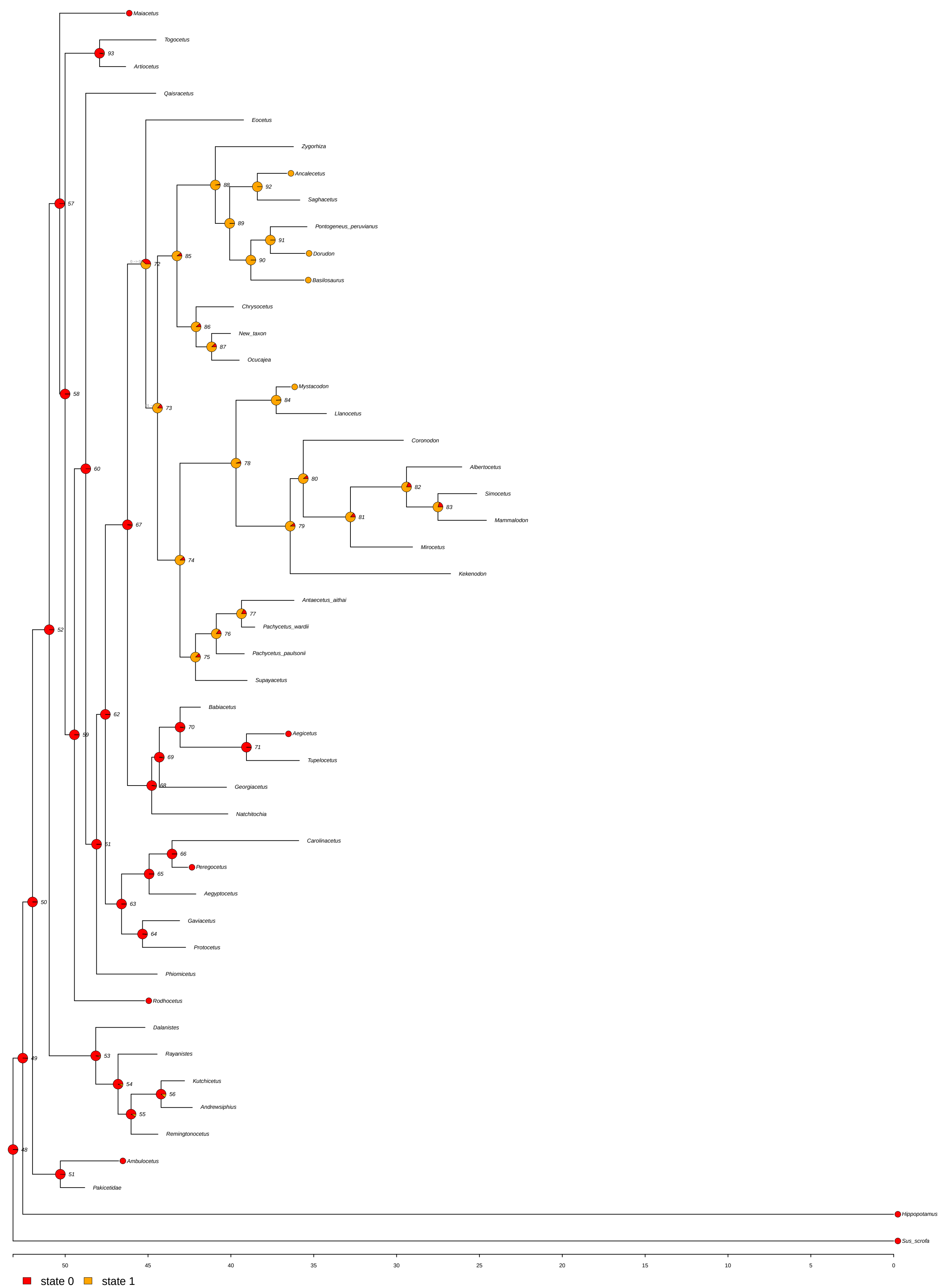

Maiacetus
Togocetus
93
Artiocetus
Qaisracetus
Eocetus
Zygorhiza
Ancalecetus
88
92
Saghacetus
89
Pontogeneus_peruvianus
91
Dorudon
90
85
72
Basilosaurus
Chrysocetus
86
New_taxon
87
Ocucajea
Mystacodon
84
Llanocetus
73
Coronodon
78
Albertocetus
80
82
Simocetus
83
81
79
Mammalodon
Mirocetus
74
Kekenodon
Antaecetus_aithai
77
Pachycetus_wardii
76
Pachycetus_paulsonii
75
Supayacetus
Babiacetus
70
Aegicetus
71
Tupelocetus
69
68
Georgiacetus
Natchitochia
Carolinacetus
66
Peregocetus
65
Aegyptocetus
63
Gaviacetus
64
Protocetus
Phiomicetus
Rodhocetus
Dalanistes
53
Rayanistes
54
Kutchicetus
56
Andrewsiphius
55
Remingtonocetus
57
58
60
67
52
62
59
61
50
49
48
Ambulocetus
51
Pakicetidae
Hippopotamus
Sus_scrofa
50
45
40
35
30
25
20
15
10
5
0
state 0
state 1

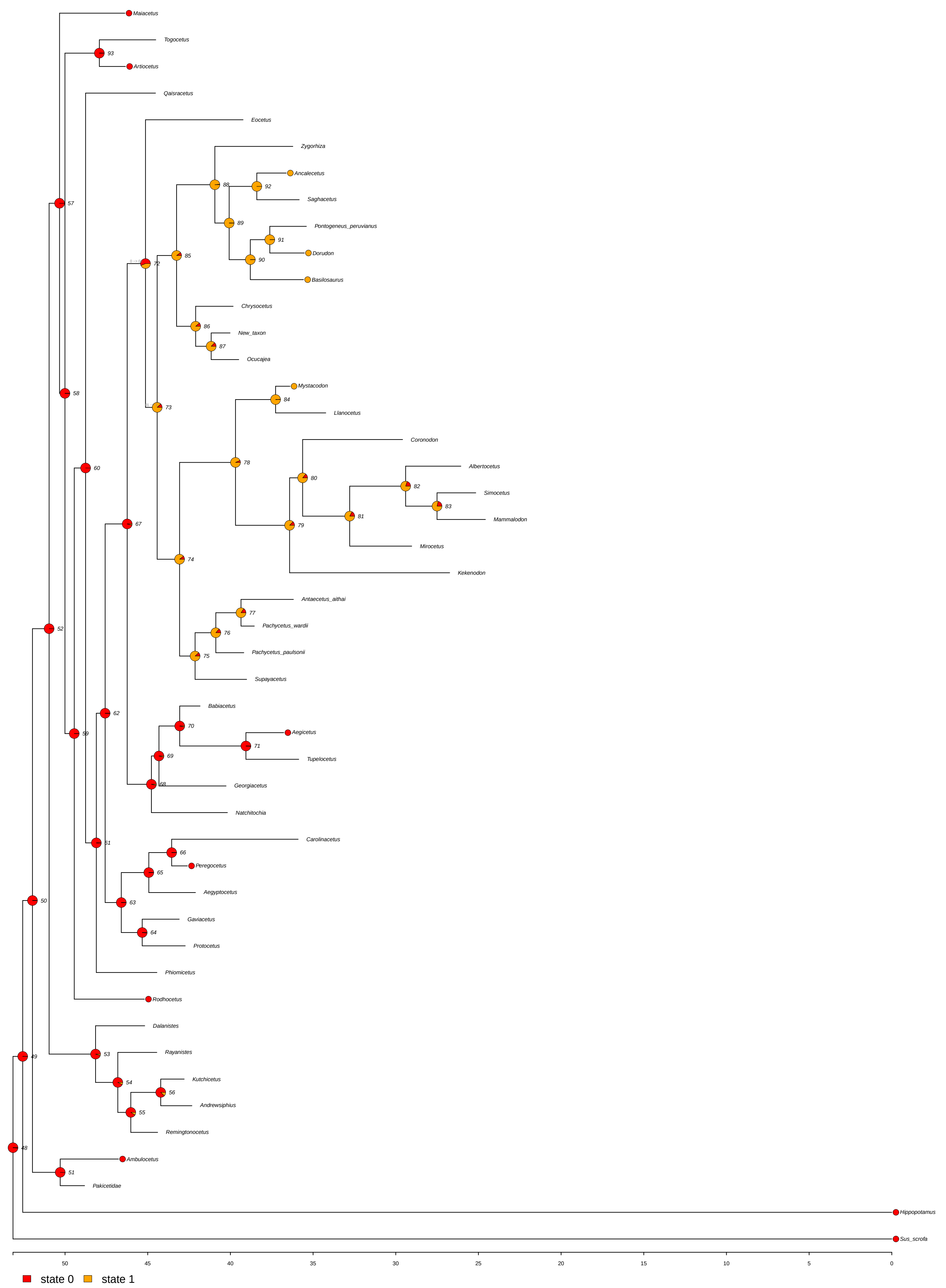

Maiacetus
Togocetus
Artiocetus
Qaisracetus
Eocetus
Zygorhiza
Ancalecetus
Saghacetus
Pontogeneus_peruvianus
Dorudon
Basilosaurus
Chrysocetus
New_taxon
Ocucajea
Mystacodon
Llanocetus
Coronodon
Albertocetus
Simocetus
Mammalodon
Mirocetus
Kekenodon
Antaecetus_aithai
Pachycetus_wardii
Pachycetus_paulsonii
Supayacetus
Babiacetus
Aegicetus
Tupelocetus
Georgiacetus
Natchitochia
Carolinacetus
Peregocetus
Aegyptocetus
Gaviacetus
Protocetus
Phiomicetus
Rodhocetus
Dalanistes
Rayanistes
Kutchicetus
Andrewsiphius
Remingtonocetus
Ambulocetus
Pakicetidae
Hippopotamus
Sus_scrofa
50
45
40
35
30
25
20
15
10
5
0
state 0
state 1

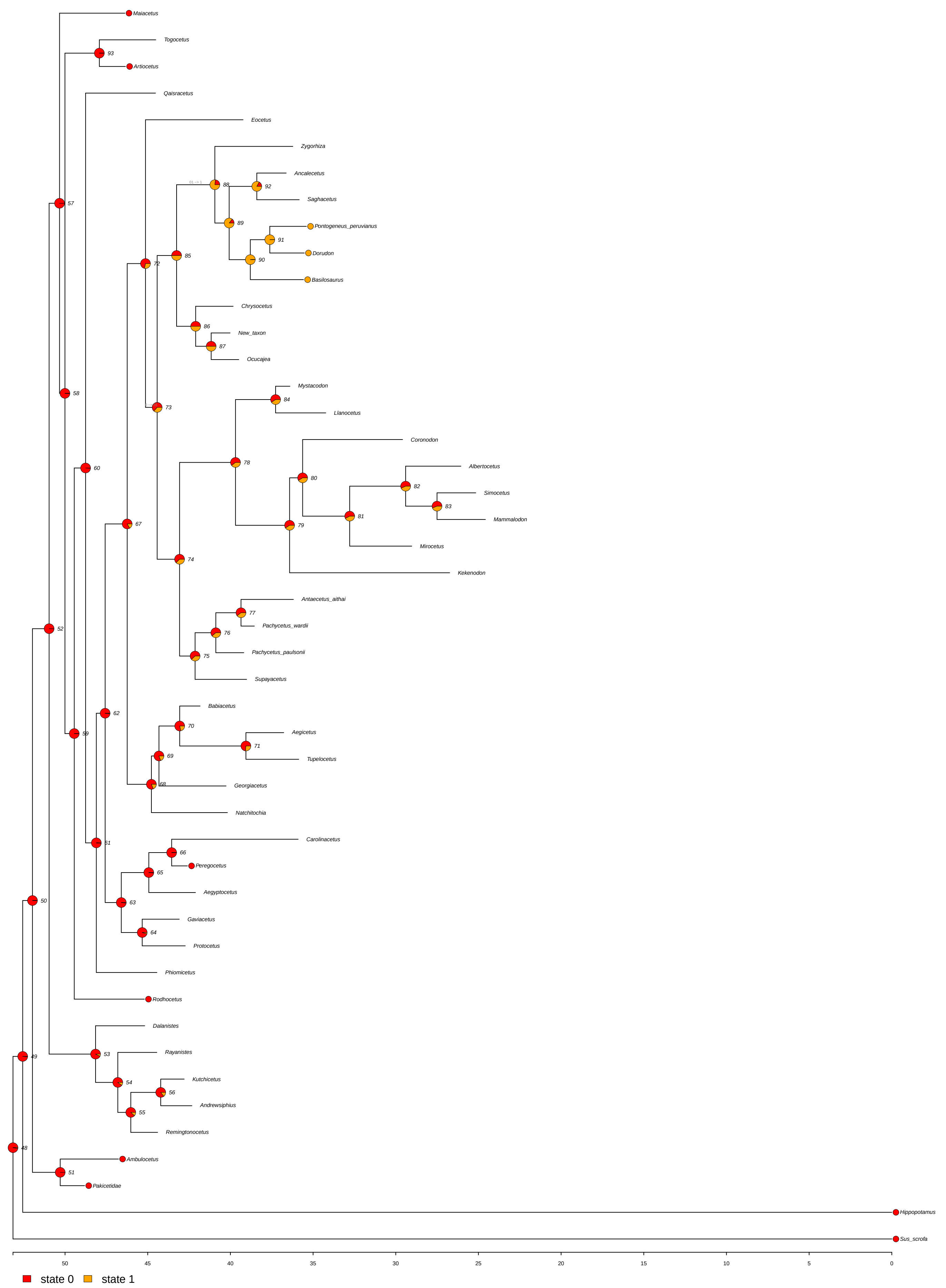
Maiacetus
Togocetus
Artiocetus
Qaisracetus
Eocetus
Zygorhiza
Ancalecetus
Saghacetus
Pontogeneus_peruvianus
Dorudon
Basilosaurus
Chrysocetus
New_taxon
Ocucajea
Mystacodon
Llanocetus
Coronodon
Albertocetus
Simocetus
Mammalodon
Mirocetus
Kekenodon
Antaecetus_aithai
Pachycetus_wardii
Pachycetus_paulsonii
Supayacetus
Babiacetus
Aegicetus
Tupelocetus
Georgiacetus
Natchitochia
Carolinacetus
Peregocetus
Aegyptocetus
Gaviacetus
Protocetus
Phiomicetus
Rodhocetus
Dalanistes
Rayanistes
Kutchicetus
Andrewsiphius
Remingtonocetus
Ambulocetus
Pakicetidae
Hippopotamus
Sus_scrofa
50
45
40
35
30
25
20
15
10
5
0
state 0
state 1

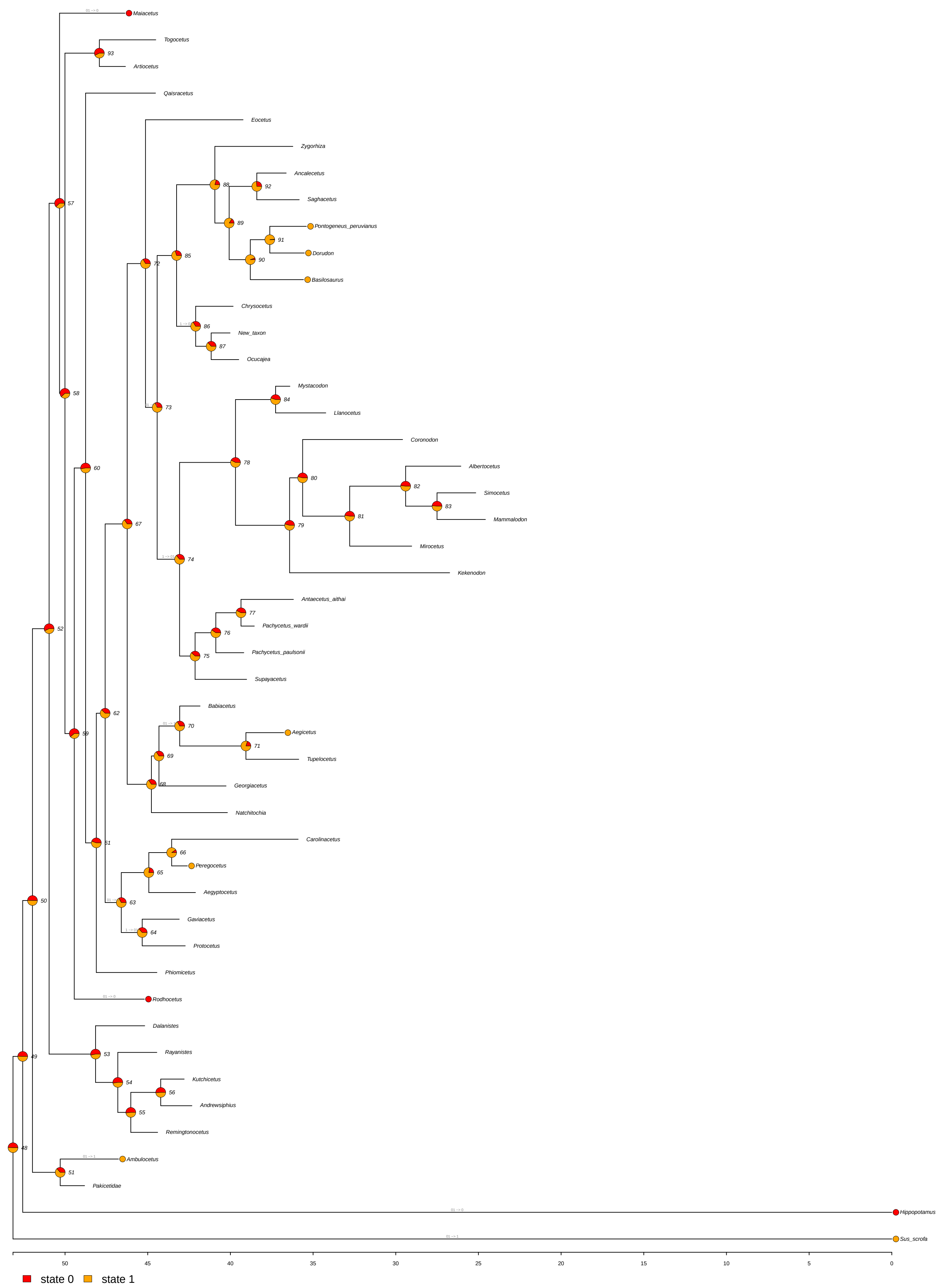

Maiacetus
Togocetus
Artiocetus
Qaisracetus
Eocetus
Zygorhiza
Ancalecetus
Saghacetus
Pontogeneus_peruvianus
Dorudon
Basilosaurus
Chrysocetus
New_taxon
Ocucajea
Mystacodon
Llanocetus
Coronodon
Albertocetus
Simocetus
Mammalodon
Mirocetus
Kekenodon
Antaecetus_aithai
Pachycetus_wardii
Pachycetus_paulsonii
Supayacetus
Babiacetus
Aegicetus
Tupelocetus
Georgiacetus
Natchitochia
Carolinacetus
Peregocetus
Aegyptocetus
Gaviacetus
Protocetus
Phiomicetus
Rodhocetus
Dalanistes
Rayanistes
Kutchicetus
Andrewsiphius
Remingtonocetus
Ambulocetus
Pakicetidae
Hippopotamus
Sus_scrofa
50
45
40
35
30
25
20
15
10
5
0
state 0
state 1

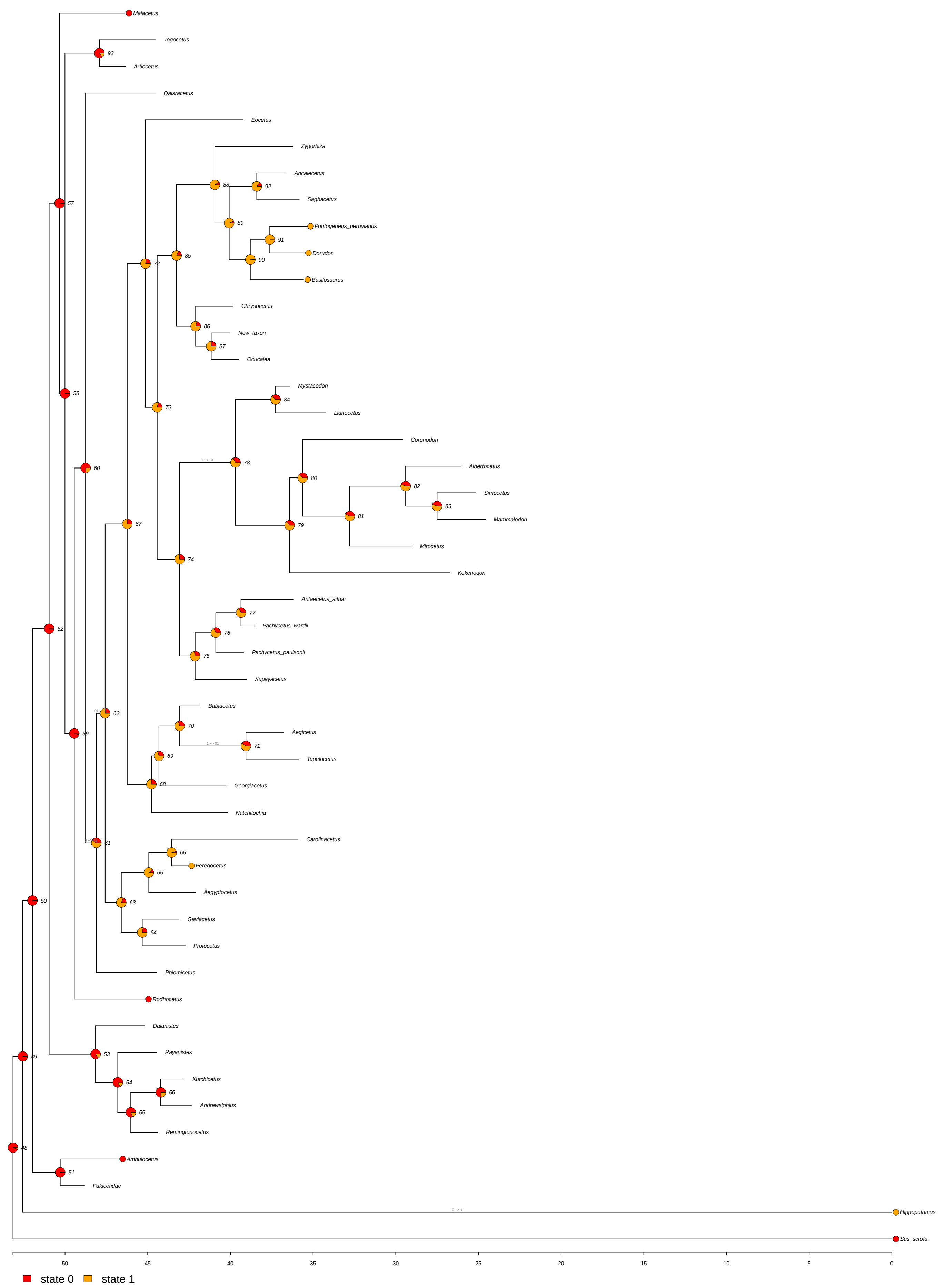

Maiacetus
Togocetus
Artiocetus
Qaisracetus
Eocetus
Zygorhiza
Ancalecetus
Saghacetus
Pontogeneus_peruvianus
Dorudon
Basilosaurus
Chrysocetus
New_taxon
Ocucajea
Mystacodon
Llanocetus
Coronodon
Albertocetus
Simocetus
Mammalodon
Mirocetus
Kekenodon
Antaecetus_aithai
Pachycetus_wardii
Pachycetus_paulsonii
Supayacetus
Babiacetus
Aegicetus
Tupelocetus
Georgiacetus
Natchitochia
Carolinacetus
Peregocetus
Aegyptocetus
Gaviacetus
Protocetus
Phiomicetus
Rodhocetus
Dalanistes
Rayanistes
Kutchicetus
Andrewsiphius
Remingtonocetus
Ambulocetus
Pakicetidae
Hippopotamus
Sus_scrofa
50
45
40
35
30
25
20
15
10
5
0
state 0
state 1

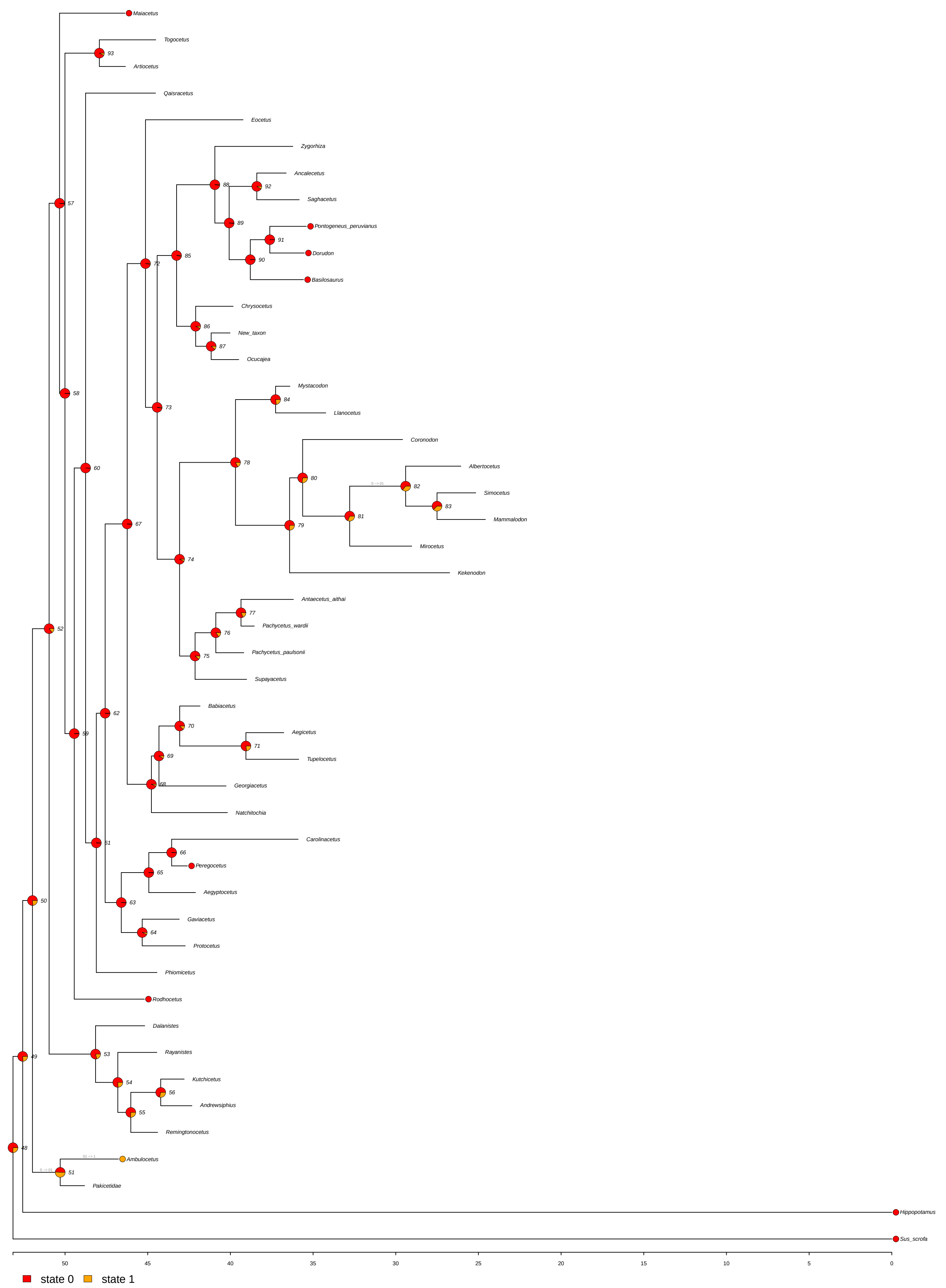

Maiacetus
Togocetus
93
Artiocetus
Qaisracetus
Eocetus
Zygorhiza
Ancalecetus
92
Saghacetus
88
89
Pontogeneus_peruvianus
91
Dorudon
90
Basilosaurus
72
85
57
Chrysocetus
86
New_taxon
87
Ocucajea
Mystacodon
84
Llanocetus
58
73
Coronodon
60
78
Albertocetus
80
0 --> 01
82
Simocetus
83
81
Mammalodon
67
79
Mirocetus
74
Kekenodon
Antaecetus_aithai
77
Pachycetus_wardii
52
76
Pachycetus_paulsonii
75
Supayacetus
Babiacetus
62
70
59
Aegicetus
71
Tupelocetus
69
68
Georgiacetus
Natchitochia
61
Carolinacetus
66
Peregocetus
65
50
Aegyptocetus
63
Gaviacetus
64
Protocetus
Phiomicetus
Rodhocetus
Dalanistes
53
Rayanistes
49
54
Kutchicetus
56
Andrewsiphius
55
Remingtonocetus
48
01 --> 1
Ambulocetus
0 --> 01
51
Pakicetidae
Hippopotamus
Sus_scrofa
50
45
40
35
30
25
20
15
10
5
0
state 0
state 1

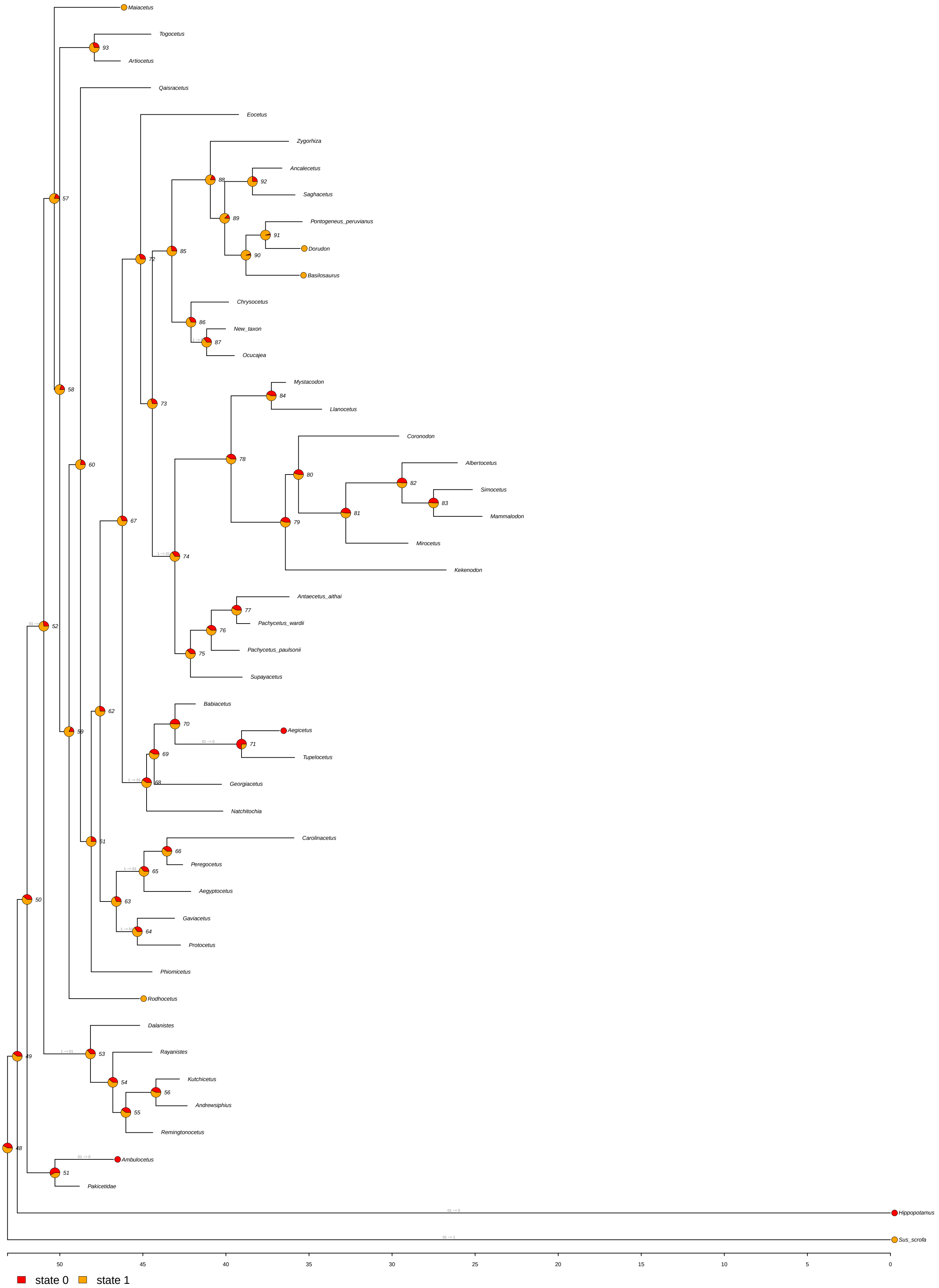

Maiacetus
Togocetus
93
Artiocetus
Qaisracetus
Eocetus
Zygorhiza
Ancalecetus
88
92
Saghacetus
Pontogeneus_peruvianus
89
91
Dorudon
90
Basilosaurus
72
85
57
Chrysocetus
86
New_taxon
87
Ocucajea
Mystacodon
84
58
73
Llanocetus
Coronodon
Albertocetus
60
78
80
82
Simocetus
83
Mammalodon
67
81
79
Mirocetus
74
Kekenodon
Antaecetus_aithai
77
52
Pachycetus_wardii
76
Pachycetus_paulsonii
75
Supayacetus
Babiacetus
62
70
Aegicetus
59
71
Tupelocetus
69
Georgiacetus
68
Natchitochia
Carolinacetus
61
66
Peregocetus
65
Aegyptocetus
50
63
Gaviacetus
64
Protocetus
Phiomicetus
Rodhocetus
Dalanistes
Rayanistes
53
49
54
Kutchicetus
56
Andrewsiphius
55
Remingtonocetus
48
Ambulocetus
51
Pakicetidae
Hippopotamus
Sus_scrofa
50
45
40
35
30
25
20
15
10
5
0
state 0
state 1

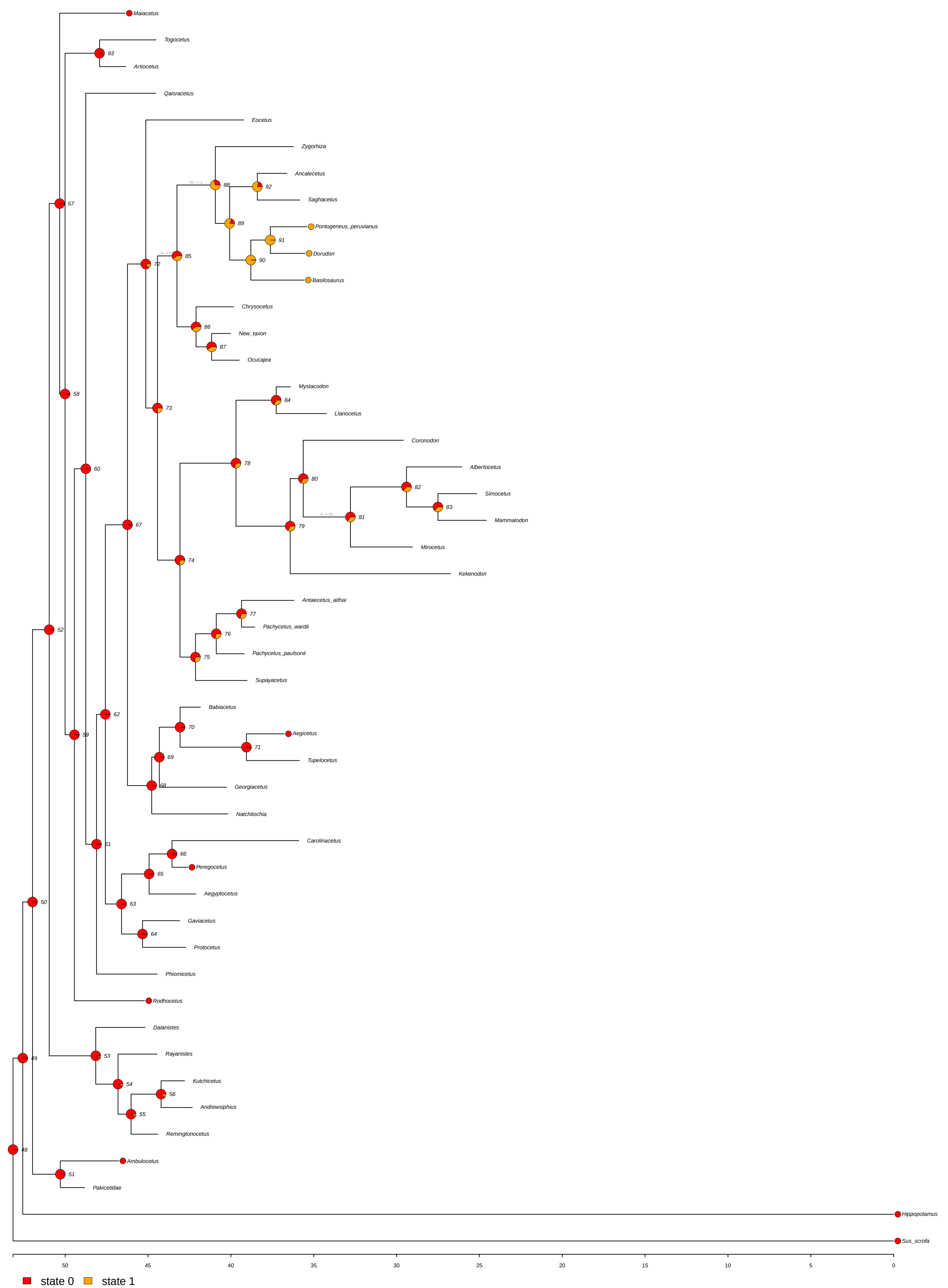
Maiacetus
Togocetus
93
Artiocetus
Qaisracetus
Eocetus
Zygorhiza
Ancalecetus
88
92
Saghacetus
89
Pontogeneus_peruvianus
91
Dorudon
90
Basilosaurus
72
85
Chrysocetus
86
New_taxon
87
Ocucajea
Mystacodon
84
Llanocetus
73
Coronodon
78
Albertocetus
80
82
Simocetus
83
81
Mammalodon
79
Mirocetus
74
Kekenodon
Antaecetus_aithai
77
Pachycetus_wardii
76
Pachycetus_paulsonii
75
Supayacetus
Babiacetus
70
Aegicetus
71
Tupelocetus
69
68
Georgiacetus
Natchitochia
Carolinacetus
66
Peregocetus
65
Aegyptocetus
63
Gaviacetus
64
Protocetus
Phiomicetus
Rodhocetus
57
58
60
67
52
62
59
61
50
Dalanistes
53
Rayanistes
54
Kutchicetus
56
Andrewsiphius
55
Remingtonocetus
49
48
Ambulocetus
51
Pakicetidae
Hippopotamus
Sus_scrofa
50
45
40
35
30
25
20
15
10
5
0
state 0
state 1

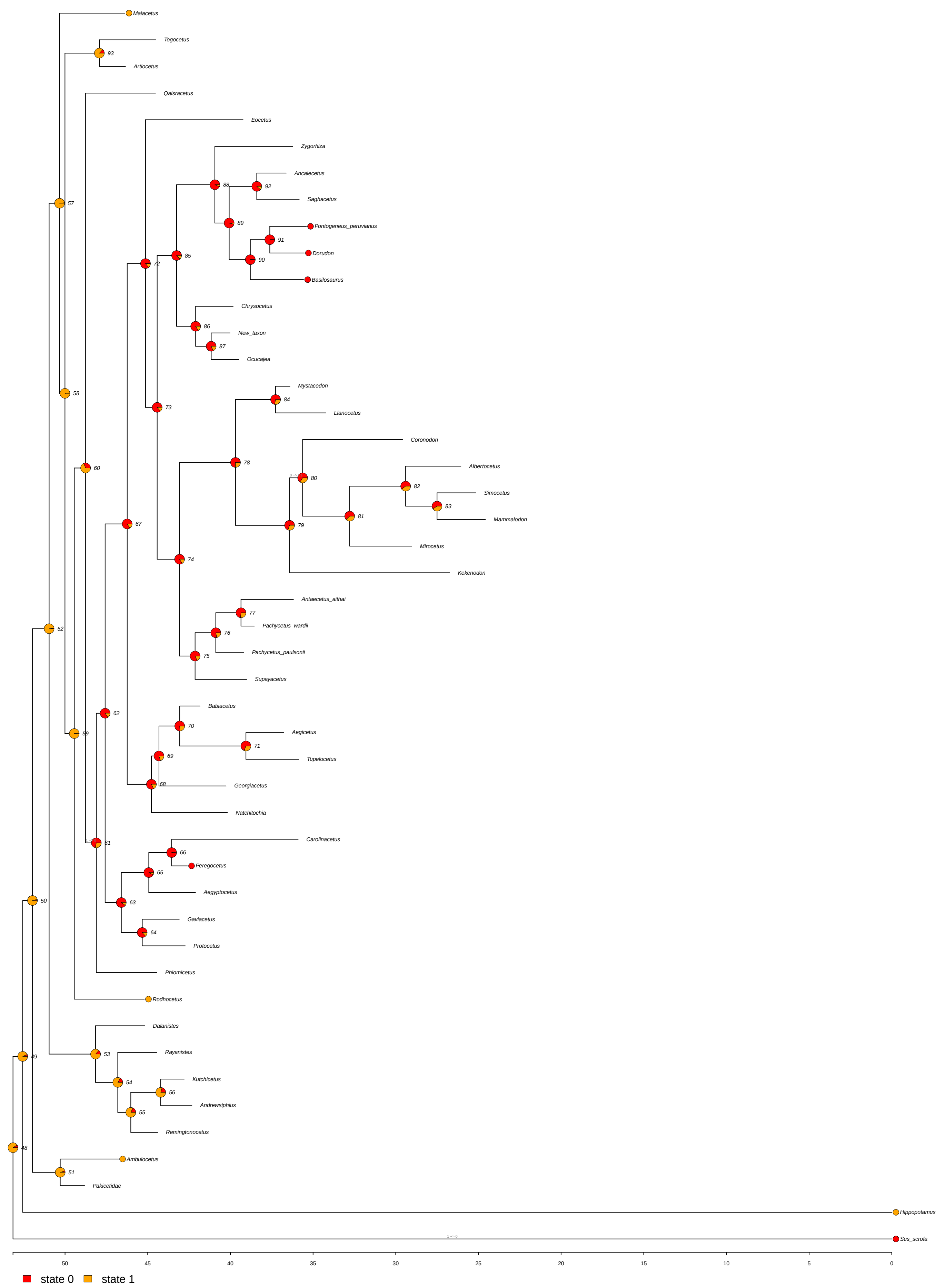

Maiacetus
Togocetus
93
Artiocetus
Qaisracetus
Eocetus
Zygorhiza
Ancalecetus
88
92
Saghacetus
89
Pontogeneus_peruvianus
91
Dorudon
90
Basilosaurus
72
85
Chrysocetus
86
New_taxon
87
Ocucajea
Mystacodon
84
Llanocetus
58
73
Coronodon
78
Albertocetus
80
82
Simocetus
83
Mammalodon
81
79
Mirocetus
Kekenodon
74
Antaecetus_aithai
77
Pachycetus_wardii
76
Pachycetus_paulsonii
75
Supayacetus
Babiacetus
62
70
Aegicetus
71
Tupelocetus
69
68
Georgiacetus
Natchitochia
Carolinacetus
66
Peregocetus
65
Aegyptocetus
63
Gaviacetus
64
Protocetus
Phiomicetus
Rodhocetus
Dalanistes
53
Rayanistes
54
Kutchicetus
56
Andrewsiphius
55
Remingtonocetus
48
Ambulocetus
51
Pakicetidae
Hippopotamus
Sus_scrofa
57
60
67
52
59
61
50
49
50
45
40
35
30
25
20
15
10
5
0
state 0
state 1

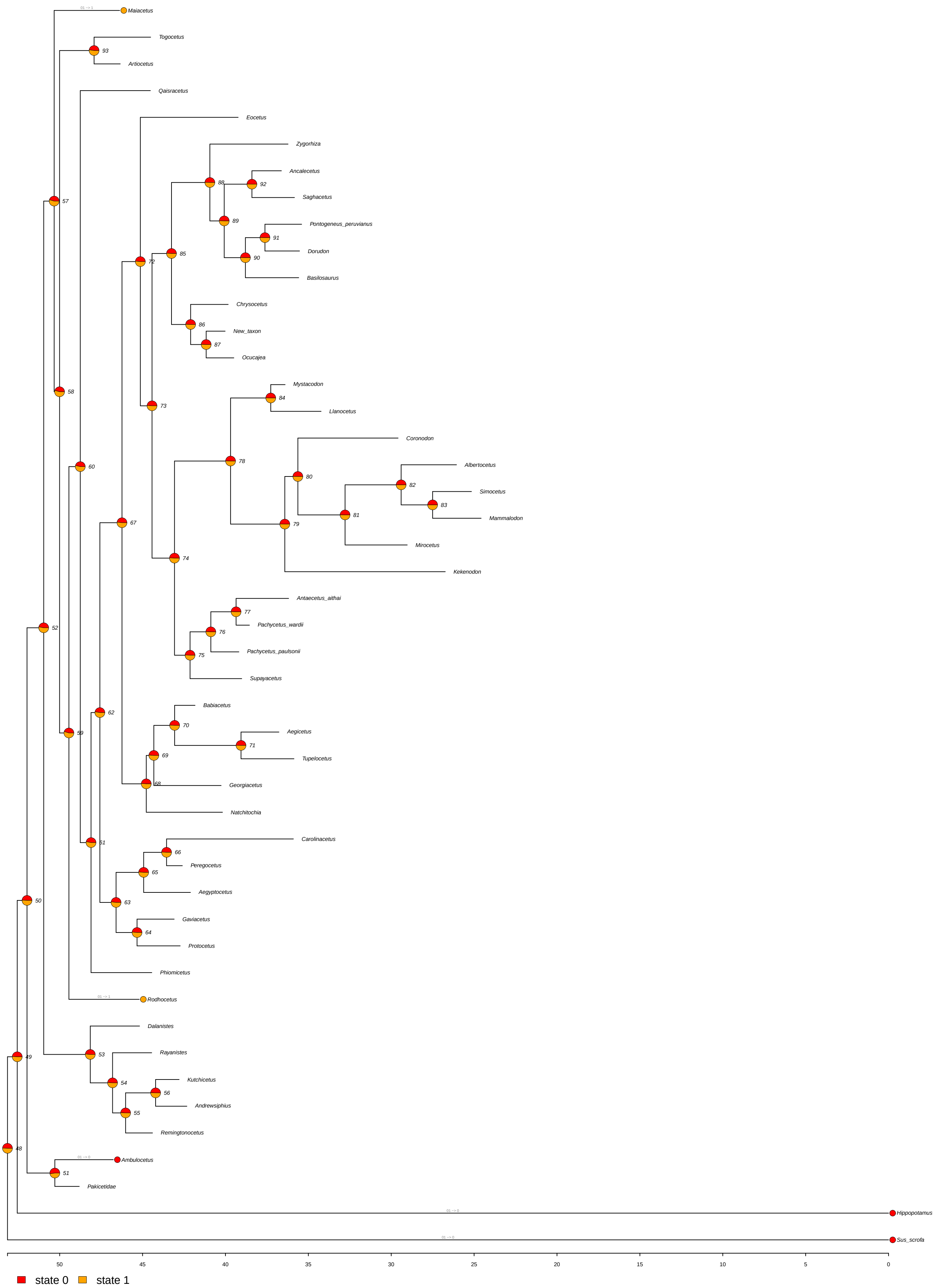

Maiacetus
Togocetus
93
Artiocetus
Qaisracetus
Eocetus
Zygorhiza
Ancalecetus
88
92
Saghacetus
57
Pontogeneus_peruvianus
89
91
Dorudon
72
85
90
Basilosaurus
Chrysocetus
86
New_taxon
87
Ocucajea
Mystacodon
84
58
Llanocetus
73
Coronodon
Albertocetus
60
78
80
82
Simocetus
83
81
Mammalodon
67
79
Mirocetus
74
Kekenodon
Antaecetus_aithai
77
Pachycetus_wardii
76
52
Pachycetus_paulsonii
75
Supayacetus
Babiacetus
62
59
70
Aegicetus
71
69
Tupelocetus
68
Georgiacetus
Natchitochia
61
Carolinacetus
66
Peregocetus
65
Aegyptocetus
50
63
Gaviacetus
64
Protocetus
Phiomicetus
Rodhocetus
Dalanistes
53
Rayanistes
49
54
Kutchicetus
56
Andrewsiphius
55
Remingtonocetus
48
Ambulocetus
51
Pakicetidae
Hippopotamus
Sus_scrofa
50
45
40
35
30
25
20
15
10
5
0
state 0
state 1

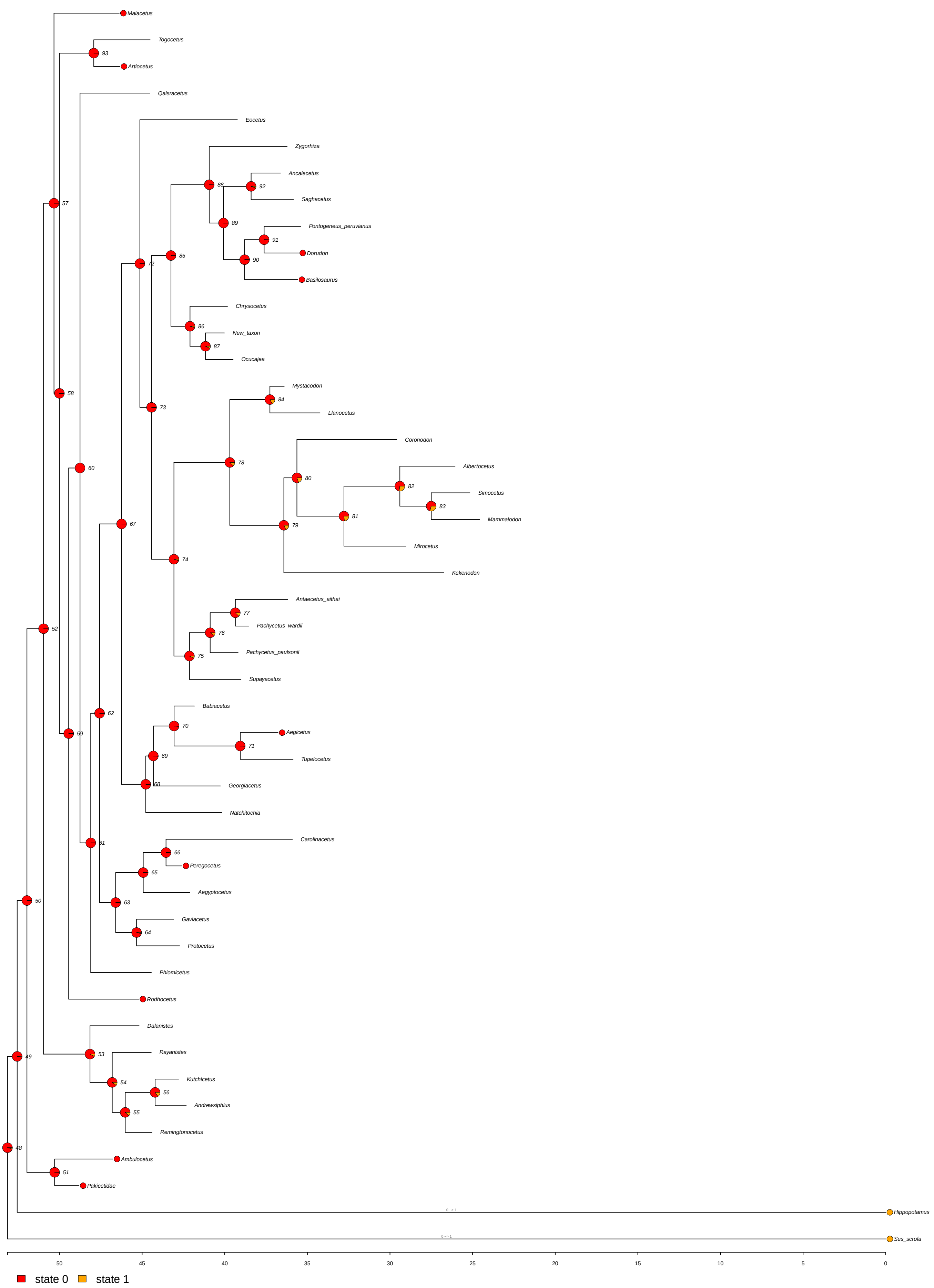

Maiacetus
Togocetus
93
Artiocetus
Qaisracetus
Eocetus
Zygorhiza
Ancalecetus
88
92
Saghacetus
89
Pontogeneus_peruvianus
91
Dorudon
90
Basilosaurus
57
72
85
Chrysocetus
86
New_taxon
87
Ocucajea
Mystacodon
84
Llanocetus
58
73
Coronodon
78
Albertocetus
80
82
Simocetus
83
Mammalodon
60
81
67
79
Mirocetus
74
Kekenodon
Antaecetus_aithai
77
Pachycetus_wardii
76
52
Pachycetus_paulsonii
75
Supayacetus
Babiacetus
62
70
Aegicetus
59
71
Tupelocetus
69
68
Georgiacetus
Natchitochia
Carolinacetus
61
66
Peregocetus
65
Aegyptocetus
50
63
Gaviacetus
64
Protocetus
Phiomicetus
Rodhocetus
Dalanistes
Rayanistes
53
49
54
Kutchicetus
56
Andrewsiphius
55
Remingtonocetus
48
Ambulocetus
51
Pakicetidae
0 --> 1
Hippopotamus
0 --> 1
Sus_scrofa
50
45
40
35
30
25
20
15
10
5
0
state 0
state 1

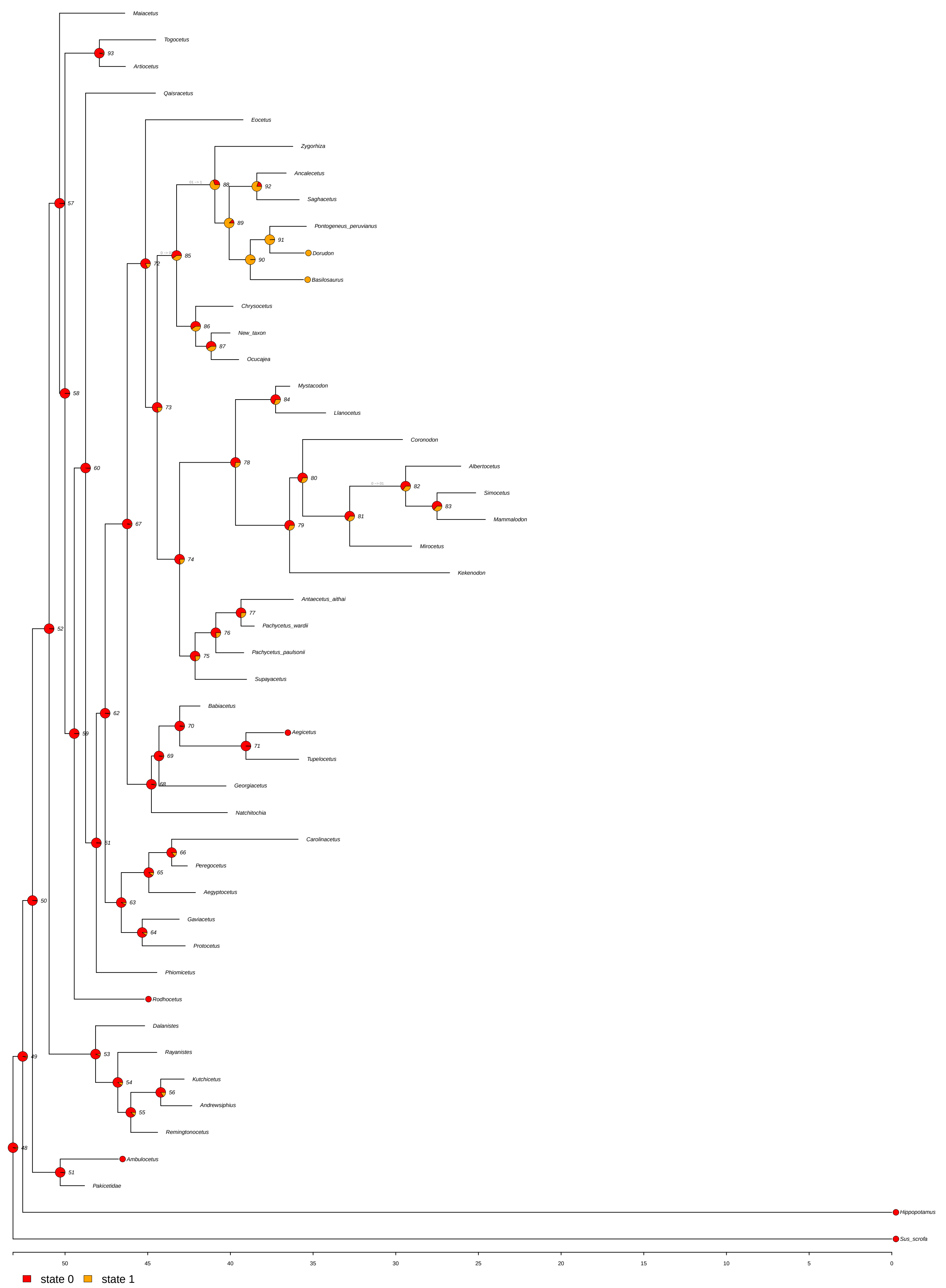
Maiacetus
Togocetus
93
Artiocetus
Qaisracetus
Eocetus
Zygorhiza
Ancalecetus
01 -> 1
88
92
Saghacetus
89
Pontogeneus_peruvianus
91
Dorudon
90
Basilosaurus
0 -> 01
85
72
57
Chrysocetus
86
New_taxon
87
Ocucajea
Mystacodon
84
Llanocetus
58
73
Coronodon
78
Albertocetus
80
0 -> 01
82
Simocetus
60
83
Mammalodon
67
81
79
Mirocetus
74
Kekenodon
Antaecetus_aithai
77
Pachycetus_wardii
76
52
Pachycetus_paulsonii
75
Supayacetus
Babiacetus
62
70
Aegicetus
59
71
Tupelocetus
69
68
Georgiacetus
Natchitochia
Carolinacetus
61
66
Peregocetus
65
Aegyptocetus
50
63
Gaviacetus
64
Protocetus
Phiomicetus
Rodhocetus
Dalanistes
Rayanistes
53
49
54
Kutchicetus
56
Andrewsiphius
55
Remingtonocetus
48
Ambulocetus
51
Pakicetidae
Hippopotamus
Sus_scrofa
50
45
40
35
30
25
20
15
10
5
0
state 0
state 1

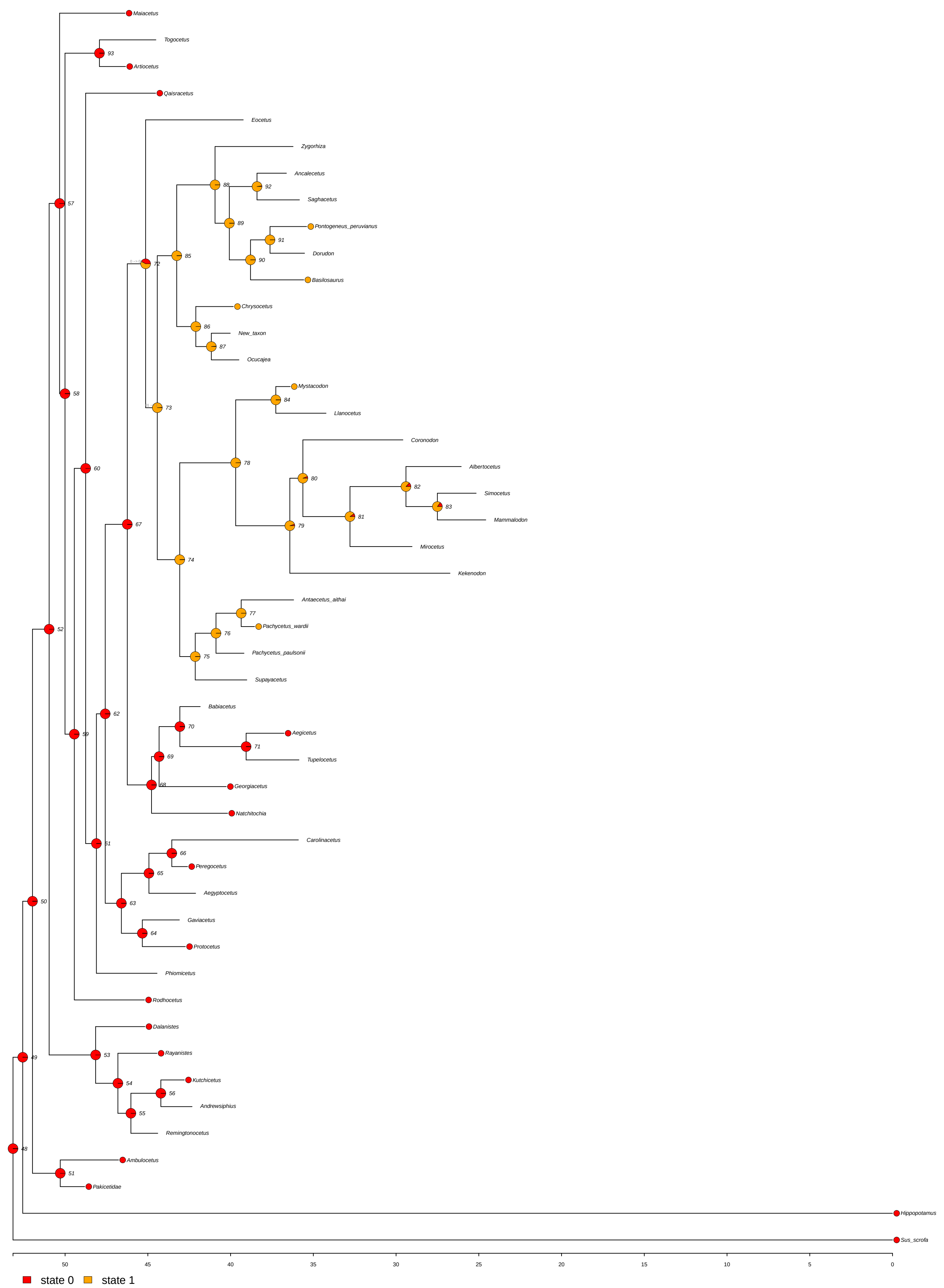
Maiacetus
Togocetus
Artiocetus
Qaisracetus
Eocetus
Zygorhiza
Ancalecetus
Saghacetus
Pontogeneus_peruvianus
Dorudon
Basilosaurus
Chrysocetus
New_taxon
Ocucajea
Mystacodon
Llanocetus
Coronodon
Albertocetus
Simocetus
Mammalodon
Mirocetus
Kekenodon
Antaecetus_aithai
Pachycetus_wardii
Pachycetus_paulsonii
Supayacetus
Babiacetus
Aegicetus
Tupelocetus
Georgiacetus
Natchitochia
Carolinacetus
Peregocetus
Aegyptocetus
Gaviacetus
Protocetus
Phiomicetus
Rodhocetus
Dalanistes
Rayanistes
Kutchicetus
Andrewsiphius
Remingtonocetus
Ambulocetus
Pakicetidae
Hippopotamus
Sus_scrofa
50
45
40
35
30
25
20
15
10
5
0
state 0
state 1

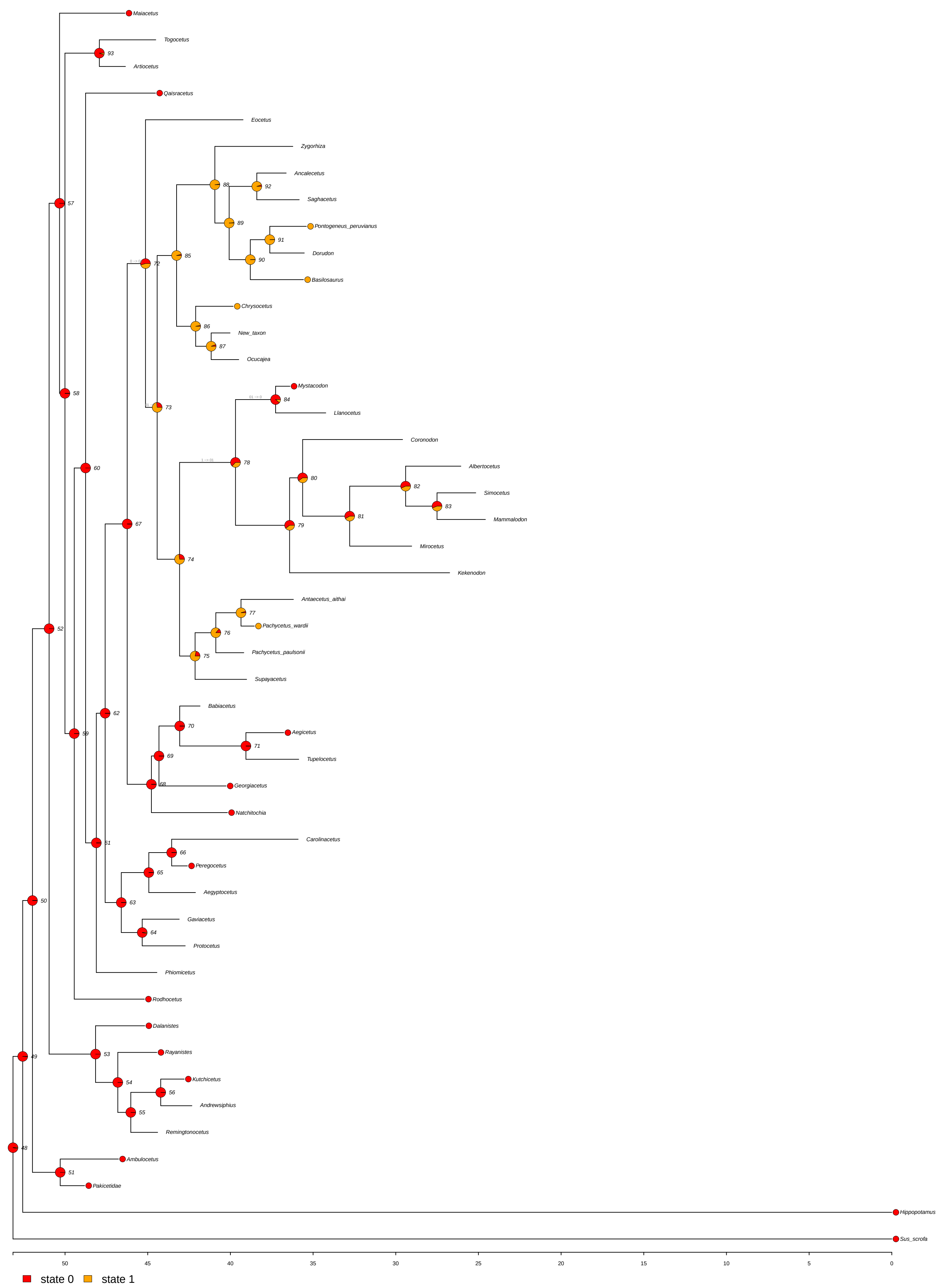

Maiacetus
Togocetus
93
Artiocetus
Qaisracetus
Eocetus
Zygorhiza
Ancalecetus
88
92
Saghacetus
89
Pontogeneus_peruvianus
91
Dorudon
85
90
72
Basilosaurus
Chrysocetus
86
New_taxon
87
Ocucajea
Mystacodon
84
Llanocetus
73
Coronodon
78
Albertocetus
80
82
Simocetus
83
81
Mammalodon
79
Mirocetus
74
Kekenodon
Antaecetus_aithai
77
Pachycetus_wardii
76
Pachycetus_paulsonii
75
Supayacetus
Babiacetus
70
Aegicetus
71
Tupelocetus
69
68
Georgiacetus
Natchitochia
Carolinacetus
66
Peregocetus
65
Aegyptocetus
63
Gaviacetus
64
Protocetus
Phiomicetus
Rodhocetus
Dalanistes
53
Rayanistes
54
Kutchicetus
56
55
Andrewsiphius
Remingtonocetus
Ambulocetus
51
Pakicetidae
Hippopotamus
Sus_scrofa
57
58
60
67
52
62
59
61
50
49
48
50
45
40
35
30
25
20
15
10
5
0
state 0
state 1

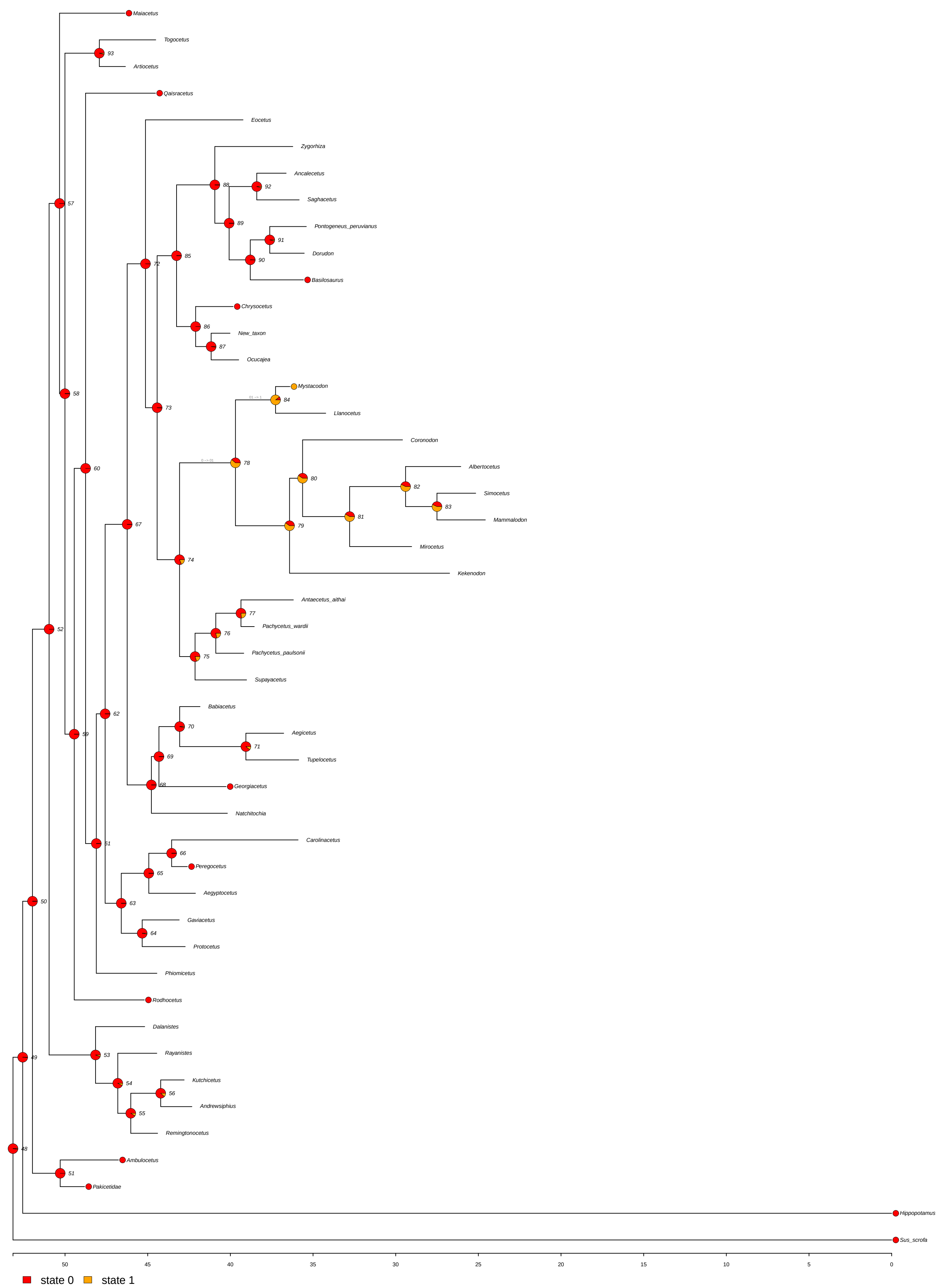
Maiacetus
Togocetus
93
Artiocetus
Qaisracetus
Eocetus
Zygorhiza
Ancalecetus
88
92
Saghacetus
Pontogeneus_peruvianus
89
91
Dorudon
90
Basilosaurus
57
72
85
Chrysocetus
New_taxon
86
87
Ocucajea
Mystacodon
01 --> 1
84
Llanocetus
58
73
Coronodon
0 --> 01
78
60
80
Albertocetus
82
Simocetus
83
Mammalodon
81
67
79
Mirocetus
74
Kekenodon
Antaecetus_aithai
77
Pachycetus_wardii
76
52
Pachycetus_paulsonii
75
Supayacetus
Babiacetus
62
59
70
Aegicetus
71
Tupelocetus
69
68
Georgiacetus
Natchitochia
Carolinacetus
61
66
Peregocetus
65
Aegyptocetus
50
63
Gaviacetus
64
Protocetus
Phiomicetus
Rodhocetus
Dalanistes
53
Rayanistes
49
54
Kutchicetus
56
Andrewsiphius
55
Remingtonocetus
48
Ambulocetus
51
Pakicetidae
Hippopotamus
Sus_scrofa
50
45
40
35
30
25
20
15
10
5
0
state 0
state 1

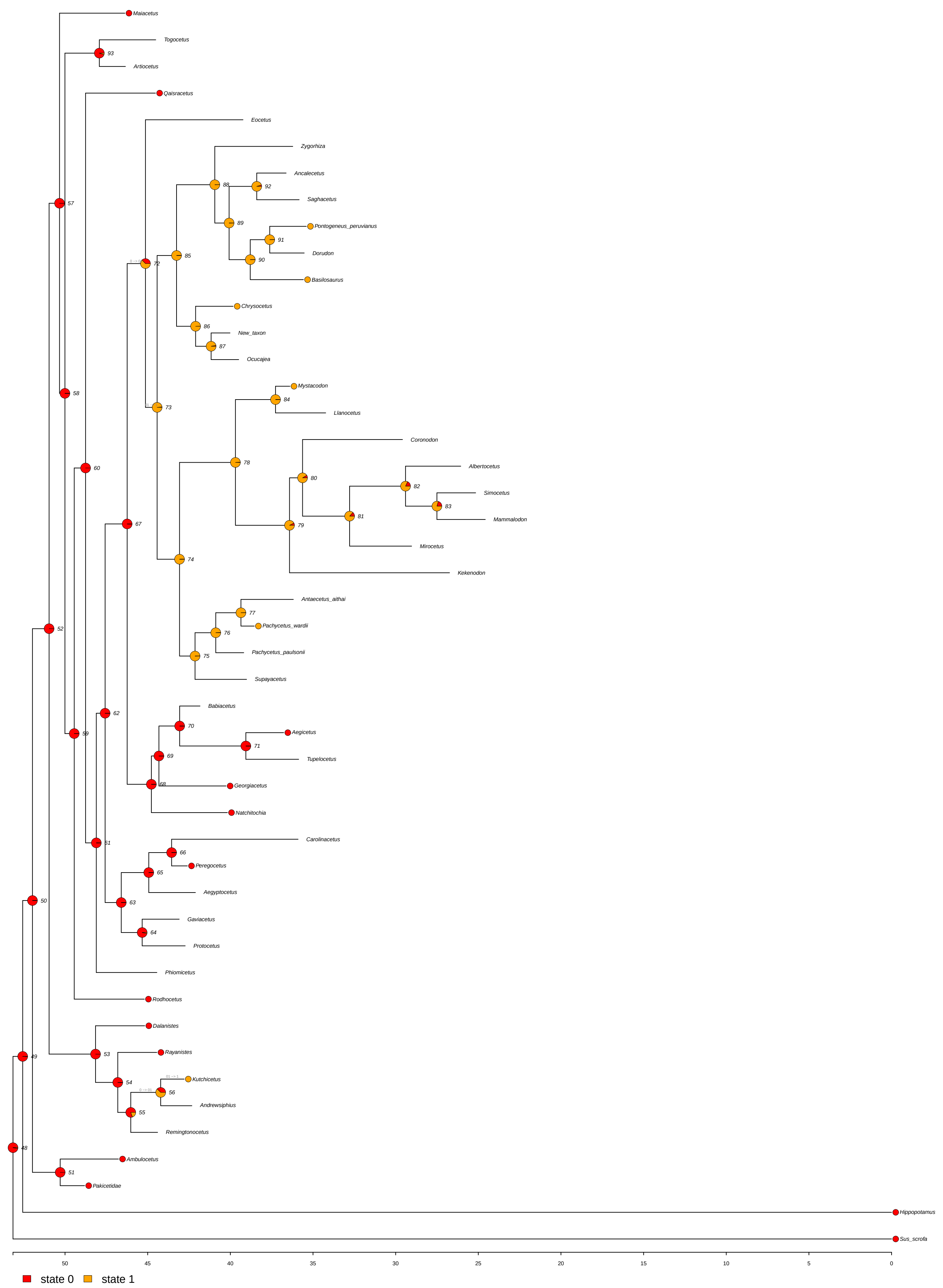

Maiacetus
Togocetus
93
Artiocetus
Qaisracetus
Eocetus
Zygorhiza
Ancalecetus
88
92
Saghacetus
89
Pontogeneus_peruvianus
91
Dorudon
90
72
85
Basilosaurus
Chrysocetus
86
New_taxon
87
Ocucajea
57
Mystacodon
84
58
Llanocetus
73
Coronodon
78
Albertocetus
60
80
82
Simocetus
83
Mammalodon
67
81
79
Mirocetus
74
Kekenodon
Antaecetus_aithai
77
52
76
Pachycetus_wardii
Pachycetus_paulsonii
75
Supayacetus
Babiacetus
62
70
Aegicetus
59
71
Tupelocetus
69
68
Georgiacetus
Natchitochia
Carolinacetus
61
66
Peregocetus
65
50
Aegyptocetus
63
Gaviacetus
64
Protocetus
Phiomicetus
Rodhocetus
Dalanistes
49
53
Rayanistes
01 -> 1
Kutchicetus
54
0 -> 01
56
Andrewsiphius
55
Remingtonocetus
48
Ambulocetus
51
Pakicetidae
Hippopotamus
Sus_scrofa
50
45
40
35
30
25
20
15
10
5
0
state 0
state 1

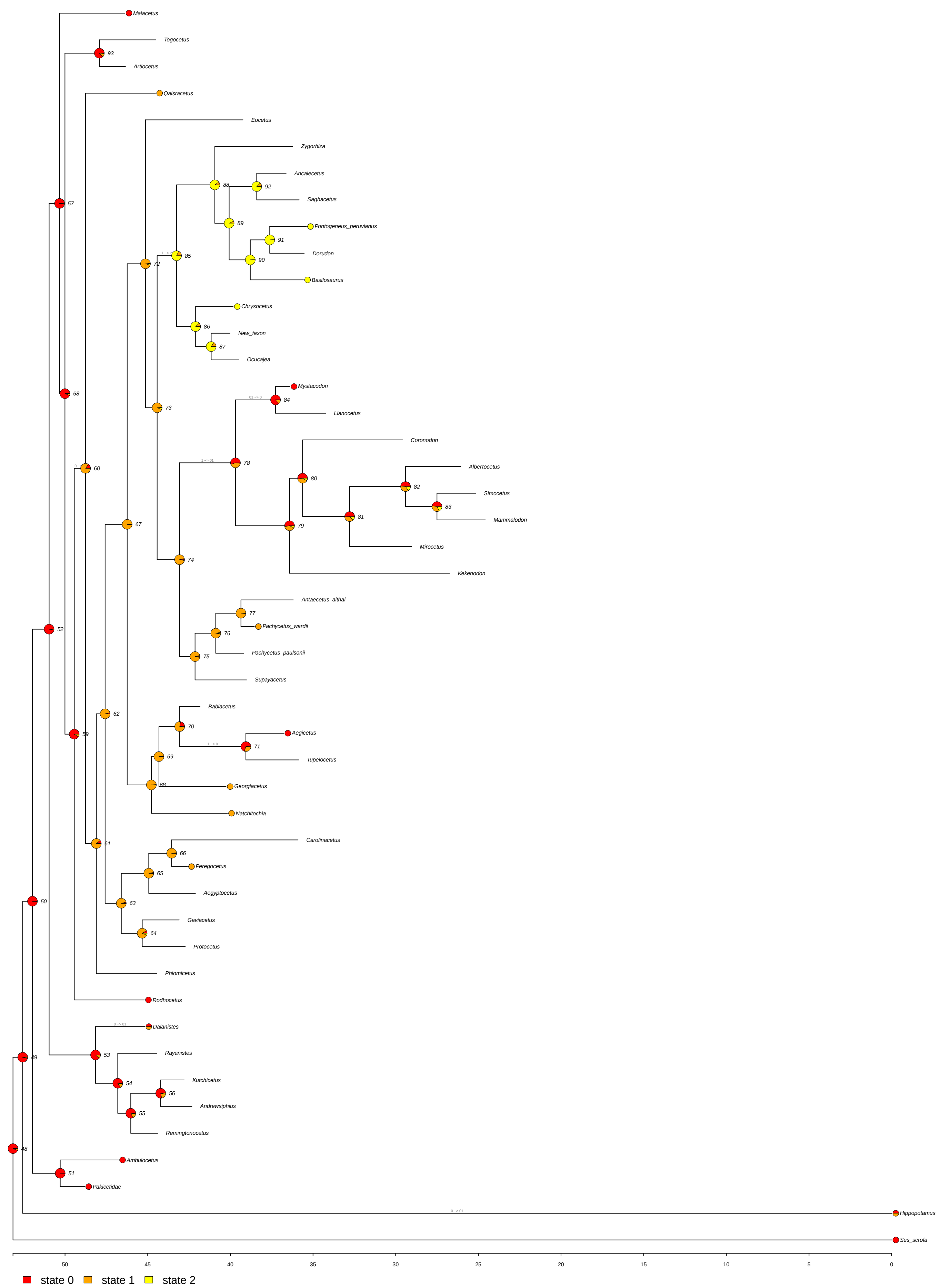

Maiacetus
Togocetus
Artiocetus
Qaisracetus
Eocetus
Zygorhiza
Ancalecetus
Saghacetus
Pontogeneus_peruvianus
Dorudon
Basilosaurus
Chrysocetus
New_taxon
Ocucajea
Mystacodon
Llanocetus
Coronodon
Albertocetus
Simocetus
Mammalodon
Mirocetus
Kekenodon
Antaecetus_aithai
Pachycetus_wardii
Pachycetus_paulsonii
Supayacetus
Babiacetus
Aegicetus
Tupelocetus
Georgiacetus
Natchitochia
Carolinacetus
Peregocetus
Aegyptocetus
Gaviacetus
Protocetus
Phiomicetus
Rodhocetus
Dalanistes
Rayanistes
Kutchicetus
Andrewsiphius
Remingtonocetus
Ambulocetus
Pakicetidae
Hippopotamus
Sus_scrofa
50
45
40
35
30
25
20
15
10
5
0
state 0
state 1
state 2

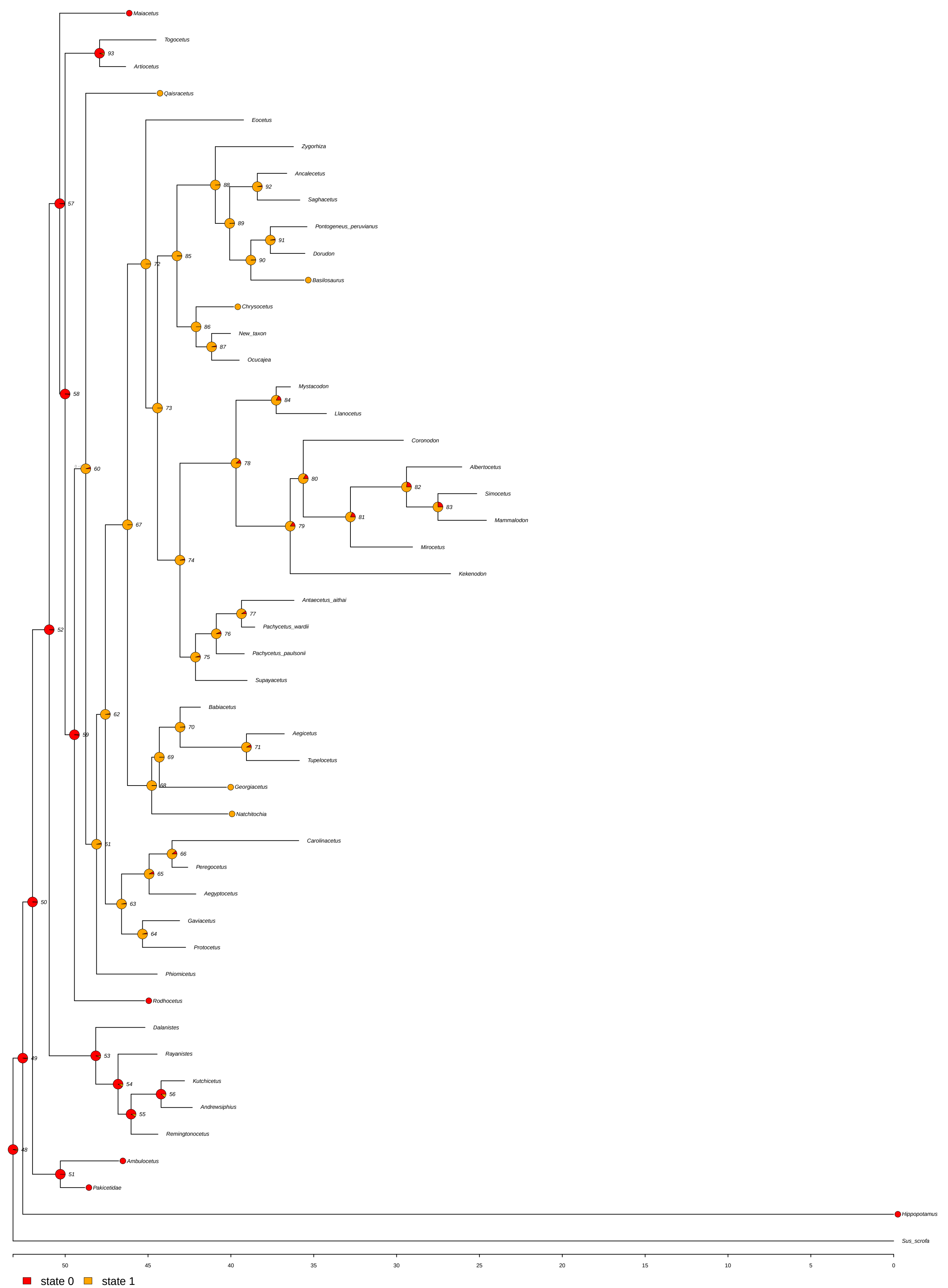

Maiacetus
Togocetus
93
Artiocetus
Qaisracetus
Eocetus
Zygorhiza
Ancalecetus
88
92
Saghacetus
Pontogeneus_peruvianus
89
91
Dorudon
90
Basilosaurus
57
72
85
Chrysocetus
86
New_taxon
87
Ocucajea
Mystacodon
84
Llanocetus
58
73
Coronodon
78
Albertocetus
80
82
Simocetus
83
Mammalodon
60
67
81
79
Mirocetus
74
Kekenodon
Antaecetus_aithai
77
Pachycetus_wardii
76
52
Pachycetus_paulsonii
75
Supayacetus
Babiacetus
62
70
Aegicetus
59
71
Tupelocetus
69
68
Georgiacetus
Natchitochia
Carolinacetus
61
66
Peregocetus
65
Aegyptocetus
50
63
Gaviacetus
64
Protocetus
Phiomicetus
Rodhocetus
Dalanistes
53
Rayanistes
49
54
Kutchicetus
56
Andrewsiphius
55
Remingtonocetus
48
Ambulocetus
51
Pakicetidae
Hippopotamus
Sus_scrofa
50
45
40
35
30
25
20
15
10
5
0
state 0
state 1

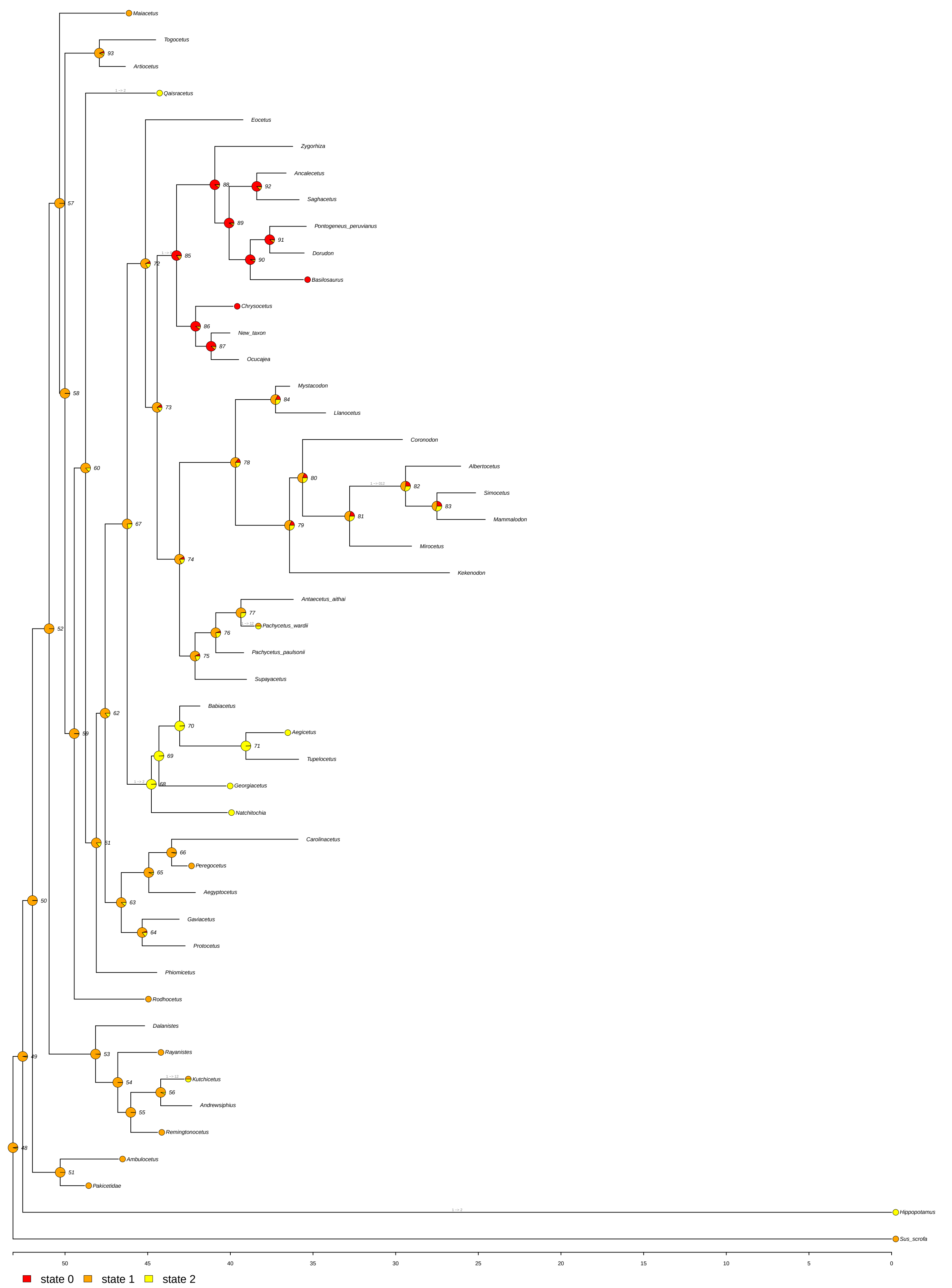

Maiacetus
Togocetus
93
Artiocetus
1 --> 2
Qaisracetus
Eocetus
Zygorhiza
Ancalecetus
88
92
Saghacetus
57
89
Pontogeneus_peruvianus
91
72
85
Dorudon
90
Basilosaurus
Chrysocetus
86
New_taxon
87
Ocucajea
Mystacodon
84
58
73
Llanocetus
Coronodon
60
78
Albertocetus
80
1 --> 012
82
Simocetus
83
Mammalodon
67
79
81
Mirocetus
74
Kekenodon
Antaecetus_aithai
77
52
Pachycetus_wardii
76
75
Pachycetus_paulsonii
Supayacetus
Babiacetus
62
59
70
Aegicetus
71
69
Tupelocetus
1 --> 2
68
Georgiacetus
Natchitochia
Carolinacetus
61
66
Peregocetus
65
50
Aegyptocetus
63
Gaviacetus
64
Protocetus
Phiomicetus
Rodhocetus
Dalanistes
49
53
Rayanistes
54
1 --> 12
Kutchicetus
56
Andrewsiphius
55
Remingtonocetus
48
Ambulocetus
51
Pakicetidae
1 --> 2
Hippopotamus
Sus_scrofa
50
45
40
35
30
25
20
15
10
5
0
state 0
state 1
state 2

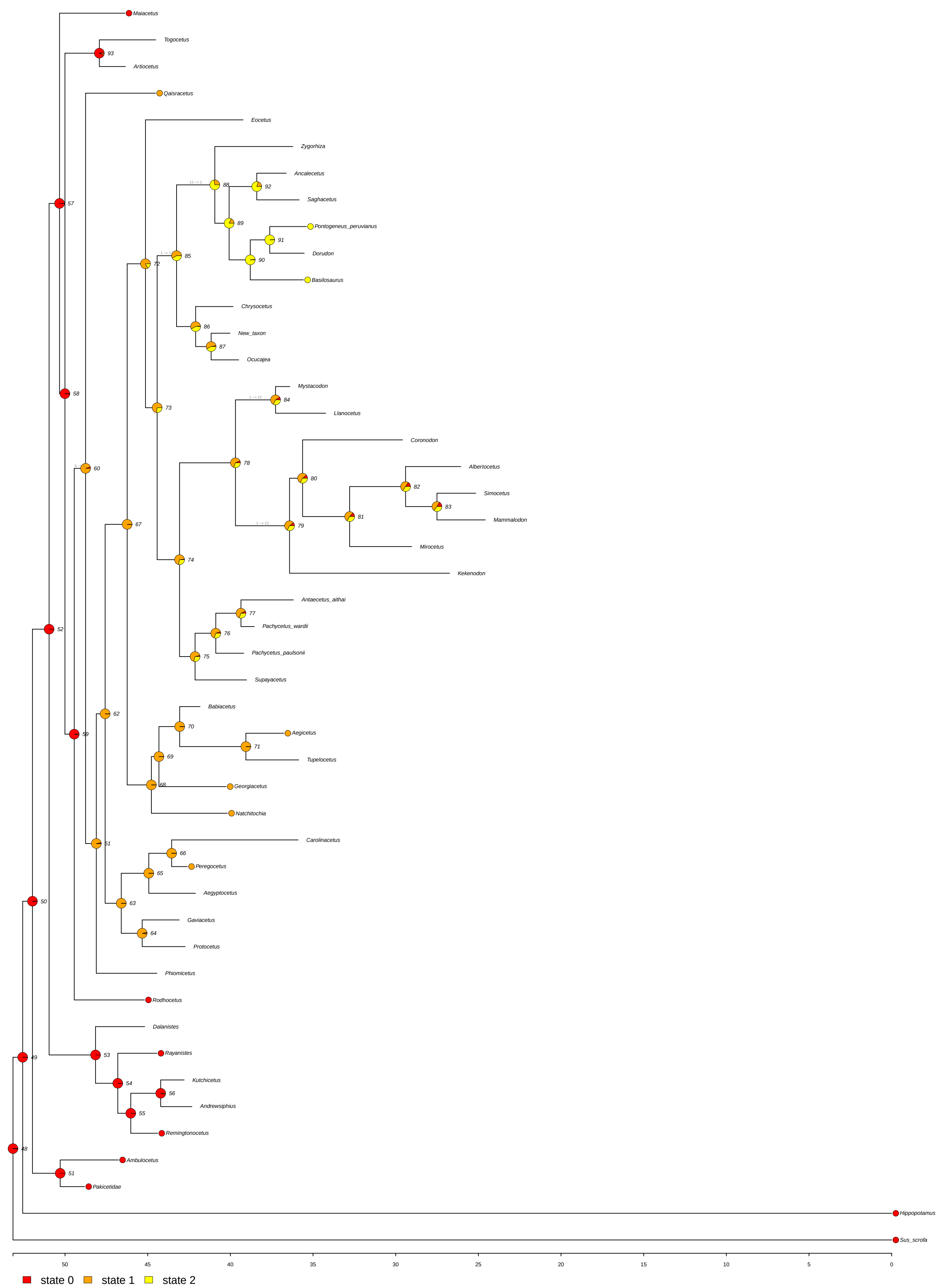

Maiacetus
Togocetus
93
Artiocetus
Qaisracetus
Eocetus
Zygorhiza
Ancalecetus
88
92
Saghacetus
89
Pontogeneus_peruvianus
91
85
72
90
Dorudon
Basilosaurus
Chrysocetus
86
New_taxon
87
Ocucajea
Mystacodon
84
Llanocetus
57
58
73
Coronodon
78
80
Albertocetus
82
Simocetus
83
60
81
Mammalodon
79
67
Mirocetus
74
Kekenodon
Antaecetus_aithai
77
Pachycetus_wardii
76
Pachycetus_paulsonii
75
52
Supayacetus
Babiacetus
62
70
Aegicetus
59
71
Tupelocetus
69
68
Georgiacetus
Natchitochia
Carolinacetus
61
66
Peregocetus
65
Aegyptocetus
50
63
Gaviacetus
64
Protocetus
Phiomicetus
Rodhocetus
Dalanistes
53
Rayanistes
49
54
Kutchicetus
56
Andrewsiphius
55
Remingtonocetus
48
Ambulocetus
51
Pakicetidae
Hippopotamus
Sus_scrofa
50
45
40
35
30
25
20
15
10
5
0
state 0
state 1
state 2

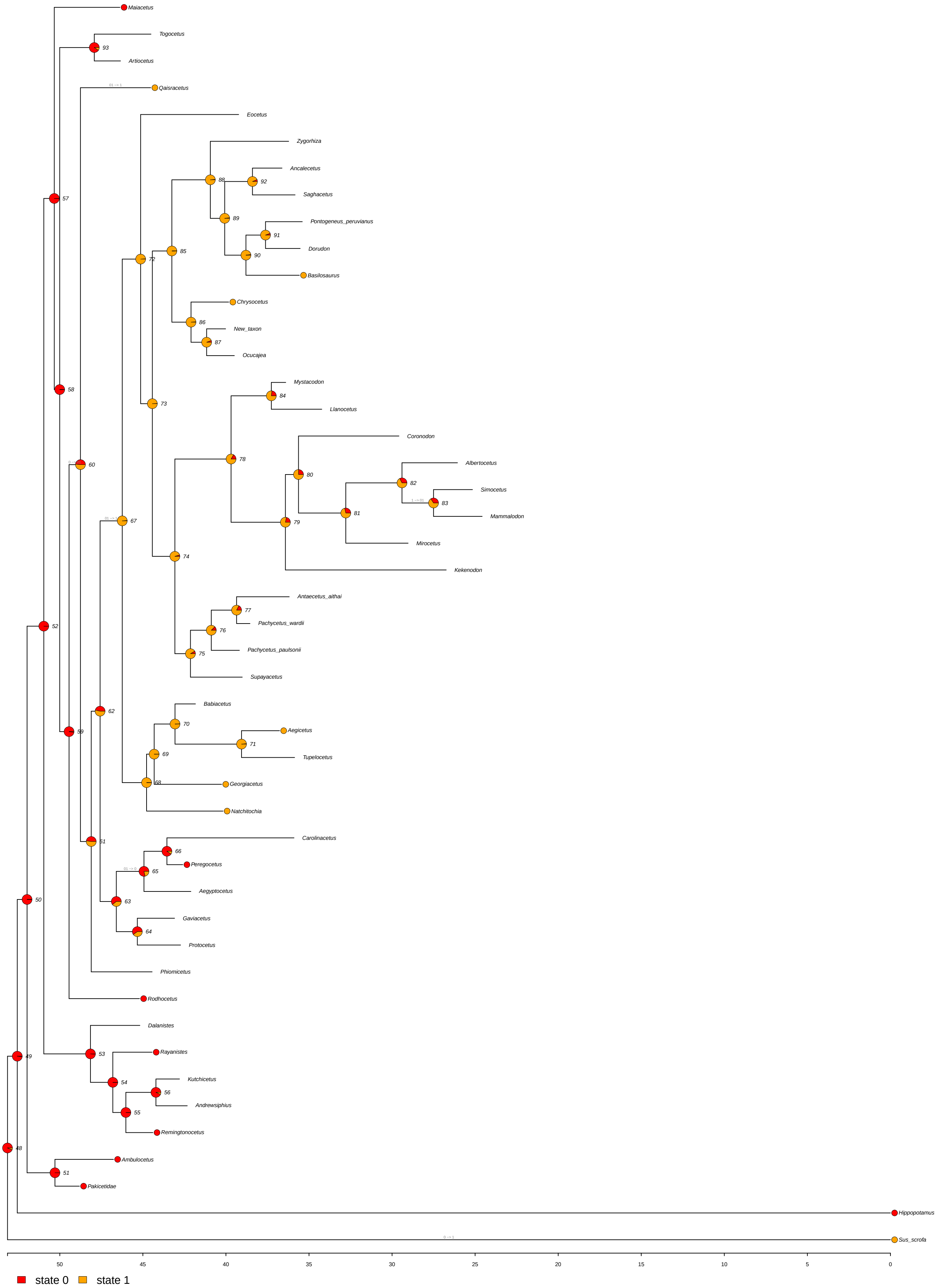

Maiacetus
Togocetus
93
Artiocetus
01 -> 1
Qaisracetus
Eocetus
Zygorhiza
Ancalecetus
88
92
Saghacetus
89
Pontogeneus_peruvianus
91
Dorudon
90
Basilosaurus
72
85
Chrysocetus
86
New_taxon
87
Ocucajea
57
Mystacodon
84
Llanocetus
58
73
Coronodon
78
80
Albertocetus
82
Simocetus
1 -> 01
83
Mammalodon
60
81
79
Mirocetus
67
74
Kekenodon
Antaecetus_aithai
77
Pachycetus_wardii
76
Pachycetus_paulsonii
75
52
Supayacetus
Babiacetus
62
70
59
Aegicetus
71
Tupelocetus
69
68
Georgiacetus
Natchitochia
Carolinacetus
61
66
Peregocetus
01 -> 0
65
Aegyptocetus
50
63
Gaviacetus
64
Protocetus
Phiomicetus
Rodhocetus
Dalanistes
53
Rayanistes
49
54
Kutchicetus
56
Andrewsiphius
55
Remingtonocetus
48
Ambulocetus
51
Pakicetidae
Hippopotamus
0 -> 1
Sus_scrofa
50
45
40
35
30
25
20
15
10
5
0
state 0
state 1

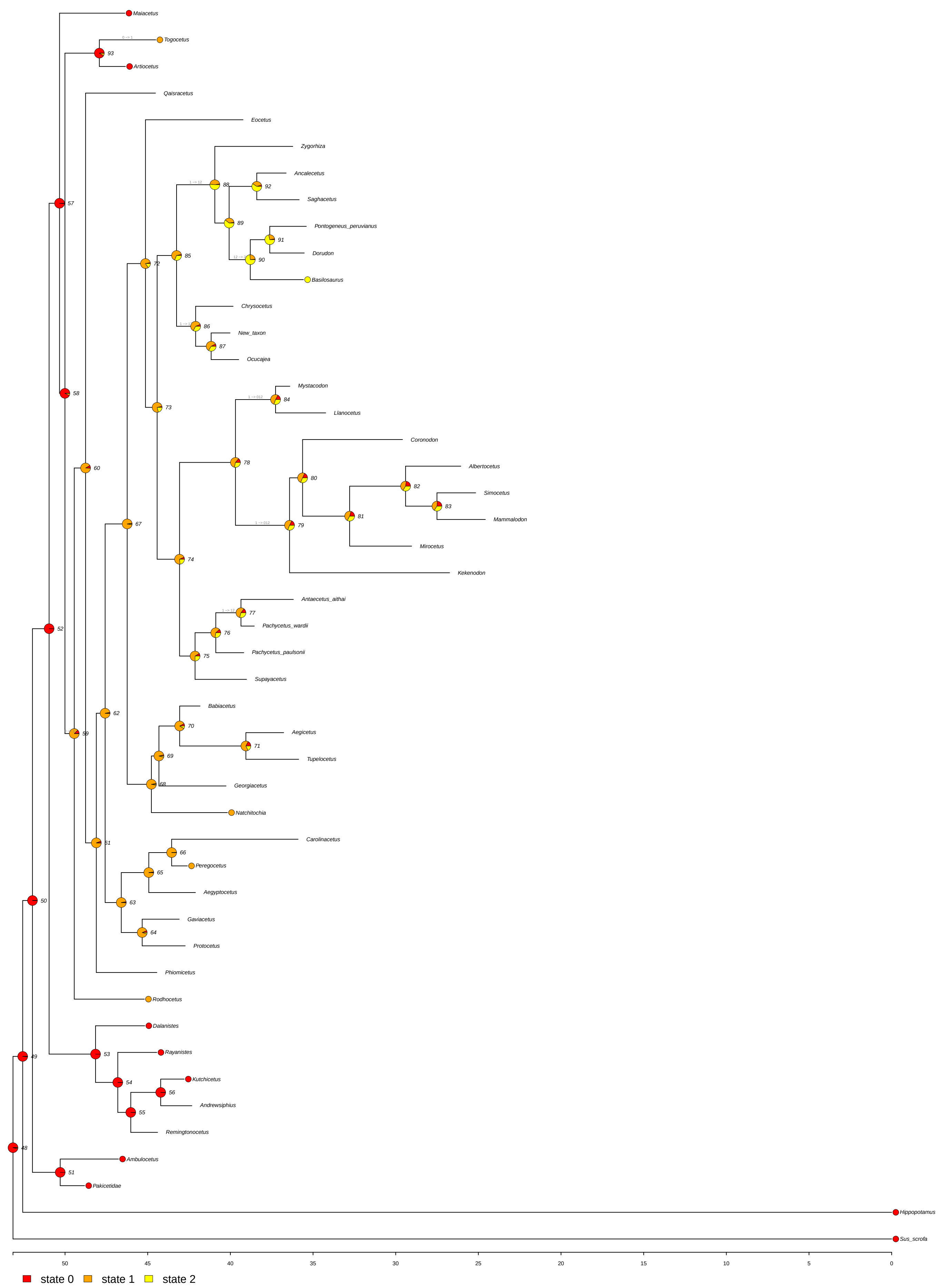

Maiacetus
Togocetus
Artiocetus
Qaisracetus
Eocetus
Zygorhiza
Ancalecetus
Saghacetus
Pontogeneus_peruvianus
Dorudon
Basilosaurus
Chrysocetus
New_taxon
Ocucajea
Mystacodon
Llanocetus
Coronodon
Albertocetus
Simocetus
Mammalodon
Mirocetus
Kekenodon
Antaecetus_aithai
Pachycetus_wardii
Pachycetus_paulsonii
Supayacetus
Babiacetus
Aegicetus
Tupelocetus
Georgiacetus
Natchitochia
Carolinacetus
Peregocetus
Aegyptocetus
Gaviacetus
Protocetus
Phiomicetus
Rodhocetus
Dalanistes
Rayanistes
Kutchicetus
Andrewsiphius
Remingtonocetus
Ambulocetus
Pakicetidae
Hippopotamus
Sus_scrofa
50
45
40
35
30
25
20
15
10
5
0
state 0
state 1
state 2

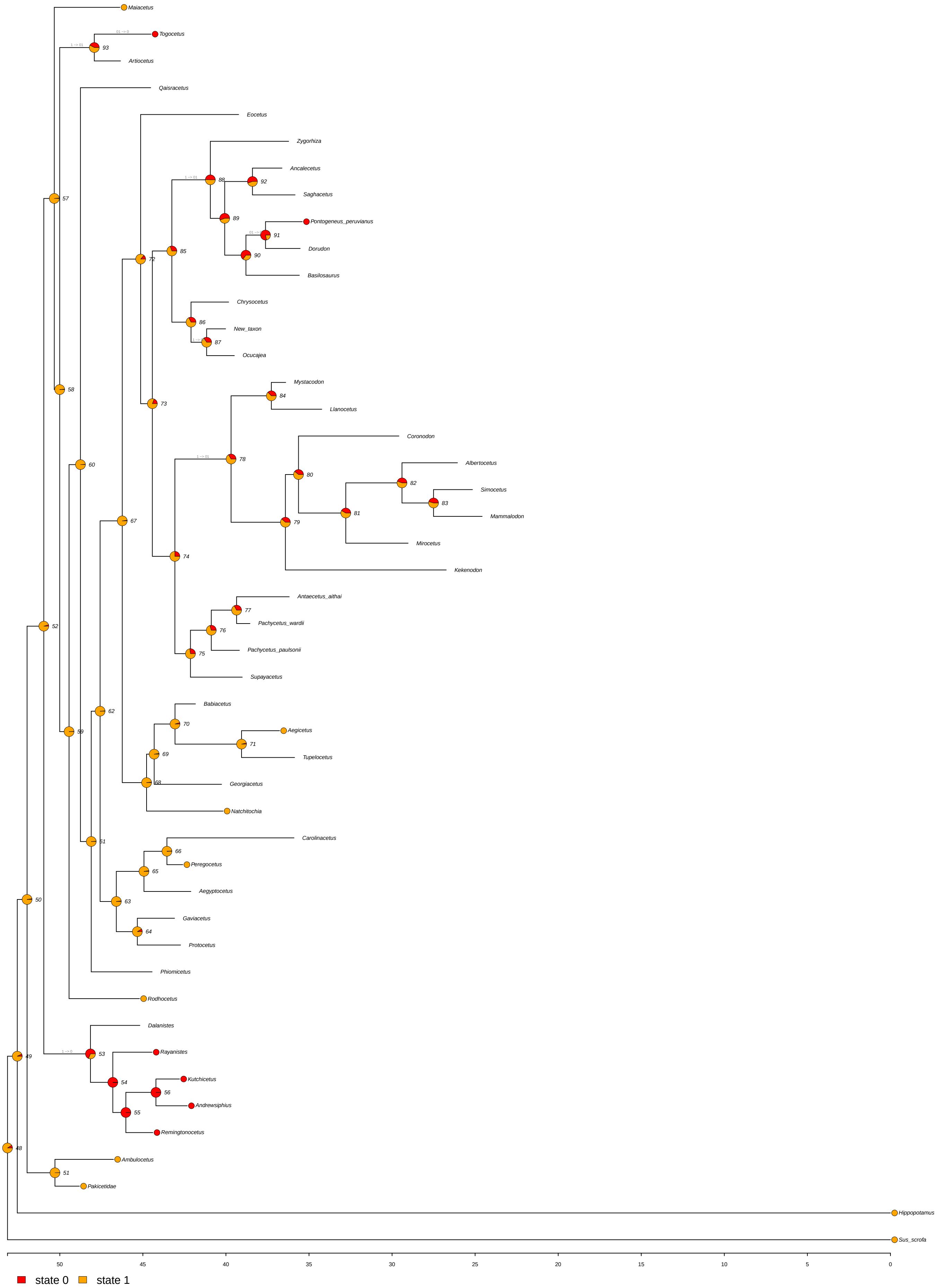
Maiacetus
Togocetus
01 -> 0
1 -> 01
93
Artiocetus
Qaisracetus
Eocetus
Zygorhiza
Ancalecetus
88
92
Saghacetus
89
Pontogeneus_peruvianus
91
Dorudon
90
Basilosaurus
72
85
Chrysocetus
86
New_taxon
87
Ocucajea
57
58
Mystacodon
84
Llanocetus
73
Coronodon
60
78
80
Albertocetus
82
Simocetus
83
Mammalodon
67
79
81
Mirocetus
Kekenodon
74
Antaecetus_aithai
77
52
Pachycetus_wardii
76
Pachycetus_paulsonii
75
Supayacetus
Babiacetus
62
70
Aegicetus
59
71
69
Tupelocetus
68
Georgiacetus
Natchitochia
Carolinacetus
61
66
Peregocetus
65
50
Aegyptocetus
63
Gaviacetus
64
Protocetus
Phiomicetus
Rodhocetus
Dalanistes
1 -> 0
49
53
Rayanistes
54
Kutchicetus
56
Andrewsiphius
55
Remingtonocetus
48
Ambulocetus
51
Pakicetidae
Hippopotamus
Sus_scrofa
50
45
40
35
30
25
20
15
10
5
0
state 0
state 1

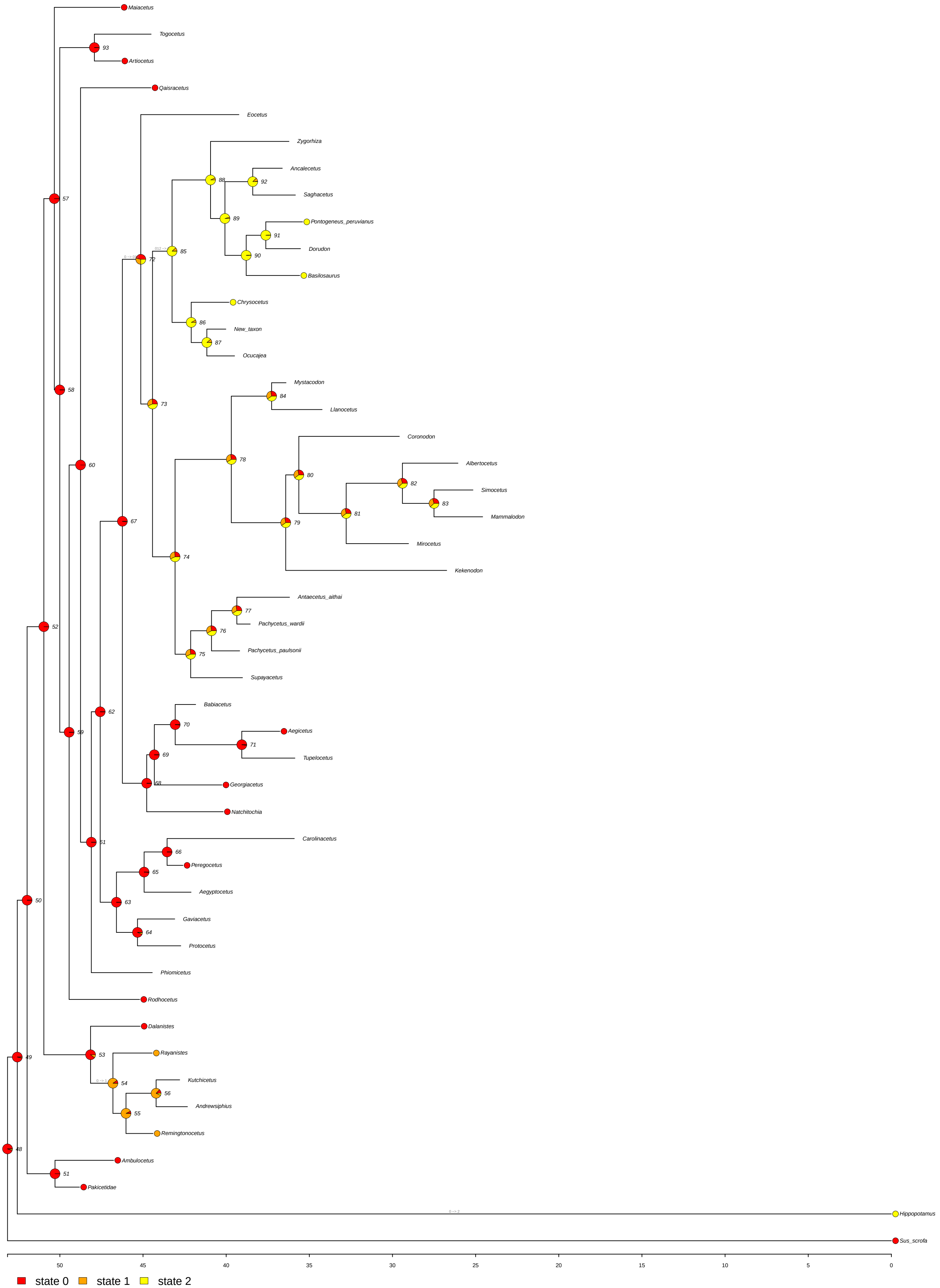
Maiacetus
Togocetus
Artiocetus
Qaisracetus
Eocetus
Zygorhiza
Ancalecetus
Saghacetus
Pontogeneus_peruvianus
Dorudon
Basilosaurus
Chrysocetus
New_taxon
Ocucajea
Mystacodon
Llanocetus
Coronodon
Albertocetus
Simocetus
Mammalodon
Mirocetus
Kekenodon
Antaecetus_aithai
Pachycetus_wardii
Pachycetus_paulsonii
Supayacetus
Babiacetus
Aegicetus
Tupelocetus
Georgiacetus
Natchitochia
Carolinacetus
Peregocetus
Aegyptocetus
Gaviacetus
Protocetus
Phiomicetus
Rodhocetus
Dalanistes
Rayanistes
Kutchicetus
Andrewsiphius
Remingtonocetus
Ambulocetus
Pakicetidae
Hippopotamus
Sus_scrofa
50
45
40
35
30
25
20
15
10
5
0
state 0
state 1
state 2

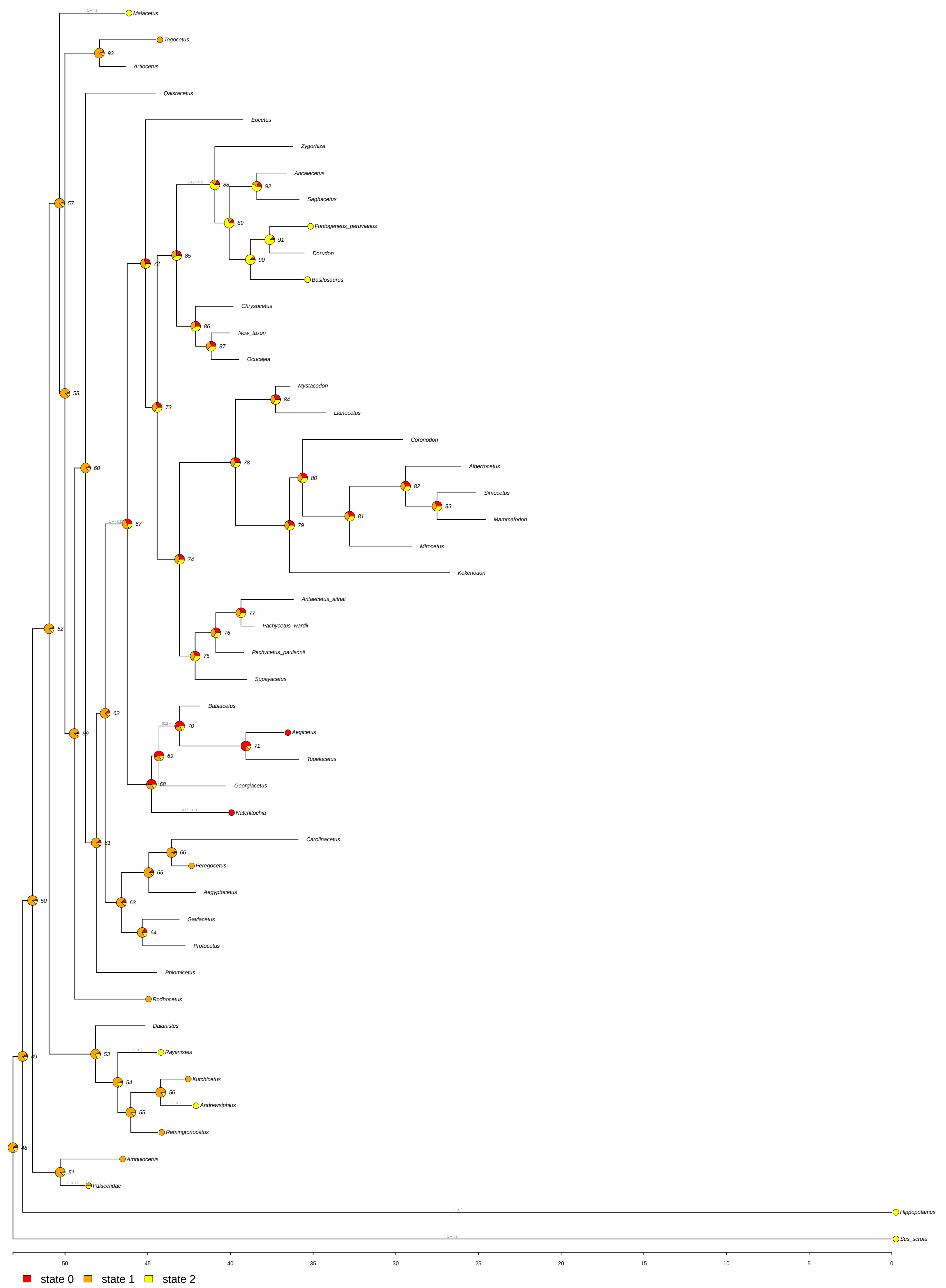
Maiacetus
Togocetus
93
Artiocetus
Qaisracetus
Eocetus
Zygorhiza
Ancalecetus
88
92
Saghacetus
89
Pontogeneus_peruvianus
91
Dorudon
90
Basilosaurus
72
85
Chrysocetus
86
New_taxon
87
Ocucajea
57
58
Mystacodon
84
Llanocetus
73
Coronodon
60
78
Albertocetus
80
82
Simocetus
83
Mammalodon
81
79
67
Mirocetus
74
Kekenodon
Antaecetus_aithai
77
Pachycetus_wardii
52
76
Pachycetus_paulsonii
75
Supayacetus
Babiacetus
62
59
70
Aegicetus
71
Tupelocetus
69
68
Georgiacetus
Natchitochia
Carolinacetus
61
66
Peregocetus
65
Aegyptocetus
50
63
Gaviacetus
64
Protocetus
Phiomicetus
Rodhocetus
Dalanistes
49
53
Rayanistes
54
Kutchicetus
56
55
Andrewsiphius
Remingtonocetus
48
Ambulocetus
51
Pakicetidae
Hippopotamus
Sus_scrofa
50
45
40
35
30
25
20
15
10
5
0
state 0
state 1
state 2

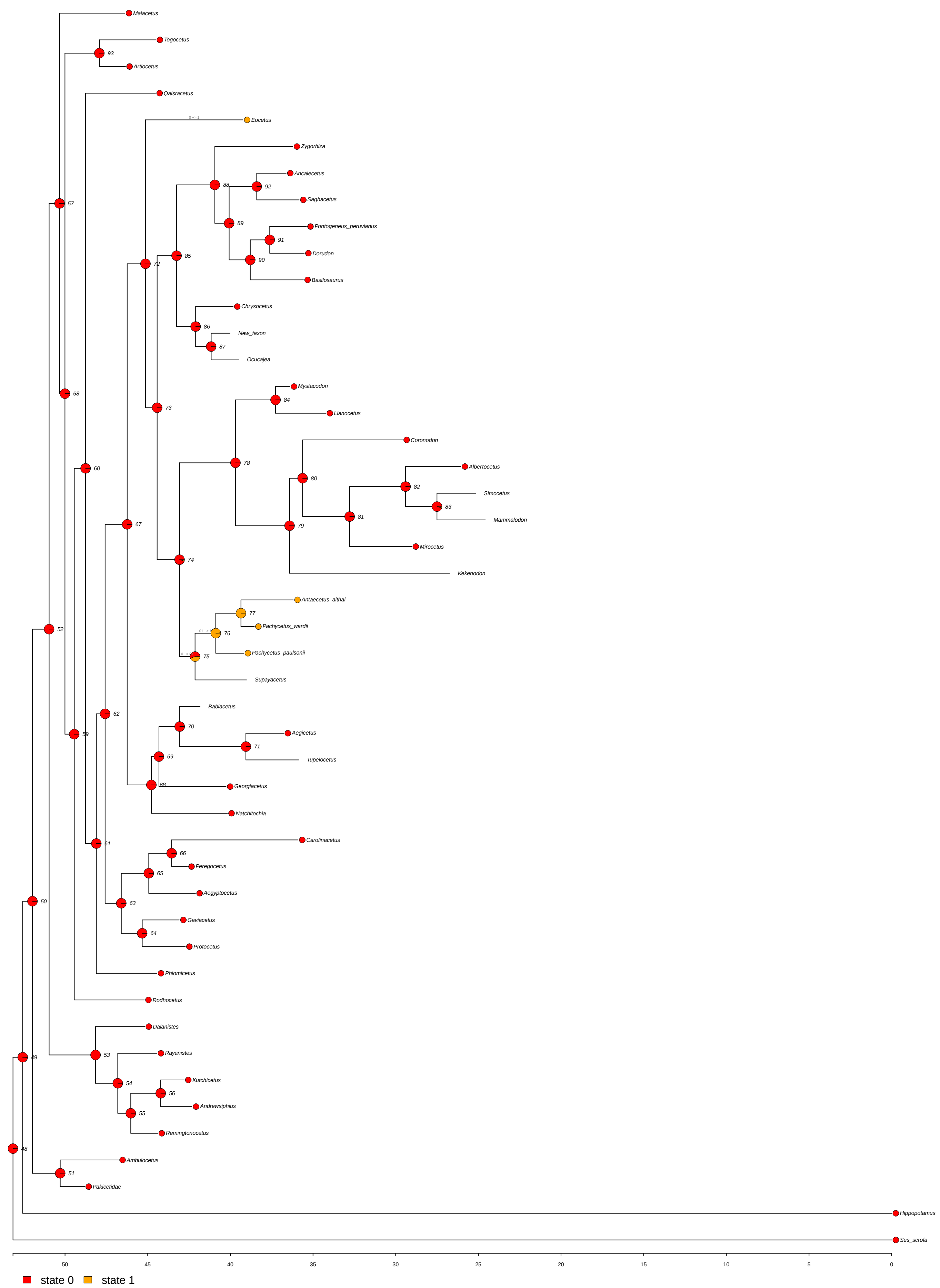

Maiacetus
Togocetus
Artiocetus
Qaisracetus
Eocetus
Zygorhiza
Ancalecetus
Saghacetus
Pontogeneus_peruvianus
Dorudon
Basilosaurus
Chrysocetus
New_taxon
Ocucajea
Mystacodon
Llanocetus
Coronodon
Albertocetus
Simocetus
Mammalodon
Mirocetus
Kekenodon
Antaecetus_aithai
Pachycetus_wardii
Pachycetus_paulsonii
Supayacetus
Babiacetus
Aegicetus
Tupelocetus
Georgiacetus
Natchitochia
Carolinacetus
Peregocetus
Aegyptocetus
Gaviacetus
Protocetus
Phiomicetus
Rodhocetus
Dalanistes
Rayanistes
Kutchicetus
Andrewsiphius
Remingtonocetus
Ambulocetus
Pakicetidae
Hippopotamus
Sus_scrofa
50 45 40 35 30 25 20 15 10 5 0
state 0
state 1

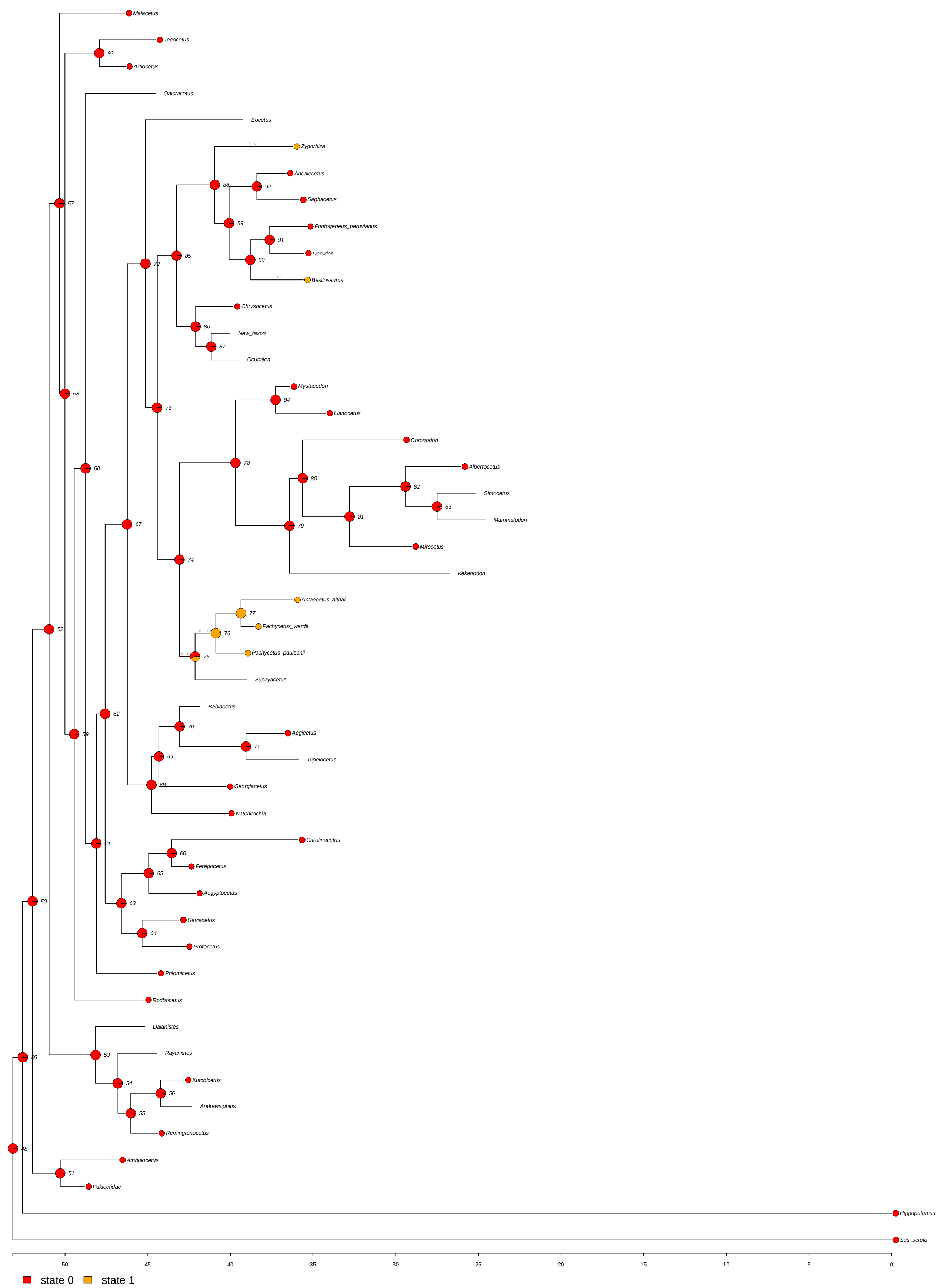
Maiacetus
Togocetus
93
Artiocetus
Qaisracetus
Eocetus
0 --> 1
Zygorhiza
Ancalecetus
88
92
Saghacetus
89
Pontogeneus_peruvianus
91
Dorudon
90
0 --> 1
Basilosaurus
57
72
85
Chrysocetus
86
New_taxon
87
Ocucajea
Mystacodon
84
Llanocetus
58
73
Coronodon
78
80
Albertocetus
60
82
Simocetus
83
67
81
Mammalodon
79
Mirocetus
74
Kekenodon
Antaecetus_aithai
77
Pachycetus_wardii
52
76
Pachycetus_paulsonii
75
Supayacetus
Babiacetus
62
70
Aegicetus
59
71
Tupelocetus
69
68
Georgiacetus
Natchitochia
Carolinacetus
61
66
Peregocetus
65
Aegyptocetus
50
63
Gaviacetus
64
Protocetus
Phiomicetus
Rodhocetus
Dalanistes
53
Rayanistes
49
54
Kutchicetus
56
55
Andrewsiphius
Remingtonocetus
48
Ambulocetus
51
Pakicetidae
Hippopotamus
Sus_scrofa
50
45
40
35
30
25
20
15
10
5
0
state 0
state 1

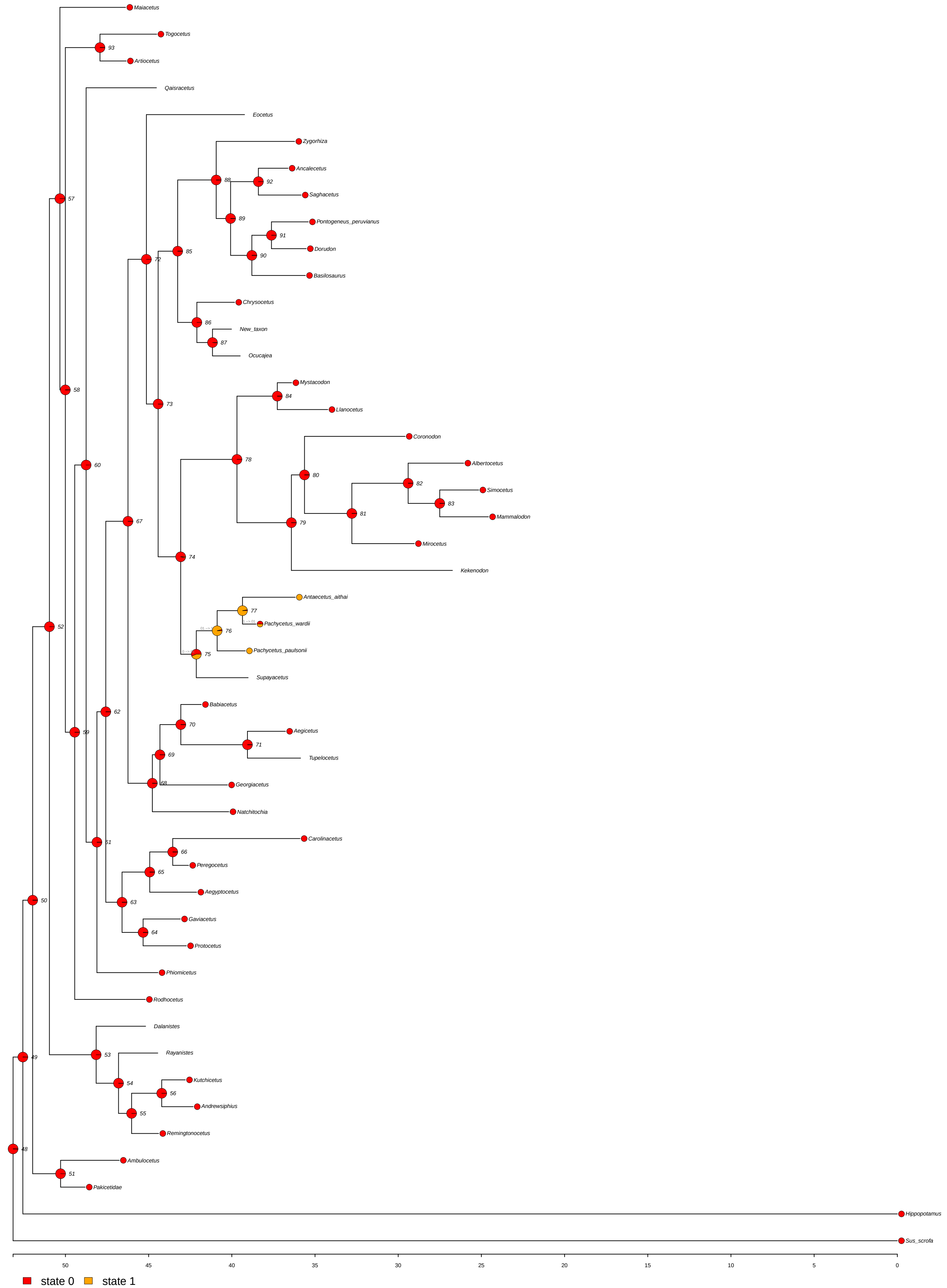
Maiacetus
Togocetus
93
Artiocetus
Qaisracetus
Eocetus
Zygorhiza
Ancalecetus
88
92
Saghacetus
89
Pontogeneus_peruvianus
91
Dorudon
90
Basilosaurus
57
72
85
Chrysocetus
86
New_taxon
87
Ocucajea
Mystacodon
84
58
73
Llanocetus
Coronodon
60
78
Albertocetus
80
82
Simocetus
83
Mammalodon
67
79
81
Mirocetus
74
Kekenodon
Antaecetus_aithai
77
Pachycetus_wardii
52
76
75
Pachycetus_paulsonii
Supayacetus
Babiacetus
62
70
Aegicetus
59
71
69
Tupelocetus
68
Georgiacetus
Natchitochia
Carolinacetus
61
66
Peregocetus
65
Aegyptocetus
50
63
Gaviacetus
64
Protocetus
Phiomicetus
Rodhocetus
Dalanistes
Rayanistes
53
49
Kutchicetus
54
56
55
Andrewsiphius
Remingtonocetus
48
Ambulocetus
51
Pakicetidae
Hippopotamus
Sus_scrofa
50
45
40
35
30
25
20
15
10
5
0
state 0
state 1
