# Supplementary figures and images for: A diminutive new basilosaurid whale reveals the trajectory of the cetacean life histories during the Eocene
Source: Commun Biol. 2023 Aug 10;6:707. doi: 10.1038/s42003-023-04986-w (PMC10415296; doi:10.1038/s42003-023-04986-w)

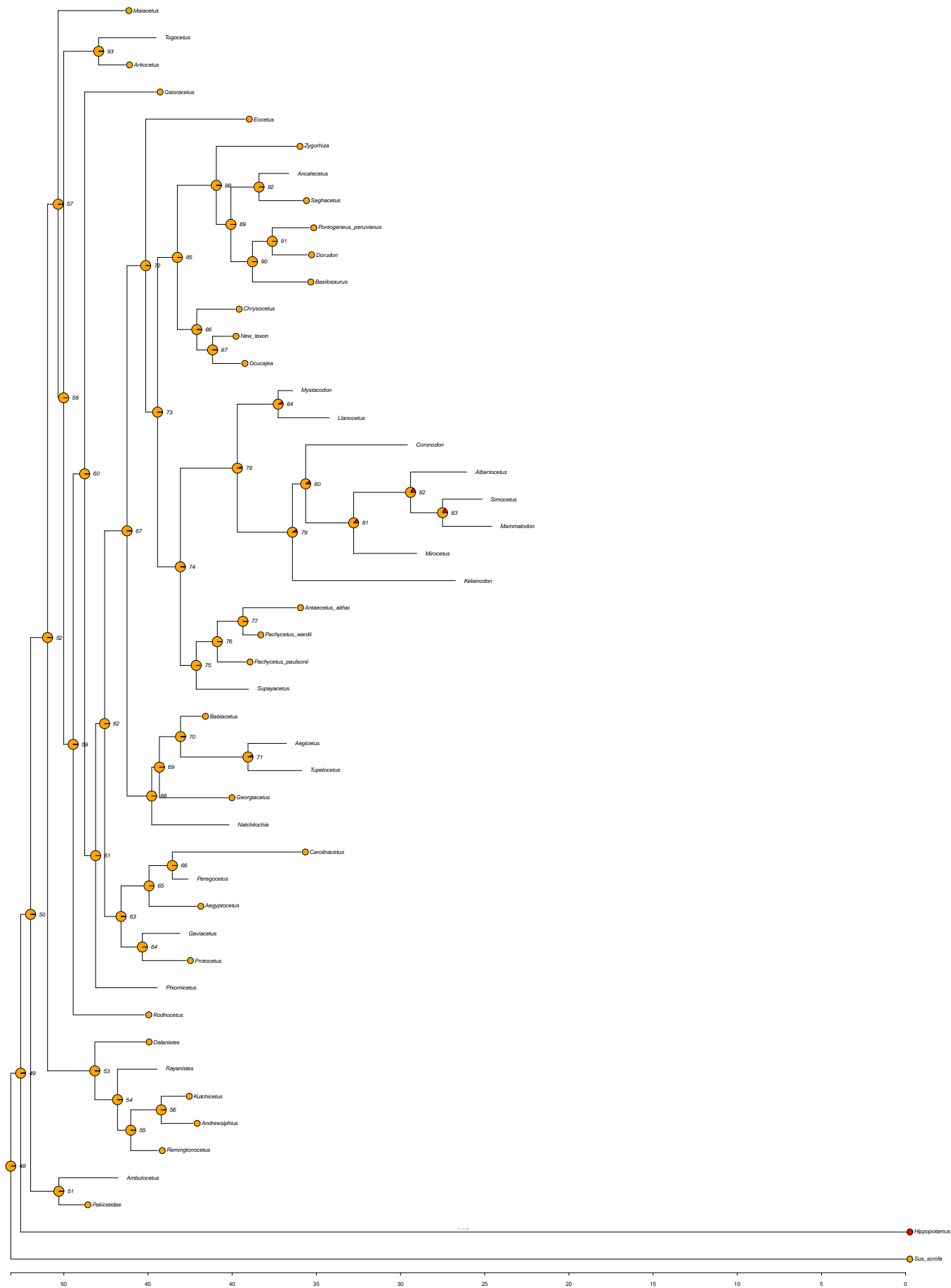

Supplement: Supplementary file 6 — Supplementary Data 3 [file 42003_2023_4986_MOESM6_ESM.zip › Supplementary Data 3/Supplementary Data 1_BTD_ASR/trait_0001_tree.plot.pdf]

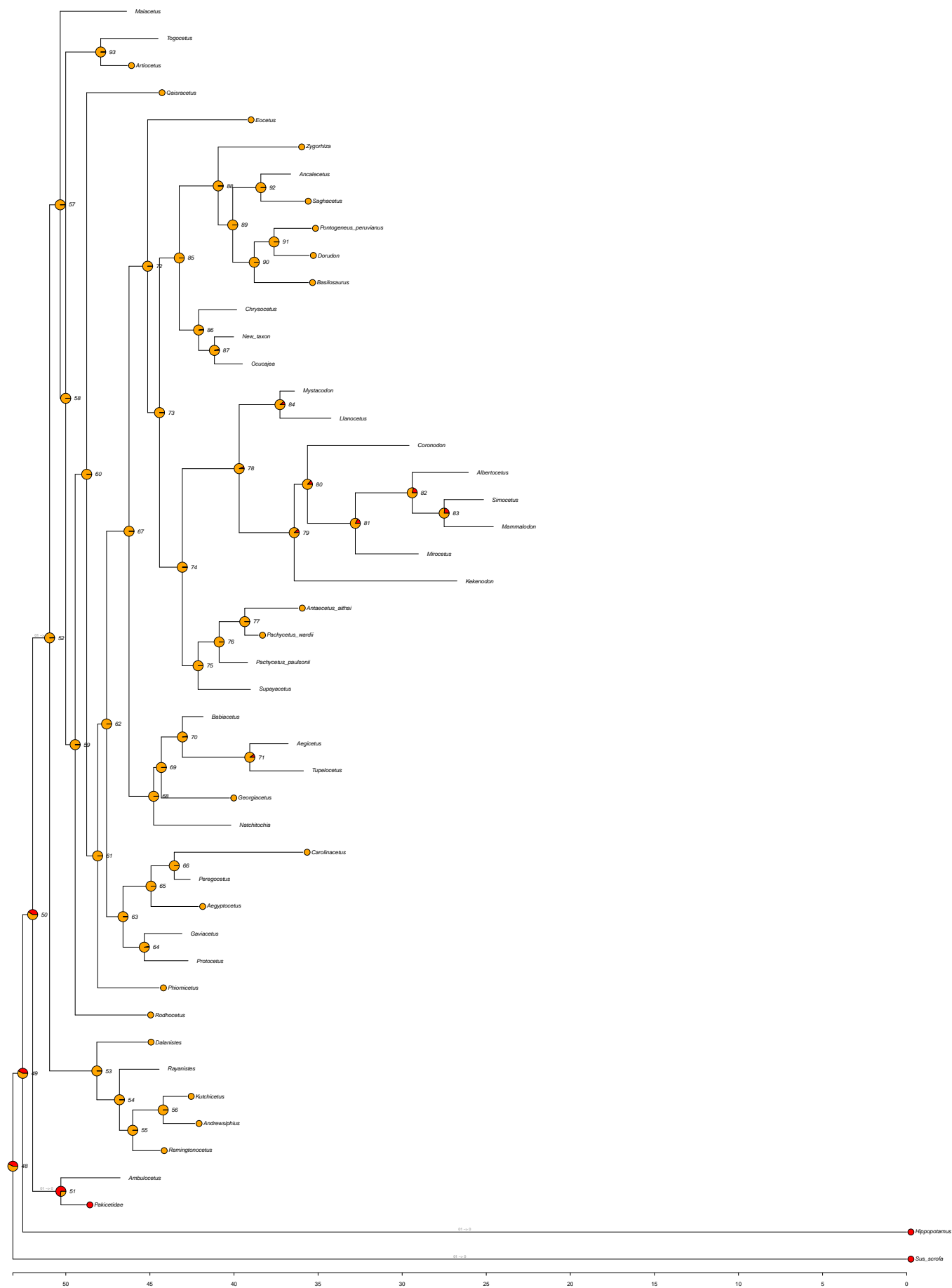

Supplement: Supplementary file 6 — Supplementary Data 3 [file 42003_2023_4986_MOESM6_ESM.zip › Supplementary Data 3/Supplementary Data 1_BTD_ASR/trait_0002_tree.plot.pdf]

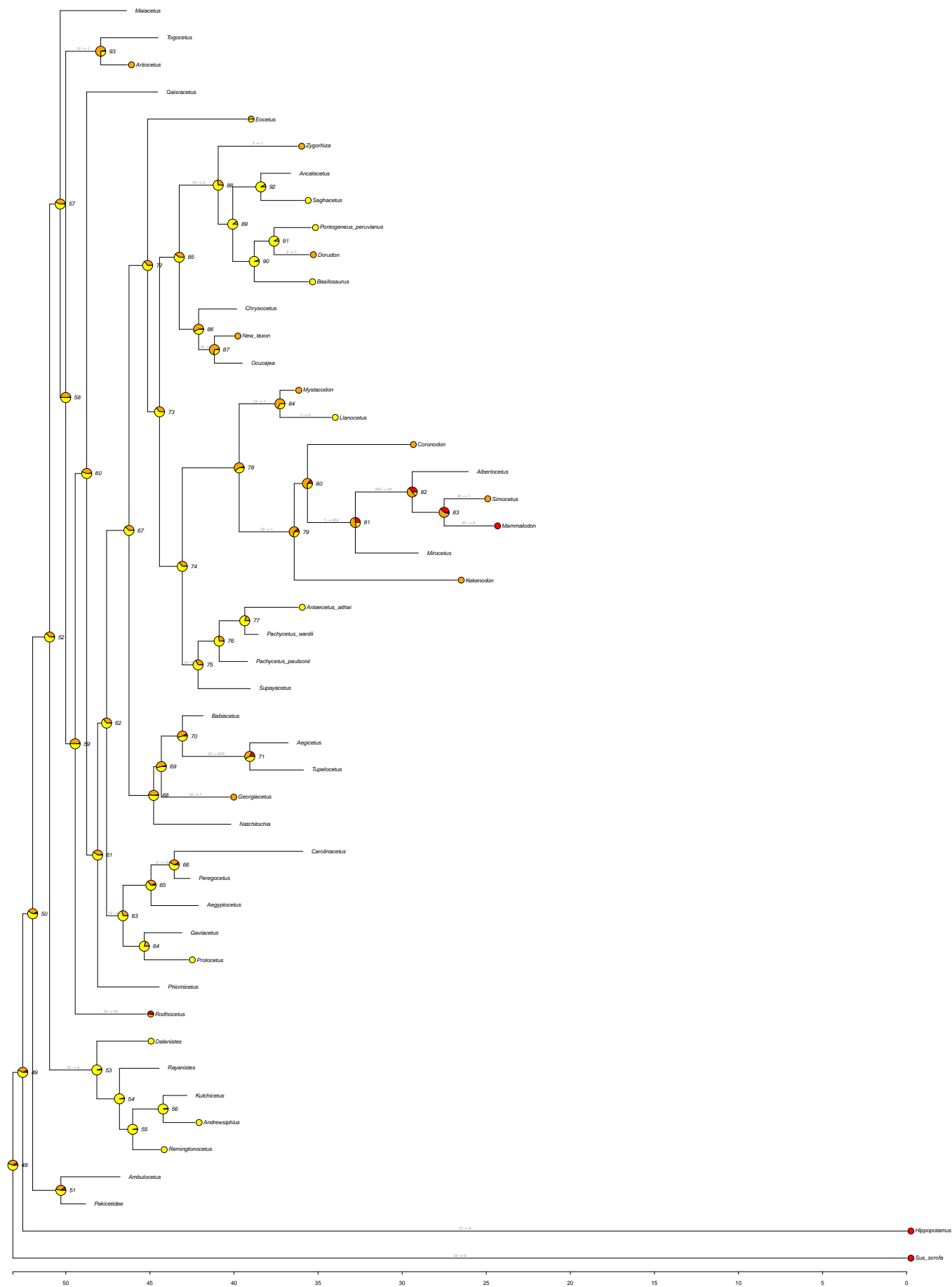

Supplement: Supplementary file 6 — Supplementary Data 3 [file 42003_2023_4986_MOESM6_ESM.zip › Supplementary Data 3/Supplementary Data 1_BTD_ASR/trait_0003_tree.plot.pdf]

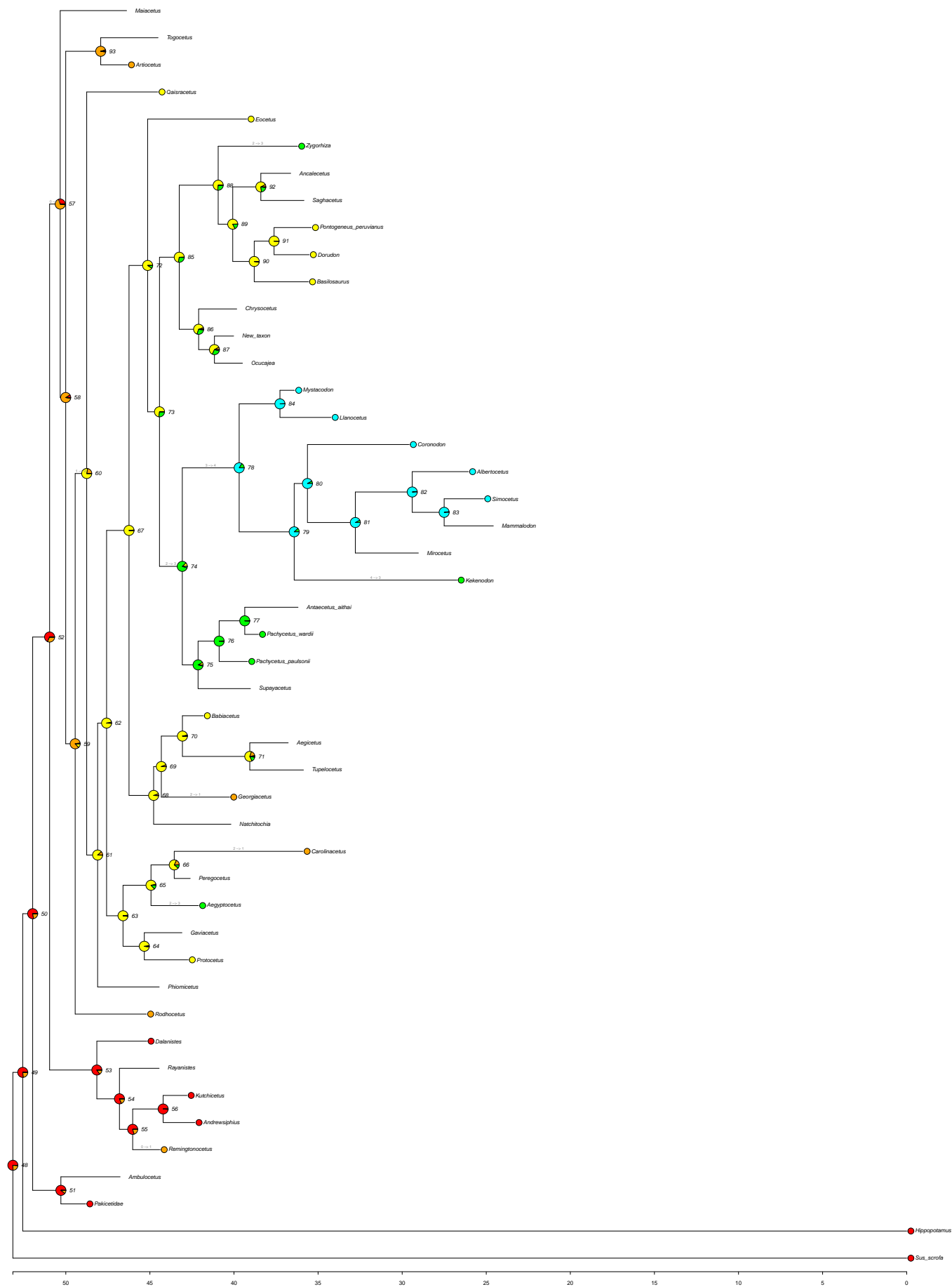

Supplement: Supplementary file 6 — Supplementary Data 3 [file 42003_2023_4986_MOESM6_ESM.zip › Supplementary Data 3/Supplementary Data 1_BTD_ASR/trait_0004_tree.plot.pdf]

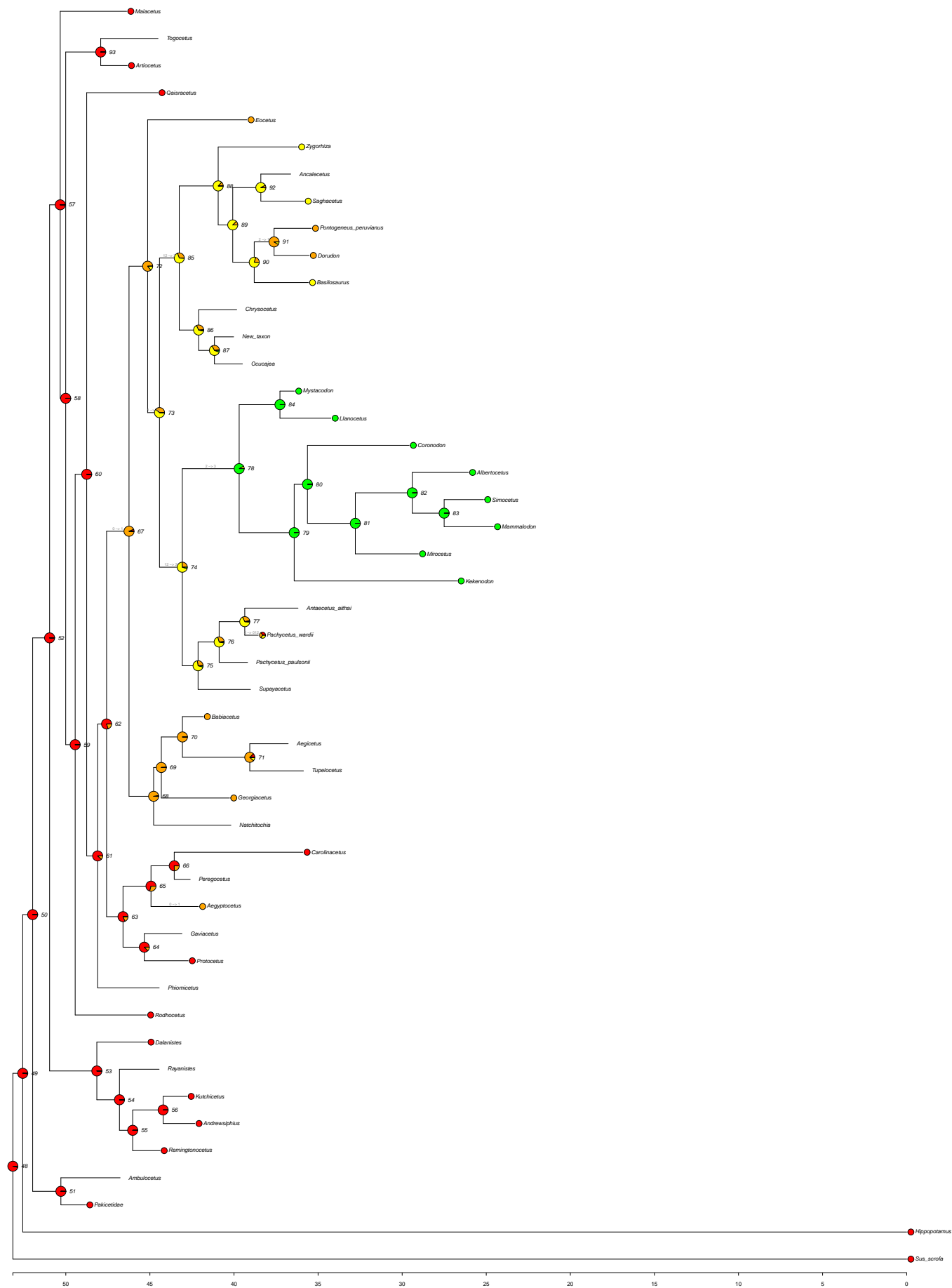

Supplement: Supplementary file 6 — Supplementary Data 3 [file 42003_2023_4986_MOESM6_ESM.zip › Supplementary Data 3/Supplementary Data 1_BTD_ASR/trait_0005_tree.plot.pdf]

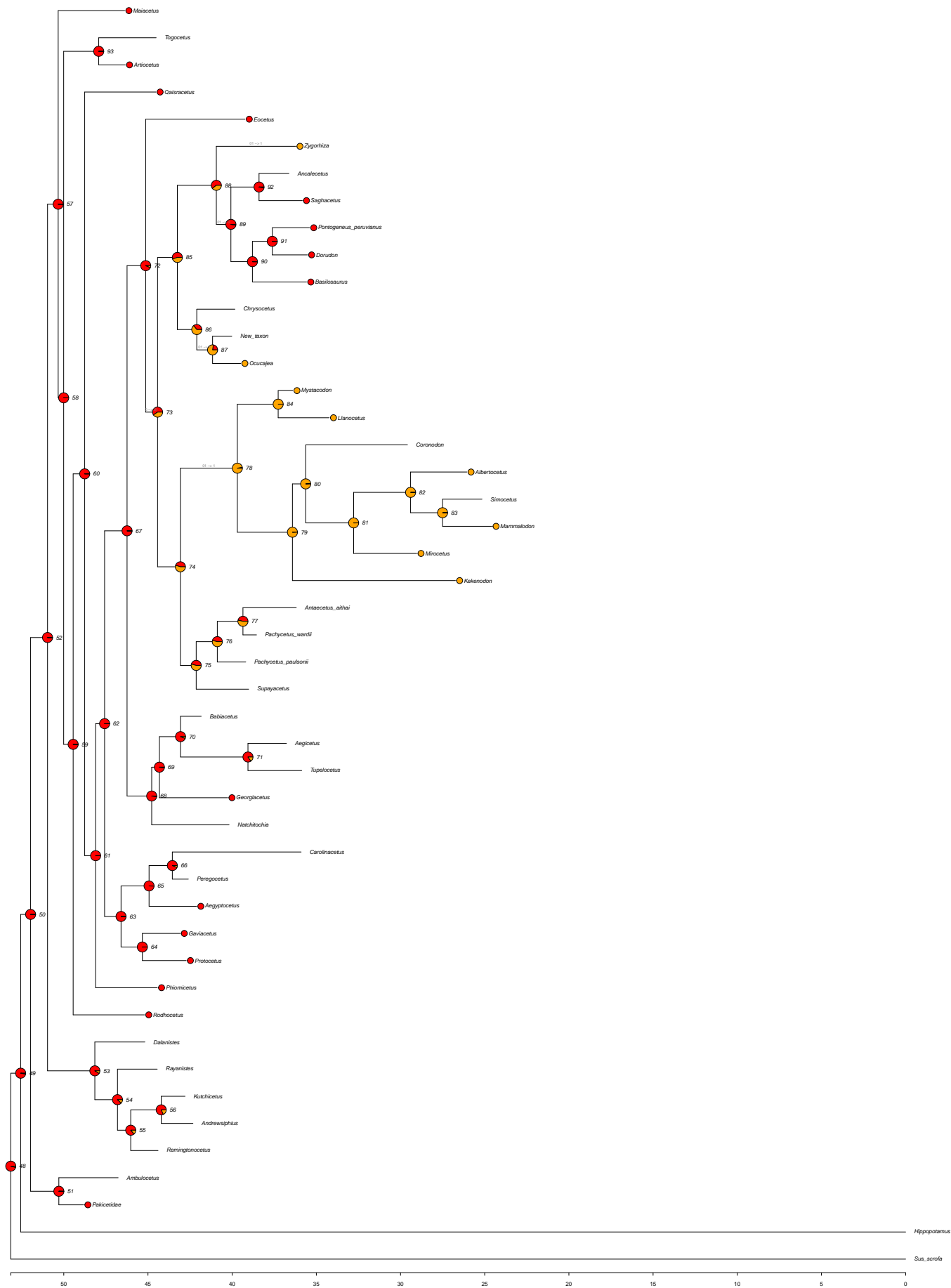

state 0 state 1

Supplement: Supplementary file 6 — Supplementary Data 3 [file 42003_2023_4986_MOESM6_ESM.zip › Supplementary Data 3/Supplementary Data 1_BTD_ASR/trait_0006_tree.plot.pdf]

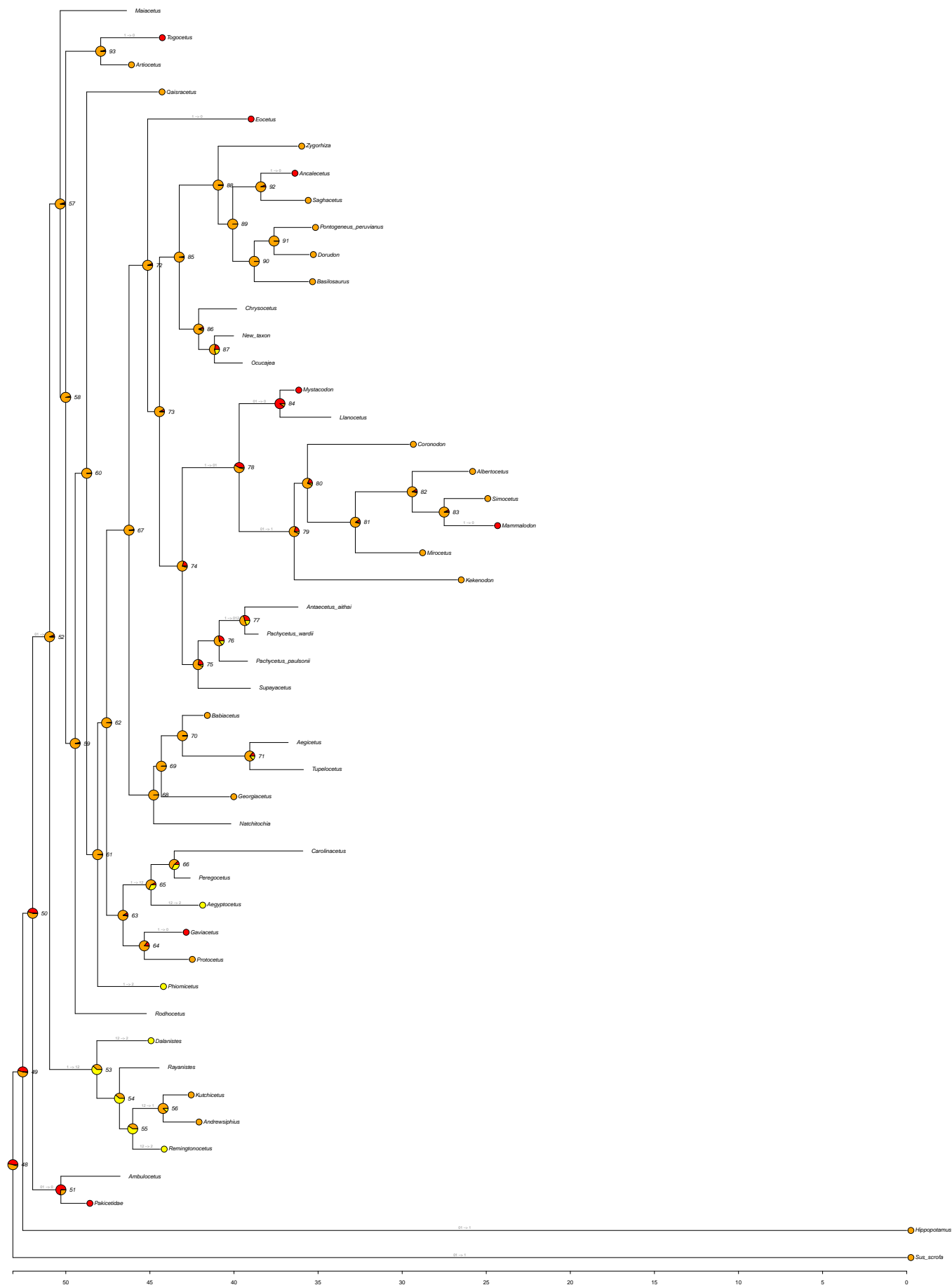

Supplement: Supplementary file 6 — Supplementary Data 3 [file 42003_2023_4986_MOESM6_ESM.zip › Supplementary Data 3/Supplementary Data 1_BTD_ASR/trait_0007_tree.plot.pdf]

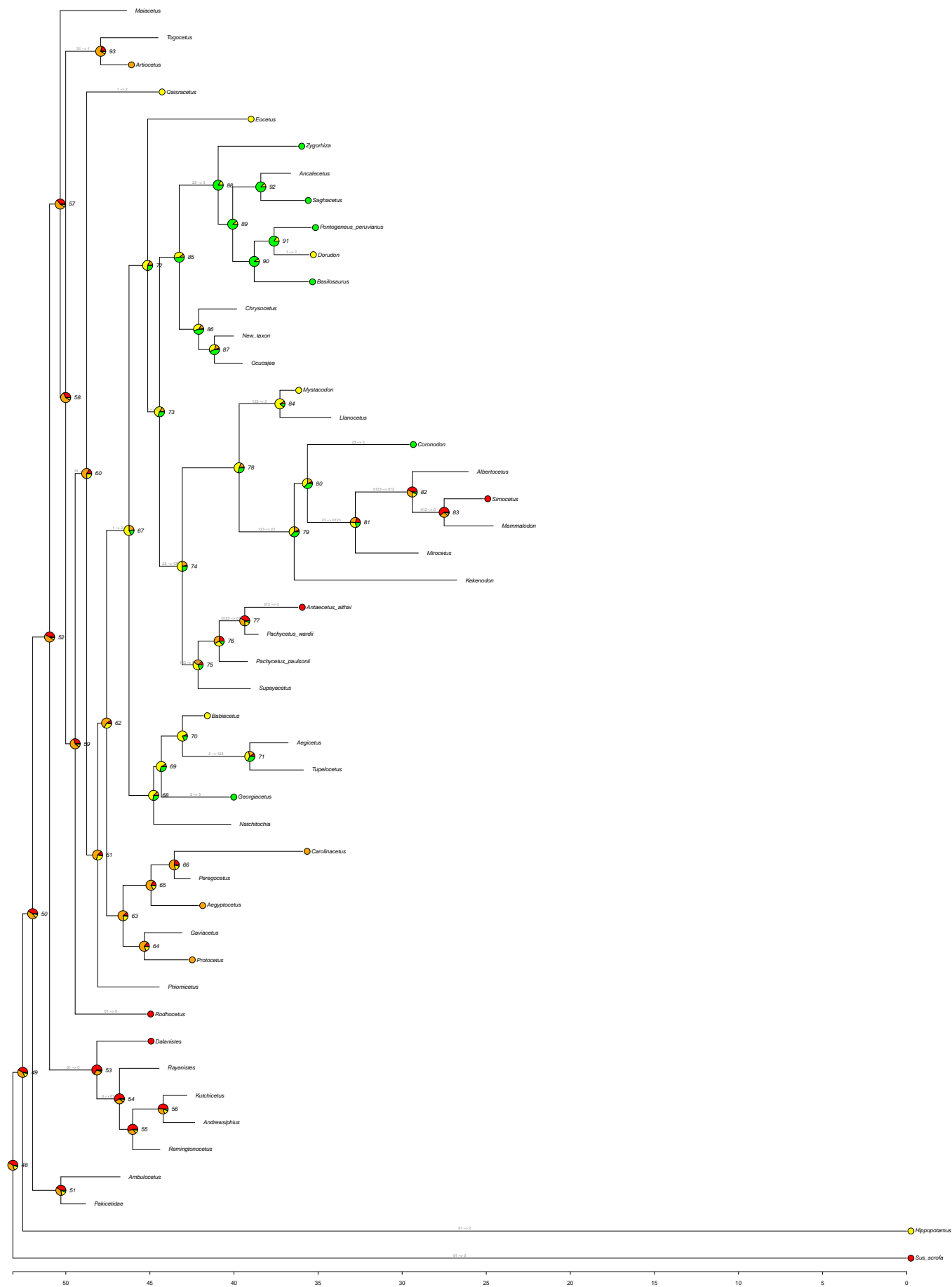

Supplement: Supplementary file 6 — Supplementary Data 3 [file 42003_2023_4986_MOESM6_ESM.zip › Supplementary Data 3/Supplementary Data 1_BTD_ASR/trait_0008_tree.plot.pdf]

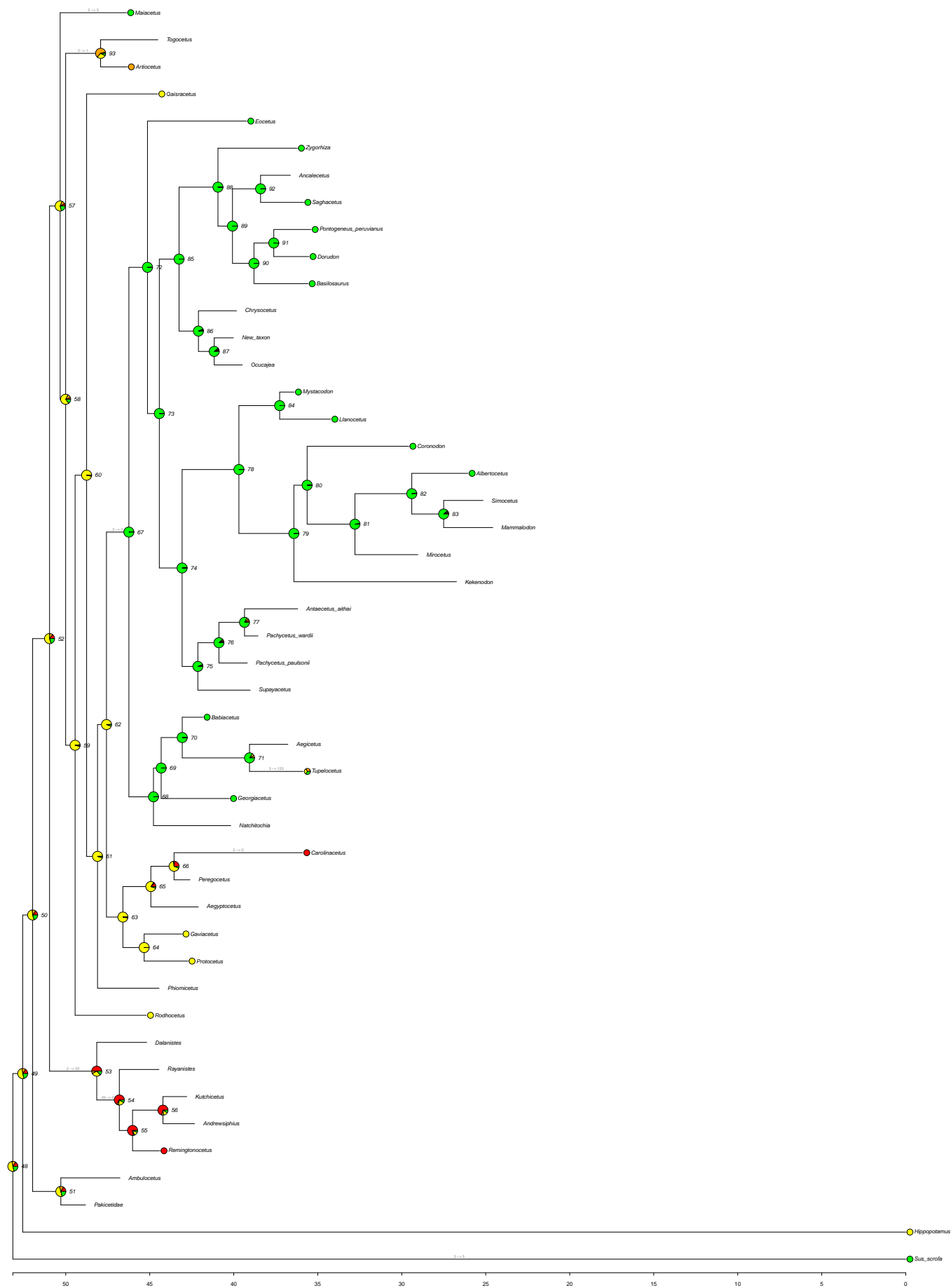

Supplement: Supplementary file 6 — Supplementary Data 3 [file 42003_2023_4986_MOESM6_ESM.zip › Supplementary Data 3/Supplementary Data 1_BTD_ASR/trait_0009_tree.plot.pdf]

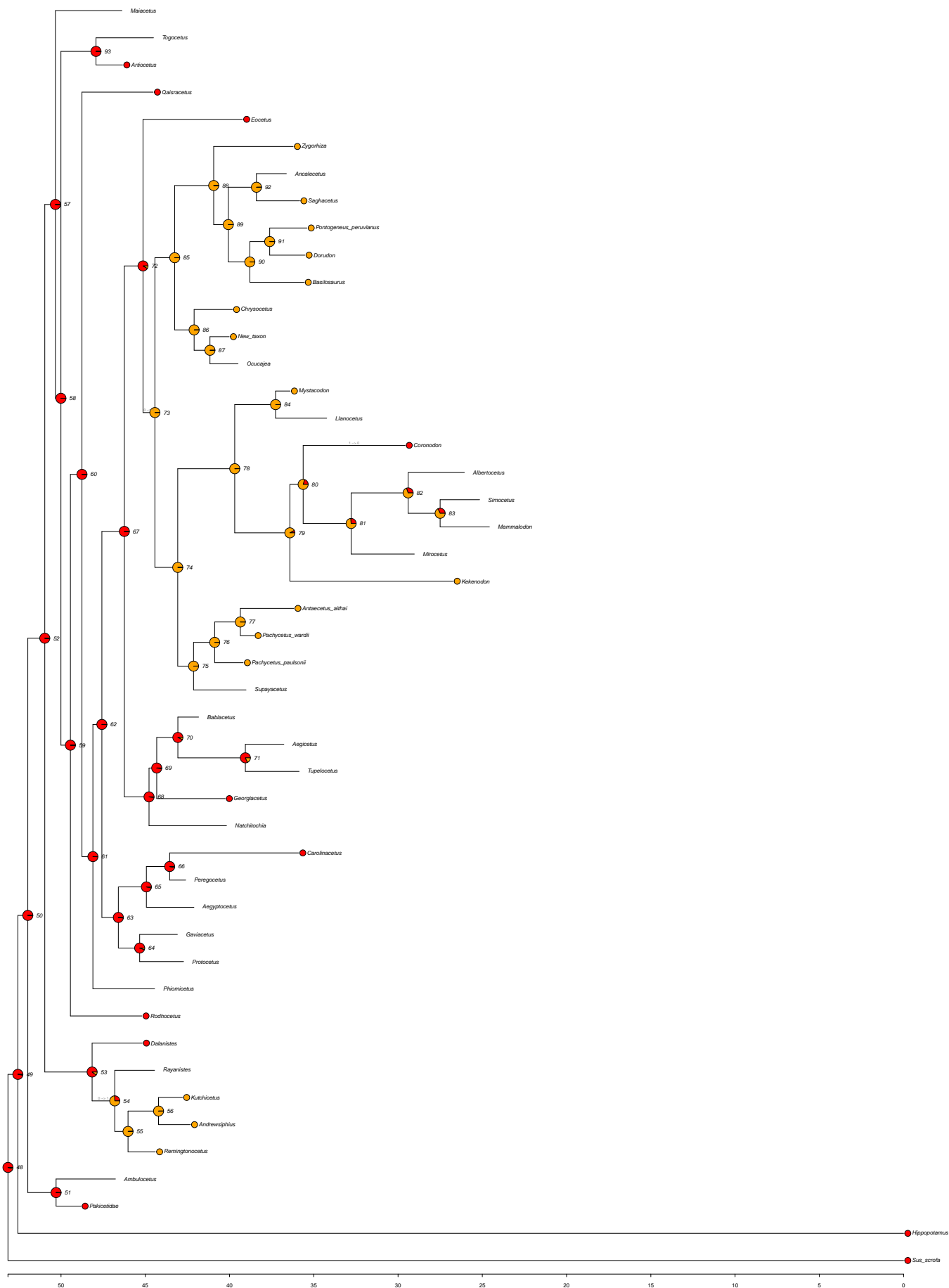

Supplement: Supplementary file 6 — Supplementary Data 3 [file 42003_2023_4986_MOESM6_ESM.zip › Supplementary Data 3/Supplementary Data 1_BTD_ASR/trait_0010_tree.plot.pdf]

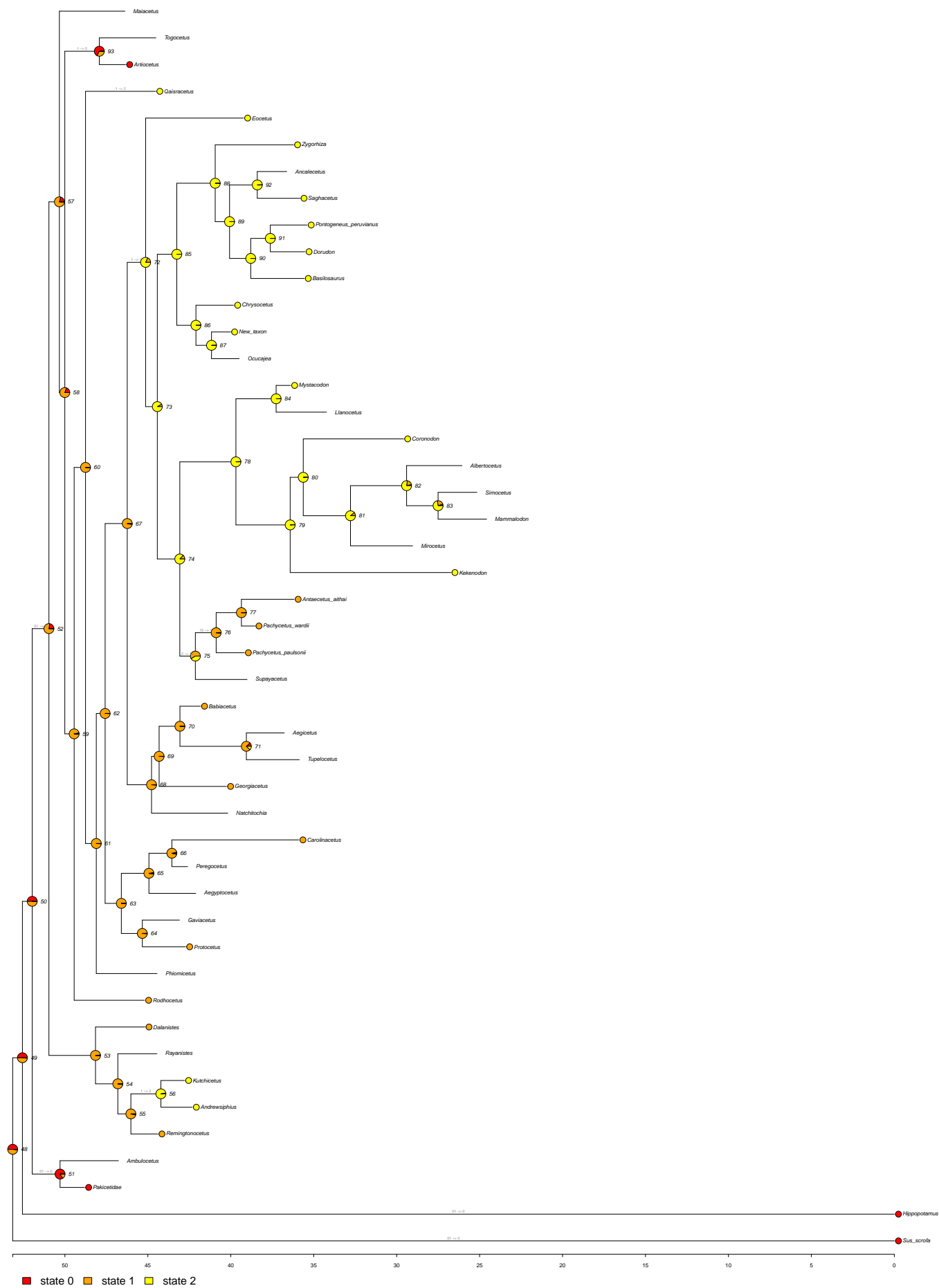

Supplement: Supplementary file 6 — Supplementary Data 3 [file 42003_2023_4986_MOESM6_ESM.zip › Supplementary Data 3/Supplementary Data 1_BTD_ASR/trait_0011_tree.plot.pdf]

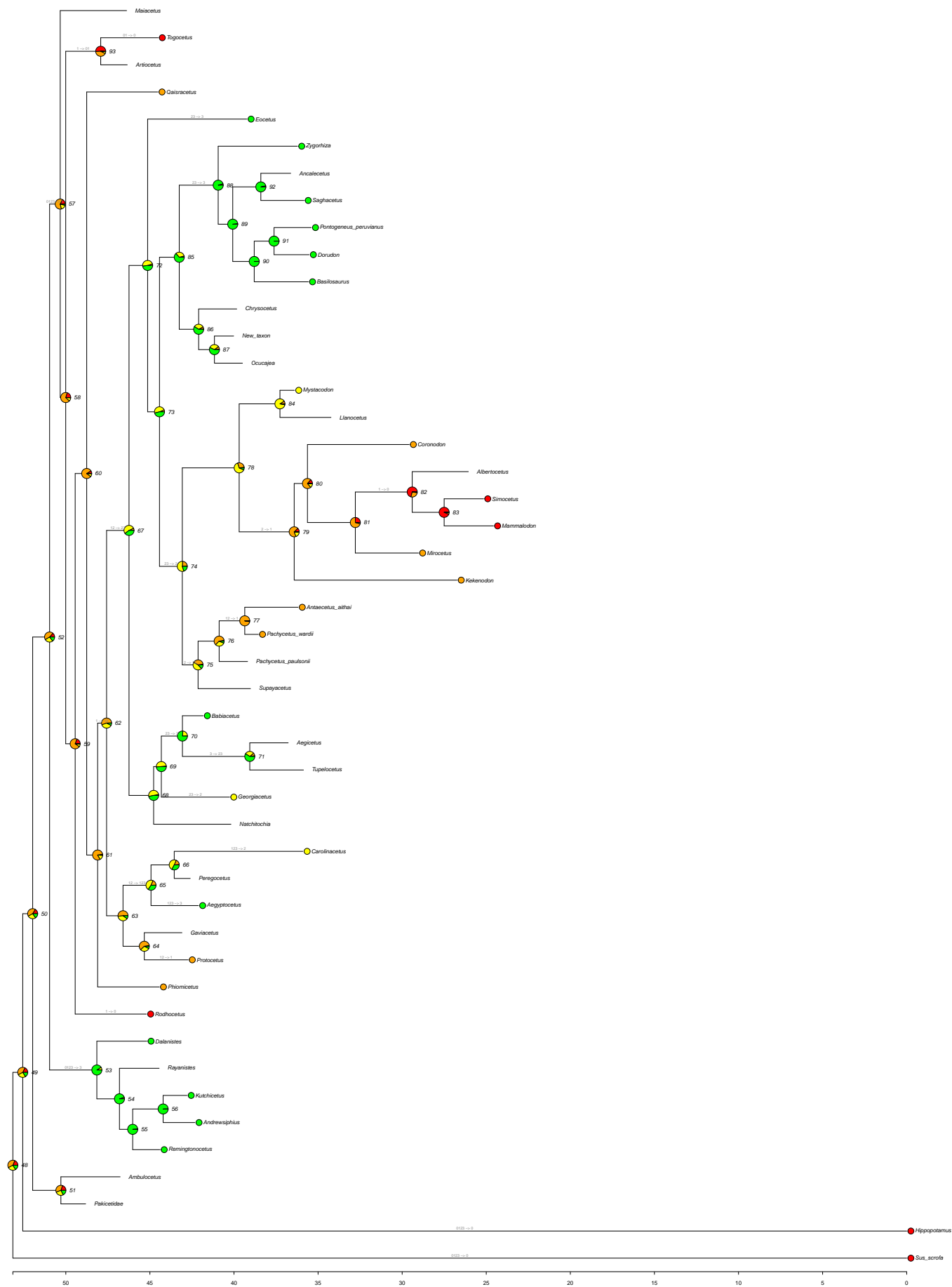

Supplement: Supplementary file 6 — Supplementary Data 3 [file 42003_2023_4986_MOESM6_ESM.zip › Supplementary Data 3/Supplementary Data 1_BTD_ASR/trait_0012_tree.plot.pdf]

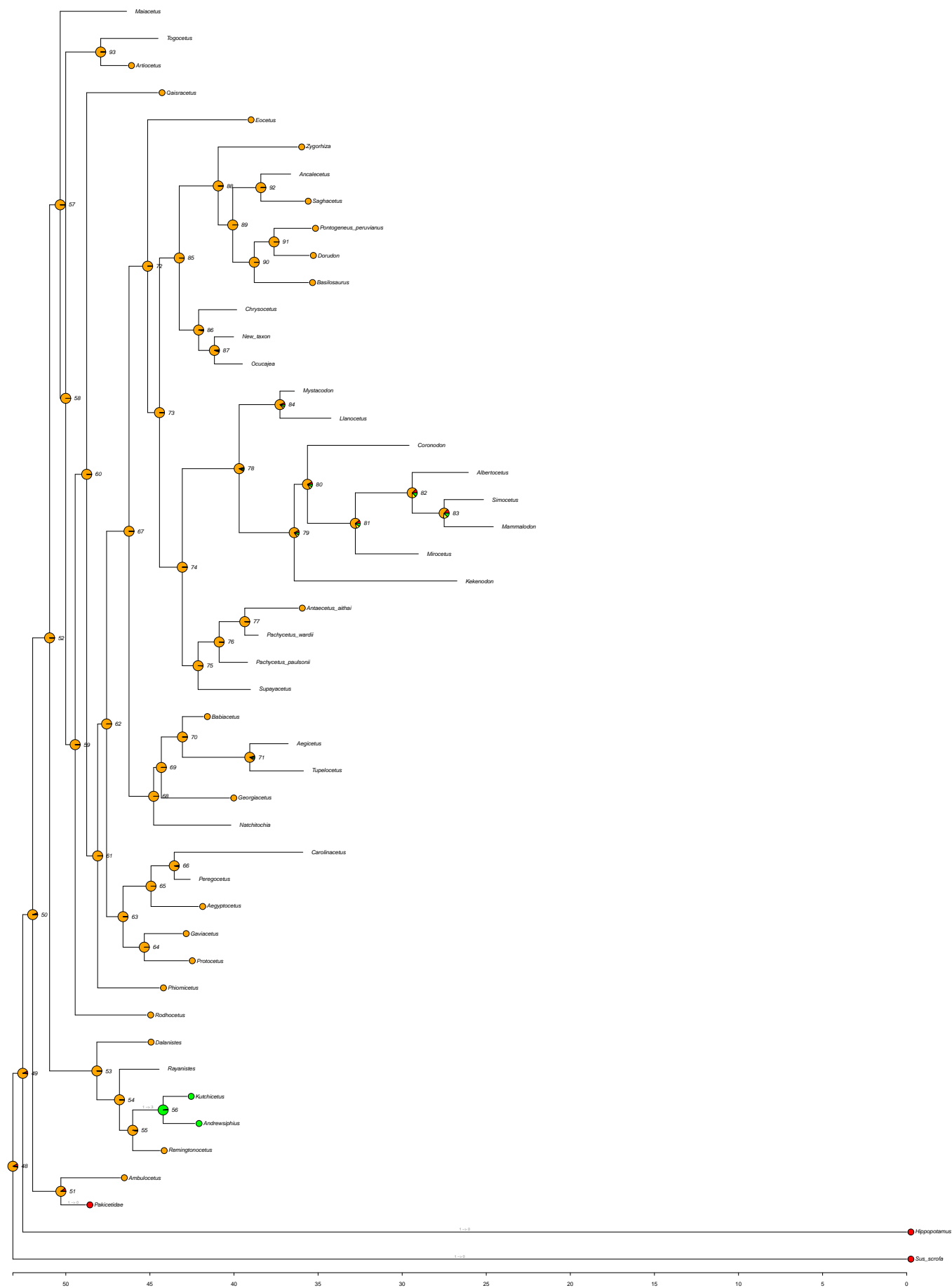

Supplement: Supplementary file 6 — Supplementary Data 3 [file 42003_2023_4986_MOESM6_ESM.zip › Supplementary Data 3/Supplementary Data 1_BTD_ASR/trait_0013_tree.plot.pdf]

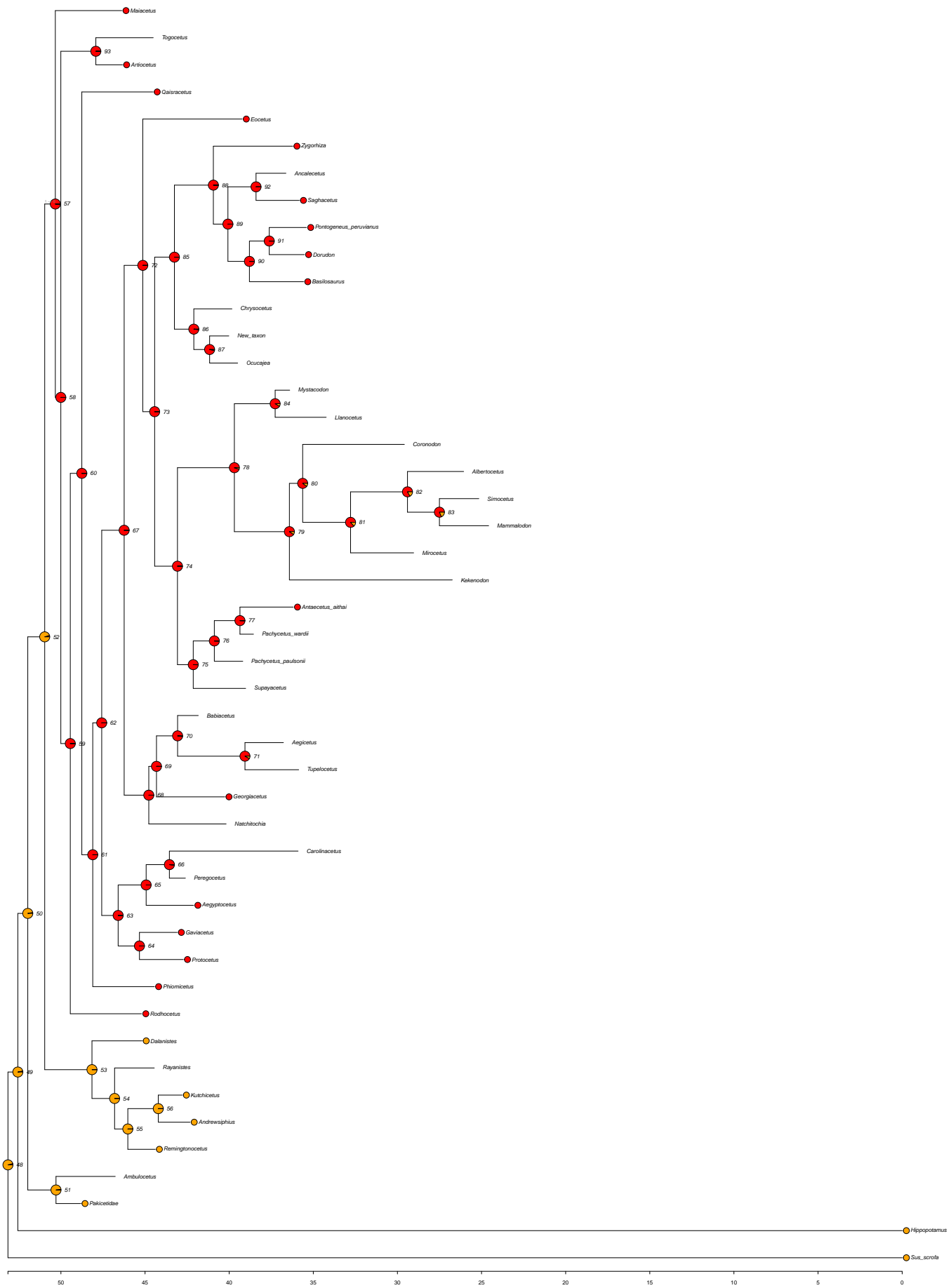

Supplement: Supplementary file 6 — Supplementary Data 3 [file 42003_2023_4986_MOESM6_ESM.zip › Supplementary Data 3/Supplementary Data 1_BTD_ASR/trait_0014_tree.plot.pdf]

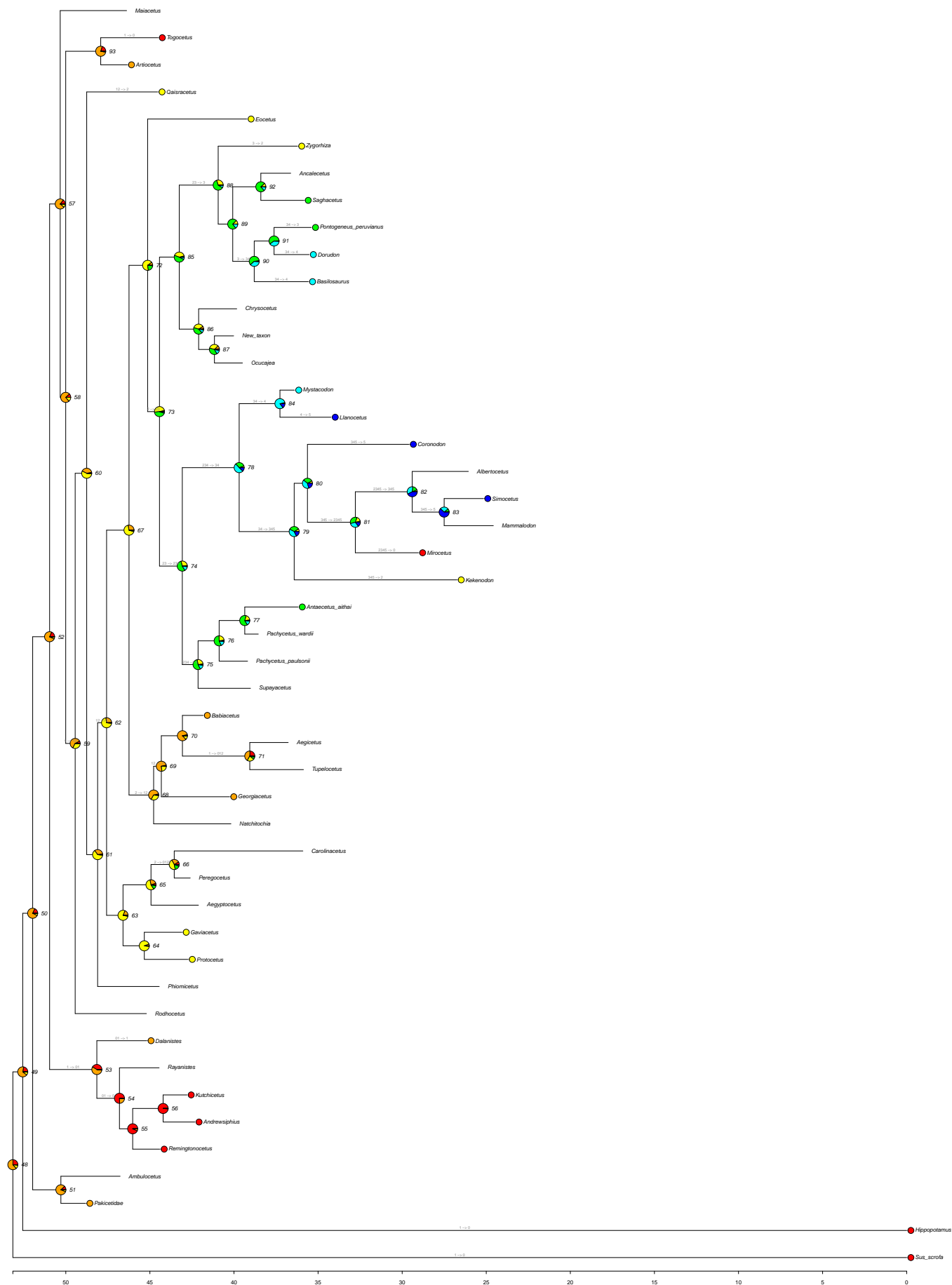

Supplement: Supplementary file 6 — Supplementary Data 3 [file 42003_2023_4986_MOESM6_ESM.zip › Supplementary Data 3/Supplementary Data 1_BTD_ASR/trait_0015_tree.plot.pdf]

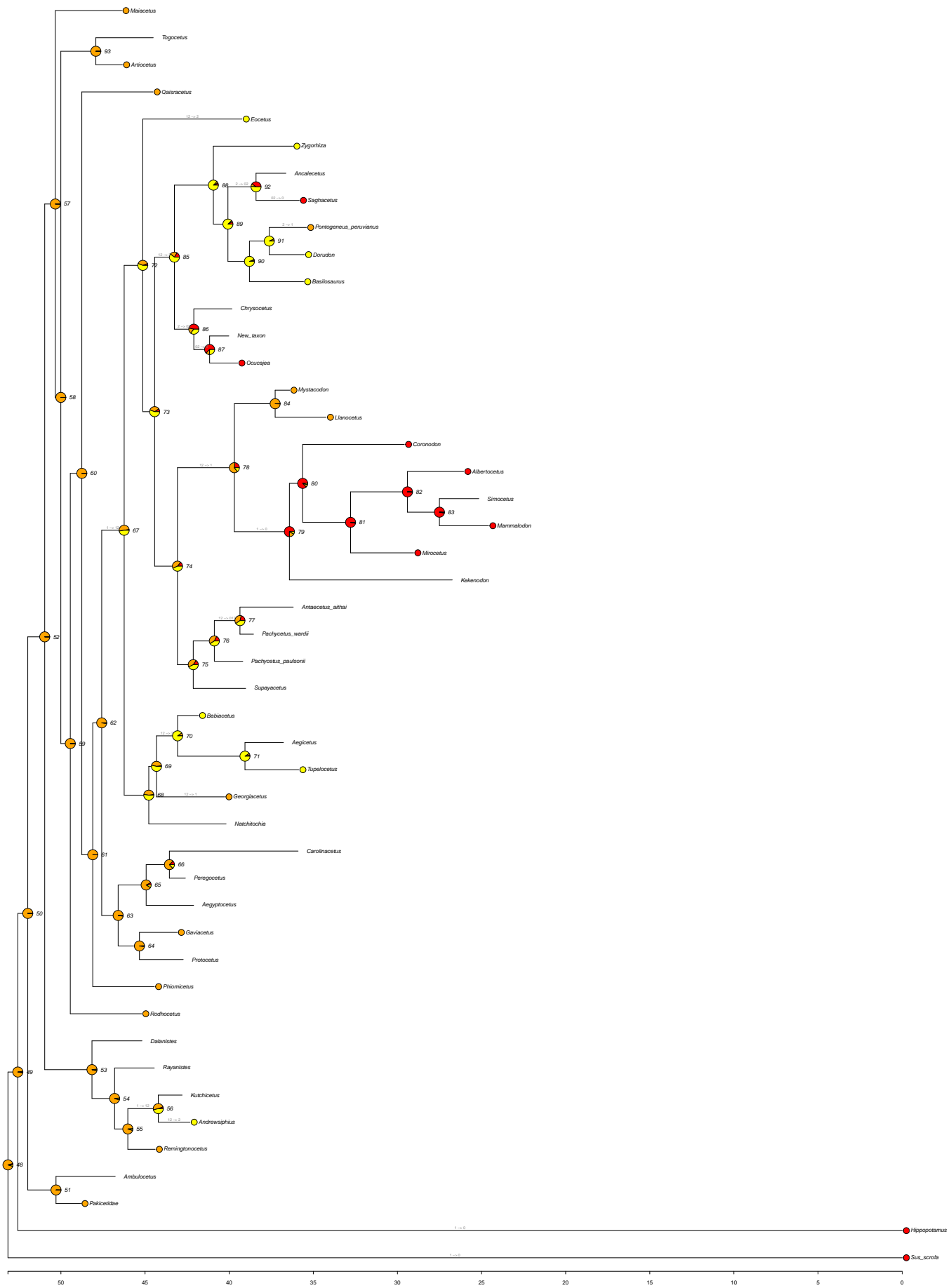

Supplement: Supplementary file 6 — Supplementary Data 3 [file 42003_2023_4986_MOESM6_ESM.zip › Supplementary Data 3/Supplementary Data 1_BTD_ASR/trait_0016_tree.plot.pdf]

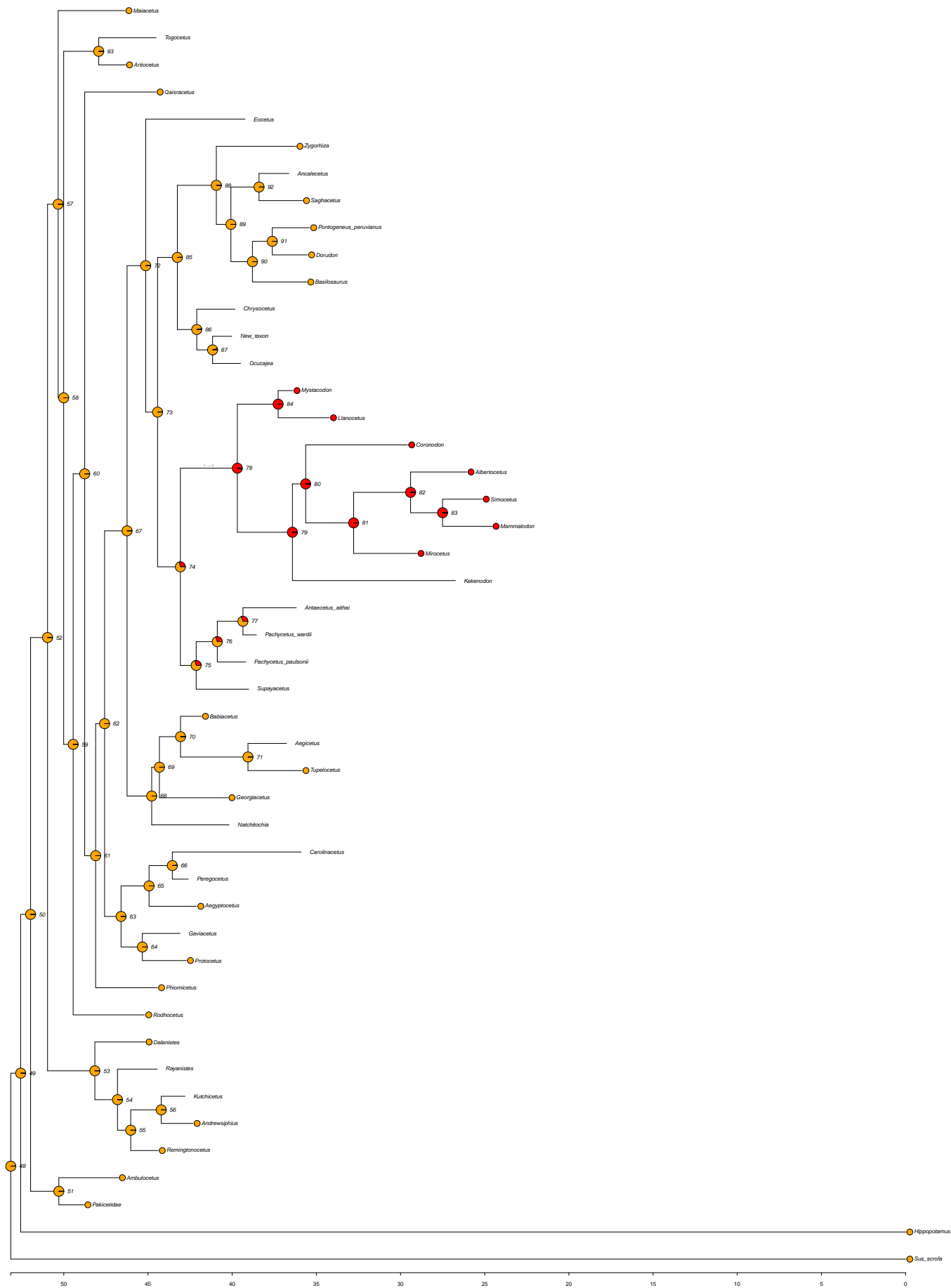

Supplement: Supplementary file 6 — Supplementary Data 3 [file 42003_2023_4986_MOESM6_ESM.zip › Supplementary Data 3/Supplementary Data 1_BTD_ASR/trait_0017_tree.plot.pdf]

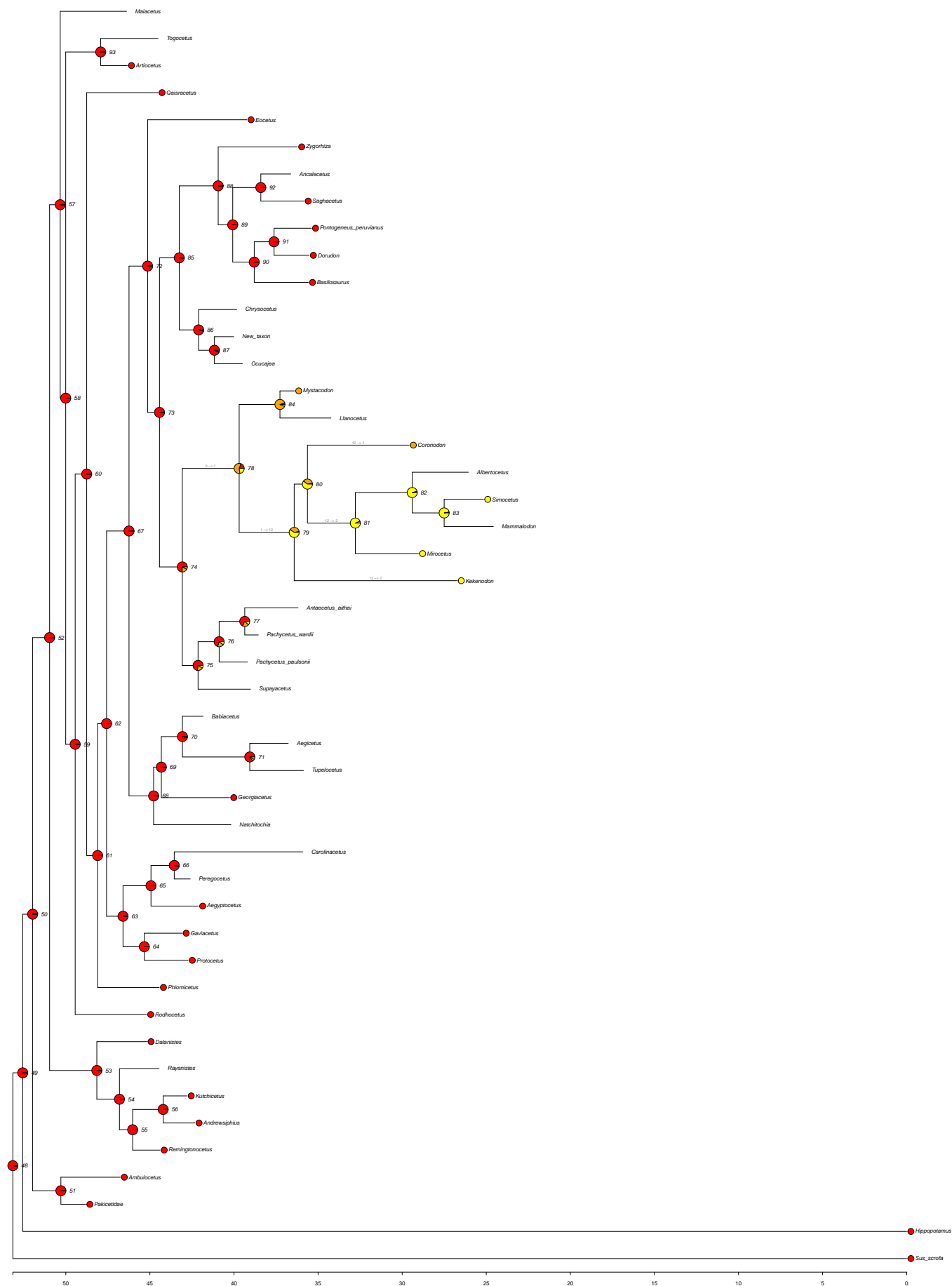

Supplement: Supplementary file 6 — Supplementary Data 3 [file 42003_2023_4986_MOESM6_ESM.zip › Supplementary Data 3/Supplementary Data 1_BTD_ASR/trait_0018_tree.plot.pdf]

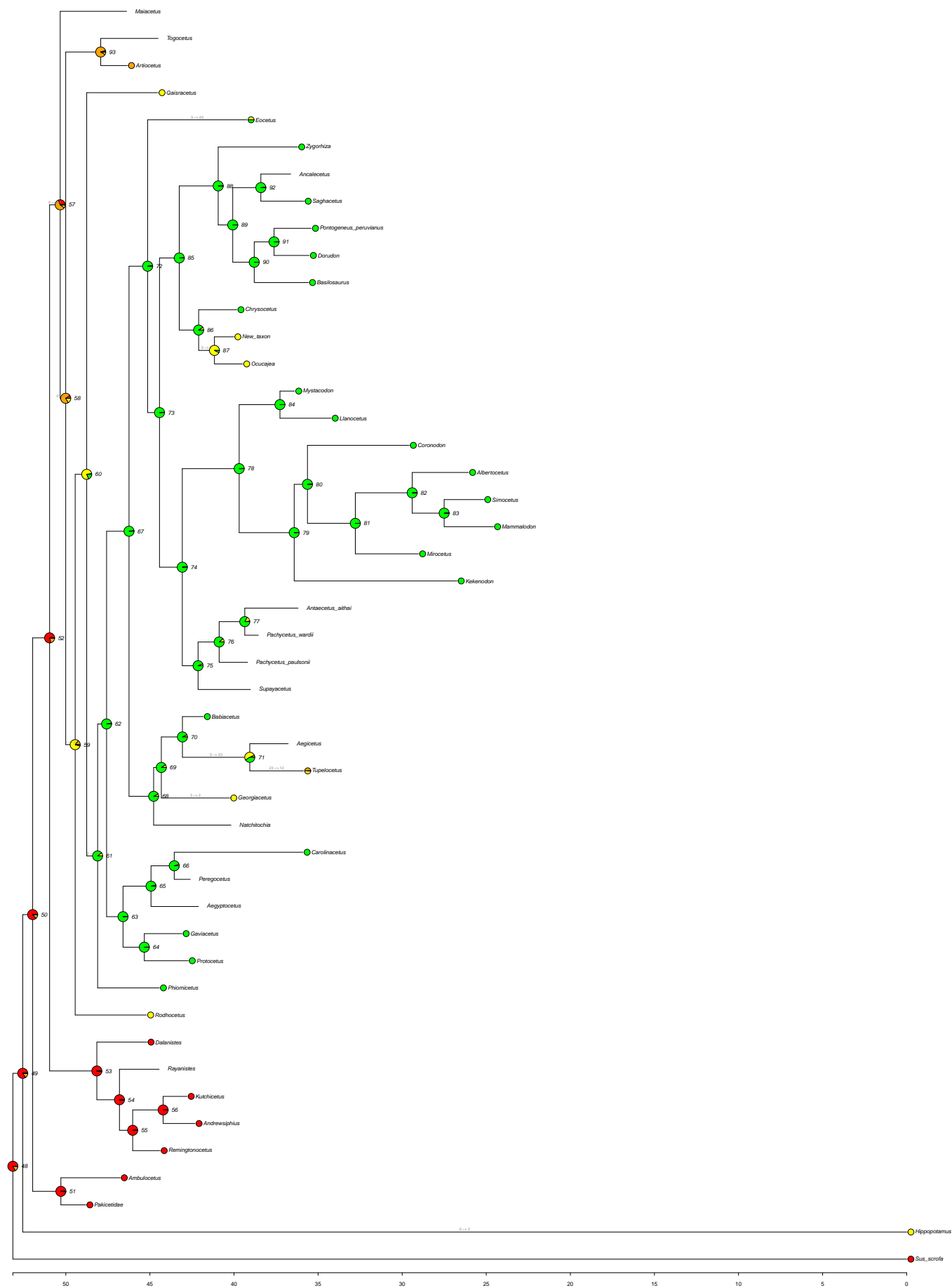

Supplement: Supplementary file 6 — Supplementary Data 3 [file 42003_2023_4986_MOESM6_ESM.zip › Supplementary Data 3/Supplementary Data 1_BTD_ASR/trait_0019_tree.plot.pdf]

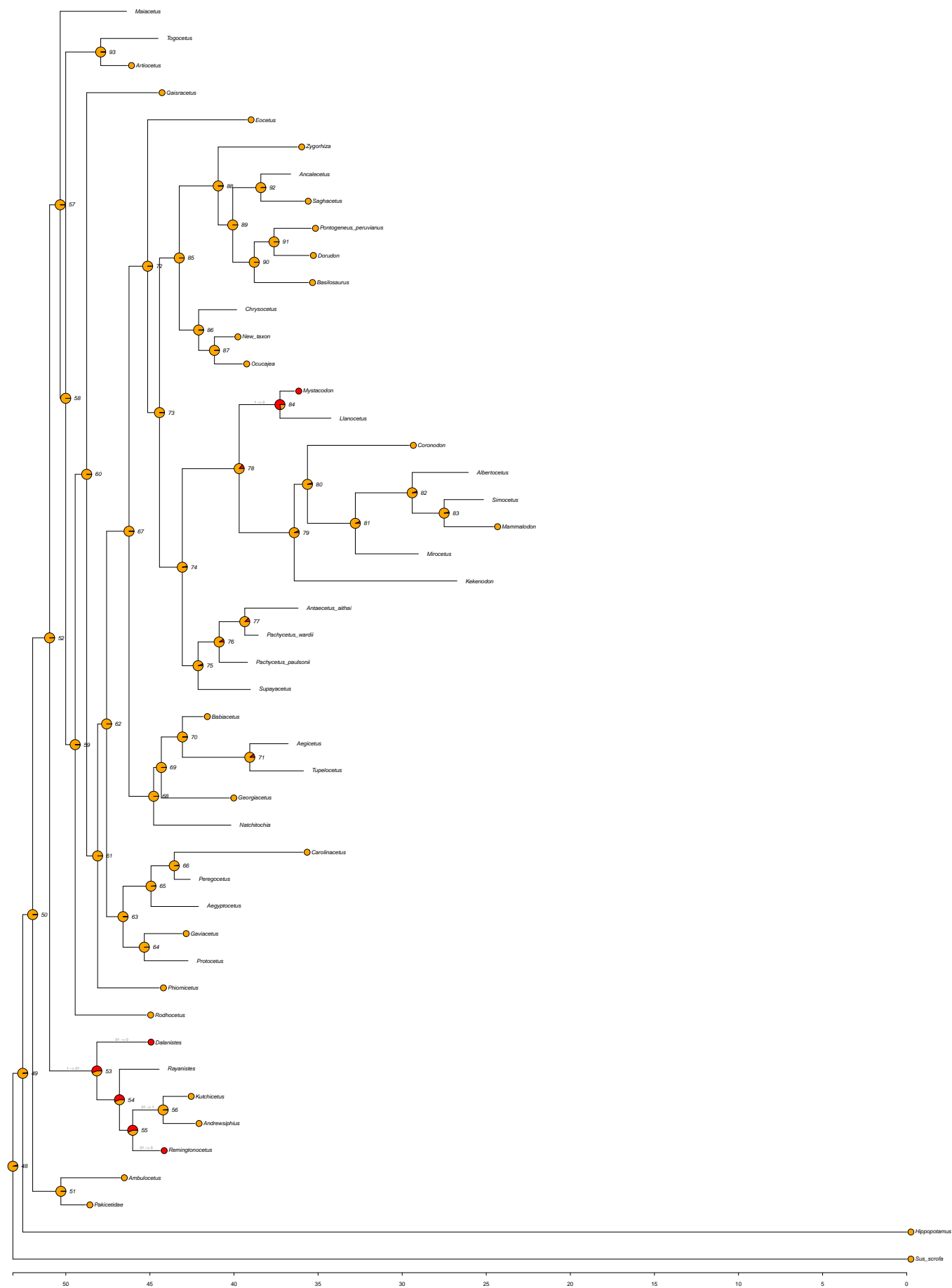

Supplement: Supplementary file 6 — Supplementary Data 3 [file 42003_2023_4986_MOESM6_ESM.zip › Supplementary Data 3/Supplementary Data 1_BTD_ASR/trait_0020_tree.plot.pdf]

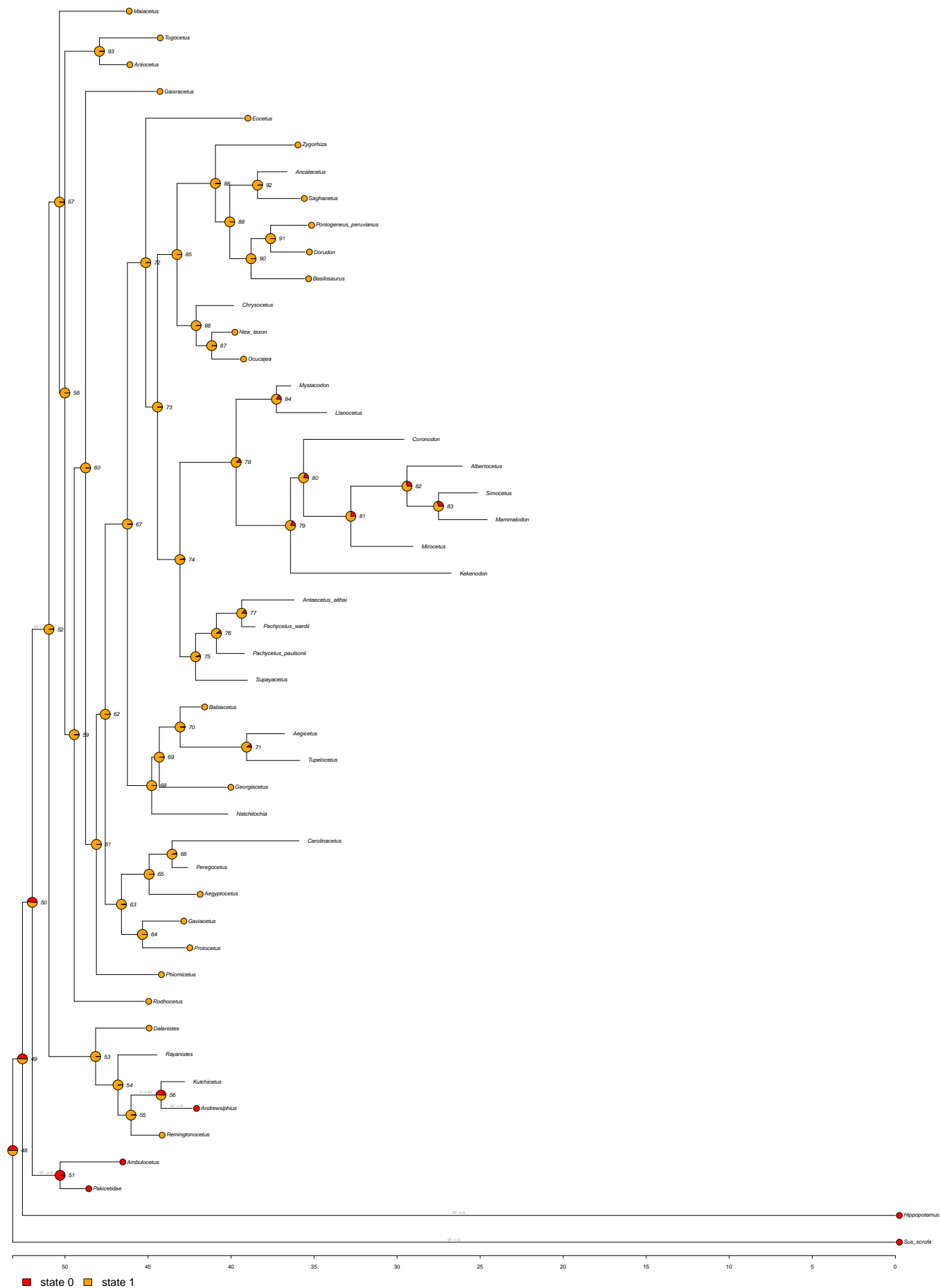

Supplement: Supplementary file 6 — Supplementary Data 3 [file 42003_2023_4986_MOESM6_ESM.zip › Supplementary Data 3/Supplementary Data 1_BTD_ASR/trait_0021_tree.plot.pdf]

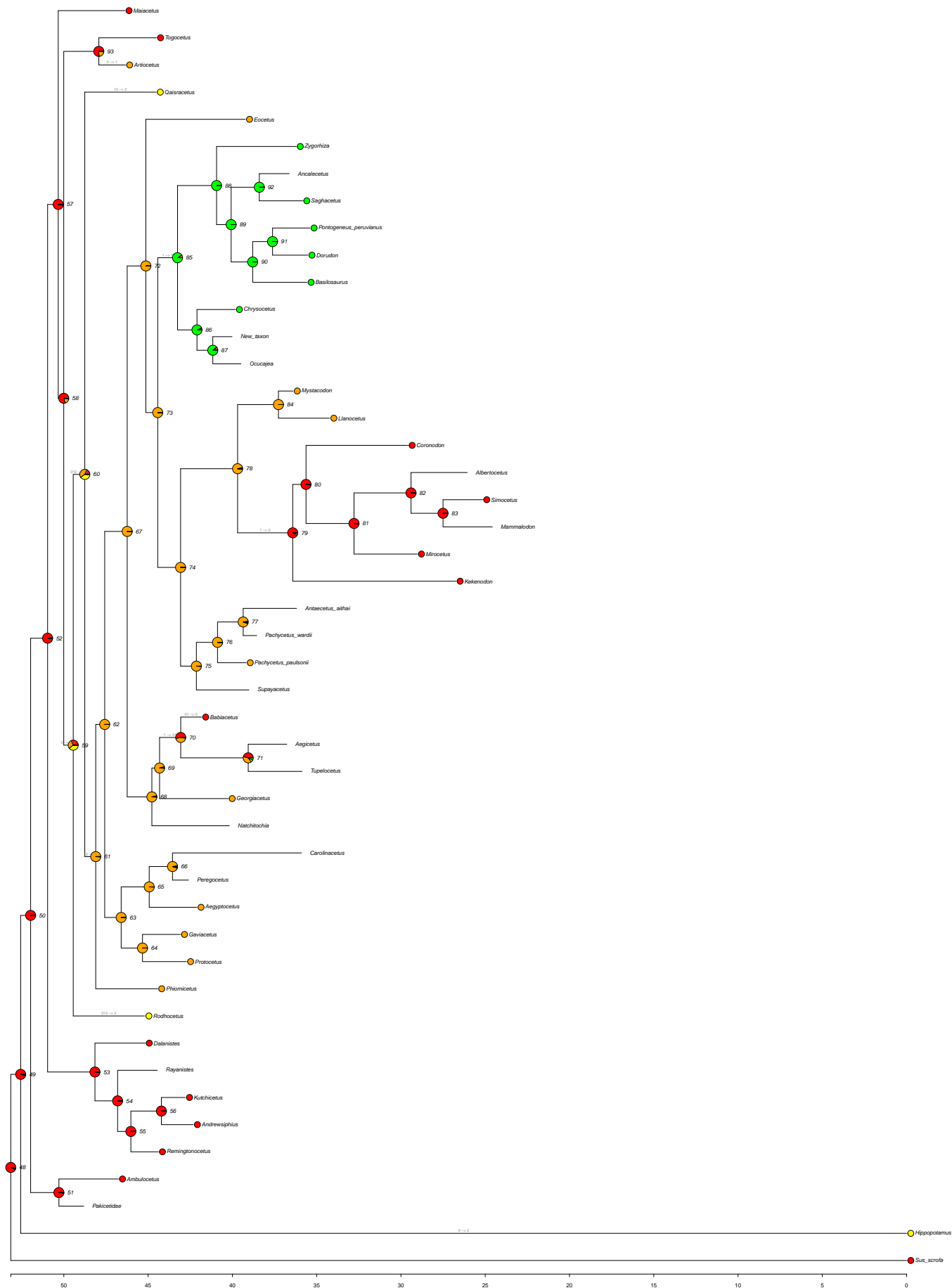

Supplement: Supplementary file 6 — Supplementary Data 3 [file 42003_2023_4986_MOESM6_ESM.zip › Supplementary Data 3/Supplementary Data 1_BTD_ASR/trait_0022_tree.plot.pdf]

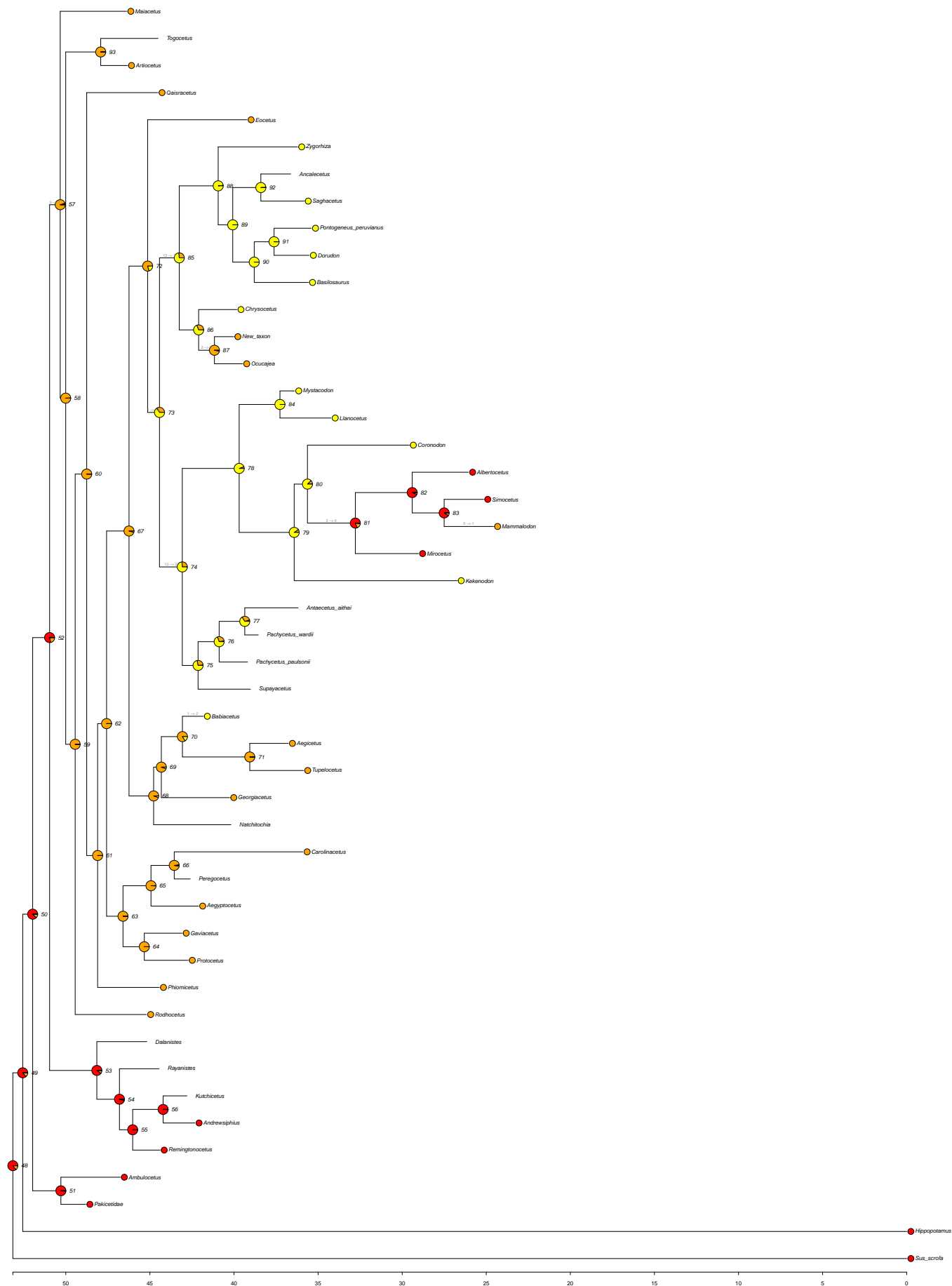

Supplement: Supplementary file 6 — Supplementary Data 3 [file 42003_2023_4986_MOESM6_ESM.zip › Supplementary Data 3/Supplementary Data 1_BTD_ASR/trait_0023_tree.plot.pdf]

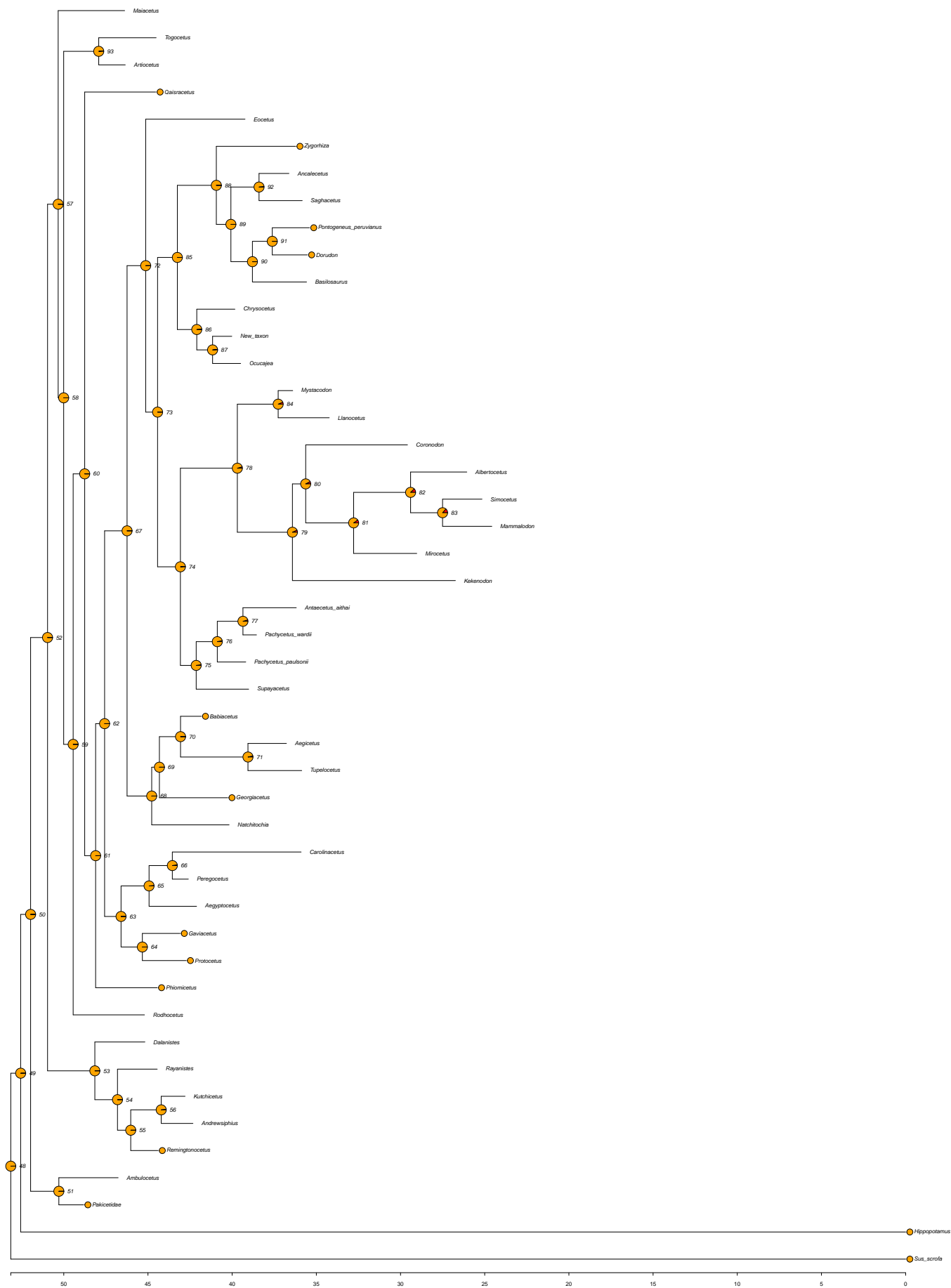

Supplement: Supplementary file 6 — Supplementary Data 3 [file 42003_2023_4986_MOESM6_ESM.zip › Supplementary Data 3/Supplementary Data 1_BTD_ASR/trait_0024_tree.plot.pdf]

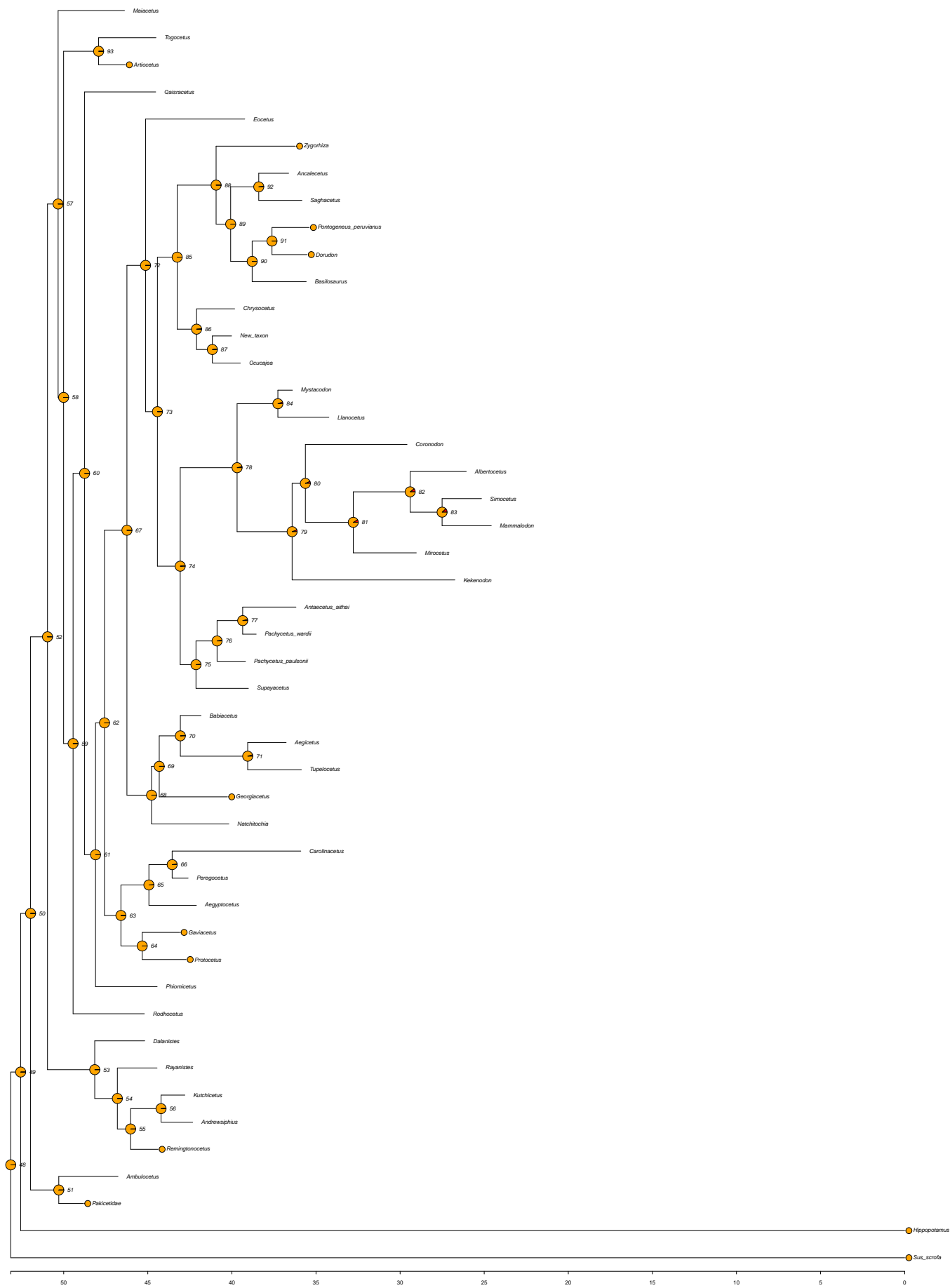

state 0 state 1

Supplement: Supplementary file 6 — Supplementary Data 3 [file 42003_2023_4986_MOESM6_ESM.zip › Supplementary Data 3/Supplementary Data 1_BTD_ASR/trait_0025_tree.plot.pdf]

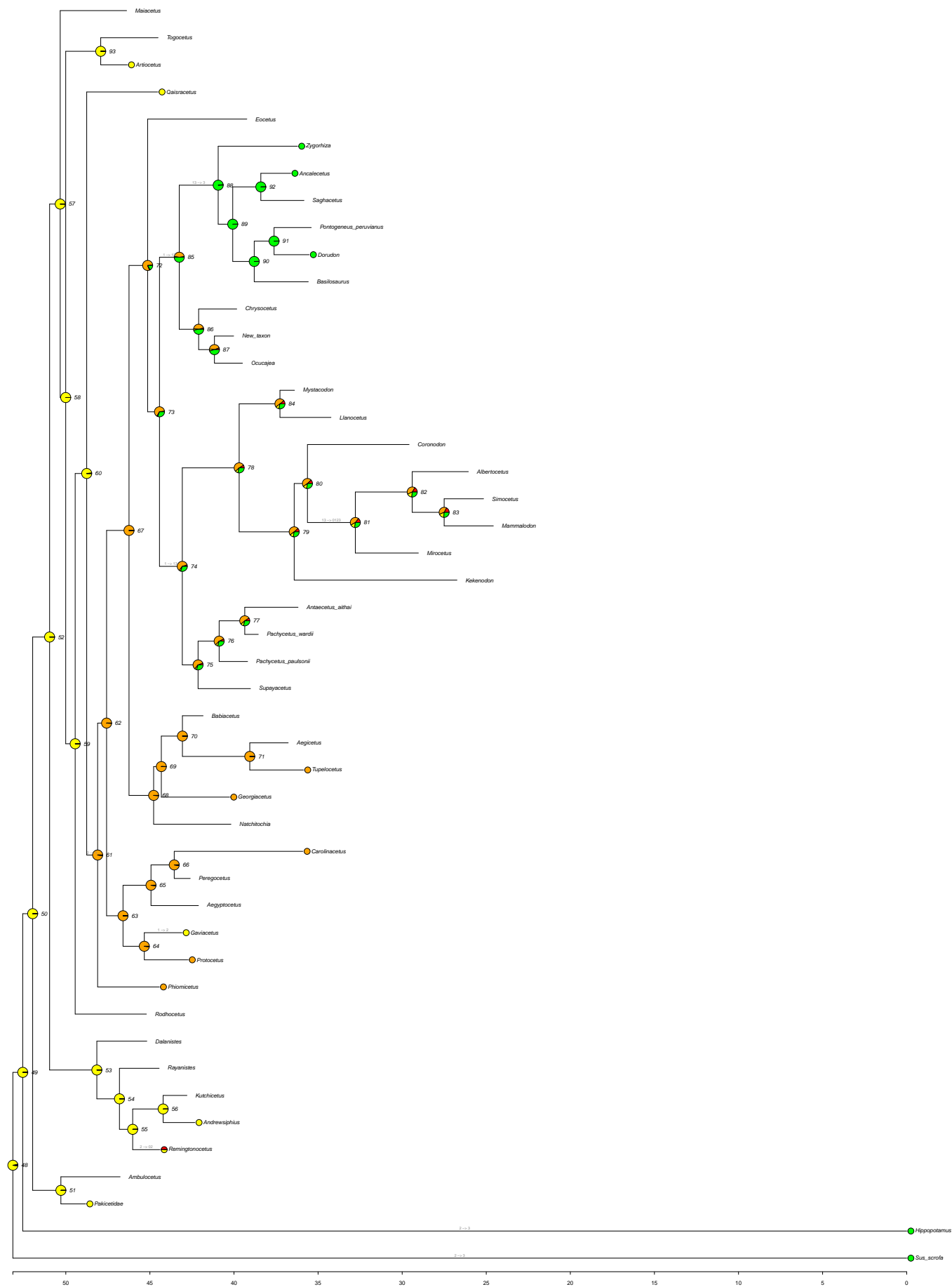

Supplement: Supplementary file 6 — Supplementary Data 3 [file 42003_2023_4986_MOESM6_ESM.zip › Supplementary Data 3/Supplementary Data 1_BTD_ASR/trait_0026_tree.plot.pdf]

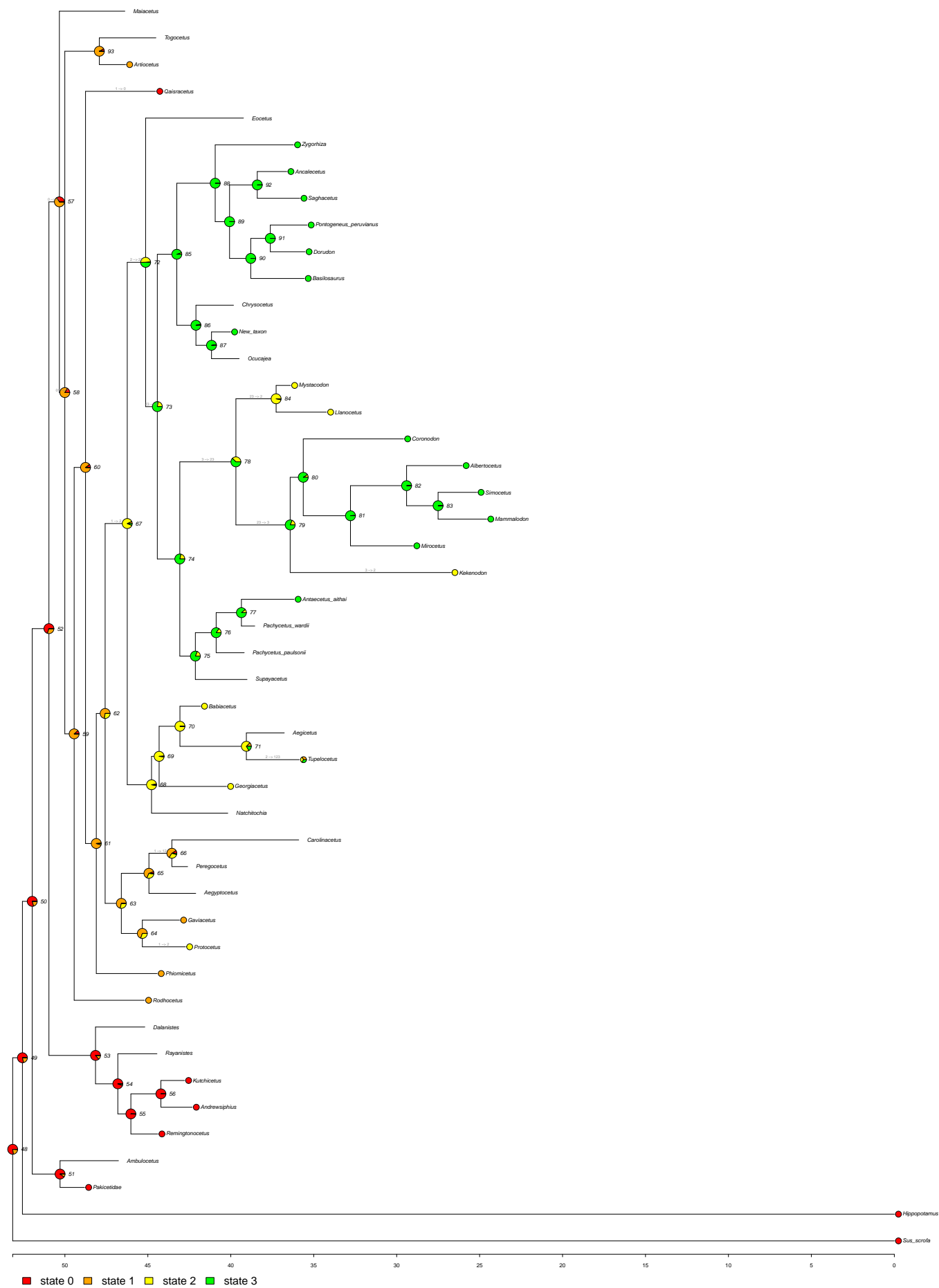

Supplement: Supplementary file 6 — Supplementary Data 3 [file 42003_2023_4986_MOESM6_ESM.zip › Supplementary Data 3/Supplementary Data 1_BTD_ASR/trait_0027_tree.plot.pdf]

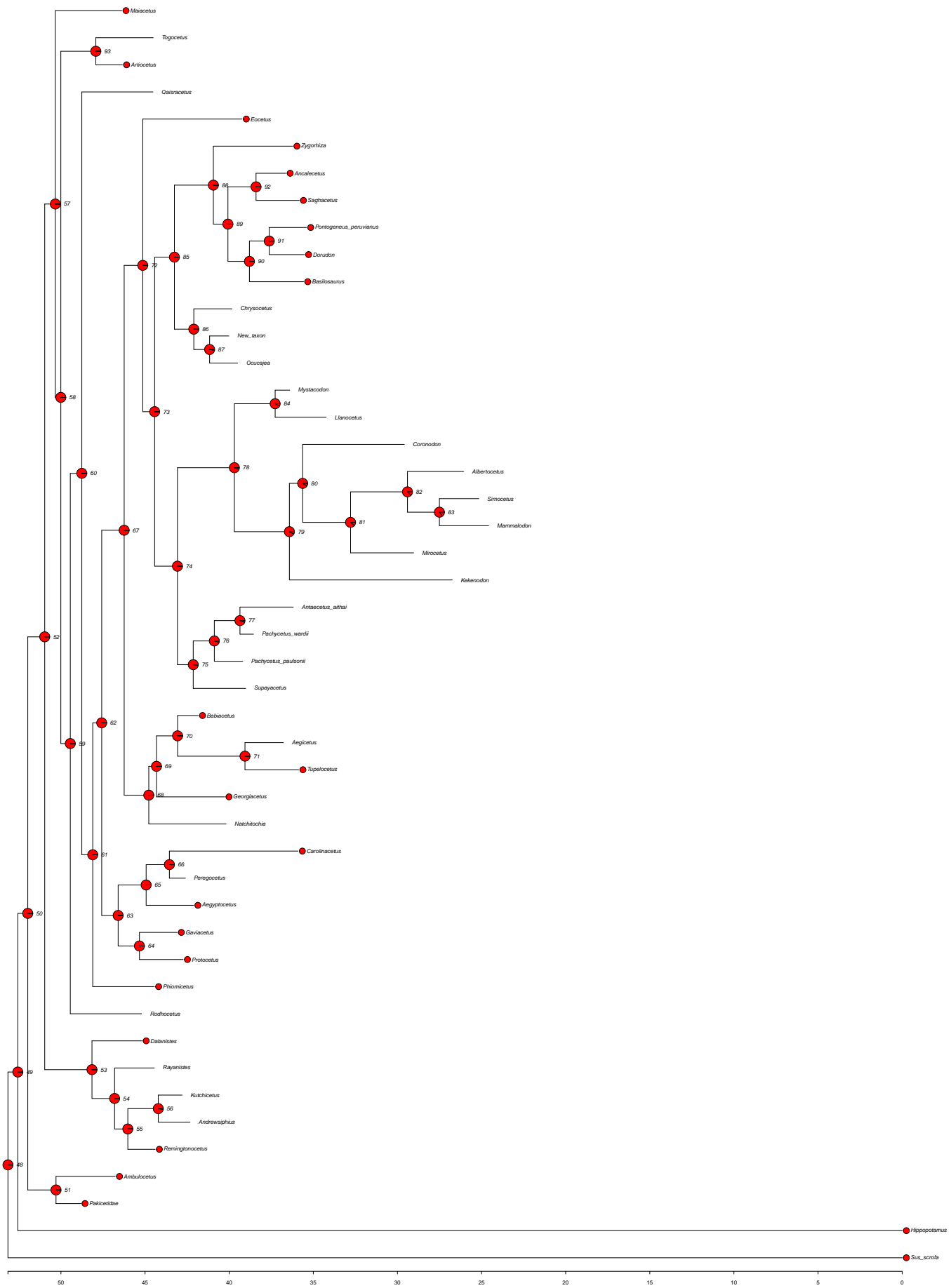

■ state 0

Supplement: Supplementary file 6 — Supplementary Data 3 [file 42003_2023_4986_MOESM6_ESM.zip › Supplementary Data 3/Supplementary Data 1_BTD_ASR/trait_0028_tree.plot.pdf]

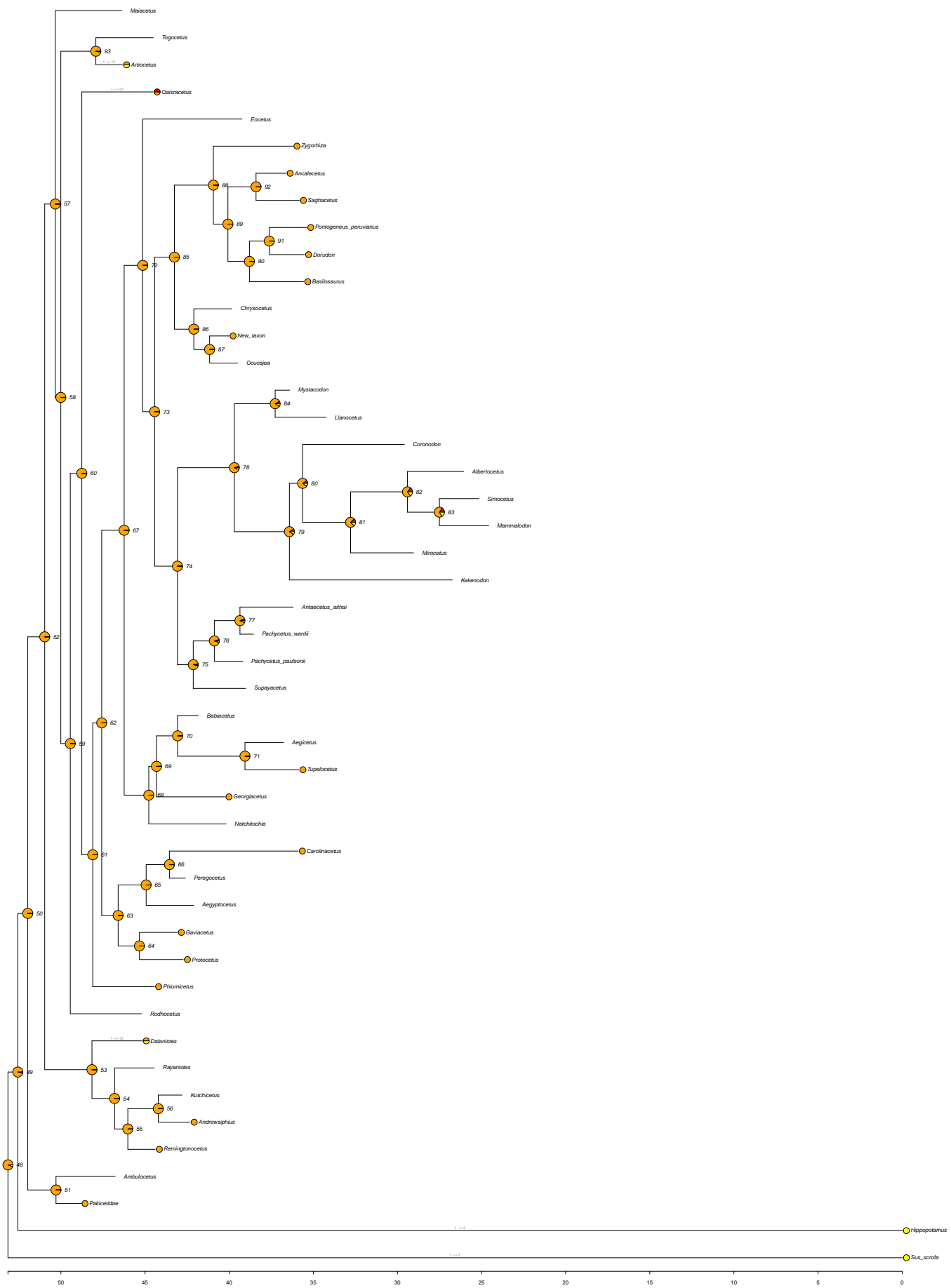

state 0 state 1 state 2

Supplement: Supplementary file 6 — Supplementary Data 3 [file 42003_2023_4986_MOESM6_ESM.zip › Supplementary Data 3/Supplementary Data 1_BTD_ASR/trait_0029_tree.plot.pdf]

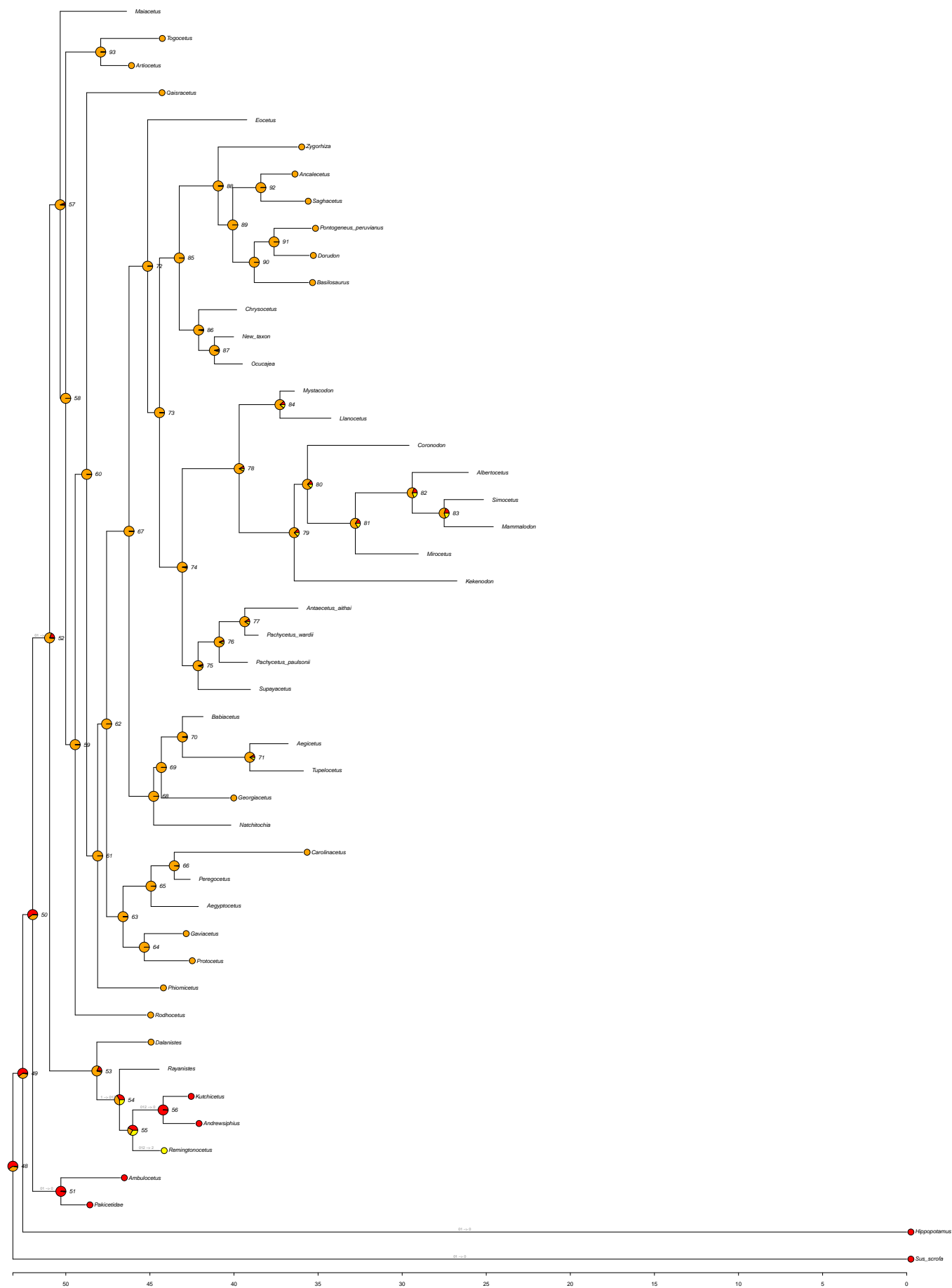

Supplement: Supplementary file 6 — Supplementary Data 3 [file 42003_2023_4986_MOESM6_ESM.zip › Supplementary Data 3/Supplementary Data 1_BTD_ASR/trait_0030_tree.plot.pdf]

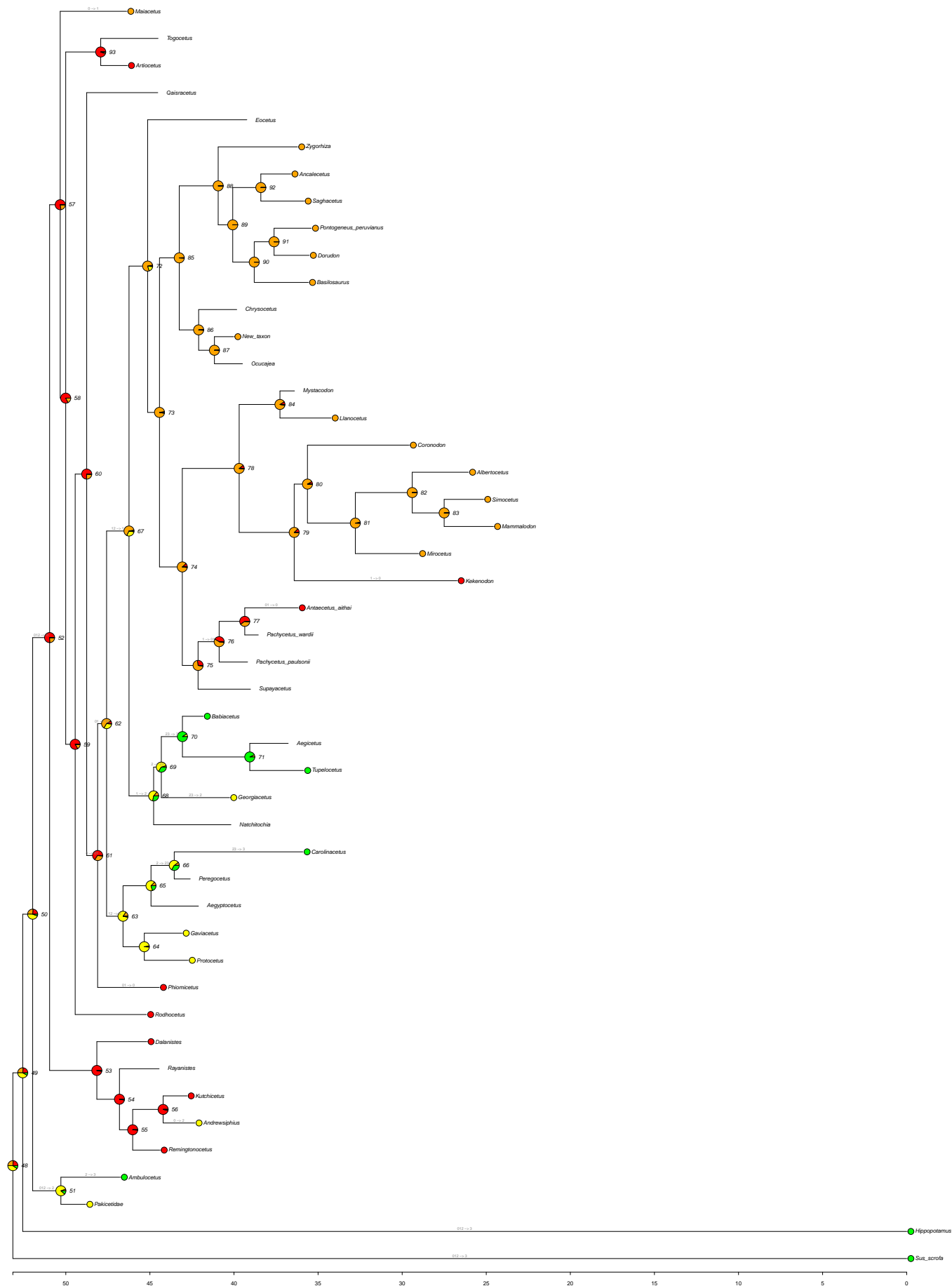

Supplement: Supplementary file 6 — Supplementary Data 3 [file 42003_2023_4986_MOESM6_ESM.zip › Supplementary Data 3/Supplementary Data 1_BTD_ASR/trait_0031_tree.plot.pdf]

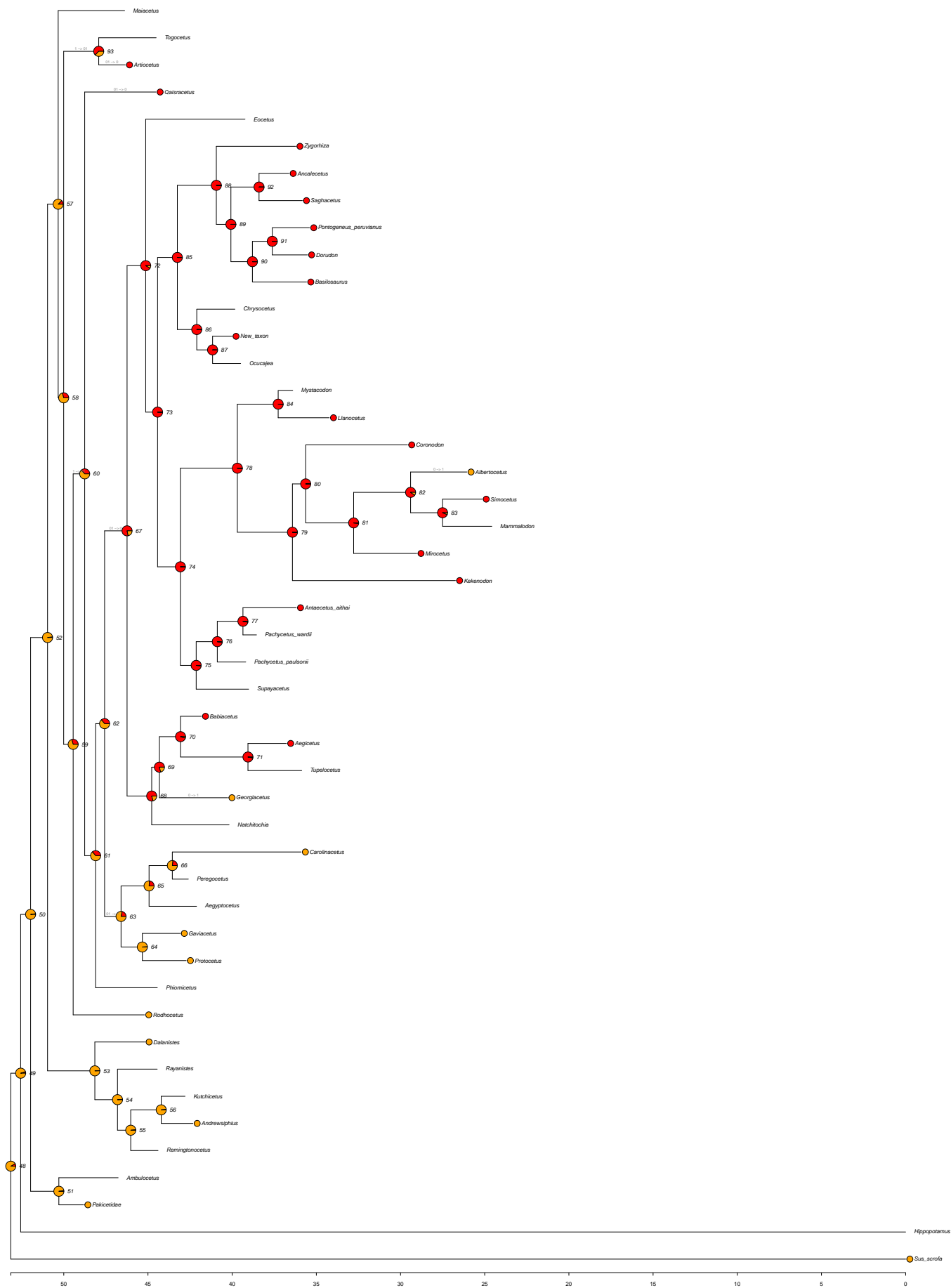

Supplement: Supplementary file 6 — Supplementary Data 3 [file 42003_2023_4986_MOESM6_ESM.zip › Supplementary Data 3/Supplementary Data 1_BTD_ASR/trait_0032_tree.plot.pdf]

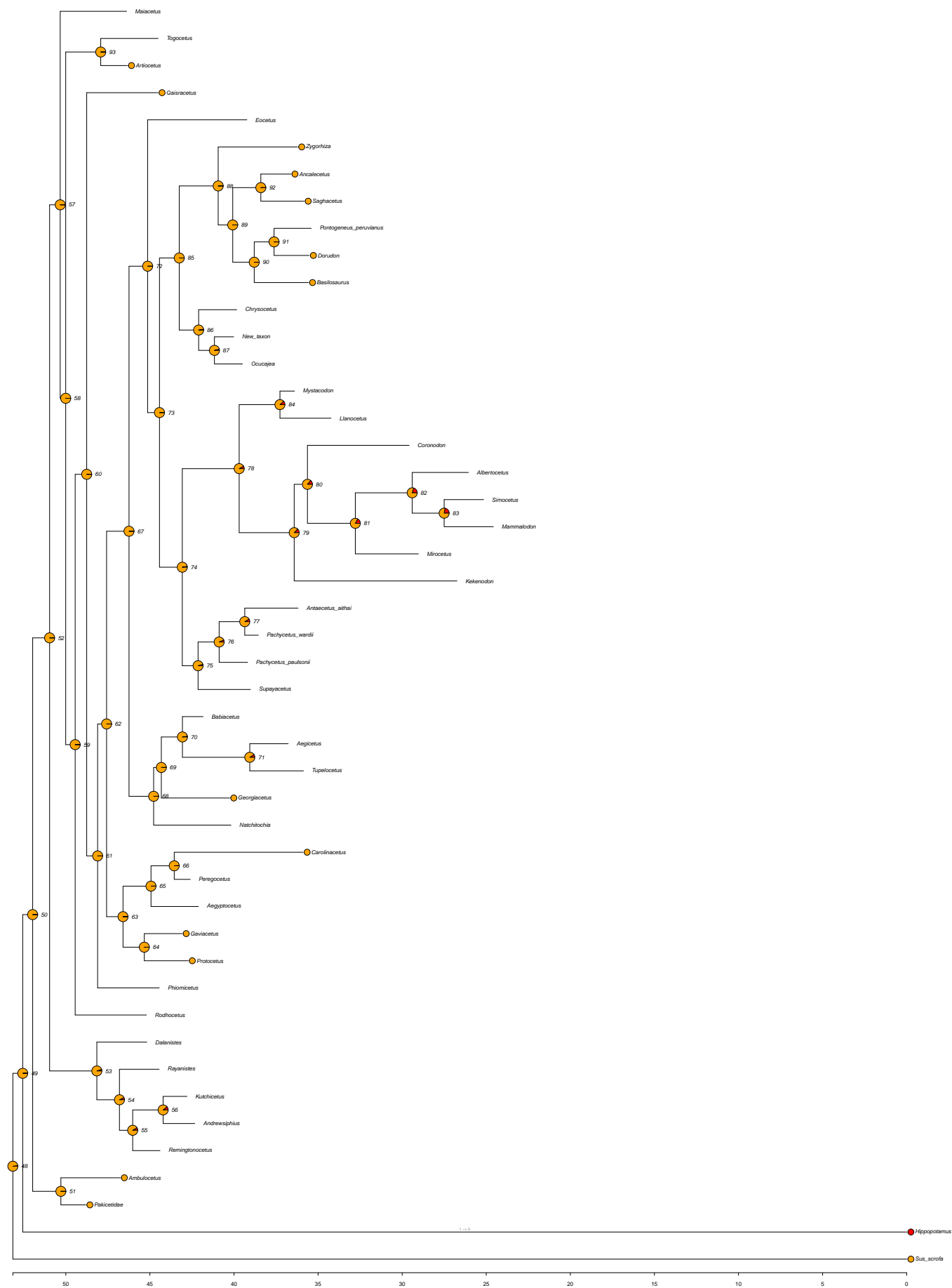

Supplement: Supplementary file 6 — Supplementary Data 3 [file 42003_2023_4986_MOESM6_ESM.zip › Supplementary Data 3/Supplementary Data 1_BTD_ASR/trait_0033_tree.plot.pdf]

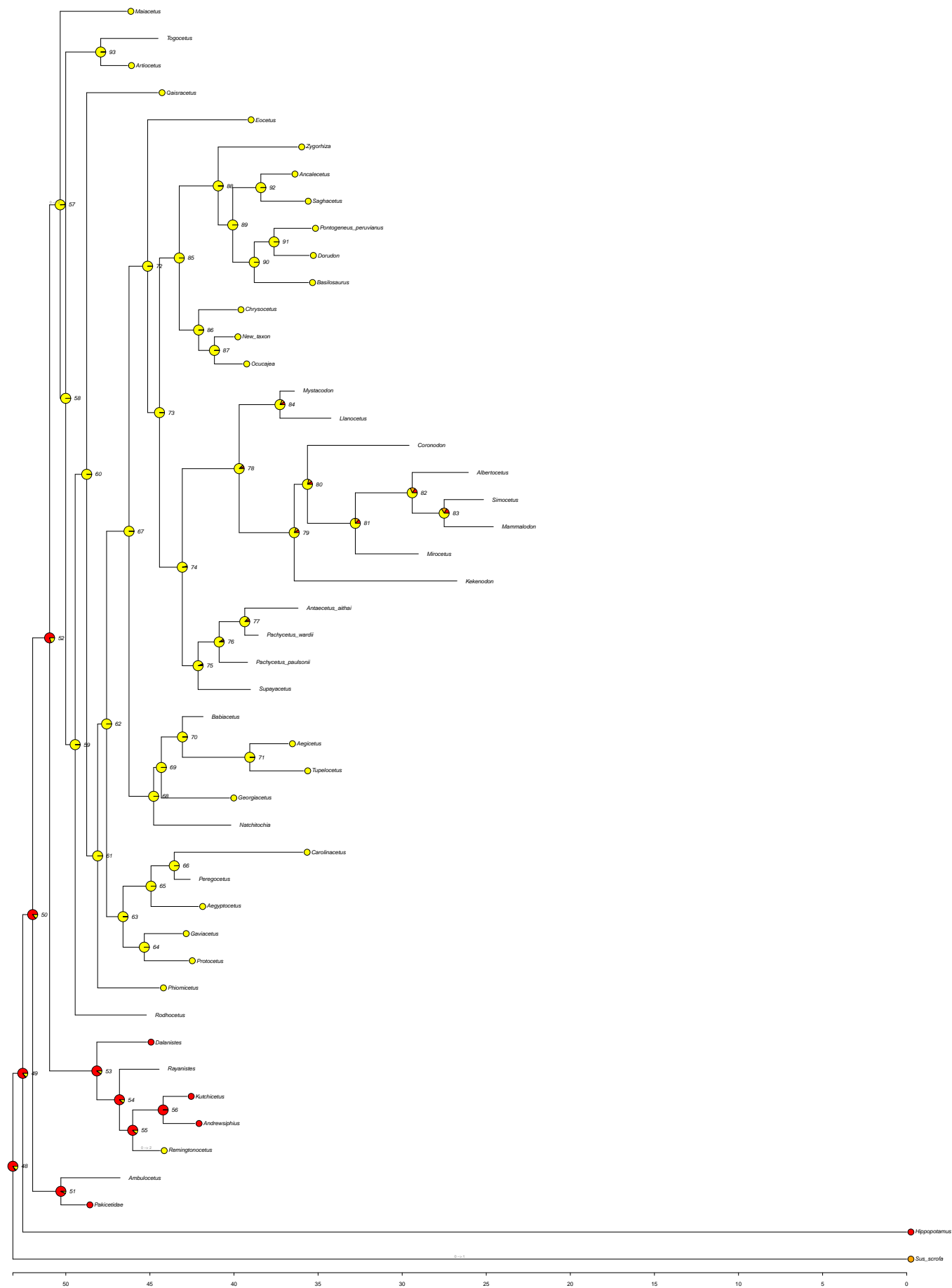

Supplement: Supplementary file 6 — Supplementary Data 3 [file 42003_2023_4986_MOESM6_ESM.zip › Supplementary Data 3/Supplementary Data 1_BTD_ASR/trait_0034_tree.plot.pdf]

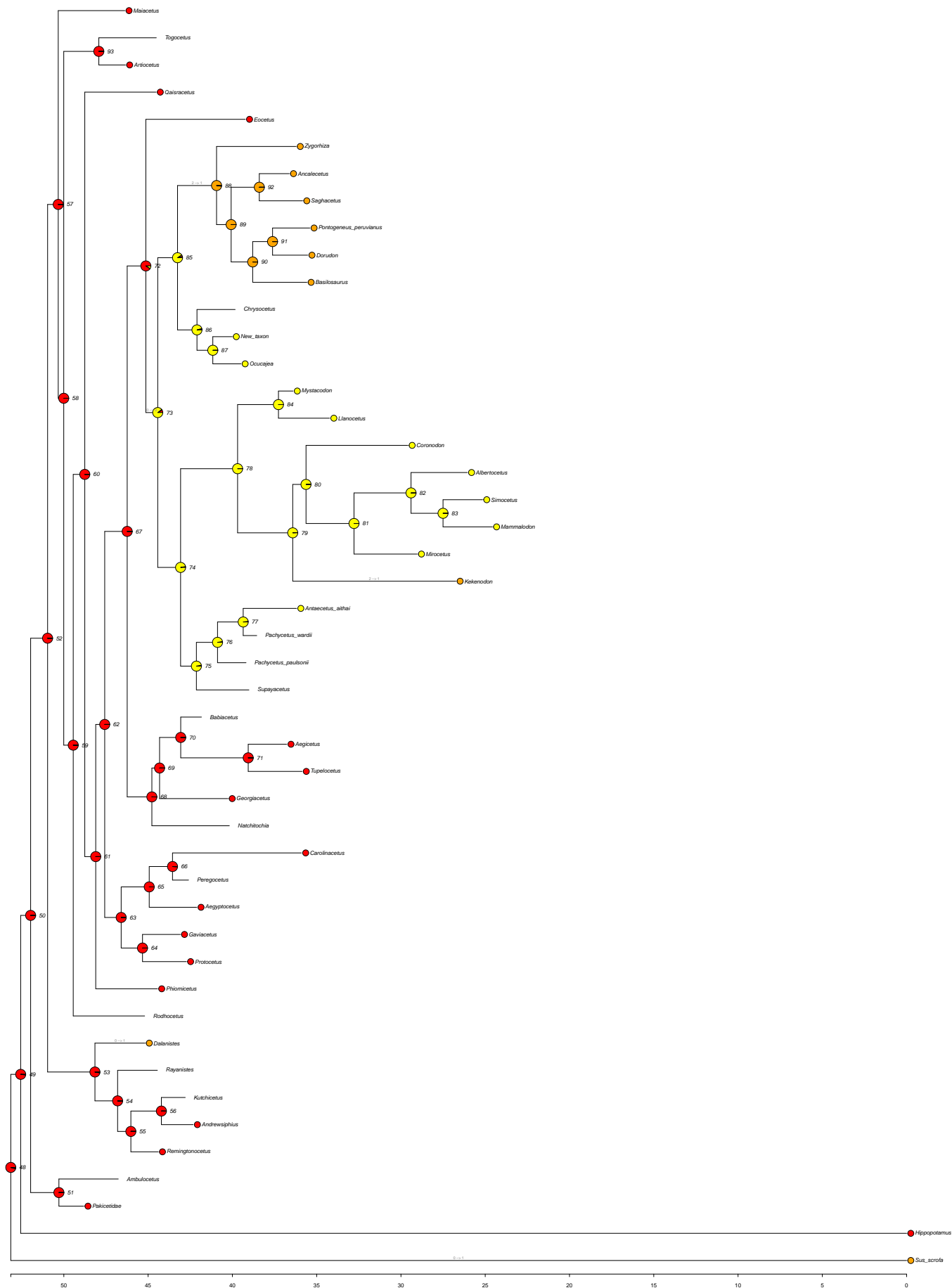

Supplement: Supplementary file 6 — Supplementary Data 3 [file 42003_2023_4986_MOESM6_ESM.zip › Supplementary Data 3/Supplementary Data 1_BTD_ASR/trait_0035_tree.plot.pdf]

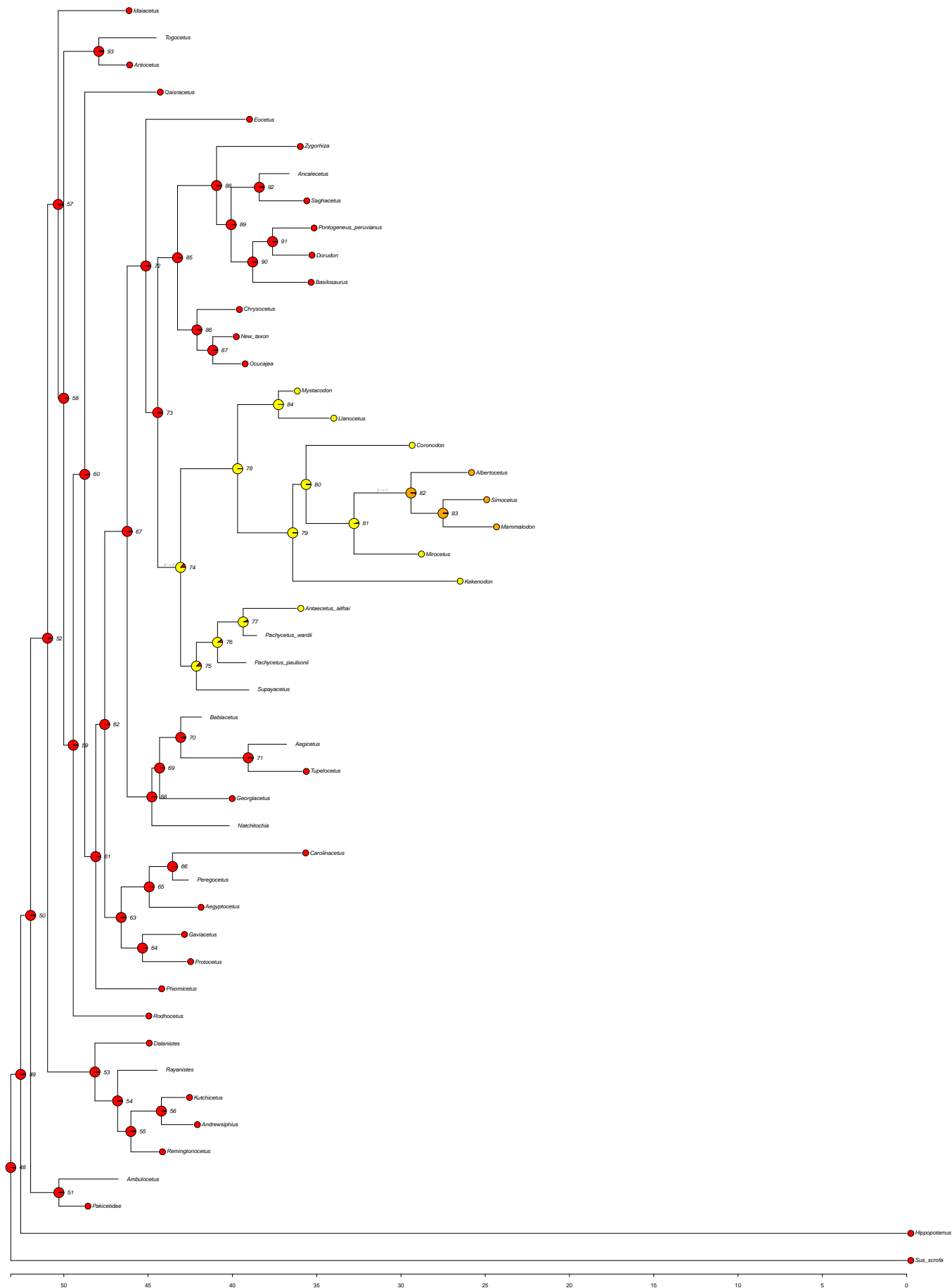

Supplement: Supplementary file 6 — Supplementary Data 3 [file 42003_2023_4986_MOESM6_ESM.zip › Supplementary Data 3/Supplementary Data 1_BTD_ASR/trait_0036_tree.plot.pdf]

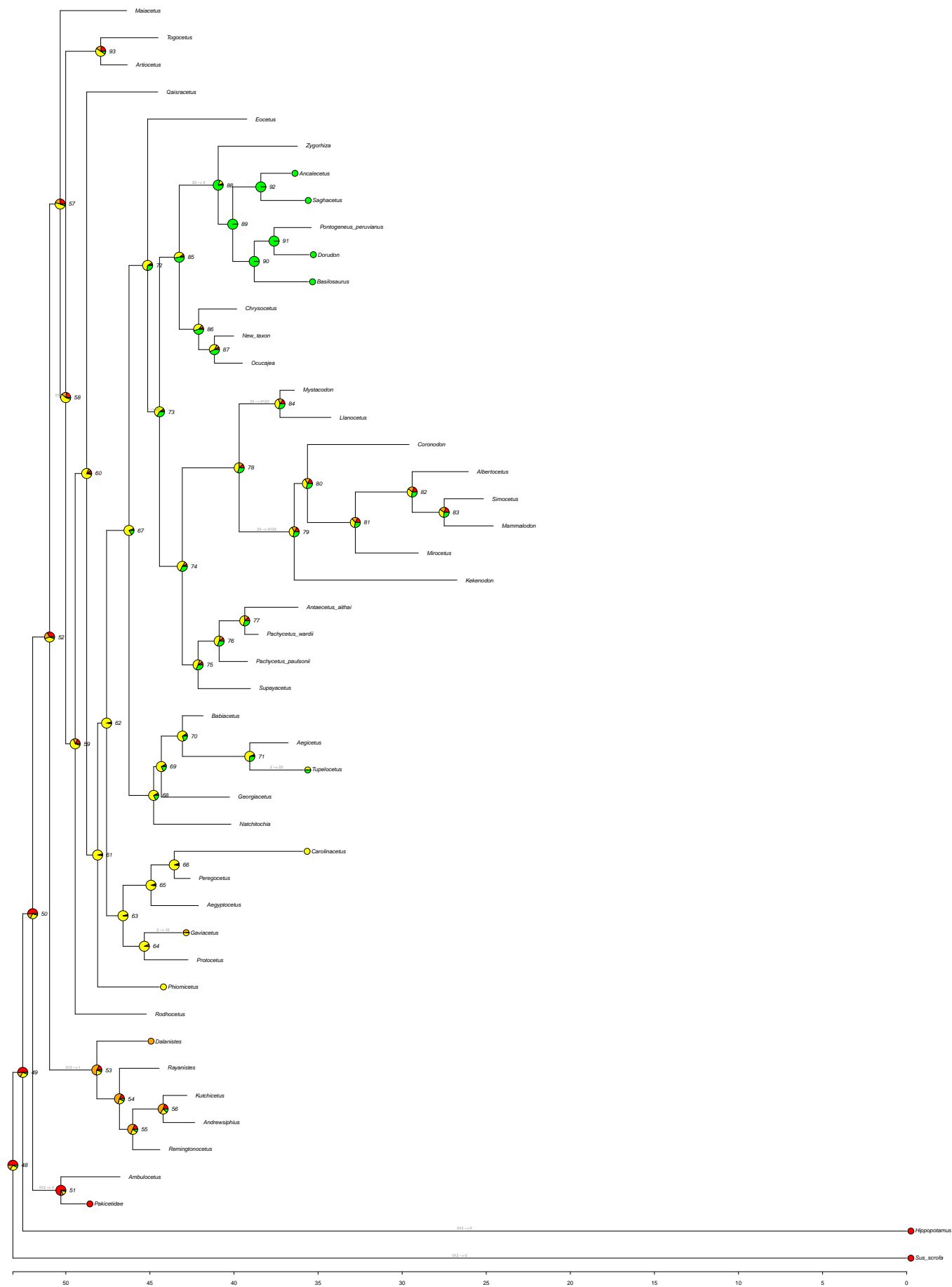

Supplement: Supplementary file 6 — Supplementary Data 3 [file 42003_2023_4986_MOESM6_ESM.zip › Supplementary Data 3/Supplementary Data 1_BTD_ASR/trait_0037_tree.plot.pdf]

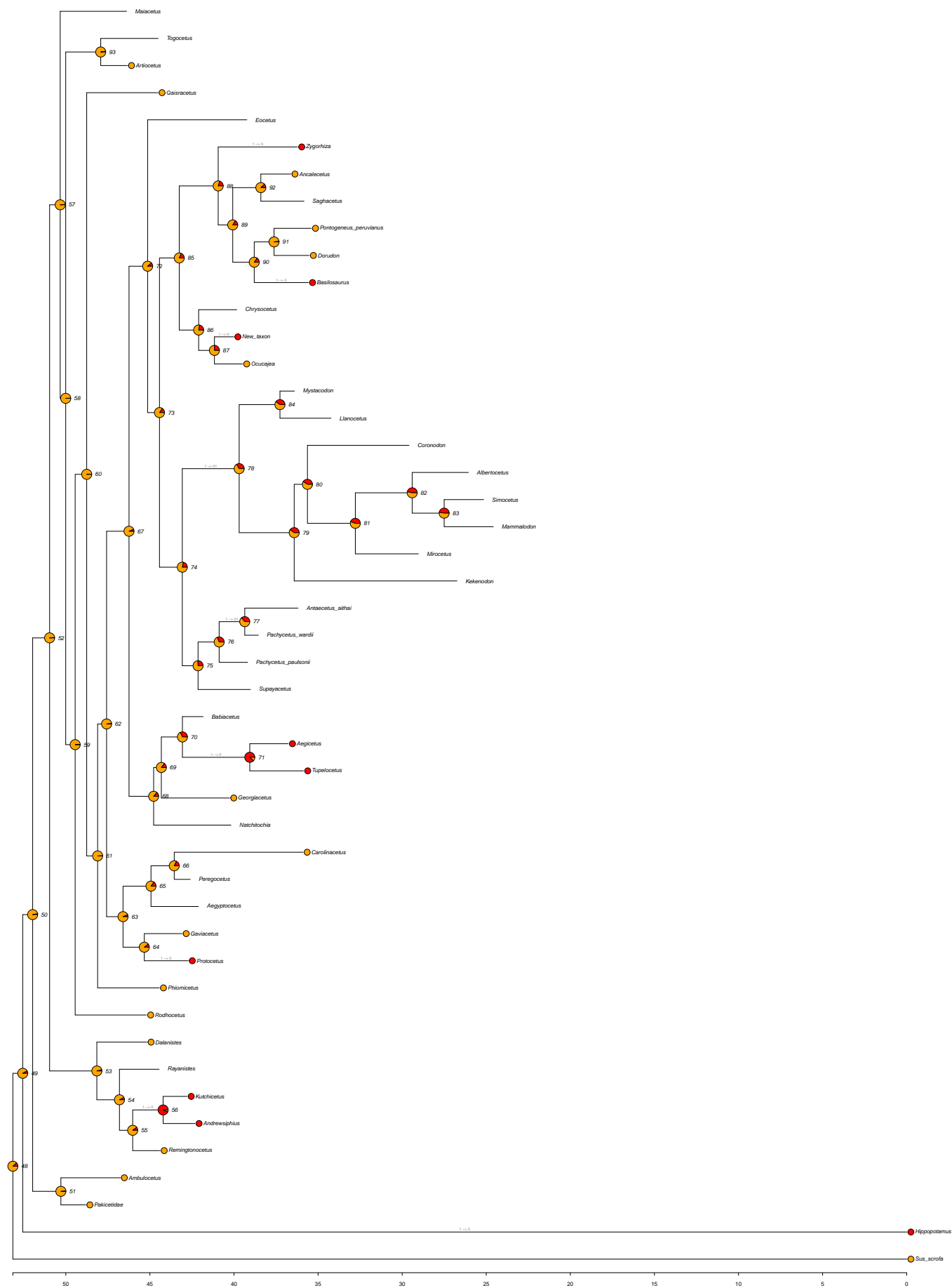

Supplement: Supplementary file 6 — Supplementary Data 3 [file 42003_2023_4986_MOESM6_ESM.zip › Supplementary Data 3/Supplementary Data 1_BTD_ASR/trait_0038_tree.plot.pdf]

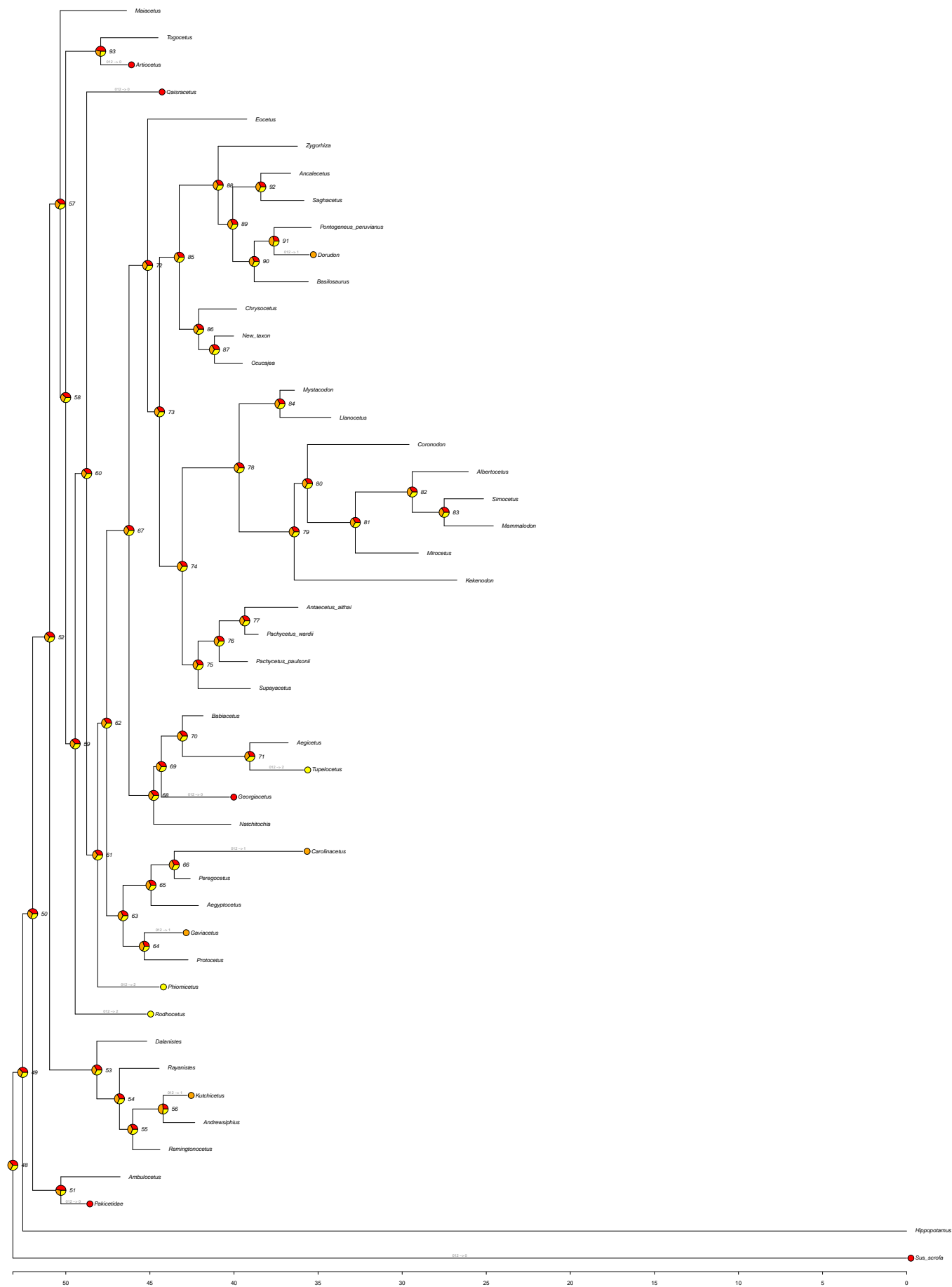

Supplement: Supplementary file 6 — Supplementary Data 3 [file 42003_2023_4986_MOESM6_ESM.zip › Supplementary Data 3/Supplementary Data 1_BTD_ASR/trait_0039_tree.plot.pdf]

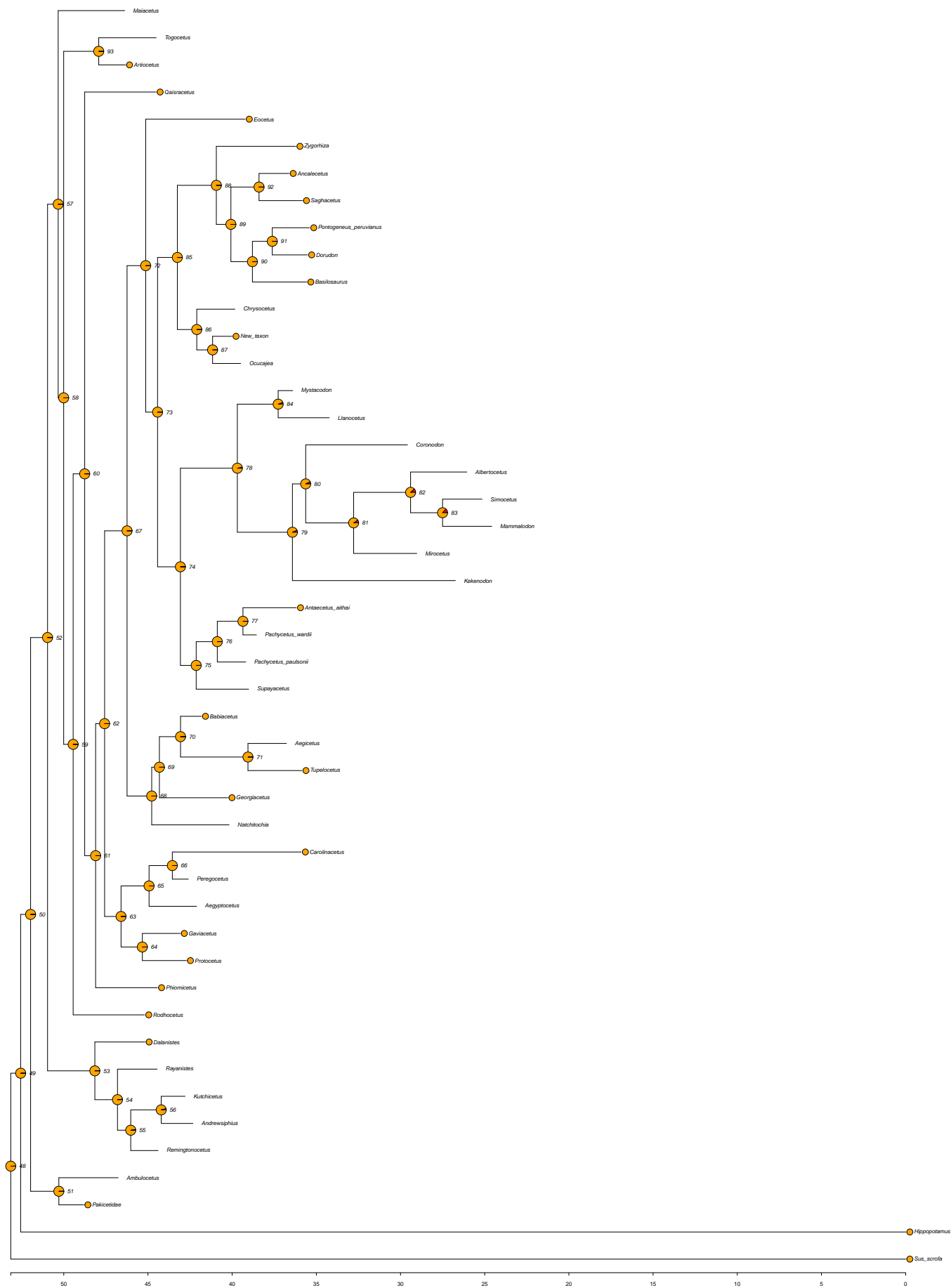

Supplement: Supplementary file 6 — Supplementary Data 3 [file 42003_2023_4986_MOESM6_ESM.zip › Supplementary Data 3/Supplementary Data 1_BTD_ASR/trait_0040_tree.plot.pdf]

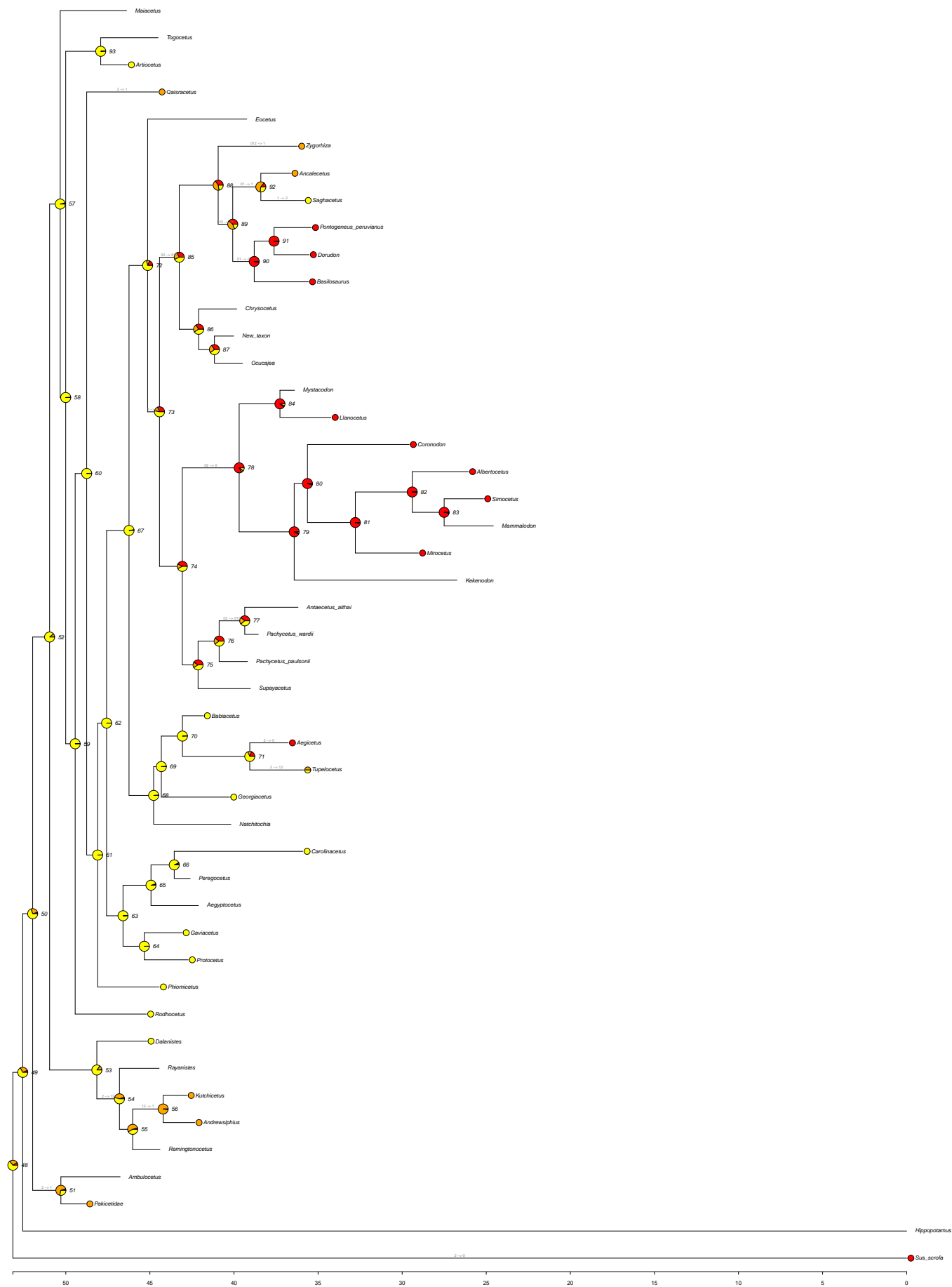

Supplement: Supplementary file 6 — Supplementary Data 3 [file 42003_2023_4986_MOESM6_ESM.zip › Supplementary Data 3/Supplementary Data 1_BTD_ASR/trait_0041_tree.plot.pdf]

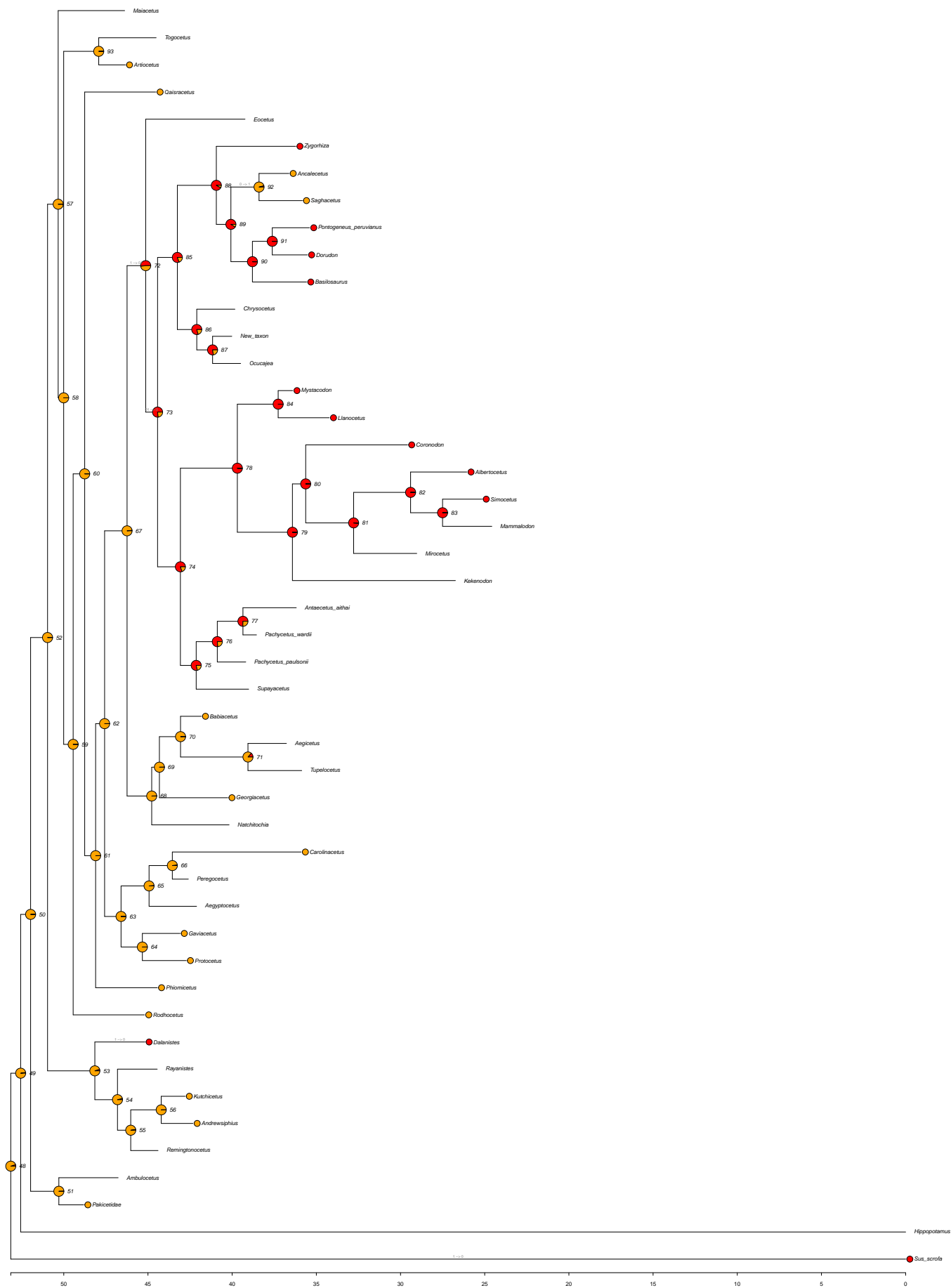

Supplement: Supplementary file 6 — Supplementary Data 3 [file 42003_2023_4986_MOESM6_ESM.zip › Supplementary Data 3/Supplementary Data 1_BTD_ASR/trait_0042_tree.plot.pdf]

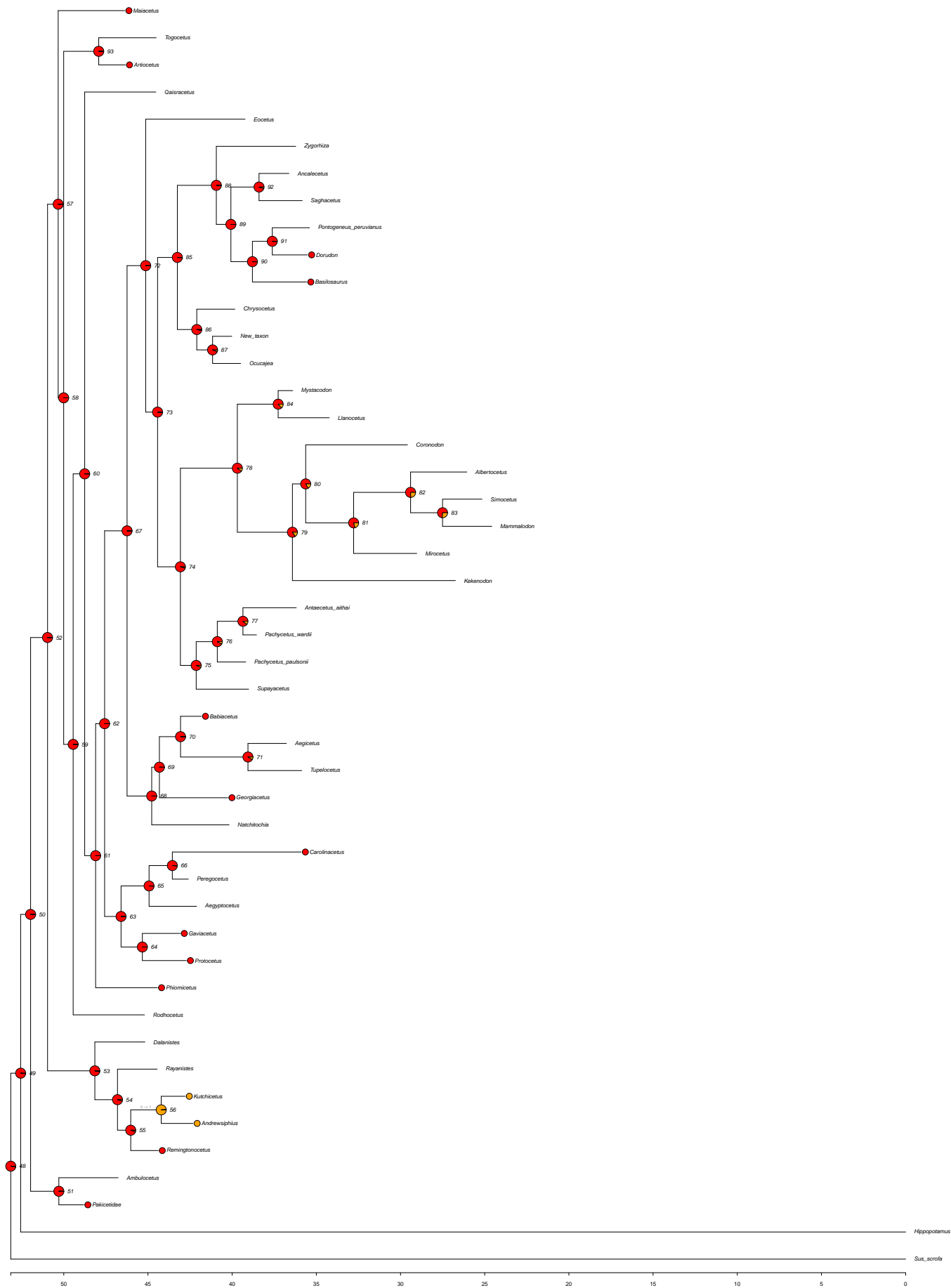

Supplement: Supplementary file 6 — Supplementary Data 3 [file 42003_2023_4986_MOESM6_ESM.zip › Supplementary Data 3/Supplementary Data 1_BTD_ASR/trait_0043_tree.plot.pdf]

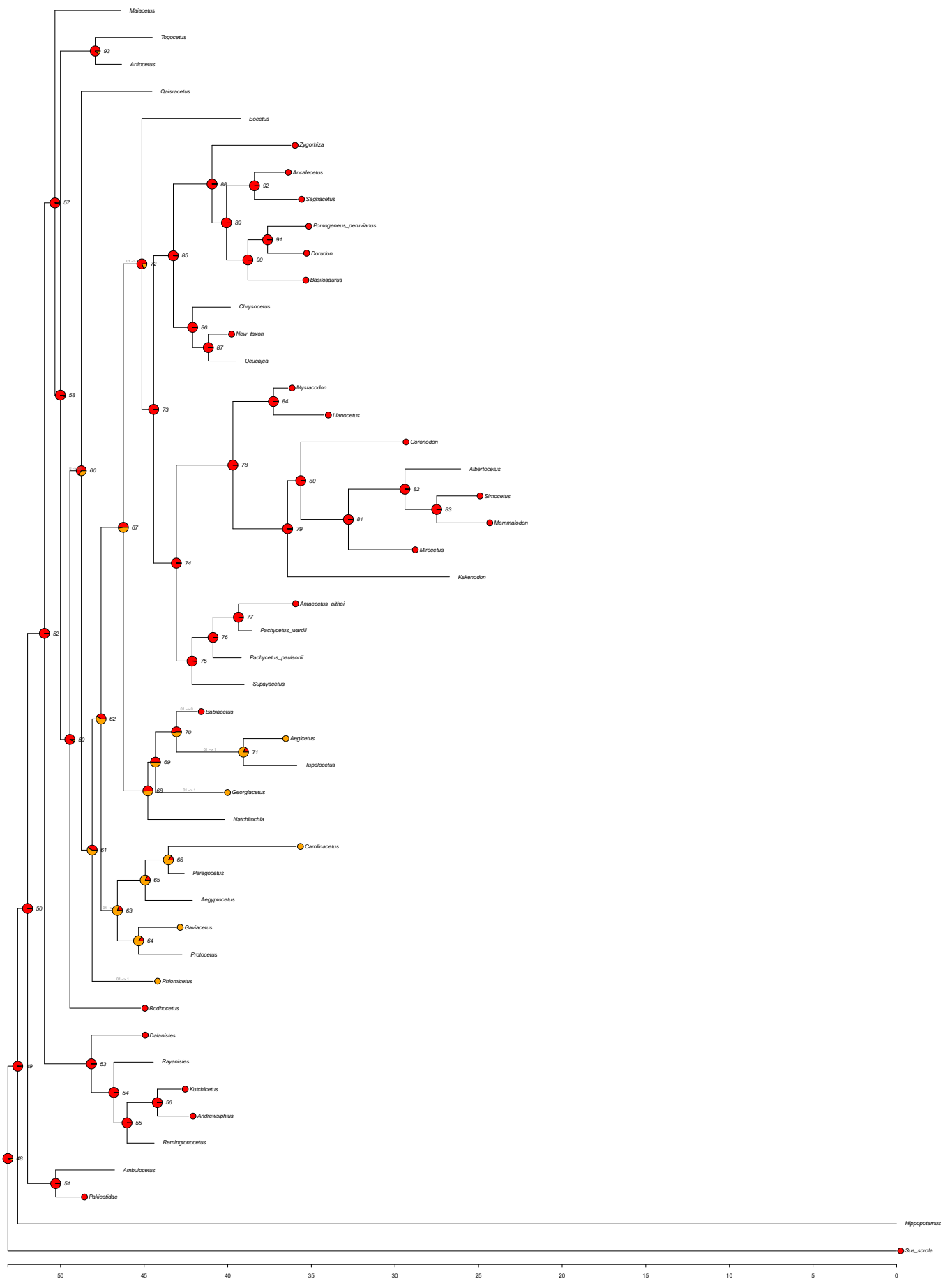

Supplement: Supplementary file 6 — Supplementary Data 3 [file 42003_2023_4986_MOESM6_ESM.zip › Supplementary Data 3/Supplementary Data 1_BTD_ASR/trait_0044_tree.plot.pdf]

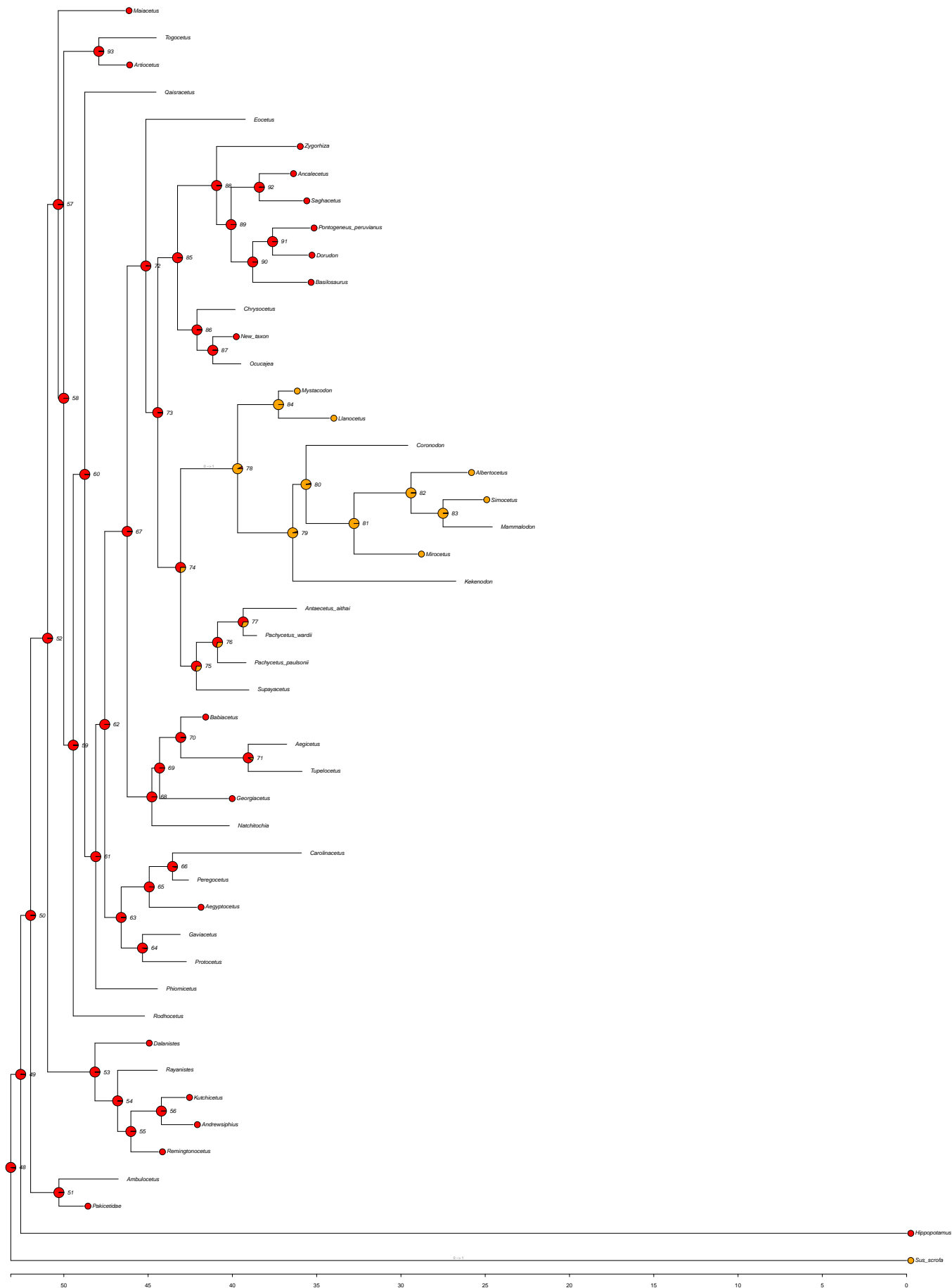

Supplement: Supplementary file 6 — Supplementary Data 3 [file 42003_2023_4986_MOESM6_ESM.zip › Supplementary Data 3/Supplementary Data 1_BTD_ASR/trait_0045_tree.plot.pdf]

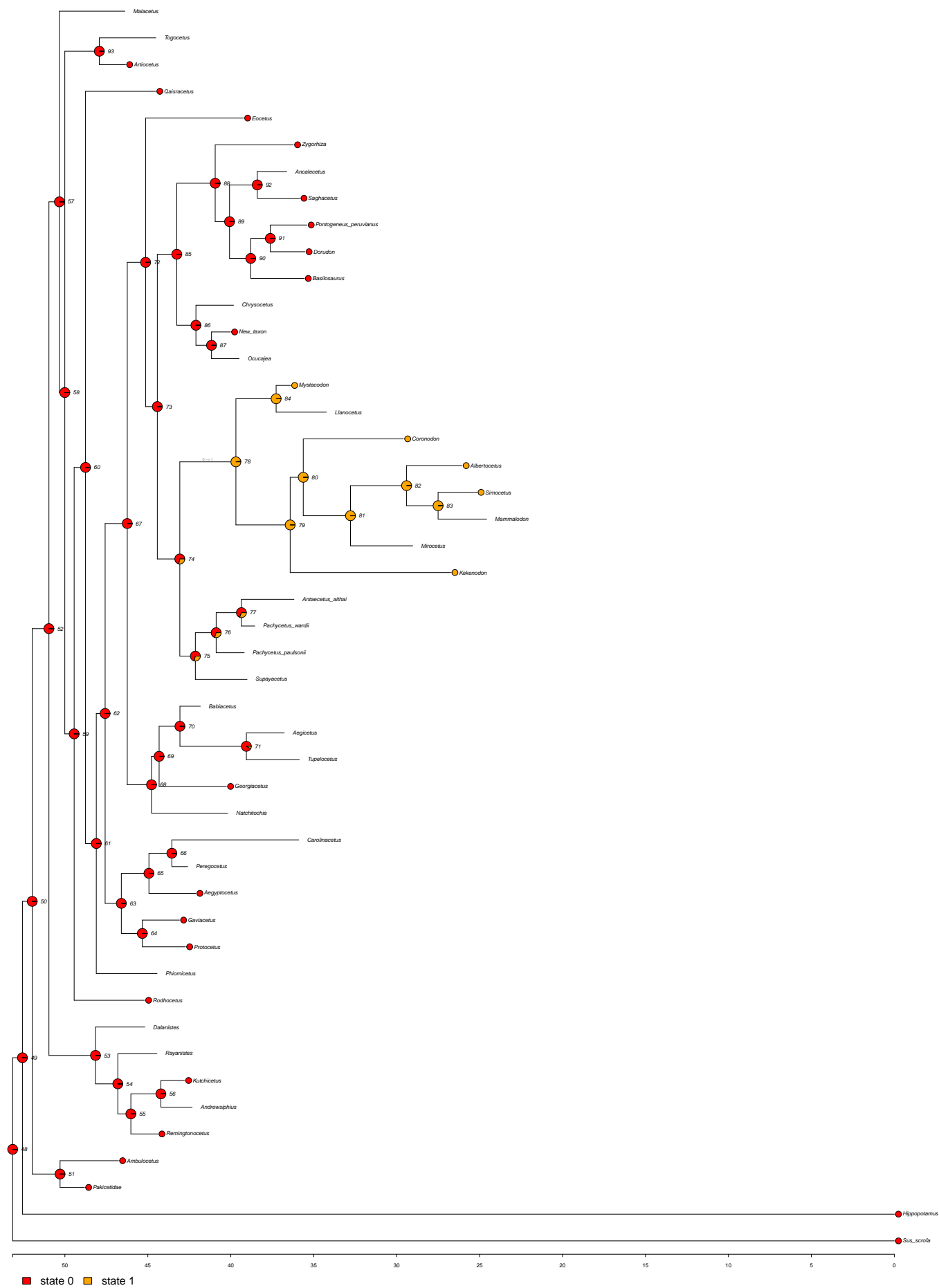

Supplement: Supplementary file 6 — Supplementary Data 3 [file 42003_2023_4986_MOESM6_ESM.zip › Supplementary Data 3/Supplementary Data 1_BTD_ASR/trait_0046_tree.plot.pdf]

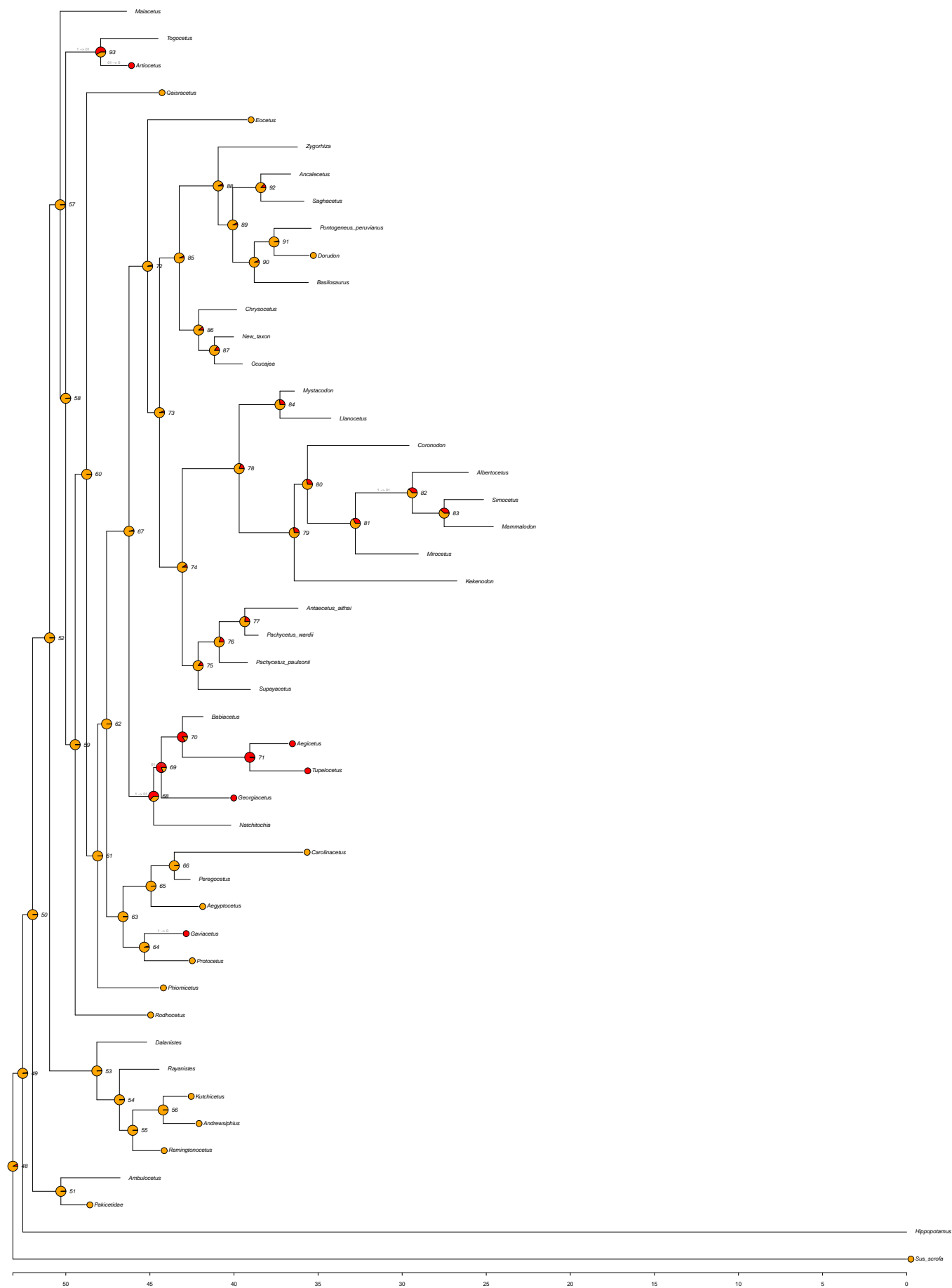

Supplement: Supplementary file 6 — Supplementary Data 3 [file 42003_2023_4986_MOESM6_ESM.zip › Supplementary Data 3/Supplementary Data 1_BTD_ASR/trait_0047_tree.plot.pdf]

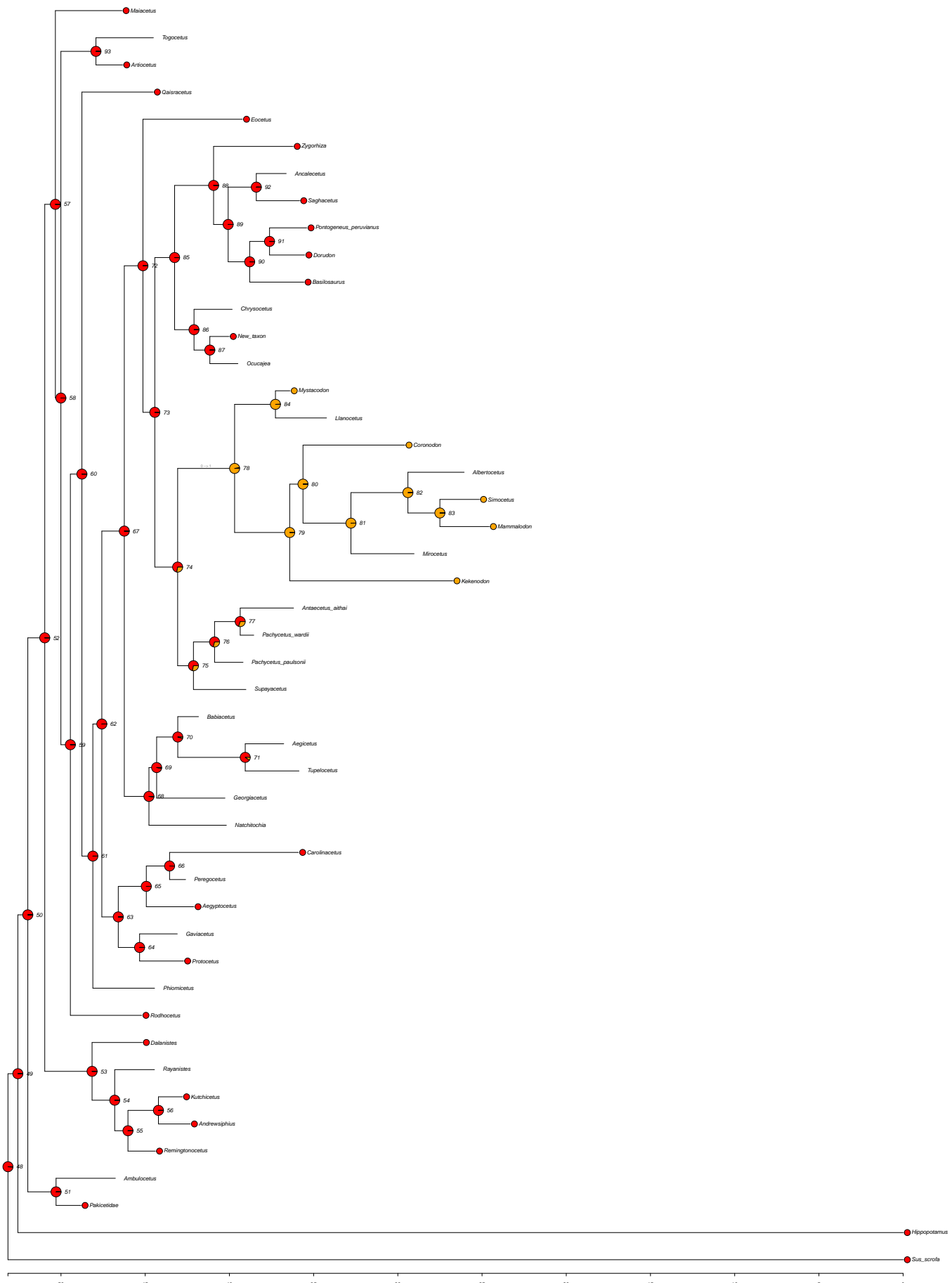

state 0 state 1

Supplement: Supplementary file 6 — Supplementary Data 3 [file 42003_2023_4986_MOESM6_ESM.zip › Supplementary Data 3/Supplementary Data 1_BTD_ASR/trait_0048_tree.plot.pdf]

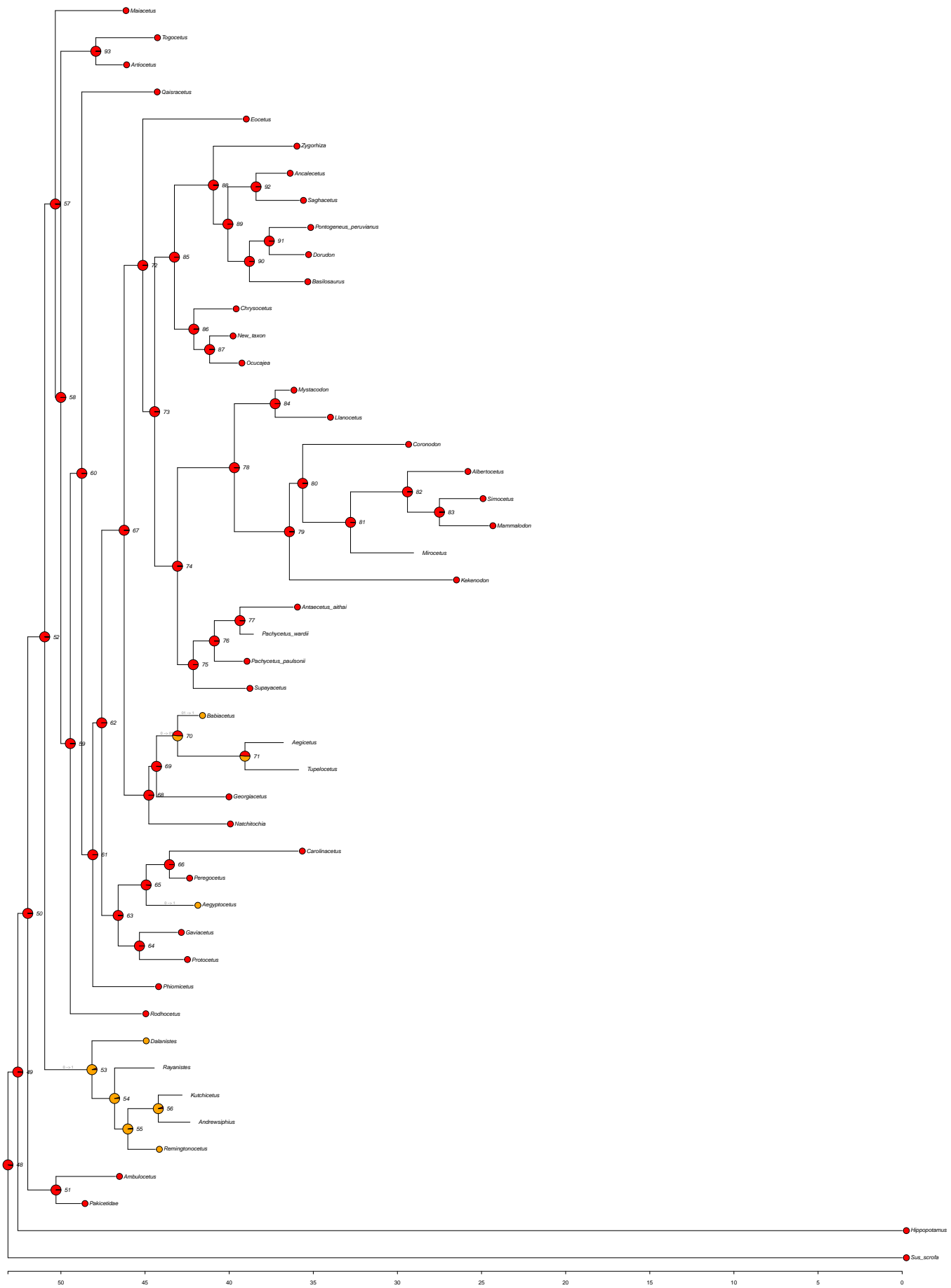

Supplement: Supplementary file 6 — Supplementary Data 3 [file 42003_2023_4986_MOESM6_ESM.zip › Supplementary Data 3/Supplementary Data 1_BTD_ASR/trait_0049_tree.plot.pdf]

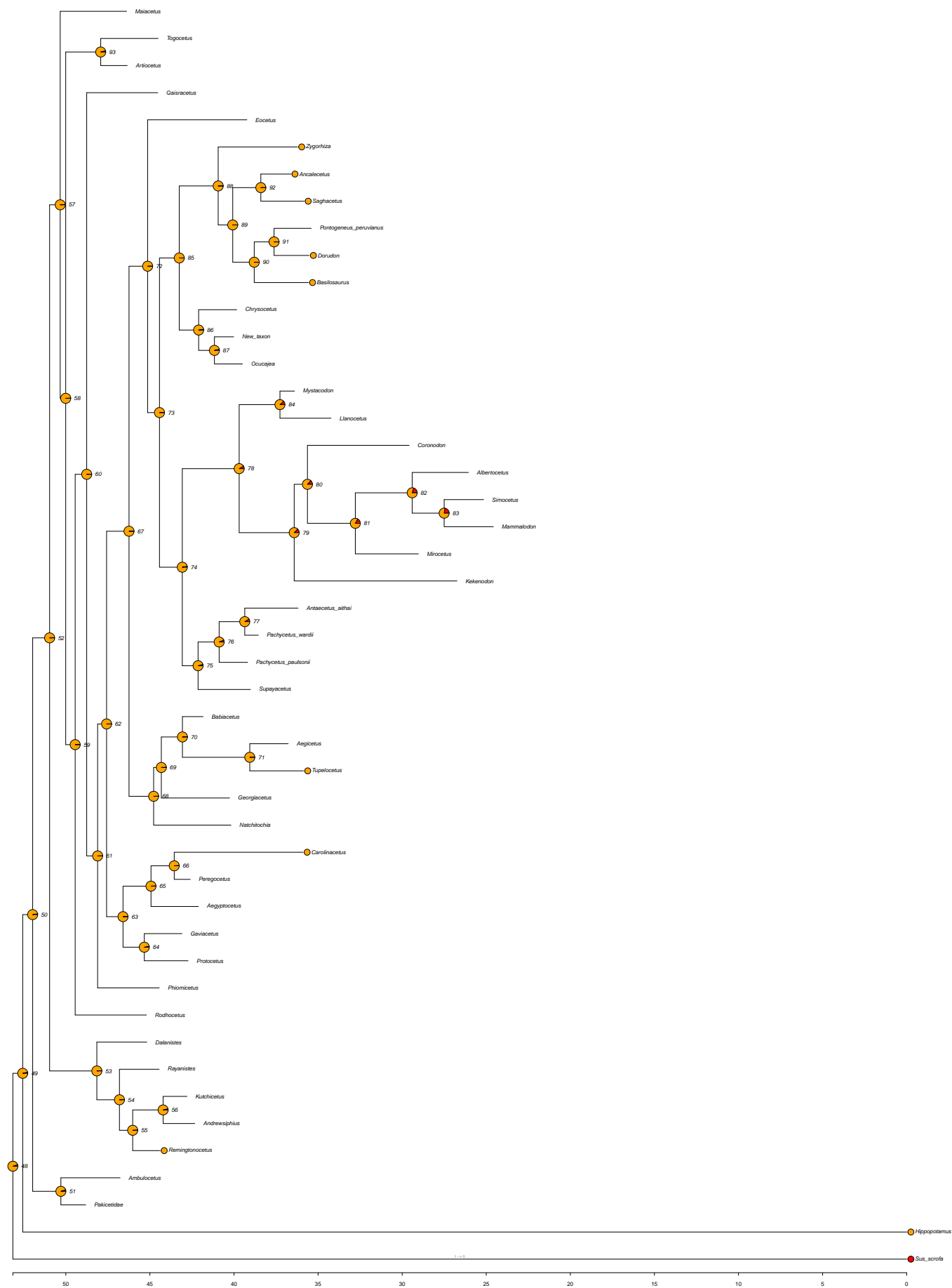

Supplement: Supplementary file 6 — Supplementary Data 3 [file 42003_2023_4986_MOESM6_ESM.zip › Supplementary Data 3/Supplementary Data 1_BTD_ASR/trait_0050_tree.plot.pdf]

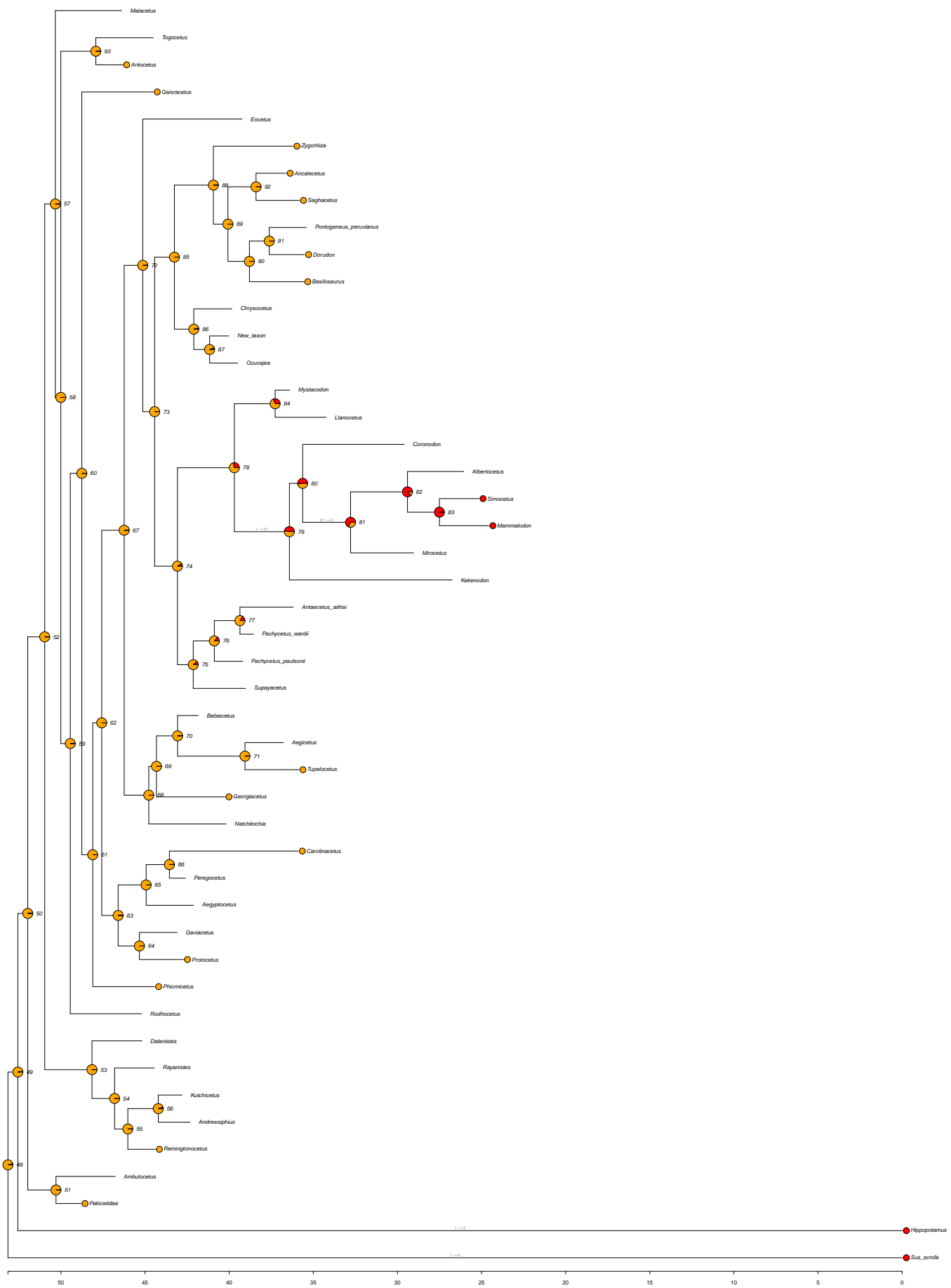

state 0 state 1

Supplement: Supplementary file 6 — Supplementary Data 3 [file 42003_2023_4986_MOESM6_ESM.zip › Supplementary Data 3/Supplementary Data 1_BTD_ASR/trait_0051_tree.plot.pdf]

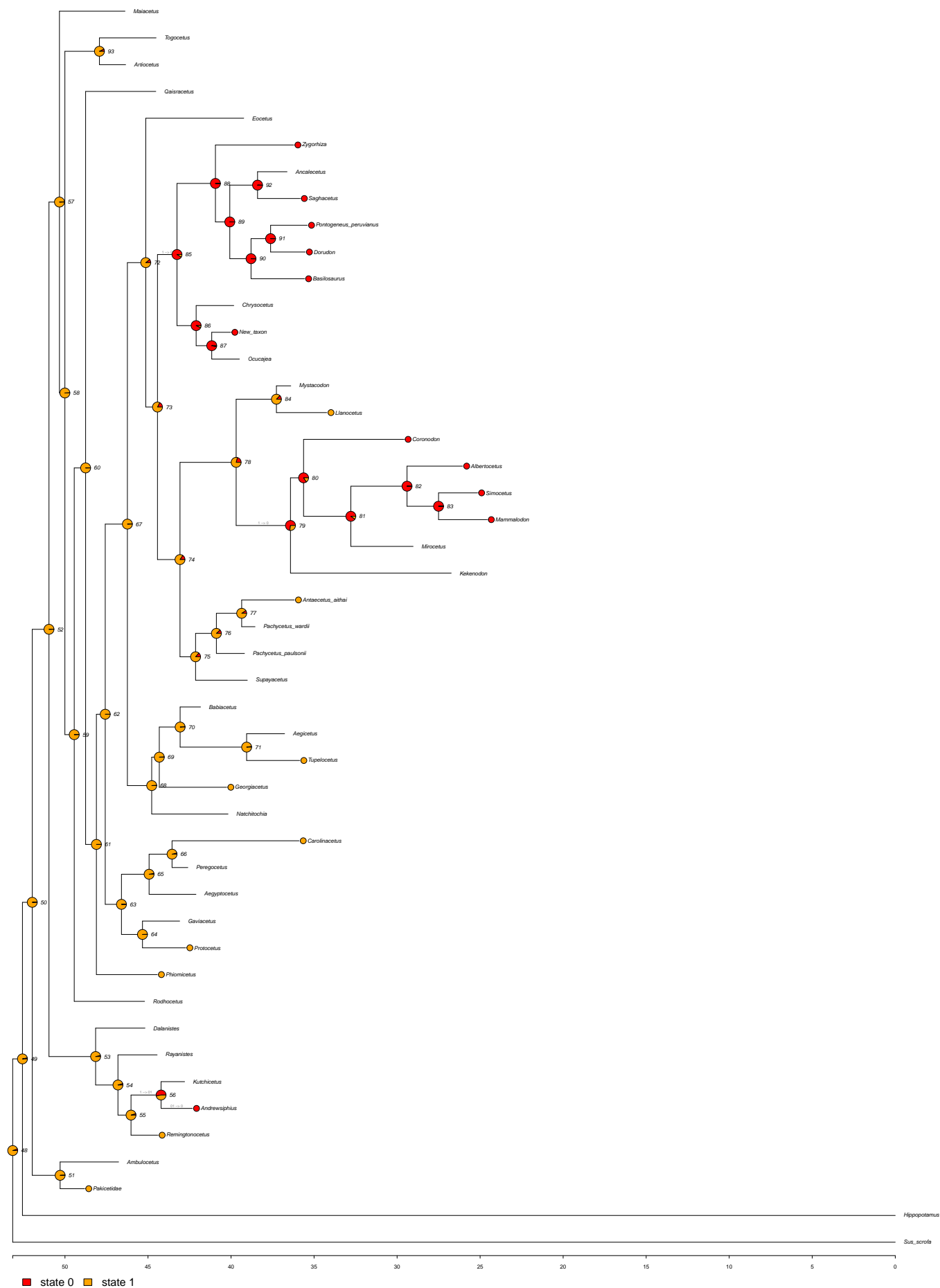

Supplement: Supplementary file 6 — Supplementary Data 3 [file 42003_2023_4986_MOESM6_ESM.zip › Supplementary Data 3/Supplementary Data 1_BTD_ASR/trait_0052_tree.plot.pdf]

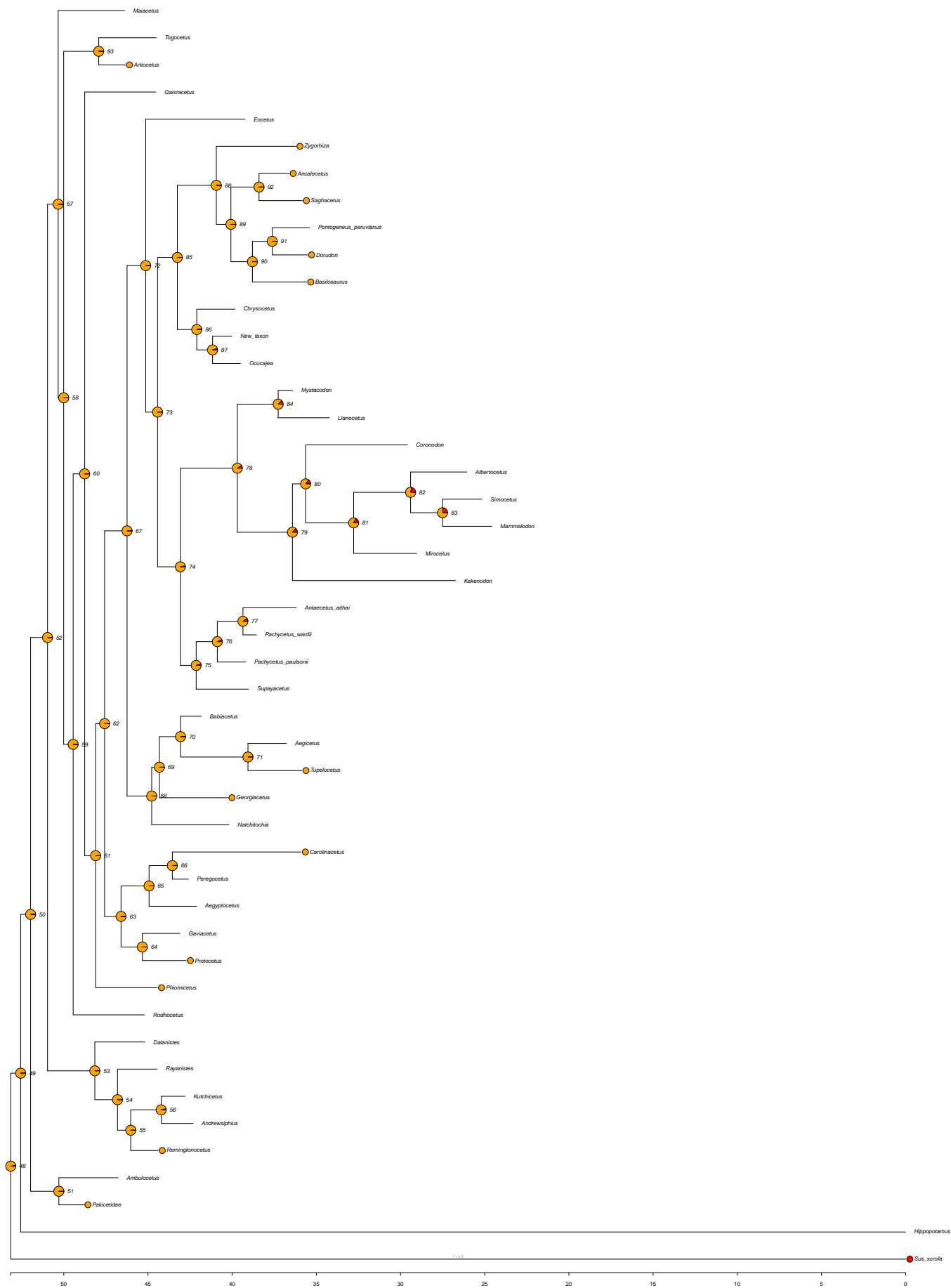

Supplement: Supplementary file 6 — Supplementary Data 3 [file 42003_2023_4986_MOESM6_ESM.zip › Supplementary Data 3/Supplementary Data 1_BTD_ASR/trait_0053_tree.plot.pdf]

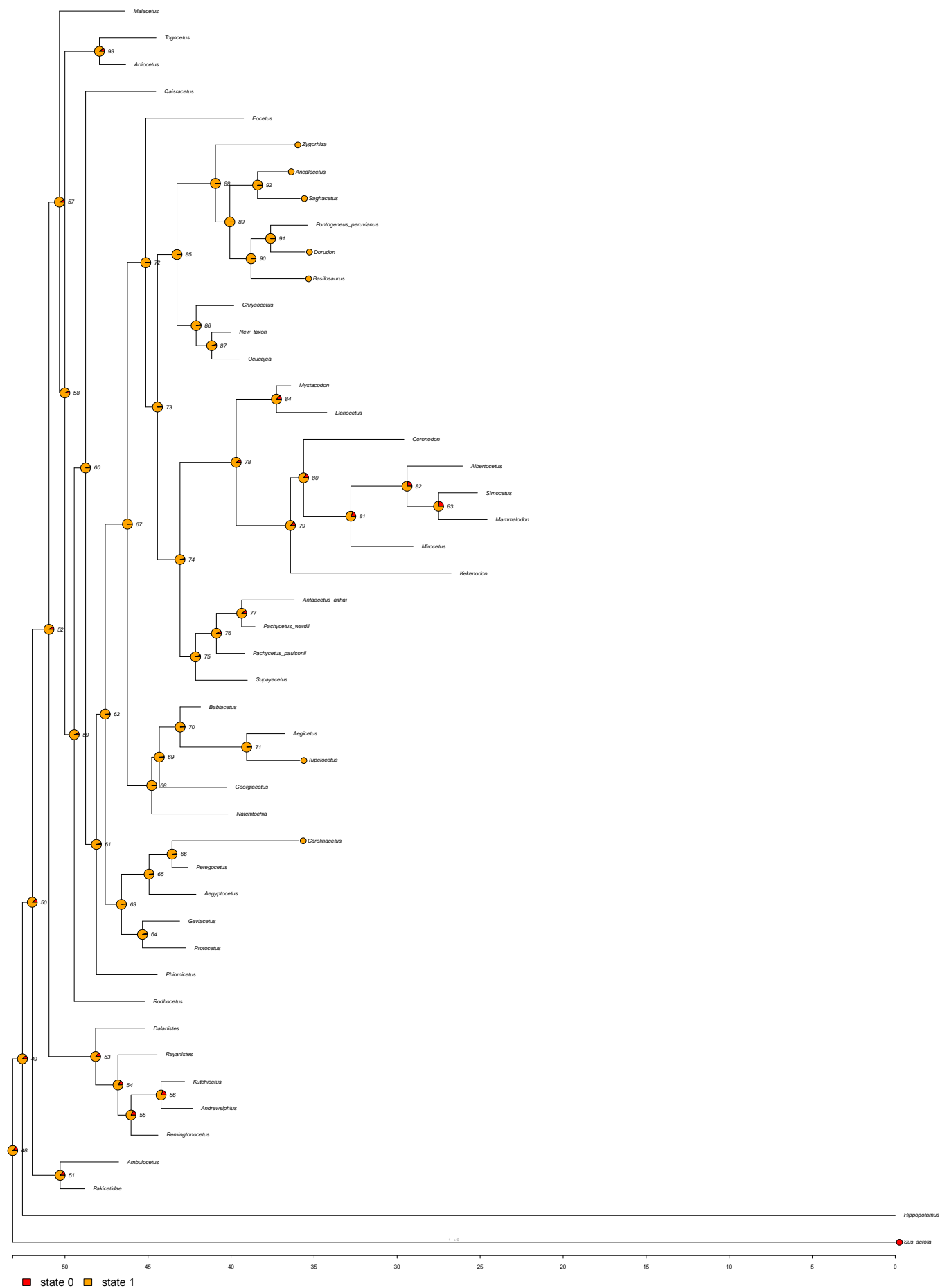

Supplement: Supplementary file 6 — Supplementary Data 3 [file 42003_2023_4986_MOESM6_ESM.zip › Supplementary Data 3/Supplementary Data 1_BTD_ASR/trait_0054_tree.plot.pdf]

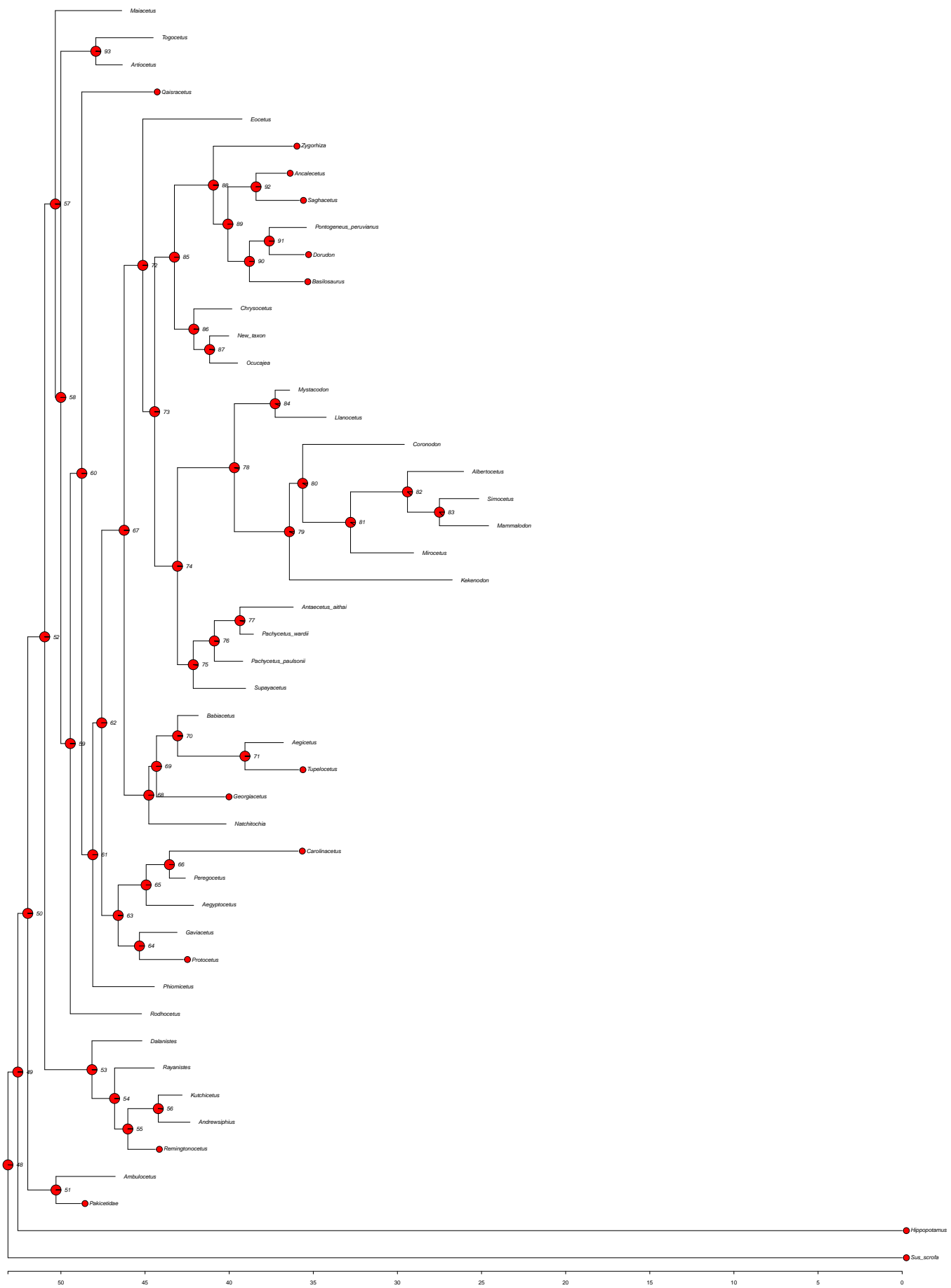

Supplement: Supplementary file 6 — Supplementary Data 3 [file 42003_2023_4986_MOESM6_ESM.zip › Supplementary Data 3/Supplementary Data 1_BTD_ASR/trait_0055_tree.plot.pdf]

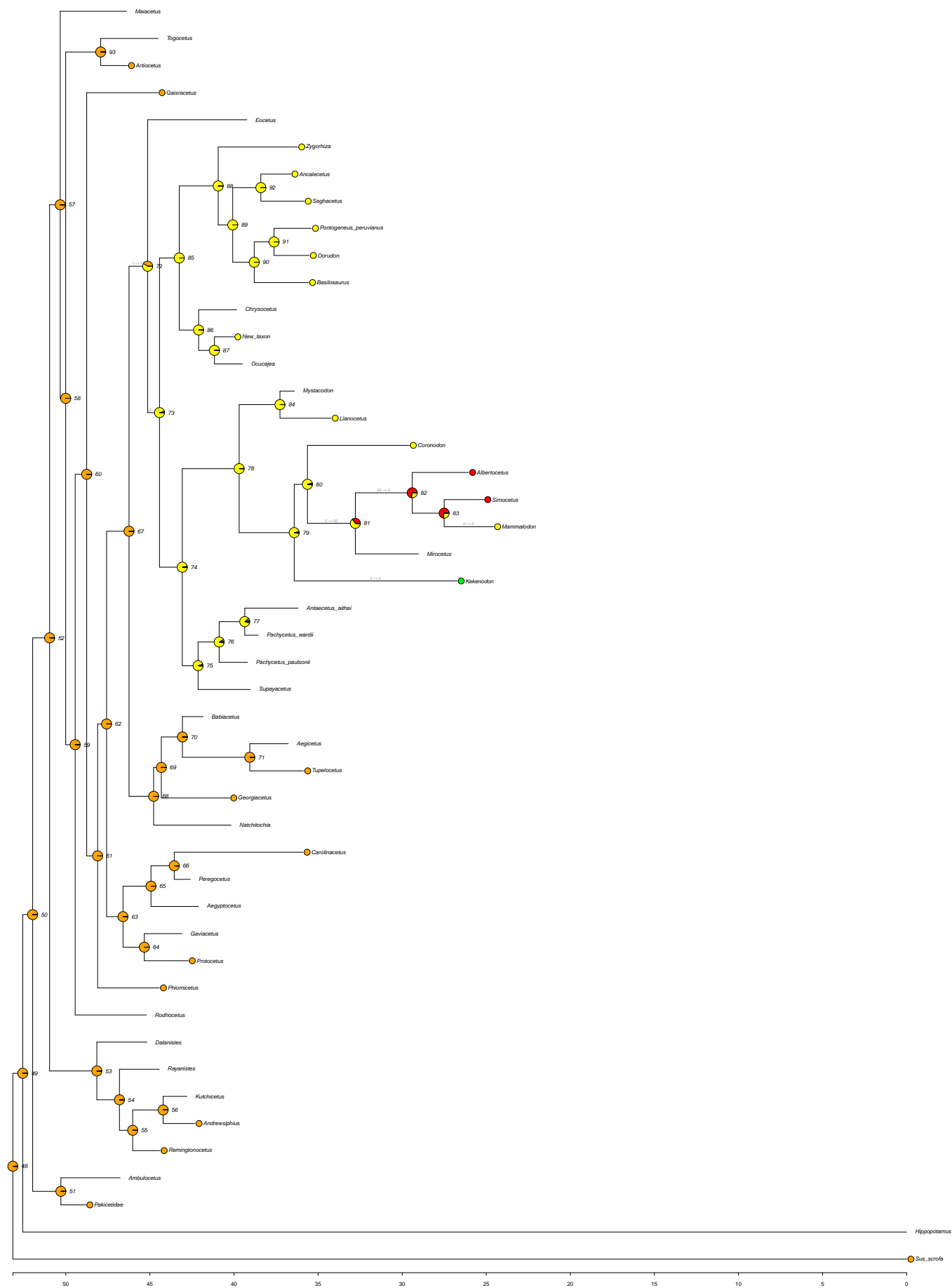

Supplement: Supplementary file 6 — Supplementary Data 3 [file 42003_2023_4986_MOESM6_ESM.zip › Supplementary Data 3/Supplementary Data 1_BTD_ASR/trait_0056_tree.plot.pdf]

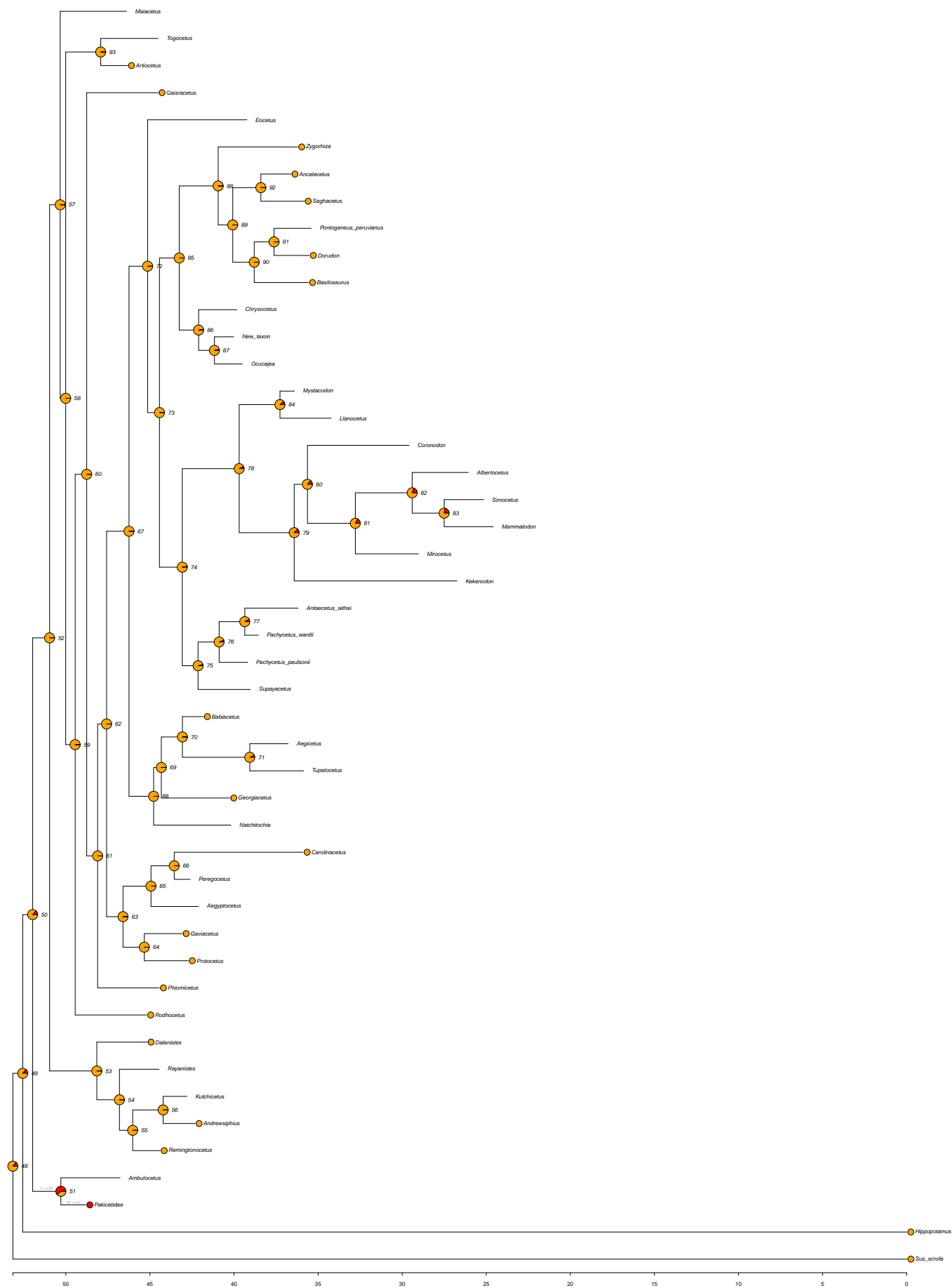

Supplement: Supplementary file 6 — Supplementary Data 3 [file 42003_2023_4986_MOESM6_ESM.zip › Supplementary Data 3/Supplementary Data 1_BTD_ASR/trait_0057_tree.plot.pdf]

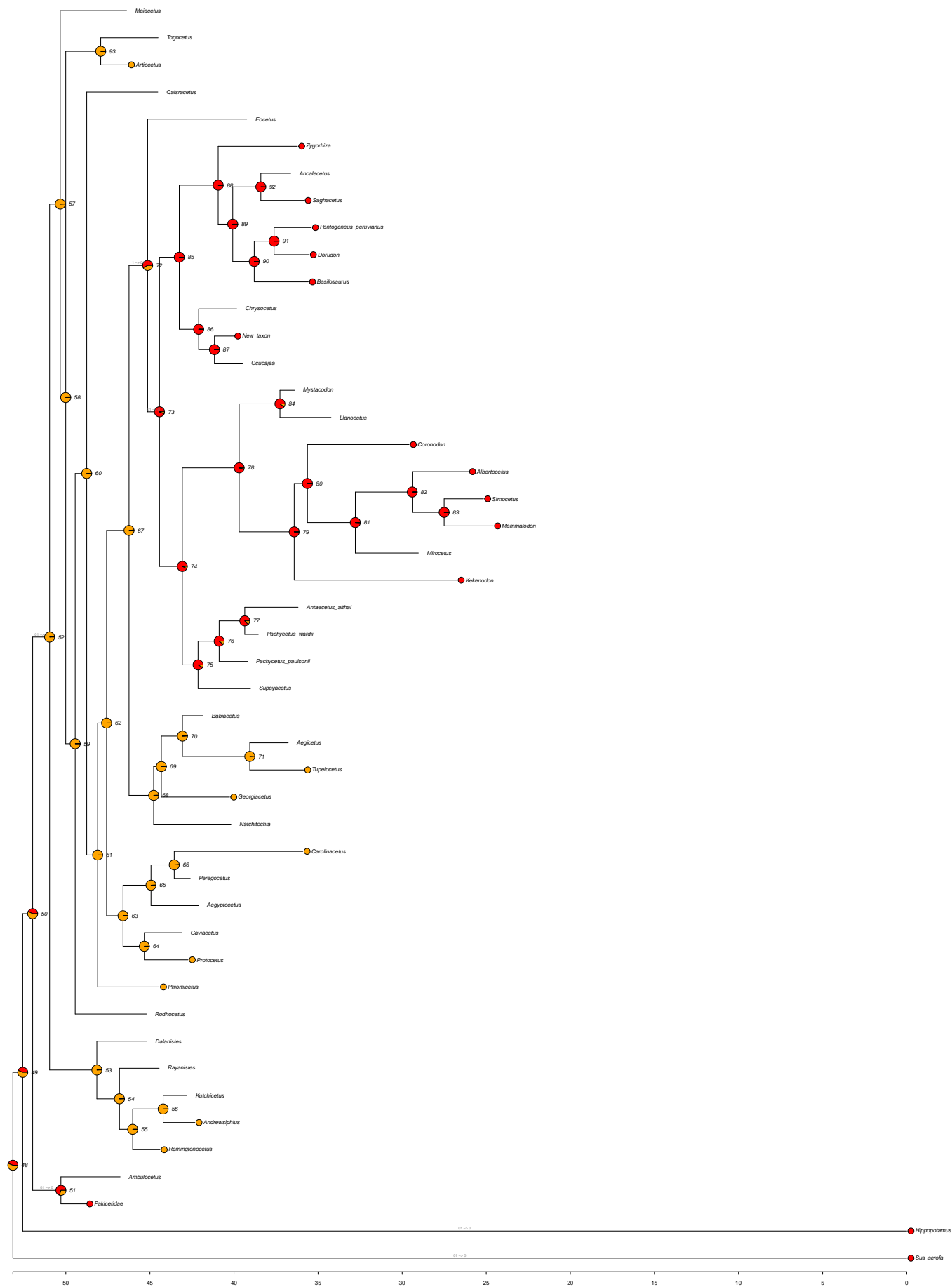

state 0 state 1

Supplement: Supplementary file 6 — Supplementary Data 3 [file 42003_2023_4986_MOESM6_ESM.zip › Supplementary Data 3/Supplementary Data 1_BTD_ASR/trait_0058_tree.plot.pdf]

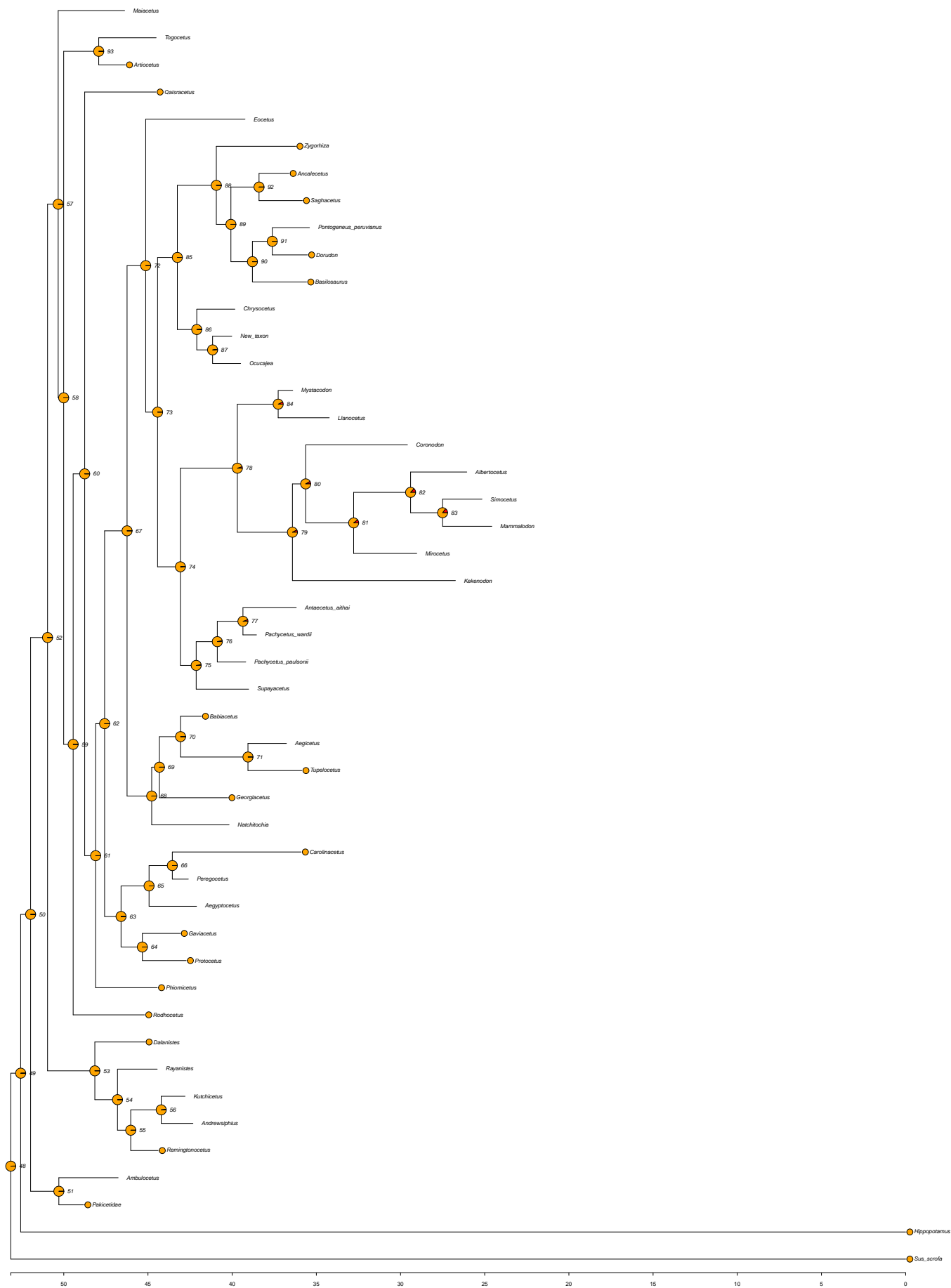

Supplement: Supplementary file 6 — Supplementary Data 3 [file 42003_2023_4986_MOESM6_ESM.zip › Supplementary Data 3/Supplementary Data 1_BTD_ASR/trait_0059_tree.plot.pdf]

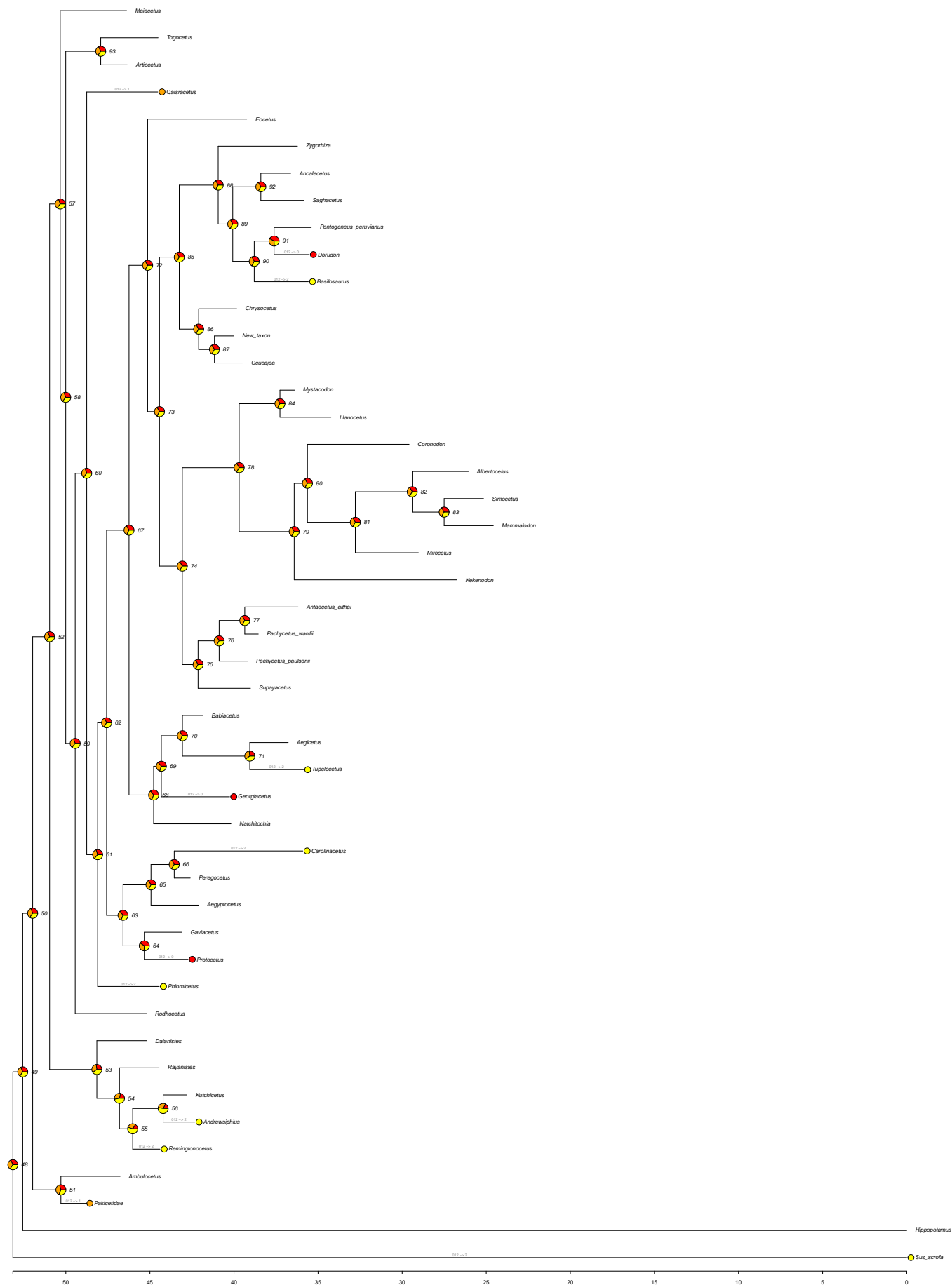

state 0 state 1 state 2

Supplement: Supplementary file 6 — Supplementary Data 3 [file 42003_2023_4986_MOESM6_ESM.zip › Supplementary Data 3/Supplementary Data 1_BTD_ASR/trait_0060_tree.plot.pdf]

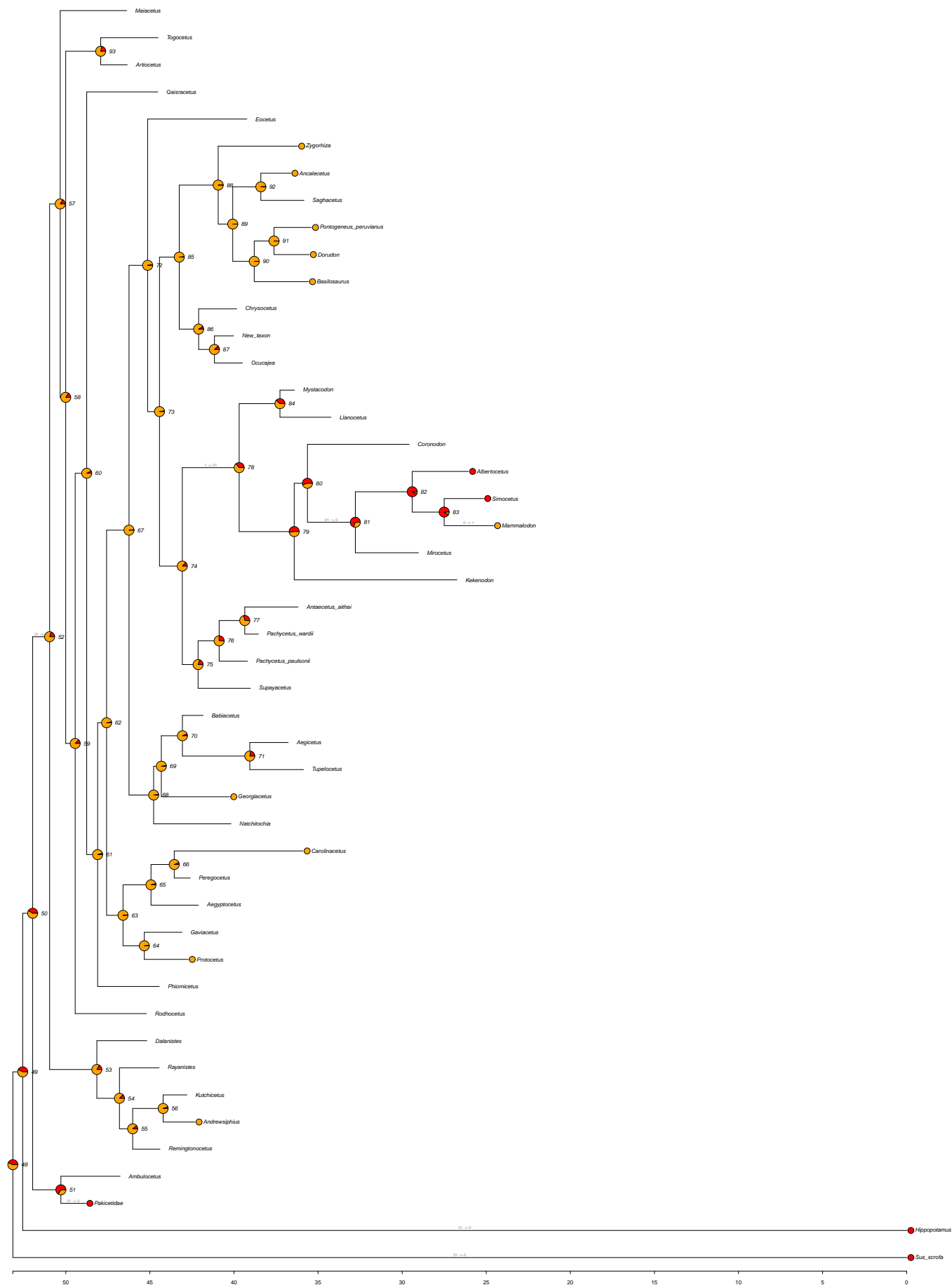

Supplement: Supplementary file 6 — Supplementary Data 3 [file 42003_2023_4986_MOESM6_ESM.zip › Supplementary Data 3/Supplementary Data 1_BTD_ASR/trait_0061_tree.plot.pdf]

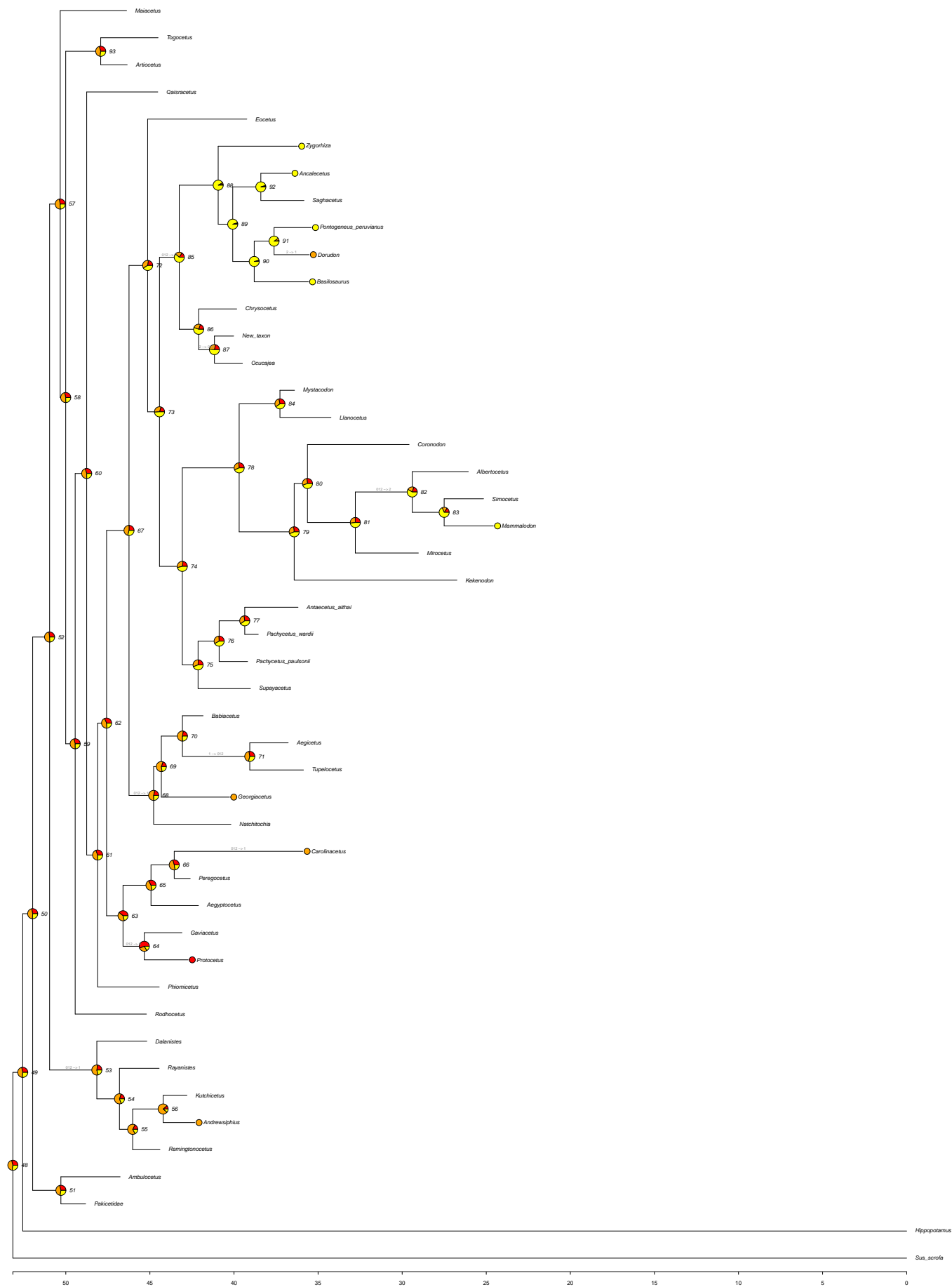

Supplement: Supplementary file 6 — Supplementary Data 3 [file 42003_2023_4986_MOESM6_ESM.zip › Supplementary Data 3/Supplementary Data 1_BTD_ASR/trait_0062_tree.plot.pdf]

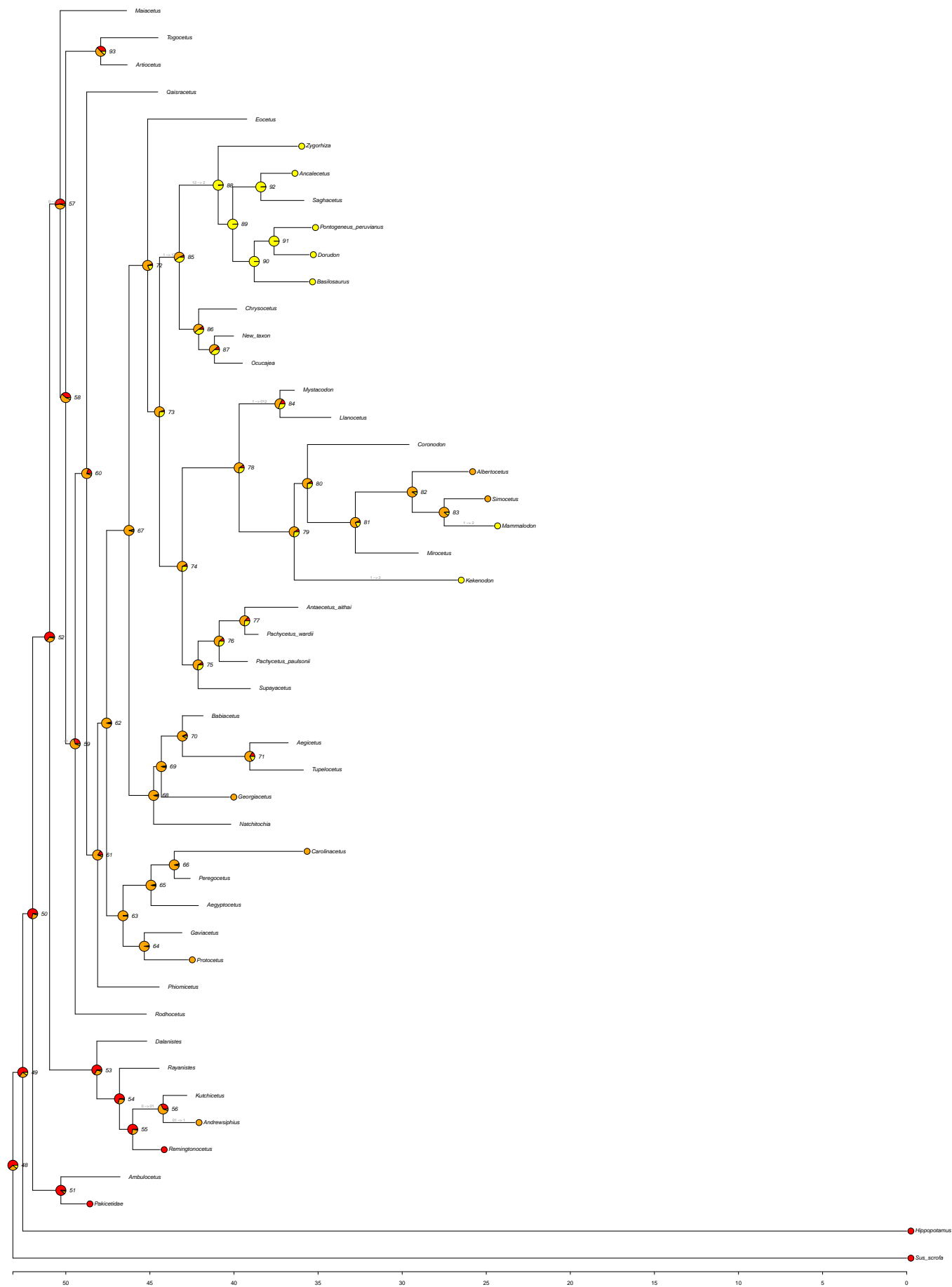

Supplement: Supplementary file 6 — Supplementary Data 3 [file 42003_2023_4986_MOESM6_ESM.zip › Supplementary Data 3/Supplementary Data 1_BTD_ASR/trait_0063_tree.plot.pdf]

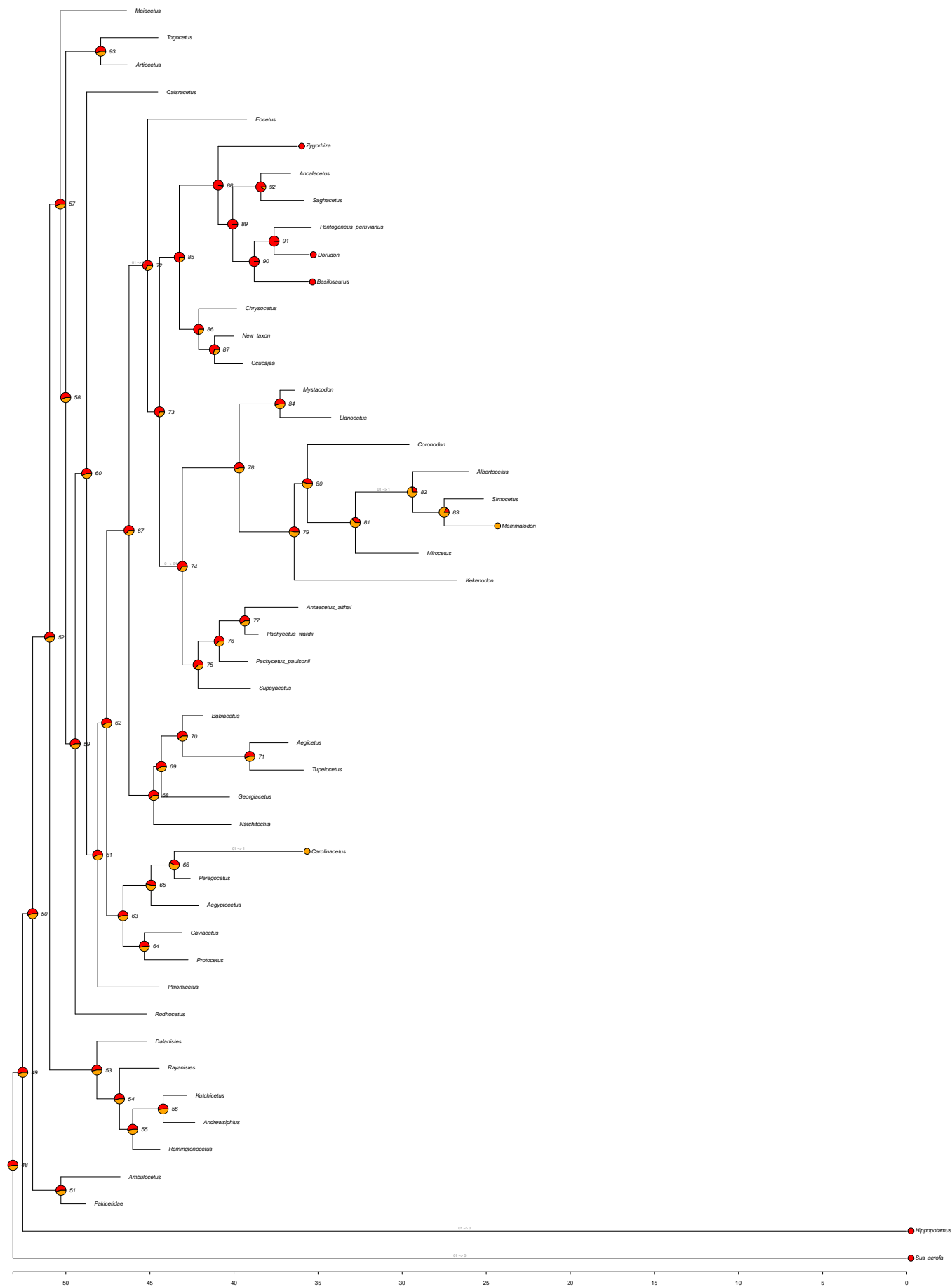

Supplement: Supplementary file 6 — Supplementary Data 3 [file 42003_2023_4986_MOESM6_ESM.zip › Supplementary Data 3/Supplementary Data 1_BTD_ASR/trait_0064_tree.plot.pdf]

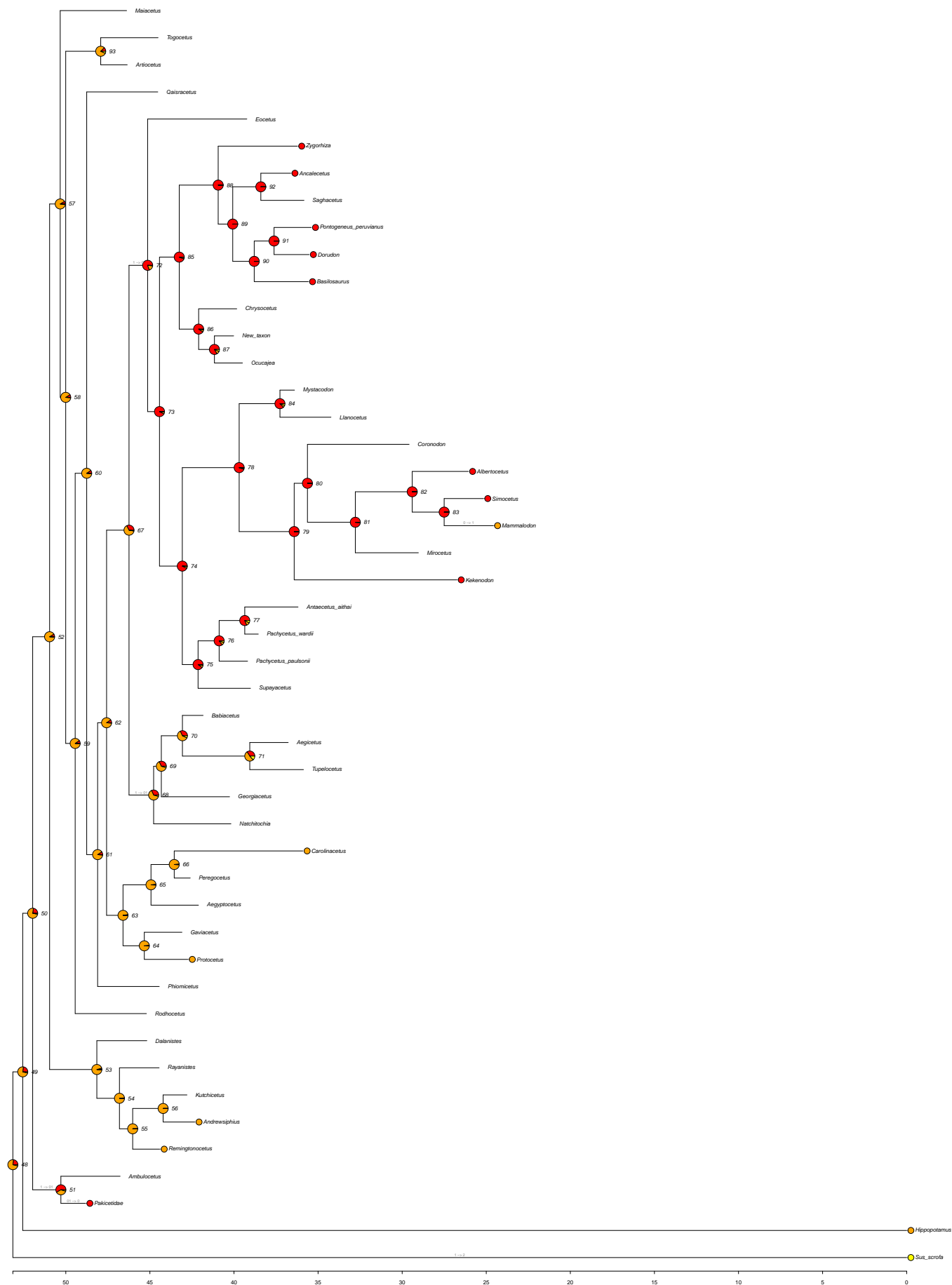

Supplement: Supplementary file 6 — Supplementary Data 3 [file 42003_2023_4986_MOESM6_ESM.zip › Supplementary Data 3/Supplementary Data 1_BTD_ASR/trait_0065_tree.plot.pdf]

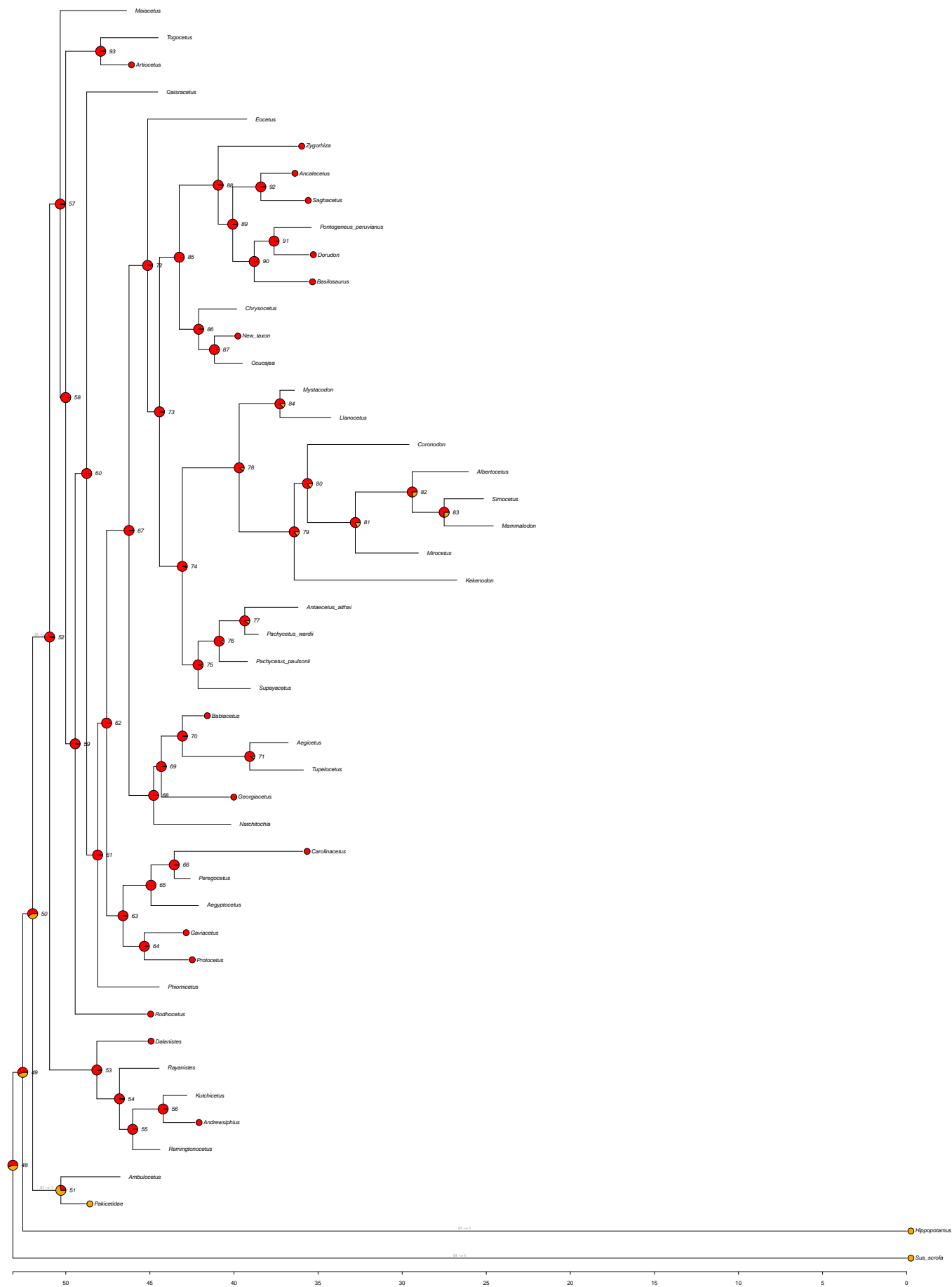

Supplement: Supplementary file 6 — Supplementary Data 3 [file 42003_2023_4986_MOESM6_ESM.zip › Supplementary Data 3/Supplementary Data 1_BTD_ASR/trait_0066_tree.plot.pdf]

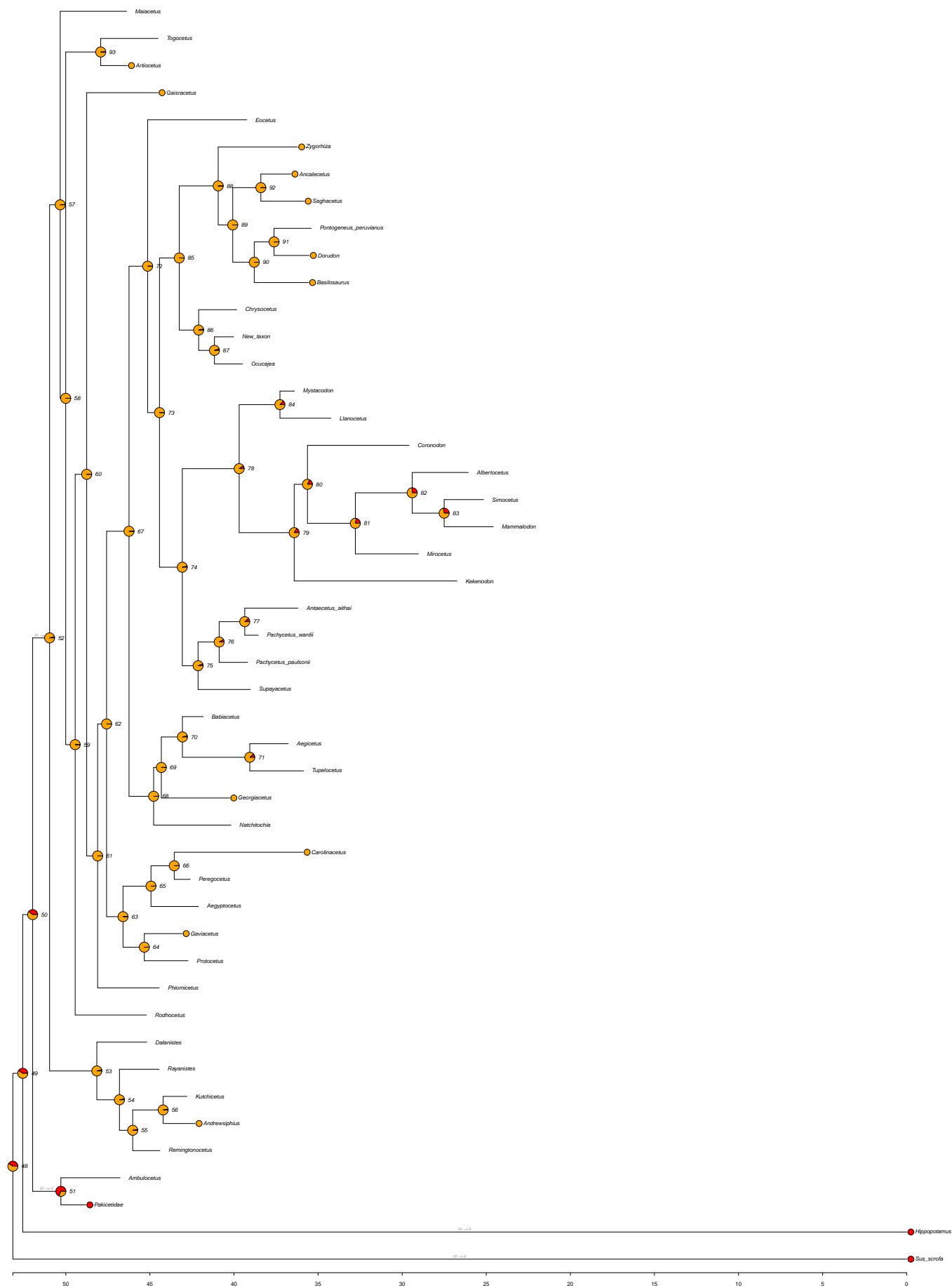

Supplement: Supplementary file 6 — Supplementary Data 3 [file 42003_2023_4986_MOESM6_ESM.zip › Supplementary Data 3/Supplementary Data 1_BTD_ASR/trait_0067_tree.plot.pdf]

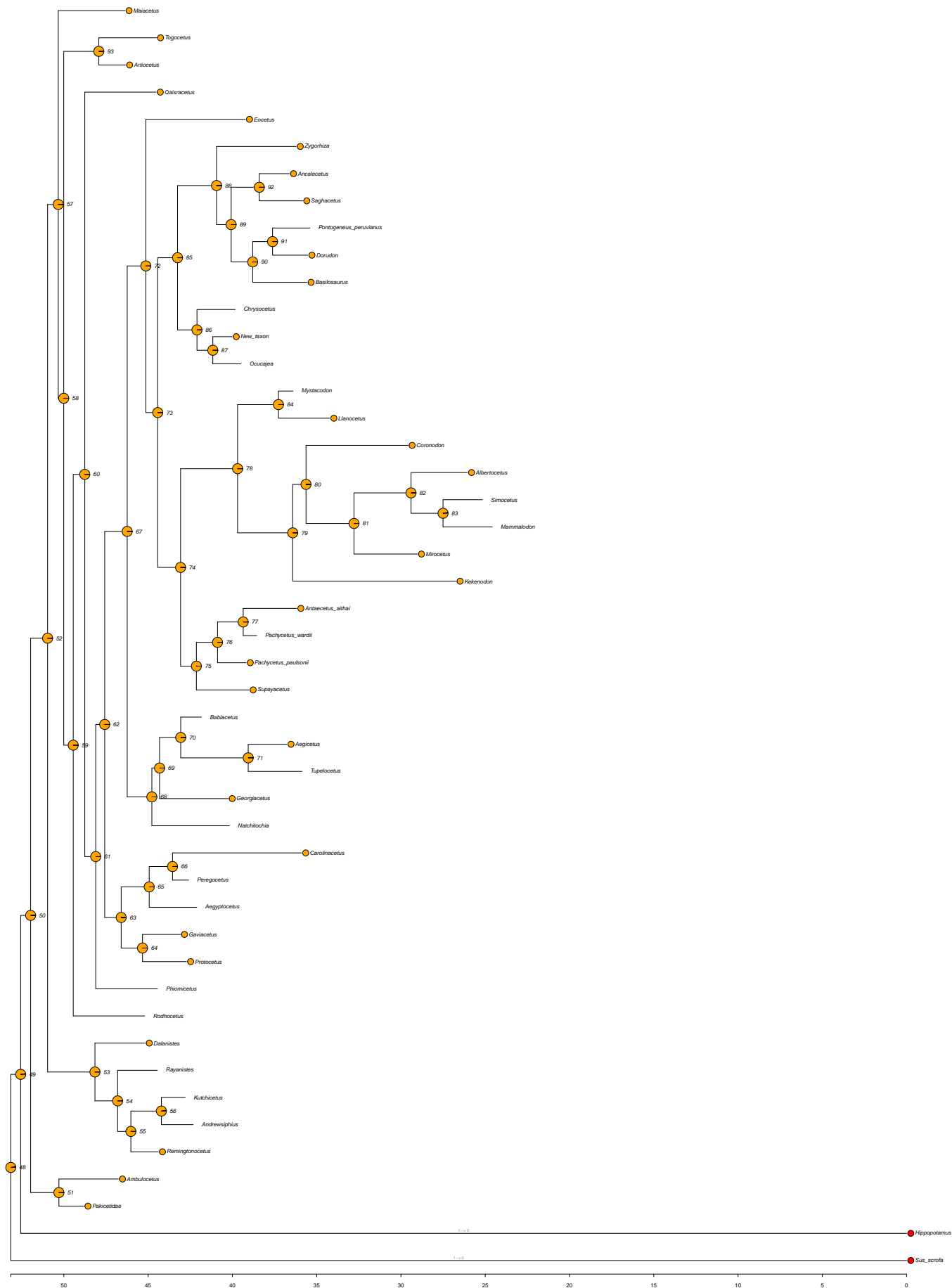

Supplement: Supplementary file 6 — Supplementary Data 3 [file 42003_2023_4986_MOESM6_ESM.zip › Supplementary Data 3/Supplementary Data 1_BTD_ASR/trait_0068_tree.plot.pdf]

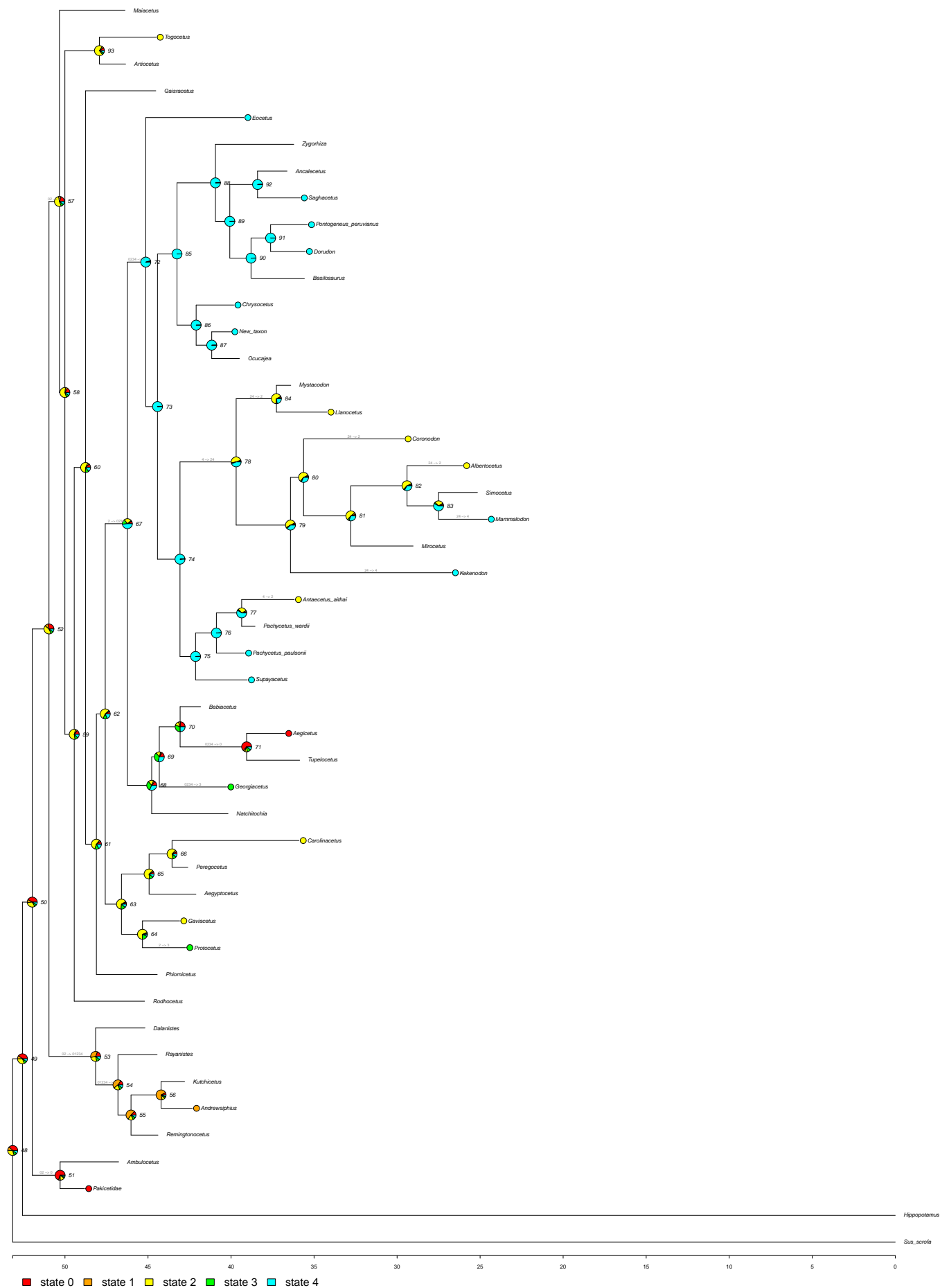

Supplement: Supplementary file 6 — Supplementary Data 3 [file 42003_2023_4986_MOESM6_ESM.zip › Supplementary Data 3/Supplementary Data 1_BTD_ASR/trait_0069_tree.plot.pdf]

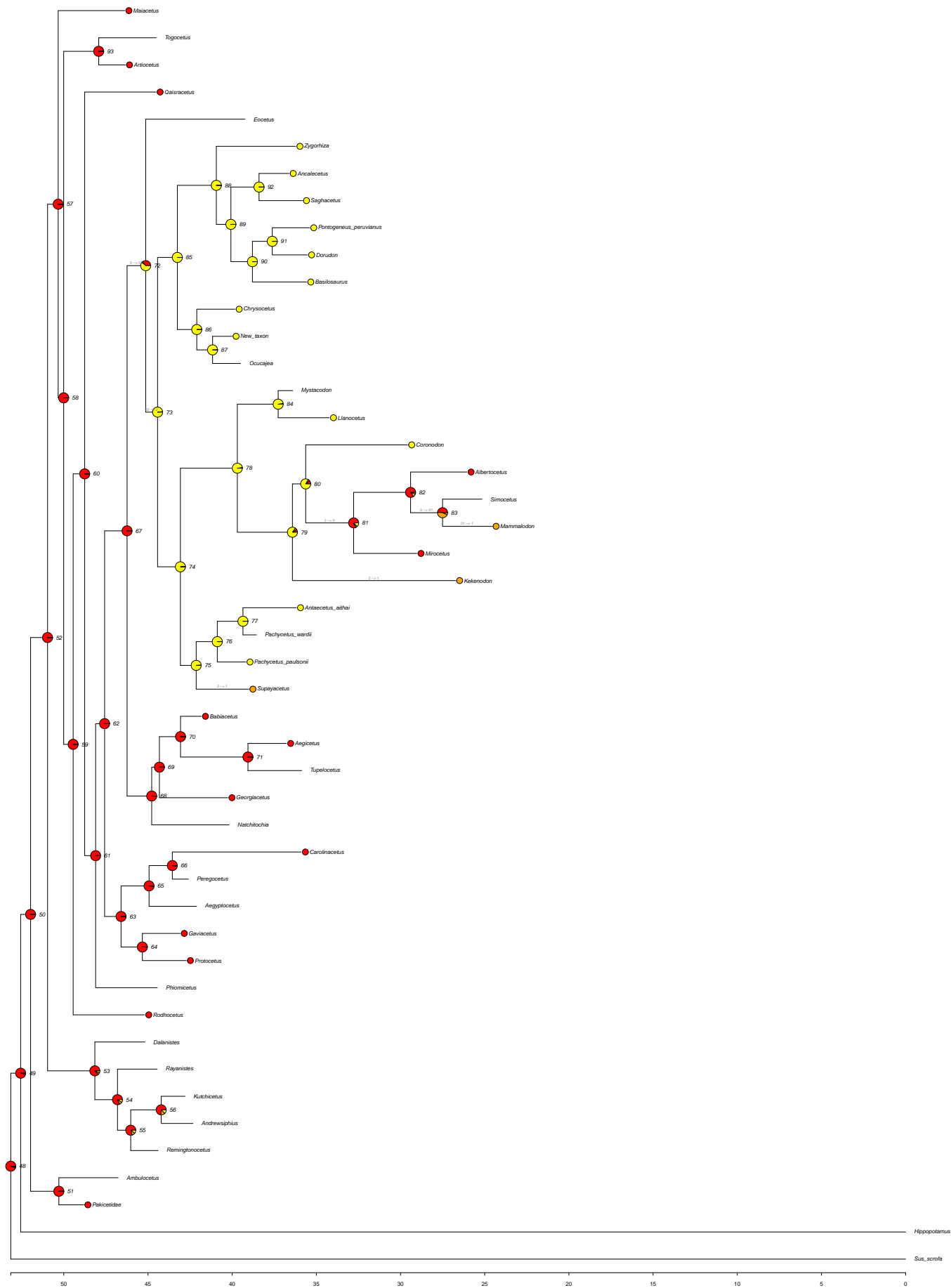

Supplement: Supplementary file 6 — Supplementary Data 3 [file 42003_2023_4986_MOESM6_ESM.zip › Supplementary Data 3/Supplementary Data 1_BTD_ASR/trait_0070_tree.plot.pdf]

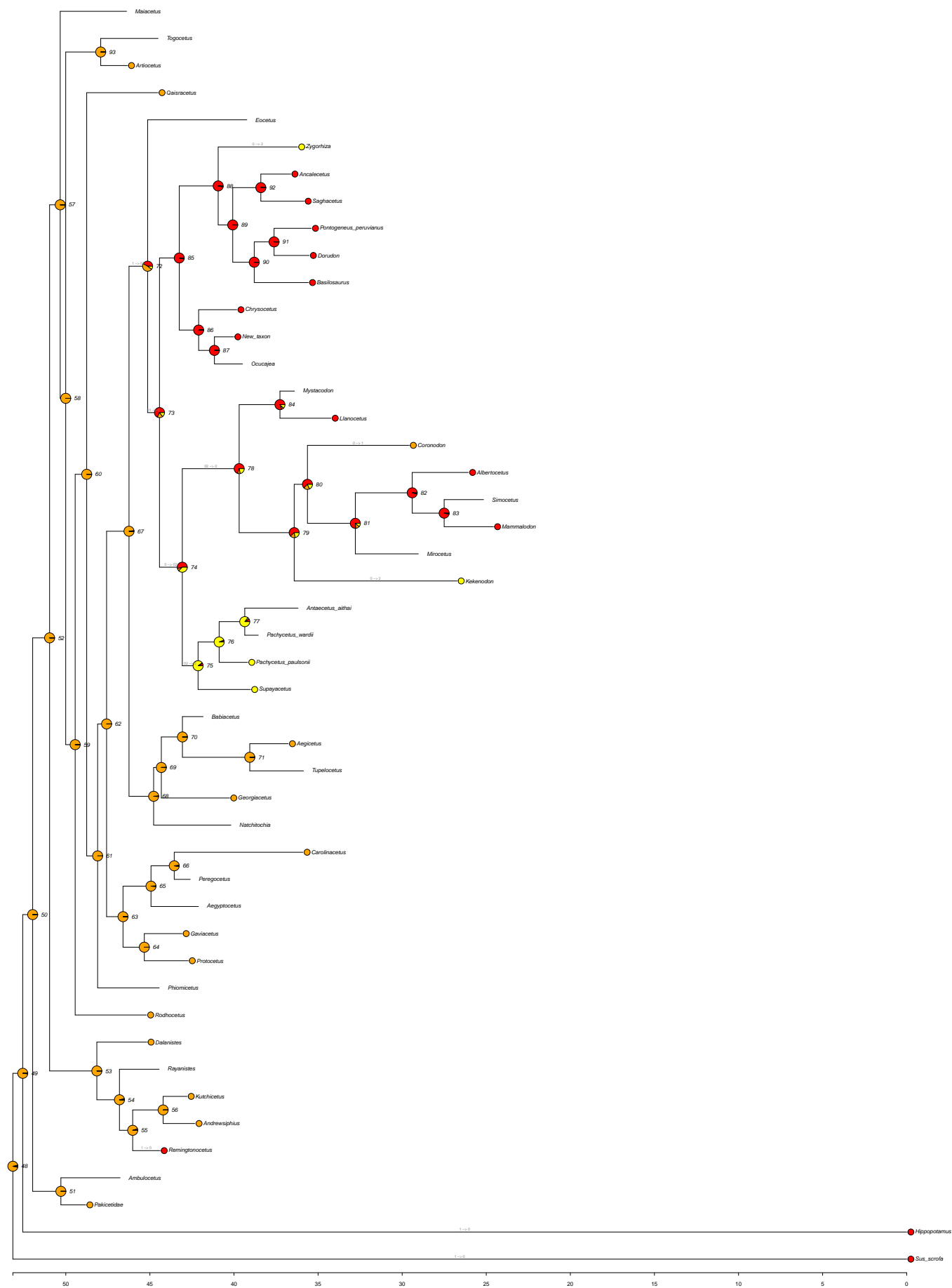

Supplement: Supplementary file 6 — Supplementary Data 3 [file 42003_2023_4986_MOESM6_ESM.zip › Supplementary Data 3/Supplementary Data 1_BTD_ASR/trait_0071_tree.plot.pdf]

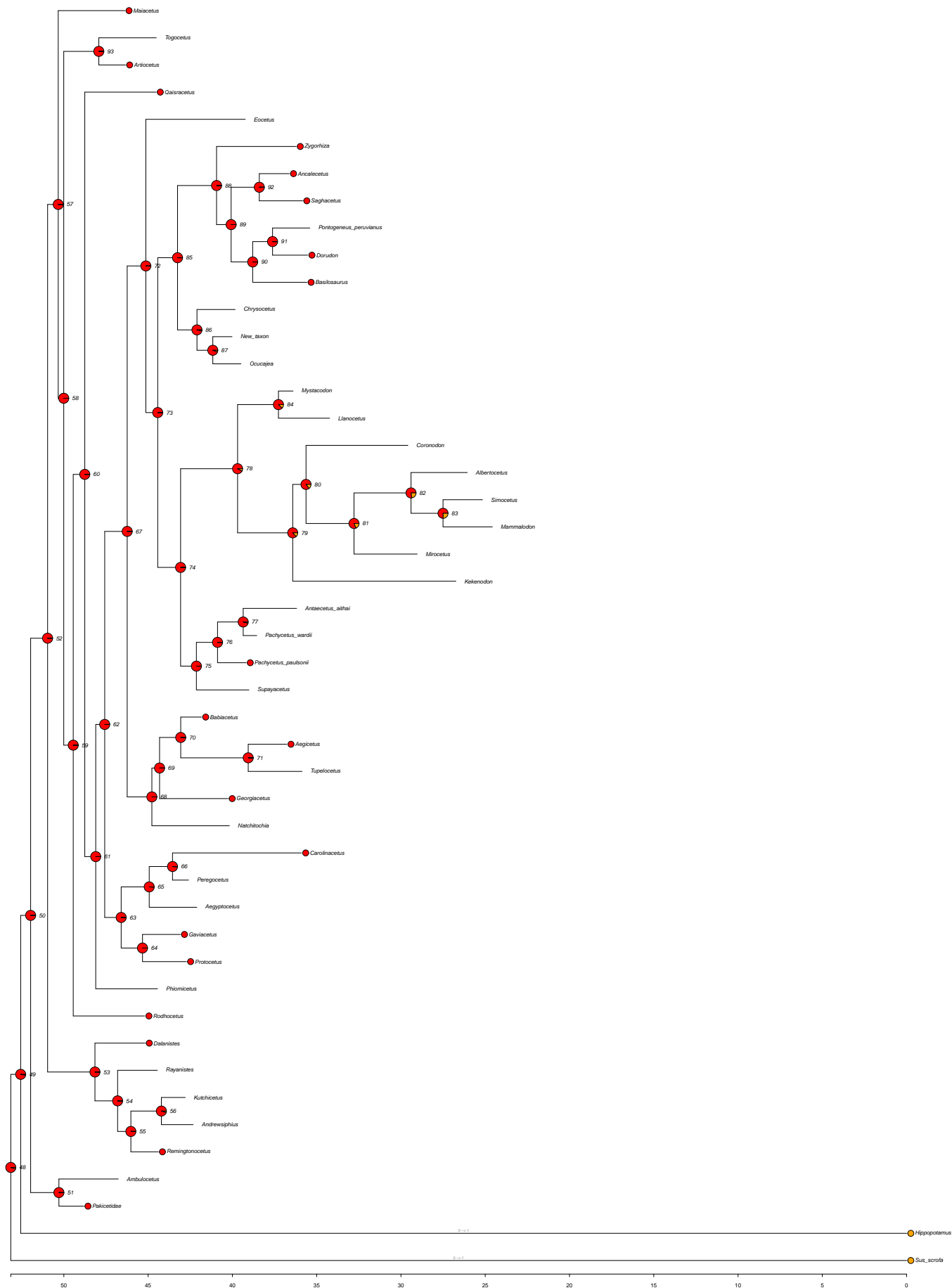

Supplement: Supplementary file 6 — Supplementary Data 3 [file 42003_2023_4986_MOESM6_ESM.zip › Supplementary Data 3/Supplementary Data 1_BTD_ASR/trait_0072_tree.plot.pdf]

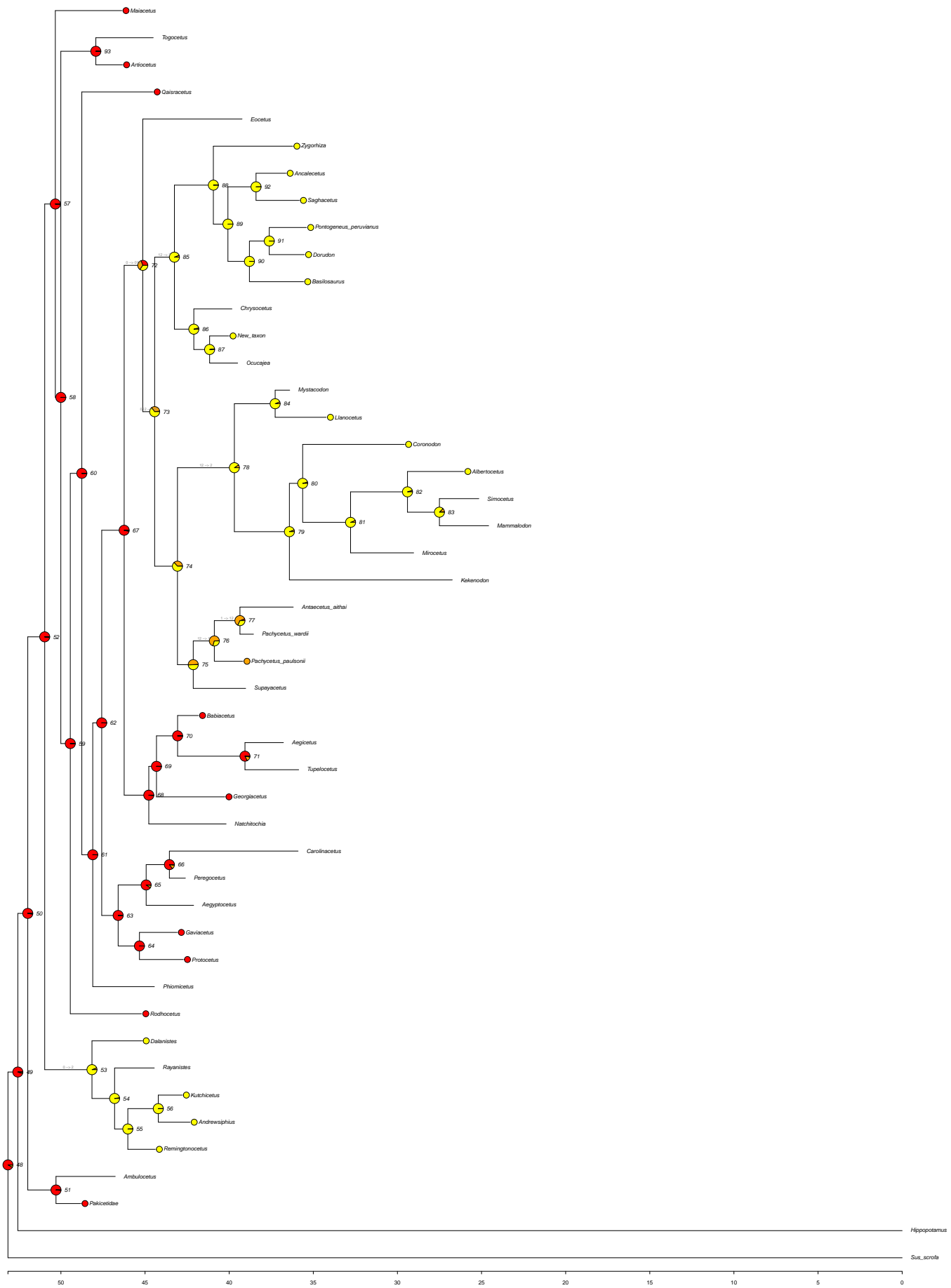

Supplement: Supplementary file 6 — Supplementary Data 3 [file 42003_2023_4986_MOESM6_ESM.zip › Supplementary Data 3/Supplementary Data 1_BTD_ASR/trait_0073_tree.plot.pdf]

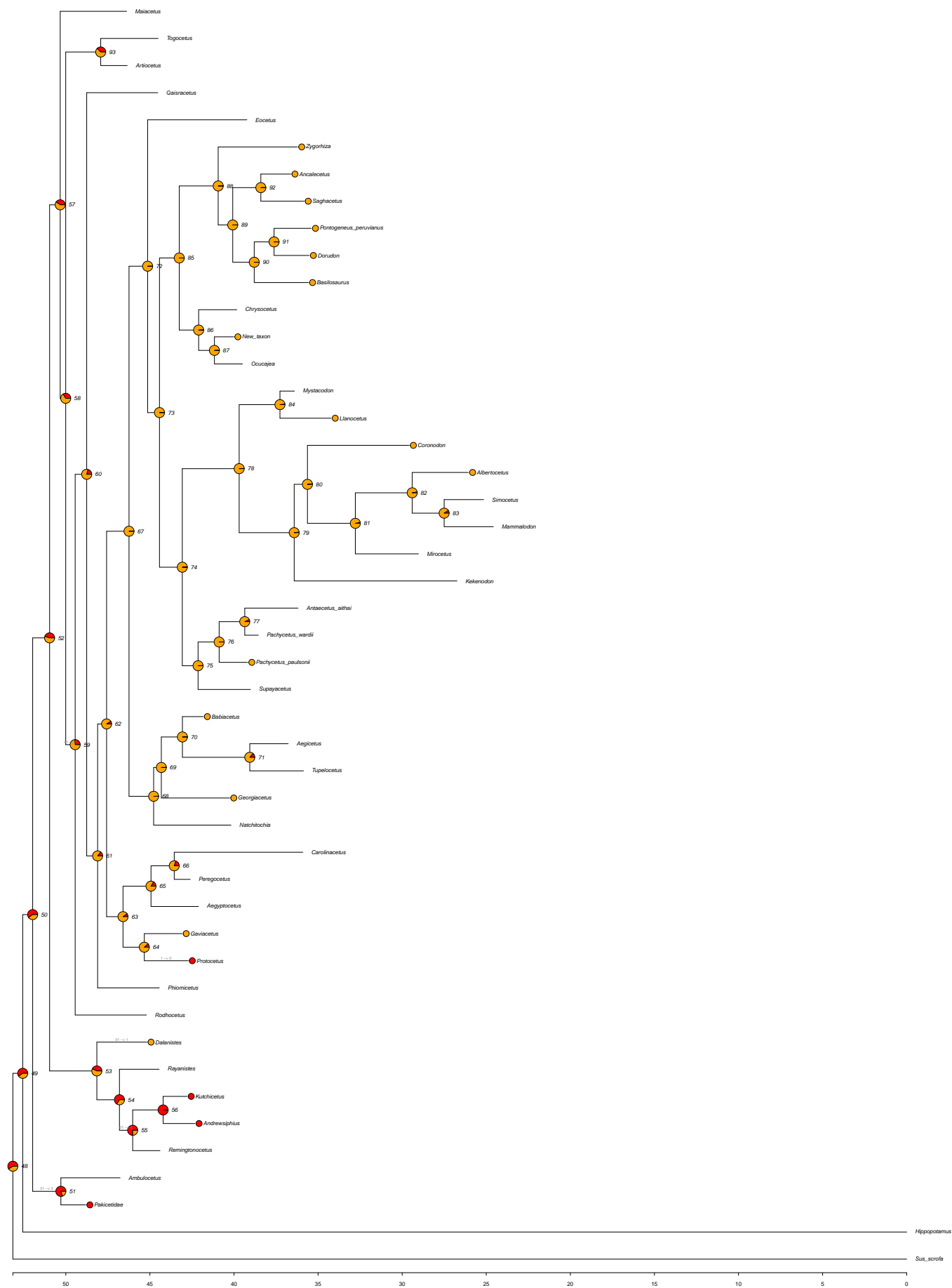

state 0 state 1

Supplement: Supplementary file 6 — Supplementary Data 3 [file 42003_2023_4986_MOESM6_ESM.zip › Supplementary Data 3/Supplementary Data 1_BTD_ASR/trait_0074_tree.plot.pdf]

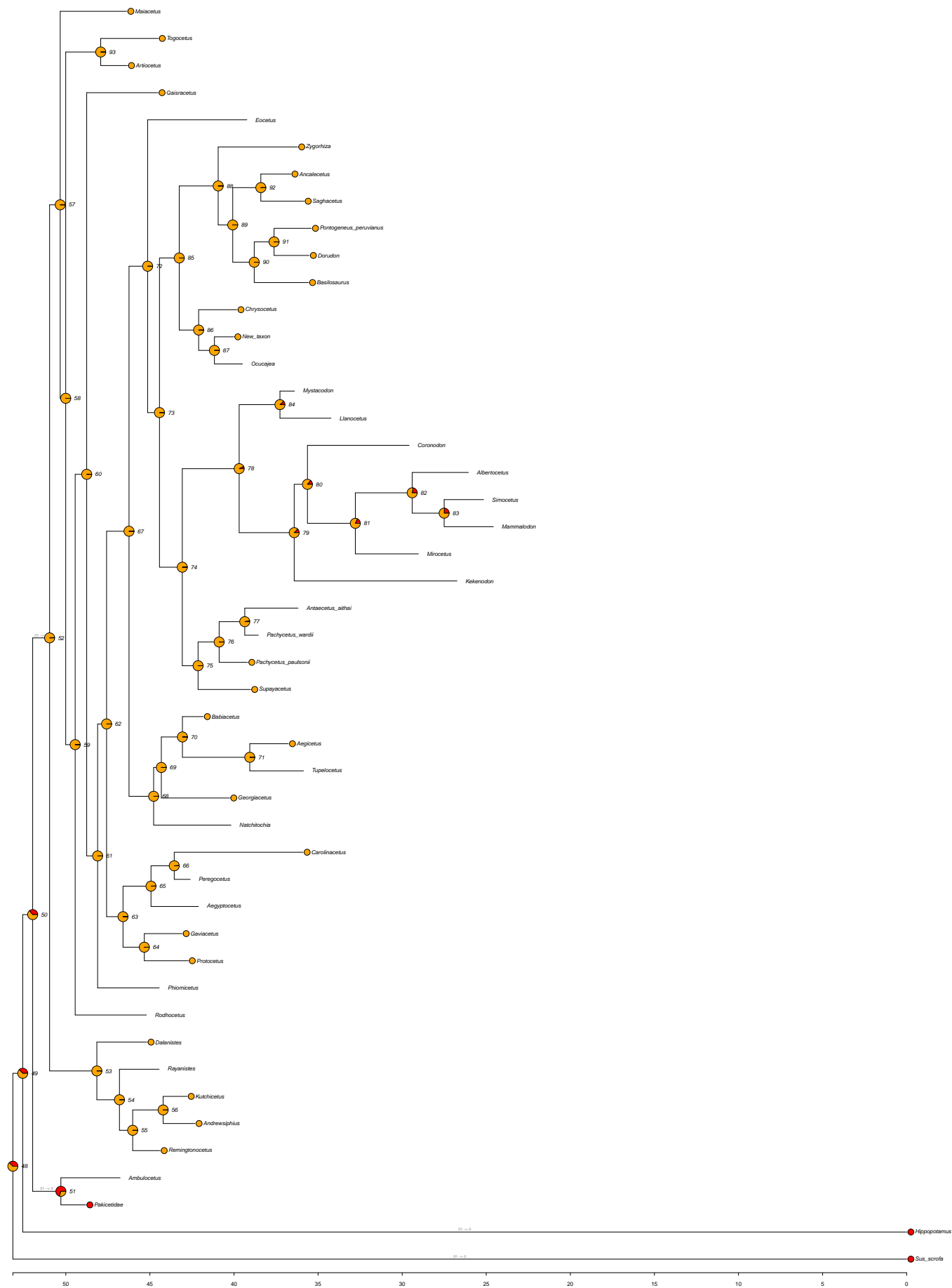

Supplement: Supplementary file 6 — Supplementary Data 3 [file 42003_2023_4986_MOESM6_ESM.zip › Supplementary Data 3/Supplementary Data 1_BTD_ASR/trait_0075_tree.plot.pdf]

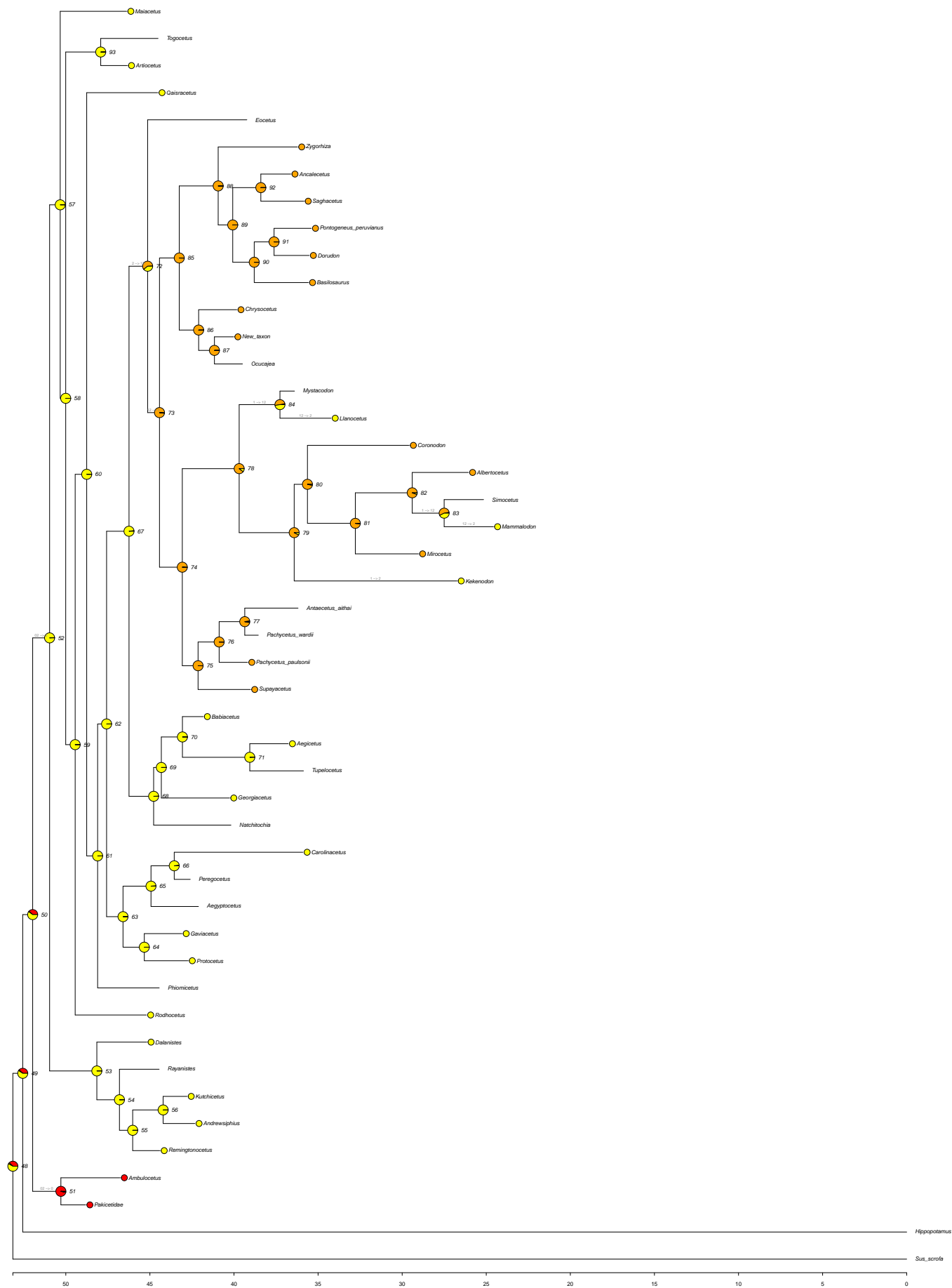

Supplement: Supplementary file 6 — Supplementary Data 3 [file 42003_2023_4986_MOESM6_ESM.zip › Supplementary Data 3/Supplementary Data 1_BTD_ASR/trait_0076_tree.plot.pdf]

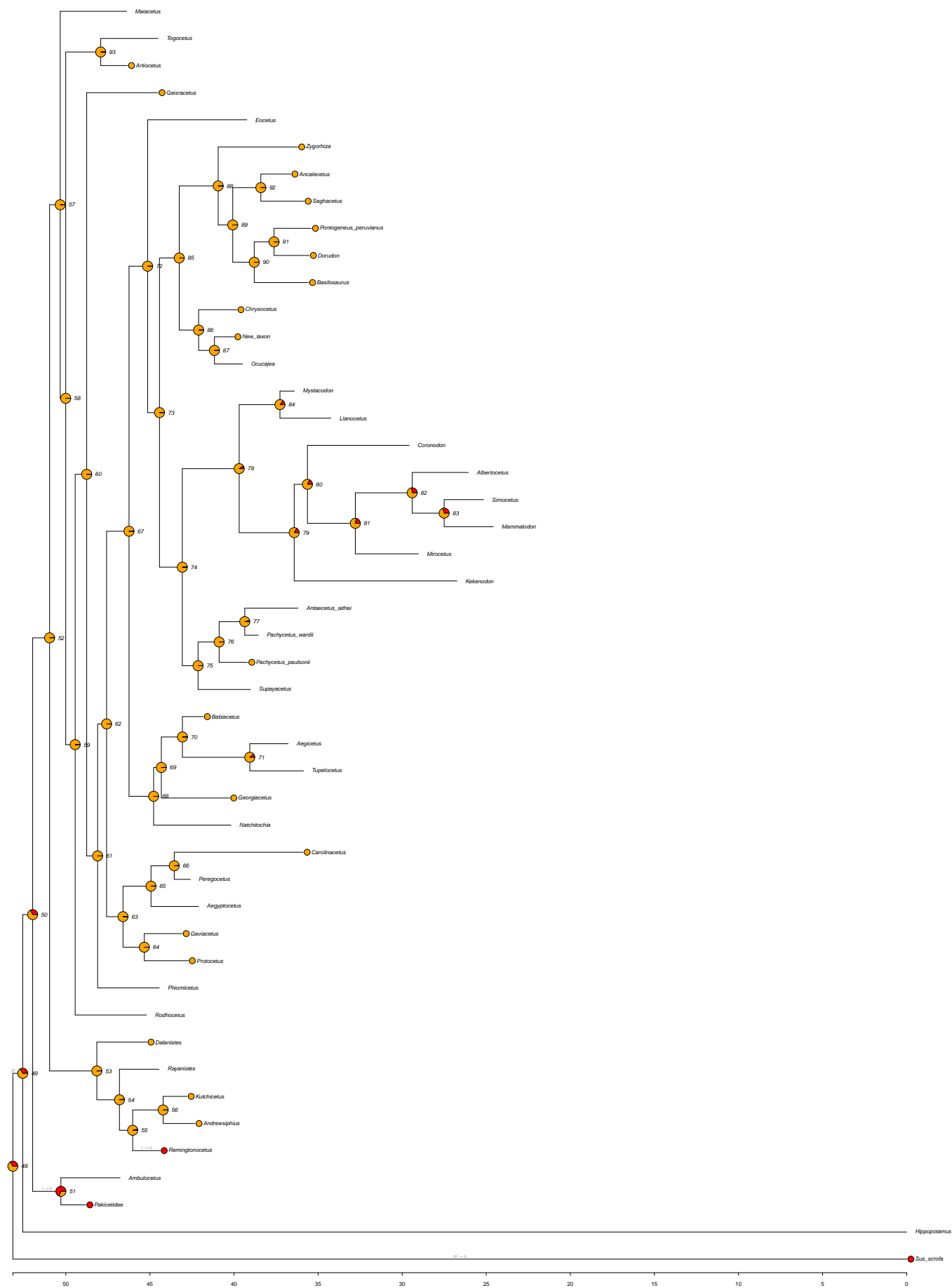

Supplement: Supplementary file 6 — Supplementary Data 3 [file 42003_2023_4986_MOESM6_ESM.zip › Supplementary Data 3/Supplementary Data 1_BTD_ASR/trait_0077_tree.plot.pdf]

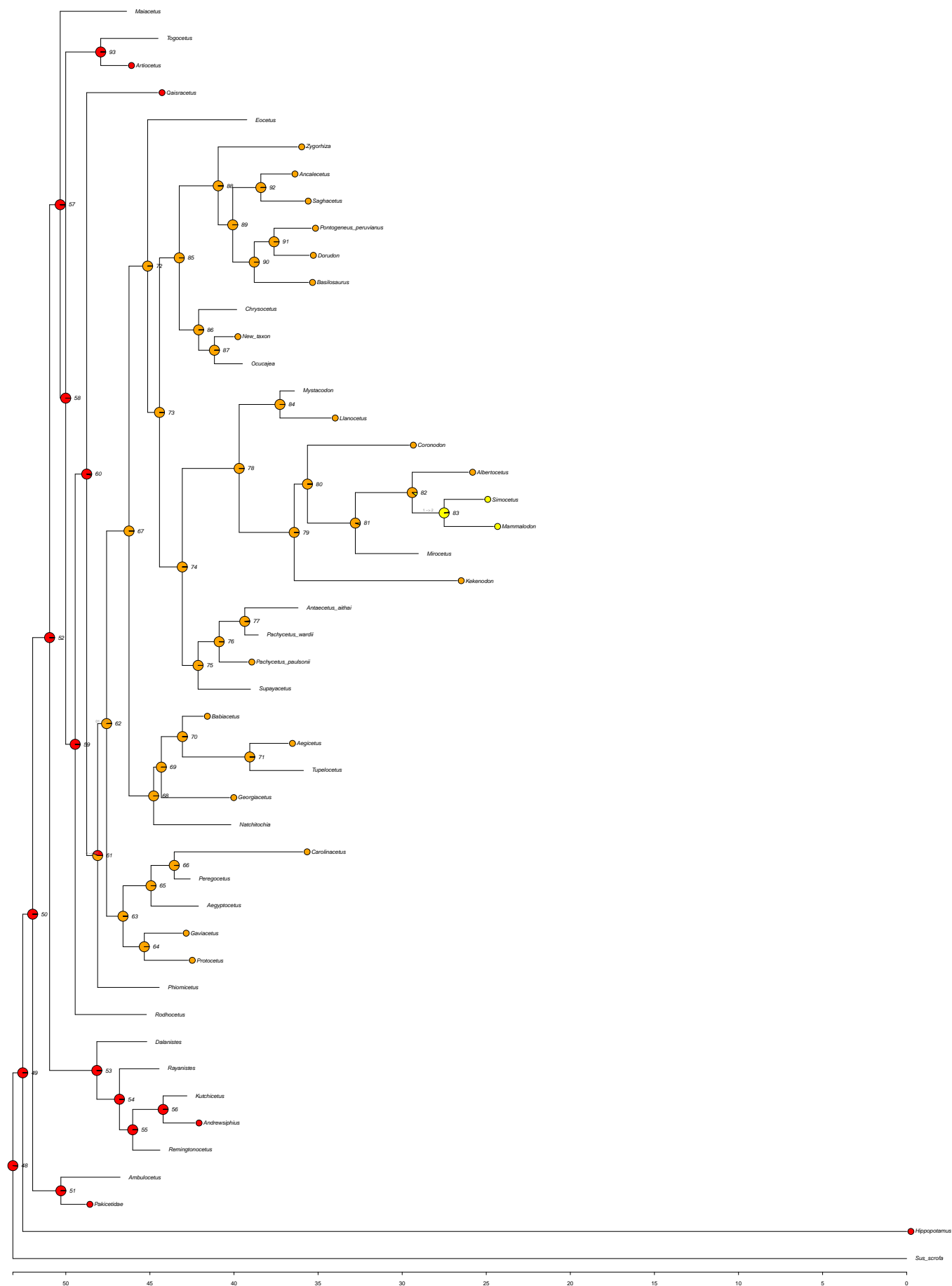

Supplement: Supplementary file 6 — Supplementary Data 3 [file 42003_2023_4986_MOESM6_ESM.zip › Supplementary Data 3/Supplementary Data 1_BTD_ASR/trait_0078_tree.plot.pdf]

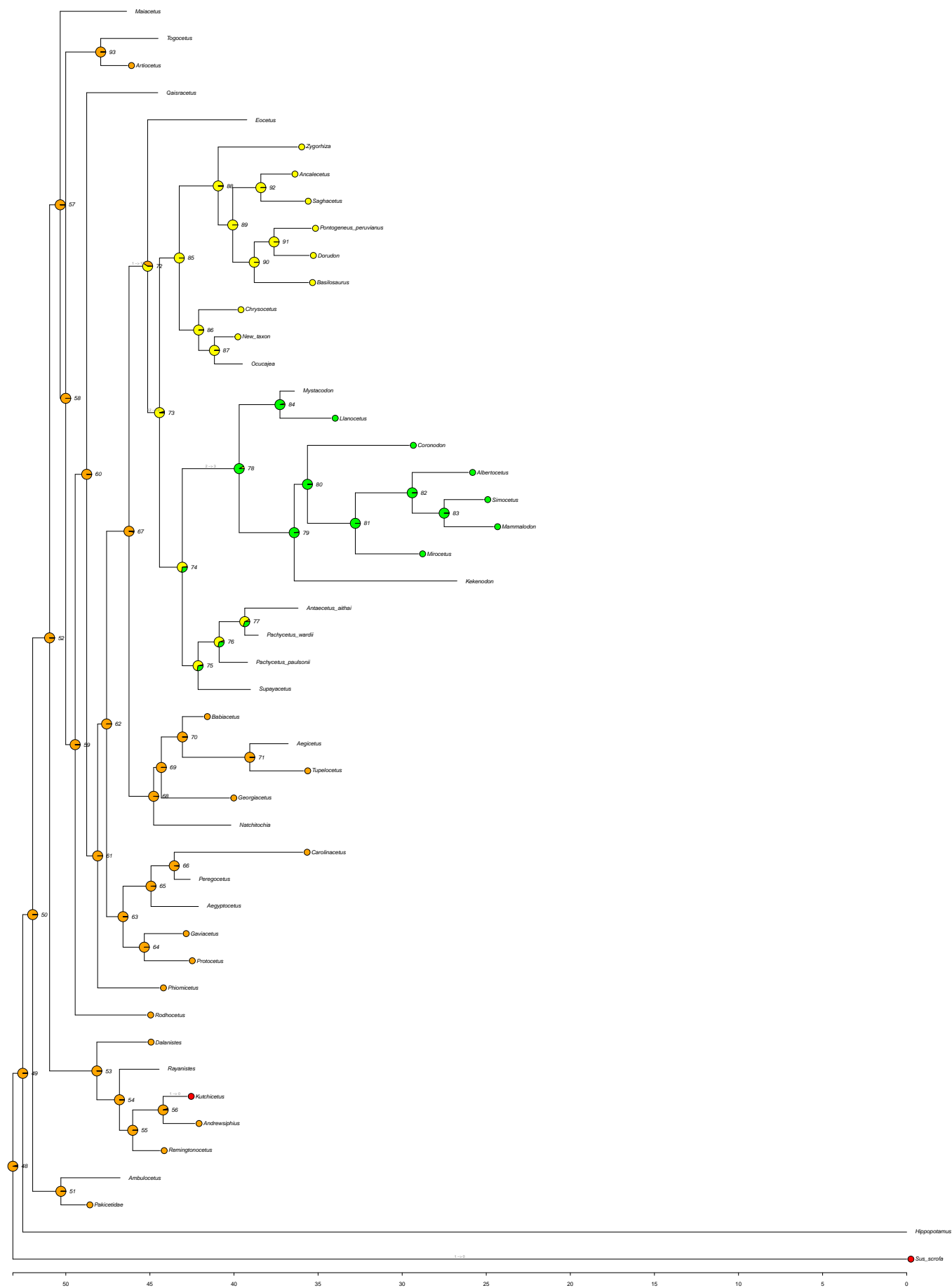

Supplement: Supplementary file 6 — Supplementary Data 3 [file 42003_2023_4986_MOESM6_ESM.zip › Supplementary Data 3/Supplementary Data 1_BTD_ASR/trait_0079_tree.plot.pdf]

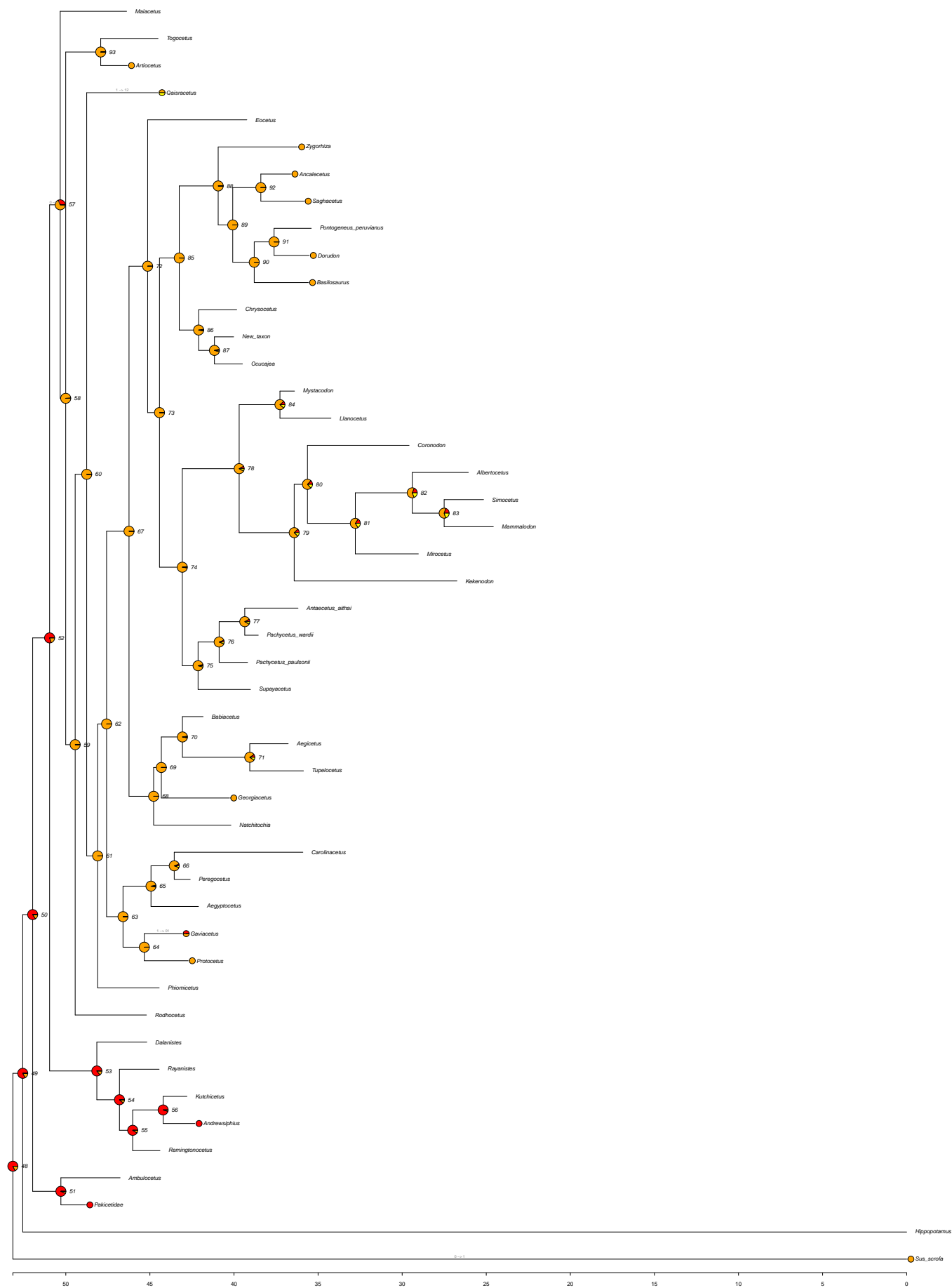

state 0 state 1 state 2

Supplement: Supplementary file 6 — Supplementary Data 3 [file 42003_2023_4986_MOESM6_ESM.zip › Supplementary Data 3/Supplementary Data 1_BTD_ASR/trait_0080_tree.plot.pdf]

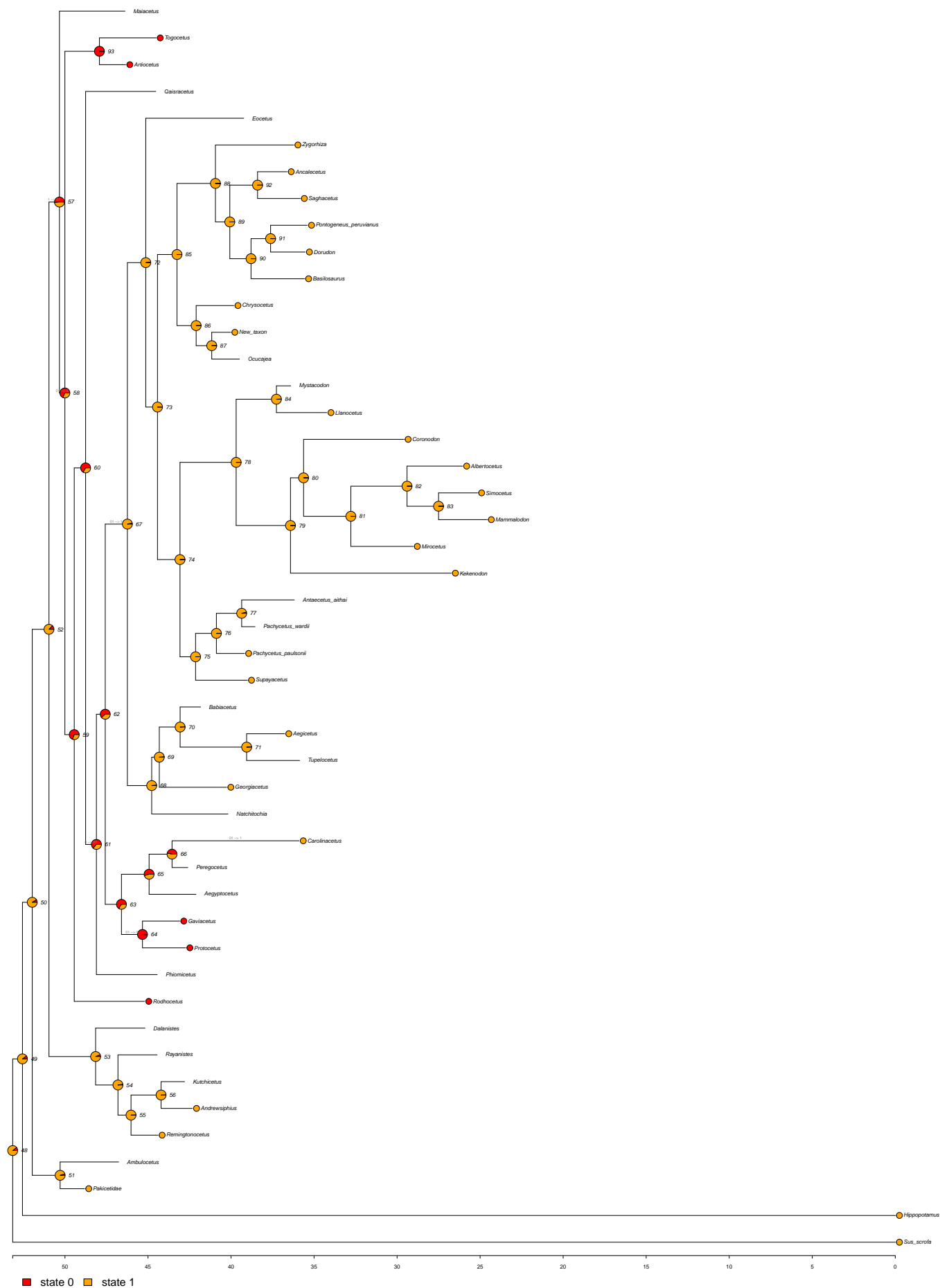

Supplement: Supplementary file 6 — Supplementary Data 3 [file 42003_2023_4986_MOESM6_ESM.zip › Supplementary Data 3/Supplementary Data 1_BTD_ASR/trait_0081_tree.plot.pdf]

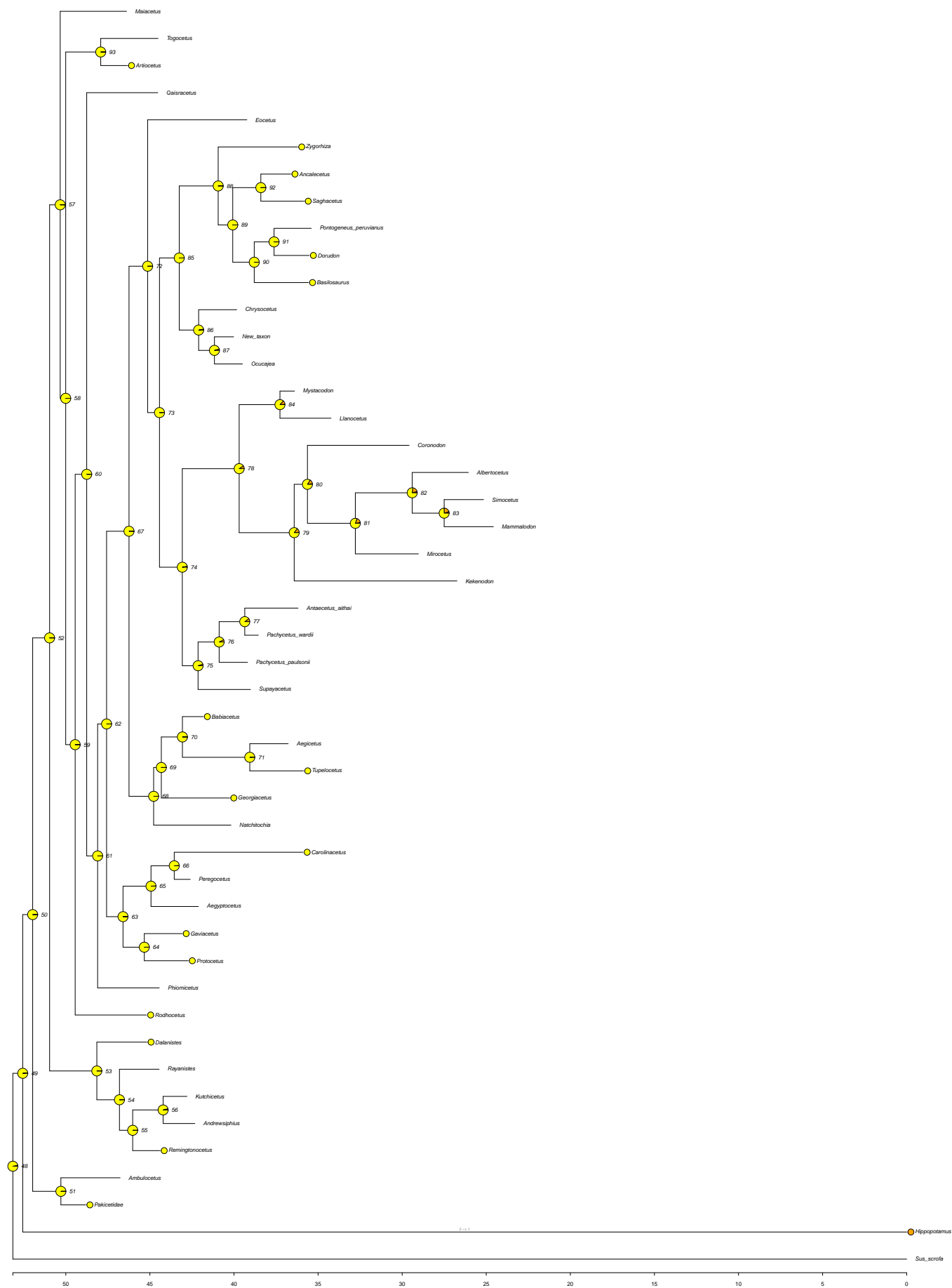

Supplement: Supplementary file 6 — Supplementary Data 3 [file 42003_2023_4986_MOESM6_ESM.zip › Supplementary Data 3/Supplementary Data 1_BTD_ASR/trait_0082_tree.plot.pdf]

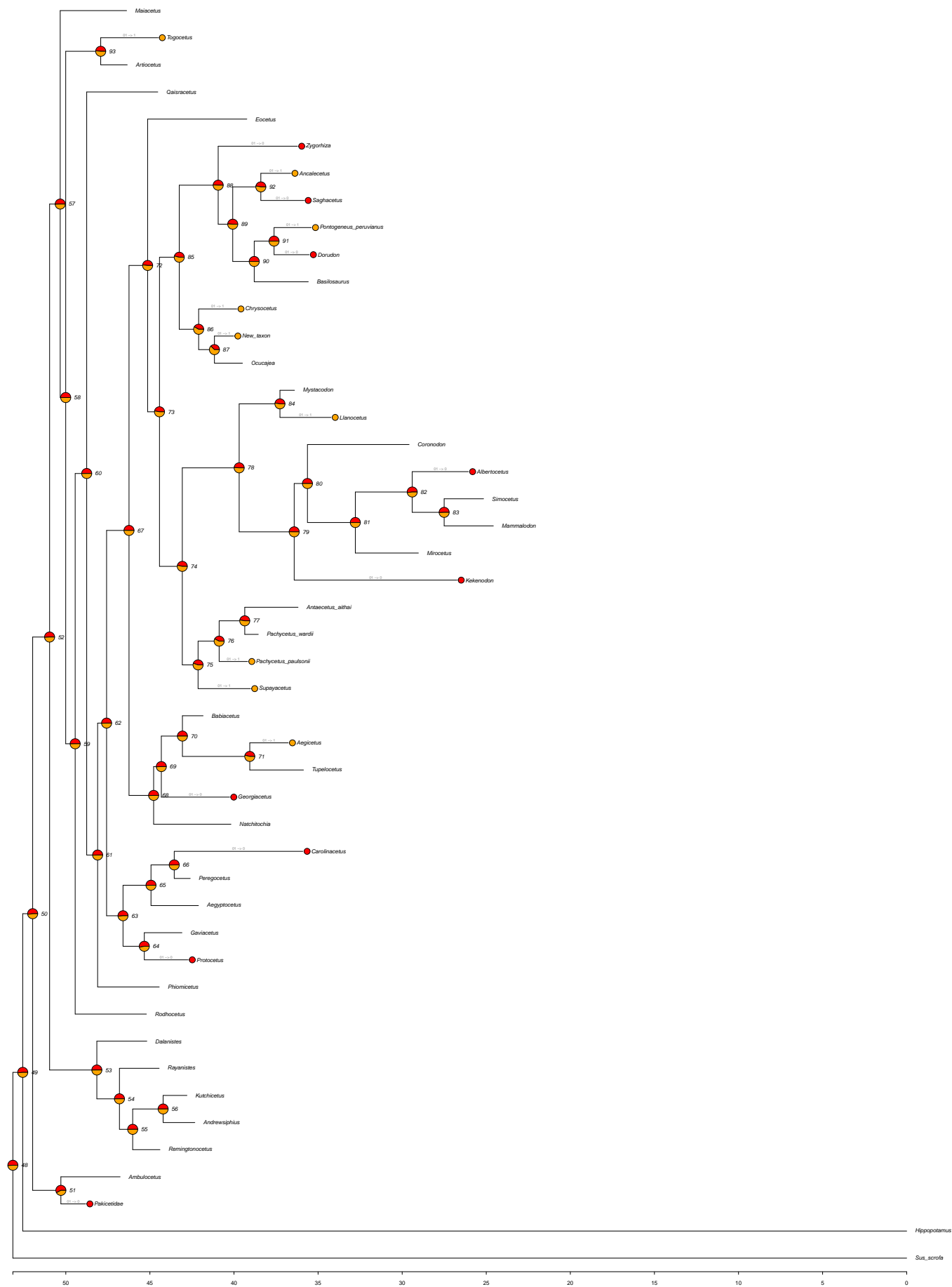

Supplement: Supplementary file 6 — Supplementary Data 3 [file 42003_2023_4986_MOESM6_ESM.zip › Supplementary Data 3/Supplementary Data 1_BTD_ASR/trait_0083_tree.plot.pdf]

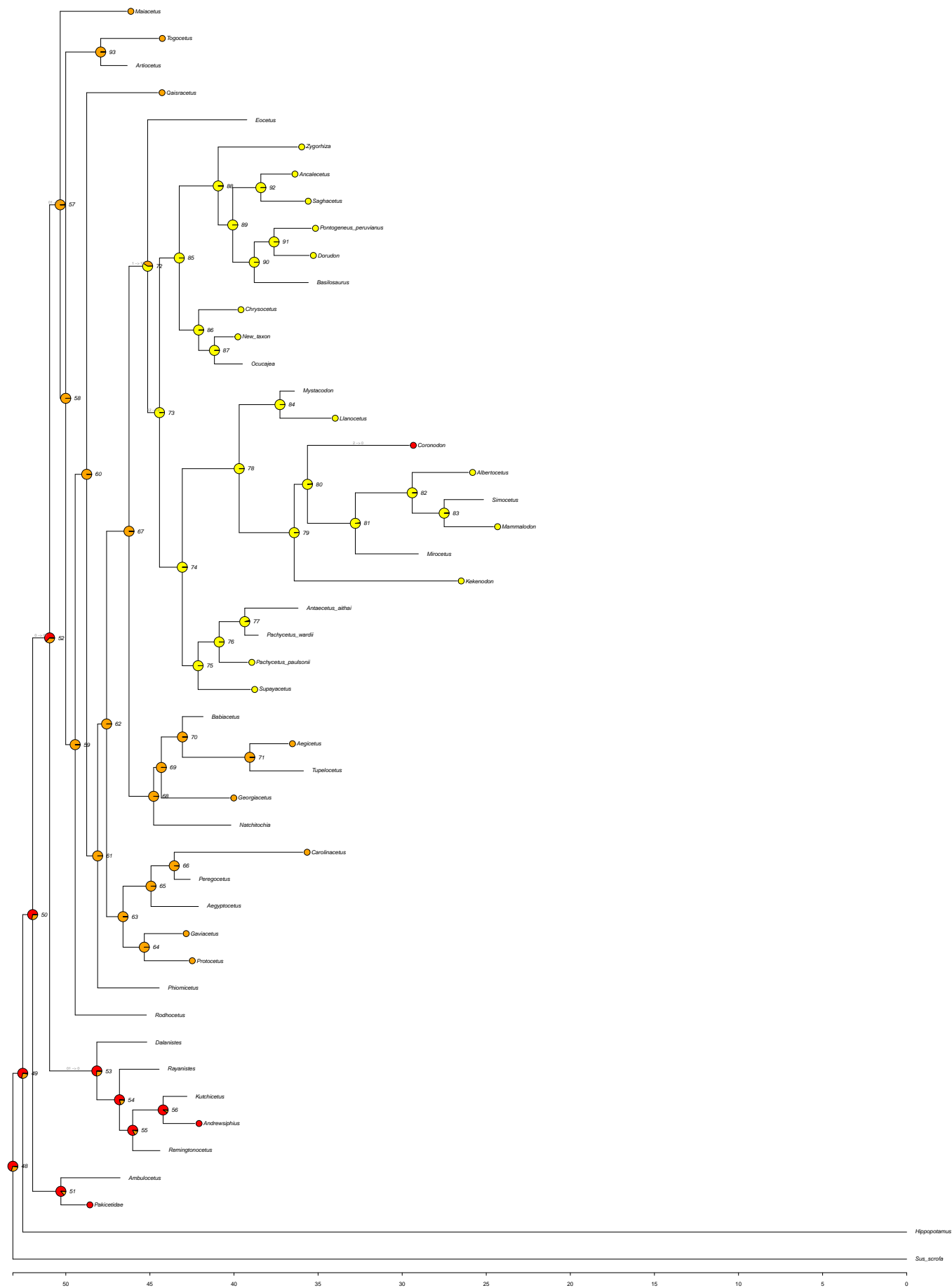

Supplement: Supplementary file 6 — Supplementary Data 3 [file 42003_2023_4986_MOESM6_ESM.zip › Supplementary Data 3/Supplementary Data 1_BTD_ASR/trait_0084_tree.plot.pdf]

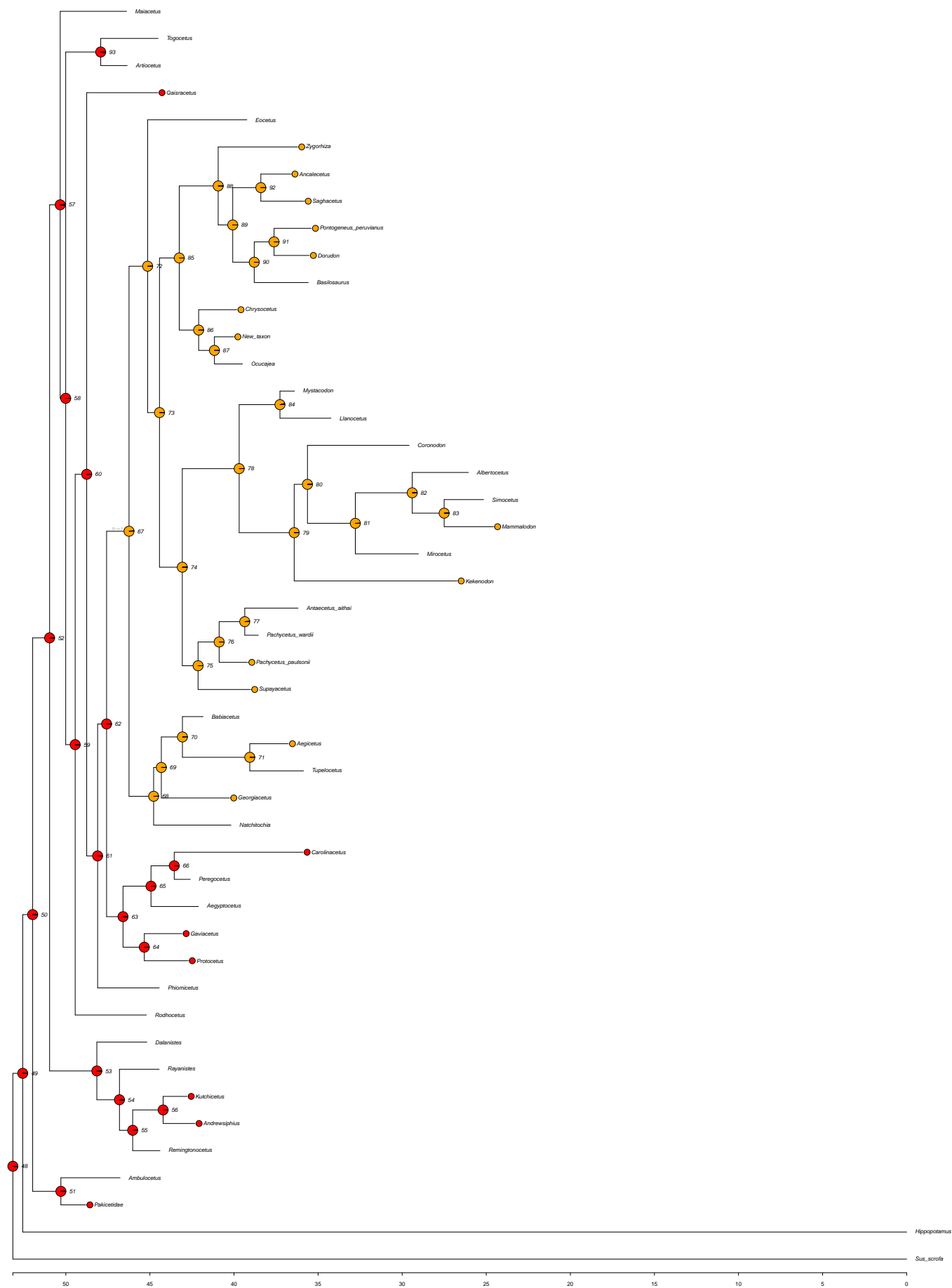

Supplement: Supplementary file 6 — Supplementary Data 3 [file 42003_2023_4986_MOESM6_ESM.zip › Supplementary Data 3/Supplementary Data 1_BTD_ASR/trait_0085_tree.plot.pdf]

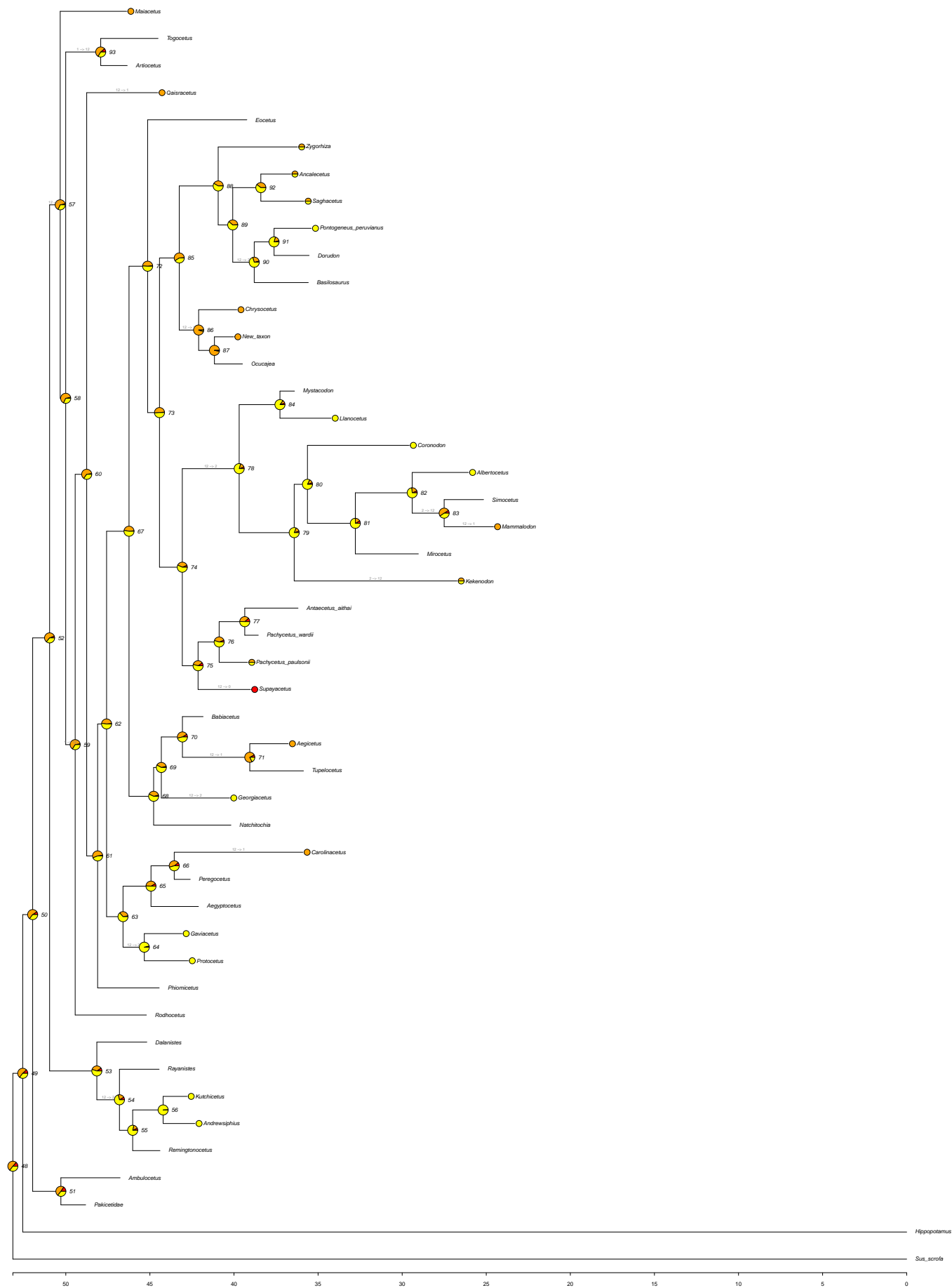

Supplement: Supplementary file 6 — Supplementary Data 3 [file 42003_2023_4986_MOESM6_ESM.zip › Supplementary Data 3/Supplementary Data 1_BTD_ASR/trait_0086_tree.plot.pdf]

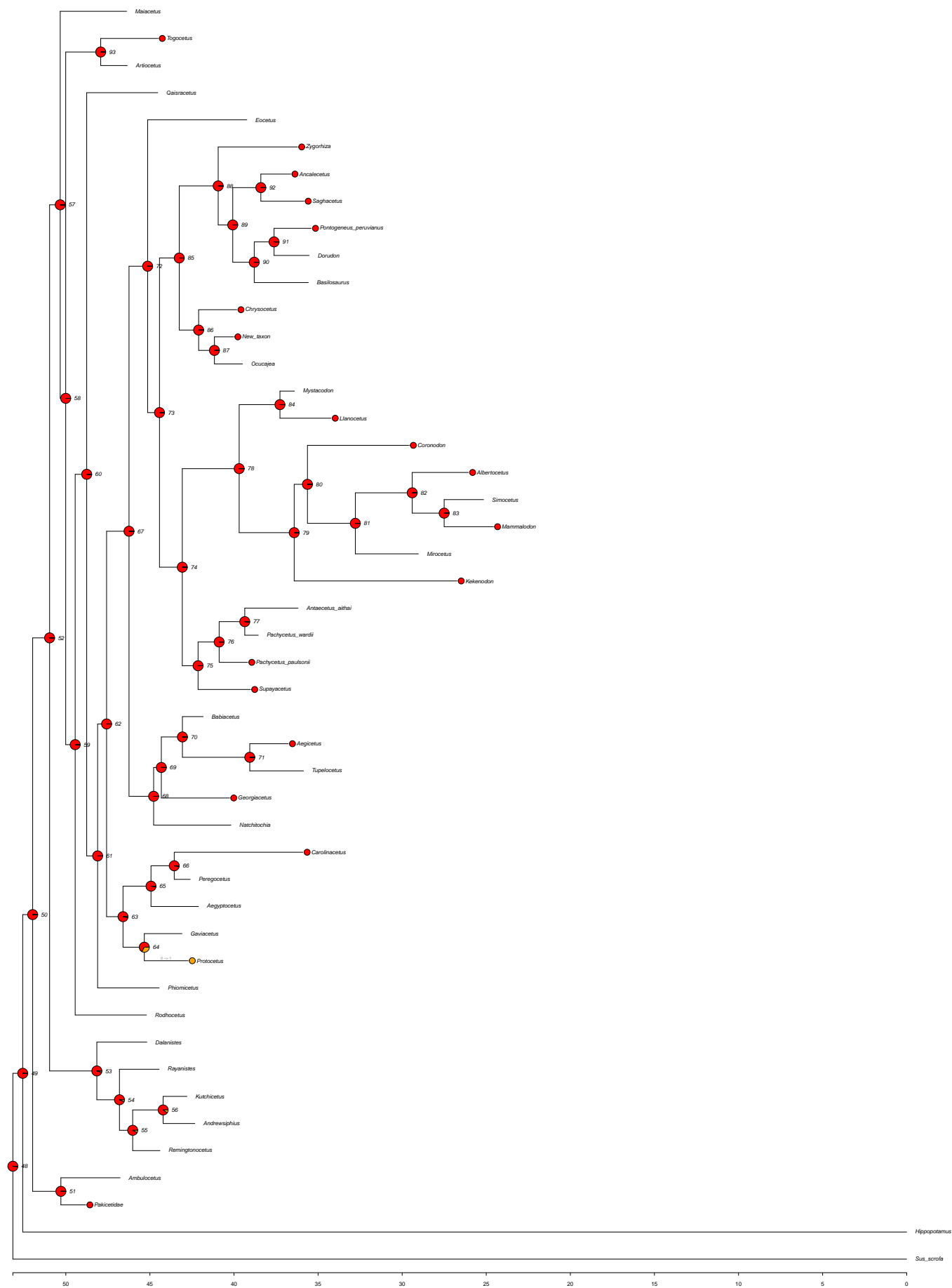

Supplement: Supplementary file 6 — Supplementary Data 3 [file 42003_2023_4986_MOESM6_ESM.zip › Supplementary Data 3/Supplementary Data 1_BTD_ASR/trait_0087_tree.plot.pdf]

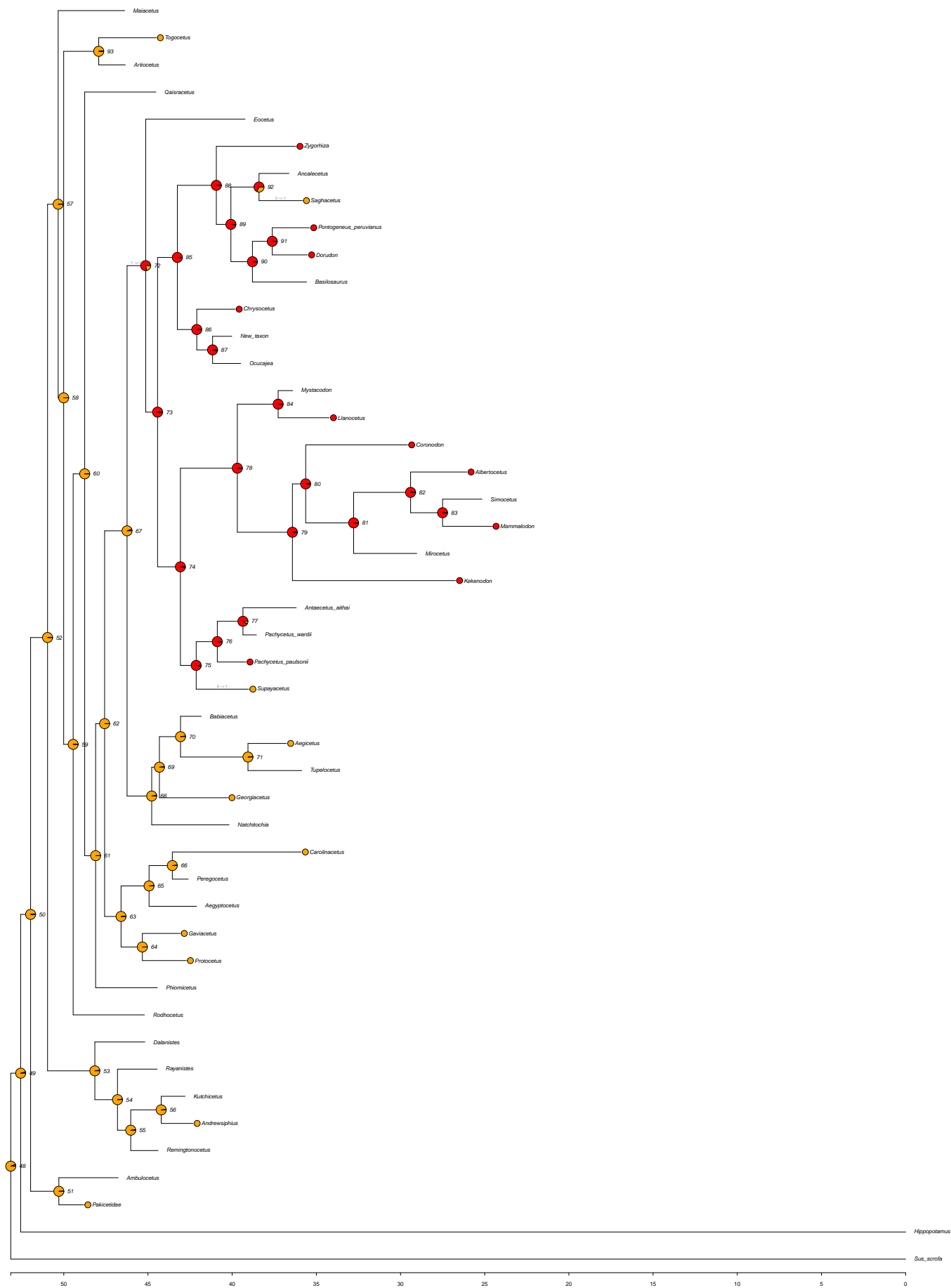

state 0 state 1

Supplement: Supplementary file 6 — Supplementary Data 3 [file 42003_2023_4986_MOESM6_ESM.zip › Supplementary Data 3/Supplementary Data 1_BTD_ASR/trait_0088_tree.plot.pdf]

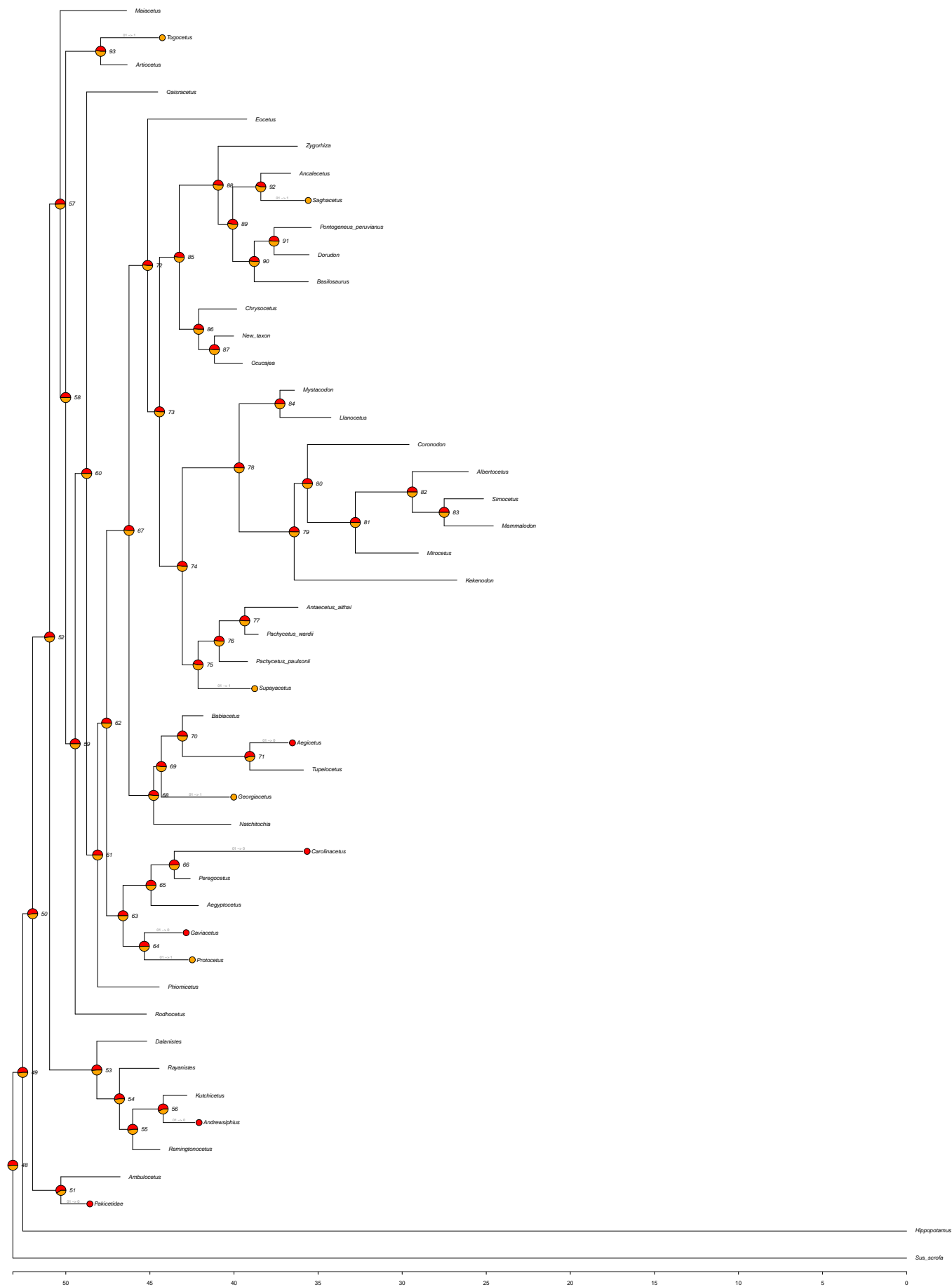

Supplement: Supplementary file 6 — Supplementary Data 3 [file 42003_2023_4986_MOESM6_ESM.zip › Supplementary Data 3/Supplementary Data 1_BTD_ASR/trait_0089_tree.plot.pdf]

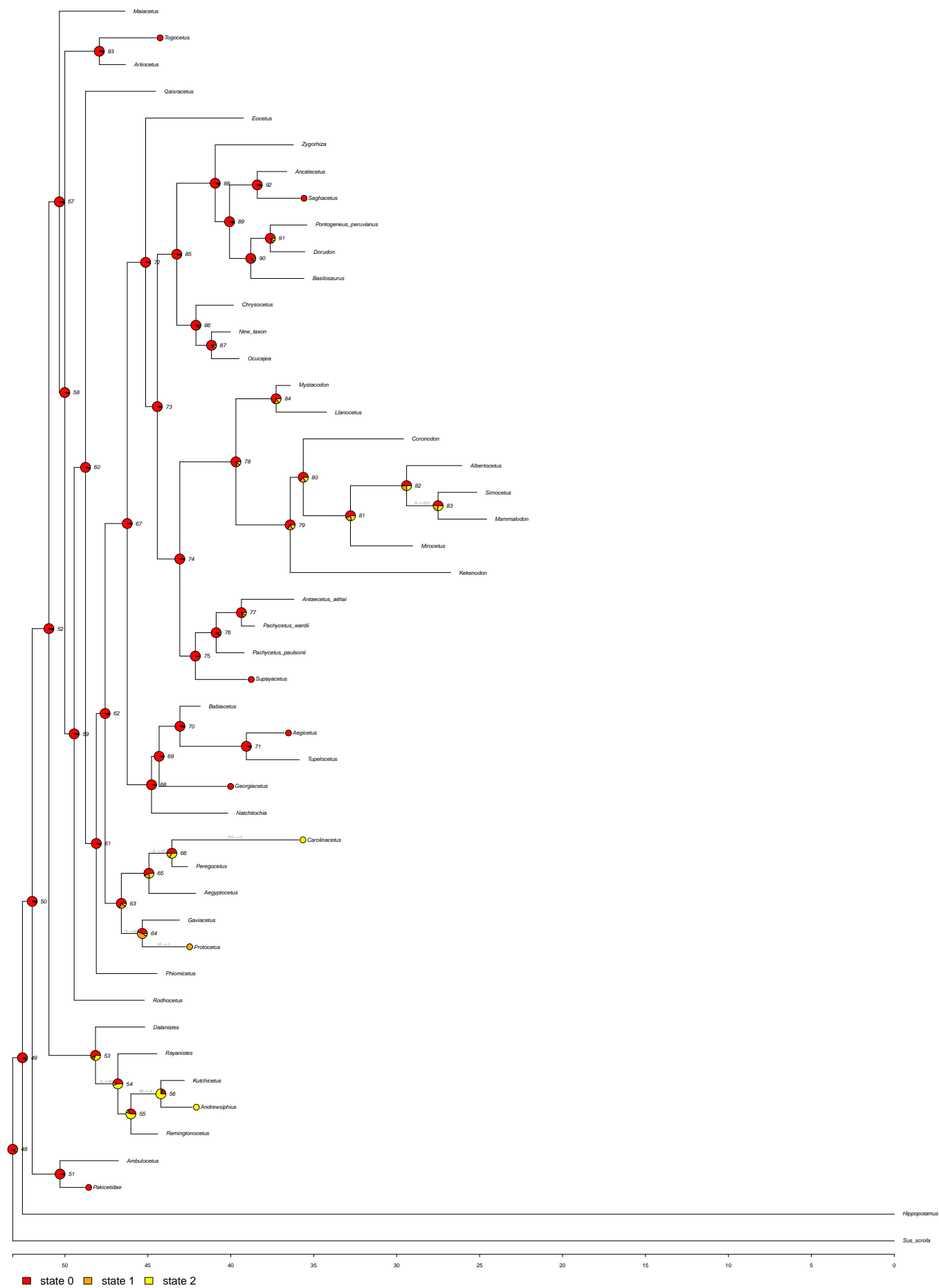

Supplement: Supplementary file 6 — Supplementary Data 3 [file 42003_2023_4986_MOESM6_ESM.zip › Supplementary Data 3/Supplementary Data 1_BTD_ASR/trait_0090_tree.plot.pdf]

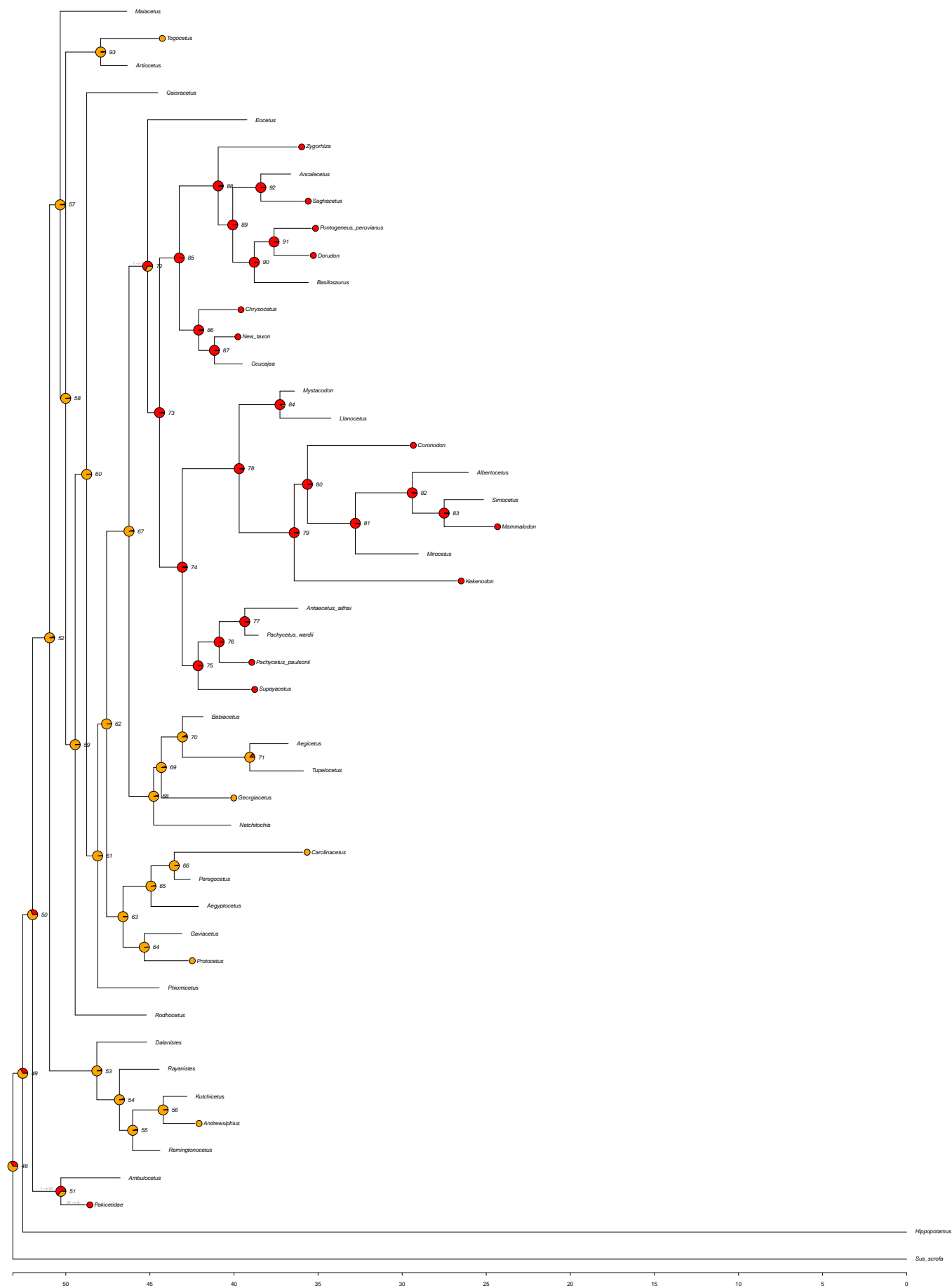

Supplement: Supplementary file 6 — Supplementary Data 3 [file 42003_2023_4986_MOESM6_ESM.zip › Supplementary Data 3/Supplementary Data 1_BTD_ASR/trait_0091_tree.plot.pdf]

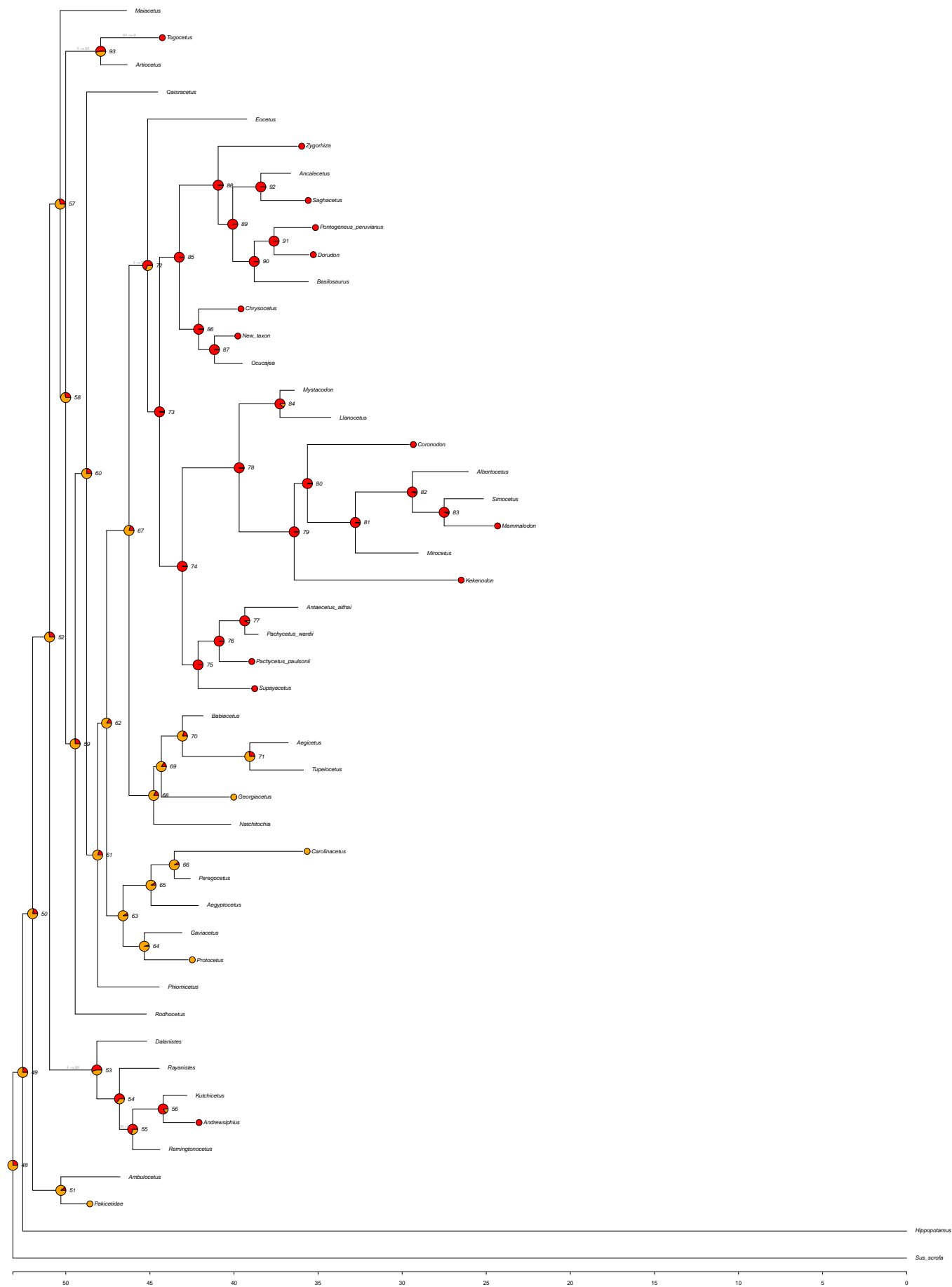

Supplement: Supplementary file 6 — Supplementary Data 3 [file 42003_2023_4986_MOESM6_ESM.zip › Supplementary Data 3/Supplementary Data 1_BTD_ASR/trait_0092_tree.plot.pdf]

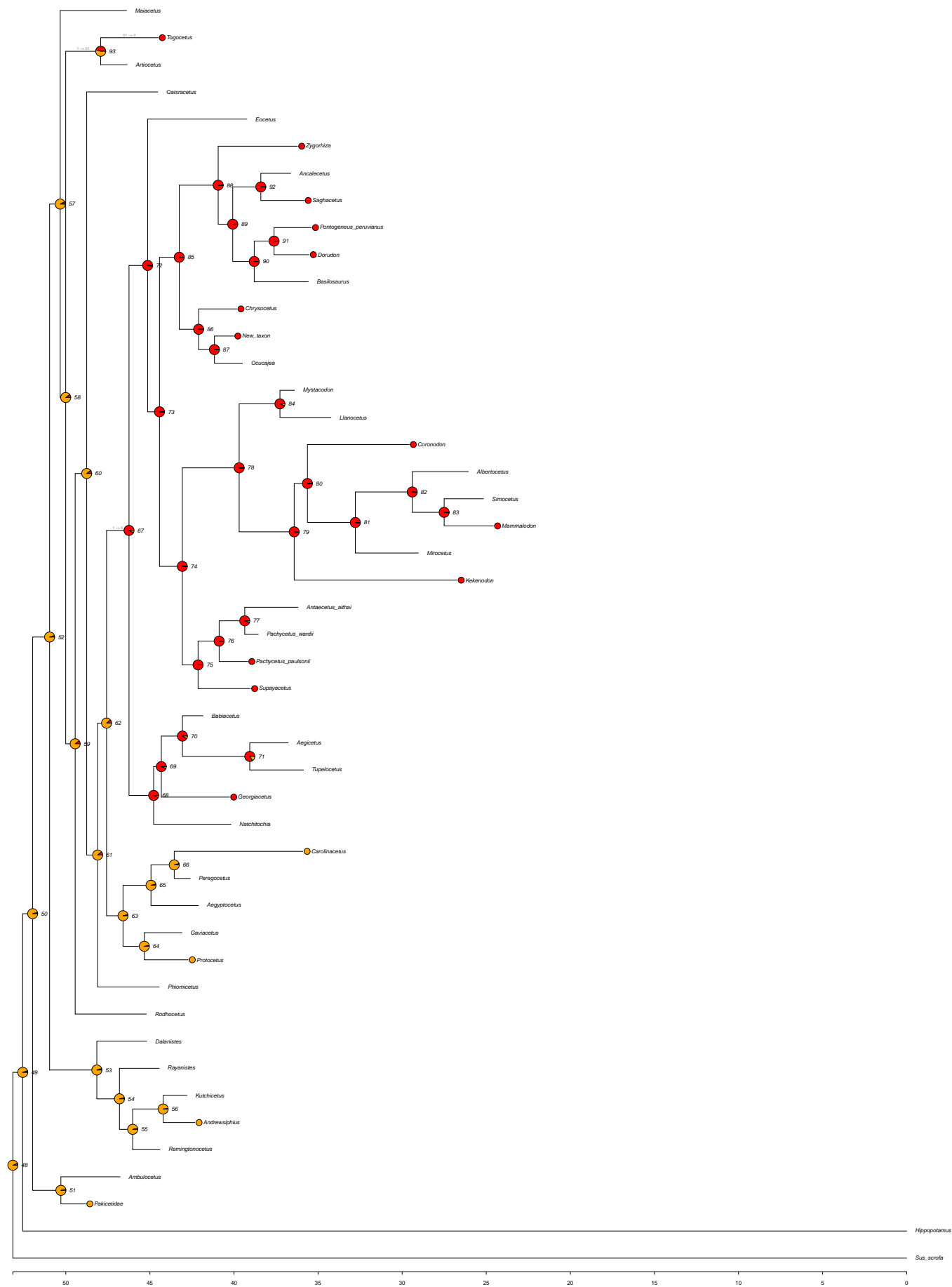

Supplement: Supplementary file 6 — Supplementary Data 3 [file 42003_2023_4986_MOESM6_ESM.zip › Supplementary Data 3/Supplementary Data 1_BTD_ASR/trait_0093_tree.plot.pdf]

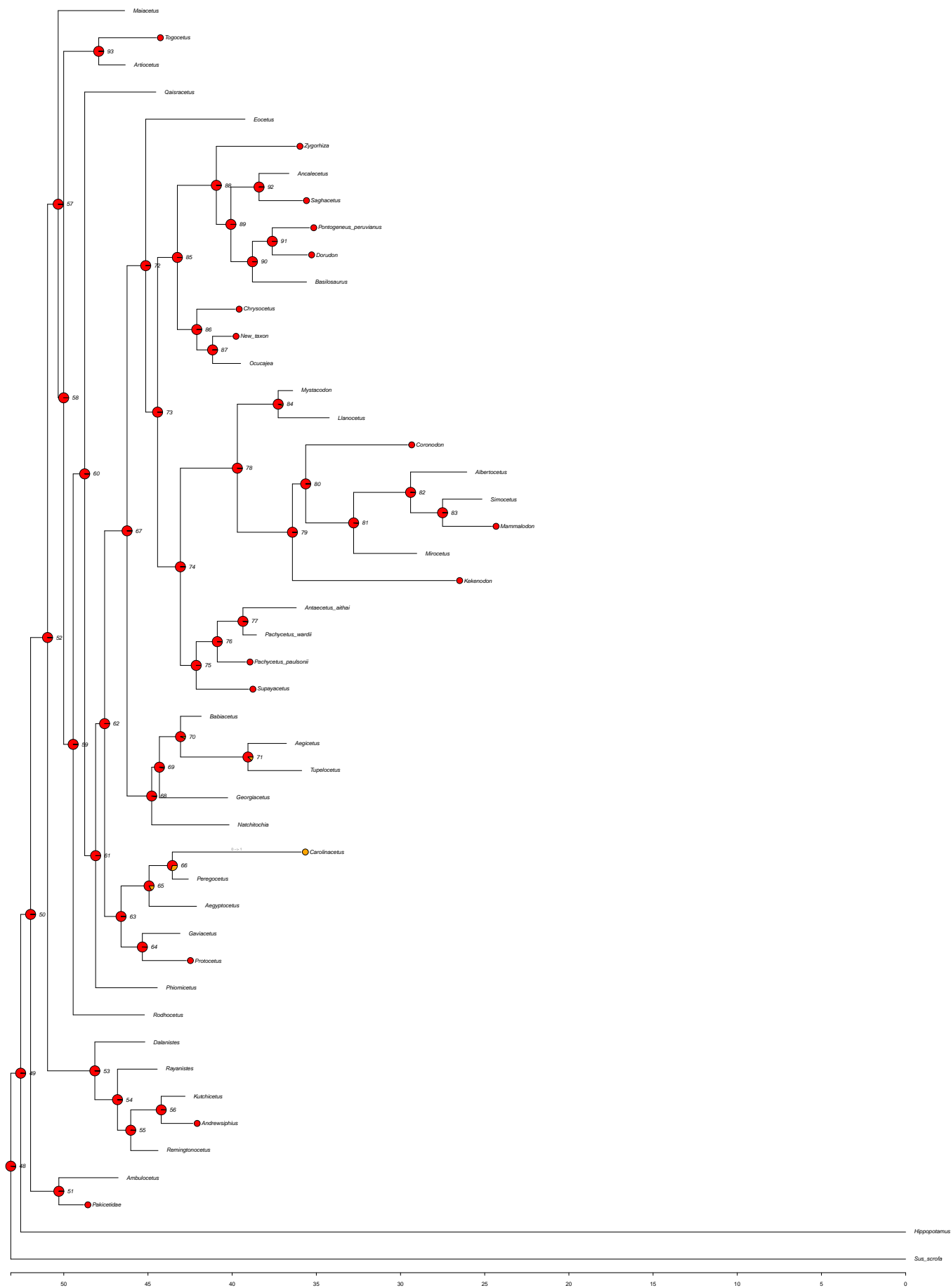

Supplement: Supplementary file 6 — Supplementary Data 3 [file 42003_2023_4986_MOESM6_ESM.zip › Supplementary Data 3/Supplementary Data 1_BTD_ASR/trait_0094_tree.plot.pdf]

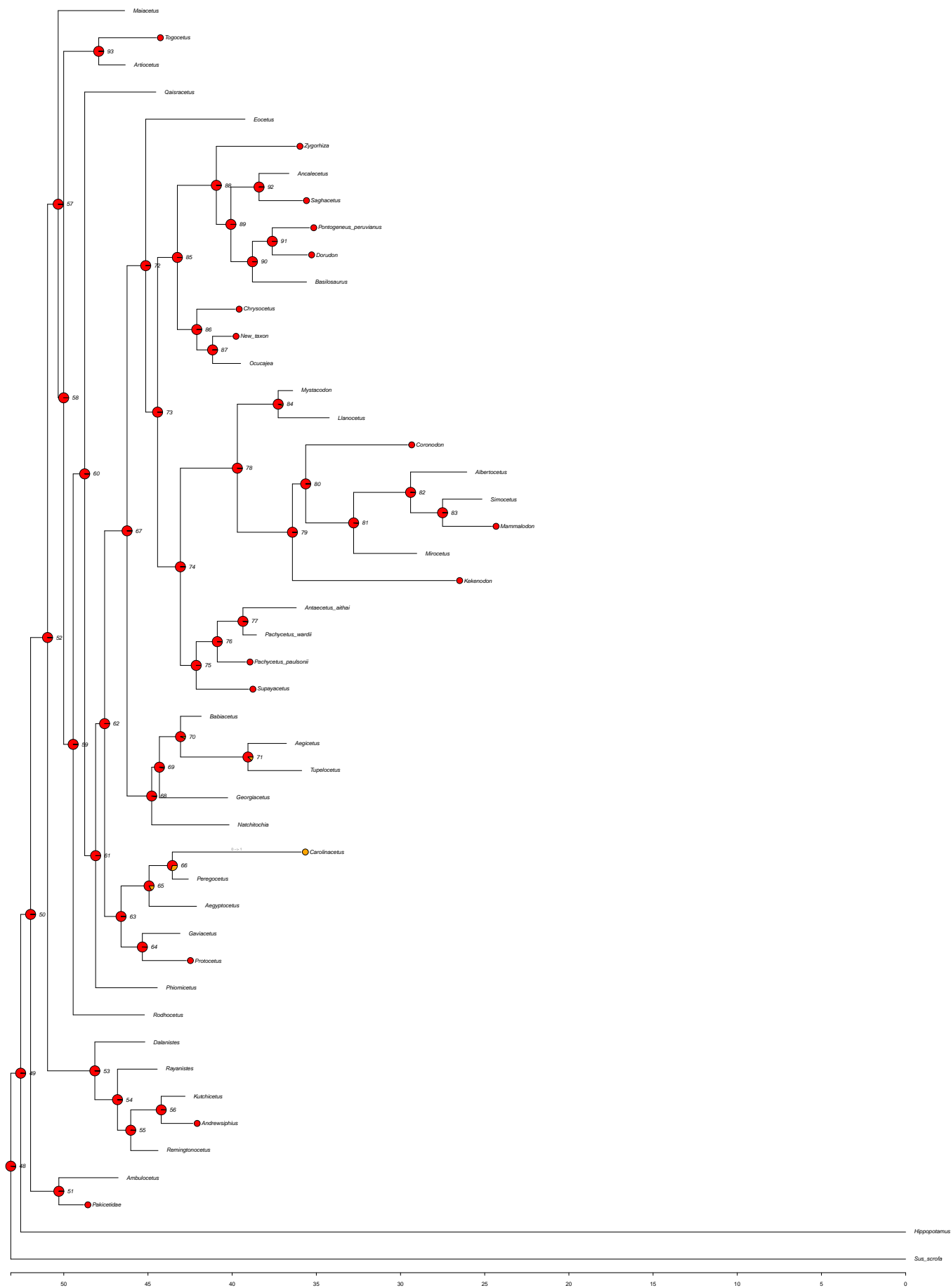

state 0 state 1

Supplement: Supplementary file 6 — Supplementary Data 3 [file 42003_2023_4986_MOESM6_ESM.zip › Supplementary Data 3/Supplementary Data 1_BTD_ASR/trait_0095_tree.plot.pdf]

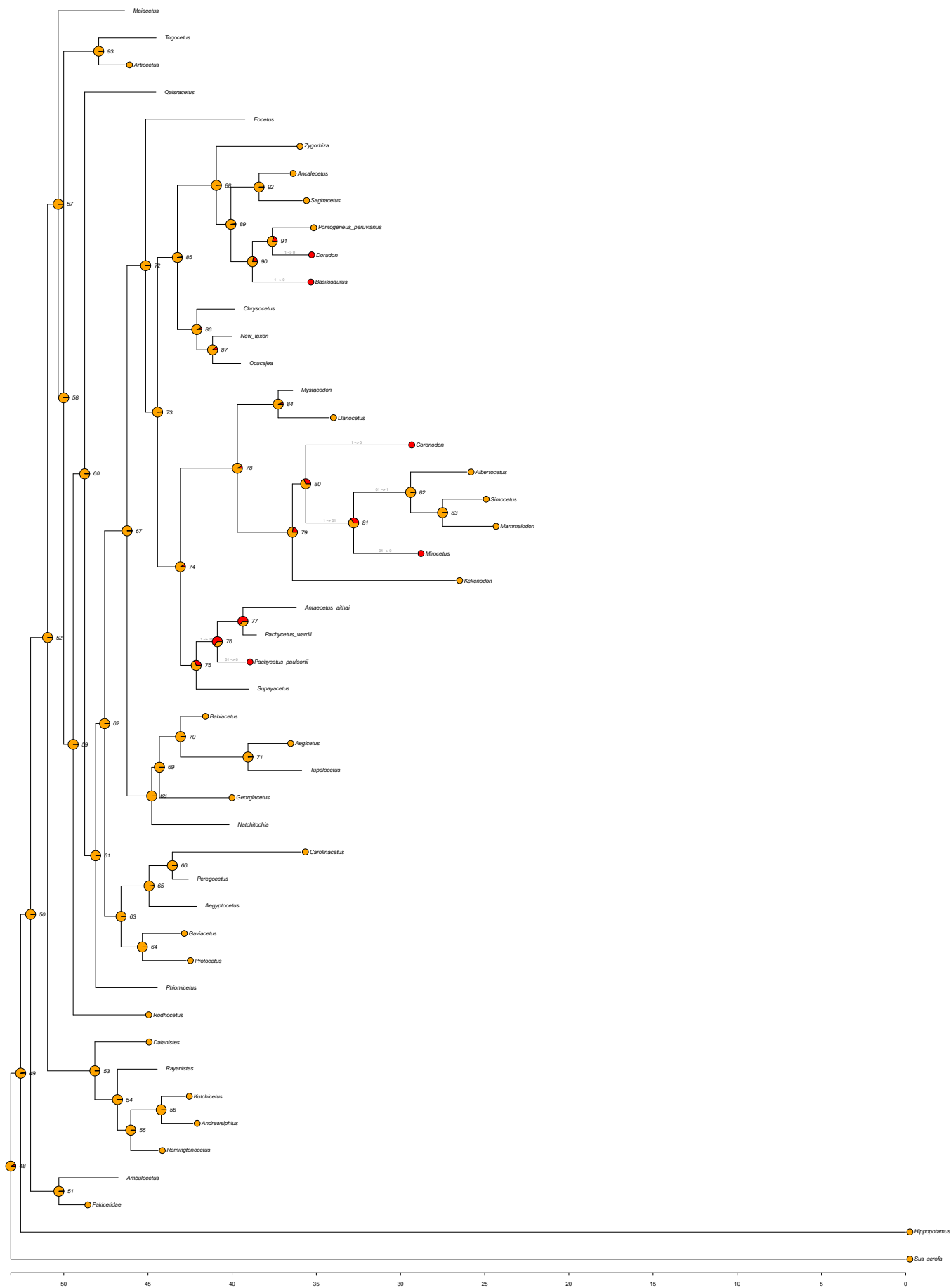

Supplement: Supplementary file 6 — Supplementary Data 3 [file 42003_2023_4986_MOESM6_ESM.zip › Supplementary Data 3/Supplementary Data 1_BTD_ASR/trait_0096_tree.plot.pdf]

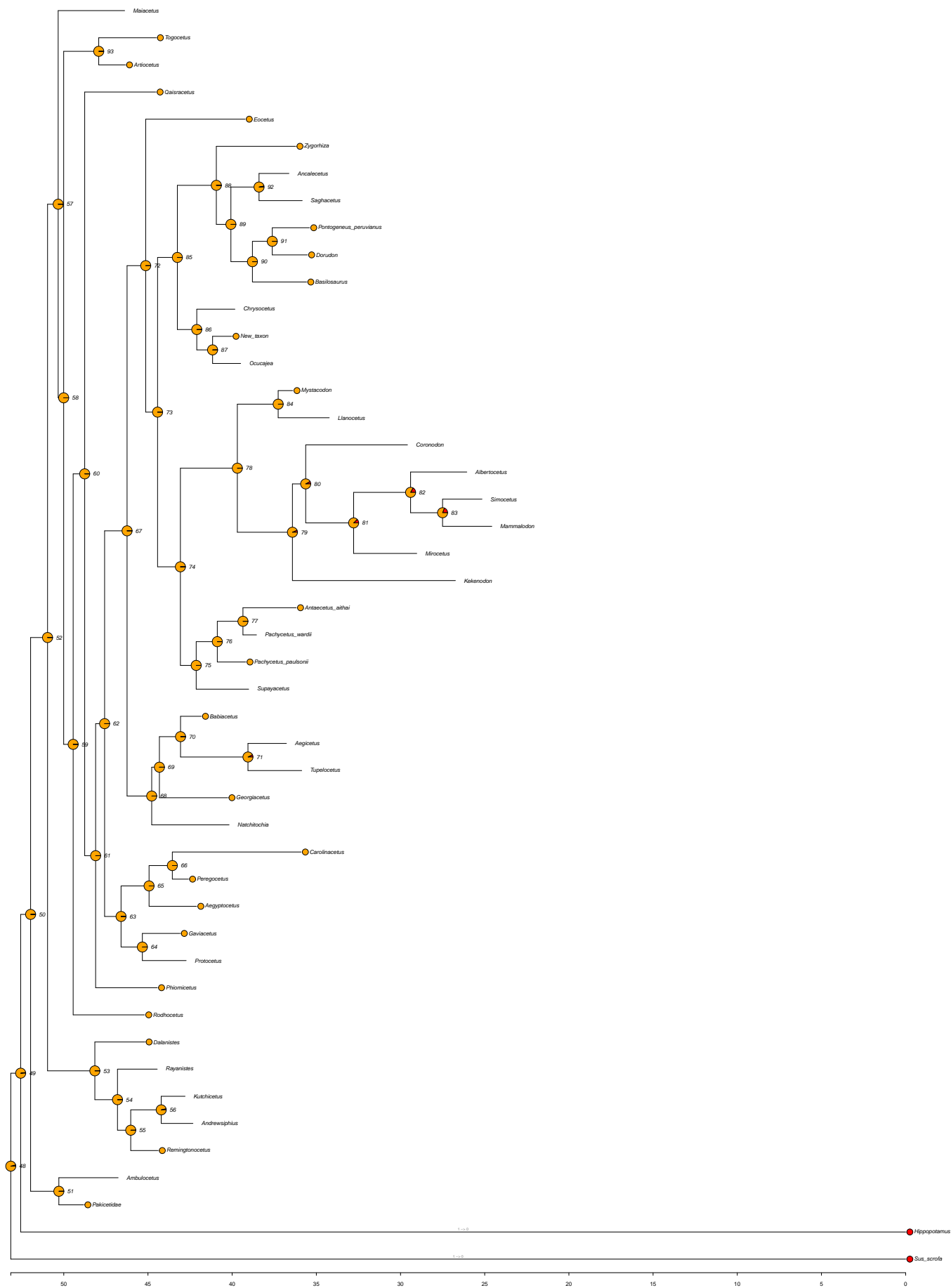

Supplement: Supplementary file 6 — Supplementary Data 3 [file 42003_2023_4986_MOESM6_ESM.zip › Supplementary Data 3/Supplementary Data 1_BTD_ASR/trait_0097_tree.plot.pdf]

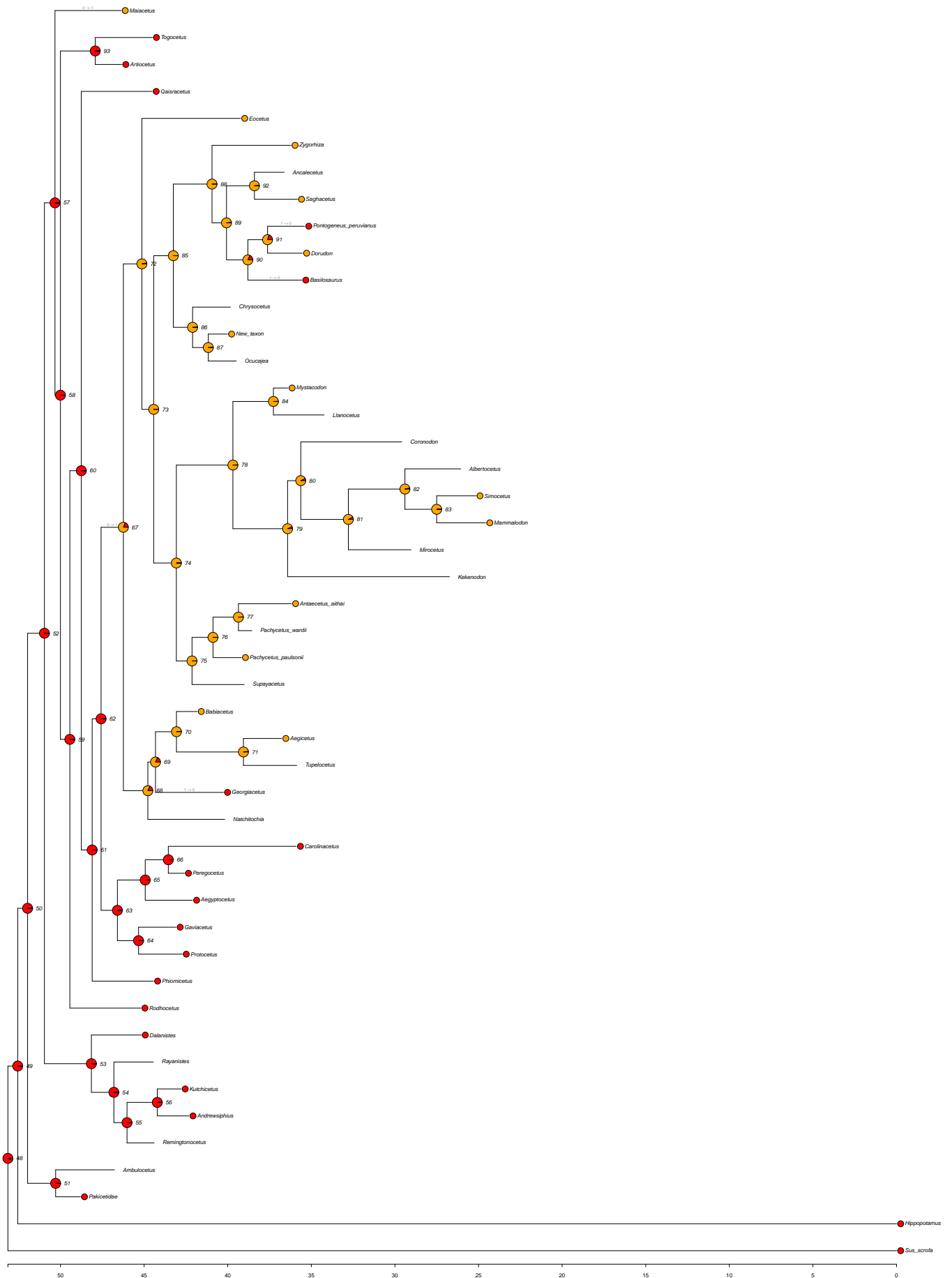

Supplement: Supplementary file 6 — Supplementary Data 3 [file 42003_2023_4986_MOESM6_ESM.zip › Supplementary Data 3/Supplementary Data 1_BTD_ASR/trait_0098_tree.plot.pdf]

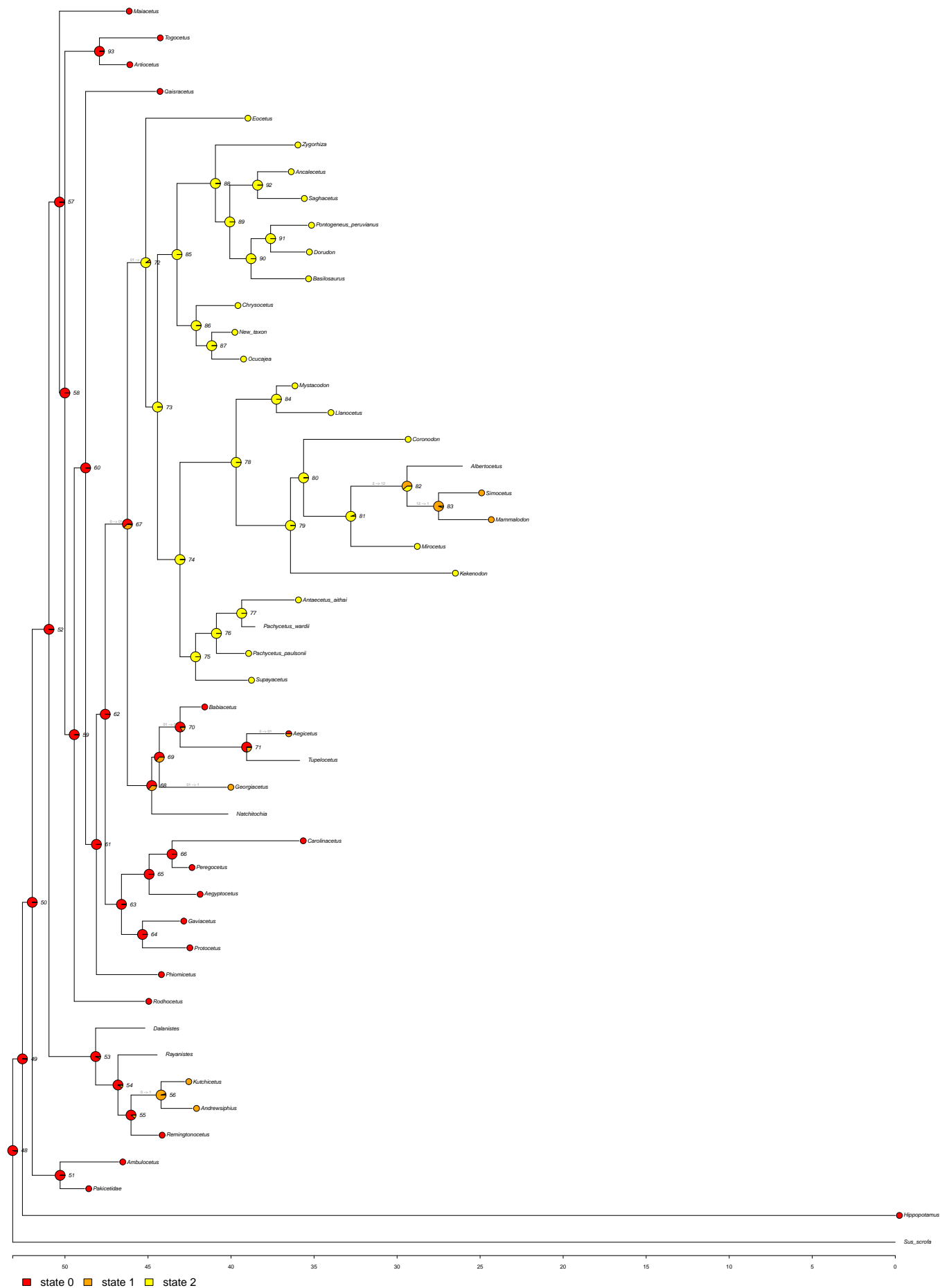

Supplement: Supplementary file 6 — Supplementary Data 3 [file 42003_2023_4986_MOESM6_ESM.zip › Supplementary Data 3/Supplementary Data 1_BTD_ASR/trait_0099_tree.plot.pdf]
