# Supplementary material for: A diminutive new basilosaurid whale reveals the trajectory of the cetacean life histories during the Eocene
Source: Commun Biol. 2023 Aug 10;6:707. doi: 10.1038/s42003-023-04986-w (PMC10415296; doi:10.1038/s42003-023-04986-w)
Supplement: Supplementary file 7 — Reporting Summary [file 42003_2023_4986_MOESM7_ESM.pdf]

nature portfolio

Corresponding author(s): Mohamed S. Antar

Last updated by author(s): May 13, 2023

Reporting Summary

Nature Portfolio wishes to improve the reproducibility of the work that we publish. This form provides structure for consistency and transparency in reporting. For further information on Nature Portfolio policies, see our Editorial Policies and the Editorial Policy Checklist.

Statistics

For all statistical analyses, confirm that the following items are present in the figure legend, table legend, main text, or Methods section.

n/a	Confirmed	
[x]	[ ]	The exact sample size (*n*) for each experimental group/condition, given as a discrete number and unit of measurement
[ ]	[x]	A statement on whether measurements were taken from distinct samples or whether the same sample was measured repeatedly
[x]	[ ]	The statistical test(s) used AND whether they are one- or two-sided<br>*Only common tests should be described solely by name; describe more complex techniques in the Methods section.*
[x]	[ ]	A description of all covariates tested
[x]	[ ]	A description of any assumptions or corrections, such as tests of normality and adjustment for multiple comparisons
[x]	[ ]	A full description of the statistical parameters including central tendency (e.g. means) or other basic estimates (e.g. regression coefficient) AND variation (e.g. standard deviation) or associated estimates of uncertainty (e.g. confidence intervals)
[x]	[ ]	For null hypothesis testing, the test statistic (e.g. *F*, *t*, *r*) with confidence intervals, effect sizes, degrees of freedom and *P* value noted<br>*Give P values as exact values whenever suitable.*
[ ]	[x]	For Bayesian analysis, information on the choice of priors and Markov chain Monte Carlo settings
[x]	[ ]	For hierarchical and complex designs, identification of the appropriate level for tests and full reporting of outcomes
[x]	[ ]	Estimates of effect sizes (e.g. Cohen's *d*, Pearson's *r*), indicating how they were calculated

Our web collection on statistics for biologists contains articles on many of the points above.

Software and code

Policy information about availability of computer code

Data collection	No data collection software was used in this study.
Data analysis	Observations on CT slices and 3D visualization were done using the software package Aviso 4.1.2 (Visage Imaging Inc., Chelmsford, MA). The phylogenetic and ancestral state reconstructions (ASRs) methods used in our study are extensively described in the Methods section of our main text and in our online supplement. The Bayesian tip-dating (BTD) analysis were conducted using MrBayes 3.2.7. For ancestral state reconstructions (ASRs) on the allcompat trees derived from BTD analyses, we used MBASR v. 2022.11.06. The 3D scanned data obtained from the Artec Leo scanner was processed using the post-processing software Artec Studio 17.

For manuscripts utilizing custom algorithms or software that are central to the research but not yet described in published literature, software must be made available to editors and reviewers. We strongly encourage code deposition in a community repository (e.g. GitHub). See the Nature Portfolio guidelines for submitting code & software for further information.

Data

Policy information about availability of data

All manuscripts must include a data availability statement. This statement should provide the following information, where applicable:

- Accession codes, unique identifiers, or web links for publicly available datasets
- A description of any restrictions on data availability
- For clinical datasets or third party data, please ensure that the statement adheres to our policy

All data supporting the findings of this study are available within the paper and its Supplementary Information files. Additionally, the data that support the findings of this study have been deposited in figshare and are available in three supplementary datasets: Supplementary Data 1 (Archaeocete-dominated matrix of Tutcetus in Nexus format), Supplementary Data 2 (Pelagicete-dominated matrix of Tutcetus in Nexus format), and Supplementary Data 3 (results from the ancestral state reconstructions on the allcompat trees derived from Bayesian tip-dating analyses in a Zip file). The figshare repository can be accessed at https://doi.org/10.6084/m9.figshare.22811183. The holotype specimen of Tutcetus (MUVP 501) is housed in the Mansoura University Vertebrate Paleontology Center (MUVP), Mansoura University, Egypt. All necessary permissions were obtained for the study of the specimens used in this research.

Human research participants

Policy information about studies involving human research participants and Sex and Gender in Research.

Reporting on sex and gender	N/A
Population characteristics	N/A
Recruitment	N/A
Ethics oversight	N/A

Note that full information on the approval of the study protocol must also be provided in the manuscript.

Field-specific reporting

Please select the one below that is the best fit for your research. If you are not sure, read the appropriate sections before making your selection.

☒ Life sciences ☐ Behavioural & social sciences ☐ Ecological, evolutionary & environmental sciences

For a reference copy of the document with all sections, see nature.com/documents/nr-reporting-summary-flat.pdf

Life sciences study design

All studies must disclose on these points even when the disclosure is negative.

Sample size	The new basilosaurid whale taxon described in this study is represented by a single partial skeleton, MUVP 501.
Data exclusions	No relevant data were excluded from the analysis.
Replication	No experiments (in the strict sense of the word) were performed; hence, this field does not apply to our study.
Randomization	There were no experimental groups; hence, this field does not apply.
Blinding	There was no group allocation; hence, this field does not apply.

Reporting for specific materials, systems and methods

We require information from authors about some types of materials, experimental systems and methods used in many studies. Here, indicate whether each material, system or method listed is relevant to your study. If you are not sure if a list item applies to your research, read the appropriate section before selecting a response.

Materials & experimental systems

n/a	Involved in the study
☒	☐ Antibodies
☒	☐ Eukaryotic cell lines
☐	☒ Palaeontology and archaeology
☒	☐ Animals and other organisms
☒	☐ Clinical data
☒	☐ Dual use research of concern

Methods

n/a	Involved in the study
☒	☐ ChIP-seq
☒	☐ Flow cytometry
☒	☐ MRI-based neuroimaging

Palaeontology and Archaeology

Specimen provenance: Wadi El-Rayan valley (40 km northeast of Wadi El-Hitan World Heritage Site) of the Fayum Depression in the Western Desert of Egypt.

Specimen deposition: Mansoura University Vertebrate Paleontology Center (MUVP), Mansoura University, Egypt.

Dating methods: No additional dating procedures were taken in this study.

☒ Tick this box to confirm that the raw and calibrated dates are available in the paper or in Supplementary Information.

Ethics oversight: Mansoura University, Mansoura, Egypt

Note that full information on the approval of the study protocol must also be provided in the manuscript.
